# Supplementary material for: HIVprotI: an integrated web based platform for prediction and design of HIV proteins inhibitors
Source: J Cheminform. 2018 Mar 9;10:12. doi: 10.1186/s13321-018-0266-y (PMC5845081; doi:10.1186/s13321-018-0266-y)
Supplement: Supplementary file 2 — Additional file 2. Source code of HIVProtI web server. [file 13321_2018_266_MOESM2_ESM.zip › HIVprotI_Source-code/hivprotI/weka-3-6-6/doc/serialized-form.html]

 
 
 
 
 
 
Serialized Form
 

 

 

 
function windowTitle()
{
    if (location.href.indexOf('is-external=true') == -1) {
        parent.document.title="Serialized Form";
    }
}
 
 
 

 

 
 


 
   
  
 
 
 
   
 
   
          Overview     
        Package   
        Class   
          Tree     
          Deprecated     
          Index     
          Help           Weka's home     
   
 
 
  
 
 
 

 
  
 PREV 
 NEXT  
  
    FRAMES     
   NO FRAMES     
  
    All Classes  ');
  }
  //-->
 
 
    All Classes  
 


  
 
 
  
 

 
 
 
Serialized Form 
 
 

 
 
  
 Package   weka.associations   
 
 

 
   
 
 
  
 Class  weka.associations.AbstractAssociator  extends java.lang.Object implements Serializable   
 
 

 
 serialVersionUID:  -3017644543382432070L

 

 
   
 
 
  
 Class  weka.associations.Apriori  extends  AbstractAssociator  implements Serializable   
 
 

 
 serialVersionUID:  3277498842319212687L

 
   
 
 
  
 Serialized Fields   
 
 

 
m_minSupport 
 
double  m_minSupport  
 
 The minimum support.
 
 
 
 
 
 
m_upperBoundMinSupport 
 
double  m_upperBoundMinSupport  
 
 The upper bound on the support
 
 
 
 
 
 
m_lowerBoundMinSupport 
 
double  m_lowerBoundMinSupport  
 
 The lower bound for the minimum support.
 
 
 
 
 
 
m_metricType 
 
int  m_metricType  
 
 The selected metric type.
 
 
 
 
 
 
m_minMetric 
 
double  m_minMetric  
 
 The minimum metric score.
 
 
 
 
 
 
m_numRules 
 
int  m_numRules  
 
 The maximum number of rules that are output.
 
 
 
 
 
 
m_delta 
 
double  m_delta  
 
 Delta by which m_minSupport is decreased in each iteration.
 
 
 
 
 
 
m_significanceLevel 
 
double  m_significanceLevel  
 
 Significance level for optional significance test.
 
 
 
 
 
 
m_cycles 
 
int  m_cycles  
 
 Number of cycles used before required number of rules was one.
 
 
 
 
 
 
m_Ls 
 
 FastVector   m_Ls  
 
 The set of all sets of itemsets L.
 
 
 
 
 
 
m_hashtables 
 
 FastVector   m_hashtables  
 
 The same information stored in hash tables.
 
 
 
 
 
 
m_allTheRules 
 
 FastVector []  m_allTheRules  
 
 The list of all generated rules.
 
 
 
 
 
 
m_instances 
 
 Instances   m_instances  
 
 The instances (transactions) to be used for generating 
      the association rules.
 
 
 
 
 
 
m_outputItemSets 
 
boolean  m_outputItemSets  
 
 Output itemsets found?
 
 
 
 
 
 
m_removeMissingCols 
 
boolean  m_removeMissingCols  
 
 Remove columns with all missing values
 
 
 
 
 
 
m_verbose 
 
boolean  m_verbose  
 
 Report progress iteratively
 
 
 
 
 
 
m_onlyClass 
 
 Instances   m_onlyClass  
 
 Only the class attribute of all Instances.
 
 
 
 
 
 
m_classIndex 
 
int  m_classIndex  
 
 The class index.
 
 
 
 
 
 
m_car 
 
boolean  m_car  
 
 Flag indicating whether class association rules are mined.
 
 
 
 

 
   
 
 
  
 Class  weka.associations.AprioriItemSet  extends  ItemSet  implements Serializable   
 
 

 
 serialVersionUID:  7684467755712672058L

 

 
   
 
 
  
 Class  weka.associations.CaRuleGeneration  extends  RuleGeneration  implements Serializable   
 
 

 
 serialVersionUID:  3065752149646517703L

 

 
   
 
 
  
 Class  weka.associations.FilteredAssociator  extends  SingleAssociatorEnhancer  implements Serializable   
 
 

 
 serialVersionUID:  -4523450618538717400L

 
   
 
 
  
 Serialized Fields   
 
 

 
m_Filter 
 
 Filter   m_Filter  
 
 The filter
 
 
 
 
 
 
m_FilteredInstances 
 
 Instances   m_FilteredInstances  
 
 The instance structure of the filtered instances
 
 
 
 
 
 
m_ClassIndex 
 
int  m_ClassIndex  
 
 The class index.
 
 
 
 

 
   
 
 
  
 Class  weka.associations.FPGrowth  extends  AbstractAssociator  implements Serializable   
 
 

 
 serialVersionUID:  3620717108603442911L

 
   
 
 
  
 Serialized Fields   
 
 

 
m_numRulesToFind 
 
int  m_numRulesToFind  
 
 The number of rules to find
 
 
 
 
 
 
m_upperBoundMinSupport 
 
double  m_upperBoundMinSupport  
 
 The upper bound on the minimum support
 
 
 
 
 
 
m_lowerBoundMinSupport 
 
double  m_lowerBoundMinSupport  
 
 The lower bound on minimum support
 
 
 
 
 
 
m_delta 
 
double  m_delta  
 
 The amount by which to decrease the support in each iteration
 
 
 
 
 
 
m_findAllRulesForSupportLevel 
 
boolean  m_findAllRulesForSupportLevel  
 
 If true, just all rules meeting the lower bound on the minimum
 support will be found. The number of rules to find will be
 ignored and the iterative reduction of support will not
 be done.
 
 
 
 
 
 
m_positiveIndex 
 
int  m_positiveIndex  
 
 The index (1 based) of binary attributes to treat as the positive value
 
 
 
 
 
 
m_metric 
 
 FPGrowth.AssociationRule.METRIC_TYPE   m_metric  
 
 
 
 
 
 
m_metricThreshold 
 
double  m_metricThreshold  
 
 
 
 
 
 
m_largeItemSets 
 
weka.associations.FPGrowth.FrequentItemSets  m_largeItemSets  
 
 Holds the large item sets found
 
 
 
 
 
 
m_rules 
 
java.util.List<E>  m_rules  
 
 Holds the rules
 
 
 
 
 
 
m_maxItems 
 
int  m_maxItems  
 
 
 
 
 
 
m_transactionsMustContain 
 
java.lang.String  m_transactionsMustContain  
 
 If set, limit the transactions (instances) input to the
  algorithm to those that contain these items
 
 
 
 
 
 
m_mustContainOR 
 
boolean  m_mustContainOR  
 
 Use OR rather than AND when considering must contain lists
 
 
 
 
 
 
m_rulesMustContain 
 
java.lang.String  m_rulesMustContain  
 
 If set, then only output rules containing these itmes
 
 
 
 

 
   
 
 
  
 Class  weka.associations.FPGrowth.AssociationRule  extends java.lang.Object implements Serializable   
 
 

 
 serialVersionUID:  -661269018702294489L

 
   
 
 
  
 Serialized Fields   
 
 

 
m_metricType 
 
 FPGrowth.AssociationRule.METRIC_TYPE   m_metricType  
 
 The metric type for this rule
 
 
 
 
 
 
m_premise 
 
java.util.Collection<E>  m_premise  
 
 The premise of the rule
 
 
 
 
 
 
m_consequence 
 
java.util.Collection<E>  m_consequence  
 
 The consequence of the rule
 
 
 
 
 
 
m_premiseSupport 
 
int  m_premiseSupport  
 
 The support for the premise
 
 
 
 
 
 
m_consequenceSupport 
 
int  m_consequenceSupport  
 
 The support for the consequence
 
 
 
 
 
 
m_totalSupport 
 
int  m_totalSupport  
 
 The total support for the item set (premise + consequence)
 
 
 
 
 
 
m_totalTransactions 
 
int  m_totalTransactions  
 
 The total number of transactions in the data
 
 
 
 

 
   
 
 
  
 Class  weka.associations.FPGrowth.BinaryItem  extends java.lang.Object implements Serializable   
 
 

 
 serialVersionUID:  -3372941834914147669L

 
   
 
 
  
 Serialized Fields   
 
 

 
m_frequency 
 
int  m_frequency  
 
 The frequency of the item
 
 
 
 
 
 
m_attribute 
 
 Attribute   m_attribute  
 
 The attribute that the item corresponds to
 
 
 
 
 
 
m_valueIndex 
 
int  m_valueIndex  
 
 The index of the value considered to be positive
 
 
 
 

 
   
 
 
  
 Class weka.associations.FPGrowth.FPTreeNode extends java.lang.Object implements Serializable   
 
 

 
 serialVersionUID:  4396315323673737660L

 
   
 
 
  
 Serialized Fields   
 
 

 
m_levelSibling 
 
weka.associations.FPGrowth.FPTreeNode  m_levelSibling  
 
 link to another sibling at this level in the tree
 
 
 
 
 
 
m_parent 
 
weka.associations.FPGrowth.FPTreeNode  m_parent  
 
 link to the parent node
 
 
 
 
 
 
m_item 
 
 FPGrowth.BinaryItem   m_item  
 
 item at this node
 
 
 
 
 
 
m_ID 
 
int  m_ID  
 
 ID (for graphing the tree)
 
 
 
 
 
 
m_children 
 
java.util.Map<K,V>  m_children  
 
 the children of this node
 
 
 
 
 
 
m_projectedCounts 
 
weka.associations.FPGrowth.ShadowCounts  m_projectedCounts  
 
 counts associated with projected versions of this node
 
 
 
 

 
   
 
 
  
 Class weka.associations.FPGrowth.FPTreeRoot.Header extends java.lang.Object implements Serializable   
 
 

 
 serialVersionUID:  -6583156284891368909L

 
   
 
 
  
 Serialized Fields   
 
 

 
m_headerList 
 
java.util.List<E>  m_headerList  
 
 The list of pointers into the tree structure
 
 
 
 
 
 
m_projectedHeaderCounts 
 
weka.associations.FPGrowth.ShadowCounts  m_projectedHeaderCounts  
 
 Projected header counts for this entry
 
 
 
 

 
   
 
 
  
 Class weka.associations.FPGrowth.FrequentBinaryItemSet extends java.lang.Object implements Serializable   
 
 

 
 serialVersionUID:  -6543815873565829448L

 
   
 
 
  
 Serialized Fields   
 
 

 
m_items 
 
java.util.ArrayList<E>  m_items  
 
 The list of items in the item set
 
 
 
 
 
 
m_support 
 
int  m_support  
 
 the support of this item set
 
 
 
 

 
   
 
 
  
 Class weka.associations.FPGrowth.FrequentItemSets extends java.lang.Object implements Serializable   
 
 

 
 serialVersionUID:  4173606872363973588L

 
   
 
 
  
 Serialized Fields   
 
 

 
m_sets 
 
java.util.ArrayList<E>  m_sets  
 
 The list of frequent item sets
 
 
 
 
 
 
m_numberOfTransactions 
 
int  m_numberOfTransactions  
 
 The total number of transactions in the data
 
 
 
 

 
   
 
 
  
 Class weka.associations.FPGrowth.ShadowCounts extends java.lang.Object implements Serializable   
 
 

 
 serialVersionUID:  4435433714185969155L

 
   
 
 
  
 Serialized Fields   
 
 

 
m_counts 
 
java.util.ArrayList<E>  m_counts  
 
 Holds the counts at different recursion levels
 
 
 
 

 
   
 
 
  
 Class  weka.associations.GeneralizedSequentialPatterns  extends  AbstractAssociator  implements Serializable   
 
 

 
 serialVersionUID:  -4119691320812254676L

 
   
 
 
  
 Serialized Fields   
 
 

 
m_MinSupport 
 
double  m_MinSupport  
 
 the minimum support threshold
 
 
 
 
 
 
m_DataSeqID 
 
int  m_DataSeqID  
 
 number indicating the attribute holding the data sequence ID
 
 
 
 
 
 
m_OriginalDataSet 
 
 Instances   m_OriginalDataSet  
 
 original sequential data set to be used for sequential patterns extraction
 
 
 
 
 
 
m_AllSequentialPatterns 
 
 FastVector   m_AllSequentialPatterns  
 
 all generated frequent sequences, i.e. sequential patterns
 
 
 
 
 
 
m_Cycles 
 
int  m_Cycles  
 
 number of cycles performed until termination
 
 
 
 
 
 
m_CycleStart 
 
java.lang.String  m_CycleStart  
 
 String indicating the starting time of an cycle.
 
 
 
 
 
 
m_CycleEnd 
 
java.lang.String  m_CycleEnd  
 
 String indicating the ending time of an cycle.
 
 
 
 
 
 
m_AlgorithmStart 
 
java.lang.String  m_AlgorithmStart  
 
 String indicating the starting time of the algorithm.
 
 
 
 
 
 
m_FilterAttributes 
 
java.lang.String  m_FilterAttributes  
 
 String containing the attribute numbers that are used for result 
 filtering; -1 means no filtering
 
 
 
 
 
 
m_FilterAttrVector 
 
 FastVector   m_FilterAttrVector  
 
 Vector containing the attribute numbers that are used for result 
 filtering; -1 means no filtering
 
 
 
 
 
 
m_Debug 
 
boolean  m_Debug  
 
 Whether the classifier is run in debug mode.
 
 
 
 

 
   
 
 
  
 Class  weka.associations.ItemSet  extends java.lang.Object implements Serializable   
 
 

 
 serialVersionUID:  2724000045282835791L

 
   
 
 
  
 Serialized Fields   
 
 

 
m_items 
 
int[]  m_items  
 
 The items stored as an array of of ints.
 
 
 
 
 
 
m_counter 
 
int  m_counter  
 
 Counter for how many transactions contain this item set.
 
 
 
 
 
 
m_totalTransactions 
 
int  m_totalTransactions  
 
 The total number of transactions
 
 
 
 

 
   
 
 
  
 Class  weka.associations.LabeledItemSet  extends  ItemSet  implements Serializable   
 
 

 
 serialVersionUID:  4158771925518299903L

 
   
 
 
  
 Serialized Fields   
 
 

 
m_classLabel 
 
int  m_classLabel  
 
 The class label.
 
 
 
 
 
 
m_ruleSupCounter 
 
int  m_ruleSupCounter  
 
 The support of the rule.
 
 
 
 

 
   
 
 
  
 Class  weka.associations.PredictiveApriori  extends  AbstractAssociator  implements Serializable   
 
 

 
 serialVersionUID:  8109088846865075341L

 
   
 
 
  
 Serialized Fields   
 
 

 
m_premiseCount 
 
int  m_premiseCount  
 
 The minimum support.
 
 
 
 
 
 
m_numRules 
 
int  m_numRules  
 
 The maximum number of rules that are output.
 
 
 
 
 
 
m_Ls 
 
 FastVector   m_Ls  
 
 The set of all sets of itemsets.
 
 
 
 
 
 
m_hashtables 
 
 FastVector   m_hashtables  
 
 The same information stored in hash tables.
 
 
 
 
 
 
m_allTheRules 
 
 FastVector []  m_allTheRules  
 
 The list of all generated rules.
 
 
 
 
 
 
m_instances 
 
 Instances   m_instances  
 
 The instances (transactions) to be used for generating 
      the association rules.
 
 
 
 
 
 
m_priors 
 
java.util.Hashtable<K,V>  m_priors  
 
 The hashtable containing the prior probabilities.
 
 
 
 
 
 
m_midPoints 
 
double[]  m_midPoints  
 
 The mid points of the intervals used for the prior estimation.
 
 
 
 
 
 
m_expectation 
 
double  m_expectation  
 
 The expected predictive accuracy a rule needs to be a candidate for the output.
 
 
 
 
 
 
m_best 
 
java.util.TreeSet<E>  m_best  
 
 The n best rules.
 
 
 
 
 
 
m_bestChanged 
 
boolean  m_bestChanged  
 
 Flag keeping track if the list of the n best rules has changed.
 
 
 
 
 
 
m_count 
 
int  m_count  
 
 Counter for the time of generation for an association rule.
 
 
 
 
 
 
m_priorEstimator 
 
 PriorEstimation   m_priorEstimator  
 
 The prior estimator.
 
 
 
 
 
 
m_classIndex 
 
int  m_classIndex  
 
 The class index.
 
 
 
 
 
 
m_car 
 
boolean  m_car  
 
 Flag indicating whether class association rules are mined.
 
 
 
 

 
   
 
 
  
 Class  weka.associations.PriorEstimation  extends java.lang.Object implements Serializable   
 
 

 
 serialVersionUID:  5570863216522496271L

 
   
 
 
  
 Serialized Fields   
 
 

 
m_numRandRules 
 
int  m_numRandRules  
 
 The number of rnadom rules.
 
 
 
 
 
 
m_numIntervals 
 
int  m_numIntervals  
 
 The number of intervals.
 
 
 
 
 
 
m_randNum 
 
java.util.Random  m_randNum  
 
 The random number generator.
 
 
 
 
 
 
m_instances 
 
 Instances   m_instances  
 
 The instances for which association rules are mined.
 
 
 
 
 
 
m_CARs 
 
boolean  m_CARs  
 
 Flag indicating whether standard association rules or class association rules are mined.
 
 
 
 
 
 
m_distribution 
 
java.util.Hashtable<K,V>  m_distribution  
 
 Hashtable to store the confidence values of randomly generated rules.
 
 
 
 
 
 
m_priors 
 
java.util.Hashtable<K,V>  m_priors  
 
 Hashtable containing the estimated prior probabilities.
 
 
 
 
 
 
m_sum 
 
double  m_sum  
 
 Sums up the confidences of all rules with a certain length.
 
 
 
 
 
 
m_midPoints 
 
double[]  m_midPoints  
 
 The mid points of the discrete intervals in which the interval [0,1] is divided.
 
 
 
 

 
   
 
 
  
 Class  weka.associations.RuleGeneration  extends java.lang.Object implements Serializable   
 
 

 
 serialVersionUID:  -8927041669872491432L

 
   
 
 
  
 Serialized Fields   
 
 

 
m_items 
 
int[]  m_items  
 
 The items stored as an array of of integer.
 
 
 
 
 
 
m_counter 
 
int  m_counter  
 
 Counter for how many transactions contain this item set.
 
 
 
 
 
 
m_totalTransactions 
 
int  m_totalTransactions  
 
 The total number of transactions
 
 
 
 
 
 
m_change 
 
boolean  m_change  
 
 Flag indicating whether the list fo the best rules has changed.
 
 
 
 
 
 
m_expectation 
 
double  m_expectation  
 
 The minimum expected predictive accuracy that is needed to be a candidate for the list of the best rules.
 
 
 
 
 
 
m_minRuleCount 
 
int  m_minRuleCount  
 
 The minimum support a rule needs to be a candidate for the list of the best rules.
 
 
 
 
 
 
m_midPoints 
 
double[]  m_midPoints  
 
 Sorted array of the mied points of the intervals used for prior estimation.
 
 
 
 
 
 
m_priors 
 
java.util.Hashtable<K,V>  m_priors  
 
 Hashtable conatining the estimated prior probabilities.
 
 
 
 
 
 
m_best 
 
java.util.TreeSet<E>  m_best  
 
 The list of the actual  n  best rules.
 
 
 
 
 
 
m_count 
 
int  m_count  
 
 Integer indicating the generation time of a rule.
 
 
 
 
 
 
m_instances 
 
 Instances   m_instances  
 
 The instances.
 
 
 
 

 
   
 
 
  
 Class  weka.associations.RuleItem  extends java.lang.Object implements Serializable   
 
 

 
 serialVersionUID:  -3761299128347476534L

 
   
 
 
  
 Serialized Fields   
 
 

 
m_premise 
 
 ItemSet   m_premise  
 
 The premise of a rule.
 
 
 
 
 
 
m_consequence 
 
 ItemSet   m_consequence  
 
 The consequence of a rule.
 
 
 
 
 
 
m_accuracy 
 
double  m_accuracy  
 
 The expected predictive accuracy of a rule.
 
 
 
 
 
 
m_genTime 
 
int  m_genTime  
 
 The generation time of a rule.
 
 
 
 

 
   
 
 
  
 Class  weka.associations.SingleAssociatorEnhancer  extends  AbstractAssociator  implements Serializable   
 
 

 
 serialVersionUID:  -3665885256363525164L

 
   
 
 
  
 Serialized Fields   
 
 

 
m_Associator 
 
 Associator   m_Associator  
 
 The base associator to use
 
 
 
 

 
   
 
 
  
 Class  weka.associations.Tertius  extends  AbstractAssociator  implements Serializable   
 
 

 
 serialVersionUID:  5556726848380738179L

 
   
 
 
  
 Serialized Fields   
 
 

 
m_results 
 
 SimpleLinkedList   m_results  
 
 The results.
 
 
 
 
 
 
m_hypotheses 
 
int  m_hypotheses  
 
 Number of hypotheses considered.
 
 
 
 
 
 
m_explored 
 
int  m_explored  
 
 Number of hypotheses explored.
 
 
 
 
 
 
m_time 
 
java.util.Date  m_time  
 
 Time needed for the search.
 
 
 
 
 
 
m_valuesText 
 
java.awt.TextField  m_valuesText  
 
 Field to output the current values.
 
 
 
 
 
 
m_instances 
 
 Instances   m_instances  
 
 Instances used for the search.
 
 
 
 
 
 
m_predicates 
 
java.util.ArrayList<E>  m_predicates  
 
 Predicates used in the rules.
 
 
 
 
 
 
m_status 
 
int  m_status  
 
 Status of the search.
 
 
 
 
 
 
m_best 
 
int  m_best  
 
 Number of best confirmation values to search.
 
 
 
 
 
 
m_frequencyThreshold 
 
double  m_frequencyThreshold  
 
 Frequency threshold for the body and the negation of the head.
 
 
 
 
 
 
m_confirmationThreshold 
 
double  m_confirmationThreshold  
 
 Confirmation threshold for the rules.
 
 
 
 
 
 
m_noiseThreshold 
 
double  m_noiseThreshold  
 
 Maximal number of counter-instances.
 
 
 
 
 
 
m_repeat 
 
boolean  m_repeat  
 
 Repeat attributes ?
 
 
 
 
 
 
m_numLiterals 
 
int  m_numLiterals  
 
 Number of literals in a rule.
 
 
 
 
 
 
m_negation 
 
int  m_negation  
 
 Type of negation used in the rules.
 
 
 
 
 
 
m_classification 
 
boolean  m_classification  
 
 Classification bias.
 
 
 
 
 
 
m_classIndex 
 
int  m_classIndex  
 
 Index of class attribute.
 
 
 
 
 
 
m_horn 
 
boolean  m_horn  
 
 Horn clauses bias.
 
 
 
 
 
 
m_equivalent 
 
boolean  m_equivalent  
 
 Perform test on equivalent rules ?
 
 
 
 
 
 
m_sameClause 
 
boolean  m_sameClause  
 
 Perform test on same clauses ?
 
 
 
 
 
 
m_subsumption 
 
boolean  m_subsumption  
 
 Perform subsumption test ?
 
 
 
 
 
 
m_missing 
 
int  m_missing  
 
 Way of handling missing values in the search.
 
 
 
 
 
 
m_roc 
 
boolean  m_roc  
 
 Perform ROC analysis ?
 
 
 
 
 
 
m_partsString 
 
java.lang.String  m_partsString  
 
 Name of the file containing the parts for individual-based learning.
 
 
 
 
 
 
m_parts 
 
 Instances   m_parts  
 
 Part instances for individual-based learning.
 
 
 
 
 
 
m_printValues 
 
int  m_printValues  
 
 Type of values output.
 
 
 
 
 

 
 
  
 Package   weka.associations.gsp   
 
 

 
   
 
 
  
 Class  weka.associations.gsp.Element  extends java.lang.Object implements Serializable   
 
 

 
 serialVersionUID:  -7900701276019516371L

 
   
 
 
  
 Serialized Fields   
 
 

 
m_Events 
 
int[]  m_Events  
 
 events/items stored as an array of ints
 
 
 
 

 
   
 
 
  
 Class  weka.associations.gsp.Sequence  extends java.lang.Object implements Serializable   
 
 

 
 serialVersionUID:  -5001018056339156390L

 
   
 
 
  
 Serialized Fields   
 
 

 
m_SupportCount 
 
int  m_SupportCount  
 
 the support count of the Sequence
 
 
 
 
 
 
m_Elements 
 
 FastVector   m_Elements  
 
 ordered list of the comprised elements/itemsets
 
 
 
 
 

 
 
  
 Package   weka.associations.tertius   
 
 

 
   
 
 
  
 Class  weka.associations.tertius.AttributeValueLiteral  extends  Literal  implements Serializable   
 
 

 
 serialVersionUID:  4077436297281456239L

 
   
 
 
  
 Serialized Fields   
 
 

 
m_value 
 
java.lang.String  m_value  
 
 
 
 
 
 
m_index 
 
int  m_index  
 
 
 
 

 
   
 
 
  
 Class  weka.associations.tertius.Body  extends  LiteralSet  implements Serializable   
 
 

 
 serialVersionUID:  4870689270432218016L

 

 
   
 
 
  
 Class  weka.associations.tertius.Head  extends  LiteralSet  implements Serializable   
 
 

 
 serialVersionUID:  5068076274253706199L

 

 
   
 
 
  
 Class  weka.associations.tertius.IndividualInstance  extends  Instance  implements Serializable   
 
 

 
 serialVersionUID:  -7903938733476585114L

 
   
 
 
  
 Serialized Fields   
 
 

 
m_parts 
 
 Instances   m_parts  
 
 
 
 

 
   
 
 
  
 Class  weka.associations.tertius.IndividualInstances  extends  Instances  implements Serializable   
 
 

 
 serialVersionUID:  -7355054814895636733L

 

 
   
 
 
  
 Class  weka.associations.tertius.IndividualLiteral  extends  AttributeValueLiteral  implements Serializable   
 
 

 
 serialVersionUID:  4712404824517887435L

 
   
 
 
  
 Serialized Fields   
 
 

 
m_type 
 
int  m_type  
 
 
 
 

 
   
 
 
  
 Class  weka.associations.tertius.Literal  extends java.lang.Object implements Serializable   
 
 

 
 serialVersionUID:  2675363669503575771L

 
   
 
 
  
 Serialized Fields   
 
 

 
m_predicate 
 
 Predicate   m_predicate  
 
 
 
 
 
 
m_sign 
 
int  m_sign  
 
 
 
 
 
 
m_negation 
 
 Literal   m_negation  
 
 
 
 
 
 
m_missing 
 
int  m_missing  
 
 
 
 

 
   
 
 
  
 Class  weka.associations.tertius.LiteralSet  extends java.lang.Object implements Serializable   
 
 

 
 serialVersionUID:  6094536488654503152L

 
   
 
 
  
 Serialized Fields   
 
 

 
m_literals 
 
java.util.ArrayList<E>  m_literals  
 
 Literals contained in this set.
 
 
 
 
 
 
m_lastLiteral 
 
 Literal   m_lastLiteral  
 
 Last literal added to this set.
 
 
 
 
 
 
m_numInstances 
 
int  m_numInstances  
 
 Number of instances in the data the set deals with.
 
 
 
 
 
 
m_counterInstances 
 
java.util.ArrayList<E>  m_counterInstances  
 
 Set of counter-instances of this part of the rule.
 
 
 
 
 
 
m_counter 
 
int  m_counter  
 
 Counter for the number of counter-instances.
 
 
 
 
 
 
m_type 
 
int  m_type  
 
 Type of properties expressed in this set 
 (individual or parts properties).
 
 
 
 

 
   
 
 
  
 Class  weka.associations.tertius.Predicate  extends java.lang.Object implements Serializable   
 
 

 
 serialVersionUID:  -8374702481965026640L

 
   
 
 
  
 Serialized Fields   
 
 

 
m_literals 
 
java.util.ArrayList<E>  m_literals  
 
 
 
 
 
 
m_name 
 
java.lang.String  m_name  
 
 
 
 
 
 
m_index 
 
int  m_index  
 
 
 
 
 
 
m_isClass 
 
boolean  m_isClass  
 
 
 
 

 
   
 
 
  
 Class  weka.associations.tertius.Rule  extends java.lang.Object implements Serializable   
 
 

 
 serialVersionUID:  -7763378359090435505L

 
   
 
 
  
 Serialized Fields   
 
 

 
m_body 
 
 Body   m_body  
 
 The body of the rule.
 
 
 
 
 
 
m_head 
 
 Head   m_head  
 
 The head of the rule.
 
 
 
 
 
 
m_repeatPredicate 
 
boolean  m_repeatPredicate  
 
 Can repeat predicates in the rule ?
 
 
 
 
 
 
m_maxLiterals 
 
int  m_maxLiterals  
 
 Maximal number of literals in the rule.
 
 
 
 
 
 
m_negBody 
 
boolean  m_negBody  
 
 Can there be negations in the body ?
 
 
 
 
 
 
m_negHead 
 
boolean  m_negHead  
 
 Can there be negations in the head ?
 
 
 
 
 
 
m_classRule 
 
boolean  m_classRule  
 
 Is this rule a classification rule ?
 
 
 
 
 
 
m_singleHead 
 
boolean  m_singleHead  
 
 Can there be only one literal in the head ?
 
 
 
 
 
 
m_numInstances 
 
int  m_numInstances  
 
 Number of instances in the data this rule deals with.
 
 
 
 
 
 
m_counterInstances 
 
java.util.ArrayList<E>  m_counterInstances  
 
 Set of counter-instances of this rule.
 
 
 
 
 
 
m_counter 
 
int  m_counter  
 
 Counter for the counter-instances of this rule.
 
 
 
 
 
 
m_confirmation 
 
double  m_confirmation  
 
 Confirmation of this rule.
 
 
 
 
 
 
m_optimistic 
 
double  m_optimistic  
 
 Optimistic estimate of this rule.
 
 
 
 

 
   
 
 
  
 Class  weka.associations.tertius.SimpleLinkedList  extends java.lang.Object implements Serializable   
 
 

 
 serialVersionUID:  -1491148276509976299L

 
   
 
 
  
 Serialization Methods   
 
 

 
    
readObject 
 
private void  readObject (java.io.ObjectInputStream s)
                 throws java.io.IOException,
                        java.lang.ClassNotFoundException 
 
 Reconstitute this  LinkedList  instance from a stream (that is
 deserialize it).
 
  

  Throws: 
  java.io.IOException 
  java.lang.ClassNotFoundException  
 
 
 
    
writeObject 
 
private void  writeObject (java.io.ObjectOutputStream s)
                  throws java.io.IOException 
 
 Save the state of this  LinkedList  instance to a stream (that
 is, serialize it).
 
  
  Serial Data:  
   The size of the list (the number of elements it
                   contains) is emitted (int), followed by all of its
 elements (each an Object) in the proper order. 

  Throws: 
  java.io.IOException  
 
 
   
 
 
  
 Serialized Fields   
 
 

 
first 
 
weka.associations.tertius.SimpleLinkedList.Entry  first  
 
 
 
 
 
 
last 
 
weka.associations.tertius.SimpleLinkedList.Entry  last  
 
 
 
 

 
   
 
 
  
 Class  weka.associations.tertius.SimpleLinkedList.LinkedListInverseIterator  extends java.lang.Object implements Serializable   
 
 

 
 serialVersionUID:  6290379064027832108L

 
   
 
 
  
 Serialized Fields   
 
 

 
current 
 
weka.associations.tertius.SimpleLinkedList.Entry  current  
 
 
 
 
 
 
lastReturned 
 
weka.associations.tertius.SimpleLinkedList.Entry  lastReturned  
 
 
 
 

 
   
 
 
  
 Class  weka.associations.tertius.SimpleLinkedList.LinkedListIterator  extends java.lang.Object implements Serializable   
 
 

 
 serialVersionUID:  -2448555236100426759L

 
   
 
 
  
 Serialized Fields   
 
 

 
current 
 
weka.associations.tertius.SimpleLinkedList.Entry  current  
 
 
 
 
 
 
lastReturned 
 
weka.associations.tertius.SimpleLinkedList.Entry  lastReturned  
 
 
 
 
 

 
 
  
 Package   weka.attributeSelection   
 
 

 
   
 
 
  
 Class  weka.attributeSelection.ASEvaluation  extends java.lang.Object implements Serializable   
 
 

 
 serialVersionUID:  2091705669885950849L

 

 
   
 
 
  
 Class  weka.attributeSelection.ASSearch  extends java.lang.Object implements Serializable   
 
 

 
 serialVersionUID:  7591673350342236548L

 

 
   
 
 
  
 Class  weka.attributeSelection.AttributeSelection  extends java.lang.Object implements Serializable   
 
 

 
 serialVersionUID:  4170171824147584330L

 
   
 
 
  
 Serialized Fields   
 
 

 
m_trainInstances 
 
 Instances   m_trainInstances  
 
 the instances to select attributes from
 
 
 
 
 
 
m_ASEvaluator 
 
 ASEvaluation   m_ASEvaluator  
 
 the attribute/subset evaluator
 
 
 
 
 
 
m_searchMethod 
 
 ASSearch   m_searchMethod  
 
 the search method
 
 
 
 
 
 
m_numFolds 
 
int  m_numFolds  
 
 the number of folds to use for cross validation
 
 
 
 
 
 
m_selectionResults 
 
java.lang.StringBuffer  m_selectionResults  
 
 holds a string describing the results of the attribute selection
 
 
 
 
 
 
m_doRank 
 
boolean  m_doRank  
 
 rank features (if allowed by the search method)
 
 
 
 
 
 
m_doXval 
 
boolean  m_doXval  
 
 do cross validation
 
 
 
 
 
 
m_seed 
 
int  m_seed  
 
 seed used to randomly shuffle instances for cross validation
 
 
 
 
 
 
m_numToSelect 
 
int  m_numToSelect  
 
 number of attributes requested from ranked results
 
 
 
 
 
 
m_selectedAttributeSet 
 
int[]  m_selectedAttributeSet  
 
 the selected attributes
 
 
 
 
 
 
m_attributeRanking 
 
double[][]  m_attributeRanking  
 
 the attribute indexes and associated merits if a ranking is produced
 
 
 
 
 
 
m_transformer 
 
 AttributeTransformer   m_transformer  
 
 if a feature selection run involves an attribute transformer
 
 
 
 
 
 
m_attributeFilter 
 
 Remove   m_attributeFilter  
 
 the attribute filter for processing instances with respect to
      the most recent feature selection run
 
 
 
 
 
 
m_rankResults 
 
double[][]  m_rankResults  
 
 hold statistics for repeated feature selection, such as
      under cross validation
 
 
 
 
 
 
m_subsetResults 
 
double[]  m_subsetResults  
 
 
 
 
 
 
m_trials 
 
int  m_trials  
 
 
 
 

 
   
 
 
  
 Class  weka.attributeSelection.AttributeSetEvaluator  extends  ASEvaluation  implements Serializable   
 
 

 
 serialVersionUID:  -5744881009422257389L

 

 
   
 
 
  
 Class  weka.attributeSelection.BestFirst  extends  ASSearch  implements Serializable   
 
 

 
 serialVersionUID:  7841338689536821867L

 
   
 
 
  
 Serialized Fields   
 
 

 
m_maxStale 
 
int  m_maxStale  
 
 maximum number of stale nodes before terminating search
 
 
 
 
 
 
m_searchDirection 
 
int  m_searchDirection  
 
 0 == backward search, 1 == forward search, 2 == bidirectional
 
 
 
 
 
 
m_starting 
 
int[]  m_starting  
 
 holds an array of starting attributes
 
 
 
 
 
 
m_startRange 
 
 Range   m_startRange  
 
 holds the start set for the search as a Range
 
 
 
 
 
 
m_hasClass 
 
boolean  m_hasClass  
 
 does the data have a class
 
 
 
 
 
 
m_classIndex 
 
int  m_classIndex  
 
 holds the class index
 
 
 
 
 
 
m_numAttribs 
 
int  m_numAttribs  
 
 number of attributes in the data
 
 
 
 
 
 
m_totalEvals 
 
int  m_totalEvals  
 
 total number of subsets evaluated during a search
 
 
 
 
 
 
m_debug 
 
boolean  m_debug  
 
 for debugging
 
 
 
 
 
 
m_bestMerit 
 
double  m_bestMerit  
 
 holds the merit of the best subset found
 
 
 
 
 
 
m_cacheSize 
 
int  m_cacheSize  
 
 holds the maximum size of the lookup cache for evaluated subsets
 
 
 
 

 
   
 
 
  
 Class  weka.attributeSelection.BestFirst.Link2  extends java.lang.Object implements Serializable   
 
 

 
 serialVersionUID:  -8236598311516351420L

 
   
 
 
  
 Serialized Fields   
 
 

 
m_data 
 
java.lang.Object[]  m_data  
 
 
 
 
 
 
m_merit 
 
double  m_merit  
 
 
 
 

 
   
 
 
  
 Class  weka.attributeSelection.BestFirst.LinkedList2  extends  FastVector  implements Serializable   
 
 

 
 serialVersionUID:  3250538292330398929L

 
   
 
 
  
 Serialized Fields   
 
 

 
m_MaxSize 
 
int  m_MaxSize  
 
 Max number of elements in the list
 
 
 
 

 
   
 
 
  
 Class  weka.attributeSelection.CfsSubsetEval  extends  ASEvaluation  implements Serializable   
 
 

 
 serialVersionUID:  747878400813276317L

 
   
 
 
  
 Serialized Fields   
 
 

 
m_trainInstances 
 
 Instances   m_trainInstances  
 
 The training instances
 
 
 
 
 
 
m_disTransform 
 
 Discretize   m_disTransform  
 
 Discretise attributes when class in nominal
 
 
 
 
 
 
m_classIndex 
 
int  m_classIndex  
 
 The class index
 
 
 
 
 
 
m_isNumeric 
 
boolean  m_isNumeric  
 
 Is the class numeric
 
 
 
 
 
 
m_numAttribs 
 
int  m_numAttribs  
 
 Number of attributes in the training data
 
 
 
 
 
 
m_numInstances 
 
int  m_numInstances  
 
 Number of instances in the training data
 
 
 
 
 
 
m_missingSeparate 
 
boolean  m_missingSeparate  
 
 Treat missing values as separate values
 
 
 
 
 
 
m_locallyPredictive 
 
boolean  m_locallyPredictive  
 
 Include locally predicitive attributes
 
 
 
 
 
 
m_corr_matrix 
 
float[][]  m_corr_matrix  
 
 Holds the matrix of attribute correlations
 
 
 
 
 
 
m_std_devs 
 
double[]  m_std_devs  
 
 Standard deviations of attributes (when using pearsons correlation)
 
 
 
 
 
 
m_c_Threshold 
 
double  m_c_Threshold  
 
 Threshold for admitting locally predictive features
 
 
 
 

 
   
 
 
  
 Class  weka.attributeSelection.ChiSquaredAttributeEval  extends  ASEvaluation  implements Serializable   
 
 

 
 serialVersionUID:  -8316857822521717692L

 
   
 
 
  
 Serialized Fields   
 
 

 
m_missing_merge 
 
boolean  m_missing_merge  
 
 Treat missing values as a seperate value
 
 
 
 
 
 
m_Binarize 
 
boolean  m_Binarize  
 
 Just binarize numeric attributes
 
 
 
 
 
 
m_ChiSquareds 
 
double[]  m_ChiSquareds  
 
 The chi-squared value for each attribute
 
 
 
 

 
   
 
 
  
 Class  weka.attributeSelection.ClassifierSubsetEval  extends  HoldOutSubsetEvaluator  implements Serializable   
 
 

 
 serialVersionUID:  7532217899385278710L

 
   
 
 
  
 Serialized Fields   
 
 

 
m_trainingInstances 
 
 Instances   m_trainingInstances  
 
 training instances
 
 
 
 
 
 
m_classIndex 
 
int  m_classIndex  
 
 class index
 
 
 
 
 
 
m_numAttribs 
 
int  m_numAttribs  
 
 number of attributes in the training data
 
 
 
 
 
 
m_numInstances 
 
int  m_numInstances  
 
 number of training instances
 
 
 
 
 
 
m_Classifier 
 
 Classifier   m_Classifier  
 
 holds the classifier to use for error estimates
 
 
 
 
 
 
m_Evaluation 
 
 Evaluation   m_Evaluation  
 
 holds the evaluation object to use for evaluating the classifier
 
 
 
 
 
 
m_holdOutFile 
 
java.io.File  m_holdOutFile  
 
 the file that containts hold out/test instances
 
 
 
 
 
 
m_holdOutInstances 
 
 Instances   m_holdOutInstances  
 
 the instances to test on
 
 
 
 
 
 
m_useTraining 
 
boolean  m_useTraining  
 
 evaluate on training data rather than seperate hold out/test set
 
 
 
 

 
   
 
 
  
 Class  weka.attributeSelection.ConsistencySubsetEval  extends  ASEvaluation  implements Serializable   
 
 

 
 serialVersionUID:  -2880323763295270402L

 
   
 
 
  
 Serialized Fields   
 
 

 
m_trainInstances 
 
 Instances   m_trainInstances  
 
 training instances
 
 
 
 
 
 
m_classIndex 
 
int  m_classIndex  
 
 class index
 
 
 
 
 
 
m_numAttribs 
 
int  m_numAttribs  
 
 number of attributes in the training data
 
 
 
 
 
 
m_numInstances 
 
int  m_numInstances  
 
 number of instances in the training data
 
 
 
 
 
 
m_disTransform 
 
 Discretize   m_disTransform  
 
 Discretise numeric attributes
 
 
 
 
 
 
m_table 
 
java.util.Hashtable<K,V>  m_table  
 
 Hash table for evaluating feature subsets
 
 
 
 

 
   
 
 
  
 Class  weka.attributeSelection.ConsistencySubsetEval.hashKey  extends java.lang.Object implements Serializable   
 
 

 
 serialVersionUID:  6144138512017017408L

 
   
 
 
  
 Serialized Fields   
 
 

 
attributes 
 
double[]  attributes  
 
 Array of attribute values for an instance
 
 
 
 
 
 
missing 
 
boolean[]  missing  
 
 True for an index if the corresponding attribute value is missing.
 
 
 
 
 
 
key 
 
int  key  
 
 The key
 
 
 
 

 
   
 
 
  
 Class  weka.attributeSelection.CostSensitiveASEvaluation  extends  ASEvaluation  implements Serializable   
 
 

 
 serialVersionUID:  -7045833833363396977L

 
   
 
 
  
 Serialized Fields   
 
 

 
m_MatrixSource 
 
int  m_MatrixSource  
 
 Indicates the current cost matrix source
 
 
 
 
 
 
m_OnDemandDirectory 
 
java.io.File  m_OnDemandDirectory  
 
 The directory used when loading cost files on demand, null indicates
 current directory
 
 
 
 
 
 
m_CostFile 
 
java.lang.String  m_CostFile  
 
 The name of the cost file, for command line options
 
 
 
 
 
 
m_CostMatrix 
 
 CostMatrix   m_CostMatrix  
 
 The cost matrix
 
 
 
 
 
 
m_evaluator 
 
 ASEvaluation   m_evaluator  
 
 The base evaluator to use
 
 
 
 
 
 
m_seed 
 
int  m_seed  
 
 random number seed
 
 
 
 

 
   
 
 
  
 Class  weka.attributeSelection.CostSensitiveAttributeEval  extends  CostSensitiveASEvaluation  implements Serializable   
 
 

 
 serialVersionUID:  4484876541145458447L

 

 
   
 
 
  
 Class  weka.attributeSelection.CostSensitiveSubsetEval  extends  CostSensitiveASEvaluation  implements Serializable   
 
 

 
 serialVersionUID:  2924546096103426700L

 

 
   
 
 
  
 Class  weka.attributeSelection.ExhaustiveSearch  extends  ASSearch  implements Serializable   
 
 

 
 serialVersionUID:  5741842861142379712L

 
   
 
 
  
 Serialized Fields   
 
 

 
m_bestGroup 
 
java.util.BitSet  m_bestGroup  
 
 the best feature set found during the search
 
 
 
 
 
 
m_bestMerit 
 
double  m_bestMerit  
 
 the merit of the best subset found
 
 
 
 
 
 
m_hasClass 
 
boolean  m_hasClass  
 
 does the data have a class
 
 
 
 
 
 
m_classIndex 
 
int  m_classIndex  
 
 holds the class index
 
 
 
 
 
 
m_numAttribs 
 
int  m_numAttribs  
 
 number of attributes in the data
 
 
 
 
 
 
m_verbose 
 
boolean  m_verbose  
 
 if true, then ouput new best subsets as the search progresses
 
 
 
 
 
 
m_evaluations 
 
int  m_evaluations  
 
 the number of subsets evaluated during the search
 
 
 
 

 
   
 
 
  
 Class  weka.attributeSelection.FilteredAttributeEval  extends  ASEvaluation  implements Serializable   
 
 

 
 serialVersionUID:  2111121880778327334L

 
   
 
 
  
 Serialized Fields   
 
 

 
m_evaluator 
 
 AttributeEvaluator   m_evaluator  
 
 Base evaluator
 
 
 
 
 
 
m_filter 
 
 Filter   m_filter  
 
 Filter
 
 
 
 
 
 
m_filteredInstances 
 
 Instances   m_filteredInstances  
 
 Filtered instances structure
 
 
 
 

 
   
 
 
  
 Class  weka.attributeSelection.FilteredSubsetEval  extends  ASEvaluation  implements Serializable   
 
 

 
 serialVersionUID:  2111121880778327334L

 
   
 
 
  
 Serialized Fields   
 
 

 
m_evaluator 
 
 SubsetEvaluator   m_evaluator  
 
 Base evaluator
 
 
 
 
 
 
m_filter 
 
 Filter   m_filter  
 
 Filter
 
 
 
 
 
 
m_filteredInstances 
 
 Instances   m_filteredInstances  
 
 Filtered instances structure
 
 
 
 

 
   
 
 
  
 Class  weka.attributeSelection.GainRatioAttributeEval  extends  ASEvaluation  implements Serializable   
 
 

 
 serialVersionUID:  -8504656625598579926L

 
   
 
 
  
 Serialized Fields   
 
 

 
m_trainInstances 
 
 Instances   m_trainInstances  
 
 The training instances
 
 
 
 
 
 
m_classIndex 
 
int  m_classIndex  
 
 The class index
 
 
 
 
 
 
m_numAttribs 
 
int  m_numAttribs  
 
 The number of attributes
 
 
 
 
 
 
m_numInstances 
 
int  m_numInstances  
 
 The number of instances
 
 
 
 
 
 
m_numClasses 
 
int  m_numClasses  
 
 The number of classes
 
 
 
 
 
 
m_missing_merge 
 
boolean  m_missing_merge  
 
 Merge missing values
 
 
 
 

 
   
 
 
  
 Class  weka.attributeSelection.GeneticSearch  extends  ASSearch  implements Serializable   
 
 

 
 serialVersionUID:  -1618264232838472679L

 
   
 
 
  
 Serialized Fields   
 
 

 
m_starting 
 
int[]  m_starting  
 
 holds a starting set as an array of attributes. Becomes one member of the
 initial random population
 
 
 
 
 
 
m_startRange 
 
 Range   m_startRange  
 
 holds the start set for the search as a Range
 
 
 
 
 
 
m_hasClass 
 
boolean  m_hasClass  
 
 does the data have a class
 
 
 
 
 
 
m_classIndex 
 
int  m_classIndex  
 
 holds the class index
 
 
 
 
 
 
m_numAttribs 
 
int  m_numAttribs  
 
 number of attributes in the data
 
 
 
 
 
 
m_population 
 
weka.attributeSelection.GeneticSearch.GABitSet[]  m_population  
 
 the current population
 
 
 
 
 
 
m_popSize 
 
int  m_popSize  
 
 the number of individual solutions
 
 
 
 
 
 
m_best 
 
weka.attributeSelection.GeneticSearch.GABitSet  m_best  
 
 the best population member found during the search
 
 
 
 
 
 
m_bestFeatureCount 
 
int  m_bestFeatureCount  
 
 the number of features in the best population member
 
 
 
 
 
 
m_lookupTableSize 
 
int  m_lookupTableSize  
 
 the number of entries to cache for lookup
 
 
 
 
 
 
m_lookupTable 
 
java.util.Hashtable<K,V>  m_lookupTable  
 
 the lookup table
 
 
 
 
 
 
m_random 
 
java.util.Random  m_random  
 
 random number generation
 
 
 
 
 
 
m_seed 
 
int  m_seed  
 
 seed for random number generation
 
 
 
 
 
 
m_pCrossover 
 
double  m_pCrossover  
 
 the probability of crossover occuring
 
 
 
 
 
 
m_pMutation 
 
double  m_pMutation  
 
 the probability of mutation occuring
 
 
 
 
 
 
m_sumFitness 
 
double  m_sumFitness  
 
 sum of the current population fitness
 
 
 
 
 
 
m_maxFitness 
 
double  m_maxFitness  
 
 
 
 
 
 
m_minFitness 
 
double  m_minFitness  
 
 
 
 
 
 
m_avgFitness 
 
double  m_avgFitness  
 
 
 
 
 
 
m_maxGenerations 
 
int  m_maxGenerations  
 
 the maximum number of generations to evaluate
 
 
 
 
 
 
m_reportFrequency 
 
int  m_reportFrequency  
 
 how often reports are generated
 
 
 
 
 
 
m_generationReports 
 
java.lang.StringBuffer  m_generationReports  
 
 holds the generation reports
 
 
 
 

 
   
 
 
  
 Class weka.attributeSelection.GeneticSearch.GABitSet extends java.lang.Object implements Serializable   
 
 

 
 serialVersionUID:  -2930607837482622224L

 
   
 
 
  
 Serialized Fields   
 
 

 
m_chromosome 
 
java.util.BitSet  m_chromosome  
 
 the bitset
 
 
 
 
 
 
m_objective 
 
double  m_objective  
 
 holds raw merit
 
 
 
 
 
 
m_fitness 
 
double  m_fitness  
 
 the fitness
 
 
 
 

 
   
 
 
  
 Class  weka.attributeSelection.GreedyStepwise  extends  ASSearch  implements Serializable   
 
 

 
 serialVersionUID:  -6312951970168325471L

 
   
 
 
  
 Serialized Fields   
 
 

 
m_hasClass 
 
boolean  m_hasClass  
 
 does the data have a class
 
 
 
 
 
 
m_classIndex 
 
int  m_classIndex  
 
 holds the class index
 
 
 
 
 
 
m_numAttribs 
 
int  m_numAttribs  
 
 number of attributes in the data
 
 
 
 
 
 
m_rankingRequested 
 
boolean  m_rankingRequested  
 
 true if the user has requested a ranked list of attributes
 
 
 
 
 
 
m_doRank 
 
boolean  m_doRank  
 
 go from one side of the search space to the other in order to generate
 a ranking
 
 
 
 
 
 
m_doneRanking 
 
boolean  m_doneRanking  
 
 used to indicate whether or not ranking has been performed
 
 
 
 
 
 
m_threshold 
 
double  m_threshold  
 
 A threshold by which to discard attributes---used by the
 AttributeSelection module
 
 
 
 
 
 
m_numToSelect 
 
int  m_numToSelect  
 
 The number of attributes to select. -1 indicates that all attributes
      are to be retained. Has precedence over m_threshold
 
 
 
 
 
 
m_calculatedNumToSelect 
 
int  m_calculatedNumToSelect  
 
 
 
 
 
 
m_bestMerit 
 
double  m_bestMerit  
 
 the merit of the best subset found
 
 
 
 
 
 
m_rankedAtts 
 
double[][]  m_rankedAtts  
 
 a ranked list of attribute indexes
 
 
 
 
 
 
m_rankedSoFar 
 
int  m_rankedSoFar  
 
 
 
 
 
 
m_best_group 
 
java.util.BitSet  m_best_group  
 
 the best subset found
 
 
 
 
 
 
m_ASEval 
 
 ASEvaluation   m_ASEval  
 
 
 
 
 
 
m_Instances 
 
 Instances   m_Instances  
 
 
 
 
 
 
m_startRange 
 
 Range   m_startRange  
 
 holds the start set for the search as a Range
 
 
 
 
 
 
m_starting 
 
int[]  m_starting  
 
 holds an array of starting attributes
 
 
 
 
 
 
m_backward 
 
boolean  m_backward  
 
 Use a backwards search instead of a forwards one
 
 
 
 
 
 
m_conservativeSelection 
 
boolean  m_conservativeSelection  
 
 If set then attributes will continue to be added during a forward
      search as long as the merit does not degrade
 
 
 
 

 
   
 
 
  
 Class  weka.attributeSelection.HoldOutSubsetEvaluator  extends  ASEvaluation  implements Serializable   
 
 

 
 serialVersionUID:  8280529785412054174L

 

 
   
 
 
  
 Class  weka.attributeSelection.InfoGainAttributeEval  extends  ASEvaluation  implements Serializable   
 
 

 
 serialVersionUID:  -1949849512589218930L

 
   
 
 
  
 Serialized Fields   
 
 

 
m_missing_merge 
 
boolean  m_missing_merge  
 
 Treat missing values as a seperate value
 
 
 
 
 
 
m_Binarize 
 
boolean  m_Binarize  
 
 Just binarize numeric attributes
 
 
 
 
 
 
m_InfoGains 
 
double[]  m_InfoGains  
 
 The info gain for each attribute
 
 
 
 

 
   
 
 
  
 Class  weka.attributeSelection.LatentSemanticAnalysis  extends  UnsupervisedAttributeEvaluator  implements Serializable   
 
 

 
 serialVersionUID:  -8712112988018106198L

 
   
 
 
  
 Serialized Fields   
 
 

 
m_trainInstances 
 
 Instances   m_trainInstances  
 
 The data to transform analyse/transform
 
 
 
 
 
 
m_trainHeader 
 
 Instances   m_trainHeader  
 
 Keep a copy for the class attribute (if set) and for checking
 header compatibility
 
 
 
 
 
 
m_transformedFormat 
 
 Instances   m_transformedFormat  
 
 The header for the transformed data format
 
 
 
 
 
 
m_hasClass 
 
boolean  m_hasClass  
 
 Data has a class set
 
 
 
 
 
 
m_classIndex 
 
int  m_classIndex  
 
 Class index
 
 
 
 
 
 
m_numAttributes 
 
int  m_numAttributes  
 
 Number of attributes
 
 
 
 
 
 
m_numInstances 
 
int  m_numInstances  
 
 Number of instances
 
 
 
 
 
 
m_transpose 
 
boolean  m_transpose  
 
 Is transpose necessary because numAttributes  
 
 
 
 
 
m_u 
 
 Matrix   m_u  
 
 Will hold the left singular vectors
 
 
 
 
 
 
m_s 
 
 Matrix   m_s  
 
 Will hold the singular values
 
 
 
 
 
 
m_v 
 
 Matrix   m_v  
 
 Will hold the right singular values
 
 
 
 
 
 
m_transformationMatrix 
 
 Matrix   m_transformationMatrix  
 
 Will hold the matrix used to transform instances to the new feature space
 
 
 
 
 
 
m_replaceMissingFilter 
 
 ReplaceMissingValues   m_replaceMissingFilter  
 
 Filters for original data
 
 
 
 
 
 
m_normalizeFilter 
 
 Normalize   m_normalizeFilter  
 
 
 
 
 
 
m_nominalToBinaryFilter 
 
 NominalToBinary   m_nominalToBinaryFilter  
 
 
 
 
 
 
m_attributeFilter 
 
 Remove   m_attributeFilter  
 
 
 
 
 
 
m_outputNumAttributes 
 
int  m_outputNumAttributes  
 
 The number of attributes in the LSA transformed data
 
 
 
 
 
 
m_normalize 
 
boolean  m_normalize  
 
 Normalize the input data?
 
 
 
 
 
 
m_rank 
 
double  m_rank  
 
 The approximation rank to use (between 0 and 1 means coverage proportion)
 
 
 
 
 
 
m_sumSquaredSingularValues 
 
double  m_sumSquaredSingularValues  
 
 The sum of the squares of the singular values
 
 
 
 
 
 
m_actualRank 
 
int  m_actualRank  
 
 The actual rank number to use for computation
 
 
 
 
 
 
m_maxAttributesInName 
 
int  m_maxAttributesInName  
 
 Maximum number of attributes in the transformed attribute name
 
 
 
 

 
   
 
 
  
 Class  weka.attributeSelection.LFSMethods.Link2  extends java.lang.Object implements Serializable   
 
 

 
 serialVersionUID:  -7422719407475185086L

 
   
 
 
  
 Serialized Fields   
 
 

 
m_data 
 
java.lang.Object[]  m_data  
 
 
 
 
 
 
m_merit 
 
double  m_merit  
 
 
 
 

 
   
 
 
  
 Class  weka.attributeSelection.LFSMethods.LinkedList2  extends  FastVector  implements Serializable   
 
 

 
 serialVersionUID:  -7776010892419656105L

 
   
 
 
  
 Serialized Fields   
 
 

 
m_MaxSize 
 
int  m_MaxSize  
 
 
 
 

 
   
 
 
  
 Class  weka.attributeSelection.LinearForwardSelection  extends  ASSearch  implements Serializable   
 
 

 
   
 
 
  
 Serialized Fields   
 
 

 
m_maxStale 
 
int  m_maxStale  
 
 maximum number of stale nodes before terminating search
 
 
 
 
 
 
m_forwardSearchMethod 
 
int  m_forwardSearchMethod  
 
 0 == forward selection, 1 == floating forward search
 
 
 
 
 
 
m_performRanking 
 
boolean  m_performRanking  
 
 perform initial ranking to select top-ranked attributes
 
 
 
 
 
 
m_numUsedAttributes 
 
int  m_numUsedAttributes  
 
 number of top-ranked attributes that are taken into account for the
 search
 
 
 
 
 
 
m_linearSelectionType 
 
int  m_linearSelectionType  
 
 0 == fixed-set, 1 == fixed-width
 
 
 
 
 
 
m_starting 
 
int[]  m_starting  
 
 holds an array of starting attributes
 
 
 
 
 
 
m_startRange 
 
 Range   m_startRange  
 
 holds the start set for the search as a Range
 
 
 
 
 
 
m_hasClass 
 
boolean  m_hasClass  
 
 does the data have a class
 
 
 
 
 
 
m_classIndex 
 
int  m_classIndex  
 
 holds the class index
 
 
 
 
 
 
m_numAttribs 
 
int  m_numAttribs  
 
 number of attributes in the data
 
 
 
 
 
 
m_totalEvals 
 
int  m_totalEvals  
 
 total number of subsets evaluated during a search
 
 
 
 
 
 
m_verbose 
 
boolean  m_verbose  
 
 for debugging
 
 
 
 
 
 
m_bestMerit 
 
double  m_bestMerit  
 
 holds the merit of the best subset found
 
 
 
 
 
 
m_cacheSize 
 
int  m_cacheSize  
 
 holds the maximum size of the lookup cache for evaluated subsets
 
 
 
 

 
   
 
 
  
 Class  weka.attributeSelection.OneRAttributeEval  extends  ASEvaluation  implements Serializable   
 
 

 
 serialVersionUID:  4386514823886856980L

 
   
 
 
  
 Serialized Fields   
 
 

 
m_trainInstances 
 
 Instances   m_trainInstances  
 
 The training instances
 
 
 
 
 
 
m_classIndex 
 
int  m_classIndex  
 
 The class index
 
 
 
 
 
 
m_numAttribs 
 
int  m_numAttribs  
 
 The number of attributes
 
 
 
 
 
 
m_numInstances 
 
int  m_numInstances  
 
 The number of instances
 
 
 
 
 
 
m_randomSeed 
 
int  m_randomSeed  
 
 Random number seed
 
 
 
 
 
 
m_folds 
 
int  m_folds  
 
 Number of folds for cross validation
 
 
 
 
 
 
m_evalUsingTrainingData 
 
boolean  m_evalUsingTrainingData  
 
 Use training data to evaluate merit rather than x-val
 
 
 
 
 
 
m_minBucketSize 
 
int  m_minBucketSize  
 
 Passed on to OneR
 
 
 
 

 
   
 
 
  
 Class  weka.attributeSelection.PrincipalComponents  extends  UnsupervisedAttributeEvaluator  implements Serializable   
 
 

 
 serialVersionUID:  -3675307197777734007L

 
   
 
 
  
 Serialized Fields   
 
 

 
m_trainInstances 
 
 Instances   m_trainInstances  
 
 The data to transform analyse/transform
 
 
 
 
 
 
m_trainHeader 
 
 Instances   m_trainHeader  
 
 Keep a copy for the class attribute (if set)
 
 
 
 
 
 
m_transformedFormat 
 
 Instances   m_transformedFormat  
 
 The header for the transformed data format
 
 
 
 
 
 
m_originalSpaceFormat 
 
 Instances   m_originalSpaceFormat  
 
 The header for data transformed back to the original space
 
 
 
 
 
 
m_hasClass 
 
boolean  m_hasClass  
 
 Data has a class set
 
 
 
 
 
 
m_classIndex 
 
int  m_classIndex  
 
 Class index
 
 
 
 
 
 
m_numAttribs 
 
int  m_numAttribs  
 
 Number of attributes
 
 
 
 
 
 
m_numInstances 
 
int  m_numInstances  
 
 Number of instances
 
 
 
 
 
 
m_correlation 
 
double[][]  m_correlation  
 
 Correlation/covariance matrix for the original data
 
 
 
 
 
 
m_means 
 
double[]  m_means  
 
 
 
 
 
 
m_stdDevs 
 
double[]  m_stdDevs  
 
 
 
 
 
 
m_center 
 
boolean  m_center  
 
 If true, center (rather than standardize) the data and
 compute PCA from covariance (rather than correlation)
 matrix.
 
 
 
 
 
 
m_eigenvectors 
 
double[][]  m_eigenvectors  
 
 Will hold the unordered linear transformations of the (normalized)
      original data
 
 
 
 
 
 
m_eigenvalues 
 
double[]  m_eigenvalues  
 
 Eigenvalues for the corresponding eigenvectors
 
 
 
 
 
 
m_sortedEigens 
 
int[]  m_sortedEigens  
 
 Sorted eigenvalues
 
 
 
 
 
 
m_sumOfEigenValues 
 
double  m_sumOfEigenValues  
 
 sum of the eigenvalues
 
 
 
 
 
 
m_replaceMissingFilter 
 
 ReplaceMissingValues   m_replaceMissingFilter  
 
 Filters for original data
 
 
 
 
 
 
m_nominalToBinFilter 
 
 NominalToBinary   m_nominalToBinFilter  
 
 
 
 
 
 
m_attributeFilter 
 
 Remove   m_attributeFilter  
 
 
 
 
 
 
m_centerFilter 
 
 Center   m_centerFilter  
 
 
 
 
 
 
m_standardizeFilter 
 
 Standardize   m_standardizeFilter  
 
 
 
 
 
 
m_outputNumAtts 
 
int  m_outputNumAtts  
 
 The number of attributes in the pc transformed data
 
 
 
 
 
 
m_coverVariance 
 
double  m_coverVariance  
 
 the amount of variance to cover in the original data when
      retaining the best n PC's
 
 
 
 
 
 
m_transBackToOriginal 
 
boolean  m_transBackToOriginal  
 
 transform the data through the pc space and back to the original
      space ?
 
 
 
 
 
 
m_maxAttrsInName 
 
int  m_maxAttrsInName  
 
 maximum number of attributes in the transformed attribute name
 
 
 
 
 
 
m_eTranspose 
 
double[][]  m_eTranspose  
 
 holds the transposed eigenvectors for converting back to the
      original space
 
 
 
 

 
   
 
 
  
 Class  weka.attributeSelection.RaceSearch  extends  ASSearch  implements Serializable   
 
 

 
 serialVersionUID:  4015453851212985720L

 
   
 
 
  
 Serialized Fields   
 
 

 
m_Instances 
 
 Instances   m_Instances  
 
 the training instances
 
 
 
 
 
 
m_raceType 
 
int  m_raceType  
 
 the selected search type
 
 
 
 
 
 
m_xvalType 
 
int  m_xvalType  
 
 the selected xval type
 
 
 
 
 
 
m_classIndex 
 
int  m_classIndex  
 
 the class index
 
 
 
 
 
 
m_numAttribs 
 
int  m_numAttribs  
 
 the number of attributes in the data
 
 
 
 
 
 
m_totalEvals 
 
int  m_totalEvals  
 
 the total number of partially/fully evaluated subsets
 
 
 
 
 
 
m_bestMerit 
 
double  m_bestMerit  
 
 holds the merit of the best subset found
 
 
 
 
 
 
m_theEvaluator 
 
 HoldOutSubsetEvaluator   m_theEvaluator  
 
 the subset evaluator to use
 
 
 
 
 
 
m_sigLevel 
 
double  m_sigLevel  
 
 the significance level for comparisons
 
 
 
 
 
 
m_delta 
 
double  m_delta  
 
 threshold for comparisons
 
 
 
 
 
 
m_samples 
 
int  m_samples  
 
 the number of samples above which to begin testing for similarity
      between competing subsets
 
 
 
 
 
 
m_numFolds 
 
int  m_numFolds  
 
 number of cross validation folds---equal to the number of instances
      for leave-one-out cv
 
 
 
 
 
 
m_ASEval 
 
 ASEvaluation   m_ASEval  
 
 the attribute evaluator to generate the initial ranking when
      doing a rank race
 
 
 
 
 
 
m_Ranking 
 
int[]  m_Ranking  
 
 will hold the attribute ranking produced by the above attribute
      evaluator if doing a rank search
 
 
 
 
 
 
m_debug 
 
boolean  m_debug  
 
 verbose output for monitoring the search and debugging
 
 
 
 
 
 
m_rankingRequested 
 
boolean  m_rankingRequested  
 
 If true then produce a ranked list of attributes by fully traversing
      a forward hillclimb race
 
 
 
 
 
 
m_rankedAtts 
 
double[][]  m_rankedAtts  
 
 The ranked list of attributes produced if m_rankingRequested is true
 
 
 
 
 
 
m_rankedSoFar 
 
int  m_rankedSoFar  
 
 The number of attributes ranked so far (if ranking is requested)
 
 
 
 
 
 
m_numToSelect 
 
int  m_numToSelect  
 
 The number of attributes to retain if a ranking is requested. -1
      indicates that all attributes are to be retained. Has precedence over
      m_threshold
 
 
 
 
 
 
m_calculatedNumToSelect 
 
int  m_calculatedNumToSelect  
 
 
 
 
 
 
m_threshold 
 
double  m_threshold  
 
 the threshold for removing attributes if ranking is requested
 
 
 
 

 
   
 
 
  
 Class  weka.attributeSelection.RandomSearch  extends  ASSearch  implements Serializable   
 
 

 
 serialVersionUID:  7479392617377425484L

 
   
 
 
  
 Serialized Fields   
 
 

 
m_starting 
 
int[]  m_starting  
 
 holds a starting set as an array of attributes.
 
 
 
 
 
 
m_startRange 
 
 Range   m_startRange  
 
 holds the start set as a range
 
 
 
 
 
 
m_bestGroup 
 
java.util.BitSet  m_bestGroup  
 
 the best feature set found during the search
 
 
 
 
 
 
m_bestMerit 
 
double  m_bestMerit  
 
 the merit of the best subset found
 
 
 
 
 
 
m_onlyConsiderBetterAndSmaller 
 
boolean  m_onlyConsiderBetterAndSmaller  
 
 only accept a feature set as being "better" than the best if its
 merit is better or equal to the best, and it contains fewer
 features than the best (this allows LVF to be implimented).
 
 
 
 
 
 
m_hasClass 
 
boolean  m_hasClass  
 
 does the data have a class
 
 
 
 
 
 
m_classIndex 
 
int  m_classIndex  
 
 holds the class index
 
 
 
 
 
 
m_numAttribs 
 
int  m_numAttribs  
 
 number of attributes in the data
 
 
 
 
 
 
m_seed 
 
int  m_seed  
 
 seed for random number generation
 
 
 
 
 
 
m_searchSize 
 
double  m_searchSize  
 
 percentage of the search space to consider
 
 
 
 
 
 
m_iterations 
 
int  m_iterations  
 
 the number of iterations performed
 
 
 
 
 
 
m_random 
 
java.util.Random  m_random  
 
 random number object
 
 
 
 
 
 
m_verbose 
 
boolean  m_verbose  
 
 output new best subsets as the search progresses
 
 
 
 

 
   
 
 
  
 Class  weka.attributeSelection.Ranker  extends  ASSearch  implements Serializable   
 
 

 
 serialVersionUID:  -9086714848510751934L

 
   
 
 
  
 Serialized Fields   
 
 

 
m_starting 
 
int[]  m_starting  
 
 Holds the starting set as an array of attributes
 
 
 
 
 
 
m_startRange 
 
 Range   m_startRange  
 
 Holds the start set for the search as a range
 
 
 
 
 
 
m_attributeList 
 
int[]  m_attributeList  
 
 Holds the ordered list of attributes
 
 
 
 
 
 
m_attributeMerit 
 
double[]  m_attributeMerit  
 
 Holds the list of attribute merit scores
 
 
 
 
 
 
m_hasClass 
 
boolean  m_hasClass  
 
 Data has class attribute---if unsupervised evaluator then no class
 
 
 
 
 
 
m_classIndex 
 
int  m_classIndex  
 
 Class index of the data if supervised evaluator
 
 
 
 
 
 
m_numAttribs 
 
int  m_numAttribs  
 
 The number of attribtes
 
 
 
 
 
 
m_threshold 
 
double  m_threshold  
 
 A threshold by which to discard attributes---used by the
 AttributeSelection module
 
 
 
 
 
 
m_numToSelect 
 
int  m_numToSelect  
 
 The number of attributes to select. -1 indicates that all attributes
      are to be retained. Has precedence over m_threshold
 
 
 
 
 
 
m_calculatedNumToSelect 
 
int  m_calculatedNumToSelect  
 
 Used to compute the number to select
 
 
 
 

 
   
 
 
  
 Class  weka.attributeSelection.RankSearch  extends  ASSearch  implements Serializable   
 
 

 
 serialVersionUID:  -7992268736874353755L

 
   
 
 
  
 Serialized Fields   
 
 

 
m_hasClass 
 
boolean  m_hasClass  
 
 does the data have a class
 
 
 
 
 
 
m_classIndex 
 
int  m_classIndex  
 
 holds the class index
 
 
 
 
 
 
m_numAttribs 
 
int  m_numAttribs  
 
 number of attributes in the data
 
 
 
 
 
 
m_best_group 
 
java.util.BitSet  m_best_group  
 
 the best subset found
 
 
 
 
 
 
m_ASEval 
 
 ASEvaluation   m_ASEval  
 
 the attribute evaluator to use for generating the ranking
 
 
 
 
 
 
m_SubsetEval 
 
 ASEvaluation   m_SubsetEval  
 
 the subset evaluator with which to evaluate the ranking
 
 
 
 
 
 
m_Instances 
 
 Instances   m_Instances  
 
 the training instances
 
 
 
 
 
 
m_bestMerit 
 
double  m_bestMerit  
 
 the merit of the best subset found
 
 
 
 
 
 
m_Ranking 
 
int[]  m_Ranking  
 
 will hold the attribute ranking
 
 
 
 
 
 
m_add 
 
int  m_add  
 
 add this many attributes in each iteration from the ranking
 
 
 
 
 
 
m_startPoint 
 
int  m_startPoint  
 
 start from this point in the ranking
 
 
 
 

 
   
 
 
  
 Class  weka.attributeSelection.ReliefFAttributeEval  extends  ASEvaluation  implements Serializable   
 
 

 
 serialVersionUID:  -8422186665795839379L

 
   
 
 
  
 Serialized Fields   
 
 

 
m_trainInstances 
 
 Instances   m_trainInstances  
 
 The training instances
 
 
 
 
 
 
m_classIndex 
 
int  m_classIndex  
 
 The class index
 
 
 
 
 
 
m_numAttribs 
 
int  m_numAttribs  
 
 The number of attributes
 
 
 
 
 
 
m_numInstances 
 
int  m_numInstances  
 
 The number of instances
 
 
 
 
 
 
m_numericClass 
 
boolean  m_numericClass  
 
 Numeric class
 
 
 
 
 
 
m_numClasses 
 
int  m_numClasses  
 
 The number of classes if class is nominal
 
 
 
 
 
 
m_ndc 
 
double  m_ndc  
 
 Used to hold the probability of a different class val given nearest
 instances (numeric class)
 
 
 
 
 
 
m_nda 
 
double[]  m_nda  
 
 Used to hold the prob of different value of an attribute given
 nearest instances (numeric class case)
 
 
 
 
 
 
m_ndcda 
 
double[]  m_ndcda  
 
 Used to hold the prob of a different class val and different att
 val given nearest instances (numeric class case)
 
 
 
 
 
 
m_weights 
 
double[]  m_weights  
 
 Holds the weights that relief assigns to attributes
 
 
 
 
 
 
m_classProbs 
 
double[]  m_classProbs  
 
 Prior class probabilities (discrete class case)
 
 
 
 
 
 
m_sampleM 
 
int  m_sampleM  
 
 The number of instances to sample when estimating attributes
 default == -1, use all instances
 
 
 
 
 
 
m_Knn 
 
int  m_Knn  
 
 The number of nearest hits/misses
 
 
 
 
 
 
m_karray 
 
double[][][]  m_karray  
 
 k nearest scores + instance indexes for n classes
 
 
 
 
 
 
m_maxArray 
 
double[]  m_maxArray  
 
 Upper bound for numeric attributes
 
 
 
 
 
 
m_minArray 
 
double[]  m_minArray  
 
 Lower bound for numeric attributes
 
 
 
 
 
 
m_worst 
 
double[]  m_worst  
 
 Keep track of the farthest instance for each class
 
 
 
 
 
 
m_index 
 
int[]  m_index  
 
 Index in the m_karray of the farthest instance for each class
 
 
 
 
 
 
m_stored 
 
int[]  m_stored  
 
 Number of nearest neighbours stored of each class
 
 
 
 
 
 
m_seed 
 
int  m_seed  
 
 Random number seed used for sampling instances
 
 
 
 
 
 
m_weightsByRank 
 
double[]  m_weightsByRank  
 
 used to (optionally) weight nearest neighbours by their distance
  from the instance in question. Each entry holds 
  exp(-((rank(r_i, i_j)/sigma)^2)) where rank(r_i,i_j) is the rank of
  instance i_j in a sequence of instances ordered by the distance
  from r_i. sigma is a user defined parameter, default=20
 
 
 
 
 
 
m_sigma 
 
int  m_sigma  
 
 
 
 
 
 
m_weightByDistance 
 
boolean  m_weightByDistance  
 
 Weight by distance rather than equal weights
 
 
 
 

 
   
 
 
  
 Class  weka.attributeSelection.ScatterSearchV1  extends  ASSearch  implements Serializable   
 
 

 
 serialVersionUID:  -8512041420388121326L

 
   
 
 
  
 Serialized Fields   
 
 

 
m_numAttribs 
 
int  m_numAttribs  
 
 number of attributes in the data
 
 
 
 
 
 
m_classIndex 
 
int  m_classIndex  
 
 holds the class index
 
 
 
 
 
 
m_treshold 
 
double  m_treshold  
 
 holds the treshhold that delimits the good attributes
 
 
 
 
 
 
m_initialThreshold 
 
double  m_initialThreshold  
 
 the initial threshold
 
 
 
 
 
 
m_typeOfCombination 
 
int  m_typeOfCombination  
 
 the kind of comination betwen parents ((0)greedy combination/(1)reduced greedy combination)
 
 
 
 
 
 
m_random 
 
java.util.Random  m_random  
 
 random number generation
 
 
 
 
 
 
m_seed 
 
int  m_seed  
 
 seed for random number generation
 
 
 
 
 
 
m_debug 
 
boolean  m_debug  
 
 verbose output for monitoring the search and debugging
 
 
 
 
 
 
m_InformationReports 
 
java.lang.StringBuffer  m_InformationReports  
 
 holds a report of the search
 
 
 
 
 
 
m_totalEvals 
 
int  m_totalEvals  
 
 total number of subsets evaluated during a search
 
 
 
 
 
 
m_bestMerit 
 
double  m_bestMerit  
 
 holds the merit of the best subset found
 
 
 
 
 
 
m_processinTime 
 
long  m_processinTime  
 
 time for procesing the search method
 
 
 
 
 
 
m_population 
 
java.util.List<E>  m_population  
 
 holds the Initial Population of Subsets
 
 
 
 
 
 
m_popSize 
 
int  m_popSize  
 
 holds the population size
 
 
 
 
 
 
m_initialPopSize 
 
int  m_initialPopSize  
 
 holds the user selected initial population size
 
 
 
 
 
 
m_calculatedInitialPopSize 
 
int  m_calculatedInitialPopSize  
 
 if no initial user pop size, then this holds the initial
 pop size calculated from the number of attributes in the data
 (for use in the toString() method)
 
 
 
 
 
 
m_attributeRanking 
 
java.util.List<E>  m_attributeRanking  
 
 holds the attributes ranked
 
 
 
 
 
 
ASEvaluator 
 
 SubsetEvaluator   ASEvaluator  
 
 Evaluator used to know the significance of a subset (for guiding the search)
 
 
 
 

 
   
 
 
  
 Class  weka.attributeSelection.ScatterSearchV1.Subset  extends java.lang.Object implements Serializable   
 
 

 
   
 
 
  
 Serialized Fields   
 
 

 
merit 
 
double  merit  
 
 
 
 
 
 
subset 
 
java.util.BitSet  subset  
 
 
 
 

 
   
 
 
  
 Class  weka.attributeSelection.SubsetSizeForwardSelection  extends  ASSearch  implements Serializable   
 
 

 
   
 
 
  
 Serialized Fields   
 
 

 
m_performRanking 
 
boolean  m_performRanking  
 
 perform initial ranking to select top-ranked attributes
 
 
 
 
 
 
m_numUsedAttributes 
 
int  m_numUsedAttributes  
 
 number of top-ranked attributes that are taken into account for the
 search
 
 
 
 
 
 
m_linearSelectionType 
 
int  m_linearSelectionType  
 
 0 == fixed-set, 1 == fixed-width
 
 
 
 
 
 
m_setSizeEval 
 
 ASEvaluation   m_setSizeEval  
 
 the subset evaluator to use for subset size determination
 
 
 
 
 
 
m_numFolds 
 
int  m_numFolds  
 
 Number of cross validation folds for subset size determination (default =
 5).
 
 
 
 
 
 
m_seed 
 
int  m_seed  
 
 Seed for cross validation subset size determination. (default = 1)
 
 
 
 
 
 
m_numAttribs 
 
int  m_numAttribs  
 
 number of attributes in the data
 
 
 
 
 
 
m_totalEvals 
 
int  m_totalEvals  
 
 total number of subsets evaluated during a search
 
 
 
 
 
 
m_verbose 
 
boolean  m_verbose  
 
 for debugging
 
 
 
 
 
 
m_bestMerit 
 
double  m_bestMerit  
 
 holds the merit of the best subset found
 
 
 
 
 
 
m_cacheSize 
 
int  m_cacheSize  
 
 holds the maximum size of the lookup cache for evaluated subsets
 
 
 
 

 
   
 
 
  
 Class  weka.attributeSelection.SVMAttributeEval  extends  ASEvaluation  implements Serializable   
 
 

 
 serialVersionUID:  -6489975709033967447L

 
   
 
 
  
 Serialized Fields   
 
 

 
m_attScores 
 
double[]  m_attScores  
 
 The attribute scores
 
 
 
 
 
 
m_numToEliminate 
 
int  m_numToEliminate  
 
 Constant rate of attribute elimination per iteration
 
 
 
 
 
 
m_percentToEliminate 
 
int  m_percentToEliminate  
 
 Percentage rate of attribute elimination, trumps constant
      rate (above threshold), ignored if = 0
 
 
 
 
 
 
m_percentThreshold 
 
int  m_percentThreshold  
 
 Threshold below which percent elimination switches to
      constant elimination
 
 
 
 
 
 
m_smoCParameter 
 
double  m_smoCParameter  
 
 Complexity parameter to pass on to SMO
 
 
 
 
 
 
m_smoTParameter 
 
double  m_smoTParameter  
 
 Tolerance parameter to pass on to SMO
 
 
 
 
 
 
m_smoPParameter 
 
double  m_smoPParameter  
 
 Epsilon parameter to pass on to SMO
 
 
 
 
 
 
m_smoFilterType 
 
int  m_smoFilterType  
 
 Filter parameter to pass on to SMO
 
 
 
 

 
   
 
 
  
 Class  weka.attributeSelection.SymmetricalUncertAttributeEval  extends  ASEvaluation  implements Serializable   
 
 

 
 serialVersionUID:  -8096505776132296416L

 
   
 
 
  
 Serialized Fields   
 
 

 
m_trainInstances 
 
 Instances   m_trainInstances  
 
 The training instances
 
 
 
 
 
 
m_classIndex 
 
int  m_classIndex  
 
 The class index
 
 
 
 
 
 
m_numAttribs 
 
int  m_numAttribs  
 
 The number of attributes
 
 
 
 
 
 
m_numInstances 
 
int  m_numInstances  
 
 The number of instances
 
 
 
 
 
 
m_numClasses 
 
int  m_numClasses  
 
 The number of classes
 
 
 
 
 
 
m_missing_merge 
 
boolean  m_missing_merge  
 
 Treat missing values as a seperate value
 
 
 
 

 
   
 
 
  
 Class  weka.attributeSelection.UnsupervisedAttributeEvaluator  extends  ASEvaluation  implements Serializable   
 
 

 
 serialVersionUID:  -4100897318675336178L

 

 
   
 
 
  
 Class  weka.attributeSelection.UnsupervisedSubsetEvaluator  extends  ASEvaluation  implements Serializable   
 
 

 
 serialVersionUID:  627934376267488763L

 

 
   
 
 
  
 Class  weka.attributeSelection.WrapperSubsetEval  extends  ASEvaluation  implements Serializable   
 
 

 
 serialVersionUID:  -4573057658746728675L

 
   
 
 
  
 Serialized Fields   
 
 

 
m_trainInstances 
 
 Instances   m_trainInstances  
 
 training instances
 
 
 
 
 
 
m_classIndex 
 
int  m_classIndex  
 
 class index
 
 
 
 
 
 
m_numAttribs 
 
int  m_numAttribs  
 
 number of attributes in the training data
 
 
 
 
 
 
m_numInstances 
 
int  m_numInstances  
 
 number of instances in the training data
 
 
 
 
 
 
m_Evaluation 
 
 Evaluation   m_Evaluation  
 
 holds an evaluation object
 
 
 
 
 
 
m_BaseClassifier 
 
 Classifier   m_BaseClassifier  
 
 holds the base classifier object
 
 
 
 
 
 
m_folds 
 
int  m_folds  
 
 number of folds to use for cross validation
 
 
 
 
 
 
m_seed 
 
int  m_seed  
 
 random number seed
 
 
 
 
 
 
m_threshold 
 
double  m_threshold  
 
 the threshold by which to do further cross validations when
 estimating the accuracy of a subset
 
 
 
 
 

 
 
  
 Package   weka.classifiers   
 
 

 
   
 
 
  
 Class  weka.classifiers.Classifier  extends java.lang.Object implements Serializable   
 
 

 
 serialVersionUID:  6502780192411755341L

 
   
 
 
  
 Serialized Fields   
 
 

 
m_Debug 
 
boolean  m_Debug  
 
 Whether the classifier is run in debug mode.
 
 
 
 

 
   
 
 
  
 Class  weka.classifiers.CostMatrix  extends java.lang.Object implements Serializable   
 
 

 
 serialVersionUID:  -1973792250544554965L

 
   
 
 
  
 Serialized Fields   
 
 

 
m_size 
 
int  m_size  
 
 
 
 
 
 
m_matrix 
 
java.lang.Object[][]  m_matrix  
 
 [rows][columns]
 
 
 
 

 
   
 
 
  
 Class  weka.classifiers.IteratedSingleClassifierEnhancer  extends  SingleClassifierEnhancer  implements Serializable   
 
 

 
 serialVersionUID:  -6217979135443319724L

 
   
 
 
  
 Serialized Fields   
 
 

 
m_Classifiers 
 
 Classifier []  m_Classifiers  
 
 Array for storing the generated base classifiers.
 
 
 
 
 
 
m_NumIterations 
 
int  m_NumIterations  
 
 The number of iterations.
 
 
 
 

 
   
 
 
  
 Class  weka.classifiers.MultipleClassifiersCombiner  extends  Classifier  implements Serializable   
 
 

 
 serialVersionUID:  2776436621129422119L

 
   
 
 
  
 Serialized Fields   
 
 

 
m_Classifiers 
 
 Classifier []  m_Classifiers  
 
 Array for storing the generated base classifiers.
 
 
 
 

 
   
 
 
  
 Class  weka.classifiers.RandomizableClassifier  extends  Classifier  implements Serializable   
 
 

 
 serialVersionUID:  -8816375798262351903L

 
   
 
 
  
 Serialized Fields   
 
 

 
m_Seed 
 
int  m_Seed  
 
 The random number seed.
 
 
 
 

 
   
 
 
  
 Class  weka.classifiers.RandomizableIteratedSingleClassifierEnhancer  extends  IteratedSingleClassifierEnhancer  implements Serializable   
 
 

 
 serialVersionUID:  5063351391524938557L

 
   
 
 
  
 Serialized Fields   
 
 

 
m_Seed 
 
int  m_Seed  
 
 The random number seed.
 
 
 
 

 
   
 
 
  
 Class  weka.classifiers.RandomizableMultipleClassifiersCombiner  extends  MultipleClassifiersCombiner  implements Serializable   
 
 

 
 serialVersionUID:  5057936555724785679L

 
   
 
 
  
 Serialized Fields   
 
 

 
m_Seed 
 
int  m_Seed  
 
 The random number seed.
 
 
 
 

 
   
 
 
  
 Class  weka.classifiers.RandomizableSingleClassifierEnhancer  extends  SingleClassifierEnhancer  implements Serializable   
 
 

 
 serialVersionUID:  558286687096157160L

 
   
 
 
  
 Serialized Fields   
 
 

 
m_Seed 
 
int  m_Seed  
 
 The random number seed.
 
 
 
 

 
   
 
 
  
 Class  weka.classifiers.SingleClassifierEnhancer  extends  Classifier  implements Serializable   
 
 

 
 serialVersionUID:  -3665885256363525164L

 
   
 
 
  
 Serialized Fields   
 
 

 
m_Classifier 
 
 Classifier   m_Classifier  
 
 The base classifier to use
 
 
 
 
 

 
 
  
 Package   weka.classifiers.bayes   
 
 

 
   
 
 
  
 Class  weka.classifiers.bayes.AODE  extends  Classifier  implements Serializable   
 
 

 
 serialVersionUID:  9197439980415113523L

 
   
 
 
  
 Serialized Fields   
 
 

 
m_CondiCounts 
 
double[][][]  m_CondiCounts  
 
 3D array (m_NumClasses * m_TotalAttValues * m_TotalAttValues)
 of attribute counts, i.e., the number of times an attribute value occurs
 in conjunction with another attribute value and a class value.
 
 
 
 
 
 
m_ClassCounts 
 
double[]  m_ClassCounts  
 
 The number of times each class value occurs in the dataset
 
 
 
 
 
 
m_SumForCounts 
 
double[][]  m_SumForCounts  
 
 The sums of attribute-class counts  
    -- if there are no missing values for att, then
       m_SumForCounts[classVal][att] will be the same as
       m_ClassCounts[classVal]
 
 
 
 
 
 
m_NumClasses 
 
int  m_NumClasses  
 
 The number of classes
 
 
 
 
 
 
m_NumAttributes 
 
int  m_NumAttributes  
 
 The number of attributes in dataset, including class
 
 
 
 
 
 
m_NumInstances 
 
int  m_NumInstances  
 
 The number of instances in the dataset
 
 
 
 
 
 
m_ClassIndex 
 
int  m_ClassIndex  
 
 The index of the class attribute
 
 
 
 
 
 
m_Instances 
 
 Instances   m_Instances  
 
 The dataset
 
 
 
 
 
 
m_TotalAttValues 
 
int  m_TotalAttValues  
 
 The total number of values (including an extra for each attribute's 
 missing value, which are included in m_CondiCounts) for all attributes 
 (not including class). E.g., for three atts each with two possible values,
 m_TotalAttValues would be 9 (6 values + 3 missing).
 This variable is used when allocating space for m_CondiCounts matrix.
 
 
 
 
 
 
m_StartAttIndex 
 
int[]  m_StartAttIndex  
 
 The starting index (in the m_CondiCounts matrix) of the values for each
 attribute
 
 
 
 
 
 
m_NumAttValues 
 
int[]  m_NumAttValues  
 
 The number of values for each attribute
 
 
 
 
 
 
m_Frequencies 
 
double[]  m_Frequencies  
 
 The frequency of each attribute value for the dataset
 
 
 
 
 
 
m_SumInstances 
 
double  m_SumInstances  
 
 The number of valid class values observed in dataset 
  -- with no missing classes, this number is the same as m_NumInstances.
 
 
 
 
 
 
m_Limit 
 
int  m_Limit  
 
 An att's frequency must be this value or more to be a superParent
 
 
 
 
 
 
m_Debug 
 
boolean  m_Debug  
 
 If true, outputs debugging info
 
 
 
 
 
 
m_MEstimates 
 
boolean  m_MEstimates  
 
 flag for using m-estimates
 
 
 
 
 
 
m_Weight 
 
int  m_Weight  
 
 value for m in m-estimate
 
 
 
 

 
   
 
 
  
 Class  weka.classifiers.bayes.AODEsr  extends  Classifier  implements Serializable   
 
 

 
 serialVersionUID:  5602143019183068848L

 
   
 
 
  
 Serialized Fields   
 
 

 
m_CondiCounts 
 
double[][][]  m_CondiCounts  
 
 3D array (m_NumClasses * m_TotalAttValues * m_TotalAttValues)
 of attribute counts, i.e. the number of times an attribute value occurs
 in conjunction with another attribute value and a class value.
 
 
 
 
 
 
m_CondiCountsNoClass 
 
double[][]  m_CondiCountsNoClass  
 
 2D array (m_TotalAttValues * m_TotalAttValues) of attributes counts.
 similar to m_CondiCounts, but ignoring class value.
 
 
 
 
 
 
m_ClassCounts 
 
double[]  m_ClassCounts  
 
 The number of times each class value occurs in the dataset
 
 
 
 
 
 
m_SumForCounts 
 
double[][]  m_SumForCounts  
 
 The sums of attribute-class counts  
    -- if there are no missing values for att, then
       m_SumForCounts[classVal][att] will be the same as
       m_ClassCounts[classVal]
 
 
 
 
 
 
m_NumClasses 
 
int  m_NumClasses  
 
 The number of classes
 
 
 
 
 
 
m_NumAttributes 
 
int  m_NumAttributes  
 
 The number of attributes in dataset, including class
 
 
 
 
 
 
m_NumInstances 
 
int  m_NumInstances  
 
 The number of instances in the dataset
 
 
 
 
 
 
m_ClassIndex 
 
int  m_ClassIndex  
 
 The index of the class attribute
 
 
 
 
 
 
m_Instances 
 
 Instances   m_Instances  
 
 The dataset
 
 
 
 
 
 
m_TotalAttValues 
 
int  m_TotalAttValues  
 
 The total number of values (including an extra for each attribute's 
 missing value, which are included in m_CondiCounts) for all attributes 
 (not including class).  Eg. for three atts each with two possible values,
 m_TotalAttValues would be 9 (6 values + 3 missing).
 This variable is used when allocating space for m_CondiCounts matrix.
 
 
 
 
 
 
m_StartAttIndex 
 
int[]  m_StartAttIndex  
 
 The starting index (in the m_CondiCounts matrix) of the values for each attribute
 
 
 
 
 
 
m_NumAttValues 
 
int[]  m_NumAttValues  
 
 The number of values for each attribute
 
 
 
 
 
 
m_Frequencies 
 
double[]  m_Frequencies  
 
 The frequency of each attribute value for the dataset
 
 
 
 
 
 
m_SumInstances 
 
double  m_SumInstances  
 
 The number of valid class values observed in dataset 
  -- with no missing classes, this number is the same as m_NumInstances.
 
 
 
 
 
 
m_Limit 
 
int  m_Limit  
 
 An att's frequency must be this value or more to be a superParent
 
 
 
 
 
 
m_Debug 
 
boolean  m_Debug  
 
 If true, outputs debugging info
 
 
 
 
 
 
m_MWeight 
 
double  m_MWeight  
 
 m value for m-estimation
 
 
 
 
 
 
m_Laplace 
 
boolean  m_Laplace  
 
 Using LapLace estimation or not
 
 
 
 
 
 
m_Critical 
 
int  m_Critical  
 
 the critical value for the specialization-generalization
 
 
 
 

 
   
 
 
  
 Class  weka.classifiers.bayes.BayesianLogisticRegression  extends  Classifier  implements Serializable   
 
 

 
 serialVersionUID:  -8013478897911757631L

 
   
 
 
  
 Serialized Fields   
 
 

 
debug 
 
boolean  debug  
 
 DEBUG Mode
 
 
 
 
 
 
NormalizeData 
 
boolean  NormalizeData  
 
 Choose whether to normalize data or not
 
 
 
 
 
 
Tolerance 
 
double  Tolerance  
 
 Tolerance criteria for the stopping criterion.
 
 
 
 
 
 
Threshold 
 
double  Threshold  
 
 Threshold for binary classification of probabilisitic estimate
 
 
 
 
 
 
PriorClass 
 
int  PriorClass  
 
 Distribution Prior class
 
 
 
 
 
 
NumFolds 
 
int  NumFolds  
 
 NumFolds for CV based Hyperparameters selection
 
 
 
 
 
 
HyperparameterSelection 
 
int  HyperparameterSelection  
 
 Hyperparameter selection method
 
 
 
 
 
 
ClassIndex 
 
int  ClassIndex  
 
 The class index from the training data
 
 
 
 
 
 
HyperparameterValue 
 
double  HyperparameterValue  
 
 Best hyperparameter for test phase
 
 
 
 
 
 
HyperparameterRange 
 
java.lang.String  HyperparameterRange  
 
 CV Hyperparameter Range
 
 
 
 
 
 
maxIterations 
 
int  maxIterations  
 
 Maximum number of iterations
 
 
 
 
 
 
iterationCounter 
 
int  iterationCounter  
 
 Iteration counter
 
 
 
 
 
 
BetaVector 
 
double[]  BetaVector  
 
 Array for storing coefficients of Bayesian regression model.
 
 
 
 
 
 
DeltaBeta 
 
double[]  DeltaBeta  
 
 Array to store Regression Coefficient updates.
 
 
 
 
 
 
DeltaUpdate 
 
double[]  DeltaUpdate  
 
 Trust Region Radius Update
 
 
 
 
 
 
Delta 
 
double[]  Delta  
 
 Trust Region Radius
 
 
 
 
 
 
Hyperparameters 
 
double[]  Hyperparameters  
 
 Array to store Hyperparameter values for each feature.
 
 
 
 
 
 
R 
 
double[]  R  
 
 R(i)= BetaVector X x(i) X y(i).
 This an intermediate value with respect to vector BETA, input values and corresponding class labels
 
 
 
 
 
 
DeltaR 
 
double[]  DeltaR  
 
 This vector is used to store the increments on the R(i). It is also used to determining the stopping criterion.
 
 
 
 
 
 
Change 
 
double  Change  
 
 This variable is used to keep track of change in
 the value of delta summation of r(i).
 
 
 
 
 
 
m_Filter 
 
 Filter   m_Filter  
 
 Filter interface used to point to weka.filters.unsupervised.attribute.Normalize object
 
 
 
 
 
 
m_Instances 
 
 Instances   m_Instances  
 
 Dataset provided to do Training/Test set.
 
 
 
 
 
 
m_PriorUpdate 
 
 Prior   m_PriorUpdate  
 
 Prior class object interface
 
 
 
 

 
   
 
 
  
 Class  weka.classifiers.bayes.BayesNet  extends  Classifier  implements Serializable   
 
 

 
 serialVersionUID:  746037443258775954L

 
   
 
 
  
 Serialized Fields   
 
 

 
m_ParentSets 
 
 ParentSet []  m_ParentSets  
 
 The parent sets.
 
 
 
 
 
 
m_Distributions 
 
 Estimator [][]  m_Distributions  
 
 The attribute estimators containing CPTs.
 
 
 
 
 
 
m_DiscretizeFilter 
 
 Discretize   m_DiscretizeFilter  
 
 filter used to quantize continuous variables, if any
 
 
 
 
 
 
m_nNonDiscreteAttribute 
 
int  m_nNonDiscreteAttribute  
 
 attribute index of a non-nominal attribute
 
 
 
 
 
 
m_MissingValuesFilter 
 
 ReplaceMissingValues   m_MissingValuesFilter  
 
 filter used to fill in missing values, if any
 
 
 
 
 
 
m_NumClasses 
 
int  m_NumClasses  
 
 The number of classes
 
 
 
 
 
 
m_Instances 
 
 Instances   m_Instances  
 
 The dataset header for the purposes of printing out a semi-intelligible
 model
 
 
 
 
 
 
m_ADTree 
 
 ADNode   m_ADTree  
 
 Datastructure containing ADTree representation of the database.
 This may result in more efficient access to the data.
 
 
 
 
 
 
m_otherBayesNet 
 
 BIFReader   m_otherBayesNet  
 
 Bayes network to compare the structure with.
 
 
 
 
 
 
m_bUseADTree 
 
boolean  m_bUseADTree  
 
 Use the experimental ADTree datastructure for calculating contingency tables
 
 
 
 
 
 
m_SearchAlgorithm 
 
 SearchAlgorithm   m_SearchAlgorithm  
 
 Search algorithm used for learning the structure of a network.
 
 
 
 
 
 
m_BayesNetEstimator 
 
 BayesNetEstimator   m_BayesNetEstimator  
 
 Search algorithm used for learning the structure of a network.
 
 
 
 

 
   
 
 
  
 Class  weka.classifiers.bayes.ComplementNaiveBayes  extends  Classifier  implements Serializable   
 
 

 
 serialVersionUID:  7246302925903086397L

 
   
 
 
  
 Serialized Fields   
 
 

 
wordWeights 
 
double[][]  wordWeights  
 
 Weight of words for each class. The weight is actually the
      log of the probability of a word (w) given a class (c) 
      (i.e. log(Pr[w|c])). The format of the matrix is: 
      wordWeights[class][wordAttribute]
 
 
 
 
 
 
smoothingParameter 
 
double  smoothingParameter  
 
 Holds the smoothing value to avoid word probabilities of zero. 
        P.S.: According to the paper this is the Alpha i parameter
 
 
 
 
 
 
m_normalizeWordWeights 
 
boolean  m_normalizeWordWeights  
 
 True if the words weights are to be normalized
 
 
 
 
 
 
numClasses 
 
int  numClasses  
 
 Holds the number of Class values present in the set of specified 
        instances
 
 
 
 
 
 
header 
 
 Instances   header  
 
 The instances header that'll be used in toString
 
 
 
 

 
   
 
 
  
 Class  weka.classifiers.bayes.DMNBtext  extends  Classifier  implements Serializable   
 
 

 
 serialVersionUID:  5932177450183457085L

 
   
 
 
  
 Serialized Fields   
 
 

 
m_NumIterations 
 
int  m_NumIterations  
 
 The number of iterations.
 
 
 
 
 
 
m_MultinomialWord 
 
boolean  m_MultinomialWord  
 
 
 
 
 
 
m_numClasses 
 
int  m_numClasses  
 
 
 
 
 
 
m_headerInfo 
 
 Instances   m_headerInfo  
 
 
 
 
 
 
m_binaryClassifiers 
 
 DMNBtext.DNBBinary []  m_binaryClassifiers  
 
 
 
 

 
   
 
 
  
 Class  weka.classifiers.bayes.DMNBtext.DNBBinary  extends java.lang.Object implements Serializable   
 
 

 
   
 
 
  
 Serialized Fields   
 
 

 
m_perWordPerClass 
 
double[][]  m_perWordPerClass  
 
 The number of iterations.
 
 
 
 
 
 
m_wordsPerClass 
 
double[]  m_wordsPerClass  
 
 
 
 
 
 
m_classIndex 
 
int  m_classIndex  
 
 
 
 
 
 
m_classDistribution 
 
double[]  m_classDistribution  
 
 
 
 
 
 
m_numAttributes 
 
int  m_numAttributes  
 
 number of unique words
 
 
 
 
 
 
m_targetClass 
 
int  m_targetClass  
 
 
 
 
 
 
m_WordLaplace 
 
double  m_WordLaplace  
 
 
 
 
 
 
m_coefficient 
 
double[]  m_coefficient  
 
 
 
 
 
 
m_classRatio 
 
double  m_classRatio  
 
 
 
 
 
 
m_wordRatio 
 
double  m_wordRatio  
 
 
 
 

 
   
 
 
  
 Class  weka.classifiers.bayes.HNB  extends  Classifier  implements Serializable   
 
 

 
 serialVersionUID:  -4503874444306113214L

 
   
 
 
  
 Serialized Fields   
 
 

 
m_ClassCounts 
 
double[]  m_ClassCounts  
 
 The number of each class value occurs in the dataset
 
 
 
 
 
 
m_ClassAttAttCounts 
 
double[][][]  m_ClassAttAttCounts  
 
 The number of class and two attributes values occurs in the dataset
 
 
 
 
 
 
m_NumAttValues 
 
int[]  m_NumAttValues  
 
 The number of values for each attribute in the dataset
 
 
 
 
 
 
m_TotalAttValues 
 
int  m_TotalAttValues  
 
 The number of values for all attributes in the dataset
 
 
 
 
 
 
m_NumClasses 
 
int  m_NumClasses  
 
 The number of classes in the dataset
 
 
 
 
 
 
m_NumAttributes 
 
int  m_NumAttributes  
 
 The number of attributes including class in the dataset
 
 
 
 
 
 
m_NumInstances 
 
int  m_NumInstances  
 
 The number of instances in the dataset
 
 
 
 
 
 
m_ClassIndex 
 
int  m_ClassIndex  
 
 The index of the class attribute in the dataset
 
 
 
 
 
 
m_StartAttIndex 
 
int[]  m_StartAttIndex  
 
 The starting index of each attribute in the dataset
 
 
 
 
 
 
m_condiMutualInfo 
 
double[][]  m_condiMutualInfo  
 
 The 2D array of conditional mutual information of each pair attributes
 
 
 
 

 
   
 
 
  
 Class  weka.classifiers.bayes.NaiveBayes  extends  Classifier  implements Serializable   
 
 

 
 serialVersionUID:  5995231201785697655L

 
   
 
 
  
 Serialized Fields   
 
 

 
m_Distributions 
 
 Estimator [][]  m_Distributions  
 
 The attribute estimators.
 
 
 
 
 
 
m_ClassDistribution 
 
 Estimator   m_ClassDistribution  
 
 The class estimator.
 
 
 
 
 
 
m_UseKernelEstimator 
 
boolean  m_UseKernelEstimator  
 
 Whether to use kernel density estimator rather than normal distribution
 for numeric attributes
 
 
 
 
 
 
m_UseDiscretization 
 
boolean  m_UseDiscretization  
 
 Whether to use discretization than normal distribution
 for numeric attributes
 
 
 
 
 
 
m_NumClasses 
 
int  m_NumClasses  
 
 The number of classes (or 1 for numeric class)
 
 
 
 
 
 
m_Instances 
 
 Instances   m_Instances  
 
 The dataset header for the purposes of printing out a semi-intelligible 
 model
 
 
 
 
 
 
m_Disc 
 
 Discretize   m_Disc  
 
 The discretization filter.
 
 
 
 
 
 
m_displayModelInOldFormat 
 
boolean  m_displayModelInOldFormat  
 
 
 
 

 
   
 
 
  
 Class  weka.classifiers.bayes.NaiveBayesMultinomial  extends  Classifier  implements Serializable   
 
 

 
 serialVersionUID:  5932177440181257085L

 
   
 
 
  
 Serialized Fields   
 
 

 
m_probOfWordGivenClass 
 
double[][]  m_probOfWordGivenClass  
 
 probability that a word (w) exists in a class (H) (i.e. Pr[w|H])
 The matrix is in the this format: probOfWordGivenClass[class][wordAttribute]
 NOTE: the values are actually the log of Pr[w|H]
 
 
 
 
 
 
m_probOfClass 
 
double[]  m_probOfClass  
 
 the probability of a class (i.e. Pr[H])
 
 
 
 
 
 
m_numAttributes 
 
int  m_numAttributes  
 
 number of unique words
 
 
 
 
 
 
m_numClasses 
 
int  m_numClasses  
 
 number of class values
 
 
 
 
 
 
m_lnFactorialCache 
 
double[]  m_lnFactorialCache  
 
 cache lnFactorial computations
 
 
 
 
 
 
m_headerInfo 
 
 Instances   m_headerInfo  
 
 copy of header information for use in toString method
 
 
 
 

 
   
 
 
  
 Class  weka.classifiers.bayes.NaiveBayesMultinomialUpdateable  extends  NaiveBayesMultinomial  implements Serializable   
 
 

 
 serialVersionUID:  -7204398796974263186L

 
   
 
 
  
 Serialized Fields   
 
 

 
m_wordsPerClass 
 
double[]  m_wordsPerClass  
 
 the word count per class
 
 
 
 

 
   
 
 
  
 Class  weka.classifiers.bayes.NaiveBayesSimple  extends  Classifier  implements Serializable   
 
 

 
 serialVersionUID:  -1478242251770381214L

 
   
 
 
  
 Serialized Fields   
 
 

 
m_Counts 
 
double[][][]  m_Counts  
 
 All the counts for nominal attributes.
 
 
 
 
 
 
m_Means 
 
double[][]  m_Means  
 
 The means for numeric attributes.
 
 
 
 
 
 
m_Devs 
 
double[][]  m_Devs  
 
 The standard deviations for numeric attributes.
 
 
 
 
 
 
m_Priors 
 
double[]  m_Priors  
 
 The prior probabilities of the classes.
 
 
 
 
 
 
m_Instances 
 
 Instances   m_Instances  
 
 The instances used for training.
 
 
 
 

 
   
 
 
  
 Class  weka.classifiers.bayes.NaiveBayesUpdateable  extends  NaiveBayes  implements Serializable   
 
 

 
 serialVersionUID:  -5354015843807192221L

 

 
   
 
 
  
 Class  weka.classifiers.bayes.WAODE  extends  Classifier  implements Serializable   
 
 

 
 serialVersionUID:  2170978824284697882L

 
   
 
 
  
 Serialized Fields   
 
 

 
m_ClassCounts 
 
double[]  m_ClassCounts  
 
 The number of each class value occurs in the dataset
 
 
 
 
 
 
m_AttCounts 
 
double[]  m_AttCounts  
 
 The number of each attribute value occurs in the dataset
 
 
 
 
 
 
m_AttAttCounts 
 
double[][]  m_AttAttCounts  
 
 The number of two attributes values occurs in the dataset
 
 
 
 
 
 
m_ClassAttAttCounts 
 
double[][][]  m_ClassAttAttCounts  
 
 The number of class and two attributes values occurs in the dataset
 
 
 
 
 
 
m_NumAttValues 
 
int[]  m_NumAttValues  
 
 The number of values for each attribute in the dataset
 
 
 
 
 
 
m_TotalAttValues 
 
int  m_TotalAttValues  
 
 The number of values for all attributes in the dataset
 
 
 
 
 
 
m_NumClasses 
 
int  m_NumClasses  
 
 The number of classes in the dataset
 
 
 
 
 
 
m_NumAttributes 
 
int  m_NumAttributes  
 
 The number of attributes including class in the dataset
 
 
 
 
 
 
m_NumInstances 
 
int  m_NumInstances  
 
 The number of instances in the dataset
 
 
 
 
 
 
m_ClassIndex 
 
int  m_ClassIndex  
 
 The index of the class attribute in the dataset
 
 
 
 
 
 
m_StartAttIndex 
 
int[]  m_StartAttIndex  
 
 The starting index of each attribute in the dataset
 
 
 
 
 
 
m_mutualInformation 
 
double[]  m_mutualInformation  
 
 The array of mutual information between each attribute and class
 
 
 
 
 
 
m_Header 
 
 Instances   m_Header  
 
 the header information of the training data
 
 
 
 
 
 
m_Internals 
 
boolean  m_Internals  
 
 whether to print more internals in the toString method
 
 
  See Also:    WAODE.toString()   
 
 
 
m_ZeroR 
 
 Classifier   m_ZeroR  
 
 a ZeroR model in case no model can be built from the data
 
 
 
 
 

 
 
  
 Package   weka.classifiers.bayes.blr   
 
 

 
   
 
 
  
 Class  weka.classifiers.bayes.blr.GaussianPriorImpl  extends  Prior  implements Serializable   
 
 

 
 serialVersionUID:  -2995684220141159223L

 

 
   
 
 
  
 Class  weka.classifiers.bayes.blr.LaplacePriorImpl  extends  Prior  implements Serializable   
 
 

 
 serialVersionUID:  2353576123257012607L

 
   
 
 
  
 Serialized Fields   
 
 

 
m_Instances 
 
 Instances   m_Instances  
 
 
 
 
 
 
Beta 
 
double  Beta  
 
 
 
 
 
 
Hyperparameter 
 
double  Hyperparameter  
 
 
 
 
 
 
DeltaUpdate 
 
double  DeltaUpdate  
 
 
 
 
 
 
R 
 
double[]  R  
 
 
 
 
 
 
Delta 
 
double  Delta  
 
 
 
 

 
   
 
 
  
 Class  weka.classifiers.bayes.blr.Prior  extends java.lang.Object implements Serializable   
 
 

 
   
 
 
  
 Serialized Fields   
 
 

 
m_Instances 
 
 Instances   m_Instances  
 
 
 
 
 
 
Beta 
 
double  Beta  
 
 
 
 
 
 
Hyperparameter 
 
double  Hyperparameter  
 
 
 
 
 
 
DeltaUpdate 
 
double  DeltaUpdate  
 
 
 
 
 
 
R 
 
double[]  R  
 
 
 
 
 
 
Delta 
 
double  Delta  
 
 
 
 
 
 
log_posterior 
 
double  log_posterior  
 
 
 
 
 
 
log_likelihood 
 
double  log_likelihood  
 
 
 
 
 
 
penalty 
 
double  penalty  
 
 
 
 
 

 
 
  
 Package   weka.classifiers.bayes.net   
 
 

 
   
 
 
  
 Class  weka.classifiers.bayes.net.ADNode  extends java.lang.Object implements Serializable   
 
 

 
 serialVersionUID:  397409728366910204L

 
   
 
 
  
 Serialized Fields   
 
 

 
m_VaryNodes 
 
 VaryNode []  m_VaryNodes  
 
 list of VaryNode children
 
 
 
 
 
 
m_Instances 
 
 Instance []  m_Instances  
 
 list of Instance children (either m_Instances or m_VaryNodes is instantiated)
 
 
 
 
 
 
m_nCount 
 
int  m_nCount  
 
 count
 
 
 
 
 
 
m_nStartNode 
 
int  m_nStartNode  
 
 first node in VaryNode array
 
 
 
 

 
   
 
 
  
 Class  weka.classifiers.bayes.net.BayesNetGenerator  extends  EditableBayesNet  implements Serializable   
 
 

 
 serialVersionUID:  -7462571170596157720L

 
   
 
 
  
 Serialized Fields   
 
 

 
m_nSeed 
 
int  m_nSeed  
 
 the seed value
 
 
 
 
 
 
random 
 
java.util.Random  random  
 
 the random number generator
 
 
 
 
 
 
m_bGenerateNet 
 
boolean  m_bGenerateNet  
 
 
 
 
 
 
m_nNrOfNodes 
 
int  m_nNrOfNodes  
 
 
 
 
 
 
m_nNrOfArcs 
 
int  m_nNrOfArcs  
 
 
 
 
 
 
m_nNrOfInstances 
 
int  m_nNrOfInstances  
 
 
 
 
 
 
m_nCardinality 
 
int  m_nCardinality  
 
 
 
 
 
 
m_sBIFFile 
 
java.lang.String  m_sBIFFile  
 
 
 
 

 
   
 
 
  
 Class  weka.classifiers.bayes.net.BIFReader  extends  BayesNet  implements Serializable   
 
 

 
 serialVersionUID:  -8358864680379881429L

 
   
 
 
  
 Serialized Fields   
 
 

 
m_nPositionX 
 
int[]  m_nPositionX  
 
 
 
 
 
 
m_nPositionY 
 
int[]  m_nPositionY  
 
 
 
 
 
 
m_order 
 
int[]  m_order  
 
 
 
 
 
 
m_sFile 
 
java.lang.String  m_sFile  
 
 the current filename
 
 
 
 

 
   
 
 
  
 Class  weka.classifiers.bayes.net.EditableBayesNet  extends  BayesNet  implements Serializable   
 
 

 
 serialVersionUID:  746037443258735954L

 
   
 
 
  
 Serialized Fields   
 
 

 
m_nPositionX 
 
 FastVector   m_nPositionX  
 
 location of nodes, used for graph drawing *
 
 
 
 
 
 
m_nPositionY 
 
 FastVector   m_nPositionY  
 
 
 
 
 
 
m_fMarginP 
 
 FastVector   m_fMarginP  
 
 marginal distributions *
 
 
 
 
 
 
m_nEvidence 
 
 FastVector   m_nEvidence  
 
 evidence values, used for evidence propagation *
 
 
 
 
 
 
m_undoStack 
 
 FastVector   m_undoStack  
 
 undo stack for undoin edit actions, or redo edit actions
 
 
 
 
 
 
m_nCurrentEditAction 
 
int  m_nCurrentEditAction  
 
 current action in undo stack
 
 
 
 
 
 
m_nSavedPointer 
 
int  m_nSavedPointer  
 
 action that the network is saved
 
 
 
 
 
 
m_bNeedsUndoAction 
 
boolean  m_bNeedsUndoAction  
 
 flag to indicate whether an edit action needs to introduce an undo
 action. This is only false when an undo or redo action is performed.
 
 
 
 

 
   
 
 
  
 Class  weka.classifiers.bayes.net.GUI  extends javax.swing.JPanel implements Serializable   
 
 

 
 serialVersionUID:  -2038911085935515624L

 
   
 
 
  
 Serialized Fields   
 
 

 
m_layoutEngine 
 
 LayoutEngine   m_layoutEngine  
 
 The current LayoutEngine
 
 
 
 
 
 
m_GraphPanel 
 
weka.classifiers.bayes.net.GUI.GraphPanel  m_GraphPanel  
 
 Panel actually displaying the graph
 
 
 
 
 
 
m_BayesNet 
 
 EditableBayesNet   m_BayesNet  
 
 Container of Bayesian network
 
 
 
 
 
 
m_sFileName 
 
java.lang.String  m_sFileName  
 
 String containing file name storing current network
 
 
 
 
 
 
m_marginCalculator 
 
 MarginCalculator   m_marginCalculator  
 
 used for calculating marginals in Bayesian netwowrks
 
 
 
 
 
 
m_marginCalculatorWithEvidence 
 
 MarginCalculator   m_marginCalculatorWithEvidence  
 
 used for calculating marginals in Bayesian netwowrks when evidence is
 present
 
 
 
 
 
 
m_bViewMargins 
 
boolean  m_bViewMargins  
 
 flag indicating whether marginal distributions of each of the nodes 
 should be shown in display.
 
 
 
 
 
 
m_bViewCliques 
 
boolean  m_bViewCliques  
 
 
 
 
 
 
m_menuBar 
 
javax.swing.JMenuBar  m_menuBar  
 
 The menu bar
 
 
 
 
 
 
m_Instances 
 
 Instances   m_Instances  
 
 data selected from file. Used to train a Bayesian network on
 
 
 
 
 
 
m_jTfZoom 
 
javax.swing.JTextField  m_jTfZoom  
 
 Text field for specifying zoom
 
 
 
 
 
 
m_jTbTools 
 
javax.swing.JToolBar  m_jTbTools  
 
 toolbar containing buttons at top of window
 
 
 
 
 
 
m_jStatusBar 
 
javax.swing.JLabel  m_jStatusBar  
 
 status bar at bottom of window
 
 
 
 
 
 
m_jTfNodeWidth 
 
javax.swing.JTextField  m_jTfNodeWidth  
 
 TextField for node's width
 
 
 
 
 
 
m_jTfNodeHeight 
 
javax.swing.JTextField  m_jTfNodeHeight  
 
 TextField for nodes height
 
 
 
 
 
 
m_jScrollPane 
 
javax.swing.JScrollPane  m_jScrollPane  
 
 this contains the m_GraphPanel GraphPanel
 
 
 
 
 
 
ICONPATH 
 
java.lang.String  ICONPATH  
 
 path for icons
 
 
 
 
 
 
m_fScale 
 
double  m_fScale  
 
 current zoom value
 
 
 
 
 
 
m_nNodeHeight 
 
int  m_nNodeHeight  
 
 standard width of node
 
 
 
 
 
 
m_nNodeWidth 
 
int  m_nNodeWidth  
 
 
 
 
 
 
m_nPaddedNodeWidth 
 
int  m_nPaddedNodeWidth  
 
 
 
 
 
 
m_nZoomPercents 
 
int[]  m_nZoomPercents  
 
 used when using zoomIn and zoomOut buttons
 
 
 
 
 
 
a_new 
 
javax.swing.Action  a_new  
 
 actions triggered by GUI events
 
 
 
 
 
 
a_quit 
 
javax.swing.Action  a_quit  
 
 
 
 
 
 
a_save 
 
javax.swing.Action  a_save  
 
 
 
 
 
 
a_export 
 
weka.classifiers.bayes.net.GUI.ActionExport  a_export  
 
 
 
 
 
 
a_print 
 
weka.classifiers.bayes.net.GUI.ActionPrint  a_print  
 
 
 
 
 
 
a_load 
 
javax.swing.Action  a_load  
 
 
 
 
 
 
a_zoomin 
 
javax.swing.Action  a_zoomin  
 
 
 
 
 
 
a_zoomout 
 
javax.swing.Action  a_zoomout  
 
 
 
 
 
 
a_layout 
 
javax.swing.Action  a_layout  
 
 
 
 
 
 
a_saveas 
 
javax.swing.Action  a_saveas  
 
 
 
 
 
 
a_viewtoolbar 
 
javax.swing.Action  a_viewtoolbar  
 
 
 
 
 
 
a_viewstatusbar 
 
javax.swing.Action  a_viewstatusbar  
 
 
 
 
 
 
a_networkgenerator 
 
javax.swing.Action  a_networkgenerator  
 
 
 
 
 
 
a_datagenerator 
 
javax.swing.Action  a_datagenerator  
 
 
 
 
 
 
a_datasetter 
 
javax.swing.Action  a_datasetter  
 
 
 
 
 
 
a_learn 
 
javax.swing.Action  a_learn  
 
 
 
 
 
 
a_learnCPT 
 
javax.swing.Action  a_learnCPT  
 
 
 
 
 
 
a_help 
 
javax.swing.Action  a_help  
 
 
 
 
 
 
a_about 
 
javax.swing.Action  a_about  
 
 
 
 
 
 
a_addnode 
 
weka.classifiers.bayes.net.GUI.ActionAddNode  a_addnode  
 
 
 
 
 
 
a_delnode 
 
javax.swing.Action  a_delnode  
 
 
 
 
 
 
a_cutnode 
 
javax.swing.Action  a_cutnode  
 
 
 
 
 
 
a_copynode 
 
javax.swing.Action  a_copynode  
 
 
 
 
 
 
a_pastenode 
 
javax.swing.Action  a_pastenode  
 
 
 
 
 
 
a_selectall 
 
javax.swing.Action  a_selectall  
 
 
 
 
 
 
a_addarc 
 
javax.swing.Action  a_addarc  
 
 
 
 
 
 
a_delarc 
 
javax.swing.Action  a_delarc  
 
 
 
 
 
 
a_undo 
 
javax.swing.Action  a_undo  
 
 
 
 
 
 
a_redo 
 
javax.swing.Action  a_redo  
 
 
 
 
 
 
a_alignleft 
 
javax.swing.Action  a_alignleft  
 
 
 
 
 
 
a_alignright 
 
javax.swing.Action  a_alignright  
 
 
 
 
 
 
a_aligntop 
 
javax.swing.Action  a_aligntop  
 
 
 
 
 
 
a_alignbottom 
 
javax.swing.Action  a_alignbottom  
 
 
 
 
 
 
a_centerhorizontal 
 
javax.swing.Action  a_centerhorizontal  
 
 
 
 
 
 
a_centervertical 
 
javax.swing.Action  a_centervertical  
 
 
 
 
 
 
a_spacehorizontal 
 
javax.swing.Action  a_spacehorizontal  
 
 
 
 
 
 
a_spacevertical 
 
javax.swing.Action  a_spacevertical  
 
 
 
 
 
 
m_nCurrentNode 
 
int  m_nCurrentNode  
 
 node currently selected through right clicking
 
 
 
 
 
 
m_Selection 
 
weka.classifiers.bayes.net.GUI.Selection  m_Selection  
 
 selection of nodes
 
 
 
 
 
 
m_nSelectedRect 
 
java.awt.Rectangle  m_nSelectedRect  
 
 selection rectangle drawn through dragging with left mouse button
 
 
 
 
 
 
m_clipboard 
 
weka.classifiers.bayes.net.GUI.ClipBoard  m_clipboard  
 
 
 
 

 
   
 
 
  
 Class  weka.classifiers.bayes.net.MarginCalculator  extends java.lang.Object implements Serializable   
 
 

 
 serialVersionUID:  650278019241175534L

 
   
 
 
  
 Serialized Fields   
 
 

 
m_debug 
 
boolean  m_debug  
 
 
 
 
 
 
m_root 
 
 MarginCalculator.JunctionTreeNode   m_root  
 
 
 
 
 
 
jtNodes 
 
 MarginCalculator.JunctionTreeNode []  jtNodes  
 
 
 
 
 
 
m_Margins 
 
double[][]  m_Margins  
 
 
 
 

 
   
 
 
  
 Class  weka.classifiers.bayes.net.MarginCalculator.JunctionTreeNode  extends java.lang.Object implements Serializable   
 
 

 
 serialVersionUID:  650278019241175536L

 
   
 
 
  
 Serialized Fields   
 
 

 
m_bayesNet 
 
 BayesNet   m_bayesNet  
 
 reference Bayes net for information about variables like name, cardinality, etc.
 but not for relations between nodes
 
 
 
 
 
 
m_nNodes 
 
int[]  m_nNodes  
 
 nodes of the Bayes net in this junction node
 
 
 
 
 
 
m_nCardinality 
 
int  m_nCardinality  
 
 cardinality of the instances of variables in this junction node
 
 
 
 
 
 
m_fi 
 
double[]  m_fi  
 
 potentials for first network
 
 
 
 
 
 
m_P 
 
double[]  m_P  
 
 distribution over this junction node according to first Bayes network
 
 
 
 
 
 
m_MarginalP 
 
double[][]  m_MarginalP  
 
 
 
 
 
 
m_parentSeparator 
 
 MarginCalculator.JunctionTreeSeparator   m_parentSeparator  
 
 
 
 
 
 
m_children 
 
java.util.Vector<E>  m_children  
 
 
 
 

 
   
 
 
  
 Class  weka.classifiers.bayes.net.MarginCalculator.JunctionTreeSeparator  extends java.lang.Object implements Serializable   
 
 

 
 serialVersionUID:  6502780192411755343L

 
   
 
 
  
 Serialized Fields   
 
 

 
m_nNodes 
 
int[]  m_nNodes  
 
 
 
 
 
 
m_nCardinality 
 
int  m_nCardinality  
 
 
 
 
 
 
m_fiParent 
 
double[]  m_fiParent  
 
 
 
 
 
 
m_fiChild 
 
double[]  m_fiChild  
 
 
 
 
 
 
m_parentNode 
 
 MarginCalculator.JunctionTreeNode   m_parentNode  
 
 
 
 
 
 
m_childNode 
 
 MarginCalculator.JunctionTreeNode   m_childNode  
 
 
 
 
 
 
m_bayesNet 
 
 BayesNet   m_bayesNet  
 
 
 
 

 
   
 
 
  
 Class  weka.classifiers.bayes.net.ParentSet  extends java.lang.Object implements Serializable   
 
 

 
 serialVersionUID:  4155021284407181838L

 
   
 
 
  
 Serialized Fields   
 
 

 
m_nParents 
 
int[]  m_nParents  
 
 Holds indexes of parents
 
 
 
 
 
 
m_nNrOfParents 
 
int  m_nNrOfParents  
 
 Holds number of parents
 
 
 
 
 
 
m_nCardinalityOfParents 
 
int  m_nCardinalityOfParents  
 
 Holds cardinality  of parents (= number of instantiations the parents can take)
 
 
 
 

 
   
 
 
  
 Class  weka.classifiers.bayes.net.VaryNode  extends java.lang.Object implements Serializable   
 
 

 
 serialVersionUID:  -6196294370675872424L

 
   
 
 
  
 Serialized Fields   
 
 

 
m_iNode 
 
int  m_iNode  
 
 index of the node varied
 
 
 
 
 
 
m_nMCV 
 
int  m_nMCV  
 
 most common value
 
 
 
 
 
 
m_ADNodes 
 
 ADNode []  m_ADNodes  
 
 list of ADNode children
 
 
 
 
 

 
 
  
 Package   weka.classifiers.bayes.net.estimate   
 
 

 
   
 
 
  
 Class  weka.classifiers.bayes.net.estimate.BayesNetEstimator  extends java.lang.Object implements Serializable   
 
 

 
 serialVersionUID:  2184330197666253884L

 
   
 
 
  
 Serialized Fields   
 
 

 
m_fAlpha 
 
double  m_fAlpha  
 
 Holds prior on count
 
 
 
 

 
   
 
 
  
 Class  weka.classifiers.bayes.net.estimate.BMAEstimator  extends  SimpleEstimator  implements Serializable   
 
 

 
 serialVersionUID:  -1846028304233257309L

 
   
 
 
  
 Serialized Fields   
 
 

 
m_bUseK2Prior 
 
boolean  m_bUseK2Prior  
 
 whether to use K2 prior
 
 
 
 

 
   
 
 
  
 Class  weka.classifiers.bayes.net.estimate.DiscreteEstimatorBayes  extends  Estimator  implements Serializable   
 
 

 
 serialVersionUID:  4215400230843212684L

 
   
 
 
  
 Serialized Fields   
 
 

 
m_Counts 
 
double[]  m_Counts  
 
 Hold the counts
 
 
 
 
 
 
m_SumOfCounts 
 
double  m_SumOfCounts  
 
 Hold the sum of counts
 
 
 
 
 
 
m_nSymbols 
 
int  m_nSymbols  
 
 Holds number of symbols in distribution
 
 
 
 
 
 
m_fPrior 
 
double  m_fPrior  
 
 Holds the prior probability
 
 
 
 

 
   
 
 
  
 Class  weka.classifiers.bayes.net.estimate.DiscreteEstimatorFullBayes  extends  DiscreteEstimatorBayes  implements Serializable   
 
 

 
 serialVersionUID:  6774941981423312133L

 

 
   
 
 
  
 Class  weka.classifiers.bayes.net.estimate.MultiNomialBMAEstimator  extends  BayesNetEstimator  implements Serializable   
 
 

 
 serialVersionUID:  8330705772601586313L

 
   
 
 
  
 Serialized Fields   
 
 

 
m_bUseK2Prior 
 
boolean  m_bUseK2Prior  
 
 whether to use K2 prior
 
 
 
 

 
   
 
 
  
 Class  weka.classifiers.bayes.net.estimate.SimpleEstimator  extends  BayesNetEstimator  implements Serializable   
 
 

 
 serialVersionUID:  5874941612331806172L

 
 

 
 
  
 Package   weka.classifiers.bayes.net.search   
 
 

 
   
 
 
  
 Class  weka.classifiers.bayes.net.search.SearchAlgorithm  extends java.lang.Object implements Serializable   
 
 

 
 serialVersionUID:  6164792240778525312L

 
   
 
 
  
 Serialized Fields   
 
 

 
m_nMaxNrOfParents 
 
int  m_nMaxNrOfParents  
 
 Holds upper bound on number of parents
 
 
 
 
 
 
m_bInitAsNaiveBayes 
 
boolean  m_bInitAsNaiveBayes  
 
 determines whether initial structure is an empty graph or a Naive Bayes network
 
 
 
 
 
 
m_bMarkovBlanketClassifier 
 
boolean  m_bMarkovBlanketClassifier  
 
 Determines whether after structure is found a MarkovBlanketClassifier correction should be applied
 If this is true, m_bInitAsNaiveBayes is overridden and interpreted as false.
 
 
 
 
 

 
 
  
 Package   weka.classifiers.bayes.net.search.ci   
 
 

 
   
 
 
  
 Class  weka.classifiers.bayes.net.search.ci.CISearchAlgorithm  extends  LocalScoreSearchAlgorithm  implements Serializable   
 
 

 
 serialVersionUID:  3165802334119704560L

 
   
 
 
  
 Serialized Fields   
 
 

 
m_BayesNet 
 
 BayesNet   m_BayesNet  
 
 
 
 
 
 
m_instances 
 
 Instances   m_instances  
 
 
 
 

 
   
 
 
  
 Class  weka.classifiers.bayes.net.search.ci.ICSSearchAlgorithm  extends  CISearchAlgorithm  implements Serializable   
 
 

 
 serialVersionUID:  -2510985917284798576L

 
   
 
 
  
 Serialized Fields   
 
 

 
m_nMaxCardinality 
 
int  m_nMaxCardinality  
 
 maximum size of separating set
 
 
 
 
 

 
 
  
 Package   weka.classifiers.bayes.net.search.fixed   
 
 

 
   
 
 
  
 Class  weka.classifiers.bayes.net.search.fixed.FromFile  extends  SearchAlgorithm  implements Serializable   
 
 

 
 serialVersionUID:  7334358169507619525L

 
   
 
 
  
 Serialized Fields   
 
 

 
m_sBIFFile 
 
java.lang.String  m_sBIFFile  
 
 name of file to read structure from
 
 
 
 

 
   
 
 
  
 Class  weka.classifiers.bayes.net.search.fixed.NaiveBayes  extends  SearchAlgorithm  implements Serializable   
 
 

 
 serialVersionUID:  -4808572519709755811L

 
 

 
 
  
 Package   weka.classifiers.bayes.net.search.global   
 
 

 
   
 
 
  
 Class  weka.classifiers.bayes.net.search.global.GeneticSearch  extends  GlobalScoreSearchAlgorithm  implements Serializable   
 
 

 
 serialVersionUID:  4236165533882462203L

 
   
 
 
  
 Serialized Fields   
 
 

 
m_nRuns 
 
int  m_nRuns  
 
 number of runs
 
 
 
 
 
 
m_nPopulationSize 
 
int  m_nPopulationSize  
 
 size of population
 
 
 
 
 
 
m_nDescendantPopulationSize 
 
int  m_nDescendantPopulationSize  
 
 size of descendant population
 
 
 
 
 
 
m_bUseCrossOver 
 
boolean  m_bUseCrossOver  
 
 use cross-over?
 
 
 
 
 
 
m_bUseMutation 
 
boolean  m_bUseMutation  
 
 use mutation?
 
 
 
 
 
 
m_bUseTournamentSelection 
 
boolean  m_bUseTournamentSelection  
 
 use tournament selection or take best sub-population
 
 
 
 
 
 
m_nSeed 
 
int  m_nSeed  
 
 random number seed
 
 
 
 
 
 
m_random 
 
java.util.Random  m_random  
 
 random number generator
 
 
 
 

 
   
 
 
  
 Class  weka.classifiers.bayes.net.search.global.GlobalScoreSearchAlgorithm  extends  SearchAlgorithm  implements Serializable   
 
 

 
 serialVersionUID:  7341389867906199781L

 
   
 
 
  
 Serialized Fields   
 
 

 
m_BayesNet 
 
 BayesNet   m_BayesNet  
 
 points to Bayes network for which a structure is searched for
 
 
 
 
 
 
m_bUseProb 
 
boolean  m_bUseProb  
 
 toggle between scoring using accuracy = 0-1 loss (when false) or class probabilities (when true)
 
 
 
 
 
 
m_nNrOfFolds 
 
int  m_nNrOfFolds  
 
 number of folds for k-fold cross validation
 
 
 
 
 
 
m_nCVType 
 
int  m_nCVType  
 
 Holds the cross validation strategy used to measure quality of network
 
 
 
 

 
   
 
 
  
 Class  weka.classifiers.bayes.net.search.global.HillClimber  extends  GlobalScoreSearchAlgorithm  implements Serializable   
 
 

 
 serialVersionUID:  -3885042888195820149L

 
   
 
 
  
 Serialized Fields   
 
 

 
m_bUseArcReversal 
 
boolean  m_bUseArcReversal  
 
 use the arc reversal operator
 
 
 
 

 
   
 
 
  
 Class  weka.classifiers.bayes.net.search.global.K2  extends  GlobalScoreSearchAlgorithm  implements Serializable   
 
 

 
 serialVersionUID:  -6626871067466338256L

 
   
 
 
  
 Serialized Fields   
 
 

 
m_bRandomOrder 
 
boolean  m_bRandomOrder  
 
 Holds flag to indicate ordering should be random
 
 
 
 

 
   
 
 
  
 Class  weka.classifiers.bayes.net.search.global.RepeatedHillClimber  extends  HillClimber  implements Serializable   
 
 

 
 serialVersionUID:  -7359197180460703069L

 
   
 
 
  
 Serialized Fields   
 
 

 
m_nRuns 
 
int  m_nRuns  
 
 number of runs
 
 
 
 
 
 
m_nSeed 
 
int  m_nSeed  
 
 random number seed
 
 
 
 
 
 
m_random 
 
java.util.Random  m_random  
 
 random number generator
 
 
 
 

 
   
 
 
  
 Class  weka.classifiers.bayes.net.search.global.SimulatedAnnealing  extends  GlobalScoreSearchAlgorithm  implements Serializable   
 
 

 
 serialVersionUID:  -5482721887881010916L

 
   
 
 
  
 Serialized Fields   
 
 

 
m_fTStart 
 
double  m_fTStart  
 
 start temperature
 
 
 
 
 
 
m_fDelta 
 
double  m_fDelta  
 
 change in temperature at every run
 
 
 
 
 
 
m_nRuns 
 
int  m_nRuns  
 
 number of runs
 
 
 
 
 
 
m_bUseArcReversal 
 
boolean  m_bUseArcReversal  
 
 use the arc reversal operator
 
 
 
 
 
 
m_nSeed 
 
int  m_nSeed  
 
 random number seed
 
 
 
 
 
 
m_random 
 
java.util.Random  m_random  
 
 random number generator
 
 
 
 

 
   
 
 
  
 Class  weka.classifiers.bayes.net.search.global.TabuSearch  extends  HillClimber  implements Serializable   
 
 

 
 serialVersionUID:  1176705618756672292L

 
   
 
 
  
 Serialized Fields   
 
 

 
m_nRuns 
 
int  m_nRuns  
 
 number of runs
 
 
 
 
 
 
m_nTabuList 
 
int  m_nTabuList  
 
 size of tabu list
 
 
 
 
 
 
m_oTabuList 
 
weka.classifiers.bayes.net.search.global.HillClimber.Operation[]  m_oTabuList  
 
 the actual tabu list
 
 
 
 

 
   
 
 
  
 Class  weka.classifiers.bayes.net.search.global.TAN  extends  GlobalScoreSearchAlgorithm  implements Serializable   
 
 

 
 serialVersionUID:  1715277053980895298L

 
 

 
 
  
 Package   weka.classifiers.bayes.net.search.local   
 
 

 
   
 
 
  
 Class  weka.classifiers.bayes.net.search.local.GeneticSearch  extends  LocalScoreSearchAlgorithm  implements Serializable   
 
 

 
 serialVersionUID:  -7037070678911459757L

 
   
 
 
  
 Serialized Fields   
 
 

 
m_nRuns 
 
int  m_nRuns  
 
 number of runs
 
 
 
 
 
 
m_nPopulationSize 
 
int  m_nPopulationSize  
 
 size of population
 
 
 
 
 
 
m_nDescendantPopulationSize 
 
int  m_nDescendantPopulationSize  
 
 size of descendant population
 
 
 
 
 
 
m_bUseCrossOver 
 
boolean  m_bUseCrossOver  
 
 use cross-over?
 
 
 
 
 
 
m_bUseMutation 
 
boolean  m_bUseMutation  
 
 use mutation?
 
 
 
 
 
 
m_bUseTournamentSelection 
 
boolean  m_bUseTournamentSelection  
 
 use tournament selection or take best sub-population
 
 
 
 
 
 
m_nSeed 
 
int  m_nSeed  
 
 random number seed
 
 
 
 
 
 
m_random 
 
java.util.Random  m_random  
 
 random number generator
 
 
 
 

 
   
 
 
  
 Class  weka.classifiers.bayes.net.search.local.HillClimber  extends  LocalScoreSearchAlgorithm  implements Serializable   
 
 

 
 serialVersionUID:  4322783593818122403L

 
   
 
 
  
 Serialized Fields   
 
 

 
m_Cache 
 
weka.classifiers.bayes.net.search.local.HillClimber.Cache  m_Cache  
 
 cache for storing score differences
 
 
 
 
 
 
m_bUseArcReversal 
 
boolean  m_bUseArcReversal  
 
 use the arc reversal operator
 
 
 
 

 
   
 
 
  
 Class  weka.classifiers.bayes.net.search.local.K2  extends  LocalScoreSearchAlgorithm  implements Serializable   
 
 

 
 serialVersionUID:  6176545934752116631L

 
   
 
 
  
 Serialized Fields   
 
 

 
m_bRandomOrder 
 
boolean  m_bRandomOrder  
 
 Holds flag to indicate ordering should be random
 
 
 
 

 
   
 
 
  
 Class  weka.classifiers.bayes.net.search.local.LAGDHillClimber  extends  HillClimber  implements Serializable   
 
 

 
 serialVersionUID:  7217437499439184344L

 
   
 
 
  
 Serialized Fields   
 
 

 
m_nNrOfLookAheadSteps 
 
int  m_nNrOfLookAheadSteps  
 
 Number of Look Ahead Steps
 
 
 
 
 
 
m_nNrOfGoodOperations 
 
int  m_nNrOfGoodOperations  
 
 Number of Good Operations per Step
 
 
 
 

 
   
 
 
  
 Class  weka.classifiers.bayes.net.search.local.LocalScoreSearchAlgorithm  extends  SearchAlgorithm  implements Serializable   
 
 

 
 serialVersionUID:  3325995552474190374L

 
   
 
 
  
 Serialized Fields   
 
 

 
m_BayesNet 
 
 BayesNet   m_BayesNet  
 
 points to Bayes network for which a structure is searched for
 
 
 
 
 
 
m_fAlpha 
 
double  m_fAlpha  
 
 Holds prior on count
 
 
 
 
 
 
m_nScoreType 
 
int  m_nScoreType  
 
 Holds the score type used to measure quality of network
 
 
 
 

 
   
 
 
  
 Class  weka.classifiers.bayes.net.search.local.RepeatedHillClimber  extends  HillClimber  implements Serializable   
 
 

 
 serialVersionUID:  -6574084564213041174L

 
   
 
 
  
 Serialized Fields   
 
 

 
m_nRuns 
 
int  m_nRuns  
 
 number of runs
 
 
 
 
 
 
m_nSeed 
 
int  m_nSeed  
 
 random number seed
 
 
 
 
 
 
m_random 
 
java.util.Random  m_random  
 
 random number generator
 
 
 
 

 
   
 
 
  
 Class  weka.classifiers.bayes.net.search.local.SimulatedAnnealing  extends  LocalScoreSearchAlgorithm  implements Serializable   
 
 

 
 serialVersionUID:  6951955606060513191L

 
   
 
 
  
 Serialized Fields   
 
 

 
m_fTStart 
 
double  m_fTStart  
 
 start temperature
 
 
 
 
 
 
m_fDelta 
 
double  m_fDelta  
 
 change in temperature at every run
 
 
 
 
 
 
m_nRuns 
 
int  m_nRuns  
 
 number of runs
 
 
 
 
 
 
m_bUseArcReversal 
 
boolean  m_bUseArcReversal  
 
 use the arc reversal operator
 
 
 
 
 
 
m_nSeed 
 
int  m_nSeed  
 
 random number seed
 
 
 
 
 
 
m_random 
 
java.util.Random  m_random  
 
 random number generator
 
 
 
 

 
   
 
 
  
 Class  weka.classifiers.bayes.net.search.local.TabuSearch  extends  HillClimber  implements Serializable   
 
 

 
 serialVersionUID:  1457344073228786447L

 
   
 
 
  
 Serialized Fields   
 
 

 
m_nRuns 
 
int  m_nRuns  
 
 number of runs
 
 
 
 
 
 
m_nTabuList 
 
int  m_nTabuList  
 
 size of tabu list
 
 
 
 
 
 
m_oTabuList 
 
weka.classifiers.bayes.net.search.local.HillClimber.Operation[]  m_oTabuList  
 
 the actual tabu list
 
 
 
 

 
   
 
 
  
 Class  weka.classifiers.bayes.net.search.local.TAN  extends  LocalScoreSearchAlgorithm  implements Serializable   
 
 

 
 serialVersionUID:  965182127977228690L

 
 

 
 
  
 Package   weka.classifiers.evaluation   
 
 

 
   
 
 
  
 Class  weka.classifiers.evaluation.ConfusionMatrix  extends  Matrix  implements Serializable   
 
 

 
 serialVersionUID:  -181789981401504090L

 
   
 
 
  
 Serialized Fields   
 
 

 
m_ClassNames 
 
java.lang.String[]  m_ClassNames  
 
 Stores the names of the classes
 
 
 
 

 
   
 
 
  
 Class  weka.classifiers.evaluation.NominalPrediction  extends java.lang.Object implements Serializable   
 
 

 
 serialVersionUID:  -8871333992740492788L

 
   
 
 
  
 Serialized Fields   
 
 

 
m_Distribution 
 
double[]  m_Distribution  
 
 The predicted probabilities
 
 
 
 
 
 
m_Actual 
 
double  m_Actual  
 
 The actual class value
 
 
 
 
 
 
m_Predicted 
 
double  m_Predicted  
 
 The predicted class value
 
 
 
 
 
 
m_Weight 
 
double  m_Weight  
 
 The weight assigned to this prediction
 
 
 
 

 
   
 
 
  
 Class  weka.classifiers.evaluation.NumericPrediction  extends java.lang.Object implements Serializable   
 
 

 
 serialVersionUID:  -4880216423674233887L

 
   
 
 
  
 Serialized Fields   
 
 

 
m_Actual 
 
double  m_Actual  
 
 The actual class value
 
 
 
 
 
 
m_Predicted 
 
double  m_Predicted  
 
 The predicted class value
 
 
 
 
 
 
m_Weight 
 
double  m_Weight  
 
 The weight assigned to this prediction
 
 
 
 
 

 
 
  
 Package   weka.classifiers.functions   
 
 

 
   
 
 
  
 Class  weka.classifiers.functions.GaussianProcesses  extends  Classifier  implements Serializable   
 
 

 
 serialVersionUID:  -8620066949967678545L

 
   
 
 
  
 Serialized Fields   
 
 

 
m_NominalToBinary 
 
 NominalToBinary   m_NominalToBinary  
 
 The filter used to make attributes numeric.
 
 
 
 
 
 
m_Filter 
 
 Filter   m_Filter  
 
 The filter used to standardize/normalize all values.
 
 
 
 
 
 
m_filterType 
 
int  m_filterType  
 
 Whether to normalize/standardize/neither
 
 
 
 
 
 
m_Missing 
 
 ReplaceMissingValues   m_Missing  
 
 The filter used to get rid of missing values.
 
 
 
 
 
 
m_checksTurnedOff 
 
boolean  m_checksTurnedOff  
 
 Turn off all checks and conversions? Turning them off assumes
      that data is purely numeric, doesn't contain any missing values,
      and has a numeric class.
 
 
 
 
 
 
m_delta 
 
double  m_delta  
 
 Gaussian Noise Value.
 
 
 
 
 
 
m_classIndex 
 
int  m_classIndex  
 
 The class index from the training data
 
 
 
 
 
 
m_Alin 
 
double  m_Alin  
 
 The parameters of the linear transforamtion realized 
 by the filter on the class attribute
 
 
 
 
 
 
m_Blin 
 
double  m_Blin  
 
 
 
 
 
 
m_kernel 
 
 Kernel   m_kernel  
 
 Kernel to use
 
 
 
 
 
 
m_NumTrain 
 
int  m_NumTrain  
 
 The number of training instances
 
 
 
 
 
 
m_avg_target 
 
double  m_avg_target  
 
 The training data.
 
 
 
 
 
 
m_C 
 
 Matrix   m_C  
 
 The covariance matrix.
 
 
 
 
 
 
m_t 
 
 Matrix   m_t  
 
 The vector of target values.
 
 
 
 
 
 
m_KernelIsLinear 
 
boolean  m_KernelIsLinear  
 
 whether the kernel is a linear one
 
 
 
 

 
   
 
 
  
 Class  weka.classifiers.functions.IsotonicRegression  extends  Classifier  implements Serializable   
 
 

 
 serialVersionUID:  1679336022835454137L

 
   
 
 
  
 Serialized Fields   
 
 

 
m_attribute 
 
 Attribute   m_attribute  
 
 The chosen attribute
 
 
 
 
 
 
m_cuts 
 
double[]  m_cuts  
 
 The array of cut points
 
 
 
 
 
 
m_values 
 
double[]  m_values  
 
 The predicted value in each interval.
 
 
 
 
 
 
m_minMsq 
 
double  m_minMsq  
 
 The minimum mean squared error that has been achieved.
 
 
 
 
 
 
m_ZeroR 
 
 Classifier   m_ZeroR  
 
 a ZeroR model in case no model can be built from the data
 
 
 
 

 
   
 
 
  
 Class  weka.classifiers.functions.LeastMedSq  extends  Classifier  implements Serializable   
 
 

 
 serialVersionUID:  4288954049987652970L

 
   
 
 
  
 Serialized Fields   
 
 

 
m_Residuals 
 
double[]  m_Residuals  
 
 
 
 
 
 
m_weight 
 
double[]  m_weight  
 
 
 
 
 
 
m_SSR 
 
double  m_SSR  
 
 
 
 
 
 
m_scalefactor 
 
double  m_scalefactor  
 
 
 
 
 
 
m_bestMedian 
 
double  m_bestMedian  
 
 
 
 
 
 
m_currentRegression 
 
 LinearRegression   m_currentRegression  
 
 
 
 
 
 
m_bestRegression 
 
 LinearRegression   m_bestRegression  
 
 
 
 
 
 
m_ls 
 
 LinearRegression   m_ls  
 
 
 
 
 
 
m_Data 
 
 Instances   m_Data  
 
 
 
 
 
 
m_RLSData 
 
 Instances   m_RLSData  
 
 
 
 
 
 
m_SubSample 
 
 Instances   m_SubSample  
 
 
 
 
 
 
m_MissingFilter 
 
 ReplaceMissingValues   m_MissingFilter  
 
 
 
 
 
 
m_TransformFilter 
 
 NominalToBinary   m_TransformFilter  
 
 
 
 
 
 
m_SplitFilter 
 
 RemoveRange   m_SplitFilter  
 
 
 
 
 
 
m_samplesize 
 
int  m_samplesize  
 
 
 
 
 
 
m_samples 
 
int  m_samples  
 
 
 
 
 
 
m_israndom 
 
boolean  m_israndom  
 
 
 
 
 
 
m_debug 
 
boolean  m_debug  
 
 
 
 
 
 
m_random 
 
java.util.Random  m_random  
 
 
 
 
 
 
m_randomseed 
 
long  m_randomseed  
 
 
 
 

 
   
 
 
  
 Class  weka.classifiers.functions.LibLINEAR  extends  Classifier  implements Serializable   
 
 

 
 serialVersionUID:  230504711L

 
   
 
 
  
 Serialized Fields   
 
 

 
m_Model 
 
java.lang.Object  m_Model  
 
 LibLINEAR Model
 
 
 
 
 
 
m_Filter 
 
 Filter   m_Filter  
 
 for normalizing the data
 
 
 
 
 
 
m_Normalize 
 
boolean  m_Normalize  
 
 normalize input data
 
 
 
 
 
 
m_SVMType 
 
int  m_SVMType  
 
 the SVM solver type
 
 
 
 
 
 
m_eps 
 
double  m_eps  
 
 stopping criteria
 
 
 
 
 
 
m_Cost 
 
double  m_Cost  
 
 cost Parameter C
 
 
 
 
 
 
m_Bias 
 
double  m_Bias  
 
 bias term value
 
 
 
 
 
 
m_WeightLabel 
 
int[]  m_WeightLabel  
 
 
 
 
 
 
m_Weight 
 
double[]  m_Weight  
 
 
 
 
 
 
m_ProbabilityEstimates 
 
boolean  m_ProbabilityEstimates  
 
 whether to generate probability estimates instead of +1/-1 in case of
 classification problems
 
 
 
 
 
 
m_ReplaceMissingValues 
 
 ReplaceMissingValues   m_ReplaceMissingValues  
 
 The filter used to get rid of missing values.
 
 
 
 
 
 
m_NominalToBinary 
 
 NominalToBinary   m_NominalToBinary  
 
 The filter used to make attributes numeric.
 
 
 
 
 
 
m_nominalToBinary 
 
boolean  m_nominalToBinary  
 
 If true, the nominal to binary filter is applied
 
 
 
 
 
 
m_noReplaceMissingValues 
 
boolean  m_noReplaceMissingValues  
 
 If true, the replace missing values filter is not applied
 
 
 
 

 
   
 
 
  
 Class  weka.classifiers.functions.LibSVM  extends  Classifier  implements Serializable   
 
 

 
 serialVersionUID:  14172L

 
   
 
 
  
 Serialized Fields   
 
 

 
m_Model 
 
java.lang.Object  m_Model  
 
 LibSVM Model
 
 
 
 
 
 
m_Filter 
 
 Filter   m_Filter  
 
 for normalizing the data
 
 
 
 
 
 
m_ReplaceMissingValues 
 
 ReplaceMissingValues   m_ReplaceMissingValues  
 
 The filter used to get rid of missing values.
 
 
 
 
 
 
m_Normalize 
 
boolean  m_Normalize  
 
 normalize input data
 
 
 
 
 
 
m_noReplaceMissingValues 
 
boolean  m_noReplaceMissingValues  
 
 If true, the replace missing values filter is not applied
 
 
 
 
 
 
m_SVMType 
 
int  m_SVMType  
 
 the SVM type
 
 
 
 
 
 
m_KernelType 
 
int  m_KernelType  
 
 the kernel type
 
 
 
 
 
 
m_Degree 
 
int  m_Degree  
 
 for poly - in older versions of libsvm declared as a double.
 At least since 2.82 it is an int.
 
 
 
 
 
 
m_Gamma 
 
double  m_Gamma  
 
 for poly/rbf/sigmoid
 
 
 
 
 
 
m_GammaActual 
 
double  m_GammaActual  
 
 for poly/rbf/sigmoid (the actual gamma)
 
 
 
 
 
 
m_Coef0 
 
double  m_Coef0  
 
 for poly/sigmoid
 
 
 
 
 
 
m_CacheSize 
 
double  m_CacheSize  
 
 in MB
 
 
 
 
 
 
m_eps 
 
double  m_eps  
 
 stopping criteria
 
 
 
 
 
 
m_Cost 
 
double  m_Cost  
 
 cost, for C_SVC, EPSILON_SVR and NU_SVR
 
 
 
 
 
 
m_WeightLabel 
 
int[]  m_WeightLabel  
 
 for C_SVC
 
 
 
 
 
 
m_Weight 
 
double[]  m_Weight  
 
 for C_SVC
 
 
 
 
 
 
m_nu 
 
double  m_nu  
 
 for NU_SVC, ONE_CLASS, and NU_SVR
 
 
 
 
 
 
m_Loss 
 
double  m_Loss  
 
 loss, for EPSILON_SVR
 
 
 
 
 
 
m_Shrinking 
 
boolean  m_Shrinking  
 
 use the shrinking heuristics
 
 
 
 
 
 
m_ProbabilityEstimates 
 
boolean  m_ProbabilityEstimates  
 
 whether to generate probability estimates instead of +1/-1 in case of 
 classification problems
 
 
 
 

 
   
 
 
  
 Class  weka.classifiers.functions.LinearRegression  extends  Classifier  implements Serializable   
 
 

 
 serialVersionUID:  -3364580862046573747L

 
   
 
 
  
 Serialized Fields   
 
 

 
m_Coefficients 
 
double[]  m_Coefficients  
 
 Array for storing coefficients of linear regression.
 
 
 
 
 
 
m_SelectedAttributes 
 
boolean[]  m_SelectedAttributes  
 
 Which attributes are relevant?
 
 
 
 
 
 
m_TransformedData 
 
 Instances   m_TransformedData  
 
 Variable for storing transformed training data.
 
 
 
 
 
 
m_MissingFilter 
 
 ReplaceMissingValues   m_MissingFilter  
 
 The filter for removing missing values.
 
 
 
 
 
 
m_TransformFilter 
 
 NominalToBinary   m_TransformFilter  
 
 The filter storing the transformation from nominal to 
      binary attributes.
 
 
 
 
 
 
m_ClassStdDev 
 
double  m_ClassStdDev  
 
 The standard deviations of the class attribute
 
 
 
 
 
 
m_ClassMean 
 
double  m_ClassMean  
 
 The mean of the class attribute
 
 
 
 
 
 
m_ClassIndex 
 
int  m_ClassIndex  
 
 The index of the class attribute
 
 
 
 
 
 
m_Means 
 
double[]  m_Means  
 
 The attributes means
 
 
 
 
 
 
m_StdDevs 
 
double[]  m_StdDevs  
 
 The attribute standard deviations
 
 
 
 
 
 
b_Debug 
 
boolean  b_Debug  
 
 True if debug output will be printed
 
 
 
 
 
 
m_AttributeSelection 
 
int  m_AttributeSelection  
 
 The current attribute selection method
 
 
 
 
 
 
m_EliminateColinearAttributes 
 
boolean  m_EliminateColinearAttributes  
 
 Try to eliminate correlated attributes?
 
 
 
 
 
 
m_checksTurnedOff 
 
boolean  m_checksTurnedOff  
 
 Turn off all checks and conversions?
 
 
 
 
 
 
m_Ridge 
 
double  m_Ridge  
 
 The ridge parameter
 
 
 
 

 
   
 
 
  
 Class  weka.classifiers.functions.Logistic  extends  Classifier  implements Serializable   
 
 

 
 serialVersionUID:  3932117032546553727L

 
   
 
 
  
 Serialized Fields   
 
 

 
m_Par 
 
double[][]  m_Par  
 
 The coefficients (optimized parameters) of the model
 
 
 
 
 
 
m_Data 
 
double[][]  m_Data  
 
 The data saved as a matrix
 
 
 
 
 
 
m_NumPredictors 
 
int  m_NumPredictors  
 
 The number of attributes in the model
 
 
 
 
 
 
m_ClassIndex 
 
int  m_ClassIndex  
 
 The index of the class attribute
 
 
 
 
 
 
m_NumClasses 
 
int  m_NumClasses  
 
 The number of the class labels
 
 
 
 
 
 
m_Ridge 
 
double  m_Ridge  
 
 The ridge parameter.
 
 
 
 
 
 
m_AttFilter 
 
 RemoveUseless   m_AttFilter  
 
 An attribute filter
 
 
 
 
 
 
m_NominalToBinary 
 
 NominalToBinary   m_NominalToBinary  
 
 The filter used to make attributes numeric.
 
 
 
 
 
 
m_ReplaceMissingValues 
 
 ReplaceMissingValues   m_ReplaceMissingValues  
 
 The filter used to get rid of missing values.
 
 
 
 
 
 
m_Debug 
 
boolean  m_Debug  
 
 Debugging output
 
 
 
 
 
 
m_LL 
 
double  m_LL  
 
 Log-likelihood of the searched model
 
 
 
 
 
 
m_MaxIts 
 
int  m_MaxIts  
 
 The maximum number of iterations.
 
 
 
 
 
 
m_structure 
 
 Instances   m_structure  
 
 
 
 

 
   
 
 
  
 Class  weka.classifiers.functions.MultilayerPerceptron  extends  Classifier  implements Serializable   
 
 

 
 serialVersionUID:  -5990607817048210779L

 
   
 
 
  
 Serialized Fields   
 
 

 
m_ZeroR 
 
 Classifier   m_ZeroR  
 
 a ZeroR model in case no model can be built from the data 
 or the network predicts all zeros for the classes
 
 
 
 
 
 
m_useDefaultModel 
 
boolean  m_useDefaultModel  
 
 Whether to use the default ZeroR model
 
 
 
 
 
 
m_instances 
 
 Instances   m_instances  
 
 The training instances.
 
 
 
 
 
 
m_currentInstance 
 
 Instance   m_currentInstance  
 
 The current instance running through the network.
 
 
 
 
 
 
m_numeric 
 
boolean  m_numeric  
 
 A flag to say that it's a numeric class.
 
 
 
 
 
 
m_attributeRanges 
 
double[]  m_attributeRanges  
 
 The ranges for all the attributes.
 
 
 
 
 
 
m_attributeBases 
 
double[]  m_attributeBases  
 
 The base values for all the attributes.
 
 
 
 
 
 
m_outputs 
 
weka.classifiers.functions.MultilayerPerceptron.NeuralEnd[]  m_outputs  
 
 The output units.(only feeds the errors, does no calcs)
 
 
 
 
 
 
m_inputs 
 
weka.classifiers.functions.MultilayerPerceptron.NeuralEnd[]  m_inputs  
 
 The input units.(only feeds the inputs does no calcs)
 
 
 
 
 
 
m_neuralNodes 
 
 NeuralConnection []  m_neuralNodes  
 
 All the nodes that actually comprise the logical neural net.
 
 
 
 
 
 
m_numClasses 
 
int  m_numClasses  
 
 The number of classes.
 
 
 
 
 
 
m_numAttributes 
 
int  m_numAttributes  
 
 The number of attributes.
 
 
 
 
 
 
m_nodePanel 
 
weka.classifiers.functions.MultilayerPerceptron.NodePanel  m_nodePanel  
 
 The panel the nodes are displayed on.
 
 
 
 
 
 
m_controlPanel 
 
weka.classifiers.functions.MultilayerPerceptron.ControlPanel  m_controlPanel  
 
 The control panel.
 
 
 
 
 
 
m_nextId 
 
int  m_nextId  
 
 The next id number available for default naming.
 
 
 
 
 
 
m_selected 
 
 FastVector   m_selected  
 
 A Vector list of the units currently selected.
 
 
 
 
 
 
m_graphers 
 
 FastVector   m_graphers  
 
 A Vector list of the graphers.
 
 
 
 
 
 
m_numEpochs 
 
int  m_numEpochs  
 
 The number of epochs to train through.
 
 
 
 
 
 
m_stopIt 
 
boolean  m_stopIt  
 
 a flag to state if the network should be running, or stopped.
 
 
 
 
 
 
m_stopped 
 
boolean  m_stopped  
 
 a flag to state that the network has in fact stopped.
 
 
 
 
 
 
m_accepted 
 
boolean  m_accepted  
 
 a flag to state that the network should be accepted the way it is.
 
 
 
 
 
 
m_win 
 
javax.swing.JFrame  m_win  
 
 The window for the network.
 
 
 
 
 
 
m_autoBuild 
 
boolean  m_autoBuild  
 
 A flag to tell the build classifier to automatically build a neural net.
 
 
 
 
 
 
m_gui 
 
boolean  m_gui  
 
 A flag to state that the gui for the network should be brought up.
      To allow interaction while training.
 
 
 
 
 
 
m_valSize 
 
int  m_valSize  
 
 An int to say how big the validation set should be.
 
 
 
 
 
 
m_driftThreshold 
 
int  m_driftThreshold  
 
 The number to to use to quit on validation testing.
 
 
 
 
 
 
m_randomSeed 
 
int  m_randomSeed  
 
 The number used to seed the random number generator.
 
 
 
 
 
 
m_random 
 
java.util.Random  m_random  
 
 The actual random number generator.
 
 
 
 
 
 
m_useNomToBin 
 
boolean  m_useNomToBin  
 
 A flag to state that a nominal to binary filter should be used.
 
 
 
 
 
 
m_nominalToBinaryFilter 
 
 NominalToBinary   m_nominalToBinaryFilter  
 
 The actual filter.
 
 
 
 
 
 
m_hiddenLayers 
 
java.lang.String  m_hiddenLayers  
 
 The string that defines the hidden layers
 
 
 
 
 
 
m_normalizeAttributes 
 
boolean  m_normalizeAttributes  
 
 This flag states that the user wants the input values normalized.
 
 
 
 
 
 
m_decay 
 
boolean  m_decay  
 
 This flag states that the user wants the learning rate to decay.
 
 
 
 
 
 
m_learningRate 
 
double  m_learningRate  
 
 This is the learning rate for the network.
 
 
 
 
 
 
m_momentum 
 
double  m_momentum  
 
 This is the momentum for the network.
 
 
 
 
 
 
m_epoch 
 
int  m_epoch  
 
 Shows the number of the epoch that the network just finished.
 
 
 
 
 
 
m_error 
 
double  m_error  
 
 Shows the error of the epoch that the network just finished.
 
 
 
 
 
 
m_reset 
 
boolean  m_reset  
 
 This flag states that the user wants the network to restart if it
 is found to be generating infinity or NaN for the error value. This
 would restart the network with the current options except that the
 learning rate would be smaller than before, (perhaps half of its current
 value). This option will not be available if the gui is chosen (if the
 gui is open the user can fix the network themselves, it is an 
 architectural minefield for the network to be reset with the gui open).
 
 
 
 
 
 
m_normalizeClass 
 
boolean  m_normalizeClass  
 
 This flag states that the user wants the class to be normalized while
 processing in the network is done. (the final answer will be in the
 original range regardless). This option will only be used when the class
 is numeric.
 
 
 
 
 
 
m_sigmoidUnit 
 
 SigmoidUnit   m_sigmoidUnit  
 
 this is a sigmoid unit.
 
 
 
 
 
 
m_linearUnit 
 
 LinearUnit   m_linearUnit  
 
 This is a linear unit.
 
 
 
 

 
   
 
 
  
 Class weka.classifiers.functions.MultilayerPerceptron.NeuralEnd extends  NeuralConnection  implements Serializable   
 
 

 
 serialVersionUID:  7305185603191183338L

 
   
 
 
  
 Serialized Fields   
 
 

 
m_link 
 
int  m_link  
 
 the value that represents the instance value this node represents. 
 For an input it is the attribute number, for an output, if nominal
 it is the class value.
 
 
 
 
 
 
m_input 
 
boolean  m_input  
 
 True if node is an input, False if it's an output.
 
 
 
 

 
   
 
 
  
 Class  weka.classifiers.functions.PaceRegression  extends  Classifier  implements Serializable   
 
 

 
 serialVersionUID:  7230266976059115435L

 
   
 
 
  
 Serialized Fields   
 
 

 
m_Model 
 
 Instances   m_Model  
 
 The model used
 
 
 
 
 
 
m_Coefficients 
 
double[]  m_Coefficients  
 
 Array for storing coefficients of linear regression.
 
 
 
 
 
 
m_ClassIndex 
 
int  m_ClassIndex  
 
 The index of the class attribute
 
 
 
 
 
 
m_Debug 
 
boolean  m_Debug  
 
 True if debug output will be printed
 
 
 
 
 
 
paceEstimator 
 
int  paceEstimator  
 
 the estimator
 
 
 
 
 
 
olscThreshold 
 
double  olscThreshold  
 
 
 
 

 
   
 
 
  
 Class  weka.classifiers.functions.PLSClassifier  extends  Classifier  implements Serializable   
 
 

 
 serialVersionUID:  4819775160590973256L

 
   
 
 
  
 Serialized Fields   
 
 

 
m_Filter 
 
 PLSFilter   m_Filter  
 
 the PLS filter
 
 
 
 
 
 
m_ActualFilter 
 
 PLSFilter   m_ActualFilter  
 
 the actual filter to use
 
 
 
 

 
   
 
 
  
 Class  weka.classifiers.functions.RBFNetwork  extends  Classifier  implements Serializable   
 
 

 
 serialVersionUID:  -3669814959712675720L

 
   
 
 
  
 Serialized Fields   
 
 

 
m_logistic 
 
 Logistic   m_logistic  
 
 The logistic regression for classification problems
 
 
 
 
 
 
m_linear 
 
 LinearRegression   m_linear  
 
 The linear regression for numeric problems
 
 
 
 
 
 
m_basisFilter 
 
 ClusterMembership   m_basisFilter  
 
 The filter for producing the meta data
 
 
 
 
 
 
m_standardize 
 
 Standardize   m_standardize  
 
 Filter used for normalizing the data
 
 
 
 
 
 
m_numClusters 
 
int  m_numClusters  
 
 The number of clusters (basis functions to generate)
 
 
 
 
 
 
m_ridge 
 
double  m_ridge  
 
 The ridge parameter for the logistic regression.
 
 
 
 
 
 
m_maxIts 
 
int  m_maxIts  
 
 The maximum number of iterations for logistic regression.
 
 
 
 
 
 
m_clusteringSeed 
 
int  m_clusteringSeed  
 
 The seed to pass on to K-means
 
 
 
 
 
 
m_minStdDev 
 
double  m_minStdDev  
 
 The minimum standard deviation
 
 
 
 
 
 
m_ZeroR 
 
 Classifier   m_ZeroR  
 
 a ZeroR model in case no model can be built from the data
 
 
 
 

 
   
 
 
  
 Class  weka.classifiers.functions.SimpleLinearRegression  extends  Classifier  implements Serializable   
 
 

 
 serialVersionUID:  1679336022895414137L

 
   
 
 
  
 Serialized Fields   
 
 

 
m_attribute 
 
 Attribute   m_attribute  
 
 The chosen attribute
 
 
 
 
 
 
m_attributeIndex 
 
int  m_attributeIndex  
 
 The index of the chosen attribute
 
 
 
 
 
 
m_slope 
 
double  m_slope  
 
 The slope
 
 
 
 
 
 
m_intercept 
 
double  m_intercept  
 
 The intercept
 
 
 
 
 
 
m_suppressErrorMessage 
 
boolean  m_suppressErrorMessage  
 
 If true, suppress error message if no useful attribute was found
 
 
 
 

 
   
 
 
  
 Class  weka.classifiers.functions.SimpleLogistic  extends  Classifier  implements Serializable   
 
 

 
 serialVersionUID:  7397710626304705059L

 
   
 
 
  
 Serialized Fields   
 
 

 
m_boostedModel 
 
 LogisticBase   m_boostedModel  
 
 The actual logistic regression model
 
 
 
 
 
 
m_NominalToBinary 
 
 NominalToBinary   m_NominalToBinary  
 
 Filter for converting nominal attributes to binary ones
 
 
 
 
 
 
m_ReplaceMissingValues 
 
 ReplaceMissingValues   m_ReplaceMissingValues  
 
 Filter for replacing missing values
 
 
 
 
 
 
m_numBoostingIterations 
 
int  m_numBoostingIterations  
 
 If non-negative, use this as fixed number of LogitBoost iterations
 
 
 
 
 
 
m_maxBoostingIterations 
 
int  m_maxBoostingIterations  
 
 Maximum number of iterations for LogitBoost
 
 
 
 
 
 
m_heuristicStop 
 
int  m_heuristicStop  
 
 Parameter for the heuristic for early stopping of LogitBoost
 
 
 
 
 
 
m_useCrossValidation 
 
boolean  m_useCrossValidation  
 
 If true, cross-validate number of LogitBoost iterations
 
 
 
 
 
 
m_errorOnProbabilities 
 
boolean  m_errorOnProbabilities  
 
 If true, use minimize error on probabilities instead of misclassification error
 
 
 
 
 
 
m_weightTrimBeta 
 
double  m_weightTrimBeta  
 
 Threshold for trimming weights. Instances with a weight lower than this (as a percentage
 of total weights) are not included in the regression fit.
 
 
 
 
 
 
m_useAIC 
 
boolean  m_useAIC  
 
 If true, the AIC is used to choose the best iteration
 
 
 
 

 
   
 
 
  
 Class  weka.classifiers.functions.SMO  extends  Classifier  implements Serializable   
 
 

 
 serialVersionUID:  -6585883636378691736L

 
   
 
 
  
 Serialized Fields   
 
 

 
m_classifiers 
 
 SMO.BinarySMO [][]  m_classifiers  
 
 The binary classifier(s)
 
 
 
 
 
 
m_C 
 
double  m_C  
 
 The complexity parameter.
 
 
 
 
 
 
m_eps 
 
double  m_eps  
 
 Epsilon for rounding.
 
 
 
 
 
 
m_tol 
 
double  m_tol  
 
 Tolerance for accuracy of result.
 
 
 
 
 
 
m_filterType 
 
int  m_filterType  
 
 Whether to normalize/standardize/neither
 
 
 
 
 
 
m_NominalToBinary 
 
 NominalToBinary   m_NominalToBinary  
 
 The filter used to make attributes numeric.
 
 
 
 
 
 
m_Filter 
 
 Filter   m_Filter  
 
 The filter used to standardize/normalize all values.
 
 
 
 
 
 
m_Missing 
 
 ReplaceMissingValues   m_Missing  
 
 The filter used to get rid of missing values.
 
 
 
 
 
 
m_classIndex 
 
int  m_classIndex  
 
 The class index from the training data
 
 
 
 
 
 
m_classAttribute 
 
 Attribute   m_classAttribute  
 
 The class attribute
 
 
 
 
 
 
m_KernelIsLinear 
 
boolean  m_KernelIsLinear  
 
 whether the kernel is a linear one
 
 
 
 
 
 
m_checksTurnedOff 
 
boolean  m_checksTurnedOff  
 
 Turn off all checks and conversions? Turning them off assumes
      that data is purely numeric, doesn't contain any missing values,
      and has a nominal class. Turning them off also means that
      no header information will be stored if the machine is linear. 
      Finally, it also assumes that no instance has a weight equal to 0.
 
 
 
 
 
 
m_fitLogisticModels 
 
boolean  m_fitLogisticModels  
 
 Whether logistic models are to be fit
 
 
 
 
 
 
m_numFolds 
 
int  m_numFolds  
 
 The number of folds for the internal cross-validation
 
 
 
 
 
 
m_randomSeed 
 
int  m_randomSeed  
 
 The random number seed
 
 
 
 
 
 
m_kernel 
 
 Kernel   m_kernel  
 
 the kernel to use
 
 
 
 

 
   
 
 
  
 Class  weka.classifiers.functions.SMO.BinarySMO  extends java.lang.Object implements Serializable   
 
 

 
 serialVersionUID:  -8246163625699362456L

 
   
 
 
  
 Serialized Fields   
 
 

 
m_alpha 
 
double[]  m_alpha  
 
 The Lagrange multipliers.
 
 
 
 
 
 
m_b 
 
double  m_b  
 
 The thresholds.
 
 
 
 
 
 
m_bLow 
 
double  m_bLow  
 
 The thresholds.
 
 
 
 
 
 
m_bUp 
 
double  m_bUp  
 
 The thresholds.
 
 
 
 
 
 
m_iLow 
 
int  m_iLow  
 
 The indices for m_bLow and m_bUp
 
 
 
 
 
 
m_iUp 
 
int  m_iUp  
 
 The indices for m_bLow and m_bUp
 
 
 
 
 
 
m_data 
 
 Instances   m_data  
 
 The training data.
 
 
 
 
 
 
m_weights 
 
double[]  m_weights  
 
 Weight vector for linear machine.
 
 
 
 
 
 
m_sparseWeights 
 
double[]  m_sparseWeights  
 
 Variables to hold weight vector in sparse form.
        (To reduce storage requirements.)
 
 
 
 
 
 
m_sparseIndices 
 
int[]  m_sparseIndices  
 
 
 
 
 
 
m_kernel 
 
 Kernel   m_kernel  
 
 Kernel to use
 
 
 
 
 
 
m_class 
 
double[]  m_class  
 
 The transformed class values.
 
 
 
 
 
 
m_errors 
 
double[]  m_errors  
 
 The current set of errors for all non-bound examples.
 
 
 
 
 
 
m_I0 
 
 SMOset   m_I0  
 
 {i: 0  
 
 
 
 
 
m_I1 
 
 SMOset   m_I1  
 
 {i: m_class[i] = 1, m_alpha[i] = 0}
 
 
 
 
 
 
m_I2 
 
 SMOset   m_I2  
 
 {i: m_class[i] = -1, m_alpha[i] =C}
 
 
 
 
 
 
m_I3 
 
 SMOset   m_I3  
 
 {i: m_class[i] = 1, m_alpha[i] = C}
 
 
 
 
 
 
m_I4 
 
 SMOset   m_I4  
 
 {i: m_class[i] = -1, m_alpha[i] = 0}
 
 
 
 
 
 
m_supportVectors 
 
 SMOset   m_supportVectors  
 
 The set of support vectors
 
 
 
 
 
 
m_logistic 
 
 Logistic   m_logistic  
 
 Stores logistic regression model for probability estimate
 
 
 
 
 
 
m_sumOfWeights 
 
double  m_sumOfWeights  
 
 Stores the weight of the training instances
 
 
 
 

 
   
 
 
  
 Class  weka.classifiers.functions.SMOreg  extends  Classifier  implements Serializable   
 
 

 
 serialVersionUID:  -7149606251113102827L

 
   
 
 
  
 Serialized Fields   
 
 

 
m_filterType 
 
int  m_filterType  
 
 Whether to normalize/standardize/neither
 
 
 
 
 
 
m_NominalToBinary 
 
 NominalToBinary   m_NominalToBinary  
 
 The filter used to make attributes numeric.
 
 
 
 
 
 
m_Filter 
 
 Filter   m_Filter  
 
 The filter used to standardize/normalize all values.
 
 
 
 
 
 
m_Missing 
 
 ReplaceMissingValues   m_Missing  
 
 The filter used to get rid of missing values.
 
 
 
 
 
 
m_onlyNumeric 
 
boolean  m_onlyNumeric  
 
 Only numeric attributes in the dataset? If so, less need to filter
 
 
 
 
 
 
m_C 
 
double  m_C  
 
 capacity parameter
 
 
 
 
 
 
m_x1 
 
double  m_x1  
 
 coefficients used by normalization filter for doing its linear transformation 
 so that result = svmoutput * m_x1 + m_x0
 
 
 
 
 
 
m_x0 
 
double  m_x0  
 
 
 
 
 
 
m_optimizer 
 
 RegOptimizer   m_optimizer  
 
 contains the algorithm used for learning
 
 
 
 
 
 
m_kernel 
 
 Kernel   m_kernel  
 
 the configured kernel
 
 
 
 

 
   
 
 
  
 Class  weka.classifiers.functions.SPegasos  extends  Classifier  implements Serializable   
 
 

 
 serialVersionUID:  -3732968666673530290L

 
   
 
 
  
 Serialized Fields   
 
 

 
m_replaceMissing 
 
 ReplaceMissingValues   m_replaceMissing  
 
 Replace missing values
 
 
 
 
 
 
m_nominalToBinary 
 
 NominalToBinary   m_nominalToBinary  
 
 Convert nominal attributes to numerically coded binary ones
 
 
 
 
 
 
m_normalize 
 
 Normalize   m_normalize  
 
 Normalize the training data
 
 
 
 
 
 
m_lambda 
 
double  m_lambda  
 
 The regularization parameter
 
 
 
 
 
 
m_weights 
 
double[]  m_weights  
 
 Stores the weights (+ bias in the last element)
 
 
 
 
 
 
m_t 
 
double  m_t  
 
 Holds the current iteration number
 
 
 
 
 
 
m_epochs 
 
int  m_epochs  
 
 The number of epochs to perform (batch learning). Total iterations is
  m_epochs * num instances
 
 
 
 
 
 
m_dontNormalize 
 
boolean  m_dontNormalize  
 
 Turn off normalization of the input data. This option gets
 forced for incremental training.
 
 
 
 
 
 
m_dontReplaceMissing 
 
boolean  m_dontReplaceMissing  
 
 Turn off global replacement of missing values. Missing values
  will be ignored instead. This option gets forced for
  incremental training.
 
 
 
 
 
 
m_data 
 
 Instances   m_data  
 
 Holds the header of the training data
 
 
 
 
 
 
m_loss 
 
int  m_loss  
 
 The current loss function to minimize
 
 
 
 

 
   
 
 
  
 Class  weka.classifiers.functions.VotedPerceptron  extends  Classifier  implements Serializable   
 
 

 
 serialVersionUID:  -1072429260104568698L

 
   
 
 
  
 Serialized Fields   
 
 

 
m_MaxK 
 
int  m_MaxK  
 
 The maximum number of alterations to the perceptron
 
 
 
 
 
 
m_NumIterations 
 
int  m_NumIterations  
 
 The number of iterations
 
 
 
 
 
 
m_Exponent 
 
double  m_Exponent  
 
 The exponent
 
 
 
 
 
 
m_K 
 
int  m_K  
 
 The actual number of alterations
 
 
 
 
 
 
m_Additions 
 
int[]  m_Additions  
 
 The training instances added to the perceptron
 
 
 
 
 
 
m_IsAddition 
 
boolean[]  m_IsAddition  
 
 Addition or subtraction?
 
 
 
 
 
 
m_Weights 
 
int[]  m_Weights  
 
 The weights for each perceptron
 
 
 
 
 
 
m_Train 
 
 Instances   m_Train  
 
 The training instances
 
 
 
 
 
 
m_Seed 
 
int  m_Seed  
 
 Seed used for shuffling the dataset
 
 
 
 
 
 
m_NominalToBinary 
 
 NominalToBinary   m_NominalToBinary  
 
 The filter used to make attributes numeric.
 
 
 
 
 
 
m_ReplaceMissingValues 
 
 ReplaceMissingValues   m_ReplaceMissingValues  
 
 The filter used to get rid of missing values.
 
 
 
 

 
   
 
 
  
 Class  weka.classifiers.functions.Winnow  extends  Classifier  implements Serializable   
 
 

 
 serialVersionUID:  3543770107994321324L

 
   
 
 
  
 Serialized Fields   
 
 

 
m_Balanced 
 
boolean  m_Balanced  
 
 Use the balanced variant?
 
 
 
 
 
 
m_numIterations 
 
int  m_numIterations  
 
 The number of iterations
 
 
 
 
 
 
m_Alpha 
 
double  m_Alpha  
 
 The promotion coefficient
 
 
 
 
 
 
m_Beta 
 
double  m_Beta  
 
 The demotion coefficient
 
 
 
 
 
 
m_Threshold 
 
double  m_Threshold  
 
 Prediction threshold,  
 
 
 
 
 
m_Seed 
 
int  m_Seed  
 
 Random seed used for shuffling the dataset, -1 == disable
 
 
 
 
 
 
m_Mistakes 
 
int  m_Mistakes  
 
 Accumulated mistake count (for statistics)
 
 
 
 
 
 
m_defaultWeight 
 
double  m_defaultWeight  
 
 Starting weights for the prediction vector(s)
 
 
 
 
 
 
m_predPosVector 
 
double[]  m_predPosVector  
 
 The weight vector for prediction (pos)
 
 
 
 
 
 
m_predNegVector 
 
double[]  m_predNegVector  
 
 The weight vector for prediction (neg)
 
 
 
 
 
 
m_actualThreshold 
 
double  m_actualThreshold  
 
 The true threshold used for prediction
 
 
 
 
 
 
m_Train 
 
 Instances   m_Train  
 
 The training instances
 
 
 
 
 
 
m_NominalToBinary 
 
 NominalToBinary   m_NominalToBinary  
 
 The filter used to make attributes numeric.
 
 
 
 
 
 
m_ReplaceMissingValues 
 
 ReplaceMissingValues   m_ReplaceMissingValues  
 
 The filter used to get rid of missing values.
 
 
 
 
 

 
 
  
 Package   weka.classifiers.functions.neural   
 
 

 
   
 
 
  
 Class  weka.classifiers.functions.neural.LinearUnit  extends java.lang.Object implements Serializable   
 
 

 
 serialVersionUID:  8572152807755673630L

 

 
   
 
 
  
 Class  weka.classifiers.functions.neural.NeuralConnection  extends java.lang.Object implements Serializable   
 
 

 
 serialVersionUID:  -286208828571059163L

 
   
 
 
  
 Serialized Fields   
 
 

 
m_inputList 
 
 NeuralConnection []  m_inputList  
 
 The list of inputs to this unit.
 
 
 
 
 
 
m_outputList 
 
 NeuralConnection []  m_outputList  
 
 The list of outputs from this unit.
 
 
 
 
 
 
m_inputNums 
 
int[]  m_inputNums  
 
 The numbering for the connections at the other end of the input lines.
 
 
 
 
 
 
m_outputNums 
 
int[]  m_outputNums  
 
 The numbering for the connections at the other end of the out lines.
 
 
 
 
 
 
m_numInputs 
 
int  m_numInputs  
 
 The number of inputs.
 
 
 
 
 
 
m_numOutputs 
 
int  m_numOutputs  
 
 The number of outputs.
 
 
 
 
 
 
m_unitValue 
 
double  m_unitValue  
 
 The output value for this unit, NaN if not calculated.
 
 
 
 
 
 
m_unitError 
 
double  m_unitError  
 
 The error value for this unit, NaN if not calculated.
 
 
 
 
 
 
m_weightsUpdated 
 
boolean  m_weightsUpdated  
 
 True if the weights have already been updated.
 
 
 
 
 
 
m_id 
 
java.lang.String  m_id  
 
 The string that uniquely (provided naming is done properly) identifies
 this unit.
 
 
 
 
 
 
m_type 
 
int  m_type  
 
 The type of unit this is.
 
 
 
 
 
 
m_x 
 
double  m_x  
 
 The x coord of this unit purely for displaying purposes.
 
 
 
 
 
 
m_y 
 
double  m_y  
 
 The y coord of this unit purely for displaying purposes.
 
 
 
 

 
   
 
 
  
 Class  weka.classifiers.functions.neural.NeuralNode  extends  NeuralConnection  implements Serializable   
 
 

 
 serialVersionUID:  -1085750607680839163L

 
   
 
 
  
 Serialized Fields   
 
 

 
m_weights 
 
double[]  m_weights  
 
 The weights for each of the input connections, and the threshold.
 
 
 
 
 
 
m_bestWeights 
 
double[]  m_bestWeights  
 
 The best (lowest error) weights. Only used when validation set is used
 
 
 
 
 
 
m_changeInWeights 
 
double[]  m_changeInWeights  
 
 The change in the weights.
 
 
 
 
 
 
m_random 
 
java.util.Random  m_random  
 
 
 
 
 
 
m_methods 
 
 NeuralMethod   m_methods  
 
 Performs the operations for this node. Currently this
 defines that the node is either a sigmoid or a linear unit.
 
 
 
 

 
   
 
 
  
 Class  weka.classifiers.functions.neural.SigmoidUnit  extends java.lang.Object implements Serializable   
 
 

 
 serialVersionUID:  -5162958458177475652L

 
 

 
 
  
 Package   weka.classifiers.functions.pace   
 
 

 
   
 
 
  
 Class  weka.classifiers.functions.pace.PaceMatrix  extends  Matrix  implements Serializable   
 
 

 
 serialVersionUID:  2699925616857843973L

 
 

 
 
  
 Package   weka.classifiers.functions.supportVector   
 
 

 
   
 
 
  
 Class  weka.classifiers.functions.supportVector.CachedKernel  extends  Kernel  implements Serializable   
 
 

 
 serialVersionUID:  702810182699015136L

 
   
 
 
  
 Serialized Fields   
 
 

 
m_kernelEvals 
 
int  m_kernelEvals  
 
 Counts the number of kernel evaluations.
 
 
 
 
 
 
m_cacheHits 
 
int  m_cacheHits  
 
 Counts the number of kernel cache hits.
 
 
 
 
 
 
m_cacheSize 
 
int  m_cacheSize  
 
 The size of the cache (a prime number)
 
 
 
 
 
 
m_storage 
 
double[]  m_storage  
 
 Kernel cache
 
 
 
 
 
 
m_keys 
 
long[]  m_keys  
 
 
 
 
 
 
m_kernelMatrix 
 
double[][]  m_kernelMatrix  
 
 The kernel matrix if full cache is used (i.e. size is set to 0)
 
 
 
 
 
 
m_numInsts 
 
int  m_numInsts  
 
 The number of instance in the dataset
 
 
 
 
 
 
m_cacheSlots 
 
int  m_cacheSlots  
 
 number of cache slots in an entry
 
 
 
 

 
   
 
 
  
 Class  weka.classifiers.functions.supportVector.Kernel  extends java.lang.Object implements Serializable   
 
 

 
 serialVersionUID:  -6102771099905817064L

 
   
 
 
  
 Serialized Fields   
 
 

 
m_data 
 
 Instances   m_data  
 
 The dataset
 
 
 
 
 
 
m_Debug 
 
boolean  m_Debug  
 
 enables debugging output
 
 
 
 
 
 
m_ChecksTurnedOff 
 
boolean  m_ChecksTurnedOff  
 
 Turns off all checks
 
 
 
 

 
   
 
 
  
 Class  weka.classifiers.functions.supportVector.NormalizedPolyKernel  extends  PolyKernel  implements Serializable   
 
 

 
 serialVersionUID:  1248574185532130851L

 

 
   
 
 
  
 Class  weka.classifiers.functions.supportVector.PolyKernel  extends  CachedKernel  implements Serializable   
 
 

 
 serialVersionUID:  -321831645846363201L

 
   
 
 
  
 Serialized Fields   
 
 

 
m_lowerOrder 
 
boolean  m_lowerOrder  
 
 Use lower-order terms?
 
 
 
 
 
 
m_exponent 
 
double  m_exponent  
 
 The exponent for the polynomial kernel.
 
 
 
 

 
   
 
 
  
 Class  weka.classifiers.functions.supportVector.PrecomputedKernelMatrixKernel  extends  Kernel  implements Serializable   
 
 

 
 serialVersionUID:  -321831645846363333L

 
   
 
 
  
 Serialized Fields   
 
 

 
m_KernelMatrixFile 
 
java.io.File  m_KernelMatrixFile  
 
 The file holding the kernel matrix.
 
 
 
 
 
 
m_KernelMatrix 
 
 Matrix   m_KernelMatrix  
 
 The kernel matrix.
 
 
 
 
 
 
m_Counter 
 
int  m_Counter  
 
 A classifier counter.
 
 
 
 

 
   
 
 
  
 Class  weka.classifiers.functions.supportVector.Puk  extends  CachedKernel  implements Serializable   
 
 

 
 serialVersionUID:  1682161522559978851L

 
   
 
 
  
 Serialized Fields   
 
 

 
m_kernelPrecalc 
 
double[]  m_kernelPrecalc  
 
 The precalculated dotproducts of <inst_i,inst_i>
 
 
 
 
 
 
m_omega 
 
double  m_omega  
 
 Omega for the Puk kernel.
 
 
 
 
 
 
m_sigma 
 
double  m_sigma  
 
 Sigma for the Puk kernel.
 
 
 
 
 
 
m_factor 
 
double  m_factor  
 
 Cached factor for the Puk kernel.
 
 
 
 

 
   
 
 
  
 Class  weka.classifiers.functions.supportVector.RBFKernel  extends  CachedKernel  implements Serializable   
 
 

 
 serialVersionUID:  5247117544316387852L

 
   
 
 
  
 Serialized Fields   
 
 

 
m_kernelPrecalc 
 
double[]  m_kernelPrecalc  
 
 The precalculated dotproducts of <inst_i,inst_i>
 
 
 
 
 
 
m_gamma 
 
double  m_gamma  
 
 Gamma for the RBF kernel.
 
 
 
 

 
   
 
 
  
 Class  weka.classifiers.functions.supportVector.RegOptimizer  extends java.lang.Object implements Serializable   
 
 

 
 serialVersionUID:  -2198266997254461814L

 
   
 
 
  
 Serialized Fields   
 
 

 
m_alpha 
 
double[]  m_alpha  
 
 alpha and alpha* arrays containing weights for solving dual problem
 
 
 
 
 
 
m_alphaStar 
 
double[]  m_alphaStar  
 
 
 
 
 
 
m_b 
 
double  m_b  
 
 offset
 
 
 
 
 
 
m_epsilon 
 
double  m_epsilon  
 
 epsilon of epsilon-insensitive cost function
 
 
 
 
 
 
m_C 
 
double  m_C  
 
 capacity parameter, copied from SMOreg
 
 
 
 
 
 
m_target 
 
double[]  m_target  
 
 class values/desired output vector
 
 
 
 
 
 
m_data 
 
 Instances   m_data  
 
 points to data set
 
 
 
 
 
 
m_kernel 
 
 Kernel   m_kernel  
 
 the kernel
 
 
 
 
 
 
m_classIndex 
 
int  m_classIndex  
 
 index of class variable in data set
 
 
 
 
 
 
m_nInstances 
 
int  m_nInstances  
 
 number of instances in data set
 
 
 
 
 
 
m_random 
 
java.util.Random  m_random  
 
 random number generator
 
 
 
 
 
 
m_nSeed 
 
int  m_nSeed  
 
 seed for initializing random number generator
 
 
 
 
 
 
m_supportVectors 
 
 SMOset   m_supportVectors  
 
 set of support vectors, that is, vectors with alpha(*)!=0
 
 
 
 
 
 
m_nEvals 
 
int  m_nEvals  
 
 number of kernel evaluations, used for printing statistics only
 
 
 
 
 
 
m_nCacheHits 
 
int  m_nCacheHits  
 
 number of kernel cache hits, used for printing statistics only
 
 
 
 
 
 
m_weights 
 
double[]  m_weights  
 
 weights for linear kernel
 
 
 
 
 
 
m_sparseWeights 
 
double[]  m_sparseWeights  
 
 Variables to hold weight vector in sparse form.
   (To reduce storage requirements.)
 
 
 
 
 
 
m_sparseIndices 
 
int[]  m_sparseIndices  
 
 
 
 
 
 
m_bModelBuilt 
 
boolean  m_bModelBuilt  
 
 flag to indicate whether the model is built yet
 
 
 
 
 
 
m_SVM 
 
 SMOreg   m_SVM  
 
 parent SMOreg class
 
 
 
 

 
   
 
 
  
 Class  weka.classifiers.functions.supportVector.RegSMO  extends  RegOptimizer  implements Serializable   
 
 

 
 serialVersionUID:  -7504070793279598638L

 
   
 
 
  
 Serialized Fields   
 
 

 
m_eps 
 
double  m_eps  
 
 tolerance parameter, smaller changes on alpha in inner loop will be ignored
 
 
 
 
 
 
m_error 
 
double[]  m_error  
 
 error cache containing m_error[i] = SVMOutput(i) - m_target[i] - m_b  
 note, we don't need m_b in the cache, since if we do, we need to maintain 
 it when m_b is updated
 
 
 
 
 
 
m_alpha1 
 
double  m_alpha1  
 
 alpha value for first candidate
 
 
 
 
 
 
m_alpha1Star 
 
double  m_alpha1Star  
 
 alpha* value for first candidate
 
 
 
 
 
 
m_alpha2 
 
double  m_alpha2  
 
 alpha value for second candidate
 
 
 
 
 
 
m_alpha2Star 
 
double  m_alpha2Star  
 
 alpha* value for second candidate
 
 
 
 

 
   
 
 
  
 Class  weka.classifiers.functions.supportVector.RegSMOImproved  extends  RegSMO  implements Serializable   
 
 

 
 serialVersionUID:  471692841446029784L

 
   
 
 
  
 Serialized Fields   
 
 

 
m_I0 
 
 SMOset   m_I0  
 
 The different sets used by the algorithm.
 
 
 
 
 
 
m_iSet 
 
int[]  m_iSet  
 
 Index set {i: 0  
 
 
 
 
 
m_bUp 
 
double  m_bUp  
 
 b.up and b.low boundaries used to determine stopping criterion
 
 
 
 
 
 
m_bLow 
 
double  m_bLow  
 
 b.up and b.low boundaries used to determine stopping criterion
 
 
 
 
 
 
m_iUp 
 
int  m_iUp  
 
 index of the instance that gave us b.up and b.low
 
 
 
 
 
 
m_iLow 
 
int  m_iLow  
 
 index of the instance that gave us b.up and b.low
 
 
 
 
 
 
m_fTolerance 
 
double  m_fTolerance  
 
 tolerance parameter used for checking stopping criterion b.up  
 
 
 
 
 
m_bUseVariant1 
 
boolean  m_bUseVariant1  
 
 set true to use variant 1 of the paper, otherwise use variant 2
 
 
 
 

 
   
 
 
  
 Class  weka.classifiers.functions.supportVector.SMOset  extends java.lang.Object implements Serializable   
 
 

 
 serialVersionUID:  -8364829283188675777L

 
   
 
 
  
 Serialized Fields   
 
 

 
m_number 
 
int  m_number  
 
 The current number of elements in the set
 
 
 
 
 
 
m_first 
 
int  m_first  
 
 The first element in the set
 
 
 
 
 
 
m_indicators 
 
boolean[]  m_indicators  
 
 Indicators
 
 
 
 
 
 
m_next 
 
int[]  m_next  
 
 The next element for each element
 
 
 
 
 
 
m_previous 
 
int[]  m_previous  
 
 The previous element for each element
 
 
 
 

 
   
 
 
  
 Class  weka.classifiers.functions.supportVector.StringKernel  extends  Kernel  implements Serializable   
 
 

 
 serialVersionUID:  -4902954211202690123L

 
   
 
 
  
 Serialized Fields   
 
 

 
m_cacheSize 
 
int  m_cacheSize  
 
 The size of the cache (a prime number)
 
 
 
 
 
 
m_internalCacheSize 
 
int  m_internalCacheSize  
 
 The size of the internal cache for intermediate results (a prime number)
 
 
 
 
 
 
m_strAttr 
 
int  m_strAttr  
 
 The attribute number of the string attribute
 
 
 
 
 
 
m_storage 
 
double[]  m_storage  
 
 Kernel cache (i.e., cache for kernel evaluations)
 
 
 
 
 
 
m_keys 
 
long[]  m_keys  
 
 
 
 
 
 
m_kernelEvals 
 
int  m_kernelEvals  
 
 Counts the number of kernel evaluations.
 
 
 
 
 
 
m_numInsts 
 
int  m_numInsts  
 
 The number of instance in the dataset
 
 
 
 
 
 
m_PruningMethod 
 
int  m_PruningMethod  
 
 the pruning method
 
 
 
 
 
 
m_lambda 
 
double  m_lambda  
 
 the decay factor that penalizes non-continuous substring matches. See [1]
 for details.
 
 
 
 
 
 
m_subsequenceLength 
 
int  m_subsequenceLength  
 
 The substring length
 
 
 
 
 
 
m_maxSubsequenceLength 
 
int  m_maxSubsequenceLength  
 
 The maximum substring length for lambda pruning
 
 
 
 
 
 
m_powersOflambda 
 
double[]  m_powersOflambda  
 
 the precalculated powers of lambda
 
 
 
 
 
 
m_normalize 
 
boolean  m_normalize  
 
 flag for switching normalization on or off. This defaults to false and
 can be turned on by the switch for feature space normalization in SMO
 
 
 
 
 
 
maxCache 
 
int  maxCache  
 
 private cache for intermediate results
 
 
 
 
 
 
cachekh 
 
double[]  cachekh  
 
 
 
 
 
 
cachekhK 
 
int[]  cachekhK  
 
 
 
 
 
 
cachekh2 
 
double[]  cachekh2  
 
 
 
 
 
 
cachekh2K 
 
int[]  cachekh2K  
 
 
 
 
 
 
m_multX 
 
int  m_multX  
 
 cached indexes for private cache
 
 
 
 
 
 
m_multY 
 
int  m_multY  
 
 
 
 
 
 
m_multZ 
 
int  m_multZ  
 
 
 
 
 
 
m_multZZ 
 
int  m_multZZ  
 
 
 
 
 
 
m_useRecursionCache 
 
boolean  m_useRecursionCache  
 
 
 
 
 

 
 
  
 Package   weka.classifiers.lazy   
 
 

 
   
 
 
  
 Class  weka.classifiers.lazy.IB1  extends  Classifier  implements Serializable   
 
 

 
 serialVersionUID:  -6152184127304895851L

 
   
 
 
  
 Serialized Fields   
 
 

 
m_Train 
 
 Instances   m_Train  
 
 The training instances used for classification.
 
 
 
 
 
 
m_MinArray 
 
double[]  m_MinArray  
 
 The minimum values for numeric attributes.
 
 
 
 
 
 
m_MaxArray 
 
double[]  m_MaxArray  
 
 The maximum values for numeric attributes.
 
 
 
 

 
   
 
 
  
 Class  weka.classifiers.lazy.IBk  extends  Classifier  implements Serializable   
 
 

 
 serialVersionUID:  -3080186098777067172L

 
   
 
 
  
 Serialized Fields   
 
 

 
m_Train 
 
 Instances   m_Train  
 
 The training instances used for classification.
 
 
 
 
 
 
m_NumClasses 
 
int  m_NumClasses  
 
 The number of class values (or 1 if predicting numeric).
 
 
 
 
 
 
m_ClassType 
 
int  m_ClassType  
 
 The class attribute type.
 
 
 
 
 
 
m_kNN 
 
int  m_kNN  
 
 The number of neighbours to use for classification (currently).
 
 
 
 
 
 
m_kNNUpper 
 
int  m_kNNUpper  
 
 The value of kNN provided by the user. This may differ from
 m_kNN if cross-validation is being used.
 
 
 
 
 
 
m_kNNValid 
 
boolean  m_kNNValid  
 
 Whether the value of k selected by cross validation has
 been invalidated by a change in the training instances.
 
 
 
 
 
 
m_WindowSize 
 
int  m_WindowSize  
 
 The maximum number of training instances allowed. When
 this limit is reached, old training instances are removed,
 so the training data is "windowed". Set to 0 for unlimited
 numbers of instances.
 
 
 
 
 
 
m_DistanceWeighting 
 
int  m_DistanceWeighting  
 
 Whether the neighbours should be distance-weighted.
 
 
 
 
 
 
m_CrossValidate 
 
boolean  m_CrossValidate  
 
 Whether to select k by cross validation.
 
 
 
 
 
 
m_MeanSquared 
 
boolean  m_MeanSquared  
 
 Whether to minimise mean squared error rather than mean absolute
 error when cross-validating on numeric prediction tasks.
 
 
 
 
 
 
m_NNSearch 
 
 NearestNeighbourSearch   m_NNSearch  
 
 for nearest-neighbor search.
 
 
 
 
 
 
m_NumAttributesUsed 
 
double  m_NumAttributesUsed  
 
 The number of attributes the contribute to a prediction.
 
 
 
 
 
 
m_defaultModel 
 
 ZeroR   m_defaultModel  
 
 Default ZeroR model to use when there are no training instances
 
 
 
 

 
   
 
 
  
 Class  weka.classifiers.lazy.KStar  extends  Classifier  implements Serializable   
 
 

 
 serialVersionUID:  332458330800479083L

 
   
 
 
  
 Serialized Fields   
 
 

 
m_Train 
 
 Instances   m_Train  
 
 The training instances used for classification.
 
 
 
 
 
 
m_NumInstances 
 
int  m_NumInstances  
 
 The number of instances in the dataset
 
 
 
 
 
 
m_NumClasses 
 
int  m_NumClasses  
 
 The number of class values
 
 
 
 
 
 
m_NumAttributes 
 
int  m_NumAttributes  
 
 The number of attributes
 
 
 
 
 
 
m_ClassType 
 
int  m_ClassType  
 
 The class attribute type
 
 
 
 
 
 
m_RandClassCols 
 
int[][]  m_RandClassCols  
 
 Table of random class value colomns
 
 
 
 
 
 
m_ComputeRandomCols 
 
int  m_ComputeRandomCols  
 
 Flag turning on and off the computation of random class colomns
 
 
 
 
 
 
m_InitFlag 
 
int  m_InitFlag  
 
 Flag turning on and off the initialisation of config variables
 
 
 
 
 
 
m_Cache 
 
 KStarCache []  m_Cache  
 
 A custom data structure for caching distinct attribute values
 and their scale factor or stop parameter.
 
 
 
 
 
 
m_MissingMode 
 
int  m_MissingMode  
 
 missing value treatment
 
 
 
 
 
 
m_BlendMethod 
 
int  m_BlendMethod  
 
 0 = use specified blend, 1 = entropic blend setting
 
 
 
 
 
 
m_GlobalBlend 
 
int  m_GlobalBlend  
 
 default sphere of influence blend setting
 
 
 
 

 
   
 
 
  
 Class  weka.classifiers.lazy.LBR  extends  Classifier  implements Serializable   
 
 

 
 serialVersionUID:  5648559277738985156L

 
   
 
 
  
 Serialized Fields   
 
 

 
m_Counts 
 
int[][][]  m_Counts  
 
 All the counts for nominal attributes.
 
 
 
 
 
 
m_tCounts 
 
int[][][]  m_tCounts  
 
 All the counts for nominal attributes.
 
 
 
 
 
 
m_Priors 
 
int[]  m_Priors  
 
 The prior probabilities of the classes.
 
 
 
 
 
 
m_tPriors 
 
int[]  m_tPriors  
 
 The prior probabilities of the classes.
 
 
 
 
 
 
m_numAtts 
 
int  m_numAtts  
 
 number of attributes for the dataset
 
 
 
 
 
 
m_numClasses 
 
int  m_numClasses  
 
 number of classes for dataset
 
 
 
 
 
 
m_numInsts 
 
int  m_numInsts  
 
 number of instances in dataset
 
 
 
 
 
 
m_Instances 
 
 Instances   m_Instances  
 
 The set of instances used for current training.
 
 
 
 
 
 
m_Errors 
 
int  m_Errors  
 
 leave-one-out errors on the training dataset.
 
 
 
 
 
 
m_ErrorFlags 
 
boolean[]  m_ErrorFlags  
 
 leave-one-out error flags on the training dataaet.
 
 
 
 
 
 
leftHand 
 
java.util.ArrayList<E>  leftHand  
 
 best attribute's index list. maybe as output result
 
 
 
 
 
 
m_subOldErrorFlags 
 
boolean[]  m_subOldErrorFlags  
 
 following is defined by wangzh, 
 the number of instances to be classified incorrectly
 on the subset.
 
 
 
 
 
 
m_RemainderErrors 
 
int  m_RemainderErrors  
 
 the number of instances to be classified incorrectly
 besides the subset.
 
 
 
 
 
 
m_Number 
 
int  m_Number  
 
 the number of instance to be processed
 
 
 
 
 
 
m_NumberOfInstances 
 
int  m_NumberOfInstances  
 
 the Number of Instances to be used in building a classifiers
 
 
 
 
 
 
m_NCV 
 
boolean  m_NCV  
 
 for printing in n-fold cross validation
 
 
 
 
 
 
m_subInstances 
 
 LBR.Indexes   m_subInstances  
 
 index of instances and attributes for the given dataset
 
 
 
 
 
 
tempSubInstances 
 
 LBR.Indexes   tempSubInstances  
 
 index of instances and attributes for the given dataset
 
 
 
 
 
 
posteriorsArray 
 
double[]  posteriorsArray  
 
 probability values array
 
 
 
 
 
 
bestCnt 
 
int  bestCnt  
 
 
 
 
 
 
tempCnt 
 
int  tempCnt  
 
 
 
 
 
 
forCnt 
 
int  forCnt  
 
 
 
 
 
 
whileCnt 
 
int  whileCnt  
 
 
 
 

 
   
 
 
  
 Class  weka.classifiers.lazy.LBR.Indexes  extends java.lang.Object implements Serializable   
 
 

 
 serialVersionUID:  -2771490019751421307L

 
   
 
 
  
 Serialized Fields   
 
 

 
m_InstIndexes 
 
boolean[]  m_InstIndexes  
 
 the array instance indexes
 
 
 
 
 
 
m_AttIndexes 
 
boolean[]  m_AttIndexes  
 
 the array attribute indexes
 
 
 
 
 
 
m_NumInstances 
 
int  m_NumInstances  
 
 the number of instances indexed
 
 
 
 
 
 
m_NumAtts 
 
int  m_NumAtts  
 
 the number of attributes indexed
 
 
 
 
 
 
m_SequentialInstIndexes 
 
int[]  m_SequentialInstIndexes  
 
 the array of instance indexes that are set to a either true or false
 
 
 
 
 
 
m_SequentialAttIndexes 
 
int[]  m_SequentialAttIndexes  
 
 an array of attribute indexes that are set to either true or false
 
 
 
 
 
 
m_SequentialInstanceIndex_valid 
 
boolean  m_SequentialInstanceIndex_valid  
 
 flag to check if sequential array must be rebuilt due to changes to the instance index
 
 
 
 
 
 
m_SequentialAttIndex_valid 
 
boolean  m_SequentialAttIndex_valid  
 
 flag to check if sequential array must be rebuilt due to changes to the attribute index
 
 
 
 
 
 
m_NumInstsSet 
 
int  m_NumInstsSet  
 
 the number of instances "in use"  or set to a the original value (true or false)
 
 
 
 
 
 
m_NumAttsSet 
 
int  m_NumAttsSet  
 
 the number of attributes "in use"  or set to a the original value (true or false)
 
 
 
 
 
 
m_NumSeqInstsSet 
 
int  m_NumSeqInstsSet  
 
 the number of sequential instances "in use"  or set to a the original value (true or false)
 
 
 
 
 
 
m_NumSeqAttsSet 
 
int  m_NumSeqAttsSet  
 
 the number of sequential attributes "in use"  or set to a the original value (true or false)
 
 
 
 
 
 
m_ClassIndex 
 
int  m_ClassIndex  
 
 the Class Index for the data set
 
 
 
 

 
   
 
 
  
 Class  weka.classifiers.lazy.LWL  extends  SingleClassifierEnhancer  implements Serializable   
 
 

 
 serialVersionUID:  1979797405383665815L

 
   
 
 
  
 Serialized Fields   
 
 

 
m_Train 
 
 Instances   m_Train  
 
 The training instances used for classification.
 
 
 
 
 
 
m_kNN 
 
int  m_kNN  
 
 The number of neighbours used to select the kernel bandwidth.
 
 
 
 
 
 
m_WeightKernel 
 
int  m_WeightKernel  
 
 The weighting kernel method currently selected.
 
 
 
 
 
 
m_UseAllK 
 
boolean  m_UseAllK  
 
 True if m_kNN should be set to all instances.
 
 
 
 
 
 
m_NNSearch 
 
 NearestNeighbourSearch   m_NNSearch  
 
 The nearest neighbour search algorithm to use. 
 (Default: weka.core.neighboursearch.LinearNNSearch)
 
 
 
 
 
 
m_ZeroR 
 
 Classifier   m_ZeroR  
 
 a ZeroR model in case no model can be built from the data.
 
 
 
 
 

 
 
  
 Package   weka.classifiers.lazy.kstar   
 
 

 
   
 
 
  
 Class  weka.classifiers.lazy.kstar.KStarCache  extends java.lang.Object implements Serializable   
 
 

 
 serialVersionUID:  -7693632394267140678L

 
   
 
 
  
 Serialized Fields   
 
 

 
m_Cache 
 
 KStarCache.CacheTable   m_Cache  
 
 cache table
 
 
 
 

 
   
 
 
  
 Class  weka.classifiers.lazy.kstar.KStarCache.CacheTable  extends java.lang.Object implements Serializable   
 
 

 
 serialVersionUID:  -8086106452588253423L

 
   
 
 
  
 Serialized Fields   
 
 

 
m_Table 
 
 KStarCache.TableEntry []  m_Table  
 
 The hash table data.
 
 
 
 
 
 
m_Count 
 
int  m_Count  
 
 The total number of entries in the hash table.
 
 
 
 
 
 
m_Threshold 
 
int  m_Threshold  
 
 Rehashes the table when count exceeds this threshold.
 
 
 
 
 
 
m_LoadFactor 
 
float  m_LoadFactor  
 
 The load factor for the hashtable.
 
 
 
 
 
 
DEFAULT_TABLE_SIZE 
 
int  DEFAULT_TABLE_SIZE  
 
 The default size of the hashtable
 
 
 
 
 
 
DEFAULT_LOAD_FACTOR 
 
float  DEFAULT_LOAD_FACTOR  
 
 The default load factor for the hashtable
 
 
 
 
 
 
EPSILON 
 
double  EPSILON  
 
 Accuracy value for equality
 
 
 
 

 
   
 
 
  
 Class  weka.classifiers.lazy.kstar.KStarCache.TableEntry  extends java.lang.Object implements Serializable   
 
 

 
 serialVersionUID:  4057602386766259138L

 
   
 
 
  
 Serialized Fields   
 
 

 
hash 
 
int  hash  
 
 attribute value hash code
 
 
 
 
 
 
key 
 
double  key  
 
 attribute value
 
 
 
 
 
 
value 
 
double  value  
 
 scale factor or stop parameter
 
 
 
 
 
 
pmiss 
 
double  pmiss  
 
 transformation probability to missing value
 
 
 
 
 
 
next 
 
 KStarCache.TableEntry   next  
 
 next table entry (separate chaining)
 
 
 
 
 

 
 
  
 Package   weka.classifiers.meta   
 
 

 
   
 
 
  
 Class  weka.classifiers.meta.AdaBoostM1  extends  RandomizableIteratedSingleClassifierEnhancer  implements Serializable   
 
 

 
 serialVersionUID:  -7378107808933117974L

 
   
 
 
  
 Serialized Fields   
 
 

 
m_Betas 
 
double[]  m_Betas  
 
 Array for storing the weights for the votes.
 
 
 
 
 
 
m_NumIterationsPerformed 
 
int  m_NumIterationsPerformed  
 
 The number of successfully generated base classifiers.
 
 
 
 
 
 
m_WeightThreshold 
 
int  m_WeightThreshold  
 
 Weight Threshold. The percentage of weight mass used in training
 
 
 
 
 
 
m_UseResampling 
 
boolean  m_UseResampling  
 
 Use boosting with reweighting?
 
 
 
 
 
 
m_NumClasses 
 
int  m_NumClasses  
 
 The number of classes
 
 
 
 
 
 
m_ZeroR 
 
 Classifier   m_ZeroR  
 
 a ZeroR model in case no model can be built from the data
 
 
 
 

 
   
 
 
  
 Class  weka.classifiers.meta.AdditiveRegression  extends  IteratedSingleClassifierEnhancer  implements Serializable   
 
 

 
 serialVersionUID:  -2368937577670527151L

 
   
 
 
  
 Serialized Fields   
 
 

 
m_shrinkage 
 
double  m_shrinkage  
 
 Shrinkage (Learning rate). Default = no shrinkage.
 
 
 
 
 
 
m_NumIterationsPerformed 
 
int  m_NumIterationsPerformed  
 
 The number of successfully generated base classifiers.
 
 
 
 
 
 
m_zeroR 
 
 ZeroR   m_zeroR  
 
 The model for the mean
 
 
 
 
 
 
m_SuitableData 
 
boolean  m_SuitableData  
 
 whether we have suitable data or nor (if not, ZeroR model is used)
 
 
 
 

 
   
 
 
  
 Class  weka.classifiers.meta.AttributeSelectedClassifier  extends  SingleClassifierEnhancer  implements Serializable   
 
 

 
 serialVersionUID:  -5951805453487947577L

 
   
 
 
  
 Serialized Fields   
 
 

 
m_AttributeSelection 
 
 AttributeSelection   m_AttributeSelection  
 
 The attribute selection object
 
 
 
 
 
 
m_Evaluator 
 
 ASEvaluation   m_Evaluator  
 
 The attribute evaluator to use
 
 
 
 
 
 
m_Search 
 
 ASSearch   m_Search  
 
 The search method to use
 
 
 
 
 
 
m_ReducedHeader 
 
 Instances   m_ReducedHeader  
 
 The header of the dimensionally reduced data
 
 
 
 
 
 
m_numClasses 
 
int  m_numClasses  
 
 The number of class vals in the training data (1 if class is numeric)
 
 
 
 
 
 
m_numAttributesSelected 
 
double  m_numAttributesSelected  
 
 The number of attributes selected by the attribute selection phase
 
 
 
 
 
 
m_selectionTime 
 
double  m_selectionTime  
 
 The time taken to select attributes in milliseconds
 
 
 
 
 
 
m_totalTime 
 
double  m_totalTime  
 
 The time taken to select attributes AND build the classifier
 
 
 
 

 
   
 
 
  
 Class  weka.classifiers.meta.Bagging  extends  RandomizableIteratedSingleClassifierEnhancer  implements Serializable   
 
 

 
 serialVersionUID:  -505879962237199703L

 
   
 
 
  
 Serialized Fields   
 
 

 
m_BagSizePercent 
 
int  m_BagSizePercent  
 
 The size of each bag sample, as a percentage of the training size
 
 
 
 
 
 
m_CalcOutOfBag 
 
boolean  m_CalcOutOfBag  
 
 Whether to calculate the out of bag error
 
 
 
 
 
 
m_OutOfBagError 
 
double  m_OutOfBagError  
 
 The out of bag error that has been calculated
 
 
 
 

 
   
 
 
  
 Class  weka.classifiers.meta.ClassificationViaClustering  extends  Classifier  implements Serializable   
 
 

 
 serialVersionUID:  -5687069451420259135L

 
   
 
 
  
 Serialized Fields   
 
 

 
m_Clusterer 
 
 Clusterer   m_Clusterer  
 
 the cluster algorithm used (template)
 
 
 
 
 
 
m_ActualClusterer 
 
 Clusterer   m_ActualClusterer  
 
 the actual cluster algorithm being used
 
 
 
 
 
 
m_OriginalHeader 
 
 Instances   m_OriginalHeader  
 
 the original training data header
 
 
 
 
 
 
m_ClusteringHeader 
 
 Instances   m_ClusteringHeader  
 
 the modified training data header
 
 
 
 
 
 
m_ClustersToClasses 
 
double[]  m_ClustersToClasses  
 
 the mapping between clusters and classes
 
 
 
 
 
 
m_ZeroR 
 
 Classifier   m_ZeroR  
 
 the default model
 
 
 
 

 
   
 
 
  
 Class  weka.classifiers.meta.ClassificationViaRegression  extends  SingleClassifierEnhancer  implements Serializable   
 
 

 
 serialVersionUID:  4500023123618669859L

 
   
 
 
  
 Serialized Fields   
 
 

 
m_Classifiers 
 
 Classifier []  m_Classifiers  
 
 The classifiers. (One for each class.)
 
 
 
 
 
 
m_ClassFilters 
 
 MakeIndicator []  m_ClassFilters  
 
 The filters used to transform the class.
 
 
 
 

 
   
 
 
  
 Class  weka.classifiers.meta.CostSensitiveClassifier  extends  RandomizableSingleClassifierEnhancer  implements Serializable   
 
 

 
 serialVersionUID:  -720658209263002404L

 
   
 
 
  
 Serialized Fields   
 
 

 
m_MatrixSource 
 
int  m_MatrixSource  
 
 Indicates the current cost matrix source
 
 
 
 
 
 
m_OnDemandDirectory 
 
java.io.File  m_OnDemandDirectory  
 
 The directory used when loading cost files on demand, null indicates
 current directory
 
 
 
 
 
 
m_CostFile 
 
java.lang.String  m_CostFile  
 
 The name of the cost file, for command line options
 
 
 
 
 
 
m_CostMatrix 
 
 CostMatrix   m_CostMatrix  
 
 The cost matrix
 
 
 
 
 
 
m_MinimizeExpectedCost 
 
boolean  m_MinimizeExpectedCost  
 
 True if the costs should be used by selecting the minimum expected
 cost (false means weight training data by the costs)
 
 
 
 

 
   
 
 
  
 Class  weka.classifiers.meta.CVParameterSelection  extends  RandomizableSingleClassifierEnhancer  implements Serializable   
 
 

 
 serialVersionUID:  -6529603380876641265L

 
   
 
 
  
 Serialized Fields   
 
 

 
m_ClassifierOptions 
 
java.lang.String[]  m_ClassifierOptions  
 
 The base classifier options (not including those being set
 by cross-validation)
 
 
 
 
 
 
m_BestClassifierOptions 
 
java.lang.String[]  m_BestClassifierOptions  
 
 The set of all classifier options as determined by cross-validation
 
 
 
 
 
 
m_InitOptions 
 
java.lang.String[]  m_InitOptions  
 
 The set of all options at initialization time. So that getOptions
      can return this.
 
 
 
 
 
 
m_BestPerformance 
 
double  m_BestPerformance  
 
 The cross-validated performance of the best options
 
 
 
 
 
 
m_CVParams 
 
 FastVector   m_CVParams  
 
 The set of parameters to cross-validate over
 
 
 
 
 
 
m_NumAttributes 
 
int  m_NumAttributes  
 
 The number of attributes in the data
 
 
 
 
 
 
m_TrainFoldSize 
 
int  m_TrainFoldSize  
 
 The number of instances in a training fold
 
 
 
 
 
 
m_NumFolds 
 
int  m_NumFolds  
 
 The number of folds used in cross-validation
 
 
 
 

 
   
 
 
  
 Class weka.classifiers.meta.CVParameterSelection.CVParameter extends java.lang.Object implements Serializable   
 
 

 
 serialVersionUID:  -4668812017709421953L

 
   
 
 
  
 Serialized Fields   
 
 

 
m_ParamChar 
 
char  m_ParamChar  
 
 Char used to identify the option of interest
 
 
 
 
 
 
m_Lower 
 
double  m_Lower  
 
 Lower bound for the CV search
 
 
 
 
 
 
m_Upper 
 
double  m_Upper  
 
 Upper bound for the CV search
 
 
 
 
 
 
m_Steps 
 
double  m_Steps  
 
 Number of steps during the search
 
 
 
 
 
 
m_ParamValue 
 
double  m_ParamValue  
 
 The parameter value with the best performance
 
 
 
 
 
 
m_AddAtEnd 
 
boolean  m_AddAtEnd  
 
 True if the parameter should be added at the end of the argument list
 
 
 
 
 
 
m_RoundParam 
 
boolean  m_RoundParam  
 
 True if the parameter should be rounded to an integer
 
 
 
 

 
   
 
 
  
 Class  weka.classifiers.meta.Dagging  extends  RandomizableSingleClassifierEnhancer  implements Serializable   
 
 

 
 serialVersionUID:  4560165876570074309L

 
   
 
 
  
 Serialized Fields   
 
 

 
m_NumFolds 
 
int  m_NumFolds  
 
 the number of folds to use to split the training data
 
 
 
 
 
 
m_Vote 
 
 Vote   m_Vote  
 
 the classifier used for voting
 
 
 
 
 
 
m_Verbose 
 
boolean  m_Verbose  
 
 whether to output some progress information during building
 
 
 
 

 
   
 
 
  
 Class  weka.classifiers.meta.Decorate  extends  RandomizableIteratedSingleClassifierEnhancer  implements Serializable   
 
 

 
 serialVersionUID:  -6020193348750269931L

 
   
 
 
  
 Serialized Fields   
 
 

 
m_Committee 
 
java.util.Vector<E>  m_Committee  
 
 Vector of classifiers that make up the committee/ensemble.
 
 
 
 
 
 
m_DesiredSize 
 
int  m_DesiredSize  
 
 The desired ensemble size.
 
 
 
 
 
 
m_ArtSize 
 
double  m_ArtSize  
 
 Amount of artificial/random instances to use - specified as a
        fraction of the training data size.
 
 
 
 
 
 
m_Random 
 
java.util.Random  m_Random  
 
 The random number generator.
 
 
 
 
 
 
m_AttributeStats 
 
java.util.Vector<E>  m_AttributeStats  
 
 Attribute statistics - used for generating artificial examples.
 
 
 
 

 
   
 
 
  
 Class  weka.classifiers.meta.END  extends  RandomizableIteratedSingleClassifierEnhancer  implements Serializable   
 
 

 
 serialVersionUID:  -4143242362912214956L

 
   
 
 
  
 Serialized Fields   
 
 

 
m_hashtable 
 
java.util.Hashtable<K,V>  m_hashtable  
 
 The hashtable containing the classifiers for the END.
 
 
 
 

 
   
 
 
  
 Class  weka.classifiers.meta.FilteredClassifier  extends  SingleClassifierEnhancer  implements Serializable   
 
 

 
 serialVersionUID:  -4523450618538717400L

 
   
 
 
  
 Serialized Fields   
 
 

 
m_Filter 
 
 Filter   m_Filter  
 
 The filter
 
 
 
 
 
 
m_FilteredInstances 
 
 Instances   m_FilteredInstances  
 
 The instance structure of the filtered instances
 
 
 
 

 
   
 
 
  
 Class  weka.classifiers.meta.Grading  extends  Stacking  implements Serializable   
 
 

 
 serialVersionUID:  5207837947890081170L

 
   
 
 
  
 Serialized Fields   
 
 

 
m_MetaClassifiers 
 
 Classifier []  m_MetaClassifiers  
 
 The meta classifiers, one for each base classifier.
 
 
 
 
 
 
m_InstPerClass 
 
double[]  m_InstPerClass  
 
 InstPerClass
 
 
 
 

 
   
 
 
  
 Class  weka.classifiers.meta.GridSearch  extends  RandomizableSingleClassifierEnhancer  implements Serializable   
 
 

 
 serialVersionUID:  -3034773968581595348L

 
   
 
 
  
 Serialized Fields   
 
 

 
m_Filter 
 
 Filter   m_Filter  
 
 the Filter
 
 
 
 
 
 
m_BestFilter 
 
 Filter   m_BestFilter  
 
 the Filter with the best setup
 
 
 
 
 
 
m_BestClassifier 
 
 Classifier   m_BestClassifier  
 
 the Classifier with the best setup
 
 
 
 
 
 
m_Values 
 
weka.classifiers.meta.GridSearch.PointDouble  m_Values  
 
 the best values
 
 
 
 
 
 
m_Evaluation 
 
int  m_Evaluation  
 
 the type of evaluation
 
 
 
 
 
 
m_Y_Property 
 
java.lang.String  m_Y_Property  
 
 the Y option to work on (without leading dash, preceding 'classifier.' 
 means to set the option for the classifier 'filter.' for the filter)
 
 
 
 
 
 
m_Y_Min 
 
double  m_Y_Min  
 
 the minimum of Y
 
 
 
 
 
 
m_Y_Max 
 
double  m_Y_Max  
 
 the maximum of Y
 
 
 
 
 
 
m_Y_Step 
 
double  m_Y_Step  
 
 the step size of Y
 
 
 
 
 
 
m_Y_Base 
 
double  m_Y_Base  
 
 the base for Y
 
 
 
 
 
 
m_Y_Expression 
 
java.lang.String  m_Y_Expression  
 
 The expression for the Y property. Available parameters for the
 expression:
  
    BASE 
    FROM (= min) 
    TO (= max) 
    STEP 
    I - the current value (from 'from' to 'to' with stepsize 'step') 
  
 
 
  See Also:    MathematicalExpression  , 
  MathExpression   
 
 
 
m_X_Property 
 
java.lang.String  m_X_Property  
 
 the X option to work on (without leading dash, preceding 'classifier.' 
 means to set the option for the classifier 'filter.' for the filter)
 
 
 
 
 
 
m_X_Min 
 
double  m_X_Min  
 
 the minimum of X
 
 
 
 
 
 
m_X_Max 
 
double  m_X_Max  
 
 the maximum of X
 
 
 
 
 
 
m_X_Step 
 
double  m_X_Step  
 
 the step size of
 
 
 
 
 
 
m_X_Base 
 
double  m_X_Base  
 
 the base for
 
 
 
 
 
 
m_X_Expression 
 
java.lang.String  m_X_Expression  
 
 The expression for the X property. Available parameters for the
 expression:
  
    BASE 
    FROM (= min) 
    TO (= max) 
    STEP 
    I - the current value (from 'from' to 'to' with stepsize 'step') 
  
 
 
  See Also:    MathematicalExpression  , 
  MathExpression   
 
 
 
m_GridIsExtendable 
 
boolean  m_GridIsExtendable  
 
 whether the grid can be extended
 
 
 
 
 
 
m_MaxGridExtensions 
 
int  m_MaxGridExtensions  
 
 maximum number of grid extensions (-1 means unlimited)
 
 
 
 
 
 
m_GridExtensionsPerformed 
 
int  m_GridExtensionsPerformed  
 
 the number of extensions performed
 
 
 
 
 
 
m_SampleSize 
 
double  m_SampleSize  
 
 the sample size to search the initial grid with
 
 
 
 
 
 
m_Traversal 
 
int  m_Traversal  
 
 the traversal
 
 
 
 
 
 
m_LogFile 
 
java.io.File  m_LogFile  
 
 the log file to use
 
 
 
 
 
 
m_Grid 
 
weka.classifiers.meta.GridSearch.Grid  m_Grid  
 
 the value-pairs grid
 
 
 
 
 
 
m_Data 
 
 Instances   m_Data  
 
 the training data
 
 
 
 
 
 
m_Cache 
 
weka.classifiers.meta.GridSearch.PerformanceCache  m_Cache  
 
 the cache for points in the grid that got calculated
 
 
 
 
 
 
m_UniformPerformance 
 
boolean  m_UniformPerformance  
 
 whether all performances in the grid are the same
 
 
 
 

 
   
 
 
  
 Class weka.classifiers.meta.GridSearch.Grid extends java.lang.Object implements Serializable   
 
 

 
 serialVersionUID:  7290732613611243139L

 
   
 
 
  
 Serialized Fields   
 
 

 
m_MinX 
 
double  m_MinX  
 
 the minimum on the X axis
 
 
 
 
 
 
m_MaxX 
 
double  m_MaxX  
 
 the maximum on the X axis
 
 
 
 
 
 
m_StepX 
 
double  m_StepX  
 
 the step size for the X axis
 
 
 
 
 
 
m_LabelX 
 
java.lang.String  m_LabelX  
 
 the label for the X axis
 
 
 
 
 
 
m_MinY 
 
double  m_MinY  
 
 the minimum on the Y axis
 
 
 
 
 
 
m_MaxY 
 
double  m_MaxY  
 
 the maximum on the Y axis
 
 
 
 
 
 
m_StepY 
 
double  m_StepY  
 
 the step size for the Y axis
 
 
 
 
 
 
m_LabelY 
 
java.lang.String  m_LabelY  
 
 the label for the Y axis
 
 
 
 
 
 
m_Width 
 
int  m_Width  
 
 the number of points on the X axis
 
 
 
 
 
 
m_Height 
 
int  m_Height  
 
 the number of points on the Y axis
 
 
 
 

 
   
 
 
  
 Class weka.classifiers.meta.GridSearch.Performance extends java.lang.Object implements Serializable   
 
 

 
 serialVersionUID:  -4374706475277588755L

 
   
 
 
  
 Serialized Fields   
 
 

 
m_Values 
 
weka.classifiers.meta.GridSearch.PointDouble  m_Values  
 
 the value pair the classifier was built with
 
 
 
 
 
 
m_CC 
 
double  m_CC  
 
 the Correlation coefficient
 
 
 
 
 
 
m_RMSE 
 
double  m_RMSE  
 
 the Root mean squared error
 
 
 
 
 
 
m_RRSE 
 
double  m_RRSE  
 
 the Root relative squared error
 
 
 
 
 
 
m_MAE 
 
double  m_MAE  
 
 the Mean absolute error
 
 
 
 
 
 
m_RAE 
 
double  m_RAE  
 
 the Relative absolute error
 
 
 
 
 
 
m_ACC 
 
double  m_ACC  
 
 the Accuracy
 
 
 
 
 
 
m_Kappa 
 
double  m_Kappa  
 
 the kappa value
 
 
 
 

 
   
 
 
  
 Class weka.classifiers.meta.GridSearch.PerformanceCache extends java.lang.Object implements Serializable   
 
 

 
 serialVersionUID:  5838863230451530252L

 
   
 
 
  
 Serialized Fields   
 
 

 
m_Cache 
 
java.util.Hashtable<K,V>  m_Cache  
 
 the cache for points in the grid that got calculated
 
 
 
 

 
   
 
 
  
 Class weka.classifiers.meta.GridSearch.PerformanceComparator extends java.lang.Object implements Serializable   
 
 

 
 serialVersionUID:  6507592831825393847L

 
   
 
 
  
 Serialized Fields   
 
 

 
m_Evaluation 
 
int  m_Evaluation  
 
 the performance measure to use for comparison
 
 
  See Also:    GridSearch.TAGS_EVALUATION   
 

 
   
 
 
  
 Class weka.classifiers.meta.GridSearch.PerformanceTable extends java.lang.Object implements Serializable   
 
 

 
 serialVersionUID:  5486491313460338379L

 
   
 
 
  
 Serialized Fields   
 
 

 
m_Grid 
 
weka.classifiers.meta.GridSearch.Grid  m_Grid  
 
 the corresponding grid
 
 
 
 
 
 
m_Performances 
 
java.util.Vector<E>  m_Performances  
 
 the performances
 
 
 
 
 
 
m_Type 
 
int  m_Type  
 
 the type of performance the table was generated for
 
 
 
 
 
 
m_Table 
 
double[][]  m_Table  
 
 the table with the values
 
 
 
 
 
 
m_Min 
 
double  m_Min  
 
 the minimum performance
 
 
 
 
 
 
m_Max 
 
double  m_Max  
 
 the maximum performance
 
 
 
 

 
   
 
 
  
 Class weka.classifiers.meta.GridSearch.PointDouble extends java.awt.geom.Point2D.Double implements Serializable   
 
 

 
 serialVersionUID:  7151661776161898119L

 

 
   
 
 
  
 Class weka.classifiers.meta.GridSearch.PointInt extends java.awt.Point implements Serializable   
 
 

 
 serialVersionUID:  -5900415163698021618L

 

 
   
 
 
  
 Class  weka.classifiers.meta.LogitBoost  extends  RandomizableIteratedSingleClassifierEnhancer  implements Serializable   
 
 

 
 serialVersionUID:  -3905660358715833753L

 
   
 
 
  
 Serialized Fields   
 
 

 
m_Classifiers 
 
 Classifier [][]  m_Classifiers  
 
 Array for storing the generated base classifiers. 
   Note: we are hiding the variable from IteratedSingleClassifierEnhancer
 
 
 
 
 
 
m_NumClasses 
 
int  m_NumClasses  
 
 The number of classes
 
 
 
 
 
 
m_NumGenerated 
 
int  m_NumGenerated  
 
 The number of successfully generated base classifiers.
 
 
 
 
 
 
m_NumFolds 
 
int  m_NumFolds  
 
 The number of folds for the internal cross-validation.
 
 
 
 
 
 
m_NumRuns 
 
int  m_NumRuns  
 
 The number of runs for the internal cross-validation.
 
 
 
 
 
 
m_WeightThreshold 
 
int  m_WeightThreshold  
 
 Weight thresholding. The percentage of weight mass used in training
 
 
 
 
 
 
m_NumericClassData 
 
 Instances   m_NumericClassData  
 
 Dummy dataset with a numeric class
 
 
 
 
 
 
m_ClassAttribute 
 
 Attribute   m_ClassAttribute  
 
 The actual class attribute (for getting class names)
 
 
 
 
 
 
m_UseResampling 
 
boolean  m_UseResampling  
 
 Use boosting with reweighting?
 
 
 
 
 
 
m_Precision 
 
double  m_Precision  
 
 The threshold on the improvement of the likelihood
 
 
 
 
 
 
m_Shrinkage 
 
double  m_Shrinkage  
 
 The value of the shrinkage parameter
 
 
 
 
 
 
m_RandomInstance 
 
java.util.Random  m_RandomInstance  
 
 The random number generator used
 
 
 
 
 
 
m_Offset 
 
double  m_Offset  
 
 The value by which the actual target value for the
      true class is offset.
 
 
 
 
 
 
m_ZeroR 
 
 Classifier   m_ZeroR  
 
 a ZeroR model in case no model can be built from the data
 
 
 
 

 
   
 
 
  
 Class  weka.classifiers.meta.MetaCost  extends  RandomizableSingleClassifierEnhancer  implements Serializable   
 
 

 
 serialVersionUID:  1205317833344726855L

 
   
 
 
  
 Serialized Fields   
 
 

 
m_MatrixSource 
 
int  m_MatrixSource  
 
 Indicates the current cost matrix source
 
 
 
 
 
 
m_OnDemandDirectory 
 
java.io.File  m_OnDemandDirectory  
 
 The directory used when loading cost files on demand, null indicates
 current directory
 
 
 
 
 
 
m_CostFile 
 
java.lang.String  m_CostFile  
 
 The name of the cost file, for command line options
 
 
 
 
 
 
m_CostMatrix 
 
 CostMatrix   m_CostMatrix  
 
 The cost matrix
 
 
 
 
 
 
m_NumIterations 
 
int  m_NumIterations  
 
 The number of iterations.
 
 
 
 
 
 
m_BagSizePercent 
 
int  m_BagSizePercent  
 
 The size of each bag sample, as a percentage of the training size
 
 
 
 

 
   
 
 
  
 Class  weka.classifiers.meta.MultiBoostAB  extends  AdaBoostM1  implements Serializable   
 
 

 
 serialVersionUID:  -6681619178187935148L

 
   
 
 
  
 Serialized Fields   
 
 

 
m_NumSubCmtys 
 
int  m_NumSubCmtys  
 
 The number of sub-committees to use
 
 
 
 
 
 
m_Random 
 
java.util.Random  m_Random  
 
 Random number generator
 
 
 
 

 
   
 
 
  
 Class  weka.classifiers.meta.MultiClassClassifier  extends  RandomizableSingleClassifierEnhancer  implements Serializable   
 
 

 
 serialVersionUID:  -3879602011542849141L

 
   
 
 
  
 Serialized Fields   
 
 

 
m_Classifiers 
 
 Classifier []  m_Classifiers  
 
 The classifiers.
 
 
 
 
 
 
m_pairwiseCoupling 
 
boolean  m_pairwiseCoupling  
 
 Use pairwise coupling with 1-vs-1
 
 
 
 
 
 
m_SumOfWeights 
 
double[]  m_SumOfWeights  
 
 Needed for pairwise coupling
 
 
 
 
 
 
m_ClassFilters 
 
 Filter []  m_ClassFilters  
 
 The filters used to transform the class.
 
 
 
 
 
 
m_ZeroR 
 
 ZeroR   m_ZeroR  
 
 ZeroR classifier for when all base classifier return zero probability.
 
 
 
 
 
 
m_ClassAttribute 
 
 Attribute   m_ClassAttribute  
 
 Internal copy of the class attribute for output purposes
 
 
 
 
 
 
m_TwoClassDataset 
 
 Instances   m_TwoClassDataset  
 
 A transformed dataset header used by the  1-against-1 method
 
 
 
 
 
 
m_RandomWidthFactor 
 
double  m_RandomWidthFactor  
 
 The multiplier when generating random codes. Will generate
 numClasses * m_RandomWidthFactor codes
 
 
 
 
 
 
m_Method 
 
int  m_Method  
 
 The multiclass method to use
 
 
 
 

 
   
 
 
  
 Class  weka.classifiers.meta.MultiScheme  extends  RandomizableMultipleClassifiersCombiner  implements Serializable   
 
 

 
 serialVersionUID:  5710744346128957520L

 
   
 
 
  
 Serialized Fields   
 
 

 
m_Classifier 
 
 Classifier   m_Classifier  
 
 The classifier that had the best performance on training data.
 
 
 
 
 
 
m_ClassifierIndex 
 
int  m_ClassifierIndex  
 
 The index into the vector for the selected scheme
 
 
 
 
 
 
m_NumXValFolds 
 
int  m_NumXValFolds  
 
 Number of folds to use for cross validation (0 means use training
 error for selection)
 
 
 
 

 
   
 
 
  
 Class  weka.classifiers.meta.OrdinalClassClassifier  extends  SingleClassifierEnhancer  implements Serializable   
 
 

 
 serialVersionUID:  -3461971774059603636L

 
   
 
 
  
 Serialized Fields   
 
 

 
m_Classifiers 
 
 Classifier []  m_Classifiers  
 
 The classifiers. (One for each class.)
 
 
 
 
 
 
m_ClassFilters 
 
 MakeIndicator []  m_ClassFilters  
 
 The filters used to transform the class.
 
 
 
 
 
 
m_ZeroR 
 
 ZeroR   m_ZeroR  
 
 ZeroR classifier for when all base classifier return zero probability.
 
 
 
 

 
   
 
 
  
 Class  weka.classifiers.meta.RacedIncrementalLogitBoost  extends  RandomizableSingleClassifierEnhancer  implements Serializable   
 
 

 
 serialVersionUID:  908598343772170052L

 
   
 
 
  
 Serialized Fields   
 
 

 
m_committees 
 
 FastVector   m_committees  
 
 The committees
 
 
 
 
 
 
m_PruningType 
 
int  m_PruningType  
 
 The pruning type used
 
 
 
 
 
 
m_UseResampling 
 
boolean  m_UseResampling  
 
 Whether to use resampling
 
 
 
 
 
 
m_NumClasses 
 
int  m_NumClasses  
 
 The number of classes
 
 
 
 
 
 
m_NumericClassData 
 
 Instances   m_NumericClassData  
 
 Dummy dataset with a numeric class
 
 
 
 
 
 
m_ClassAttribute 
 
 Attribute   m_ClassAttribute  
 
 The actual class attribute (for getting class names)
 
 
 
 
 
 
m_minChunkSize 
 
int  m_minChunkSize  
 
 The minimum chunk size used for training
 
 
 
 
 
 
m_maxChunkSize 
 
int  m_maxChunkSize  
 
 The maimum chunk size used for training
 
 
 
 
 
 
m_validationChunkSize 
 
int  m_validationChunkSize  
 
 The size of the validation set
 
 
 
 
 
 
m_numInstancesConsumed 
 
int  m_numInstancesConsumed  
 
 The number of instances consumed
 
 
 
 
 
 
m_validationSet 
 
 Instances   m_validationSet  
 
 The instances used for validation
 
 
 
 
 
 
m_currentSet 
 
 Instances   m_currentSet  
 
 The instances currently in memory for training
 
 
 
 
 
 
m_bestCommittee 
 
weka.classifiers.meta.RacedIncrementalLogitBoost.Committee  m_bestCommittee  
 
 The current best committee
 
 
 
 
 
 
m_zeroR 
 
 ZeroR   m_zeroR  
 
 The default scheme used when committees aren't ready
 
 
 
 
 
 
m_validationSetChanged 
 
boolean  m_validationSetChanged  
 
 Whether the validation set has recently been changed
 
 
 
 
 
 
m_maxBatchSizeRequired 
 
int  m_maxBatchSizeRequired  
 
 The maximum number of instances required for processing
 
 
 
 
 
 
m_RandomInstance 
 
java.util.Random  m_RandomInstance  
 
 The random number generator used
 
 
 
 

 
   
 
 
  
 Class weka.classifiers.meta.RacedIncrementalLogitBoost.Committee extends java.lang.Object implements Serializable   
 
 

 
 serialVersionUID:  5559880306684082199L

 
   
 
 
  
 Serialized Fields   
 
 

 
m_chunkSize 
 
int  m_chunkSize  
 
 
 
 
 
 
m_instancesConsumed 
 
int  m_instancesConsumed  
 
 number eaten from m_currentSet
 
 
 
 
 
 
m_models 
 
 FastVector   m_models  
 
 
 
 
 
 
m_lastValidationError 
 
double  m_lastValidationError  
 
 
 
 
 
 
m_lastLogLikelihood 
 
double  m_lastLogLikelihood  
 
 
 
 
 
 
m_modelHasChanged 
 
boolean  m_modelHasChanged  
 
 
 
 
 
 
m_modelHasChangedLL 
 
boolean  m_modelHasChangedLL  
 
 
 
 
 
 
m_validationFs 
 
double[][]  m_validationFs  
 
 
 
 
 
 
m_newValidationFs 
 
double[][]  m_newValidationFs  
 
 
 
 

 
   
 
 
  
 Class  weka.classifiers.meta.RandomCommittee  extends  RandomizableIteratedSingleClassifierEnhancer  implements Serializable   
 
 

 
 serialVersionUID:  -9204394360557300092L

 

 
   
 
 
  
 Class  weka.classifiers.meta.RandomSubSpace  extends  RandomizableIteratedSingleClassifierEnhancer  implements Serializable   
 
 

 
 serialVersionUID:  1278172513912424947L

 
   
 
 
  
 Serialized Fields   
 
 

 
m_SubSpaceSize 
 
double  m_SubSpaceSize  
 
 The size of each bag sample, as a percentage of the training size
 
 
 
 
 
 
m_ZeroR 
 
 Classifier   m_ZeroR  
 
 a ZeroR model in case no model can be built from the data
 
 
 
 

 
   
 
 
  
 Class  weka.classifiers.meta.RegressionByDiscretization  extends  SingleClassifierEnhancer  implements Serializable   
 
 

 
 serialVersionUID:  5066426153134050378L

 
   
 
 
  
 Serialized Fields   
 
 

 
m_Discretizer 
 
 Discretize   m_Discretizer  
 
 The discretization filter.
 
 
 
 
 
 
m_NumBins 
 
int  m_NumBins  
 
 The number of discretization intervals.
 
 
 
 
 
 
m_ClassMeans 
 
double[]  m_ClassMeans  
 
 The mean values for each Discretized class interval.
 
 
 
 
 
 
m_DeleteEmptyBins 
 
boolean  m_DeleteEmptyBins  
 
 Whether to delete empty intervals.
 
 
 
 
 
 
m_DiscretizedHeader 
 
 Instances   m_DiscretizedHeader  
 
 Header of discretized data.
 
 
 
 
 
 
m_UseEqualFrequency 
 
boolean  m_UseEqualFrequency  
 
 Use equal-frequency binning
 
 
 
 

 
   
 
 
  
 Class  weka.classifiers.meta.RotationForest  extends  RandomizableIteratedSingleClassifierEnhancer  implements Serializable   
 
 

 
 serialVersionUID:  -3255631880798499936L

 
   
 
 
  
 Serialized Fields   
 
 

 
m_MinGroup 
 
int  m_MinGroup  
 
 The minimum size of a group
 
 
 
 
 
 
m_MaxGroup 
 
int  m_MaxGroup  
 
 The maximum size of a group
 
 
 
 
 
 
m_NumberOfGroups 
 
boolean  m_NumberOfGroups  
 
 Whether minGroup and maxGroup refer to the number of groups or their 
 size
 
 
 
 
 
 
m_RemovedPercentage 
 
int  m_RemovedPercentage  
 
 The percentage of instances to be removed
 
 
 
 
 
 
m_Groups 
 
int[][][]  m_Groups  
 
 The attributes of each group
 
 
 
 
 
 
m_ProjectionFilter 
 
 Filter   m_ProjectionFilter  
 
 The type of projection filter
 
 
 
 
 
 
m_ProjectionFilters 
 
 Filter [][]  m_ProjectionFilters  
 
 The projection filters
 
 
 
 
 
 
m_Headers 
 
 Instances []  m_Headers  
 
 Headers of the transformed dataset
 
 
 
 
 
 
m_ReducedHeaders 
 
 Instances [][]  m_ReducedHeaders  
 
 Headers of the reduced datasets
 
 
 
 
 
 
m_RemoveUseless 
 
 RemoveUseless   m_RemoveUseless  
 
 Filter that remove useless attributes
 
 
 
 
 
 
m_Normalize 
 
 Normalize   m_Normalize  
 
 Filter that normalized the attributes
 
 
 
 

 
   
 
 
  
 Class  weka.classifiers.meta.Stacking  extends  RandomizableMultipleClassifiersCombiner  implements Serializable   
 
 

 
 serialVersionUID:  5134738557155845452L

 
   
 
 
  
 Serialized Fields   
 
 

 
m_MetaClassifier 
 
 Classifier   m_MetaClassifier  
 
 The meta classifier
 
 
 
 
 
 
m_MetaFormat 
 
 Instances   m_MetaFormat  
 
 Format for meta data
 
 
 
 
 
 
m_BaseFormat 
 
 Instances   m_BaseFormat  
 
 Format for base data
 
 
 
 
 
 
m_NumFolds 
 
int  m_NumFolds  
 
 Set the number of folds for the cross-validation
 
 
 
 

 
   
 
 
  
 Class  weka.classifiers.meta.StackingC  extends  Stacking  implements Serializable   
 
 

 
 serialVersionUID:  -6717545616603725198L

 
   
 
 
  
 Serialized Fields   
 
 

 
m_MetaClassifiers 
 
 Classifier []  m_MetaClassifiers  
 
 The meta classifiers (one for each class, like in ClassificationViaRegression)
 
 
 
 
 
 
m_attrFilter 
 
 Remove   m_attrFilter  
 
 Filter to transform metaData - Remove
 
 
 
 
 
 
m_makeIndicatorFilter 
 
 MakeIndicator   m_makeIndicatorFilter  
 
 Filter to transform metaData - MakeIndicator
 
 
 
 

 
   
 
 
  
 Class  weka.classifiers.meta.ThresholdSelector  extends  RandomizableSingleClassifierEnhancer  implements Serializable   
 
 

 
 serialVersionUID:  -1795038053239867444L

 
   
 
 
  
 Serialized Fields   
 
 

 
m_HighThreshold 
 
double  m_HighThreshold  
 
 The upper threshold used as the basis of correction
 
 
 
 
 
 
m_LowThreshold 
 
double  m_LowThreshold  
 
 The lower threshold used as the basis of correction
 
 
 
 
 
 
m_BestThreshold 
 
double  m_BestThreshold  
 
 The threshold that lead to the best performance
 
 
 
 
 
 
m_BestValue 
 
double  m_BestValue  
 
 The best value that has been observed
 
 
 
 
 
 
m_NumXValFolds 
 
int  m_NumXValFolds  
 
 The number of folds used in cross-validation
 
 
 
 
 
 
m_DesignatedClass 
 
int  m_DesignatedClass  
 
 Designated class value, determined during building
 
 
 
 
 
 
m_ClassMode 
 
int  m_ClassMode  
 
 Method to determine which class to optimize for
 
 
 
 
 
 
m_EvalMode 
 
int  m_EvalMode  
 
 The evaluation mode
 
 
 
 
 
 
m_RangeMode 
 
int  m_RangeMode  
 
 The range correction mode
 
 
 
 
 
 
m_nMeasure 
 
int  m_nMeasure  
 
 evaluation measure used for determining threshold
 
 
 
 
 
 
m_manualThreshold 
 
boolean  m_manualThreshold  
 
 True if a manually set threshold is being used
 
 
 
 
 
 
m_manualThresholdValue 
 
double  m_manualThresholdValue  
 
 -1 = not used by default
 
 
 
 

 
   
 
 
  
 Class  weka.classifiers.meta.Vote  extends  RandomizableMultipleClassifiersCombiner  implements Serializable   
 
 

 
 serialVersionUID:  -637891196294399624L

 
   
 
 
  
 Serialized Fields   
 
 

 
m_CombinationRule 
 
int  m_CombinationRule  
 
 Combination Rule variable
 
 
 
 
 
 
m_Random 
 
java.util.Random  m_Random  
 
 the random number generator used for breaking ties in majority voting
 
 
  See Also:    Vote.distributionForInstanceMajorityVoting(Instance)   
 
 

 
 
  
 Package   weka.classifiers.meta.nestedDichotomies   
 
 

 
   
 
 
  
 Class  weka.classifiers.meta.nestedDichotomies.ClassBalancedND  extends  RandomizableSingleClassifierEnhancer  implements Serializable   
 
 

 
 serialVersionUID:  5944063630650811903L

 
   
 
 
  
 Serialized Fields   
 
 

 
m_FilteredClassifier 
 
 FilteredClassifier   m_FilteredClassifier  
 
 The filtered classifier in which the base classifier is wrapped.
 
 
 
 
 
 
m_classifiers 
 
java.util.Hashtable<K,V>  m_classifiers  
 
 The hashtable for this node.
 
 
 
 
 
 
m_FirstSuccessor 
 
 ClassBalancedND   m_FirstSuccessor  
 
 The first successor
 
 
 
 
 
 
m_SecondSuccessor 
 
 ClassBalancedND   m_SecondSuccessor  
 
 The second successor
 
 
 
 
 
 
m_Range 
 
 Range   m_Range  
 
 The classes that are grouped together at the current node
 
 
 
 
 
 
m_hashtablegiven 
 
boolean  m_hashtablegiven  
 
 Is Hashtable given from END?
 
 
 
 

 
   
 
 
  
 Class  weka.classifiers.meta.nestedDichotomies.DataNearBalancedND  extends  RandomizableSingleClassifierEnhancer  implements Serializable   
 
 

 
 serialVersionUID:  5117477294209496368L

 
   
 
 
  
 Serialized Fields   
 
 

 
m_FilteredClassifier 
 
 FilteredClassifier   m_FilteredClassifier  
 
 The filtered classifier in which the base classifier is wrapped.
 
 
 
 
 
 
m_classifiers 
 
java.util.Hashtable<K,V>  m_classifiers  
 
 The hashtable for this node.
 
 
 
 
 
 
m_FirstSuccessor 
 
 DataNearBalancedND   m_FirstSuccessor  
 
 The first successor
 
 
 
 
 
 
m_SecondSuccessor 
 
 DataNearBalancedND   m_SecondSuccessor  
 
 The second successor
 
 
 
 
 
 
m_Range 
 
 Range   m_Range  
 
 The classes that are grouped together at the current node
 
 
 
 
 
 
m_hashtablegiven 
 
boolean  m_hashtablegiven  
 
 Is Hashtable given from END?
 
 
 
 

 
   
 
 
  
 Class  weka.classifiers.meta.nestedDichotomies.ND  extends  RandomizableSingleClassifierEnhancer  implements Serializable   
 
 

 
 serialVersionUID:  -6355893369855683820L

 
   
 
 
  
 Serialized Fields   
 
 

 
m_ndtree 
 
weka.classifiers.meta.nestedDichotomies.ND.NDTree  m_ndtree  
 
 The tree of classes
 
 
 
 
 
 
m_classifiers 
 
java.util.Hashtable<K,V>  m_classifiers  
 
 The hashtable containing all the classifiers
 
 
 
 
 
 
m_hashtablegiven 
 
boolean  m_hashtablegiven  
 
 Is Hashtable given from END?
 
 
 
 

 
   
 
 
  
 Class weka.classifiers.meta.nestedDichotomies.ND.NDTree extends java.lang.Object implements Serializable   
 
 

 
 serialVersionUID:  4284655952754474880L

 
   
 
 
  
 Serialized Fields   
 
 

 
m_indices 
 
 FastVector   m_indices  
 
 The indices associated with this node
 
 
 
 
 
 
m_parent 
 
weka.classifiers.meta.nestedDichotomies.ND.NDTree  m_parent  
 
 The parent
 
 
 
 
 
 
m_left 
 
weka.classifiers.meta.nestedDichotomies.ND.NDTree  m_left  
 
 The left successor
 
 
 
 
 
 
m_right 
 
weka.classifiers.meta.nestedDichotomies.ND.NDTree  m_right  
 
 The right successor
 
 
 
 
 

 
 
  
 Package   weka.classifiers.mi   
 
 

 
   
 
 
  
 Class  weka.classifiers.mi.CitationKNN  extends  Classifier  implements Serializable   
 
 

 
 serialVersionUID:  -8435377743874094852L

 
   
 
 
  
 Serialized Fields   
 
 

 
m_ClassIndex 
 
int  m_ClassIndex  
 
 The index of the class attribute
 
 
 
 
 
 
m_NumClasses 
 
int  m_NumClasses  
 
 The number of the class labels
 
 
 
 
 
 
m_IdIndex 
 
int  m_IdIndex  
 
 
 
 
 
 
m_Debug 
 
boolean  m_Debug  
 
 Debugging output
 
 
 
 
 
 
m_Classes 
 
int[]  m_Classes  
 
 Class labels for each bag
 
 
 
 
 
 
m_Attributes 
 
 Instances   m_Attributes  
 
 attribute name structure of the relational attribute
 
 
 
 
 
 
m_NumReferences 
 
int  m_NumReferences  
 
 Number of references
 
 
 
 
 
 
m_NumCiters 
 
int  m_NumCiters  
 
 Number of citers
 
 
 
 
 
 
m_TrainBags 
 
 Instances   m_TrainBags  
 
 Training bags
 
 
 
 
 
 
m_CNNDebug 
 
boolean  m_CNNDebug  
 
 Different debugging output
 
 
 
 
 
 
m_CitersDebug 
 
boolean  m_CitersDebug  
 
 
 
 
 
 
m_ReferencesDebug 
 
boolean  m_ReferencesDebug  
 
 
 
 
 
 
m_HDistanceDebug 
 
boolean  m_HDistanceDebug  
 
 
 
 
 
 
m_NeighborListDebug 
 
boolean  m_NeighborListDebug  
 
 
 
 
 
 
m_CNN 
 
weka.classifiers.mi.CitationKNN.NeighborList[]  m_CNN  
 
 C nearest neighbors considering all the bags
 
 
 
 
 
 
m_Citers 
 
int[]  m_Citers  
 
 C nearest citers
 
 
 
 
 
 
m_References 
 
int[]  m_References  
 
 R nearest references
 
 
 
 
 
 
m_HDRank 
 
int  m_HDRank  
 
 Rank associated to the Hausdorff distance
 
 
 
 
 
 
m_Diffs 
 
double[]  m_Diffs  
 
 Normalization of the euclidean distance
 
 
 
 
 
 
m_Min 
 
double[]  m_Min  
 
 
 
 
 
 
m_MinNorm 
 
double  m_MinNorm  
 
 
 
 
 
 
m_Max 
 
double[]  m_Max  
 
 
 
 
 
 
m_MaxNorm 
 
double  m_MaxNorm  
 
 
 
 

 
   
 
 
  
 Class  weka.classifiers.mi.MDD  extends  Classifier  implements Serializable   
 
 

 
 serialVersionUID:  -7273119490545290581L

 
   
 
 
  
 Serialized Fields   
 
 

 
m_ClassIndex 
 
int  m_ClassIndex  
 
 The index of the class attribute
 
 
 
 
 
 
m_Par 
 
double[]  m_Par  
 
 
 
 
 
 
m_NumClasses 
 
int  m_NumClasses  
 
 The number of the class labels
 
 
 
 
 
 
m_Classes 
 
int[]  m_Classes  
 
 Class labels for each bag
 
 
 
 
 
 
m_Data 
 
double[][][]  m_Data  
 
 MI data
 
 
 
 
 
 
m_Attributes 
 
 Instances   m_Attributes  
 
 All attribute names
 
 
 
 
 
 
m_Filter 
 
 Filter   m_Filter  
 
 The filter used to standardize/normalize all values.
 
 
 
 
 
 
m_filterType 
 
int  m_filterType  
 
 Whether to normalize/standardize/neither, default:standardize
 
 
 
 
 
 
m_Missing 
 
 ReplaceMissingValues   m_Missing  
 
 The filter used to get rid of missing values.
 
 
 
 

 
   
 
 
  
 Class  weka.classifiers.mi.MIBoost  extends  SingleClassifierEnhancer  implements Serializable   
 
 

 
 serialVersionUID:  -3808427225599279539L

 
   
 
 
  
 Serialized Fields   
 
 

 
m_Models 
 
 Classifier []  m_Models  
 
 the models for the iterations
 
 
 
 
 
 
m_NumClasses 
 
int  m_NumClasses  
 
 The number of the class labels
 
 
 
 
 
 
m_Classes 
 
int[]  m_Classes  
 
 Class labels for each bag
 
 
 
 
 
 
m_Attributes 
 
 Instances   m_Attributes  
 
 attributes name for the new dataset used to build the model
 
 
 
 
 
 
m_NumIterations 
 
int  m_NumIterations  
 
 Number of iterations
 
 
 
 
 
 
m_Beta 
 
double[]  m_Beta  
 
 Voting weights of models
 
 
 
 
 
 
m_MaxIterations 
 
int  m_MaxIterations  
 
 the maximum number of boost iterations
 
 
 
 
 
 
m_DiscretizeBin 
 
int  m_DiscretizeBin  
 
 the number of discretization bins
 
 
 
 
 
 
m_Filter 
 
 Discretize   m_Filter  
 
 filter used for discretization
 
 
 
 
 
 
m_ConvertToSI 
 
 MultiInstanceToPropositional   m_ConvertToSI  
 
 filter used to convert the MI dataset into single-instance dataset
 
 
 
 

 
   
 
 
  
 Class  weka.classifiers.mi.MIDD  extends  Classifier  implements Serializable   
 
 

 
 serialVersionUID:  4263507733600536168L

 
   
 
 
  
 Serialized Fields   
 
 

 
m_ClassIndex 
 
int  m_ClassIndex  
 
 The index of the class attribute
 
 
 
 
 
 
m_Par 
 
double[]  m_Par  
 
 
 
 
 
 
m_NumClasses 
 
int  m_NumClasses  
 
 The number of the class labels
 
 
 
 
 
 
m_Classes 
 
int[]  m_Classes  
 
 Class labels for each bag
 
 
 
 
 
 
m_Data 
 
double[][][]  m_Data  
 
 MI data
 
 
 
 
 
 
m_Attributes 
 
 Instances   m_Attributes  
 
 All attribute names
 
 
 
 
 
 
m_Filter 
 
 Filter   m_Filter  
 
 The filter used to standardize/normalize all values.
 
 
 
 
 
 
m_filterType 
 
int  m_filterType  
 
 Whether to normalize/standardize/neither, default:standardize
 
 
 
 
 
 
m_Missing 
 
 ReplaceMissingValues   m_Missing  
 
 The filter used to get rid of missing values.
 
 
 
 

 
   
 
 
  
 Class  weka.classifiers.mi.MIEMDD  extends  RandomizableClassifier  implements Serializable   
 
 

 
 serialVersionUID:  3899547154866223734L

 
   
 
 
  
 Serialized Fields   
 
 

 
m_ClassIndex 
 
int  m_ClassIndex  
 
 The index of the class attribute
 
 
 
 
 
 
m_Par 
 
double[]  m_Par  
 
 
 
 
 
 
m_NumClasses 
 
int  m_NumClasses  
 
 The number of the class labels
 
 
 
 
 
 
m_Classes 
 
int[]  m_Classes  
 
 Class labels for each bag
 
 
 
 
 
 
m_Data 
 
double[][][]  m_Data  
 
 MI data
 
 
 
 
 
 
m_Attributes 
 
 Instances   m_Attributes  
 
 All attribute names
 
 
 
 
 
 
m_emData 
 
double[][]  m_emData  
 
 MI data
 
 
 
 
 
 
m_Filter 
 
 Filter   m_Filter  
 
 The filter used to standardize/normalize all values.
 
 
 
 
 
 
m_filterType 
 
int  m_filterType  
 
 Whether to normalize/standardize/neither, default:standardize
 
 
 
 
 
 
m_Missing 
 
 ReplaceMissingValues   m_Missing  
 
 The filter used to get rid of missing values.
 
 
 
 

 
   
 
 
  
 Class  weka.classifiers.mi.MILR  extends  Classifier  implements Serializable   
 
 

 
 serialVersionUID:  1996101190172373826L

 
   
 
 
  
 Serialized Fields   
 
 

 
m_Par 
 
double[]  m_Par  
 
 
 
 
 
 
m_NumClasses 
 
int  m_NumClasses  
 
 The number of the class labels
 
 
 
 
 
 
m_Ridge 
 
double  m_Ridge  
 
 The ridge parameter.
 
 
 
 
 
 
m_Classes 
 
int[]  m_Classes  
 
 Class labels for each bag
 
 
 
 
 
 
m_Data 
 
double[][][]  m_Data  
 
 MI data
 
 
 
 
 
 
m_Attributes 
 
 Instances   m_Attributes  
 
 All attribute names
 
 
 
 
 
 
xMean 
 
double[]  xMean  
 
 
 
 
 
 
xSD 
 
double[]  xSD  
 
 
 
 
 
 
m_AlgorithmType 
 
int  m_AlgorithmType  
 
 the type of processing
 
 
 
 

 
   
 
 
  
 Class  weka.classifiers.mi.MINND  extends  Classifier  implements Serializable   
 
 

 
 serialVersionUID:  -4512599203273864994L

 
   
 
 
  
 Serialized Fields   
 
 

 
m_Neighbour 
 
int  m_Neighbour  
 
 The number of nearest neighbour for prediction
 
 
 
 
 
 
m_Mean 
 
double[][]  m_Mean  
 
 The mean for each attribute of each exemplar
 
 
 
 
 
 
m_Variance 
 
double[][]  m_Variance  
 
 The variance for each attribute of each exemplar
 
 
 
 
 
 
m_Dimension 
 
int  m_Dimension  
 
 The dimension of each exemplar, i.e. (numAttributes-2)
 
 
 
 
 
 
m_Attributes 
 
 Instances   m_Attributes  
 
 header info of the data
 
 
 
 
 
 
m_Class 
 
double[]  m_Class  
 
 The class label of each exemplar
 
 
 
 
 
 
m_NumClasses 
 
int  m_NumClasses  
 
 The number of class labels in the data
 
 
 
 
 
 
m_Weights 
 
double[]  m_Weights  
 
 The weight of each exemplar
 
 
 
 
 
 
m_Rate 
 
double  m_Rate  
 
 The learning rate in the gradient descent
 
 
 
 
 
 
m_MinArray 
 
double[]  m_MinArray  
 
 The minimum values for numeric attributes.
 
 
 
 
 
 
m_MaxArray 
 
double[]  m_MaxArray  
 
 The maximum values for numeric attributes.
 
 
 
 
 
 
m_STOP 
 
double  m_STOP  
 
 The stopping criteria of gradient descent
 
 
 
 
 
 
m_Change 
 
double[][]  m_Change  
 
 The weights that alter the dimnesion of each exemplar
 
 
 
 
 
 
m_NoiseM 
 
double[][]  m_NoiseM  
 
 The noise data of each exemplar
 
 
 
 
 
 
m_NoiseV 
 
double[][]  m_NoiseV  
 
 The noise data of each exemplar
 
 
 
 
 
 
m_ValidM 
 
double[][]  m_ValidM  
 
 The noise data of each exemplar
 
 
 
 
 
 
m_ValidV 
 
double[][]  m_ValidV  
 
 The noise data of each exemplar
 
 
 
 
 
 
m_Select 
 
int  m_Select  
 
 The number of nearest neighbour instances in the selection of noises 
    in the training data
 
 
 
 
 
 
m_Choose 
 
int  m_Choose  
 
 The number of nearest neighbour exemplars in the selection of noises 
    in the test data
 
 
 
 
 
 
m_Decay 
 
double  m_Decay  
 
 The decay rate of learning rate
 
 
 
 

 
   
 
 
  
 Class  weka.classifiers.mi.MIOptimalBall  extends  Classifier  implements Serializable   
 
 

 
 serialVersionUID:  -6465750129576777254L

 
   
 
 
  
 Serialized Fields   
 
 

 
m_Center 
 
double[]  m_Center  
 
 center of the optimal ball
 
 
 
 
 
 
m_Radius 
 
double  m_Radius  
 
 radius of the optimal ball
 
 
 
 
 
 
m_Distance 
 
double[][][]  m_Distance  
 
 the distances from each instance in a positive bag to each bag
 
 
 
 
 
 
m_Filter 
 
 Filter   m_Filter  
 
 The filter used to standardize/normalize all values.
 
 
 
 
 
 
m_filterType 
 
int  m_filterType  
 
 Whether to normalize/standardize/neither
 
 
 
 
 
 
m_ConvertToSI 
 
 MultiInstanceToPropositional   m_ConvertToSI  
 
 filter used to convert the MI dataset into single-instance dataset
 
 
 
 
 
 
m_ConvertToMI 
 
 PropositionalToMultiInstance   m_ConvertToMI  
 
 filter used to convert the single-instance dataset into MI dataset
 
 
 
 

 
   
 
 
  
 Class  weka.classifiers.mi.MISMO  extends  Classifier  implements Serializable   
 
 

 
 serialVersionUID:  -5834036950143719712L

 
   
 
 
  
 Serialized Fields   
 
 

 
m_classifiers 
 
weka.classifiers.mi.MISMO.BinaryMISMO[][]  m_classifiers  
 
 The binary classifier(s)
 
 
 
 
 
 
m_C 
 
double  m_C  
 
 The complexity parameter.
 
 
 
 
 
 
m_eps 
 
double  m_eps  
 
 Epsilon for rounding.
 
 
 
 
 
 
m_tol 
 
double  m_tol  
 
 Tolerance for accuracy of result.
 
 
 
 
 
 
m_filterType 
 
int  m_filterType  
 
 Whether to normalize/standardize/neither
 
 
 
 
 
 
m_minimax 
 
boolean  m_minimax  
 
 Use MIMinimax feature space?
 
 
 
 
 
 
m_NominalToBinary 
 
 NominalToBinary   m_NominalToBinary  
 
 The filter used to make attributes numeric.
 
 
 
 
 
 
m_Filter 
 
 Filter   m_Filter  
 
 The filter used to standardize/normalize all values.
 
 
 
 
 
 
m_Missing 
 
 ReplaceMissingValues   m_Missing  
 
 The filter used to get rid of missing values.
 
 
 
 
 
 
m_classIndex 
 
int  m_classIndex  
 
 The class index from the training data
 
 
 
 
 
 
m_classAttribute 
 
 Attribute   m_classAttribute  
 
 The class attribute
 
 
 
 
 
 
m_kernel 
 
 Kernel   m_kernel  
 
 Kernel to use
 
 
 
 
 
 
m_checksTurnedOff 
 
boolean  m_checksTurnedOff  
 
 Turn off all checks and conversions? Turning them off assumes
    that data is purely numeric, doesn't contain any missing values,
    and has a nominal class. Turning them off also means that
    no header information will be stored if the machine is linear. 
    Finally, it also assumes that no instance has a weight equal to 0.
 
 
 
 
 
 
m_fitLogisticModels 
 
boolean  m_fitLogisticModels  
 
 Whether logistic models are to be fit
 
 
 
 
 
 
m_numFolds 
 
int  m_numFolds  
 
 The number of folds for the internal cross-validation
 
 
 
 
 
 
m_randomSeed 
 
int  m_randomSeed  
 
 The random number seed
 
 
 
 

 
   
 
 
  
 Class weka.classifiers.mi.MISMO.BinaryMISMO extends java.lang.Object implements Serializable   
 
 

 
 serialVersionUID:  -7107082483475433531L

 
   
 
 
  
 Serialized Fields   
 
 

 
m_alpha 
 
double[]  m_alpha  
 
 The Lagrange multipliers.
 
 
 
 
 
 
m_b 
 
double  m_b  
 
 The thresholds.
 
 
 
 
 
 
m_bLow 
 
double  m_bLow  
 
 The thresholds.
 
 
 
 
 
 
m_bUp 
 
double  m_bUp  
 
 The thresholds.
 
 
 
 
 
 
m_iLow 
 
int  m_iLow  
 
 The indices for m_bLow and m_bUp
 
 
 
 
 
 
m_iUp 
 
int  m_iUp  
 
 The indices for m_bLow and m_bUp
 
 
 
 
 
 
m_data 
 
 Instances   m_data  
 
 The training data.
 
 
 
 
 
 
m_weights 
 
double[]  m_weights  
 
 Weight vector for linear machine.
 
 
 
 
 
 
m_sparseWeights 
 
double[]  m_sparseWeights  
 
 Variables to hold weight vector in sparse form.
      (To reduce storage requirements.)
 
 
 
 
 
 
m_sparseIndices 
 
int[]  m_sparseIndices  
 
 
 
 
 
 
m_kernel 
 
 Kernel   m_kernel  
 
 Kernel to use
 
 
 
 
 
 
m_class 
 
double[]  m_class  
 
 The transformed class values.
 
 
 
 
 
 
m_errors 
 
double[]  m_errors  
 
 The current set of errors for all non-bound examples.
 
 
 
 
 
 
m_I0 
 
 SMOset   m_I0  
 
 {i: 0  
 
 
 
 
 
m_I1 
 
 SMOset   m_I1  
 
 {i: m_class[i] = 1, m_alpha[i] = 0}
 
 
 
 
 
 
m_I2 
 
 SMOset   m_I2  
 
 {i: m_class[i] = -1, m_alpha[i] = C}
 
 
 
 
 
 
m_I3 
 
 SMOset   m_I3  
 
 {i: m_class[i] = 1, m_alpha[i] = C}
 
 
 
 
 
 
m_I4 
 
 SMOset   m_I4  
 
 {i: m_class[i] = -1, m_alpha[i] = 0}
 
 
 
 
 
 
m_supportVectors 
 
 SMOset   m_supportVectors  
 
 The set of support vectors {i: 0  
 
 
 
 
 
m_logistic 
 
 Logistic   m_logistic  
 
 Stores logistic regression model for probability estimate
 
 
 
 
 
 
m_sumOfWeights 
 
double  m_sumOfWeights  
 
 Stores the weight of the training instances
 
 
 
 

 
   
 
 
  
 Class  weka.classifiers.mi.MISVM  extends  Classifier  implements Serializable   
 
 

 
 serialVersionUID:  7622231064035278145L

 
   
 
 
  
 Serialized Fields   
 
 

 
m_SparseFilter 
 
 Filter   m_SparseFilter  
 
 The filter used to transform the sparse datasets to nonsparse
 
 
 
 
 
 
m_SVM 
 
weka.classifiers.mi.MISVM.SVM  m_SVM  
 
 The SMO classifier used to compute SVM soluton w,b for the dataset
 
 
 
 
 
 
m_kernel 
 
 Kernel   m_kernel  
 
 the kernel to use
 
 
 
 
 
 
m_C 
 
double  m_C  
 
 The complexity parameter.
 
 
 
 
 
 
m_Filter 
 
 Filter   m_Filter  
 
 The filter used to standardize/normalize all values.
 
 
 
 
 
 
m_filterType 
 
int  m_filterType  
 
 Whether to normalize/standardize/neither
 
 
 
 
 
 
m_MaxIterations 
 
int  m_MaxIterations  
 
 the maximum number of iterations to perform
 
 
 
 
 
 
m_ConvertToProp 
 
 MultiInstanceToPropositional   m_ConvertToProp  
 
 filter used to convert the MI dataset into single-instance dataset
 
 
 
 

 
   
 
 
  
 Class  weka.classifiers.mi.MIWrapper  extends  SingleClassifierEnhancer  implements Serializable   
 
 

 
 serialVersionUID:  -7707766152904315910L

 
   
 
 
  
 Serialized Fields   
 
 

 
m_NumClasses 
 
int  m_NumClasses  
 
 The number of the class labels
 
 
 
 
 
 
m_Method 
 
int  m_Method  
 
 the test method
 
 
 
 
 
 
m_ConvertToProp 
 
 MultiInstanceToPropositional   m_ConvertToProp  
 
 Filter used to convert MI dataset into single-instance dataset
 
 
 
 
 
 
m_WeightMethod 
 
int  m_WeightMethod  
 
 the single-instance weight setting method
 
 
 
 

 
   
 
 
  
 Class  weka.classifiers.mi.SimpleMI  extends  SingleClassifierEnhancer  implements Serializable   
 
 

 
 serialVersionUID:  9137795893666592662L

 
   
 
 
  
 Serialized Fields   
 
 

 
m_TransformMethod 
 
int  m_TransformMethod  
 
 the method used in transformation
 
 
 
 
 

 
 
  
 Package   weka.classifiers.mi.supportVector   
 
 

 
   
 
 
  
 Class  weka.classifiers.mi.supportVector.MIPolyKernel  extends  PolyKernel  implements Serializable   
 
 

 
 serialVersionUID:  7926421479341051777L

 

 
   
 
 
  
 Class  weka.classifiers.mi.supportVector.MIRBFKernel  extends  RBFKernel  implements Serializable   
 
 

 
 serialVersionUID:  -8711882393708956962L

 
   
 
 
  
 Serialized Fields   
 
 

 
m_kernelPrecalc 
 
double[][]  m_kernelPrecalc  
 
 The precalculated dotproducts of <inst_i,inst_i>
 
 
 
 
 

 
 
  
 Package   weka.classifiers.misc   
 
 

 
   
 
 
  
 Class  weka.classifiers.misc.HyperPipes  extends  Classifier  implements Serializable   
 
 

 
 serialVersionUID:  -7527596632268975274L

 
   
 
 
  
 Serialized Fields   
 
 

 
m_ClassIndex 
 
int  m_ClassIndex  
 
 The index of the class attribute
 
 
 
 
 
 
m_Instances 
 
 Instances   m_Instances  
 
 The structure of the training data
 
 
 
 
 
 
m_HyperPipes 
 
weka.classifiers.misc.HyperPipes.HyperPipe[]  m_HyperPipes  
 
 Stores the HyperPipe for each class
 
 
 
 
 
 
m_ZeroR 
 
 Classifier   m_ZeroR  
 
 a ZeroR model in case no model can be built from the data
 
 
 
 

 
   
 
 
  
 Class  weka.classifiers.misc.SerializedClassifier  extends  Classifier  implements Serializable   
 
 

 
 serialVersionUID:  4599593909947628642L

 
   
 
 
  
 Serialized Fields   
 
 

 
m_ModelFile 
 
java.io.File  m_ModelFile  
 
 the file where the serialized model is stored
 
 
 
 

 
   
 
 
  
 Class  weka.classifiers.misc.VFI  extends  Classifier  implements Serializable   
 
 

 
 serialVersionUID:  8081692166331321866L

 
   
 
 
  
 Serialized Fields   
 
 

 
m_ClassIndex 
 
int  m_ClassIndex  
 
 The index of the class attribute
 
 
 
 
 
 
m_NumClasses 
 
int  m_NumClasses  
 
 The number of classes
 
 
 
 
 
 
m_Instances 
 
 Instances   m_Instances  
 
 The training data
 
 
 
 
 
 
m_counts 
 
double[][][]  m_counts  
 
 The class counts for each interval of each attribute
 
 
 
 
 
 
m_globalCounts 
 
double[]  m_globalCounts  
 
 The global class counts
 
 
 
 
 
 
m_intervalBounds 
 
double[][]  m_intervalBounds  
 
 The lower bounds for each attribute
 
 
 
 
 
 
m_maxEntrop 
 
double  m_maxEntrop  
 
 The maximum entropy for the class
 
 
 
 
 
 
m_weightByConfidence 
 
boolean  m_weightByConfidence  
 
 Exponentially bias more confident intervals
 
 
 
 
 
 
m_bias 
 
double  m_bias  
 
 Bias towards more confident intervals
 
 
 
 
 
 
TINY 
 
double  TINY  
 
 
 
 
 

 
 
  
 Package   weka.classifiers.pmml.consumer   
 
 

 
   
 
 
  
 Class  weka.classifiers.pmml.consumer.GeneralRegression  extends  PMMLClassifier  implements Serializable   
 
 

 
 serialVersionUID:  2583880411828388959L

 
   
 
 
  
 Serialized Fields   
 
 

 
m_modelType 
 
weka.classifiers.pmml.consumer.GeneralRegression.ModelType  m_modelType  
 
 
 
 
 
 
m_modelName 
 
java.lang.String  m_modelName  
 
 
 
 
 
 
m_algorithmName 
 
java.lang.String  m_algorithmName  
 
 
 
 
 
 
m_functionType 
 
int  m_functionType  
 
 
 
 
 
 
m_cumulativeLinkFunction 
 
weka.classifiers.pmml.consumer.GeneralRegression.CumulativeLinkFunction  m_cumulativeLinkFunction  
 
 
 
 
 
 
m_linkFunction 
 
weka.classifiers.pmml.consumer.GeneralRegression.LinkFunction  m_linkFunction  
 
 
 
 
 
 
m_linkParameter 
 
double  m_linkParameter  
 
 
 
 
 
 
m_trialsVariable 
 
java.lang.String  m_trialsVariable  
 
 
 
 
 
 
m_trialsValue 
 
double  m_trialsValue  
 
 
 
 
 
 
m_distribution 
 
weka.classifiers.pmml.consumer.GeneralRegression.Distribution  m_distribution  
 
 
 
 
 
 
m_distParameter 
 
double  m_distParameter  
 
 
 
 
 
 
m_offsetVariable 
 
java.lang.String  m_offsetVariable  
 
 
 
 
 
 
m_offsetValue 
 
double  m_offsetValue  
 
 
 
 
 
 
m_parameterList 
 
java.util.ArrayList<E>  m_parameterList  
 
 
 
 
 
 
m_factorList 
 
java.util.ArrayList<E>  m_factorList  
 
 
 
 
 
 
m_covariateList 
 
java.util.ArrayList<E>  m_covariateList  
 
 
 
 
 
 
m_ppMatrix 
 
weka.classifiers.pmml.consumer.GeneralRegression.PPCell[][]  m_ppMatrix  
 
 
 
 
 
 
m_paramMatrix 
 
weka.classifiers.pmml.consumer.GeneralRegression.PCell[][]  m_paramMatrix  
 
 
 
 

 
   
 
 
  
 Class  weka.classifiers.pmml.consumer.NeuralNetwork  extends  PMMLClassifier  implements Serializable   
 
 

 
 serialVersionUID:  -4545904813133921249L

 
   
 
 
  
 Serialized Fields   
 
 

 
m_functionType 
 
weka.classifiers.pmml.consumer.NeuralNetwork.MiningFunction  m_functionType  
 
 The mining function
 
 
 
 
 
 
m_activationFunction 
 
weka.classifiers.pmml.consumer.NeuralNetwork.ActivationFunction  m_activationFunction  
 
 The activation function to use
 
 
 
 
 
 
m_normalizationMethod 
 
weka.classifiers.pmml.consumer.NeuralNetwork.Normalization  m_normalizationMethod  
 
 The normalization method
 
 
 
 
 
 
m_threshold 
 
double  m_threshold  
 
 Threshold activation
 
 
 
 
 
 
m_width 
 
double  m_width  
 
 Width for radial basis
 
 
 
 
 
 
m_altitude 
 
double  m_altitude  
 
 Altitude for radial basis
 
 
 
 
 
 
m_numberOfInputs 
 
int  m_numberOfInputs  
 
 The number of inputs to the network
 
 
 
 
 
 
m_numberOfLayers 
 
int  m_numberOfLayers  
 
 Number of hidden layers in the network
 
 
 
 
 
 
m_inputs 
 
weka.classifiers.pmml.consumer.NeuralNetwork.NeuralInput[]  m_inputs  
 
 The inputs to the network
 
 
 
 
 
 
m_inputMap 
 
java.util.HashMap<K,V>  m_inputMap  
 
 A map for storing network input values (computed from an incoming instance)
 
 
 
 
 
 
m_layers 
 
weka.classifiers.pmml.consumer.NeuralNetwork.NeuralLayer[]  m_layers  
 
 The hidden layers in the network
 
 
 
 
 
 
m_outputs 
 
weka.classifiers.pmml.consumer.NeuralNetwork.NeuralOutputs  m_outputs  
 
 The outputs of the network
 
 
 
 

 
   
 
 
  
 Class  weka.classifiers.pmml.consumer.PMMLClassifier  extends  Classifier  implements Serializable   
 
 

 
 serialVersionUID:  -5371600590320702971L

 
   
 
 
  
 Serialized Fields   
 
 

 
m_pmmlVersion 
 
java.lang.String  m_pmmlVersion  
 
 PMML version
 
 
 
 
 
 
m_creatorApplication 
 
java.lang.String  m_creatorApplication  
 
 Creator application
 
 
 
 
 
 
m_log 
 
 Logger   m_log  
 
 Logger
 
 
 
 
 
 
m_dataDictionary 
 
 Instances   m_dataDictionary  
 
 The data dictionary
 
 
 
 
 
 
m_miningSchema 
 
 MiningSchema   m_miningSchema  
 
 The fields and meta data used by the model
 
 
 
 

 
   
 
 
  
 Class  weka.classifiers.pmml.consumer.Regression  extends  PMMLClassifier  implements Serializable   
 
 

 
 serialVersionUID:  -5551125528409488634L

 
   
 
 
  
 Serialized Fields   
 
 

 
m_algorithmName 
 
java.lang.String  m_algorithmName  
 
 Description of the algorithm
 
 
 
 
 
 
m_regressionTables 
 
weka.classifiers.pmml.consumer.Regression.RegressionTable[]  m_regressionTables  
 
 The regression tables for this regression
 
 
 
 
 
 
m_normalizationMethod 
 
weka.classifiers.pmml.consumer.Regression.Normalization  m_normalizationMethod  
 
 The normalization to use
 
 
 
 

 
   
 
 
  
 Class weka.classifiers.pmml.consumer.Regression.RegressionTable.CategoricalPredictor extends weka.classifiers.pmml.consumer.Regression.RegressionTable.Predictor implements Serializable   
 
 

 
 serialVersionUID:  3077920125549906819L

 
   
 
 
  
 Serialized Fields   
 
 

 
m_valueName 
 
java.lang.String  m_valueName  
 
 The attribute value for this predictor
 
 
 
 
 
 
m_valueIndex 
 
int  m_valueIndex  
 
 The index of the attribute value for this predictor
 
 
 
 

 
   
 
 
  
 Class weka.classifiers.pmml.consumer.Regression.RegressionTable.NumericPredictor extends weka.classifiers.pmml.consumer.Regression.RegressionTable.Predictor implements Serializable   
 
 

 
 serialVersionUID:  -4335075205696648273L

 
   
 
 
  
 Serialized Fields   
 
 

 
m_exponent 
 
double  m_exponent  
 
 The exponent
 
 
 
 

 
   
 
 
  
 Class weka.classifiers.pmml.consumer.Regression.RegressionTable.PredictorTerm extends java.lang.Object implements Serializable   
 
 

 
 serialVersionUID:  5493100145890252757L

 
   
 
 
  
 Serialized Fields   
 
 

 
m_coefficient 
 
double  m_coefficient  
 
 The coefficient for this predictor term
 
 
 
 
 
 
m_indexes 
 
int[]  m_indexes  
 
 the indexes of the terms to be multiplied
 
 
 
 
 
 
m_fieldNames 
 
java.lang.String[]  m_fieldNames  
 
 The names of the terms (attributes) to be multiplied
 
 
 
 
 

 
 
  
 Package   weka.classifiers.rules   
 
 

 
   
 
 
  
 Class  weka.classifiers.rules.ConjunctiveRule  extends  Classifier  implements Serializable   
 
 

 
 serialVersionUID:  -5938309903225087198L

 
   
 
 
  
 Serialized Fields   
 
 

 
m_Folds 
 
int  m_Folds  
 
 The number of folds to split data into Grow and Prune for REP
 
 
 
 
 
 
m_ClassAttribute 
 
 Attribute   m_ClassAttribute  
 
 The class attribute of the data
 
 
 
 
 
 
m_Antds 
 
 FastVector   m_Antds  
 
 The vector of antecedents of this rule
 
 
 
 
 
 
m_DefDstr 
 
double[]  m_DefDstr  
 
 The default rule distribution of the data not covered
 
 
 
 
 
 
m_Cnsqt 
 
double[]  m_Cnsqt  
 
 The consequent of this rule
 
 
 
 
 
 
m_NumClasses 
 
int  m_NumClasses  
 
 Number of classes in the training data
 
 
 
 
 
 
m_Seed 
 
long  m_Seed  
 
 The seed to perform randomization
 
 
 
 
 
 
m_Random 
 
java.util.Random  m_Random  
 
 The Random object used for randomization
 
 
 
 
 
 
m_Targets 
 
 FastVector   m_Targets  
 
 The predicted classes recorded for each antecedent in the growing data
 
 
 
 
 
 
m_IsExclude 
 
boolean  m_IsExclude  
 
 Whether to use exlusive expressions for nominal attributes
 
 
 
 
 
 
m_MinNo 
 
double  m_MinNo  
 
 The minimal number of instance weights within a split
 
 
 
 
 
 
m_NumAntds 
 
int  m_NumAntds  
 
 The number of antecedents in pre-pruning
 
 
 
 

 
   
 
 
  
 Class  weka.classifiers.rules.DecisionTable  extends  Classifier  implements Serializable   
 
 

 
 serialVersionUID:  2888557078165701326L

 
   
 
 
  
 Serialized Fields   
 
 

 
m_entries 
 
java.util.Hashtable<K,V>  m_entries  
 
 The hashtable used to hold training instances
 
 
 
 
 
 
m_classPriorCounts 
 
double[]  m_classPriorCounts  
 
 The class priors to use when there is no match in the table
 
 
 
 
 
 
m_classPriors 
 
double[]  m_classPriors  
 
 
 
 
 
 
m_decisionFeatures 
 
int[]  m_decisionFeatures  
 
 Holds the final feature set
 
 
 
 
 
 
m_disTransform 
 
 Filter   m_disTransform  
 
 Discretization filter
 
 
 
 
 
 
m_delTransform 
 
 Remove   m_delTransform  
 
 Filter used to remove columns discarded by feature selection
 
 
 
 
 
 
m_ibk 
 
 IBk   m_ibk  
 
 IB1 used to classify non matching instances rather than majority class
 
 
 
 
 
 
m_theInstances 
 
 Instances   m_theInstances  
 
 Holds the original training instances
 
 
 
 
 
 
m_dtInstances 
 
 Instances   m_dtInstances  
 
 Holds the final feature selected set of instances
 
 
 
 
 
 
m_numAttributes 
 
int  m_numAttributes  
 
 The number of attributes in the dataset
 
 
 
 
 
 
m_numInstances 
 
int  m_numInstances  
 
 The number of instances in the dataset
 
 
 
 
 
 
m_classIsNominal 
 
boolean  m_classIsNominal  
 
 Class is nominal
 
 
 
 
 
 
m_useIBk 
 
boolean  m_useIBk  
 
 Use the IBk classifier rather than majority class
 
 
 
 
 
 
m_displayRules 
 
boolean  m_displayRules  
 
 Display Rules
 
 
 
 
 
 
m_CVFolds 
 
int  m_CVFolds  
 
 Number of folds for cross validating feature sets
 
 
 
 
 
 
m_rr 
 
java.util.Random  m_rr  
 
 Random numbers for use in cross validation
 
 
 
 
 
 
m_majority 
 
double  m_majority  
 
 Holds the majority class
 
 
 
 
 
 
m_search 
 
 ASSearch   m_search  
 
 The search method to use
 
 
 
 
 
 
m_evaluator 
 
 ASEvaluation   m_evaluator  
 
 Our own internal evaluator
 
 
 
 
 
 
m_evaluation 
 
 Evaluation   m_evaluation  
 
 The evaluation object used to evaluate subsets
 
 
 
 
 
 
m_evaluationMeasure 
 
int  m_evaluationMeasure  
 
 
 
 
 
 
m_saveMemory 
 
boolean  m_saveMemory  
 
 
 
 

 
   
 
 
  
 Class  weka.classifiers.rules.DecisionTableHashKey  extends java.lang.Object implements Serializable   
 
 

 
 serialVersionUID:  5674163500154964602L

 
   
 
 
  
 Serialized Fields   
 
 

 
attributes 
 
double[]  attributes  
 
 Array of attribute values for an instance
 
 
 
 
 
 
missing 
 
boolean[]  missing  
 
 True for an index if the corresponding attribute value is missing.
 
 
 
 
 
 
key 
 
int  key  
 
 The key
 
 
 
 

 
   
 
 
  
 Class  weka.classifiers.rules.DTNB  extends  DecisionTable  implements Serializable   
 
 

 
 serialVersionUID:  2999557077765701326L

 
   
 
 
  
 Serialized Fields   
 
 

 
m_NB 
 
 NaiveBayes   m_NB  
 
 The naive Bayes half of the hybrid
 
 
 
 
 
 
m_nbFeatures 
 
int[]  m_nbFeatures  
 
 The features used by naive Bayes
 
 
 
 
 
 
m_percentUsedByDT 
 
double  m_percentUsedByDT  
 
 Percentage of the total number of features used by the decision table
 
 
 
 
 
 
m_percentDeleted 
 
double  m_percentDeleted  
 
 Percentage of the features features that were dropped entirely
 
 
 
 
 
 
m_backwardWithDelete 
 
 ASSearch   m_backwardWithDelete  
 
 
 
 

 
   
 
 
  
 Class weka.classifiers.rules.DTNB.BackwardsWithDelete extends  ASSearch  implements Serializable   
 
 

 

 
   
 
 
  
 Class weka.classifiers.rules.DTNB.EvalWithDelete extends  ASEvaluation  implements Serializable   
 
 

 
   
 
 
  
 Serialized Fields   
 
 

 
m_deletedFromDTNB 
 
java.util.BitSet  m_deletedFromDTNB  
 
 
 
 

 
   
 
 
  
 Class  weka.classifiers.rules.JRip  extends  Classifier  implements Serializable   
 
 

 
 serialVersionUID:  -6589312996832147161L

 
   
 
 
  
 Serialized Fields   
 
 

 
m_Class 
 
 Attribute   m_Class  
 
 The class attribute of the data
 
 
 
 
 
 
m_Ruleset 
 
 FastVector   m_Ruleset  
 
 The ruleset
 
 
 
 
 
 
m_Distributions 
 
 FastVector   m_Distributions  
 
 The predicted class distribution
 
 
 
 
 
 
m_Optimizations 
 
int  m_Optimizations  
 
 Runs of optimizations
 
 
 
 
 
 
m_Random 
 
java.util.Random  m_Random  
 
 Random object used in this class
 
 
 
 
 
 
m_Total 
 
double  m_Total  
 
 # of all the possible conditions in a rule
 
 
 
 
 
 
m_Seed 
 
long  m_Seed  
 
 The seed to perform randomization
 
 
 
 
 
 
m_Folds 
 
int  m_Folds  
 
 The number of folds to split data into Grow and Prune for IREP
 
 
 
 
 
 
m_MinNo 
 
double  m_MinNo  
 
 The minimal number of instance weights within a split
 
 
 
 
 
 
m_Debug 
 
boolean  m_Debug  
 
 Whether in a debug mode
 
 
 
 
 
 
m_CheckErr 
 
boolean  m_CheckErr  
 
 Whether check the error rate >= 0.5 in stopping criteria
 
 
 
 
 
 
m_UsePruning 
 
boolean  m_UsePruning  
 
 Whether use pruning, i.e. the data is clean or not
 
 
 
 
 
 
m_Filter 
 
 Filter   m_Filter  
 
 The filter used to randomize the class order
 
 
 
 
 
 
m_RulesetStats 
 
 FastVector   m_RulesetStats  
 
 The RuleStats for the ruleset of each class value
 
 
 
 

 
   
 
 
  
 Class weka.classifiers.rules.JRip.RipperRule extends  Rule  implements Serializable   
 
 

 
 serialVersionUID:  -2410020717305262952L

 
   
 
 
  
 Serialized Fields   
 
 

 
m_Consequent 
 
double  m_Consequent  
 
 The internal representation of the class label to be predicted
 
 
 
 
 
 
m_Antds 
 
 FastVector   m_Antds  
 
 The vector of antecedents of this rule
 
 
 
 

 
   
 
 
  
 Class  weka.classifiers.rules.M5Rules  extends  M5Base  implements Serializable   
 
 

 
 serialVersionUID:  -1746114858746563180L

 

 
   
 
 
  
 Class  weka.classifiers.rules.NNge  extends  Classifier  implements Serializable   
 
 

 
 serialVersionUID:  4084742275553788972L

 
   
 
 
  
 Serialized Fields   
 
 

 
m_Train 
 
 Instances   m_Train  
 
 An empty instances to keep the headers, the classIndex, etc...
 
 
 
 
 
 
m_Exemplars 
 
weka.classifiers.rules.NNge.Exemplar  m_Exemplars  
 
 The list of Exemplars
 
 
 
 
 
 
m_ExemplarsByClass 
 
weka.classifiers.rules.NNge.Exemplar[]  m_ExemplarsByClass  
 
 The lists of Exemplars by class
 
 
 
 
 
 
m_MinArray 
 
double[]  m_MinArray  
 
 The minimum values for numeric attributes.
 
 
 
 
 
 
m_MaxArray 
 
double[]  m_MaxArray  
 
 The maximum values for numeric attributes.
 
 
 
 
 
 
m_NumAttemptsOfGene 
 
int  m_NumAttemptsOfGene  
 
 The number of try for generalisation
 
 
 
 
 
 
m_NumFoldersMI 
 
int  m_NumFoldersMI  
 
 The number of folder for the Mutual Information
 
 
 
 
 
 
m_MissingVector 
 
double[]  m_MissingVector  
 
 Values to use for missing value
 
 
 
 
 
 
m_MI_NumAttrClassInter 
 
int[][][]  m_MI_NumAttrClassInter  
 
 MUTUAL INFORMATION'S DATAS
 
 
 
 
 
 
m_MI_NumAttrInter 
 
int[][]  m_MI_NumAttrInter  
 
 
 
 
 
 
m_MI_MaxArray 
 
double[]  m_MI_MaxArray  
 
 
 
 
 
 
m_MI_MinArray 
 
double[]  m_MI_MinArray  
 
 
 
 
 
 
m_MI_NumAttrClassValue 
 
int[][][]  m_MI_NumAttrClassValue  
 
 
 
 
 
 
m_MI_NumAttrValue 
 
int[][]  m_MI_NumAttrValue  
 
 
 
 
 
 
m_MI_NumClass 
 
int[]  m_MI_NumClass  
 
 
 
 
 
 
m_MI_NumInst 
 
int  m_MI_NumInst  
 
 
 
 
 
 
m_MI 
 
double[]  m_MI  
 
 
 
 

 
   
 
 
  
 Class  weka.classifiers.rules.OneR  extends  Classifier  implements Serializable   
 
 

 
 serialVersionUID:  -2459427002147861445L

 
   
 
 
  
 Serialized Fields   
 
 

 
m_rule 
 
weka.classifiers.rules.OneR.OneRRule  m_rule  
 
 A 1-R rule
 
 
 
 
 
 
m_minBucketSize 
 
int  m_minBucketSize  
 
 The minimum bucket size
 
 
 
 
 
 
m_ZeroR 
 
 Classifier   m_ZeroR  
 
 a ZeroR model in case no model can be built from the data
 
 
 
 

 
   
 
 
  
 Class  weka.classifiers.rules.PART  extends  Classifier  implements Serializable   
 
 

 
 serialVersionUID:  8121455039782598361L

 
   
 
 
  
 Serialized Fields   
 
 

 
m_root 
 
 MakeDecList   m_root  
 
 The decision list
 
 
 
 
 
 
m_CF 
 
float  m_CF  
 
 Confidence level
 
 
 
 
 
 
m_minNumObj 
 
int  m_minNumObj  
 
 Minimum number of objects
 
 
 
 
 
 
m_reducedErrorPruning 
 
boolean  m_reducedErrorPruning  
 
 Use reduced error pruning?
 
 
 
 
 
 
m_numFolds 
 
int  m_numFolds  
 
 Number of folds for reduced error pruning.
 
 
 
 
 
 
m_binarySplits 
 
boolean  m_binarySplits  
 
 Binary splits on nominal attributes?
 
 
 
 
 
 
m_unpruned 
 
boolean  m_unpruned  
 
 Generate unpruned list?
 
 
 
 
 
 
m_Seed 
 
int  m_Seed  
 
 The seed for random number generation.
 
 
 
 

 
   
 
 
  
 Class  weka.classifiers.rules.Prism  extends  Classifier  implements Serializable   
 
 

 
 serialVersionUID:  1310258880025902106L

 
   
 
 
  
 Serialized Fields   
 
 

 
m_rules 
 
weka.classifiers.rules.Prism.PrismRule  m_rules  
 
 The first rule in the list of rules
 
 
 
 

 
   
 
 
  
 Class  weka.classifiers.rules.Ridor  extends  Classifier  implements Serializable   
 
 

 
 serialVersionUID:  -7261533075088314436L

 
   
 
 
  
 Serialized Fields   
 
 

 
m_Folds 
 
int  m_Folds  
 
 The number of folds to split data into Grow and Prune for IREP
 
 
 
 
 
 
m_Shuffle 
 
int  m_Shuffle  
 
 The number of shuffles performed on the data for randomization
 
 
 
 
 
 
m_Random 
 
java.util.Random  m_Random  
 
 Random object for randomization
 
 
 
 
 
 
m_Seed 
 
int  m_Seed  
 
 The seed to perform randomization
 
 
 
 
 
 
m_IsAllErr 
 
boolean  m_IsAllErr  
 
 Whether use error rate on all the data
 
 
 
 
 
 
m_IsMajority 
 
boolean  m_IsMajority  
 
 Whether use majority class as default class
 
 
 
 
 
 
m_Root 
 
weka.classifiers.rules.Ridor.Ridor_node  m_Root  
 
 The root of Ridor
 
 
 
 
 
 
m_Class 
 
 Attribute   m_Class  
 
 The class attribute of the data
 
 
 
 
 
 
m_Cover 
 
double  m_Cover  
 
 Statistics of the data
 
 
 
 
 
 
m_Err 
 
double  m_Err  
 
 Statistics of the data
 
 
 
 
 
 
m_MinNo 
 
double  m_MinNo  
 
 The minimal number of instance weights within a split
 
 
 
 

 
   
 
 
  
 Class  weka.classifiers.rules.Rule  extends java.lang.Object implements Serializable   
 
 

 
 serialVersionUID:  8815687740470471229L

 

 
   
 
 
  
 Class  weka.classifiers.rules.RuleStats  extends java.lang.Object implements Serializable   
 
 

 
 serialVersionUID:  -5708153367675298624L

 
   
 
 
  
 Serialized Fields   
 
 

 
m_Data 
 
 Instances   m_Data  
 
 The data on which the stats calculation is based
 
 
 
 
 
 
m_Ruleset 
 
 FastVector   m_Ruleset  
 
 The specific ruleset in question
 
 
 
 
 
 
m_SimpleStats 
 
 FastVector   m_SimpleStats  
 
 The simple stats of each rule
 
 
 
 
 
 
m_Filtered 
 
 FastVector   m_Filtered  
 
 The set of instances filtered by the ruleset
 
 
 
 
 
 
m_Total 
 
double  m_Total  
 
 The total number of possible conditions that could
  appear in a rule
 
 
 
 
 
 
MDL_THEORY_WEIGHT 
 
double  MDL_THEORY_WEIGHT  
 
 The theory weight in the MDL calculation
 
 
 
 
 
 
m_Distributions 
 
 FastVector   m_Distributions  
 
 The class distributions predicted by each rule
 
 
 
 

 
   
 
 
  
 Class  weka.classifiers.rules.ZeroR  extends  Classifier  implements Serializable   
 
 

 
 serialVersionUID:  48055541465867954L

 
   
 
 
  
 Serialized Fields   
 
 

 
m_ClassValue 
 
double  m_ClassValue  
 
 The class value 0R predicts.
 
 
 
 
 
 
m_Counts 
 
double[]  m_Counts  
 
 The number of instances in each class (null if class numeric).
 
 
 
 
 
 
m_Class 
 
 Attribute   m_Class  
 
 The class attribute.
 
 
 
 
 

 
 
  
 Package   weka.classifiers.rules.part   
 
 

 
   
 
 
  
 Class  weka.classifiers.rules.part.C45PruneableDecList  extends  ClassifierDecList  implements Serializable   
 
 

 
 serialVersionUID:  -2757684345218324559L

 
   
 
 
  
 Serialized Fields   
 
 

 
CF 
 
double  CF  
 
 CF
 
 
 
 

 
   
 
 
  
 Class  weka.classifiers.rules.part.ClassifierDecList  extends java.lang.Object implements Serializable   
 
 

 
 serialVersionUID:  7284358349711992497L

 
   
 
 
  
 Serialized Fields   
 
 

 
m_minNumObj 
 
int  m_minNumObj  
 
 Minimum number of objects
 
 
 
 
 
 
m_toSelectModel 
 
 ModelSelection   m_toSelectModel  
 
 The model selection method.
 
 
 
 
 
 
m_localModel 
 
 ClassifierSplitModel   m_localModel  
 
 Local model at node.
 
 
 
 
 
 
m_sons 
 
 ClassifierDecList []  m_sons  
 
 References to sons.
 
 
 
 
 
 
m_isLeaf 
 
boolean  m_isLeaf  
 
 True if node is leaf.
 
 
 
 
 
 
m_isEmpty 
 
boolean  m_isEmpty  
 
 True if node is empty.
 
 
 
 
 
 
m_train 
 
 Instances   m_train  
 
 The training instances.
 
 
 
 
 
 
m_test 
 
 Distribution   m_test  
 
 The pruning instances.
 
 
 
 
 
 
indeX 
 
int  indeX  
 
 Which son to expand?
 
 
 
 

 
   
 
 
  
 Class  weka.classifiers.rules.part.MakeDecList  extends java.lang.Object implements Serializable   
 
 

 
 serialVersionUID:  -1427481323245079123L

 
   
 
 
  
 Serialized Fields   
 
 

 
theRules 
 
java.util.Vector<E>  theRules  
 
 Vector storing the rules.
 
 
 
 
 
 
CF 
 
double  CF  
 
 The confidence for C45-type pruning.
 
 
 
 
 
 
minNumObj 
 
int  minNumObj  
 
 Minimum number of objects
 
 
 
 
 
 
toSelectModeL 
 
 ModelSelection   toSelectModeL  
 
 The model selection method.
 
 
 
 
 
 
numSetS 
 
int  numSetS  
 
 How many subsets of equal size? One used for pruning, the rest for training.
 
 
 
 
 
 
reducedErrorPruning 
 
boolean  reducedErrorPruning  
 
 Use reduced error pruning?
 
 
 
 
 
 
unpruned 
 
boolean  unpruned  
 
 Generated unpruned list?
 
 
 
 
 
 
m_seed 
 
int  m_seed  
 
 The seed for random number generation.
 
 
 
 

 
   
 
 
  
 Class  weka.classifiers.rules.part.PruneableDecList  extends  ClassifierDecList  implements Serializable   
 
 

 
 serialVersionUID:  -7228103346297172921L

 
 

 
 
  
 Package   weka.classifiers.trees   
 
 

 
   
 
 
  
 Class  weka.classifiers.trees.ADTree  extends  Classifier  implements Serializable   
 
 

 
 serialVersionUID:  -1532264837167690683L

 
   
 
 
  
 Serialized Fields   
 
 

 
m_trainInstances 
 
 Instances   m_trainInstances  
 
 The instances used to train the tree
 
 
 
 
 
 
m_root 
 
 PredictionNode   m_root  
 
 The root of the tree
 
 
 
 
 
 
m_random 
 
java.util.Random  m_random  
 
 The random number generator - used for the random search heuristic
 
 
 
 
 
 
m_lastAddedSplitNum 
 
int  m_lastAddedSplitNum  
 
 The number of the last splitter added to the tree
 
 
 
 
 
 
m_numericAttIndices 
 
int[]  m_numericAttIndices  
 
 An array containing the inidices to the numeric attributes in the data
 
 
 
 
 
 
m_nominalAttIndices 
 
int[]  m_nominalAttIndices  
 
 An array containing the inidices to the nominal attributes in the data
 
 
 
 
 
 
m_trainTotalWeight 
 
double  m_trainTotalWeight  
 
 The total weight of the instances - used to speed Z calculations
 
 
 
 
 
 
m_posTrainInstances 
 
 ReferenceInstances   m_posTrainInstances  
 
 The training instances with positive class - referencing the training dataset
 
 
 
 
 
 
m_negTrainInstances 
 
 ReferenceInstances   m_negTrainInstances  
 
 The training instances with negative class - referencing the training dataset
 
 
 
 
 
 
m_search_bestInsertionNode 
 
 PredictionNode   m_search_bestInsertionNode  
 
 The best node to insert under, as found so far by the latest search
 
 
 
 
 
 
m_search_bestSplitter 
 
 Splitter   m_search_bestSplitter  
 
 The best splitter to insert, as found so far by the latest search
 
 
 
 
 
 
m_search_smallestZ 
 
double  m_search_smallestZ  
 
 The smallest Z value found so far by the latest search
 
 
 
 
 
 
m_search_bestPathPosInstances 
 
 Instances   m_search_bestPathPosInstances  
 
 The positive instances that apply to the best path found so far
 
 
 
 
 
 
m_search_bestPathNegInstances 
 
 Instances   m_search_bestPathNegInstances  
 
 The negative instances that apply to the best path found so far
 
 
 
 
 
 
m_nodesExpanded 
 
int  m_nodesExpanded  
 
 Statistics - the number of prediction nodes investigated during search
 
 
 
 
 
 
m_examplesCounted 
 
int  m_examplesCounted  
 
 Statistics - the number of instances processed during search
 
 
 
 
 
 
m_boostingIterations 
 
int  m_boostingIterations  
 
 Option - the number of boosting iterations o perform
 
 
 
 
 
 
m_searchPath 
 
int  m_searchPath  
 
 Option - the search mode
 
 
 
 
 
 
m_randomSeed 
 
int  m_randomSeed  
 
 Option - the seed to use for a random search
 
 
 
 
 
 
m_saveInstanceData 
 
boolean  m_saveInstanceData  
 
 Option - whether the tree should remember the instance data
 
 
 
 

 
   
 
 
  
 Class  weka.classifiers.trees.BFTree  extends  RandomizableClassifier  implements Serializable   
 
 

 
 serialVersionUID:  -7035607375962528217L

 
   
 
 
  
 Serialized Fields   
 
 

 
m_PruningStrategy 
 
int  m_PruningStrategy  
 
 the pruning strategy
 
 
 
 
 
 
m_Successors 
 
 BFTree []  m_Successors  
 
 Successor nodes.
 
 
 
 
 
 
m_Attribute 
 
 Attribute   m_Attribute  
 
 Attribute used for splitting.
 
 
 
 
 
 
m_SplitValue 
 
double  m_SplitValue  
 
 Split point (for numeric attributes).
 
 
 
 
 
 
m_SplitString 
 
java.lang.String  m_SplitString  
 
 Split subset (for nominal attributes).
 
 
 
 
 
 
m_ClassValue 
 
double  m_ClassValue  
 
 Class value for a node.
 
 
 
 
 
 
m_ClassAttribute 
 
 Attribute   m_ClassAttribute  
 
 Class attribute of a dataset.
 
 
 
 
 
 
m_minNumObj 
 
int  m_minNumObj  
 
 Minimum number of instances at leaf nodes.
 
 
 
 
 
 
m_numFoldsPruning 
 
int  m_numFoldsPruning  
 
 Number of folds for the pruning.
 
 
 
 
 
 
m_isLeaf 
 
boolean  m_isLeaf  
 
 If the ndoe is leaf node.
 
 
 
 
 
 
m_FixedExpansion 
 
int  m_FixedExpansion  
 
 Fixed number of expansions (if no pruning method is used, its value is -1. Otherwise,
  its value is gotten from internal cross-validation).
 
 
 
 
 
 
m_Heuristic 
 
boolean  m_Heuristic  
 
 If use huristic search for binary split (default true). Note even if its value is true, it is only
 used when the number of values of a nominal attribute is larger than 4.
 
 
 
 
 
 
m_UseGini 
 
boolean  m_UseGini  
 
 If use Gini index as the splitting criterion - default (if not, information is used).
 
 
 
 
 
 
m_UseErrorRate 
 
boolean  m_UseErrorRate  
 
 If use error rate in internal cross-validation to fix the number of expansions - default
  (if not, root mean squared error is used).
 
 
 
 
 
 
m_UseOneSE 
 
boolean  m_UseOneSE  
 
 If use the 1SE rule to make the decision.
 
 
 
 
 
 
m_Distribution 
 
double[]  m_Distribution  
 
 Class distributions.
 
 
 
 
 
 
m_Props 
 
double[]  m_Props  
 
 Branch proportions.
 
 
 
 
 
 
m_SortedIndices 
 
int[][]  m_SortedIndices  
 
 Sorted indices.
 
 
 
 
 
 
m_Weights 
 
double[][]  m_Weights  
 
 Sorted weights.
 
 
 
 
 
 
m_Dists 
 
double[][][]  m_Dists  
 
 Distributions of each attribute for two successor nodes.
 
 
 
 
 
 
m_ClassProbs 
 
double[]  m_ClassProbs  
 
 Class probabilities.
 
 
 
 
 
 
m_TotalWeight 
 
double  m_TotalWeight  
 
 Total weights.
 
 
 
 
 
 
m_SizePer 
 
double  m_SizePer  
 
 The training data size (0-1). Default 1.
 
 
 
 

 
   
 
 
  
 Class  weka.classifiers.trees.DecisionStump  extends  Classifier  implements Serializable   
 
 

 
 serialVersionUID:  1618384535950391L

 
   
 
 
  
 Serialized Fields   
 
 

 
m_AttIndex 
 
int  m_AttIndex  
 
 The attribute used for classification.
 
 
 
 
 
 
m_SplitPoint 
 
double  m_SplitPoint  
 
 The split point (index respectively).
 
 
 
 
 
 
m_Distribution 
 
double[][]  m_Distribution  
 
 The distribution of class values or the means in each subset.
 
 
 
 
 
 
m_Instances 
 
 Instances   m_Instances  
 
 The instances used for training.
 
 
 
 
 
 
m_ZeroR 
 
 Classifier   m_ZeroR  
 
 a ZeroR model in case no model can be built from the data
 
 
 
 

 
   
 
 
  
 Class  weka.classifiers.trees.FT  extends  Classifier  implements Serializable   
 
 

 
 serialVersionUID:  -1113212459618105000L

 
   
 
 
  
 Serialized Fields   
 
 

 
m_replaceMissing 
 
 ReplaceMissingValues   m_replaceMissing  
 
 Filter to replace missing values
 
 
 
 
 
 
m_nominalToBinary 
 
 NominalToBinary   m_nominalToBinary  
 
 Filter to replace nominal attributes
 
 
 
 
 
 
m_tree 
 
 FTtree   m_tree  
 
 root of the logistic model tree
 
 
 
 
 
 
m_convertNominal 
 
boolean  m_convertNominal  
 
 convert nominal attributes to binary ?
 
 
 
 
 
 
m_errorOnProbabilities 
 
boolean  m_errorOnProbabilities  
 
 use error on probabilties instead of misclassification for stopping criterion of LogitBoost?
 
 
 
 
 
 
m_minNumInstances 
 
int  m_minNumInstances  
 
 minimum number of instances at which a node is considered for splitting
 
 
 
 
 
 
m_numBoostingIterations 
 
int  m_numBoostingIterations  
 
 if non-zero, use fixed number of iterations for LogitBoost
 
 
 
 
 
 
m_modelType 
 
int  m_modelType  
 
 Model Type, value: 0 is FT, 1 is FTLeaves, 2  is FTInner
 
 
 
 
 
 
m_weightTrimBeta 
 
double  m_weightTrimBeta  
 
 Threshold for trimming weights. Instances with a weight lower than this (as a percentage
 of total weights) are not included in the regression fit.
 
 
 
 
 
 
m_useAIC 
 
boolean  m_useAIC  
 
 If true, the AIC is used to choose the best LogitBoost iteration
 
 
 
 

 
   
 
 
  
 Class  weka.classifiers.trees.Id3  extends  Classifier  implements Serializable   
 
 

 
 serialVersionUID:  -2693678647096322561L

 
   
 
 
  
 Serialized Fields   
 
 

 
m_Successors 
 
 Id3 []  m_Successors  
 
 The node's successors.
 
 
 
 
 
 
m_Attribute 
 
 Attribute   m_Attribute  
 
 Attribute used for splitting.
 
 
 
 
 
 
m_ClassValue 
 
double  m_ClassValue  
 
 Class value if node is leaf.
 
 
 
 
 
 
m_Distribution 
 
double[]  m_Distribution  
 
 Class distribution if node is leaf.
 
 
 
 
 
 
m_ClassAttribute 
 
 Attribute   m_ClassAttribute  
 
 Class attribute of dataset.
 
 
 
 

 
   
 
 
  
 Class  weka.classifiers.trees.J48  extends  Classifier  implements Serializable   
 
 

 
 serialVersionUID:  -217733168393644444L

 
   
 
 
  
 Serialized Fields   
 
 

 
m_root 
 
 ClassifierTree   m_root  
 
 The decision tree
 
 
 
 
 
 
m_unpruned 
 
boolean  m_unpruned  
 
 Unpruned tree?
 
 
 
 
 
 
m_CF 
 
float  m_CF  
 
 Confidence level
 
 
 
 
 
 
m_minNumObj 
 
int  m_minNumObj  
 
 Minimum number of instances
 
 
 
 
 
 
m_useLaplace 
 
boolean  m_useLaplace  
 
 Determines whether probabilities are smoothed using
      Laplace correction when predictions are generated
 
 
 
 
 
 
m_reducedErrorPruning 
 
boolean  m_reducedErrorPruning  
 
 Use reduced error pruning?
 
 
 
 
 
 
m_numFolds 
 
int  m_numFolds  
 
 Number of folds for reduced error pruning.
 
 
 
 
 
 
m_binarySplits 
 
boolean  m_binarySplits  
 
 Binary splits on nominal attributes?
 
 
 
 
 
 
m_subtreeRaising 
 
boolean  m_subtreeRaising  
 
 Subtree raising to be performed?
 
 
 
 
 
 
m_noCleanup 
 
boolean  m_noCleanup  
 
 Cleanup after the tree has been built.
 
 
 
 
 
 
m_Seed 
 
int  m_Seed  
 
 Random number seed for reduced-error pruning.
 
 
 
 

 
   
 
 
  
 Class  weka.classifiers.trees.J48graft  extends  Classifier  implements Serializable   
 
 

 
 serialVersionUID:  8823716098042427799L

 
   
 
 
  
 Serialized Fields   
 
 

 
m_root 
 
 ClassifierTree   m_root  
 
 The decision tree
 
 
 
 
 
 
m_unpruned 
 
boolean  m_unpruned  
 
 Unpruned tree?
 
 
 
 
 
 
m_CF 
 
float  m_CF  
 
 Confidence level
 
 
 
 
 
 
m_minNumObj 
 
int  m_minNumObj  
 
 Minimum number of instances
 
 
 
 
 
 
m_useLaplace 
 
boolean  m_useLaplace  
 
 Determines whether probabilities are smoothed using
      Laplace correction when predictions are generated
 
 
 
 
 
 
m_numFolds 
 
int  m_numFolds  
 
 Number of folds for reduced error pruning.
 
 
 
 
 
 
m_binarySplits 
 
boolean  m_binarySplits  
 
 Binary splits on nominal attributes?
 
 
 
 
 
 
m_subtreeRaising 
 
boolean  m_subtreeRaising  
 
 Subtree raising to be performed?
 
 
 
 
 
 
m_noCleanup 
 
boolean  m_noCleanup  
 
 Cleanup after the tree has been built.
 
 
 
 
 
 
m_relabel 
 
boolean  m_relabel  
 
 relabel instances when grafting
 
 
 
 

 
   
 
 
  
 Class  weka.classifiers.trees.LADTree  extends  Classifier  implements Serializable   
 
 

 
 serialVersionUID:  -4940716114518300302L

 
   
 
 
  
 Serialized Fields   
 
 

 
Z_MAX 
 
double  Z_MAX  
 
 
 
 
 
 
m_numOfClasses 
 
int  m_numOfClasses  
 
 
 
 
 
 
m_trainInstances 
 
 ReferenceInstances   m_trainInstances  
 
 
 
 
 
 
m_root 
 
weka.classifiers.trees.LADTree.PredictionNode  m_root  
 
 
 
 
 
 
m_lastAddedSplitNum 
 
int  m_lastAddedSplitNum  
 
 
 
 
 
 
m_numericAttIndices 
 
int[]  m_numericAttIndices  
 
 
 
 
 
 
m_search_smallestLeastSquares 
 
double  m_search_smallestLeastSquares  
 
 
 
 
 
 
m_search_bestInsertionNode 
 
weka.classifiers.trees.LADTree.PredictionNode  m_search_bestInsertionNode  
 
 
 
 
 
 
m_search_bestSplitter 
 
weka.classifiers.trees.LADTree.Splitter  m_search_bestSplitter  
 
 
 
 
 
 
m_search_bestPathInstances 
 
 Instances   m_search_bestPathInstances  
 
 
 
 
 
 
m_staticPotentialSplitters2way 
 
 FastVector   m_staticPotentialSplitters2way  
 
 
 
 
 
 
m_nodesExpanded 
 
int  m_nodesExpanded  
 
 
 
 
 
 
m_examplesCounted 
 
int  m_examplesCounted  
 
 
 
 
 
 
m_boostingIterations 
 
int  m_boostingIterations  
 
 
 
 

 
   
 
 
  
 Class weka.classifiers.trees.LADTree.LADInstance extends  Instance  implements Serializable   
 
 

 
   
 
 
  
 Serialized Fields   
 
 

 
fVector 
 
double[]  fVector  
 
 
 
 
 
 
wVector 
 
double[]  wVector  
 
 
 
 
 
 
pVector 
 
double[]  pVector  
 
 
 
 
 
 
zVector 
 
double[]  zVector  
 
 
 
 

 
   
 
 
  
 Class weka.classifiers.trees.LADTree.PredictionNode extends java.lang.Object implements Serializable   
 
 

 
   
 
 
  
 Serialized Fields   
 
 

 
values 
 
double[]  values  
 
 
 
 
 
 
children 
 
 FastVector   children  
 
 
 
 

 
   
 
 
  
 Class weka.classifiers.trees.LADTree.Splitter extends java.lang.Object implements Serializable   
 
 

 
   
 
 
  
 Serialized Fields   
 
 

 
attIndex 
 
int  attIndex  
 
 
 
 
 
 
orderAdded 
 
int  orderAdded  
 
 
 
 

 
   
 
 
  
 Class weka.classifiers.trees.LADTree.TwoWayNominalSplit extends weka.classifiers.trees.LADTree.Splitter implements Serializable   
 
 

 
   
 
 
  
 Serialized Fields   
 
 

 
trueSplitValue 
 
int  trueSplitValue  
 
 
 
 
 
 
children 
 
weka.classifiers.trees.LADTree.PredictionNode[]  children  
 
 
 
 

 
   
 
 
  
 Class weka.classifiers.trees.LADTree.TwoWayNumericSplit extends weka.classifiers.trees.LADTree.Splitter implements Serializable   
 
 

 
   
 
 
  
 Serialized Fields   
 
 

 
splitPoint 
 
double  splitPoint  
 
 
 
 
 
 
children 
 
weka.classifiers.trees.LADTree.PredictionNode[]  children  
 
 
 
 

 
   
 
 
  
 Class  weka.classifiers.trees.LMT  extends  Classifier  implements Serializable   
 
 

 
 serialVersionUID:  -1113212459618104943L

 
   
 
 
  
 Serialized Fields   
 
 

 
m_replaceMissing 
 
 ReplaceMissingValues   m_replaceMissing  
 
 Filter to replace missing values
 
 
 
 
 
 
m_nominalToBinary 
 
 NominalToBinary   m_nominalToBinary  
 
 Filter to replace nominal attributes
 
 
 
 
 
 
m_tree 
 
 LMTNode   m_tree  
 
 root of the logistic model tree
 
 
 
 
 
 
m_fastRegression 
 
boolean  m_fastRegression  
 
 use heuristic that determines the number of LogitBoost iterations only once in the beginning?
 
 
 
 
 
 
m_convertNominal 
 
boolean  m_convertNominal  
 
 convert nominal attributes to binary ?
 
 
 
 
 
 
m_splitOnResiduals 
 
boolean  m_splitOnResiduals  
 
 split on residuals?
 
 
 
 
 
 
m_errorOnProbabilities 
 
boolean  m_errorOnProbabilities  
 
 use error on probabilties instead of misclassification for stopping criterion of LogitBoost?
 
 
 
 
 
 
m_minNumInstances 
 
int  m_minNumInstances  
 
 minimum number of instances at which a node is considered for splitting
 
 
 
 
 
 
m_numBoostingIterations 
 
int  m_numBoostingIterations  
 
 if non-zero, use fixed number of iterations for LogitBoost
 
 
 
 
 
 
m_weightTrimBeta 
 
double  m_weightTrimBeta  
 
 Threshold for trimming weights. Instances with a weight lower than this (as a percentage
 of total weights) are not included in the regression fit.
 
 
 
 
 
 
m_useAIC 
 
boolean  m_useAIC  
 
 If true, the AIC is used to choose the best LogitBoost iteration
 
 
 
 

 
   
 
 
  
 Class  weka.classifiers.trees.M5P  extends  M5Base  implements Serializable   
 
 

 
 serialVersionUID:  -6118439039768244417L

 

 
   
 
 
  
 Class  weka.classifiers.trees.NBTree  extends  Classifier  implements Serializable   
 
 

 
 serialVersionUID:  -4716005707058256086L

 
   
 
 
  
 Serialized Fields   
 
 

 
m_minNumObj 
 
int  m_minNumObj  
 
 Minimum number of instances
 
 
 
 
 
 
m_root 
 
 NBTreeClassifierTree   m_root  
 
 The root of the tree
 
 
 
 

 
   
 
 
  
 Class  weka.classifiers.trees.RandomForest  extends  Classifier  implements Serializable   
 
 

 
 serialVersionUID:  4216839470751428698L

 
   
 
 
  
 Serialized Fields   
 
 

 
m_numTrees 
 
int  m_numTrees  
 
 Number of trees in forest.
 
 
 
 
 
 
m_numFeatures 
 
int  m_numFeatures  
 
 Number of features to consider in random feature selection.
      If less than 1 will use int(logM+1) )
 
 
 
 
 
 
m_randomSeed 
 
int  m_randomSeed  
 
 The random seed.
 
 
 
 
 
 
m_KValue 
 
int  m_KValue  
 
 Final number of features that were considered in last build.
 
 
 
 
 
 
m_bagger 
 
 Bagging   m_bagger  
 
 The bagger.
 
 
 
 
 
 
m_MaxDepth 
 
int  m_MaxDepth  
 
 The maximum depth of the trees (0 = unlimited)
 
 
 
 

 
   
 
 
  
 Class  weka.classifiers.trees.RandomTree  extends  Classifier  implements Serializable   
 
 

 
 serialVersionUID:  8934314652175299374L

 
   
 
 
  
 Serialized Fields   
 
 

 
m_Successors 
 
 RandomTree []  m_Successors  
 
 The subtrees appended to this tree.
 
 
 
 
 
 
m_Attribute 
 
int  m_Attribute  
 
 The attribute to split on.
 
 
 
 
 
 
m_SplitPoint 
 
double  m_SplitPoint  
 
 The split point.
 
 
 
 
 
 
m_Info 
 
 Instances   m_Info  
 
 The header information.
 
 
 
 
 
 
m_Prop 
 
double[]  m_Prop  
 
 The proportions of training instances going down each branch.
 
 
 
 
 
 
m_ClassDistribution 
 
double[]  m_ClassDistribution  
 
 Class probabilities from the training data.
 
 
 
 
 
 
m_MinNum 
 
double  m_MinNum  
 
 Minimum number of instances for leaf.
 
 
 
 
 
 
m_KValue 
 
int  m_KValue  
 
 The number of attributes considered for a split.
 
 
 
 
 
 
m_randomSeed 
 
int  m_randomSeed  
 
 The random seed to use.
 
 
 
 
 
 
m_MaxDepth 
 
int  m_MaxDepth  
 
 The maximum depth of the tree (0 = unlimited)
 
 
 
 
 
 
m_NumFolds 
 
int  m_NumFolds  
 
 Determines how much data is used for backfitting
 
 
 
 
 
 
m_AllowUnclassifiedInstances 
 
boolean  m_AllowUnclassifiedInstances  
 
 Whether unclassified instances are allowed
 
 
 
 
 
 
m_ZeroR 
 
 Classifier   m_ZeroR  
 
 a ZeroR model in case no model can be built from the data
 
 
 
 

 
   
 
 
  
 Class  weka.classifiers.trees.REPTree  extends  Classifier  implements Serializable   
 
 

 
 serialVersionUID:  -9216785998198681299L

 
   
 
 
  
 Serialized Fields   
 
 

 
m_zeroR 
 
 ZeroR   m_zeroR  
 
 ZeroR model that is used if no attributes are present.
 
 
 
 
 
 
m_Tree 
 
weka.classifiers.trees.REPTree.Tree  m_Tree  
 
 The Tree object
 
 
 
 
 
 
m_NumFolds 
 
int  m_NumFolds  
 
 Number of folds for reduced error pruning.
 
 
 
 
 
 
m_Seed 
 
int  m_Seed  
 
 Seed for random data shuffling.
 
 
 
 
 
 
m_NoPruning 
 
boolean  m_NoPruning  
 
 Don't prune
 
 
 
 
 
 
m_MinNum 
 
double  m_MinNum  
 
 The minimum number of instances per leaf.
 
 
 
 
 
 
m_MinVarianceProp 
 
double  m_MinVarianceProp  
 
 The minimum proportion of the total variance (over all the data)
      required for split.
 
 
 
 
 
 
m_MaxDepth 
 
int  m_MaxDepth  
 
 Upper bound on the tree depth
 
 
 
 

 
   
 
 
  
 Class weka.classifiers.trees.REPTree.Tree extends java.lang.Object implements Serializable   
 
 

 
 serialVersionUID:  -1635481717888437935L

 
   
 
 
  
 Serialized Fields   
 
 

 
m_Info 
 
 Instances   m_Info  
 
 The header information (for printing the tree).
 
 
 
 
 
 
m_Successors 
 
weka.classifiers.trees.REPTree.Tree[]  m_Successors  
 
 The subtrees of this tree.
 
 
 
 
 
 
m_Attribute 
 
int  m_Attribute  
 
 The attribute to split on.
 
 
 
 
 
 
m_SplitPoint 
 
double  m_SplitPoint  
 
 The split point.
 
 
 
 
 
 
m_Prop 
 
double[]  m_Prop  
 
 The proportions of training instances going down each branch.
 
 
 
 
 
 
m_ClassProbs 
 
double[]  m_ClassProbs  
 
 Class probabilities from the training data in the nominal case. 
        Holds the mean in the numeric case.
 
 
 
 
 
 
m_Distribution 
 
double[]  m_Distribution  
 
 The (unnormalized) class distribution in the nominal
        case. Holds the sum of squared errors and the weight 
        in the numeric case.
 
 
 
 
 
 
m_HoldOutDist 
 
double[]  m_HoldOutDist  
 
 Class distribution of hold-out set at node in the nominal
        case.  Straight sum of weights plus sum of weighted targets in
        the numeric case (i.e. array has only two elements).
 
 
 
 
 
 
m_HoldOutError 
 
double  m_HoldOutError  
 
 The hold-out error of the node. The number of miss-classified
        instances in the nominal case, the sum of squared errors in the 
        numeric case.
 
 
 
 

 
   
 
 
  
 Class  weka.classifiers.trees.SimpleCart  extends  RandomizableClassifier  implements Serializable   
 
 

 
 serialVersionUID:  4154189200352566053L

 
   
 
 
  
 Serialized Fields   
 
 

 
m_train 
 
 Instances   m_train  
 
 Training data.
 
 
 
 
 
 
m_Successors 
 
 SimpleCart []  m_Successors  
 
 Successor nodes.
 
 
 
 
 
 
m_Attribute 
 
 Attribute   m_Attribute  
 
 Attribute used to split data.
 
 
 
 
 
 
m_SplitValue 
 
double  m_SplitValue  
 
 Split point for a numeric attribute.
 
 
 
 
 
 
m_SplitString 
 
java.lang.String  m_SplitString  
 
 Split subset used to split data for nominal attributes.
 
 
 
 
 
 
m_ClassValue 
 
double  m_ClassValue  
 
 Class value if the node is leaf.
 
 
 
 
 
 
m_ClassAttribute 
 
 Attribute   m_ClassAttribute  
 
 Class attriubte of data.
 
 
 
 
 
 
m_minNumObj 
 
double  m_minNumObj  
 
 Minimum number of instances in at the terminal nodes.
 
 
 
 
 
 
m_numFoldsPruning 
 
int  m_numFoldsPruning  
 
 Number of folds for minimal cost-complexity pruning.
 
 
 
 
 
 
m_Alpha 
 
double  m_Alpha  
 
 Alpha-value (for pruning) at the node.
 
 
 
 
 
 
m_numIncorrectModel 
 
double  m_numIncorrectModel  
 
 Number of training examples misclassified by the model (subtree rooted).
 
 
 
 
 
 
m_numIncorrectTree 
 
double  m_numIncorrectTree  
 
 Number of training examples misclassified by the model (subtree not rooted).
 
 
 
 
 
 
m_isLeaf 
 
boolean  m_isLeaf  
 
 Indicate if the node is a leaf node.
 
 
 
 
 
 
m_Prune 
 
boolean  m_Prune  
 
 If use minimal cost-compexity pruning.
 
 
 
 
 
 
m_totalTrainInstances 
 
int  m_totalTrainInstances  
 
 Total number of instances used to build the classifier.
 
 
 
 
 
 
m_Props 
 
double[]  m_Props  
 
 Proportion for each branch.
 
 
 
 
 
 
m_ClassProbs 
 
double[]  m_ClassProbs  
 
 Class probabilities.
 
 
 
 
 
 
m_Distribution 
 
double[]  m_Distribution  
 
 Distributions of leaf node (or temporary leaf node in minimal cost-complexity pruning)
 
 
 
 
 
 
m_Heuristic 
 
boolean  m_Heuristic  
 
 If use huristic search for nominal attributes in multi-class problems (default true).
 
 
 
 
 
 
m_UseOneSE 
 
boolean  m_UseOneSE  
 
 If use the 1SE rule to make final decision tree.
 
 
 
 
 
 
m_SizePer 
 
double  m_SizePer  
 
 Training data size.
 
 
 
 

 
   
 
 
  
 Class  weka.classifiers.trees.UserClassifier  extends  Classifier  implements Serializable   
 
 

 
 serialVersionUID:  6483901103562809843L

 
   
 
 
  
 Serialized Fields   
 
 

 
m_top 
 
weka.classifiers.trees.UserClassifier.TreeClass  m_top  
 
 Two references to the structure of the decision tree.
 
 
 
 
 
 
m_focus 
 
weka.classifiers.trees.UserClassifier.TreeClass  m_focus  
 
 Two references to the structure of the decision tree.
 
 
 
 
 
 
m_nextId 
 
int  m_nextId  
 
 The next number that can be used as a unique id for a node.
 
 
 
 
 
 
m_built 
 
boolean  m_built  
 
 The status of whether there is a decision tree ready or not.
 
 
 
 
 
 
m_classifiers 
 
 GenericObjectEditor   m_classifiers  
 
 A list of other m_classifiers.
 
 
 
 
 
 
m_propertyDialog 
 
 PropertyDialog   m_propertyDialog  
 
 A window for selecting other classifiers.
 
 
 
 
 

 
 
  
 Package   weka.classifiers.trees.adtree   
 
 

 
   
 
 
  
 Class  weka.classifiers.trees.adtree.PredictionNode  extends java.lang.Object implements Serializable   
 
 

 
 serialVersionUID:  6018958856358698814L

 
   
 
 
  
 Serialized Fields   
 
 

 
value 
 
double  value  
 
 The prediction value stored in this node
 
 
 
 
 
 
children 
 
 FastVector   children  
 
 The children of this node - any number of splitter nodes
 
 
 
 

 
   
 
 
  
 Class  weka.classifiers.trees.adtree.ReferenceInstances  extends  Instances  implements Serializable   
 
 

 
 serialVersionUID:  -8022666381920252997L

 

 
   
 
 
  
 Class  weka.classifiers.trees.adtree.Splitter  extends java.lang.Object implements Serializable   
 
 

 
 serialVersionUID:  8190449848490055L

 
   
 
 
  
 Serialized Fields   
 
 

 
orderAdded 
 
int  orderAdded  
 
 The number this node was in the order of nodes added to the tree
 
 
 
 

 
   
 
 
  
 Class  weka.classifiers.trees.adtree.TwoWayNominalSplit  extends  Splitter  implements Serializable   
 
 

 
 serialVersionUID:  -4598366190152721355L

 
   
 
 
  
 Serialized Fields   
 
 

 
attIndex 
 
int  attIndex  
 
 The index of the attribute the split depends on
 
 
 
 
 
 
trueSplitValue 
 
int  trueSplitValue  
 
 The attribute value that is compared against
 
 
 
 
 
 
children 
 
 PredictionNode []  children  
 
 The children of this split
 
 
 
 

 
   
 
 
  
 Class  weka.classifiers.trees.adtree.TwoWayNumericSplit  extends  Splitter  implements Serializable   
 
 

 
 serialVersionUID:  449769177903158283L

 
   
 
 
  
 Serialized Fields   
 
 

 
attIndex 
 
int  attIndex  
 
 The index of the attribute the split depends on
 
 
 
 
 
 
splitPoint 
 
double  splitPoint  
 
 The attribute value that is compared against
 
 
 
 
 
 
children 
 
 PredictionNode []  children  
 
 The children of this split
 
 
 
 
 

 
 
  
 Package   weka.classifiers.trees.ft   
 
 

 
   
 
 
  
 Class  weka.classifiers.trees.ft.FTInnerNode  extends  FTtree  implements Serializable   
 
 

 
 serialVersionUID:  -1125334488640233181L

 

 
   
 
 
  
 Class  weka.classifiers.trees.ft.FTLeavesNode  extends  FTtree  implements Serializable   
 
 

 
 serialVersionUID:  950601378326259315L

 

 
   
 
 
  
 Class  weka.classifiers.trees.ft.FTNode  extends  FTtree  implements Serializable   
 
 

 
 serialVersionUID:  2317688685139295063L

 

 
   
 
 
  
 Class  weka.classifiers.trees.ft.FTtree  extends  LogisticBase  implements Serializable   
 
 

 
 serialVersionUID:  1862737145870398755L

 
   
 
 
  
 Serialized Fields   
 
 

 
m_totalInstanceWeight 
 
double  m_totalInstanceWeight  
 
 Total number of training instances.
 
 
 
 
 
 
m_id 
 
int  m_id  
 
 Node id
 
 
 
 
 
 
m_leafModelNum 
 
int  m_leafModelNum  
 
 ID of logistic model at leaf
 
 
 
 
 
 
m_minNumInstances 
 
int  m_minNumInstances  
 
 minimum number of instances at which a node is considered for splitting
 
 
 
 
 
 
m_modelSelection 
 
 ModelSelection   m_modelSelection  
 
 ModelSelection object (for splitting)
 
 
 
 
 
 
m_nominalToBinary 
 
 NominalToBinary   m_nominalToBinary  
 
 Filter to convert nominal attributes to binary
 
 
 
 
 
 
m_higherRegressions 
 
 SimpleLinearRegression [][]  m_higherRegressions  
 
 Simple regression functions fit by LogitBoost at higher levels in the tree
 
 
 
 
 
 
m_numHigherRegressions 
 
int  m_numHigherRegressions  
 
 Number of simple regression functions fit by LogitBoost at higher levels in the tree
 
 
 
 
 
 
m_numInstances 
 
int  m_numInstances  
 
 Number of instances at the node
 
 
 
 
 
 
m_localModel 
 
 ClassifierSplitModel   m_localModel  
 
 The ClassifierSplitModel (for splitting)
 
 
 
 
 
 
m_auxLocalModel 
 
 ClassifierSplitModel   m_auxLocalModel  
 
 Auxiliary copy ClassifierSplitModel (for splitting)
 
 
 
 
 
 
m_sons 
 
 FTtree []  m_sons  
 
 Array of children of the node
 
 
 
 
 
 
m_leafclass 
 
int  m_leafclass  
 
 Stores leaf class value
 
 
 
 
 
 
m_isLeaf 
 
boolean  m_isLeaf  
 
 True if node is leaf
 
 
 
 
 
 
m_hasConstr 
 
boolean  m_hasConstr  
 
 True if node has or splits on constructor
 
 
 
 
 
 
m_constError 
 
double  m_constError  
 
 Constructor error
 
 
 
 
 
 
m_CF 
 
float  m_CF  
 
 Confidence level
 
 
 
 
 

 
 
  
 Package   weka.classifiers.trees.j48   
 
 

 
   
 
 
  
 Class  weka.classifiers.trees.j48.BinC45ModelSelection  extends  ModelSelection  implements Serializable   
 
 

 
 serialVersionUID:  179170923545122001L

 
   
 
 
  
 Serialized Fields   
 
 

 
m_minNoObj 
 
int  m_minNoObj  
 
 Minimum number of instances in interval.
 
 
 
 
 
 
m_allData 
 
 Instances   m_allData  
 
 The FULL training dataset.
 
 
 
 

 
   
 
 
  
 Class  weka.classifiers.trees.j48.BinC45Split  extends  ClassifierSplitModel  implements Serializable   
 
 

 
 serialVersionUID:  -1278776919563022474L

 
   
 
 
  
 Serialized Fields   
 
 

 
m_attIndex 
 
int  m_attIndex  
 
 Attribute to split on.
 
 
 
 
 
 
m_minNoObj 
 
int  m_minNoObj  
 
 Minimum number of objects in a split.
 
 
 
 
 
 
m_splitPoint 
 
double  m_splitPoint  
 
 Value of split point.
 
 
 
 
 
 
m_infoGain 
 
double  m_infoGain  
 
 InfoGain of split.
 
 
 
 
 
 
m_gainRatio 
 
double  m_gainRatio  
 
 GainRatio of split.
 
 
 
 
 
 
m_sumOfWeights 
 
double  m_sumOfWeights  
 
 The sum of the weights of the instances.
 
 
 
 

 
   
 
 
  
 Class  weka.classifiers.trees.j48.C45ModelSelection  extends  ModelSelection  implements Serializable   
 
 

 
 serialVersionUID:  3372204862440821989L

 
   
 
 
  
 Serialized Fields   
 
 

 
m_minNoObj 
 
int  m_minNoObj  
 
 Minimum number of objects in interval.
 
 
 
 
 
 
m_allData 
 
 Instances   m_allData  
 
 All the training data
 
 
 
 

 
   
 
 
  
 Class  weka.classifiers.trees.j48.C45PruneableClassifierTree  extends  ClassifierTree  implements Serializable   
 
 

 
 serialVersionUID:  -4813820170260388194L

 
   
 
 
  
 Serialized Fields   
 
 

 
m_pruneTheTree 
 
boolean  m_pruneTheTree  
 
 True if the tree is to be pruned.
 
 
 
 
 
 
m_CF 
 
float  m_CF  
 
 The confidence factor for pruning.
 
 
 
 
 
 
m_subtreeRaising 
 
boolean  m_subtreeRaising  
 
 Is subtree raising to be performed?
 
 
 
 
 
 
m_cleanup 
 
boolean  m_cleanup  
 
 Cleanup after the tree has been built.
 
 
 
 

 
   
 
 
  
 Class  weka.classifiers.trees.j48.C45PruneableClassifierTreeG  extends  ClassifierTree  implements Serializable   
 
 

 
 serialVersionUID:  66981207374331964L

 
   
 
 
  
 Serialized Fields   
 
 

 
m_pruneTheTree 
 
boolean  m_pruneTheTree  
 
 True if the tree is to be pruned.
 
 
 
 
 
 
m_CF 
 
float  m_CF  
 
 The confidence factor for pruning.
 
 
 
 
 
 
m_subtreeRaising 
 
boolean  m_subtreeRaising  
 
 Is subtree raising to be performed?
 
 
 
 
 
 
m_cleanup 
 
boolean  m_cleanup  
 
 Cleanup after the tree has been built.
 
 
 
 
 
 
m_relabel 
 
boolean  m_relabel  
 
 flag for using relabelling when grafting
 
 
 
 
 
 
m_BiProbCrit 
 
double  m_BiProbCrit  
 
 binomial probability critical value
 
 
 
 
 
 
m_Debug 
 
boolean  m_Debug  
 
 
 
 

 
   
 
 
  
 Class  weka.classifiers.trees.j48.C45Split  extends  ClassifierSplitModel  implements Serializable   
 
 

 
 serialVersionUID:  3064079330067903161L

 
   
 
 
  
 Serialized Fields   
 
 

 
m_complexityIndex 
 
int  m_complexityIndex  
 
 Desired number of branches.
 
 
 
 
 
 
m_attIndex 
 
int  m_attIndex  
 
 Attribute to split on.
 
 
 
 
 
 
m_minNoObj 
 
int  m_minNoObj  
 
 Minimum number of objects in a split.
 
 
 
 
 
 
m_splitPoint 
 
double  m_splitPoint  
 
 Value of split point.
 
 
 
 
 
 
m_infoGain 
 
double  m_infoGain  
 
 InfoGain of split.
 
 
 
 
 
 
m_gainRatio 
 
double  m_gainRatio  
 
 GainRatio of split.
 
 
 
 
 
 
m_sumOfWeights 
 
double  m_sumOfWeights  
 
 The sum of the weights of the instances.
 
 
 
 
 
 
m_index 
 
int  m_index  
 
 Number of split points.
 
 
 
 

 
   
 
 
  
 Class  weka.classifiers.trees.j48.ClassifierSplitModel  extends java.lang.Object implements Serializable   
 
 

 
 serialVersionUID:  4280730118393457457L

 
   
 
 
  
 Serialized Fields   
 
 

 
m_distribution 
 
 Distribution   m_distribution  
 
 Distribution of class values.
 
 
 
 
 
 
m_numSubsets 
 
int  m_numSubsets  
 
 Number of created subsets.
 
 
 
 

 
   
 
 
  
 Class  weka.classifiers.trees.j48.ClassifierTree  extends java.lang.Object implements Serializable   
 
 

 
 serialVersionUID:  -8722249377542734193L

 
   
 
 
  
 Serialized Fields   
 
 

 
m_toSelectModel 
 
 ModelSelection   m_toSelectModel  
 
 The model selection method.
 
 
 
 
 
 
m_localModel 
 
 ClassifierSplitModel   m_localModel  
 
 Local model at node.
 
 
 
 
 
 
m_sons 
 
 ClassifierTree []  m_sons  
 
 References to sons.
 
 
 
 
 
 
m_isLeaf 
 
boolean  m_isLeaf  
 
 True if node is leaf.
 
 
 
 
 
 
m_isEmpty 
 
boolean  m_isEmpty  
 
 True if node is empty.
 
 
 
 
 
 
m_train 
 
 Instances   m_train  
 
 The training instances.
 
 
 
 
 
 
m_test 
 
 Distribution   m_test  
 
 The pruning instances.
 
 
 
 
 
 
m_id 
 
int  m_id  
 
 The id for the node.
 
 
 
 

 
   
 
 
  
 Class  weka.classifiers.trees.j48.Distribution  extends java.lang.Object implements Serializable   
 
 

 
 serialVersionUID:  8526859638230806576L

 
   
 
 
  
 Serialized Fields   
 
 

 
m_perClassPerBag 
 
double[][]  m_perClassPerBag  
 
 Weight of instances per class per bag.
 
 
 
 
 
 
m_perBag 
 
double[]  m_perBag  
 
 Weight of instances per bag.
 
 
 
 
 
 
m_perClass 
 
double[]  m_perClass  
 
 Weight of instances per class.
 
 
 
 
 
 
totaL 
 
double  totaL  
 
 Total weight of instances.
 
 
 
 

 
   
 
 
  
 Class  weka.classifiers.trees.j48.EntropyBasedSplitCrit  extends  SplitCriterion  implements Serializable   
 
 

 
 serialVersionUID:  -2618691439791653056L

 

 
   
 
 
  
 Class  weka.classifiers.trees.j48.EntropySplitCrit  extends  EntropyBasedSplitCrit  implements Serializable   
 
 

 
 serialVersionUID:  5986252682266803935L

 

 
   
 
 
  
 Class  weka.classifiers.trees.j48.GainRatioSplitCrit  extends  EntropyBasedSplitCrit  implements Serializable   
 
 

 
 serialVersionUID:  -433336694718670930L

 

 
   
 
 
  
 Class  weka.classifiers.trees.j48.GraftSplit  extends  ClassifierSplitModel  implements Serializable   
 
 

 
 serialVersionUID:  722773260393182051L

 
   
 
 
  
 Serialized Fields   
 
 

 
m_graftdistro 
 
 Distribution   m_graftdistro  
 
 the distribution for graft values, from cases in atbop
 
 
 
 
 
 
m_attIndex 
 
int  m_attIndex  
 
 the attribute we are splitting on
 
 
 
 
 
 
m_splitPoint 
 
double  m_splitPoint  
 
 value of split point (if numeric attribute)
 
 
 
 
 
 
m_maxClass 
 
int  m_maxClass  
 
 dominant class of the subset specified by m_testType
 
 
 
 
 
 
m_otherLeafMaxClass 
 
int  m_otherLeafMaxClass  
 
 dominant class of the subset not specified by m_testType
 
 
 
 
 
 
m_laplace 
 
double  m_laplace  
 
 laplace value of the subset specified by m_testType for m_maxClass
 
 
 
 
 
 
m_leafdistro 
 
 Distribution   m_leafdistro  
 
 leaf for the subset specified by m_testType
 
 
 
 
 
 
m_testType 
 
int  m_testType  
 
 type of test:
 0:   test
 2: = test
 3: != test
 
 
 
 

 
   
 
 
  
 Class  weka.classifiers.trees.j48.InfoGainSplitCrit  extends  EntropyBasedSplitCrit  implements Serializable   
 
 

 
 serialVersionUID:  4892105020180728499L

 

 
   
 
 
  
 Class  weka.classifiers.trees.j48.ModelSelection  extends java.lang.Object implements Serializable   
 
 

 
 serialVersionUID:  -4850147125096133642L

 

 
   
 
 
  
 Class  weka.classifiers.trees.j48.NBTreeClassifierTree  extends  ClassifierTree  implements Serializable   
 
 

 
 serialVersionUID:  -4472639447877404786L

 

 
   
 
 
  
 Class  weka.classifiers.trees.j48.NBTreeModelSelection  extends  ModelSelection  implements Serializable   
 
 

 
 serialVersionUID:  990097748931976704L

 
   
 
 
  
 Serialized Fields   
 
 

 
m_minNoObj 
 
int  m_minNoObj  
 
 Minimum number of objects in interval.
 
 
 
 
 
 
m_allData 
 
 Instances   m_allData  
 
 All the training data
 
 
 
 

 
   
 
 
  
 Class  weka.classifiers.trees.j48.NBTreeNoSplit  extends  ClassifierSplitModel  implements Serializable   
 
 

 
 serialVersionUID:  7824804381545259618L

 
   
 
 
  
 Serialized Fields   
 
 

 
m_nb 
 
 NaiveBayesUpdateable   m_nb  
 
 the naive bayes classifier
 
 
 
 
 
 
m_disc 
 
 Discretize   m_disc  
 
 the discretizer used
 
 
 
 
 
 
m_errors 
 
double  m_errors  
 
 errors on the training data at this node
 
 
 
 

 
   
 
 
  
 Class  weka.classifiers.trees.j48.NBTreeSplit  extends  ClassifierSplitModel  implements Serializable   
 
 

 
 serialVersionUID:  8922627123884975070L

 
   
 
 
  
 Serialized Fields   
 
 

 
m_complexityIndex 
 
int  m_complexityIndex  
 
 Desired number of branches.
 
 
 
 
 
 
m_attIndex 
 
int  m_attIndex  
 
 Attribute to split on.
 
 
 
 
 
 
m_minNoObj 
 
int  m_minNoObj  
 
 Minimum number of objects in a split.
 
 
 
 
 
 
m_splitPoint 
 
double  m_splitPoint  
 
 Value of split point.
 
 
 
 
 
 
m_sumOfWeights 
 
double  m_sumOfWeights  
 
 The sum of the weights of the instances.
 
 
 
 
 
 
m_errors 
 
double  m_errors  
 
 The weight of the instances incorrectly classified by the 
      naive bayes models arising from this split
 
 
 
 
 
 
m_c45S 
 
 C45Split   m_c45S  
 
 
 
 
 
 
m_globalNB 
 
 NBTreeNoSplit   m_globalNB  
 
 The global naive bayes model for this node
 
 
 
 

 
   
 
 
  
 Class  weka.classifiers.trees.j48.NoSplit  extends  ClassifierSplitModel  implements Serializable   
 
 

 
 serialVersionUID:  -1292620749331337546L

 

 
   
 
 
  
 Class  weka.classifiers.trees.j48.PruneableClassifierTree  extends  ClassifierTree  implements Serializable   
 
 

 
 serialVersionUID:  -555775736857600201L

 
   
 
 
  
 Serialized Fields   
 
 

 
pruneTheTree 
 
boolean  pruneTheTree  
 
 True if the tree is to be pruned.
 
 
 
 
 
 
numSets 
 
int  numSets  
 
 How many subsets of equal size? One used for pruning, the rest for training.
 
 
 
 
 
 
m_cleanup 
 
boolean  m_cleanup  
 
 Cleanup after the tree has been built.
 
 
 
 
 
 
m_seed 
 
int  m_seed  
 
 The random number seed.
 
 
 
 

 
   
 
 
  
 Class  weka.classifiers.trees.j48.SplitCriterion  extends java.lang.Object implements Serializable   
 
 

 
 serialVersionUID:  5490996638027101259L

 
 

 
 
  
 Package   weka.classifiers.trees.lmt   
 
 

 
   
 
 
  
 Class  weka.classifiers.trees.lmt.LMTNode  extends  LogisticBase  implements Serializable   
 
 

 
 serialVersionUID:  1862737145870398755L

 
   
 
 
  
 Serialized Fields   
 
 

 
m_totalInstanceWeight 
 
double  m_totalInstanceWeight  
 
 Total number of training instances.
 
 
 
 
 
 
m_id 
 
int  m_id  
 
 Node id
 
 
 
 
 
 
m_leafModelNum 
 
int  m_leafModelNum  
 
 ID of logistic model at leaf
 
 
 
 
 
 
m_alpha 
 
double  m_alpha  
 
 Alpha-value (for pruning) at the node
 
 
 
 
 
 
m_numIncorrectModel 
 
double  m_numIncorrectModel  
 
 Weighted number of training examples currently misclassified by the logistic model at the node
 
 
 
 
 
 
m_numIncorrectTree 
 
double  m_numIncorrectTree  
 
 Weighted number of training examples currently misclassified by the subtree rooted at the node
 
 
 
 
 
 
m_minNumInstances 
 
int  m_minNumInstances  
 
 minimum number of instances at which a node is considered for splitting
 
 
 
 
 
 
m_modelSelection 
 
 ModelSelection   m_modelSelection  
 
 ModelSelection object (for splitting)
 
 
 
 
 
 
m_nominalToBinary 
 
 NominalToBinary   m_nominalToBinary  
 
 Filter to convert nominal attributes to binary
 
 
 
 
 
 
m_higherRegressions 
 
 SimpleLinearRegression [][]  m_higherRegressions  
 
 Simple regression functions fit by LogitBoost at higher levels in the tree
 
 
 
 
 
 
m_numHigherRegressions 
 
int  m_numHigherRegressions  
 
 Number of simple regression functions fit by LogitBoost at higher levels in the tree
 
 
 
 
 
 
m_fastRegression 
 
boolean  m_fastRegression  
 
 Use heuristic that determines the number of LogitBoost iterations only once in the beginning?
 
 
 
 
 
 
m_numInstances 
 
int  m_numInstances  
 
 Number of instances at the node
 
 
 
 
 
 
m_localModel 
 
 ClassifierSplitModel   m_localModel  
 
 The ClassifierSplitModel (for splitting)
 
 
 
 
 
 
m_sons 
 
 LMTNode []  m_sons  
 
 Array of children of the node
 
 
 
 
 
 
m_isLeaf 
 
boolean  m_isLeaf  
 
 True if node is leaf
 
 
 
 

 
   
 
 
  
 Class  weka.classifiers.trees.lmt.LogisticBase  extends  Classifier  implements Serializable   
 
 

 
 serialVersionUID:  168765678097825064L

 
   
 
 
  
 Serialized Fields   
 
 

 
m_numericDataHeader 
 
 Instances   m_numericDataHeader  
 
 Header-only version of the numeric version of the training data
 
 
 
 
 
 
m_numericData 
 
 Instances   m_numericData  
 
 Numeric version of the training data. Original class is replaced by a numeric pseudo-class.
 
 
 
 
 
 
m_train 
 
 Instances   m_train  
 
 Training data
 
 
 
 
 
 
m_useCrossValidation 
 
boolean  m_useCrossValidation  
 
 Use cross-validation to determine best number of LogitBoost iterations ?
 
 
 
 
 
 
m_errorOnProbabilities 
 
boolean  m_errorOnProbabilities  
 
 Use error on probabilities for stopping criterion of LogitBoost?
 
 
 
 
 
 
m_fixedNumIterations 
 
int  m_fixedNumIterations  
 
 Use fixed number of iterations for LogitBoost? (if negative, cross-validate number of iterations)
 
 
 
 
 
 
m_heuristicStop 
 
int  m_heuristicStop  
 
 Use heuristic to stop performing LogitBoost iterations earlier?
 If enabled, LogitBoost is stopped if the current (local) minimum of the error on a test set as 
 a function of the number of iterations has not changed for m_heuristicStop iterations.
 
 
 
 
 
 
m_numRegressions 
 
int  m_numRegressions  
 
 The number of LogitBoost iterations performed.
 
 
 
 
 
 
m_maxIterations 
 
int  m_maxIterations  
 
 The maximum number of LogitBoost iterations
 
 
 
 
 
 
m_numClasses 
 
int  m_numClasses  
 
 The number of different classes
 
 
 
 
 
 
m_regressions 
 
 SimpleLinearRegression [][]  m_regressions  
 
 Array holding the simple regression functions fit by LogitBoost
 
 
 
 
 
 
m_useAIC 
 
boolean  m_useAIC  
 
 If true, the AIC is used to choose the best iteration
 
 
 
 
 
 
m_numParameters 
 
double  m_numParameters  
 
 Effective number of parameters used for AIC / BIC automatic stopping
 
 
 
 
 
 
m_weightTrimBeta 
 
double  m_weightTrimBeta  
 
 Threshold for trimming weights. Instances with a weight lower than this (as a percentage
 of total weights) are not included in the regression fit.
 
 
 
 

 
   
 
 
  
 Class  weka.classifiers.trees.lmt.ResidualModelSelection  extends  ModelSelection  implements Serializable   
 
 

 
 serialVersionUID:  -293098783159385148L

 
   
 
 
  
 Serialized Fields   
 
 

 
m_minNumInstances 
 
int  m_minNumInstances  
 
 Minimum number of instances for leaves
 
 
 
 
 
 
m_minInfoGain 
 
double  m_minInfoGain  
 
 Minimum information gain for split
 
 
 
 

 
   
 
 
  
 Class  weka.classifiers.trees.lmt.ResidualSplit  extends  ClassifierSplitModel  implements Serializable   
 
 

 
 serialVersionUID:  -5055883734183713525L

 
   
 
 
  
 Serialized Fields   
 
 

 
m_attribute 
 
 Attribute   m_attribute  
 
 The attribute selected for the split
 
 
 
 
 
 
m_attIndex 
 
int  m_attIndex  
 
 The index of the attribute selected for the split
 
 
 
 
 
 
m_numInstances 
 
int  m_numInstances  
 
 Number of instances in the set
 
 
 
 
 
 
m_numClasses 
 
int  m_numClasses  
 
 Number of classed
 
 
 
 
 
 
m_data 
 
 Instances   m_data  
 
 The set of instances
 
 
 
 
 
 
m_dataZs 
 
double[][]  m_dataZs  
 
 The Z-values (LogitBoost response) for the set of instances
 
 
 
 
 
 
m_dataWs 
 
double[][]  m_dataWs  
 
 The LogitBoost-weights for the set of instances
 
 
 
 
 
 
m_splitPoint 
 
double  m_splitPoint  
 
 The split point (for numeric attributes)
 
 
 
 
 

 
 
  
 Package   weka.classifiers.trees.m5   
 
 

 
   
 
 
  
 Class  weka.classifiers.trees.m5.CorrelationSplitInfo  extends java.lang.Object implements Serializable   
 
 

 
 serialVersionUID:  4212734895125452770L

 
   
 
 
  
 Serialized Fields   
 
 

 
m_first 
 
int  m_first  
 
 the first instance
 
 
 
 
 
 
m_last 
 
int  m_last  
 
 the last instance
 
 
 
 
 
 
m_position 
 
int  m_position  
 
 
 
 
 
 
m_maxImpurity 
 
double  m_maxImpurity  
 
 the maximum impurity reduction
 
 
 
 
 
 
m_splitAttr 
 
int  m_splitAttr  
 
 the attribute being tested
 
 
 
 
 
 
m_splitValue 
 
double  m_splitValue  
 
 the best value on which to split
 
 
 
 
 
 
m_number 
 
int  m_number  
 
 the number of instances
 
 
 
 

 
   
 
 
  
 Class  weka.classifiers.trees.m5.M5Base  extends  Classifier  implements Serializable   
 
 

 
 serialVersionUID:  -4022221950191647679L

 
   
 
 
  
 Serialized Fields   
 
 

 
m_instances 
 
 Instances   m_instances  
 
 the instances covered by the tree/rules
 
 
 
 
 
 
m_ruleSet 
 
 FastVector   m_ruleSet  
 
 the rule set
 
 
 
 
 
 
m_generateRules 
 
boolean  m_generateRules  
 
 generate a decision list instead of a single tree.
 
 
 
 
 
 
m_unsmoothedPredictions 
 
boolean  m_unsmoothedPredictions  
 
 use unsmoothed predictions
 
 
 
 
 
 
m_replaceMissing 
 
 ReplaceMissingValues   m_replaceMissing  
 
 filter to fill in missing values
 
 
 
 
 
 
m_nominalToBinary 
 
 NominalToBinary   m_nominalToBinary  
 
 filter to convert nominal attributes to binary
 
 
 
 
 
 
m_removeUseless 
 
 RemoveUseless   m_removeUseless  
 
 for removing useless attributes
 
 
 
 
 
 
m_saveInstances 
 
boolean  m_saveInstances  
 
 Save instances at each node in an M5 tree for visualization purposes.
 
 
 
 
 
 
m_regressionTree 
 
boolean  m_regressionTree  
 
 Make a regression tree/rule instead of a model tree/rule
 
 
 
 
 
 
m_useUnpruned 
 
boolean  m_useUnpruned  
 
 Do not prune tree/rules
 
 
 
 
 
 
m_minNumInstances 
 
double  m_minNumInstances  
 
 The minimum number of instances to allow at a leaf node
 
 
 
 

 
   
 
 
  
 Class  weka.classifiers.trees.m5.PreConstructedLinearModel  extends  Classifier  implements Serializable   
 
 

 
 serialVersionUID:  2030974097051713247L

 
   
 
 
  
 Serialized Fields   
 
 

 
m_coefficients 
 
double[]  m_coefficients  
 
 The coefficients
 
 
 
 
 
 
m_intercept 
 
double  m_intercept  
 
 The intercept
 
 
 
 
 
 
m_instancesHeader 
 
 Instances   m_instancesHeader  
 
 Holds the instances header for printing the model
 
 
 
 
 
 
m_numParameters 
 
int  m_numParameters  
 
 number of coefficients in the model
 
 
 
 

 
   
 
 
  
 Class  weka.classifiers.trees.m5.Rule  extends java.lang.Object implements Serializable   
 
 

 
 serialVersionUID:  -4458627451682483204L

 
   
 
 
  
 Serialized Fields   
 
 

 
m_instances 
 
 Instances   m_instances  
 
 the instances covered by this rule
 
 
 
 
 
 
m_classIndex 
 
int  m_classIndex  
 
 the class index
 
 
 
 
 
 
m_numAttributes 
 
int  m_numAttributes  
 
 the number of attributes
 
 
 
 
 
 
m_numInstances 
 
int  m_numInstances  
 
 the number of instances in the dataset
 
 
 
 
 
 
m_splitAtts 
 
int[]  m_splitAtts  
 
 the indexes of the attributes used to split on for this rule
 
 
 
 
 
 
m_splitVals 
 
double[]  m_splitVals  
 
 the corresponding values of the split points
 
 
 
 
 
 
m_internalNodes 
 
 RuleNode []  m_internalNodes  
 
 the corresponding internal nodes. Used for smoothing rules.
 
 
 
 
 
 
m_relOps 
 
int[]  m_relOps  
 
 the corresponding relational operators (0 = " ")
 
 
 
 
 
 
m_ruleModel 
 
 RuleNode   m_ruleModel  
 
 the leaf encapsulating the linear model for this rule
 
 
 
 
 
 
m_topOfTree 
 
 RuleNode   m_topOfTree  
 
 the top of the m5 tree for this rule
 
 
 
 
 
 
m_globalStdDev 
 
double  m_globalStdDev  
 
 the standard deviation of the class for all the instances
 
 
 
 
 
 
m_globalAbsDev 
 
double  m_globalAbsDev  
 
 the absolute deviation of the class for all the instances
 
 
 
 
 
 
m_covered 
 
 Instances   m_covered  
 
 the instances covered by this rule
 
 
 
 
 
 
m_numCovered 
 
int  m_numCovered  
 
 the number of instances covered by this rule
 
 
 
 
 
 
m_notCovered 
 
 Instances   m_notCovered  
 
 the instances not covered by this rule
 
 
 
 
 
 
m_useTree 
 
boolean  m_useTree  
 
 use a pruned m5 tree rather than make a rule
 
 
 
 
 
 
m_smoothPredictions 
 
boolean  m_smoothPredictions  
 
 use the original m5 smoothing procedure
 
 
 
 
 
 
m_saveInstances 
 
boolean  m_saveInstances  
 
 Save instances at each node in an M5 tree for visualization purposes.
 
 
 
 
 
 
m_regressionTree 
 
boolean  m_regressionTree  
 
 Make a regression tree instead of a model tree
 
 
 
 
 
 
m_useUnpruned 
 
boolean  m_useUnpruned  
 
 Build unpruned tree/rule
 
 
 
 
 
 
m_minNumInstances 
 
double  m_minNumInstances  
 
 The minimum number of instances to allow at a leaf node
 
 
 
 

 
   
 
 
  
 Class  weka.classifiers.trees.m5.RuleNode  extends  Classifier  implements Serializable   
 
 

 
 serialVersionUID:  1979807611124337144L

 
   
 
 
  
 Serialized Fields   
 
 

 
m_instances 
 
 Instances   m_instances  
 
 instances reaching this node
 
 
 
 
 
 
m_classIndex 
 
int  m_classIndex  
 
 the class index
 
 
 
 
 
 
m_numInstances 
 
int  m_numInstances  
 
 the number of instances reaching this node
 
 
 
 
 
 
m_numAttributes 
 
int  m_numAttributes  
 
 the number of attributes
 
 
 
 
 
 
m_isLeaf 
 
boolean  m_isLeaf  
 
 Node is a leaf
 
 
 
 
 
 
m_splitAtt 
 
int  m_splitAtt  
 
 attribute this node splits on
 
 
 
 
 
 
m_splitValue 
 
double  m_splitValue  
 
 the value of the split attribute
 
 
 
 
 
 
m_nodeModel 
 
 PreConstructedLinearModel   m_nodeModel  
 
 the linear model at this node
 
 
 
 
 
 
m_numParameters 
 
int  m_numParameters  
 
 the number of paramters in the chosen model for this node---either
 the subtree model or the linear model.
 The constant term is counted as a paramter---this is for pruning
 purposes
 
 
 
 
 
 
m_rootMeanSquaredError 
 
double  m_rootMeanSquaredError  
 
 the mean squared error of the model at this node (either linear or
 subtree)
 
 
 
 
 
 
m_left 
 
 RuleNode   m_left  
 
 left child node
 
 
 
 
 
 
m_right 
 
 RuleNode   m_right  
 
 right child node
 
 
 
 
 
 
m_parent 
 
 RuleNode   m_parent  
 
 the parent of this node
 
 
 
 
 
 
m_splitNum 
 
double  m_splitNum  
 
 a node will not be split if it contains less then m_splitNum instances
 
 
 
 
 
 
m_devFraction 
 
double  m_devFraction  
 
 a node will not be split if its class standard deviation is less
 than 5% of the class standard deviation of all the instances
 
 
 
 
 
 
m_pruningMultiplier 
 
double  m_pruningMultiplier  
 
 
 
 
 
 
m_leafModelNum 
 
int  m_leafModelNum  
 
 the number assigned to the linear model if this node is a leaf.
 = 0 if this node is not a leaf
 
 
 
 
 
 
m_globalDeviation 
 
double  m_globalDeviation  
 
 a node will not be split if the class deviation of its
 instances is less than m_devFraction of the deviation of the
 global class
 
 
 
 
 
 
m_globalAbsDeviation 
 
double  m_globalAbsDeviation  
 
 the absolute deviation of the global class
 
 
 
 
 
 
m_indices 
 
int[]  m_indices  
 
 Indices of the attributes to be used in generating a linear model
 at this node
 
 
 
 
 
 
m_id 
 
int  m_id  
 
 Node id.
 
 
 
 
 
 
m_saveInstances 
 
boolean  m_saveInstances  
 
 Save the instances at each node (for visualizing in the
 Explorer's treevisualizer.
 
 
 
 
 
 
m_regressionTree 
 
boolean  m_regressionTree  
 
 Make a regression tree instead of a model tree
 
 
 
 

 
   
 
 
  
 Class  weka.classifiers.trees.m5.YongSplitInfo  extends java.lang.Object implements Serializable   
 
 

 
 serialVersionUID:  1864267581079767881L

 
   
 
 
  
 Serialized Fields   
 
 

 
number 
 
int  number  
 
 
 
 
 
 
first 
 
int  first  
 
 
 
 
 
 
last 
 
int  last  
 
 
 
 
 
 
position 
 
int  position  
 
 
 
 
 
 
maxImpurity 
 
double  maxImpurity  
 
 
 
 
 
 
leftAve 
 
double  leftAve  
 
 
 
 
 
 
rightAve 
 
double  rightAve  
 
 
 
 
 
 
splitAttr 
 
int  splitAttr  
 
 
 
 
 
 
splitValue 
 
double  splitValue  
 
 
 
 
 

 
 
  
 Package   weka.clusterers   
 
 

 
   
 
 
  
 Class  weka.clusterers.AbstractClusterer  extends java.lang.Object implements Serializable   
 
 

 
 serialVersionUID:  -6099962589663877632L

 

 
   
 
 
  
 Class  weka.clusterers.AbstractDensityBasedClusterer  extends  AbstractClusterer  implements Serializable   
 
 

 
 serialVersionUID:  -5950728041704213845L

 

 
   
 
 
  
 Class  weka.clusterers.CLOPE  extends  AbstractClusterer  implements Serializable   
 
 

 
 serialVersionUID:  -567567567567588L

 
   
 
 
  
 Serialized Fields   
 
 

 
clusters 
 
java.util.ArrayList<E>  clusters  
 
 Array of clusters
 
 
 
 
 
 
m_RepulsionDefault 
 
double  m_RepulsionDefault  
 
 Specifies the repulsion default
 
 
 
 
 
 
m_Repulsion 
 
double  m_Repulsion  
 
 Specifies the repulsion
 
 
 
 
 
 
m_numberOfClusters 
 
int  m_numberOfClusters  
 
 Number of clusters
 
 
 
 
 
 
m_processed_InstanceID 
 
int  m_processed_InstanceID  
 
 Counter for the processed instances
 
 
 
 
 
 
m_numberOfInstances 
 
int  m_numberOfInstances  
 
 Number of instances
 
 
 
 
 
 
m_clusterAssignments 
 
java.util.ArrayList<E>  m_clusterAssignments  
 
 
 
 
 
 
m_numberOfClustersDetermined 
 
boolean  m_numberOfClustersDetermined  
 
 whether the number of clusters was already determined
 
 
 
 

 
   
 
 
  
 Class  weka.clusterers.ClusterEvaluation  extends java.lang.Object implements Serializable   
 
 

 
 serialVersionUID:  -830188327319128005L

 
   
 
 
  
 Serialized Fields   
 
 

 
m_Clusterer 
 
 Clusterer   m_Clusterer  
 
 the clusterer
 
 
 
 
 
 
m_clusteringResults 
 
java.lang.StringBuffer  m_clusteringResults  
 
 holds a string describing the results of clustering the training data
 
 
 
 
 
 
m_numClusters 
 
int  m_numClusters  
 
 holds the number of clusters found by the clusterer
 
 
 
 
 
 
m_clusterAssignments 
 
double[]  m_clusterAssignments  
 
 holds the assigments of instances to clusters for a particular testing
      dataset
 
 
 
 
 
 
m_logL 
 
double  m_logL  
 
 holds the average log likelihood for a particular testing dataset
     if the clusterer is a DensityBasedClusterer
 
 
 
 
 
 
m_classToCluster 
 
int[]  m_classToCluster  
 
 will hold the mapping of classes to clusters (for class based 
      evaluation)
 
 
 
 

 
   
 
 
  
 Class  weka.clusterers.Cobweb  extends  RandomizableClusterer  implements Serializable   
 
 

 
 serialVersionUID:  928406656495092318L

 
   
 
 
  
 Serialized Fields   
 
 

 
m_acuity 
 
double  m_acuity  
 
 Acuity (minimum standard deviation).
 
 
 
 
 
 
m_cutoff 
 
double  m_cutoff  
 
 Cutoff (minimum category utility).
 
 
 
 
 
 
m_cobwebTree 
 
weka.clusterers.Cobweb.CNode  m_cobwebTree  
 
 Holds the root of the Cobweb tree.
 
 
 
 
 
 
m_numberOfClusters 
 
int  m_numberOfClusters  
 
 Number of clusters (nodes in the tree). Must never be queried directly, 
 only via the method numberOfClusters(). Otherwise it's not guaranteed that 
 it contains the correct value.
 
 
  See Also:    Cobweb.numberOfClusters()  , 
  Cobweb.m_numberOfClustersDetermined   
 
 
 
m_numberOfClustersDetermined 
 
boolean  m_numberOfClustersDetermined  
 
 whether the number of clusters was already determined
 
 
 
 
 
 
m_numberSplits 
 
int  m_numberSplits  
 
 the number of splits that happened
 
 
 
 
 
 
m_numberMerges 
 
int  m_numberMerges  
 
 the number of merges that happened
 
 
 
 
 
 
m_saveInstances 
 
boolean  m_saveInstances  
 
 Output instances in graph representation of Cobweb tree (Allows
 instances at nodes in the tree to be visualized in the Explorer).
 
 
 
 

 
   
 
 
  
 Class  weka.clusterers.DBScan  extends  AbstractClusterer  implements Serializable   
 
 

 
 serialVersionUID:  -1666498248451219728L

 
   
 
 
  
 Serialized Fields   
 
 

 
epsilon 
 
double  epsilon  
 
 Specifies the radius for a range-query
 
 
 
 
 
 
minPoints 
 
int  minPoints  
 
 Specifies the density (the range-query must contain at least minPoints DataObjects)
 
 
 
 
 
 
replaceMissingValues_Filter 
 
 ReplaceMissingValues   replaceMissingValues_Filter  
 
 Replace missing values in training instances
 
 
 
 
 
 
numberOfGeneratedClusters 
 
int  numberOfGeneratedClusters  
 
 Holds the number of clusters generated
 
 
 
 
 
 
database_distanceType 
 
java.lang.String  database_distanceType  
 
 Holds the distance-type that is used
 (default = weka.clusterers.forOPTICSAndDBScan.DataObjects.EuclidianDataObject)
 
 
 
 
 
 
database_Type 
 
java.lang.String  database_Type  
 
 Holds the type of the used database
 (default = weka.clusterers.forOPTICSAndDBScan.Databases.SequentialDatabase)
 
 
 
 
 
 
database 
 
 Database   database  
 
 The database that is used for DBScan
 
 
 
 
 
 
clusterID 
 
int  clusterID  
 
 Holds the current clusterID
 
 
 
 
 
 
processed_InstanceID 
 
int  processed_InstanceID  
 
 Counter for the processed instances
 
 
 
 
 
 
elapsedTime 
 
double  elapsedTime  
 
 Holds the time-value (seconds) for the duration of the clustering-process
 
 
 
 

 
   
 
 
  
 Class  weka.clusterers.EM  extends  RandomizableDensityBasedClusterer  implements Serializable   
 
 

 
 serialVersionUID:  8348181483812829475L

 
   
 
 
  
 Serialized Fields   
 
 

 
m_model 
 
 Estimator [][]  m_model  
 
 hold the discrete estimators for each cluster
 
 
 
 
 
 
m_modelNormal 
 
double[][][]  m_modelNormal  
 
 hold the normal estimators for each cluster
 
 
 
 
 
 
m_minStdDev 
 
double  m_minStdDev  
 
 default minimum standard deviation
 
 
 
 
 
 
m_minStdDevPerAtt 
 
double[]  m_minStdDevPerAtt  
 
 
 
 
 
 
m_weights 
 
double[][]  m_weights  
 
 hold the weights of each instance for each cluster
 
 
 
 
 
 
m_priors 
 
double[]  m_priors  
 
 the prior probabilities for clusters
 
 
 
 
 
 
m_loglikely 
 
double  m_loglikely  
 
 the loglikelihood of the data
 
 
 
 
 
 
m_theInstances 
 
 Instances   m_theInstances  
 
 training instances
 
 
 
 
 
 
m_num_clusters 
 
int  m_num_clusters  
 
 number of clusters selected by the user or cross validation
 
 
 
 
 
 
m_initialNumClusters 
 
int  m_initialNumClusters  
 
 the initial number of clusters requested by the user--- -1 if
      xval is to be used to find the number of clusters
 
 
 
 
 
 
m_num_attribs 
 
int  m_num_attribs  
 
 number of attributes
 
 
 
 
 
 
m_num_instances 
 
int  m_num_instances  
 
 number of training instances
 
 
 
 
 
 
m_max_iterations 
 
int  m_max_iterations  
 
 maximum iterations to perform
 
 
 
 
 
 
m_minValues 
 
double[]  m_minValues  
 
 attribute min values
 
 
 
 
 
 
m_maxValues 
 
double[]  m_maxValues  
 
 attribute max values
 
 
 
 
 
 
m_rr 
 
java.util.Random  m_rr  
 
 random number generator
 
 
 
 
 
 
m_verbose 
 
boolean  m_verbose  
 
 Verbose?
 
 
 
 
 
 
m_replaceMissing 
 
 ReplaceMissingValues   m_replaceMissing  
 
 globally replace missing values
 
 
 
 
 
 
m_displayModelInOldFormat 
 
boolean  m_displayModelInOldFormat  
 
 display model output in old-style format
 
 
 
 

 
   
 
 
  
 Class  weka.clusterers.FarthestFirst  extends  RandomizableClusterer  implements Serializable   
 
 

 
 serialVersionUID:  7499838100631329509L

 
   
 
 
  
 Serialized Fields   
 
 

 
m_instances 
 
 Instances   m_instances  
 
 training instances, not necessary to keep, 
 could be replaced by m_ClusterCentroids where needed for header info
 
 
 
 
 
 
m_ReplaceMissingFilter 
 
 ReplaceMissingValues   m_ReplaceMissingFilter  
 
 replace missing values in training instances
 
 
 
 
 
 
m_NumClusters 
 
int  m_NumClusters  
 
 number of clusters to generate
 
 
 
 
 
 
m_ClusterCentroids 
 
 Instances   m_ClusterCentroids  
 
 holds the cluster centroids
 
 
 
 
 
 
m_Min 
 
double[]  m_Min  
 
 attribute min values
 
 
 
 
 
 
m_Max 
 
double[]  m_Max  
 
 attribute max values
 
 
 
 

 
   
 
 
  
 Class  weka.clusterers.FilteredClusterer  extends  SingleClustererEnhancer  implements Serializable   
 
 

 
 serialVersionUID:  1420005943163412943L

 
   
 
 
  
 Serialized Fields   
 
 

 
m_Filter 
 
 Filter   m_Filter  
 
 The filter.
 
 
 
 
 
 
m_FilteredInstances 
 
 Instances   m_FilteredInstances  
 
 The instance structure of the filtered instances.
 
 
 
 

 
   
 
 
  
 Class  weka.clusterers.HierarchicalClusterer  extends  AbstractClusterer  implements Serializable   
 
 

 
 serialVersionUID:  1L

 
   
 
 
  
 Serialized Fields   
 
 

 
m_bDebug 
 
boolean  m_bDebug  
 
 Whether the classifier is run in debug mode.
 
 
 
 
 
 
m_bDistanceIsBranchLength 
 
boolean  m_bDistanceIsBranchLength  
 
 Whether the distance represent node height (if false) or branch length (if true).
 
 
 
 
 
 
m_instances 
 
 Instances   m_instances  
 
 training data
 
 
 
 
 
 
m_nNumClusters 
 
int  m_nNumClusters  
 
 number of clusters desired in clustering
 
 
 
 
 
 
m_DistanceFunction 
 
 DistanceFunction   m_DistanceFunction  
 
 distance function used for comparing members of a cluster
 
 
 
 
 
 
m_nLinkType 
 
int  m_nLinkType  
 
 Holds the Link type used calculate distance between clusters
 
 
 
 
 
 
m_bPrintNewick 
 
boolean  m_bPrintNewick  
 
 
 
 
 
 
m_clusters 
 
weka.clusterers.HierarchicalClusterer.Node[]  m_clusters  
 
 
 
 
 
 
m_nClusterNr 
 
int[]  m_nClusterNr  
 
 
 
 

 
   
 
 
  
 Class  weka.clusterers.MakeDensityBasedClusterer  extends  AbstractDensityBasedClusterer  implements Serializable   
 
 

 
 serialVersionUID:  -5643302427972186631L

 
   
 
 
  
 Serialized Fields   
 
 

 
m_theInstances 
 
 Instances   m_theInstances  
 
 holds training instances header information
 
 
 
 
 
 
m_priors 
 
double[]  m_priors  
 
 prior probabilities for the fitted clusters
 
 
 
 
 
 
m_modelNormal 
 
double[][][]  m_modelNormal  
 
 normal distributions fitted to each numeric attribute in each cluster
 
 
 
 
 
 
m_model 
 
 DiscreteEstimator [][]  m_model  
 
 discrete distributions fitted to each discrete attribute in each cluster
 
 
 
 
 
 
m_minStdDev 
 
double  m_minStdDev  
 
 default minimum standard deviation
 
 
 
 
 
 
m_wrappedClusterer 
 
 Clusterer   m_wrappedClusterer  
 
 The clusterer being wrapped
 
 
 
 
 
 
m_replaceMissing 
 
 ReplaceMissingValues   m_replaceMissing  
 
 globally replace missing values
 
 
 
 

 
   
 
 
  
 Class  weka.clusterers.OPTICS  extends  AbstractClusterer  implements Serializable   
 
 

 
 serialVersionUID:  274552680222105221L

 
   
 
 
  
 Serialized Fields   
 
 

 
epsilon 
 
double  epsilon  
 
 Specifies the radius for a range-query
 
 
 
 
 
 
minPoints 
 
int  minPoints  
 
 Specifies the density (the range-query must contain at least minPoints DataObjects)
 
 
 
 
 
 
replaceMissingValues_Filter 
 
 ReplaceMissingValues   replaceMissingValues_Filter  
 
 Replace missing values in training instances
 
 
 
 
 
 
numberOfGeneratedClusters 
 
int  numberOfGeneratedClusters  
 
 Holds the number of clusters generated
 
 
 
 
 
 
database_distanceType 
 
java.lang.String  database_distanceType  
 
 Holds the distance-type that is used
 (default = weka.clusterers.forOPTICSAndDBScan.DataObjects.EuclidianDataObject)
 
 
 
 
 
 
database_Type 
 
java.lang.String  database_Type  
 
 Holds the type of the used database
 (default = weka.clusterers.forOPTICSAndDBScan.Databases.SequentialDatabase)
 
 
 
 
 
 
database 
 
 Database   database  
 
 The database that is used for OPTICS
 
 
 
 
 
 
elapsedTime 
 
double  elapsedTime  
 
 Holds the time-value (seconds) for the duration of the clustering-process
 
 
 
 
 
 
writeOPTICSresults 
 
boolean  writeOPTICSresults  
 
 Flag that indicates if the results are written to a file or not
 
 
 
 
 
 
resultVector 
 
 FastVector   resultVector  
 
 Holds the ClusterOrder (dataObjects with their r_dist and c_dist) for the GUI
 
 
 
 
 
 
showGUI 
 
boolean  showGUI  
 
 whether to display the GUI after building the clusterer or not.
 
 
 
 
 
 
databaseOutput 
 
java.io.File  databaseOutput  
 
 the file to save the generated database object to.
 
 
 
 

 
   
 
 
  
 Class  weka.clusterers.RandomizableClusterer  extends  AbstractClusterer  implements Serializable   
 
 

 
 serialVersionUID:  -4819590778152242745L

 
   
 
 
  
 Serialized Fields   
 
 

 
m_SeedDefault 
 
int  m_SeedDefault  
 
 the default seed value
 
 
 
 
 
 
m_Seed 
 
int  m_Seed  
 
 The random number seed.
 
 
 
 

 
   
 
 
  
 Class  weka.clusterers.RandomizableDensityBasedClusterer  extends  AbstractDensityBasedClusterer  implements Serializable   
 
 

 
 serialVersionUID:  -5325270357918932849L

 
   
 
 
  
 Serialized Fields   
 
 

 
m_SeedDefault 
 
int  m_SeedDefault  
 
 the default seed value
 
 
 
 
 
 
m_Seed 
 
int  m_Seed  
 
 The random number seed.
 
 
 
 

 
   
 
 
  
 Class  weka.clusterers.RandomizableSingleClustererEnhancer  extends  AbstractClusterer  implements Serializable   
 
 

 
 serialVersionUID:  -644847037106316249L

 
   
 
 
  
 Serialized Fields   
 
 

 
m_SeedDefault 
 
int  m_SeedDefault  
 
 the default seed value
 
 
 
 
 
 
m_Seed 
 
int  m_Seed  
 
 The random number seed.
 
 
 
 

 
   
 
 
  
 Class  weka.clusterers.sIB  extends  RandomizableClusterer  implements Serializable   
 
 

 
 serialVersionUID:  -8652125897352654213L

 
   
 
 
  
 Serialized Fields   
 
 

 
m_data 
 
 Instances   m_data  
 
 Training data
 
 
 
 
 
 
m_numCluster 
 
int  m_numCluster  
 
 Number of clusters
 
 
 
 
 
 
m_numRestarts 
 
int  m_numRestarts  
 
 Number of restarts
 
 
 
 
 
 
m_verbose 
 
boolean  m_verbose  
 
 Verbose?
 
 
 
 
 
 
m_uniformPrior 
 
boolean  m_uniformPrior  
 
 Uniform prior probability of the documents
 
 
 
 
 
 
m_maxLoop 
 
int  m_maxLoop  
 
 Max number of iterations during each restart
 
 
 
 
 
 
m_minChange 
 
int  m_minChange  
 
 Minimum number of changes
 
 
 
 
 
 
m_replaceMissing 
 
 ReplaceMissingValues   m_replaceMissing  
 
 Globally replace missing values
 
 
 
 
 
 
m_numInstances 
 
int  m_numInstances  
 
 Number of instances
 
 
 
 
 
 
m_numAttributes 
 
int  m_numAttributes  
 
 Number of attributes
 
 
 
 
 
 
random 
 
java.util.Random  random  
 
 Randomly generate initial partition
 
 
 
 
 
 
bestT 
 
weka.clusterers.sIB.Partition  bestT  
 
 Holds the best partition built
 
 
 
 
 
 
input 
 
weka.clusterers.sIB.Input  input  
 
 Holds the statistics about the input dataset
 
 
 
 

 
   
 
 
  
 Class  weka.clusterers.SimpleKMeans  extends  RandomizableClusterer  implements Serializable   
 
 

 
 serialVersionUID:  -3235809600124455376L

 
   
 
 
  
 Serialized Fields   
 
 

 
m_ReplaceMissingFilter 
 
 ReplaceMissingValues   m_ReplaceMissingFilter  
 
 replace missing values in training instances
 
 
 
 
 
 
m_NumClusters 
 
int  m_NumClusters  
 
 number of clusters to generate
 
 
 
 
 
 
m_ClusterCentroids 
 
 Instances   m_ClusterCentroids  
 
 holds the cluster centroids
 
 
 
 
 
 
m_ClusterStdDevs 
 
 Instances   m_ClusterStdDevs  
 
 Holds the standard deviations of the numeric attributes in each cluster
 
 
 
 
 
 
m_ClusterNominalCounts 
 
int[][][]  m_ClusterNominalCounts  
 
 For each cluster, holds the frequency counts for the values of each 
 nominal attribute
 
 
 
 
 
 
m_ClusterMissingCounts 
 
int[][]  m_ClusterMissingCounts  
 
 
 
 
 
 
m_FullMeansOrMediansOrModes 
 
double[]  m_FullMeansOrMediansOrModes  
 
 Stats on the full data set for comparison purposes
 In case the attribute is numeric the value is the mean if is 
 being used the Euclidian distance or the median if Manhattan distance
 and if the attribute is nominal then it's mode is saved
 
 
 
 
 
 
m_FullStdDevs 
 
double[]  m_FullStdDevs  
 
 
 
 
 
 
m_FullNominalCounts 
 
int[][]  m_FullNominalCounts  
 
 
 
 
 
 
m_FullMissingCounts 
 
int[]  m_FullMissingCounts  
 
 
 
 
 
 
m_displayStdDevs 
 
boolean  m_displayStdDevs  
 
 Display standard deviations for numeric atts
 
 
 
 
 
 
m_dontReplaceMissing 
 
boolean  m_dontReplaceMissing  
 
 Replace missing values globally?
 
 
 
 
 
 
m_ClusterSizes 
 
int[]  m_ClusterSizes  
 
 The number of instances in each cluster
 
 
 
 
 
 
m_MaxIterations 
 
int  m_MaxIterations  
 
 Maximum number of iterations to be executed
 
 
 
 
 
 
m_Iterations 
 
int  m_Iterations  
 
 Keep track of the number of iterations completed before convergence
 
 
 
 
 
 
m_squaredErrors 
 
double[]  m_squaredErrors  
 
 Holds the squared errors for all clusters
 
 
 
 
 
 
m_DistanceFunction 
 
 DistanceFunction   m_DistanceFunction  
 
 the distance function used.
 
 
 
 
 
 
m_PreserveOrder 
 
boolean  m_PreserveOrder  
 
 Preserve order of instances
 
 
 
 
 
 
m_Assignments 
 
int[]  m_Assignments  
 
 Assignments obtained
 
 
 
 

 
   
 
 
  
 Class  weka.clusterers.SingleClustererEnhancer  extends  AbstractClusterer  implements Serializable   
 
 

 
 serialVersionUID:  4893928362926428671L

 
   
 
 
  
 Serialized Fields   
 
 

 
m_Clusterer 
 
 Clusterer   m_Clusterer  
 
 the clusterer
 
 
 
 

 
   
 
 
  
 Class  weka.clusterers.XMeans  extends  RandomizableClusterer  implements Serializable   
 
 

 
 serialVersionUID:  -7941793078404132616L

 
   
 
 
  
 Serialized Fields   
 
 

 
m_Instances 
 
 Instances   m_Instances  
 
 training instances.
 
 
 
 
 
 
m_Model 
 
 Instances   m_Model  
 
 model information, should increase readability.
 
 
 
 
 
 
m_ReplaceMissingFilter 
 
 ReplaceMissingValues   m_ReplaceMissingFilter  
 
 replace missing values in training instances.
 
 
 
 
 
 
m_BinValue 
 
double  m_BinValue  
 
 Distance value between true and false of binary attributes and 
 "same" and "different" of nominal attributes (default = 1.0).
 
 
 
 
 
 
m_Bic 
 
double  m_Bic  
 
 BIC-Score of the current model.
 
 
 
 
 
 
m_Mle 
 
double[]  m_Mle  
 
 Distortion.
 
 
 
 
 
 
m_MaxIterations 
 
int  m_MaxIterations  
 
 maximum overall iterations.
 
 
 
 
 
 
m_MaxKMeans 
 
int  m_MaxKMeans  
 
 maximum iterations to perform Kmeans part 
 if negative, iterations are not checked.
 
 
 
 
 
 
m_MaxKMeansForChildren 
 
int  m_MaxKMeansForChildren  
 
 see above, but for kMeans of splitted clusters.
 
 
 
 
 
 
m_NumClusters 
 
int  m_NumClusters  
 
 The actual number of clusters.
 
 
 
 
 
 
m_MinNumClusters 
 
int  m_MinNumClusters  
 
 min number of clusters to generate.
 
 
 
 
 
 
m_MaxNumClusters 
 
int  m_MaxNumClusters  
 
 max number of clusters to generate.
 
 
 
 
 
 
m_DistanceF 
 
 DistanceFunction   m_DistanceF  
 
 the distance function used.
 
 
 
 
 
 
m_ClusterCenters 
 
 Instances   m_ClusterCenters  
 
 cluster centers.
 
 
 
 
 
 
m_InputCenterFile 
 
java.io.File  m_InputCenterFile  
 
 file name of the output file for the cluster centers.
 
 
 
 
 
 
m_DebugVectorsInput 
 
java.io.Reader  m_DebugVectorsInput  
 
 input file for the random vectors --> USED FOR DEBUGGING.
 
 
 
 
 
 
m_DebugVectorsIndex 
 
int  m_DebugVectorsIndex  
 
 the index for the current debug vector.
 
 
 
 
 
 
m_DebugVectors 
 
 Instances   m_DebugVectors  
 
 all the debug vectors.
 
 
 
 
 
 
m_DebugVectorsFile 
 
java.io.File  m_DebugVectorsFile  
 
 file name of the input file for the random vectors.
 
 
 
 
 
 
m_CenterInput 
 
java.io.Reader  m_CenterInput  
 
 input file for the cluster centers.
 
 
 
 
 
 
m_OutputCenterFile 
 
java.io.File  m_OutputCenterFile  
 
 file name of the output file for the cluster centers.
 
 
 
 
 
 
m_CenterOutput 
 
java.io.PrintWriter  m_CenterOutput  
 
 output file for the cluster centers.
 
 
 
 
 
 
m_ClusterAssignments 
 
int[]  m_ClusterAssignments  
 
 temporary variable holding cluster assignments while iterating.
 
 
 
 
 
 
m_CutOffFactor 
 
double  m_CutOffFactor  
 
 cutoff factor - percentage of splits done in Improve-Structure part
     only relevant, if all children lost.
 
 
 
 
 
 
m_KDTree 
 
 KDTree   m_KDTree  
 
 KDTrees class if KDTrees are used.
 
 
 
 
 
 
m_UseKDTree 
 
boolean  m_UseKDTree  
 
 whether to use the KDTree (the KDTree is only initialized to be 
 configurable from the GUI).
 
 
 
 
 
 
m_IterationCount 
 
int  m_IterationCount  
 
 counts iterations done in main loop.
 
 
 
 
 
 
m_KMeansStopped 
 
int  m_KMeansStopped  
 
 counter to say how often kMeans was stopped by loop counter.
 
 
 
 
 
 
m_NumSplits 
 
int  m_NumSplits  
 
 Number of splits prepared.
 
 
 
 
 
 
m_NumSplitsDone 
 
int  m_NumSplitsDone  
 
 Number of splits accepted (including cutoff factor decisions).
 
 
 
 
 
 
m_NumSplitsStillDone 
 
int  m_NumSplitsStillDone  
 
 Number of splits accepted just because of cutoff factor.
 
 
 
 
 
 
m_DebugLevel 
 
int  m_DebugLevel  
 
 level of debug output, 0 is no output.
 
 
 
 
 
 
m_CurrDebugFlag 
 
boolean  m_CurrDebugFlag  
 
 Flag: I'm debugging.
 
 
 
 
 

 
 
  
 Package   weka.clusterers.forOPTICSAndDBScan.Databases   
 
 

 
   
 
 
  
 Class  weka.clusterers.forOPTICSAndDBScan.Databases.SequentialDatabase  extends java.lang.Object implements Serializable   
 
 

 
 serialVersionUID:  787245523118665778L

 
   
 
 
  
 Serialized Fields   
 
 

 
treeMap 
 
java.util.TreeMap<K,V>  treeMap  
 
 Internal, sorted Treemap for storing all the DataObjects
 
 
 
 
 
 
instances 
 
 Instances   instances  
 
 Holds the original instances delivered from WEKA
 
 
 
 
 
 
attributeMinValues 
 
double[]  attributeMinValues  
 
 Holds the minimum value for each attribute
 
 
 
 
 
 
attributeMaxValues 
 
double[]  attributeMaxValues  
 
 Holds the maximum value for each attribute
 
 
 
 
 

 
 
  
 Package   weka.clusterers.forOPTICSAndDBScan.DataObjects   
 
 

 
   
 
 
  
 Class  weka.clusterers.forOPTICSAndDBScan.DataObjects.EuclidianDataObject  extends java.lang.Object implements Serializable   
 
 

 
 serialVersionUID:  -4408119914898291075L

 
   
 
 
  
 Serialized Fields   
 
 

 
instance 
 
 Instance   instance  
 
 Holds the original instance
 
 
 
 
 
 
key 
 
java.lang.String  key  
 
 Holds the (unique) key that is associated with this DataObject
 
 
 
 
 
 
clusterID 
 
int  clusterID  
 
 Holds the ID of the cluster, to which this DataObject is assigned
 
 
 
 
 
 
processed 
 
boolean  processed  
 
 Holds the status for this DataObject (true, if it has been processed, else false)
 
 
 
 
 
 
c_dist 
 
double  c_dist  
 
 Holds the coreDistance for this DataObject
 
 
 
 
 
 
r_dist 
 
double  r_dist  
 
 Holds the reachabilityDistance for this DataObject
 
 
 
 
 
 
database 
 
 Database   database  
 
 Holds the database, that is the keeper of this DataObject
 
 
 
 

 
   
 
 
  
 Class  weka.clusterers.forOPTICSAndDBScan.DataObjects.ManhattanDataObject  extends java.lang.Object implements Serializable   
 
 

 
 serialVersionUID:  -3417720553766544582L

 
   
 
 
  
 Serialized Fields   
 
 

 
instance 
 
 Instance   instance  
 
 Holds the original instance
 
 
 
 
 
 
key 
 
java.lang.String  key  
 
 Holds the (unique) key that is associated with this DataObject
 
 
 
 
 
 
clusterID 
 
int  clusterID  
 
 Holds the ID of the cluster, to which this DataObject is assigned
 
 
 
 
 
 
processed 
 
boolean  processed  
 
 Holds the status for this DataObject (true, if it has been processed, else false)
 
 
 
 
 
 
c_dist 
 
double  c_dist  
 
 Holds the coreDistance for this DataObject
 
 
 
 
 
 
r_dist 
 
double  r_dist  
 
 Holds the reachabilityDistance for this DataObject
 
 
 
 
 
 
database 
 
 Database   database  
 
 Holds the database, that is the keeper of this DataObject
 
 
 
 
 

 
 
  
 Package   weka.clusterers.forOPTICSAndDBScan.OPTICS_GUI   
 
 

 
   
 
 
  
 Class  weka.clusterers.forOPTICSAndDBScan.OPTICS_GUI.GraphPanel  extends javax.swing.JComponent implements Serializable   
 
 

 
 serialVersionUID:  7917937528738361470L

 
   
 
 
  
 Serialized Fields   
 
 

 
resultVector 
 
 FastVector   resultVector  
 
 Holds the clustering results
 
 
 
 
 
 
verticalAdjustment 
 
int  verticalAdjustment  
 
 Holds the value that is multiplied with the original values of core- and reachability
 distances in order to get better graphical views
 
 
 
 
 
 
coreDistanceColor 
 
java.awt.Color  coreDistanceColor  
 
 Specifies the color for displaying core-distances
 
 
 
 
 
 
reachabilityDistanceColor 
 
java.awt.Color  reachabilityDistanceColor  
 
 Specifies the color for displaying reachability-distances
 
 
 
 
 
 
widthSlider 
 
int  widthSlider  
 
 Specifies the width for displaying the distances
 
 
 
 
 
 
showCoreDistances 
 
boolean  showCoreDistances  
 
 Holds the flag for showCoreDistances
 
 
 
 
 
 
showReachabilityDistances 
 
boolean  showReachabilityDistances  
 
 Holds the flag for showrRechabilityDistances
 
 
 
 
 
 
recentIndex 
 
int  recentIndex  
 
 Holds the index of the last toolTip
 
 
 
 

 
   
 
 
  
 Class  weka.clusterers.forOPTICSAndDBScan.OPTICS_GUI.ResultVectorTableModel  extends javax.swing.table.AbstractTableModel implements Serializable   
 
 

 
 serialVersionUID:  -7732711470435549210L

 
   
 
 
  
 Serialized Fields   
 
 

 
resultVector 
 
 FastVector   resultVector  
 
 Holds the ClusterOrder (dataObjects with their r_dist and c_dist) for the GUI
 
 
 
 

 
   
 
 
  
 Class  weka.clusterers.forOPTICSAndDBScan.OPTICS_GUI.SERObject  extends java.lang.Object implements Serializable   
 
 

 
 serialVersionUID:  -6022057864970639151L

 
   
 
 
  
 Serialized Fields   
 
 

 
resultVector 
 
 FastVector   resultVector  
 
 
 
 
 
 
databaseSize 
 
int  databaseSize  
 
 
 
 
 
 
numberOfAttributes 
 
int  numberOfAttributes  
 
 
 
 
 
 
epsilon 
 
double  epsilon  
 
 
 
 
 
 
minPoints 
 
int  minPoints  
 
 
 
 
 
 
opticsOutputs 
 
boolean  opticsOutputs  
 
 
 
 
 
 
database_Type 
 
java.lang.String  database_Type  
 
 
 
 
 
 
database_distanceType 
 
java.lang.String  database_distanceType  
 
 
 
 
 
 
numberOfGeneratedClusters 
 
int  numberOfGeneratedClusters  
 
 
 
 
 
 
elapsedTime 
 
java.lang.String  elapsedTime  
 
 
 
 
 

 
 
  
 Package   weka.core   
 
 

 
   
 
 
  
 Class  weka.core.AbstractStringDistanceFunction  extends  NormalizableDistance  implements Serializable   
 
 

 

 
   
 
 
  
 Class  weka.core.AlgVector  extends java.lang.Object implements Serializable   
 
 

 
 serialVersionUID:  -4023736016850256591L

 
   
 
 
  
 Serialized Fields   
 
 

 
m_Elements 
 
double[]  m_Elements  
 
 The values of the matrix
 
 
 
 

 
   
 
 
  
 Class  weka.core.Attribute  extends java.lang.Object implements Serializable   
 
 

 
 serialVersionUID:  -742180568732916383L

 
   
 
 
  
 Serialized Fields   
 
 

 
m_Name 
 
java.lang.String  m_Name  
 
 The attribute's name.
 
 
 
 
 
 
m_Type 
 
int  m_Type  
 
 The attribute's type.
 
 
 
 
 
 
m_Values 
 
 FastVector   m_Values  
 
 The attribute's values (if nominal or string).
 
 
 
 
 
 
m_Hashtable 
 
java.util.Hashtable<K,V>  m_Hashtable  
 
 Mapping of values to indices (if nominal or string).
 
 
 
 
 
 
m_Header 
 
 Instances   m_Header  
 
 The header information for a relation-valued attribute.
 
 
 
 
 
 
m_DateFormat 
 
java.text.SimpleDateFormat  m_DateFormat  
 
 Date format specification for date attributes
 
 
 
 
 
 
m_Index 
 
int  m_Index  
 
 The attribute's index.
 
 
 
 
 
 
m_Metadata 
 
 ProtectedProperties   m_Metadata  
 
 The attribute's metadata.
 
 
 
 
 
 
m_Ordering 
 
int  m_Ordering  
 
 The attribute's ordering.
 
 
 
 
 
 
m_IsRegular 
 
boolean  m_IsRegular  
 
 Whether the attribute is regular.
 
 
 
 
 
 
m_IsAveragable 
 
boolean  m_IsAveragable  
 
 Whether the attribute is averagable.
 
 
 
 
 
 
m_HasZeropoint 
 
boolean  m_HasZeropoint  
 
 Whether the attribute has a zeropoint.
 
 
 
 
 
 
m_Weight 
 
double  m_Weight  
 
 The attribute's weight.
 
 
 
 
 
 
m_LowerBound 
 
double  m_LowerBound  
 
 The attribute's lower numeric bound.
 
 
 
 
 
 
m_LowerBoundIsOpen 
 
boolean  m_LowerBoundIsOpen  
 
 Whether the lower bound is open.
 
 
 
 
 
 
m_UpperBound 
 
double  m_UpperBound  
 
 The attribute's upper numeric bound.
 
 
 
 
 
 
m_UpperBoundIsOpen 
 
boolean  m_UpperBoundIsOpen  
 
 Whether the upper bound is open
 
 
 
 

 
   
 
 
  
 Class  weka.core.AttributeExpression  extends java.lang.Object implements Serializable   
 
 

 
 serialVersionUID:  402130123261736245L

 
   
 
 
  
 Serialized Fields   
 
 

 
m_operatorStack 
 
java.util.Stack<E>  m_operatorStack  
 
 Operator stack
 
 
 
 
 
 
m_originalInfix 
 
java.lang.String  m_originalInfix  
 
 Holds the original infix expression
 
 
 
 
 
 
m_postFixExpVector 
 
java.util.Vector<E>  m_postFixExpVector  
 
 Holds the expression in postfix form
 
 
 
 
 
 
m_signMod 
 
boolean  m_signMod  
 
 True if the next numeric constant or attribute index is negative
 
 
 
 
 
 
m_previousTok 
 
java.lang.String  m_previousTok  
 
 Holds the previous token
 
 
 
 

 
   
 
 
  
 Class  weka.core.AttributeLocator  extends java.lang.Object implements Serializable   
 
 

 
 serialVersionUID:  -2932848827681070345L

 
   
 
 
  
 Serialized Fields   
 
 

 
m_AllowedIndices 
 
int[]  m_AllowedIndices  
 
 the attribute indices that may be inspected
 
 
 
 
 
 
m_Attributes 
 
java.util.Vector<E>  m_Attributes  
 
 contains the attribute locations, either true or false Boolean objects
 
 
 
 
 
 
m_Locators 
 
java.util.Vector<E>  m_Locators  
 
 contains the locator locations, either null or a AttributeLocator reference
 
 
 
 
 
 
m_Type 
 
int  m_Type  
 
 the type of the attribute
 
 
 
 
 
 
m_Data 
 
 Instances   m_Data  
 
 the referenced data
 
 
 
 
 
 
m_Indices 
 
int[]  m_Indices  
 
 the indices
 
 
 
 
 
 
m_LocatorIndices 
 
int[]  m_LocatorIndices  
 
 the indices of locator objects
 
 
 
 

 
   
 
 
  
 Class  weka.core.AttributeStats  extends java.lang.Object implements Serializable   
 
 

 
 serialVersionUID:  4434688832743939380L

 
   
 
 
  
 Serialized Fields   
 
 

 
intCount 
 
int  intCount  
 
 The number of int-like values
 
 
 
 
 
 
realCount 
 
int  realCount  
 
 The number of real-like values (i.e. have a fractional part)
 
 
 
 
 
 
missingCount 
 
int  missingCount  
 
 The number of missing values
 
 
 
 
 
 
distinctCount 
 
int  distinctCount  
 
 The number of distinct values
 
 
 
 
 
 
uniqueCount 
 
int  uniqueCount  
 
 The number of values that only appear once
 
 
 
 
 
 
totalCount 
 
int  totalCount  
 
 The total number of values (i.e. number of instances)
 
 
 
 
 
 
numericStats 
 
 Stats   numericStats  
 
 Stats on numeric value distributions
 
 
 
 
 
 
nominalCounts 
 
int[]  nominalCounts  
 
 Counts of each nominal value
 
 
 
 

 
   
 
 
  
 Class  weka.core.BinarySparseInstance  extends  SparseInstance  implements Serializable   
 
 

 
 serialVersionUID:  -5297388762342528737L

 

 
   
 
 
  
 Class  weka.core.Capabilities  extends java.lang.Object implements Serializable   
 
 

 
 serialVersionUID:  -5478590032325567849L

 
   
 
 
  
 Serialized Fields   
 
 

 
m_Owner 
 
 CapabilitiesHandler   m_Owner  
 
 the object that owns this capabilities instance
 
 
 
 
 
 
m_Capabilities 
 
java.util.HashSet<E>  m_Capabilities  
 
 the hashset for storing the active capabilities
 
 
 
 
 
 
m_Dependencies 
 
java.util.HashSet<E>  m_Dependencies  
 
 the hashset for storing dependent capabilities, eg for meta-classifiers
 
 
 
 
 
 
m_FailReason 
 
java.lang.Exception  m_FailReason  
 
 the reason why the test failed, used to throw an exception
 
 
 
 
 
 
m_MinimumNumberInstances 
 
int  m_MinimumNumberInstances  
 
 the minimum number of instances in a dataset
 
 
 
 
 
 
m_Test 
 
boolean  m_Test  
 
 whether to perform any tests at all
 
 
 
 
 
 
m_InstancesTest 
 
boolean  m_InstancesTest  
 
 whether to perform data based tests
 
 
 
 
 
 
m_AttributeTest 
 
boolean  m_AttributeTest  
 
 whether to perform attribute based tests
 
 
 
 
 
 
m_MissingValuesTest 
 
boolean  m_MissingValuesTest  
 
 whether to test for missing values
 
 
 
 
 
 
m_MissingClassValuesTest 
 
boolean  m_MissingClassValuesTest  
 
 whether to test for missing class values
 
 
 
 
 
 
m_MinimumNumberInstancesTest 
 
boolean  m_MinimumNumberInstancesTest  
 
 whether to test for minimum number of instances
 
 
 
 

 
   
 
 
  
 Class  weka.core.ChebyshevDistance  extends  NormalizableDistance  implements Serializable   
 
 

 
 serialVersionUID:  -7739904999895461429L

 

 
   
 
 
  
 Class  weka.core.Debug  extends java.lang.Object implements Serializable   
 
 

 
 serialVersionUID:  66171861743328020L

 
   
 
 
  
 Serialized Fields   
 
 

 
m_Enabled 
 
boolean  m_Enabled  
 
 whether logging is enabled
 
 
 
 
 
 
m_Log 
 
 Debug.Log   m_Log  
 
 for logging
 
 
 
 
 
 
m_Clock 
 
 Debug.Clock   m_Clock  
 
 for clocking
 
 
 
 

 
   
 
 
  
 Class  weka.core.Debug.Clock  extends java.lang.Object implements Serializable   
 
 

 
 serialVersionUID:  4622161807307942201L

 
   
 
 
  
 Serialized Fields   
 
 

 
m_OutputFormat 
 
int  m_OutputFormat  
 
 the format of the output
 
 
 
 
 
 
m_Start 
 
long  m_Start  
 
 the start time
 
 
 
 
 
 
m_Stop 
 
long  m_Stop  
 
 the end time
 
 
 
 
 
 
m_Running 
 
boolean  m_Running  
 
 whether the time is still clocked
 
 
 
 
 
 
m_ThreadID 
 
long  m_ThreadID  
 
 the thread ID
 
 
 
 
 
 
m_CanMeasureCpuTime 
 
boolean  m_CanMeasureCpuTime  
 
 whether the system can measure the CPU time
 
 
 
 
 
 
m_UseCpuTime 
 
boolean  m_UseCpuTime  
 
 whether to use the CPU time (by default TRUE)
 
 
 
 

 
   
 
 
  
 Class  weka.core.Debug.DBO  extends java.lang.Object implements Serializable   
 
 

 
 serialVersionUID:  -5245628124742606784L

 
   
 
 
  
 Serialized Fields   
 
 

 
m_verboseOn 
 
boolean  m_verboseOn  
 
 enables/disables output of debug information
 
 
 
 
 
 
m_outputTypes 
 
 Range   m_outputTypes  
 
 range of outputtyp
 
 
 
 

 
   
 
 
  
 Class  weka.core.Debug.Log  extends java.lang.Object implements Serializable   
 
 

 
 serialVersionUID:  1458435732111675823L

 
   
 
 
  
 Serialized Fields   
 
 

 
m_Filename 
 
java.lang.String  m_Filename  
 
 the filename, if any
 
 
 
 
 
 
m_Size 
 
int  m_Size  
 
 the size of the file (in bytes)
 
 
 
 
 
 
m_NumFiles 
 
int  m_NumFiles  
 
 the number of files for rotating the logs
 
 
 
 
 
 
m_LoggerInitFailed 
 
boolean  m_LoggerInitFailed  
 
 whether the initialization of the logger failed
 
 
 
 

 
   
 
 
  
 Class  weka.core.Debug.Random  extends java.util.Random implements Serializable   
 
 

 
 serialVersionUID:  1256846887618333956L

 
   
 
 
  
 Serialized Fields   
 
 

 
m_Debug 
 
boolean  m_Debug  
 
 whether to output debug information
 
 
 
 
 
 
m_ID 
 
long  m_ID  
 
 the unique ID for this number generator
 
 
 
 
 
 
m_Log 
 
 Debug.Log   m_Log  
 
 the log to use for outputting the data, otherwise just stdout
 
 
 
 

 
   
 
 
  
 Class  weka.core.Debug.SimpleLog  extends java.lang.Object implements Serializable   
 
 

 
 serialVersionUID:  -2671928223819510830L

 
   
 
 
  
 Serialized Fields   
 
 

 
m_Filename 
 
java.lang.String  m_Filename  
 
 the file to write to (if null then only stdout is used)
 
 
 
 

 
   
 
 
  
 Class  weka.core.Debug.Timestamp  extends java.lang.Object implements Serializable   
 
 

 
 serialVersionUID:  -6099868388466922753L

 
   
 
 
  
 Serialized Fields   
 
 

 
m_Stamp 
 
java.util.Date  m_Stamp  
 
 the actual date
 
 
 
 
 
 
m_Format 
 
java.lang.String  m_Format  
 
 the format of the timestamp
 
 
 
 
 
 
m_Formatter 
 
java.text.SimpleDateFormat  m_Formatter  
 
 handles the format of the output
 
 
 
 

 
   
 
 
  
 Class  weka.core.EditDistance  extends  AbstractStringDistanceFunction  implements Serializable   
 
 

 

 
   
 
 
  
 Class  weka.core.EuclideanDistance  extends  NormalizableDistance  implements Serializable   
 
 

 
 serialVersionUID:  1068606253458807903L

 

 
   
 
 
  
 Class  weka.core.FastVector  extends java.lang.Object implements Serializable   
 
 

 
 serialVersionUID:  -2173635135622930169L

 
   
 
 
  
 Serialized Fields   
 
 

 
m_Objects 
 
java.lang.Object[]  m_Objects  
 
 The array of objects.
 
 
 
 
 
 
m_Size 
 
int  m_Size  
 
 The current size;
 
 
 
 
 
 
m_CapacityIncrement 
 
int  m_CapacityIncrement  
 
 The capacity increment
 
 
 
 
 
 
m_CapacityMultiplier 
 
int  m_CapacityMultiplier  
 
 The capacity multiplier.
 
 
 
 

 
   
 
 
  
 Class  weka.core.Instance  extends java.lang.Object implements Serializable   
 
 

 
 serialVersionUID:  1482635194499365122L

 
   
 
 
  
 Serialized Fields   
 
 

 
m_Dataset 
 
 Instances   m_Dataset  
 
 The dataset the instance has access to.  Null if the instance
 doesn't have access to any dataset.  Only if an instance has
 access to a dataset, it knows about the actual attribute types.
 
 
 
 
 
 
m_AttValues 
 
double[]  m_AttValues  
 
 The instance's attribute values.
 
 
 
 
 
 
m_Weight 
 
double  m_Weight  
 
 The instance's weight.
 
 
 
 

 
   
 
 
  
 Class  weka.core.InstanceComparator  extends java.lang.Object implements Serializable   
 
 

 
 serialVersionUID:  -6589278678230949683L

 
   
 
 
  
 Serialized Fields   
 
 

 
m_IncludeClass 
 
boolean  m_IncludeClass  
 
 whether to include the class in the comparison
 
 
 
 

 
   
 
 
  
 Class  weka.core.Instances  extends java.lang.Object implements Serializable   
 
 

 
 serialVersionUID:  -19412345060742748L

 
   
 
 
  
 Serialized Fields   
 
 

 
m_RelationName 
 
java.lang.String  m_RelationName  
 
 The dataset's name.
 
 
 
 
 
 
m_Attributes 
 
 FastVector   m_Attributes  
 
 The attribute information.
 
 
 
 
 
 
m_Instances 
 
 FastVector   m_Instances  
 
 The instances.
 
 
 
 
 
 
m_ClassIndex 
 
int  m_ClassIndex  
 
 The class attribute's index
 
 
 
 
 
 
m_Lines 
 
int  m_Lines  
 
 The lines read so far in case of incremental loading. Since the
 StreamTokenizer will be re-initialized with every instance that is read,
 we have to keep track of the number of lines read so far.
 
 
  See Also:    Instances.readInstance(Reader)   
 
 
 
attIdx4Randomization 
 
int  attIdx4Randomization  
 
 used in randomizeAttribute and undoRandomizeAttribute to store/restore
 the index of attribute that was last shuffled, and it's original values
 
 
 
 
 
 
attIdxOrigValues 
 
double[]  attIdxOrigValues  
 
 
 
 

 
   
 
 
  
 Class  weka.core.Jython  extends java.lang.Object implements Serializable   
 
 

 
 serialVersionUID:  -6972298704460209252L

 
   
 
 
  
 Serialized Fields   
 
 

 
m_Interpreter 
 
java.lang.Object  m_Interpreter  
 
 the interpreter
 
 
 
 

 
   
 
 
  
 Class  weka.core.ManhattanDistance  extends  NormalizableDistance  implements Serializable   
 
 

 
 serialVersionUID:  6783782554224000243L

 

 
   
 
 
  
 Class  weka.core.Matrix  extends java.lang.Object implements Serializable   
 
 

 
 serialVersionUID:  -3604757095849145838L

 
   
 
 
  
 Serialized Fields   
 
 

 
m_Matrix 
 
 Matrix   m_Matrix  
 
  Deprecated.   The actual matrix
 
 
 
 

 
   
 
 
  
 Class  weka.core.NormalizableDistance  extends java.lang.Object implements Serializable   
 
 

 
   
 
 
  
 Serialized Fields   
 
 

 
m_Data 
 
 Instances   m_Data  
 
 the instances used internally.
 
 
 
 
 
 
m_DontNormalize 
 
boolean  m_DontNormalize  
 
 True if normalization is turned off (default false).
 
 
 
 
 
 
m_Ranges 
 
double[][]  m_Ranges  
 
 The range of the attributes.
 
 
 
 
 
 
m_AttributeIndices 
 
 Range   m_AttributeIndices  
 
 The range of attributes to use for calculating the distance.
 
 
 
 
 
 
m_ActiveIndices 
 
boolean[]  m_ActiveIndices  
 
 The boolean flags, whether an attribute will be used or not.
 
 
 
 
 
 
m_Validated 
 
boolean  m_Validated  
 
 Whether all the necessary preparations have been done.
 
 
 
 

 
   
 
 
  
 Class  weka.core.NoSupportForMissingValuesException  extends  WekaException  implements Serializable   
 
 

 
 serialVersionUID:  5161175307725893973L

 

 
   
 
 
  
 Class  weka.core.ProtectedProperties  extends java.util.Properties implements Serializable   
 
 

 
 serialVersionUID:  3876658672657323985L

 
   
 
 
  
 Serialized Fields   
 
 

 
closed 
 
boolean  closed  
 
 the properties need to be open during construction of the object
 
 
 
 

 
   
 
 
  
 Class  weka.core.Queue  extends java.lang.Object implements Serializable   
 
 

 
 serialVersionUID:  -1141282001146389780L

 
   
 
 
  
 Serialized Fields   
 
 

 
m_Head 
 
weka.core.Queue.QueueNode  m_Head  
 
 Store a reference to the head of the queue
 
 
 
 
 
 
m_Tail 
 
weka.core.Queue.QueueNode  m_Tail  
 
 Store a reference to the tail of the queue
 
 
 
 
 
 
m_Size 
 
int  m_Size  
 
 Store the c m_Tail.m_Nexturrent number of elements in the queue
 
 
 
 

 
   
 
 
  
 Class weka.core.Queue.QueueNode extends java.lang.Object implements Serializable   
 
 

 
 serialVersionUID:  -5119358279412097455L

 
   
 
 
  
 Serialized Fields   
 
 

 
m_Next 
 
weka.core.Queue.QueueNode  m_Next  
 
 The next node in the queue
 
 
 
 
 
 
m_Contents 
 
java.lang.Object  m_Contents  
 
 The nodes contents
 
 
 
 

 
   
 
 
  
 Class  weka.core.RandomVariates  extends java.util.Random implements Serializable   
 
 

 
 serialVersionUID:  -4763742718209460354L

 

 
   
 
 
  
 Class  weka.core.Range  extends java.lang.Object implements Serializable   
 
 

 
 serialVersionUID:  3667337062176835900L

 
   
 
 
  
 Serialized Fields   
 
 

 
m_RangeStrings 
 
java.util.Vector<E>  m_RangeStrings  
 
 Record the string representations of the columns to delete
 
 
 
 
 
 
m_Invert 
 
boolean  m_Invert  
 
 Whether matching should be inverted
 
 
 
 
 
 
m_SelectFlags 
 
boolean[]  m_SelectFlags  
 
 The array of flags for whether an column is selected
 
 
 
 
 
 
m_Upper 
 
int  m_Upper  
 
 Store the maximum value permitted in the range. -1 indicates that
      no upper value has been set
 
 
 
 

 
   
 
 
  
 Class  weka.core.RelationalLocator  extends  AttributeLocator  implements Serializable   
 
 

 
 serialVersionUID:  4646872277151854732L

 

 
   
 
 
  
 Class  weka.core.SerializedObject  extends java.lang.Object implements Serializable   
 
 

 
 serialVersionUID:  6635502953928860434L

 
   
 
 
  
 Serialized Fields   
 
 

 
m_storedObjectArray 
 
byte[]  m_storedObjectArray  
 
 The array storing the object.
 
 
 
 
 
 
m_isCompressed 
 
boolean  m_isCompressed  
 
 Whether or not the object is compressed.
 
 
 
 
 
 
m_isJython 
 
boolean  m_isJython  
 
 Whether it is a Jython object or not
 
 
 
 

 
   
 
 
  
 Class  weka.core.SingleIndex  extends java.lang.Object implements Serializable   
 
 

 
 serialVersionUID:  5285169134430839303L

 
   
 
 
  
 Serialized Fields   
 
 

 
m_IndexString 
 
java.lang.String  m_IndexString  
 
 Record the string representation of the number
 
 
 
 
 
 
m_SelectedIndex 
 
int  m_SelectedIndex  
 
 The selected index
 
 
 
 
 
 
m_Upper 
 
int  m_Upper  
 
 Store the maximum value permitted. -1 indicates that no upper
      value has been set
 
 
 
 

 
   
 
 
  
 Class  weka.core.SparseInstance  extends  Instance  implements Serializable   
 
 

 
 serialVersionUID:  -3579051291332630149L

 
   
 
 
  
 Serialized Fields   
 
 

 
m_Indices 
 
int[]  m_Indices  
 
 The index of the attribute associated with each stored value.
 
 
 
 
 
 
m_NumAttributes 
 
int  m_NumAttributes  
 
 The maximum number of values that can be stored.
 
 
 
 

 
   
 
 
  
 Class  weka.core.StringLocator  extends  AttributeLocator  implements Serializable   
 
 

 
 serialVersionUID:  7805522230268783972L

 

 
   
 
 
  
 Class  weka.core.Tag  extends java.lang.Object implements Serializable   
 
 

 
 serialVersionUID:  3326379903447135320L

 
   
 
 
  
 Serialized Fields   
 
 

 
m_ID 
 
int  m_ID  
 
 The ID
 
 
 
 
 
 
m_IDStr 
 
java.lang.String  m_IDStr  
 
 The unique string for this tag, doesn't have to be numeric
 
 
 
 
 
 
m_Readable 
 
java.lang.String  m_Readable  
 
 The descriptive text
 
 
 
 

 
   
 
 
  
 Class  weka.core.TestInstances  extends java.lang.Object implements Serializable   
 
 

 
 serialVersionUID:  -6263968936330390469L

 
   
 
 
  
 Serialized Fields   
 
 

 
m_Words 
 
java.lang.String[]  m_Words  
 
 for generating String attributes/classes
 
 
 
 
 
 
m_WordSeparators 
 
java.lang.String  m_WordSeparators  
 
 for generating String attributes/classes
 
 
 
 
 
 
m_Relation 
 
java.lang.String  m_Relation  
 
 the name of the relation
 
 
 
 
 
 
m_Seed 
 
int  m_Seed  
 
 the seed value
 
 
 
 
 
 
m_Random 
 
java.util.Random  m_Random  
 
 the random number generator
 
 
 
 
 
 
m_NumInstances 
 
int  m_NumInstances  
 
 the number of instances
 
 
 
 
 
 
m_ClassType 
 
int  m_ClassType  
 
 the class type
 
 
 
 
 
 
m_NumClasses 
 
int  m_NumClasses  
 
 the number of classes (in case of NOMINAL class)
 
 
 
 
 
 
m_ClassIndex 
 
int  m_ClassIndex  
 
 the class index (-1 is LAST, -2 means no class)
 
 
  See Also:    TestInstances.CLASS_IS_LAST  , 
  TestInstances.NO_CLASS   
 
 
 
m_NumNominal 
 
int  m_NumNominal  
 
 the number of nominal attributes
 
 
 
 
 
 
m_NumNominalValues 
 
int  m_NumNominalValues  
 
 the number of values for nominal attributes
 
 
 
 
 
 
m_NumNumeric 
 
int  m_NumNumeric  
 
 the number of numeric attributes
 
 
 
 
 
 
m_NumString 
 
int  m_NumString  
 
 the number of string attributes
 
 
 
 
 
 
m_NumDate 
 
int  m_NumDate  
 
 the number of date attributes
 
 
 
 
 
 
m_NumRelational 
 
int  m_NumRelational  
 
 the number of relational attributes
 
 
 
 
 
 
m_NumRelationalNominal 
 
int  m_NumRelationalNominal  
 
 the number of nominal attributes in a relational attribute
 
 
 
 
 
 
m_NumRelationalNominalValues 
 
int  m_NumRelationalNominalValues  
 
 the number of values for nominal attributes in relational attributes
 
 
 
 
 
 
m_NumRelationalNumeric 
 
int  m_NumRelationalNumeric  
 
 the number of numeric attributes in a relational attribute
 
 
 
 
 
 
m_NumRelationalString 
 
int  m_NumRelationalString  
 
 the number of string attributes in a relational attribute
 
 
 
 
 
 
m_NumRelationalDate 
 
int  m_NumRelationalDate  
 
 the number of date attributes in a relational attribute
 
 
 
 
 
 
m_MultiInstance 
 
boolean  m_MultiInstance  
 
 whether to generate Multi-Instance data or not
 
 
 
 
 
 
m_NumInstancesRelational 
 
int  m_NumInstancesRelational  
 
 the number of instances in relational attributes (applies also for bags
 in multi-instance)
 
 
 
 
 
 
m_RelationalFormat 
 
 Instances []  m_RelationalFormat  
 
 the format of the multi-instance data
 
 
 
 
 
 
m_RelationalClassFormat 
 
 Instances   m_RelationalClassFormat  
 
 the format of the multi-instance data of the class
 
 
 
 
 
 
m_Data 
 
 Instances   m_Data  
 
 the generated data
 
 
 
 
 
 
m_Handler 
 
 CapabilitiesHandler   m_Handler  
 
 the CapabilitiesHandler to get the Capabilities from
 
 
 
 

 
   
 
 
  
 Class  weka.core.Trie  extends java.lang.Object implements Serializable   
 
 

 
 serialVersionUID:  -5897980928817779048L

 
   
 
 
  
 Serialized Fields   
 
 

 
m_Root 
 
 Trie.TrieNode   m_Root  
 
 the root node
 
 
 
 
 
 
m_HashCode 
 
int  m_HashCode  
 
 the hash code
 
 
 
 
 
 
m_RecalcHashCode 
 
boolean  m_RecalcHashCode  
 
 whether the structure got modified and the hash code needs to be 
 re-calculated
 
 
 
 

 
   
 
 
  
 Class  weka.core.Trie.TrieNode  extends javax.swing.tree.DefaultMutableTreeNode implements Serializable   
 
 

 
 serialVersionUID:  -2252907099391881148L

 
   
 
 
  
 Serialized Fields   
 
 

 
m_Children 
 
java.util.Hashtable<K,V>  m_Children  
 
 for fast access to the children
 
 
 
 

 
   
 
 
  
 Class  weka.core.UnassignedClassException  extends java.lang.RuntimeException implements Serializable   
 
 

 
 serialVersionUID:  6268278694768818235L

 

 
   
 
 
  
 Class  weka.core.UnassignedDatasetException  extends java.lang.RuntimeException implements Serializable   
 
 

 
 serialVersionUID:  -9000116786626328854L

 

 
   
 
 
  
 Class  weka.core.UnsupportedAttributeTypeException  extends  WekaException  implements Serializable   
 
 

 
 serialVersionUID:  2658987325328414838L

 

 
   
 
 
  
 Class  weka.core.UnsupportedClassTypeException  extends  WekaException  implements Serializable   
 
 

 
 serialVersionUID:  5175741076972192151L

 

 
   
 
 
  
 Class  weka.core.WekaException  extends java.lang.Exception implements Serializable   
 
 

 
 serialVersionUID:  6428269989006208585L

 
 

 
 
  
 Package   weka.core.converters   
 
 

 
   
 
 
  
 Class  weka.core.converters.AbstractFileLoader  extends  AbstractLoader  implements Serializable   
 
 

 
   
 
 
  
 Serialized Fields   
 
 

 
m_File 
 
java.lang.String  m_File  
 
 the file
 
 
 
 
 
 
m_sourceFile 
 
java.io.File  m_sourceFile  
 
 Holds the source of the data set.
 
 
 
 
 
 
m_useRelativePath 
 
boolean  m_useRelativePath  
 
 use relative file paths
 
 
 
 

 
   
 
 
  
 Class  weka.core.converters.AbstractFileSaver  extends  AbstractSaver  implements Serializable   
 
 

 
   
 
 
  
 Serialized Fields   
 
 

 
m_outputFile 
 
java.io.File  m_outputFile  
 
 The destination file.
 
 
 
 
 
 
FILE_EXTENSION 
 
java.lang.String  FILE_EXTENSION  
 
 The file extension of the destination file.
 
 
 
 
 
 
FILE_EXTENSION_COMPRESSED 
 
java.lang.String  FILE_EXTENSION_COMPRESSED  
 
 the extension for compressed files
 
 
 
 
 
 
m_prefix 
 
java.lang.String  m_prefix  
 
 The prefix for the filename (chosen in the GUI).
 
 
 
 
 
 
m_dir 
 
java.lang.String  m_dir  
 
 The directory of the file (chosen in the GUI).
 
 
 
 
 
 
m_incrementalCounter 
 
int  m_incrementalCounter  
 
 Counter. In incremental mode after reading 100 instances they will be written to a file.
 
 
 
 
 
 
m_useRelativePath 
 
boolean  m_useRelativePath  
 
 use relative file paths
 
 
 
 

 
   
 
 
  
 Class  weka.core.converters.AbstractLoader  extends java.lang.Object implements Serializable   
 
 

 
   
 
 
  
 Serialized Fields   
 
 

 
m_retrieval 
 
int  m_retrieval  
 
 The current retrieval mode
 
 
 
 

 
   
 
 
  
 Class  weka.core.converters.AbstractSaver  extends java.lang.Object implements Serializable   
 
 

 
   
 
 
  
 Serialized Fields   
 
 

 
m_instances 
 
 Instances   m_instances  
 
 The instances that should be stored
 
 
 
 
 
 
m_retrieval 
 
int  m_retrieval  
 
 The current retrieval mode
 
 
 
 
 
 
m_writeMode 
 
int  m_writeMode  
 
 The current write mode
 
 
 
 

 
   
 
 
  
 Class  weka.core.converters.ArffLoader  extends  AbstractFileLoader  implements Serializable   
 
 

 
 serialVersionUID:  2726929550544048587L

 
   
 
 
  
 Serialized Fields   
 
 

 
m_URL 
 
java.lang.String  m_URL  
 
 the url
 
 
 
 

 
   
 
 
  
 Class  weka.core.converters.ArffSaver  extends  AbstractFileSaver  implements Serializable   
 
 

 
 serialVersionUID:  2223634248900042228L

 
   
 
 
  
 Serialized Fields   
 
 

 
m_CompressOutput 
 
boolean  m_CompressOutput  
 
 whether to compress the output
 
 
 
 

 
   
 
 
  
 Class  weka.core.converters.C45Loader  extends  AbstractFileLoader  implements Serializable   
 
 

 
 serialVersionUID:  5454329403218219L

 
   
 
 
  
 Serialized Fields   
 
 

 
m_sourceFileData 
 
java.io.File  m_sourceFileData  
 
 Describe variable  m_sourceFileData  here.
 
 
 
 
 
 
m_fileStem 
 
java.lang.String  m_fileStem  
 
 Holds the filestem.
 
 
 
 
 
 
m_numAttribs 
 
int  m_numAttribs  
 
 Number of attributes in the data (including ignore and label attributes).
 
 
 
 
 
 
m_ignore 
 
boolean[]  m_ignore  
 
 Which attributes are ignore or label. These are *not* included in the
 arff representation.
 
 
 
 

 
   
 
 
  
 Class  weka.core.converters.C45Saver  extends  AbstractFileSaver  implements Serializable   
 
 

 
 serialVersionUID:  -821428878384253377L

 

 
   
 
 
  
 Class  weka.core.converters.ConverterUtils  extends java.lang.Object implements Serializable   
 
 

 
 serialVersionUID:  -2460855349276148760L

 

 
   
 
 
  
 Class  weka.core.converters.ConverterUtils.DataSink  extends java.lang.Object implements Serializable   
 
 

 
 serialVersionUID:  -1504966891136411204L

 
   
 
 
  
 Serialized Fields   
 
 

 
m_Saver 
 
 Saver   m_Saver  
 
 the saver to use for storing the data.
 
 
 
 
 
 
m_Stream 
 
java.io.OutputStream  m_Stream  
 
 the stream to store the data in (always in ARFF format).
 
 
 
 

 
   
 
 
  
 Class  weka.core.converters.ConverterUtils.DataSource  extends java.lang.Object implements Serializable   
 
 

 
 serialVersionUID:  -613122395928757332L

 
   
 
 
  
 Serialized Fields   
 
 

 
m_File 
 
java.io.File  m_File  
 
 the file to load.
 
 
 
 
 
 
m_URL 
 
java.net.URL  m_URL  
 
 the URL to load.
 
 
 
 
 
 
m_Loader 
 
 Loader   m_Loader  
 
 the loader.
 
 
 
 
 
 
m_Incremental 
 
boolean  m_Incremental  
 
 whether the loader is incremental.
 
 
 
 
 
 
m_BatchCounter 
 
int  m_BatchCounter  
 
 the instance counter for the batch case.
 
 
 
 
 
 
m_IncrementalBuffer 
 
 Instance   m_IncrementalBuffer  
 
 the last internally read instance.
 
 
 
 
 
 
m_BatchBuffer 
 
 Instances   m_BatchBuffer  
 
 the batch buffer.
 
 
 
 

 
   
 
 
  
 Class  weka.core.converters.CSVLoader  extends  AbstractFileLoader  implements Serializable   
 
 

 
 serialVersionUID:  5607529739745491340L

 
   
 
 
  
 Serialized Fields   
 
 

 
m_cumulativeStructure 
 
 FastVector   m_cumulativeStructure  
 
 A list of hash tables for accumulating nominal values during parsing.
 
 
 
 
 
 
m_cumulativeInstances 
 
 FastVector   m_cumulativeInstances  
 
 Holds instances accumulated so far.
 
 
 
 
 
 
m_NominalAttributes 
 
 Range   m_NominalAttributes  
 
 The range of attributes to force to type nominal.
 
 
 
 
 
 
m_StringAttributes 
 
 Range   m_StringAttributes  
 
 The range of attributes to force to type string.
 
 
 
 
 
 
m_dateAttributes 
 
 Range   m_dateAttributes  
 
 The range of attributes to force to type date
 
 
 
 
 
 
m_dateFormat 
 
java.lang.String  m_dateFormat  
 
 The formatting string to use to parse dates
 
 
 
 
 
 
m_formatter 
 
java.text.SimpleDateFormat  m_formatter  
 
 The formatter to use on dates
 
 
 
 
 
 
m_MissingValue 
 
java.lang.String  m_MissingValue  
 
 The placeholder for missing values.
 
 
 
 
 
 
m_FirstCheck 
 
boolean  m_FirstCheck  
 
 whether the first row has been read.
 
 
 
 

 
   
 
 
  
 Class  weka.core.converters.CSVSaver  extends  AbstractFileSaver  implements Serializable   
 
 

 
 serialVersionUID:  476636654410701807L

 

 
   
 
 
  
 Class  weka.core.converters.DatabaseConnection  extends  DatabaseUtils  implements Serializable   
 
 

 
 serialVersionUID:  1673169848863178695L

 

 
   
 
 
  
 Class  weka.core.converters.DatabaseLoader  extends  AbstractLoader  implements Serializable   
 
 

 
 serialVersionUID:  -7936159015338318659L

 
   
 
 
  
 Serialized Fields   
 
 

 
m_structure 
 
 Instances   m_structure  
 
 The header information that is retrieved in the beginning of incremental loading
 
 
 
 
 
 
m_datasetPseudoInc 
 
 Instances   m_datasetPseudoInc  
 
 Used in pseudoincremental mode. The whole dataset from which instances will be read incrementally.
 
 
 
 
 
 
m_oldStructure 
 
 Instances   m_oldStructure  
 
 Set of instances that equals m_structure except that the auto_generated_id column is not included as an attribute
 
 
 
 
 
 
m_DataBaseConnection 
 
 DatabaseConnection   m_DataBaseConnection  
 
 The database connection
 
 
 
 
 
 
m_query 
 
java.lang.String  m_query  
 
 The user defined query to load instances. (form: SELECT *|&ltcolumn-list> FROM &lttable> [WHERE <condition>])
 
 
 
 
 
 
m_pseudoIncremental 
 
boolean  m_pseudoIncremental  
 
 Flag indicating that pseudo incremental mode is used (all instances load at once into main memeory and then incrementally from main memory instead of the database)
 
 
 
 
 
 
m_checkForTable 
 
boolean  m_checkForTable  
 
 If true it checks whether or not the table exists in the database before loading depending on jdbc metadata information.
  Set flag to false if no check is required or if jdbc metadata is not complete.
 
 
 
 
 
 
m_nominalToStringLimit 
 
int  m_nominalToStringLimit  
 
 Limit when an attribute is treated as string attribute and not as a nominal one because it has to many values.
 
 
 
 
 
 
m_rowCount 
 
int  m_rowCount  
 
 The number of rows obtained by m_query, eg the size of the ResultSet to load
 
 
 
 
 
 
m_counter 
 
int  m_counter  
 
 Indicates how many rows has already been loaded incrementally
 
 
 
 
 
 
m_choice 
 
int  m_choice  
 
 Decides which SQL statement to limit the number of rows should be used. DBMS dependent. Algorithm just tries several possibilities.
 
 
 
 
 
 
m_firstTime 
 
boolean  m_firstTime  
 
 Flag indicating that incremental process wants to read first instance
 
 
 
 
 
 
m_inc 
 
boolean  m_inc  
 
 Flag indicating that incremental mode is chosen (for command line use only)
 
 
 
 
 
 
m_orderBy 
 
 FastVector   m_orderBy  
 
 Contains the name of the columns that uniquely define a row in the ResultSet. Ensures a unique ordering of instances for indremental loading.
 
 
 
 
 
 
m_nominalIndexes 
 
java.util.Hashtable<K,V>[]  m_nominalIndexes  
 
 Stores the index of a nominal value
 
 
 
 
 
 
m_nominalStrings 
 
 FastVector []  m_nominalStrings  
 
 Stores the nominal value
 
 
 
 
 
 
m_idColumn 
 
java.lang.String  m_idColumn  
 
 Name of the primary key column that will allow unique ordering necessary for incremental loading. The name is specified in the DatabaseUtils file.
 
 
 
 
 
 
m_URL 
 
java.lang.String  m_URL  
 
 the JDBC URL to use
 
 
 
 
 
 
m_User 
 
java.lang.String  m_User  
 
 the database user to use
 
 
 
 
 
 
m_Password 
 
java.lang.String  m_Password  
 
 the database password to use
 
 
 
 
 
 
m_Keys 
 
java.lang.String  m_Keys  
 
 the keys for unique ordering
 
 
 
 

 
   
 
 
  
 Class  weka.core.converters.DatabaseSaver  extends  AbstractSaver  implements Serializable   
 
 

 
 serialVersionUID:  863971733782624956L

 
   
 
 
  
 Serialized Fields   
 
 

 
m_DataBaseConnection 
 
 DatabaseConnection   m_DataBaseConnection  
 
 The database connection.
 
 
 
 
 
 
m_tableName 
 
java.lang.String  m_tableName  
 
 The name of the table in which the instances should be stored.
 
 
 
 
 
 
m_inputFile 
 
java.lang.String  m_inputFile  
 
 An input arff file (for command line use).
 
 
 
 
 
 
m_createText 
 
java.lang.String  m_createText  
 
 The database specific type for a string (read in from the properties file).
 
 
 
 
 
 
m_createDouble 
 
java.lang.String  m_createDouble  
 
 The database specific type for a double (read in from the properties file).
 
 
 
 
 
 
m_createInt 
 
java.lang.String  m_createInt  
 
 The database specific type for an int (read in from the properties file).
 
 
 
 
 
 
m_createDate 
 
java.lang.String  m_createDate  
 
 The database specific type for a date (read in from the properties file).
 
 
 
 
 
 
m_DateFormat 
 
java.text.SimpleDateFormat  m_DateFormat  
 
 For converting the date value into a database string.
 
 
 
 
 
 
m_idColumn 
 
java.lang.String  m_idColumn  
 
 The name of the primary key column that will be automatically generated (if enabled). The name is read from DatabaseUtils.
 
 
 
 
 
 
m_count 
 
int  m_count  
 
 counts the rows and used as a primary key value.
 
 
 
 
 
 
m_id 
 
boolean  m_id  
 
 Flag indicating if a primary key column should be added.
 
 
 
 
 
 
m_tabName 
 
boolean  m_tabName  
 
 Flag indicating whether the default name of the table is the relaion name or not.
 
 
 
 
 
 
m_Username 
 
java.lang.String  m_Username  
 
 the user name for the database.
 
 
 
 
 
 
m_Password 
 
java.lang.String  m_Password  
 
 the password for the database.
 
 
 
 

 
   
 
 
  
 Class  weka.core.converters.LibSVMLoader  extends  AbstractFileLoader  implements Serializable   
 
 

 
 serialVersionUID:  4988360125354664417L

 
   
 
 
  
 Serialized Fields   
 
 

 
m_URL 
 
java.lang.String  m_URL  
 
 the url.
 
 
 
 
 
 
m_Buffer 
 
java.util.Vector<E>  m_Buffer  
 
 the buffer of the rows read so far.
 
 
 
 

 
   
 
 
  
 Class  weka.core.converters.LibSVMSaver  extends  AbstractFileSaver  implements Serializable   
 
 

 
 serialVersionUID:  2792295817125694786L

 
   
 
 
  
 Serialized Fields   
 
 

 
m_ClassIndex 
 
 SingleIndex   m_ClassIndex  
 
 the class index
 
 
 
 

 
   
 
 
  
 Class  weka.core.converters.SerializedInstancesLoader  extends  AbstractFileLoader  implements Serializable   
 
 

 
 serialVersionUID:  2391085836269030715L

 
   
 
 
  
 Serialized Fields   
 
 

 
m_Dataset 
 
 Instances   m_Dataset  
 
 Holds the structure (header) of the data set.
 
 
 
 
 
 
m_IncrementalIndex 
 
int  m_IncrementalIndex  
 
 The current index position for incremental reading
 
 
 
 

 
   
 
 
  
 Class  weka.core.converters.SerializedInstancesSaver  extends  AbstractFileSaver  implements Serializable   
 
 

 
 serialVersionUID:  -7717010648500658872L

 
   
 
 
  
 Serialized Fields   
 
 

 
m_objectstream 
 
java.io.ObjectOutputStream  m_objectstream  
 
 the output stream.
 
 
 
 

 
   
 
 
  
 Class  weka.core.converters.SVMLightLoader  extends  AbstractFileLoader  implements Serializable   
 
 

 
 serialVersionUID:  4988360125354664417L

 
   
 
 
  
 Serialized Fields   
 
 

 
m_URL 
 
java.lang.String  m_URL  
 
 the url.
 
 
 
 
 
 
m_Buffer 
 
java.util.Vector<E>  m_Buffer  
 
 the buffer of the rows read so far.
 
 
 
 

 
   
 
 
  
 Class  weka.core.converters.SVMLightSaver  extends  AbstractFileSaver  implements Serializable   
 
 

 
 serialVersionUID:  2605714599263995835L

 
   
 
 
  
 Serialized Fields   
 
 

 
m_ClassIndex 
 
 SingleIndex   m_ClassIndex  
 
 the class index.
 
 
 
 

 
   
 
 
  
 Class  weka.core.converters.TextDirectoryLoader  extends  AbstractLoader  implements Serializable   
 
 

 
 serialVersionUID:  2592118773712247647L

 
   
 
 
  
 Serialized Fields   
 
 

 
m_structure 
 
 Instances   m_structure  
 
 Holds the determined structure (header) of the data set.
 
 
 
 
 
 
m_sourceFile 
 
java.io.File  m_sourceFile  
 
 Holds the source of the data set.
 
 
 
 
 
 
m_Debug 
 
boolean  m_Debug  
 
 whether to print some debug information
 
 
 
 
 
 
m_OutputFilename 
 
boolean  m_OutputFilename  
 
 whether to include the filename as an extra attribute
 
 
 
 
 
 
m_charSet 
 
java.lang.String  m_charSet  
 
 The charset to use when loading text files (default is to just use the 
 default charset).
 
 
 
 

 
   
 
 
  
 Class  weka.core.converters.XRFFLoader  extends  AbstractFileLoader  implements Serializable   
 
 

 
 serialVersionUID:  3764533621135196582L

 
   
 
 
  
 Serialized Fields   
 
 

 
m_URL 
 
java.lang.String  m_URL  
 
 the url
 
 
 
 
 
 
m_XMLInstances 
 
 XMLInstances   m_XMLInstances  
 
 the loaded XML document
 
 
 
 

 
   
 
 
  
 Class  weka.core.converters.XRFFSaver  extends  AbstractFileSaver  implements Serializable   
 
 

 
 serialVersionUID:  -7226404765213522043L

 
   
 
 
  
 Serialized Fields   
 
 

 
m_ClassIndex 
 
 SingleIndex   m_ClassIndex  
 
 the class index
 
 
 
 
 
 
m_XMLInstances 
 
 XMLInstances   m_XMLInstances  
 
 the generated XML document
 
 
 
 
 
 
m_CompressOutput 
 
boolean  m_CompressOutput  
 
 whether to compress the output
 
 
 
 
 

 
 
  
 Package   weka.core.matrix   
 
 

 
   
 
 
  
 Class  weka.core.matrix.CholeskyDecomposition  extends java.lang.Object implements Serializable   
 
 

 
 serialVersionUID:  -8739775942782694701L

 
   
 
 
  
 Serialized Fields   
 
 

 
L 
 
double[][]  L  
 
 Array for internal storage of decomposition.
 
  internal array storage. 
 
 
 
 
n 
 
int  n  
 
 Row and column dimension (square matrix).
 
  matrix dimension. 
 
 
 
 
isspd 
 
boolean  isspd  
 
 Symmetric and positive definite flag.
 
  is symmetric and positive definite flag. 
 
 

 
   
 
 
  
 Class  weka.core.matrix.EigenvalueDecomposition  extends java.lang.Object implements Serializable   
 
 

 
 serialVersionUID:  4011654467211422319L

 
   
 
 
  
 Serialized Fields   
 
 

 
n 
 
int  n  
 
 Row and column dimension (square matrix).
 
  matrix dimension. 
 
 
 
 
issymmetric 
 
boolean  issymmetric  
 
 Symmetry flag.
 
  internal symmetry flag. 
 
 
 
 
d 
 
double[]  d  
 
 Arrays for internal storage of eigenvalues.
 
  internal storage of eigenvalues. 
 
 
 
 
e 
 
double[]  e  
 
 Arrays for internal storage of eigenvalues.
 
  internal storage of eigenvalues. 
 
 
 
 
V 
 
double[][]  V  
 
 Array for internal storage of eigenvectors.
 
  internal storage of eigenvectors. 
 
 
 
 
H 
 
double[][]  H  
 
 Array for internal storage of nonsymmetric Hessenberg form.
 
  internal storage of nonsymmetric Hessenberg form. 
 
 
 
 
ort 
 
double[]  ort  
 
 Working storage for nonsymmetric algorithm.
 
  working storage for nonsymmetric algorithm. 
 
 

 
   
 
 
  
 Class  weka.core.matrix.ExponentialFormat  extends java.text.DecimalFormat implements Serializable   
 
 

 
 serialVersionUID:  -5298981701073897741L

 
   
 
 
  
 Serialized Fields   
 
 

 
nf 
 
java.text.DecimalFormat  nf  
 
 
 
 
 
 
sign 
 
boolean  sign  
 
 
 
 
 
 
digits 
 
int  digits  
 
 
 
 
 
 
exp 
 
int  exp  
 
 
 
 
 
 
trailing 
 
boolean  trailing  
 
 
 
 

 
   
 
 
  
 Class  weka.core.matrix.FlexibleDecimalFormat  extends java.text.DecimalFormat implements Serializable   
 
 

 
 serialVersionUID:  110912192794064140L

 
   
 
 
  
 Serialized Fields   
 
 

 
nf 
 
java.text.DecimalFormat  nf  
 
 
 
 
 
 
digits 
 
int  digits  
 
 
 
 
 
 
exp 
 
boolean  exp  
 
 
 
 
 
 
intDigits 
 
int  intDigits  
 
 
 
 
 
 
decimalDigits 
 
int  decimalDigits  
 
 
 
 
 
 
expIntDigits 
 
int  expIntDigits  
 
 
 
 
 
 
expDecimalDigits 
 
int  expDecimalDigits  
 
 
 
 
 
 
power 
 
int  power  
 
 
 
 
 
 
trailing 
 
boolean  trailing  
 
 
 
 
 
 
grouping 
 
boolean  grouping  
 
 
 
 
 
 
sign 
 
boolean  sign  
 
 
 
 

 
   
 
 
  
 Class  weka.core.matrix.FloatingPointFormat  extends java.text.DecimalFormat implements Serializable   
 
 

 
 serialVersionUID:  4500373755333429499L

 
   
 
 
  
 Serialized Fields   
 
 

 
nf 
 
java.text.DecimalFormat  nf  
 
 
 
 
 
 
width 
 
int  width  
 
 
 
 
 
 
decimal 
 
int  decimal  
 
 
 
 
 
 
trailing 
 
boolean  trailing  
 
 
 
 

 
   
 
 
  
 Class  weka.core.matrix.LUDecomposition  extends java.lang.Object implements Serializable   
 
 

 
 serialVersionUID:  -2731022568037808629L

 
   
 
 
  
 Serialized Fields   
 
 

 
LU 
 
double[][]  LU  
 
 Array for internal storage of decomposition.
 
  internal array storage. 
 
 
 
 
m 
 
int  m  
 
 Row and column dimensions, and pivot sign.
 
  column dimension. 
 
 
 
 
n 
 
int  n  
 
 Row and column dimensions, and pivot sign.
 
  column dimension. 
 
 
 
 
pivsign 
 
int  pivsign  
 
 Row and column dimensions, and pivot sign.
 
  column dimension. 
 
 
 
 
piv 
 
int[]  piv  
 
 Internal storage of pivot vector.
 
  pivot vector. 
 
 

 
   
 
 
  
 Class  weka.core.matrix.Matrix  extends java.lang.Object implements Serializable   
 
 

 
 serialVersionUID:  7856794138418366180L

 
   
 
 
  
 Serialized Fields   
 
 

 
A 
 
double[][]  A  
 
 Array for internal storage of elements.
 
  internal array storage. 
 
 
 
 
m 
 
int  m  
 
 Row and column dimensions.
 
  row dimension. 
 
 
 
 
n 
 
int  n  
 
 Row and column dimensions.
 
  row dimension. 
 
 

 
   
 
 
  
 Class  weka.core.matrix.QRDecomposition  extends java.lang.Object implements Serializable   
 
 

 
 serialVersionUID:  -5013090736132211418L

 
   
 
 
  
 Serialized Fields   
 
 

 
QR 
 
double[][]  QR  
 
 Array for internal storage of decomposition.
 
  internal array storage. 
 
 
 
 
m 
 
int  m  
 
 Row and column dimensions.
 
  column dimension. 
 
 
 
 
n 
 
int  n  
 
 Row and column dimensions.
 
  column dimension. 
 
 
 
 
Rdiag 
 
double[]  Rdiag  
 
 Array for internal storage of diagonal of R.
 
  diagonal of R. 
 
 

 
   
 
 
  
 Class  weka.core.matrix.SingularValueDecomposition  extends java.lang.Object implements Serializable   
 
 

 
 serialVersionUID:  -8738089610999867951L

 
   
 
 
  
 Serialized Fields   
 
 

 
U 
 
double[][]  U  
 
 Arrays for internal storage of U and V.
 
  internal storage of U. 
 
 
 
 
V 
 
double[][]  V  
 
 Arrays for internal storage of U and V.
 
  internal storage of U. 
 
 
 
 
s 
 
double[]  s  
 
 Array for internal storage of singular values.
 
  internal storage of singular values. 
 
 
 
 
m 
 
int  m  
 
 Row and column dimensions.
 
  row dimension. 
 
 
 
 
n 
 
int  n  
 
 Row and column dimensions.
 
  row dimension. 
 
 
 

 
 
  
 Package   weka.core.neighboursearch   
 
 

 
   
 
 
  
 Class  weka.core.neighboursearch.BallTree  extends  NearestNeighbourSearch  implements Serializable   
 
 

 
 serialVersionUID:  728763855952698328L

 
   
 
 
  
 Serialized Fields   
 
 

 
m_InstList 
 
int[]  m_InstList  
 
 The instances indices sorted inorder of appearence in the tree from left
 most leaf node to the right most leaf node.
 
 
 
 
 
 
m_MaxInstancesInLeaf 
 
int  m_MaxInstancesInLeaf  
 
 The maximum number of instances in a leaf. A node is made into a leaf if 
 the number of instances in it become less than or equal to this value.
 
 
 
 
 
 
m_TreeStats 
 
 TreePerformanceStats   m_TreeStats  
 
 Tree Stats variables.
 
 
 
 
 
 
m_Root 
 
 BallNode   m_Root  
 
 The root node of the BallTree.
 
 
 
 
 
 
m_TreeConstructor 
 
 BallTreeConstructor   m_TreeConstructor  
 
 The constructor method to use to build the tree.
 
 
 
 
 
 
m_Distances 
 
double[]  m_Distances  
 
 Array holding the distances of the nearest neighbours. It is filled up
  both by nearestNeighbour() and kNearestNeighbours().
 
 
 
 

 
   
 
 
  
 Class  weka.core.neighboursearch.CoverTree  extends  NearestNeighbourSearch  implements Serializable   
 
 

 
 serialVersionUID:  7617412821497807586L

 
   
 
 
  
 Serialized Fields   
 
 

 
m_EuclideanDistance 
 
 EuclideanDistance   m_EuclideanDistance  
 
 The euclidean distance function to use.
 
 
 
 
 
 
m_Root 
 
 CoverTree.CoverTreeNode   m_Root  
 
 The root node.
 
 
 
 
 
 
m_DistanceList 
 
double[]  m_DistanceList  
 
 Array holding the distances of the nearest neighbours. It is filled up
  both by nearestNeighbour() and kNearestNeighbours().
 
 
 
 
 
 
m_NumNodes 
 
int  m_NumNodes  
 
 Number of nodes in the tree.
 
 
 
 
 
 
m_NumLeaves 
 
int  m_NumLeaves  
 
 Number of nodes in the tree.
 
 
 
 
 
 
m_MaxDepth 
 
int  m_MaxDepth  
 
 Number of nodes in the tree.
 
 
 
 
 
 
m_TreeStats 
 
 TreePerformanceStats   m_TreeStats  
 
 Tree Stats variables.
 
 
 
 
 
 
m_Base 
 
double  m_Base  
 
 The base of our expansion constant. In other words the 2 in 2^i used
 in covering tree and separation invariants of a cover tree. P.S.: In
 paper it's suggested the separation invariant is relaxed in batch
 construction.
 
 
 
 
 
 
il2 
 
double  il2  
 
 if we have base 2 then this can be viewed as 1/ln(2), which can be
 used later on to do il2*ln(d) instead of ln(d)/ln(2), to get log2(d),
 in get_scale method.
 
 
 
 

 
   
 
 
  
 Class  weka.core.neighboursearch.CoverTree.CoverTreeNode  extends java.lang.Object implements Serializable   
 
 

 
 serialVersionUID:  1808760031169036512L

 
   
 
 
  
 Serialized Fields   
 
 

 
nodeid 
 
int  nodeid  
 
 ID for the node.
 
 
 
 
 
 
idx 
 
java.lang.Integer  idx  
 
 Index of the instance represented by this node in the index array.
 
 
 
 
 
 
max_dist 
 
double  max_dist  
 
 The distance of the furthest descendant of the node.
 
 
 
 
 
 
parent_dist 
 
double  parent_dist  
 
 The distance to the nodes parent.
 
 
 
 
 
 
children 
 
 Stack < T >  children  
 
 The children of the node.
 
 
 
 
 
 
num_children 
 
int  num_children  
 
 The number of children node has.
 
 
 
 
 
 
scale 
 
int  scale  
 
 The min i that makes base^i <= max_dist.
 
 
 
 

 
   
 
 
  
 Class  weka.core.neighboursearch.KDTree  extends  NearestNeighbourSearch  implements Serializable   
 
 

 
 serialVersionUID:  1505717283763272533L

 
   
 
 
  
 Serialized Fields   
 
 

 
m_DistanceList 
 
double[]  m_DistanceList  
 
 Array holding the distances of the nearest neighbours. It is filled up both
 by nearestNeighbour() and kNearestNeighbours().
 
 
 
 
 
 
m_InstList 
 
int[]  m_InstList  
 
 Indexlist of the instances of this kdtree. Instances get sorted according
 to the splits. the nodes of the KDTree just hold their start and end
 indices
 
 
 
 
 
 
m_Root 
 
 KDTreeNode   m_Root  
 
 The root node of the tree.
 
 
 
 
 
 
m_Splitter 
 
 KDTreeNodeSplitter   m_Splitter  
 
 The node splitter.
 
 
 
 
 
 
m_NumNodes 
 
int  m_NumNodes  
 
 Tree stats.
 
 
 
 
 
 
m_NumLeaves 
 
int  m_NumLeaves  
 
 Tree stats.
 
 
 
 
 
 
m_MaxDepth 
 
int  m_MaxDepth  
 
 Tree stats.
 
 
 
 
 
 
m_TreeStats 
 
 TreePerformanceStats   m_TreeStats  
 
 Tree Stats variables.
 
 
 
 
 
 
m_NormalizeNodeWidth 
 
boolean  m_NormalizeNodeWidth  
 
 flag for normalizing.
 
 
 
 
 
 
m_EuclideanDistance 
 
 EuclideanDistance   m_EuclideanDistance  
 
 The euclidean distance function to use.
 
 
 
 
 
 
m_MinBoxRelWidth 
 
double  m_MinBoxRelWidth  
 
 minimal relative width of a KDTree rectangle.
 
 
 
 
 
 
m_MaxInstInLeaf 
 
int  m_MaxInstInLeaf  
 
 maximal number of instances in a leaf.
 
 
 
 

 
   
 
 
  
 Class  weka.core.neighboursearch.LinearNNSearch  extends  NearestNeighbourSearch  implements Serializable   
 
 

 
 serialVersionUID:  1915484723703917241L

 
   
 
 
  
 Serialized Fields   
 
 

 
m_Distances 
 
double[]  m_Distances  
 
 Array holding the distances of the nearest neighbours. It is filled up
  both by nearestNeighbour() and kNearestNeighbours().
 
 
 
 
 
 
m_SkipIdentical 
 
boolean  m_SkipIdentical  
 
 Whether to skip instances from the neighbours that are identical to the query instance.
 
 
 
 

 
   
 
 
  
 Class  weka.core.neighboursearch.NearestNeighbourSearch  extends java.lang.Object implements Serializable   
 
 

 
   
 
 
  
 Serialized Fields   
 
 

 
m_Instances 
 
 Instances   m_Instances  
 
 The neighbourhood of instances to find neighbours in.
 
 
 
 
 
 
m_kNN 
 
int  m_kNN  
 
 The number of neighbours to find.
 
 
 
 
 
 
m_DistanceFunction 
 
 DistanceFunction   m_DistanceFunction  
 
 the distance function used.
 
 
 
 
 
 
m_Stats 
 
 PerformanceStats   m_Stats  
 
 Performance statistics.
 
 
 
 
 
 
m_MeasurePerformance 
 
boolean  m_MeasurePerformance  
 
 Should we measure Performance.
 
 
 
 

 
   
 
 
  
 Class  weka.core.neighboursearch.PerformanceStats  extends java.lang.Object implements Serializable   
 
 

 
 serialVersionUID:  -7215110351388368092L

 
   
 
 
  
 Serialized Fields   
 
 

 
m_NumQueries 
 
int  m_NumQueries  
 
 The total number of queries looked at.
 
 
 
 
 
 
m_MinP 
 
double  m_MinP  
 
 The min and max data points looked for a query by 
 the NNS algorithm.
 
 
 
 
 
 
m_MaxP 
 
double  m_MaxP  
 
 The min and max data points looked for a query by 
 the NNS algorithm.
 
 
 
 
 
 
m_SumP 
 
double  m_SumP  
 
 The sum of data points looked 
 at for all the queries.
 
 
 
 
 
 
m_SumSqP 
 
double  m_SumSqP  
 
 The squared sum of data points looked 
 at for all the queries.
 
 
 
 
 
 
m_PointCount 
 
double  m_PointCount  
 
 The number of data points looked at
 for the current/last query.
 
 
 
 
 
 
m_MinC 
 
double  m_MinC  
 
 The min and max coordinates(attributes) looked
 at per query.
 
 
 
 
 
 
m_MaxC 
 
double  m_MaxC  
 
 The min and max coordinates(attributes) looked
 at per query.
 
 
 
 
 
 
m_SumC 
 
double  m_SumC  
 
 The sum of coordinates/attributes looked at
 for all the queries.
 
 
 
 
 
 
m_SumSqC 
 
double  m_SumSqC  
 
 The squared sum of coordinates/attributes looked at
 for all the queries.
 
 
 
 
 
 
m_CoordCount 
 
double  m_CoordCount  
 
 The number of coordinates looked at for
 the current/last query.
 
 
 
 

 
   
 
 
  
 Class  weka.core.neighboursearch.TreePerformanceStats  extends  PerformanceStats  implements Serializable   
 
 

 
 serialVersionUID:  -6637636693340810373L

 
   
 
 
  
 Serialized Fields   
 
 

 
m_MinLeaves 
 
int  m_MinLeaves  
 
 The min and max number leaf nodes looked 
 for a query by the tree based NNS algorithm.
 
 
 
 
 
 
m_MaxLeaves 
 
int  m_MaxLeaves  
 
 The min and max number leaf nodes looked 
 for a query by the tree based NNS algorithm.
 
 
 
 
 
 
m_SumLeaves 
 
int  m_SumLeaves  
 
 The sum of leaf nodes looked 
 at for all the queries.
 
 
 
 
 
 
m_SumSqLeaves 
 
int  m_SumSqLeaves  
 
 The squared sum of leaf nodes looked 
 at for all the queries.
 
 
 
 
 
 
m_LeafCount 
 
int  m_LeafCount  
 
 The number of leaf nodes looked at
 for the current/last query.
 
 
 
 
 
 
m_MinIntNodes 
 
int  m_MinIntNodes  
 
 The min and max number internal nodes looked 
 for a query by the tree based NNS algorithm.
 
 
 
 
 
 
m_MaxIntNodes 
 
int  m_MaxIntNodes  
 
 The min and max number internal nodes looked 
 for a query by the tree based NNS algorithm.
 
 
 
 
 
 
m_SumIntNodes 
 
int  m_SumIntNodes  
 
 The sum of internal nodes looked 
 at for all the queries.
 
 
 
 
 
 
m_SumSqIntNodes 
 
int  m_SumSqIntNodes  
 
 The squared sum of internal nodes looked 
 at for all the queries.
 
 
 
 
 
 
m_IntNodeCount 
 
int  m_IntNodeCount  
 
 The number of internal nodes looked at
 for the current/last query.
 
 
 
 
 

 
 
  
 Package   weka.core.neighboursearch.balltrees   
 
 

 
   
 
 
  
 Class  weka.core.neighboursearch.balltrees.BallNode  extends java.lang.Object implements Serializable   
 
 

 
 serialVersionUID:  -8289151861759883510L

 
   
 
 
  
 Serialized Fields   
 
 

 
m_Start 
 
int  m_Start  
 
 The start index of the portion of the master index array, 
 which stores the indices of the instances/points the node 
 contains.
 
 
 
 
 
 
m_End 
 
int  m_End  
 
 The end index of the portion of the master index array, 
 which stores indices of the instances/points the node 
 contains.
 
 
 
 
 
 
m_NumInstances 
 
int  m_NumInstances  
 
 The number of instances/points in the node.
 
 
 
 
 
 
m_NodeNumber 
 
int  m_NodeNumber  
 
 The node number/id.
 
 
 
 
 
 
m_SplitAttrib 
 
int  m_SplitAttrib  
 
 The attribute that splits this node (not 
 always used).
 
 
 
 
 
 
m_SplitVal 
 
double  m_SplitVal  
 
 The value of m_SpiltAttrib that splits this
 node (not always used).
 
 
 
 
 
 
m_Left 
 
 BallNode   m_Left  
 
 The left child of the node.
 
 
 
 
 
 
m_Right 
 
 BallNode   m_Right  
 
 The right child of the node.
 
 
 
 
 
 
m_Pivot 
 
 Instance   m_Pivot  
 
 The pivot/centre of the ball.
 
 
 
 
 
 
m_Radius 
 
double  m_Radius  
 
 The radius of this ball (hyper sphere).
 
 
 
 

 
   
 
 
  
 Class  weka.core.neighboursearch.balltrees.BallSplitter  extends java.lang.Object implements Serializable   
 
 

 
   
 
 
  
 Serialized Fields   
 
 

 
m_Instances 
 
 Instances   m_Instances  
 
 The instance on which the tree is built.
 
 
 
 
 
 
m_DistanceFunction 
 
 EuclideanDistance   m_DistanceFunction  
 
 The distance function (metric) from which
 the tree is (OR is to be) built.
 
 
 
 
 
 
m_Instlist 
 
int[]  m_Instlist  
 
 The master index array that'll be reshuffled as nodes
 are split (and the tree is constructed).
 
 
 
 

 
   
 
 
  
 Class  weka.core.neighboursearch.balltrees.BallTreeConstructor  extends java.lang.Object implements Serializable   
 
 

 
   
 
 
  
 Serialized Fields   
 
 

 
m_MaxInstancesInLeaf 
 
int  m_MaxInstancesInLeaf  
 
 The maximum number of instances allowed in a leaf.
 
 
 
 
 
 
m_MaxRelLeafRadius 
 
double  m_MaxRelLeafRadius  
 
 The maximum relative radius of a leaf node 
 (relative to the smallest ball enclosing all the 
 data (training) points).
 
 
 
 
 
 
m_FullyContainChildBalls 
 
boolean  m_FullyContainChildBalls  
 
 Should a parent ball completely enclose the balls
 of its two children, or only the points inside
 its children.
 
 
 
 
 
 
m_Instances 
 
 Instances   m_Instances  
 
 The instances on which to build the tree.
 
 
 
 
 
 
m_DistanceFunction 
 
 DistanceFunction   m_DistanceFunction  
 
 The distance function to use to build the tree.
 
 
 
 
 
 
m_NumNodes 
 
int  m_NumNodes  
 
 The number of internal and leaf nodes in the 
 built tree.
 
 
 
 
 
 
m_NumLeaves 
 
int  m_NumLeaves  
 
 The number of leaf nodes in the built tree.
 
 
 
 
 
 
m_MaxDepth 
 
int  m_MaxDepth  
 
 The depth of the built tree.
 
 
 
 
 
 
m_InstList 
 
int[]  m_InstList  
 
 The master index array.
 
 
 
 

 
   
 
 
  
 Class  weka.core.neighboursearch.balltrees.BottomUpConstructor  extends  BallTreeConstructor  implements Serializable   
 
 

 
 serialVersionUID:  5864250777657707687L

 

 
   
 
 
  
 Class  weka.core.neighboursearch.balltrees.MedianDistanceFromArbitraryPoint  extends  BallSplitter  implements Serializable   
 
 

 
 serialVersionUID:  5617378551363700558L

 
   
 
 
  
 Serialized Fields   
 
 

 
m_RandSeed 
 
int  m_RandSeed  
 
 Seed for random number generator.
 
 
 
 
 
 
m_Rand 
 
java.util.Random  m_Rand  
 
 Random number generator for selecting
 an abitrary (random) point.
 
 
 
 

 
   
 
 
  
 Class  weka.core.neighboursearch.balltrees.MedianOfWidestDimension  extends  BallSplitter  implements Serializable   
 
 

 
 serialVersionUID:  3054842574468790421L

 
   
 
 
  
 Serialized Fields   
 
 

 
m_NormalizeDimWidths 
 
boolean  m_NormalizeDimWidths  
 
 Should we normalize the widths(ranges) of the dimensions (attributes) 
 before selecting the widest one.
 
 
 
 

 
   
 
 
  
 Class  weka.core.neighboursearch.balltrees.MiddleOutConstructor  extends  BallTreeConstructor  implements Serializable   
 
 

 
 serialVersionUID:  -8523314263062524462L

 
   
 
 
  
 Serialized Fields   
 
 

 
m_RSeed 
 
int  m_RSeed  
 
 Seed form random number generator.
 
 
 
 
 
 
rand 
 
java.util.Random  rand  
 
 The random number generator for selecting 
 the first anchor point randomly 
 (if selecting randomly).
 
 
 
 
 
 
rootRadius 
 
double  rootRadius  
 
 The radius of the smallest ball enclosing all the data points.
 
 
 
 
 
 
m_RandomInitialAnchor 
 
boolean  m_RandomInitialAnchor  
 
 True if the initial anchor is chosen randomly. False if it is the furthest
 point from the mean/centroid.
 
 
 
 

 
   
 
 
  
 Class weka.core.neighboursearch.balltrees.MiddleOutConstructor.MyIdxList extends  FastVector  implements Serializable   
 
 

 
 serialVersionUID:  -2283869109722934927L

 

 
   
 
 
  
 Class  weka.core.neighboursearch.balltrees.PointsClosestToFurthestChildren  extends  BallSplitter  implements Serializable   
 
 

 
 serialVersionUID:  -2947177543565818260L

 

 
   
 
 
  
 Class  weka.core.neighboursearch.balltrees.TopDownConstructor  extends  BallTreeConstructor  implements Serializable   
 
 

 
 serialVersionUID:  -5150140645091889979L

 
   
 
 
  
 Serialized Fields   
 
 

 
m_Splitter 
 
 BallSplitter   m_Splitter  
 
 The BallSplitter algorithm used by the TopDown BallTree constructor, if it 
 is selected.
 
 
 
 
 

 
 
  
 Package   weka.core.neighboursearch.covertrees   
 
 

 
   
 
 
  
 Class  weka.core.neighboursearch.covertrees.Stack  extends java.lang.Object implements Serializable   
 
 

 
 serialVersionUID:  5604056321825539264L

 
   
 
 
  
 Serialized Fields   
 
 

 
length 
 
int  length  
 
 The number of elements in the stack.
 
 
 
 
 
 
elements 
 
java.util.ArrayList<E>  elements  
 
 The elements inside the stack.
 
 
 
 
 

 
 
  
 Package   weka.core.neighboursearch.kdtrees   
 
 

 
   
 
 
  
 Class  weka.core.neighboursearch.kdtrees.KDTreeNode  extends java.lang.Object implements Serializable   
 
 

 
 serialVersionUID:  -3660396067582792648L

 
   
 
 
  
 Serialized Fields   
 
 

 
m_NodeNumber 
 
int  m_NodeNumber  
 
 node number (only for debug).
 
 
 
 
 
 
m_Left 
 
 KDTreeNode   m_Left  
 
 left subtree; contains instances with smaller or equal to split value.
 
 
 
 
 
 
m_Right 
 
 KDTreeNode   m_Right  
 
 right subtree; contains instances with larger than split value.
 
 
 
 
 
 
m_SplitValue 
 
double  m_SplitValue  
 
 value to split on.
 
 
 
 
 
 
m_SplitDim 
 
int  m_SplitDim  
 
 attribute to split on.
 
 
 
 
 
 
m_NodeRanges 
 
double[][]  m_NodeRanges  
 
 lowest and highest value and width (= high - low) for each
 dimension.
 
 
 
 
 
 
m_NodesRectBounds 
 
double[][]  m_NodesRectBounds  
 
 The lo and high bounds of the hyper rectangle described by the
 node.
 
 
 
 
 
 
m_Start 
 
int  m_Start  
 
 The start index of the portion of the master index array, 
 which stores the indices of the instances/points the node 
 contains.
 
 
 
 
 
 
m_End 
 
int  m_End  
 
 The end index of the portion of the master index array, 
 which stores indices of the instances/points the node 
 contains.
 
 
 
 

 
   
 
 
  
 Class  weka.core.neighboursearch.kdtrees.KDTreeNodeSplitter  extends java.lang.Object implements Serializable   
 
 

 
   
 
 
  
 Serialized Fields   
 
 

 
m_Instances 
 
 Instances   m_Instances  
 
 The instances that'll be used for tree construction.
 
 
 
 
 
 
m_EuclideanDistance 
 
 EuclideanDistance   m_EuclideanDistance  
 
 The distance function used for building the tree.
 
 
 
 
 
 
m_InstList 
 
int[]  m_InstList  
 
 The master index array that'll be reshuffled as nodes
 are split and the tree is constructed.
 
 
 
 
 
 
m_NormalizeNodeWidth 
 
boolean  m_NormalizeNodeWidth  
 
 Stores whether if the width of a KDTree
 node is normalized or not.
 
 
 
 

 
   
 
 
  
 Class  weka.core.neighboursearch.kdtrees.KMeansInpiredMethod  extends  KDTreeNodeSplitter  implements Serializable   
 
 

 
 serialVersionUID:  -866783749124714304L

 

 
   
 
 
  
 Class  weka.core.neighboursearch.kdtrees.MedianOfWidestDimension  extends  KDTreeNodeSplitter  implements Serializable   
 
 

 
 serialVersionUID:  1383443320160540663L

 

 
   
 
 
  
 Class  weka.core.neighboursearch.kdtrees.MidPointOfWidestDimension  extends  KDTreeNodeSplitter  implements Serializable   
 
 

 
 serialVersionUID:  -7617277960046591906L

 

 
   
 
 
  
 Class  weka.core.neighboursearch.kdtrees.SlidingMidPointOfWidestSide  extends  KDTreeNodeSplitter  implements Serializable   
 
 

 
 serialVersionUID:  852857628205680562L

 
 

 
 
  
 Package   weka.core.pmml   
 
 

 
   
 
 
  
 Class  weka.core.pmml.BuiltInArithmetic  extends  Function  implements Serializable   
 
 

 
 serialVersionUID:  2275009453597279459L

 
   
 
 
  
 Serialized Fields   
 
 

 
m_operator 
 
weka.core.pmml.BuiltInArithmetic.Operator  m_operator  
 
 The operator for this function
 
 
 
 

 
   
 
 
  
 Class  weka.core.pmml.BuiltInMath  extends  Function  implements Serializable   
 
 

 
 serialVersionUID:  -8092338695602573652L

 
   
 
 
  
 Serialized Fields   
 
 

 
m_func 
 
weka.core.pmml.BuiltInMath.MathFunc  m_func  
 
 The function to apply
 
 
 
 

 
   
 
 
  
 Class  weka.core.pmml.BuiltInString  extends  Function  implements Serializable   
 
 

 
 serialVersionUID:  -7391516909331728653L

 
   
 
 
  
 Serialized Fields   
 
 

 
m_func 
 
weka.core.pmml.BuiltInString.StringFunc  m_func  
 
 The function to apply
 
 
 
 
 
 
m_outputDef 
 
 Attribute   m_outputDef  
 
 The output structure produced by this function
 
 
 
 

 
   
 
 
  
 Class  weka.core.pmml.Constant  extends  Expression  implements Serializable   
 
 

 
 serialVersionUID:  -304829687822452424L

 
   
 
 
  
 Serialized Fields   
 
 

 
m_categoricalConst 
 
java.lang.String  m_categoricalConst  
 
 
 
 
 
 
m_continuousConst 
 
double  m_continuousConst  
 
 
 
 

 
   
 
 
  
 Class  weka.core.pmml.DefineFunction  extends  Function  implements Serializable   
 
 

 
 serialVersionUID:  -1976646917527243888L

 
   
 
 
  
 Serialized Fields   
 
 

 
m_parameters 
 
java.util.ArrayList<E>  m_parameters  
 
 The list of parameters expected by this function. We can use this to do 
 some error/type checking when users call setParameterDefs() on us
 
 
 
 
 
 
m_optype 
 
 FieldMetaInfo.Optype   m_optype  
 
 The optype for this function
 
 
 
 
 
 
m_expression 
 
 Expression   m_expression  
 
 The Expression for this function to use
 
 
 
 

 
   
 
 
  
 Class weka.core.pmml.DefineFunction.ParameterField extends  FieldMetaInfo  implements Serializable   
 
 

 
 serialVersionUID:  3918895902507585558L

 

 
   
 
 
  
 Class  weka.core.pmml.DerivedFieldMetaInfo  extends  FieldMetaInfo  implements Serializable   
 
 

 
   
 
 
  
 Serialized Fields   
 
 

 
m_displayName 
 
java.lang.String  m_displayName  
 
 display name
 
 
 
 
 
 
m_values 
 
java.util.ArrayList<E>  m_values  
 
 the list of values (if the field is ordinal) - may be of size zero if none are specified.
 If none are specified, we may be able to construct this by querying the Expression in
 this derived field
 
 
 
 
 
 
m_expression 
 
 Expression   m_expression  
 
 the single expression that defines the value of this field
 
 
 
 

 
   
 
 
  
 Class  weka.core.pmml.Discretize  extends  Expression  implements Serializable   
 
 

 
   
 
 
  
 Serialized Fields   
 
 

 
m_fieldName 
 
java.lang.String  m_fieldName  
 
 The name of the field to be discretized
 
 
 
 
 
 
m_fieldIndex 
 
int  m_fieldIndex  
 
 The index of the field
 
 
 
 
 
 
m_mapMissingDefined 
 
boolean  m_mapMissingDefined  
 
 True if a replacement for missing values has been specified
 
 
 
 
 
 
m_mapMissingTo 
 
java.lang.String  m_mapMissingTo  
 
 The value of the missing value replacement (if defined)
 
 
 
 
 
 
m_defaultValueDefined 
 
boolean  m_defaultValueDefined  
 
 True if a default value has been specified
 
 
 
 
 
 
m_defaultValue 
 
java.lang.String  m_defaultValue  
 
 The default value (if defined)
 
 
 
 
 
 
m_bins 
 
java.util.ArrayList<E>  m_bins  
 
 The bins for this discretization
 
 
 
 
 
 
m_outputDef 
 
 Attribute   m_outputDef  
 
 The output structure of this discretization
 
 
 
 

 
   
 
 
  
 Class weka.core.pmml.Discretize.DiscretizeBin extends java.lang.Object implements Serializable   
 
 

 
 serialVersionUID:  5810063243316808400L

 
   
 
 
  
 Serialized Fields   
 
 

 
m_intervals 
 
java.util.ArrayList<E>  m_intervals  
 
 The intervals for this DiscretizeBin
 
 
 
 
 
 
m_binValue 
 
java.lang.String  m_binValue  
 
 The bin value for this DiscretizeBin
 
 
 
 

 
   
 
 
  
 Class  weka.core.pmml.Expression  extends java.lang.Object implements Serializable   
 
 

 
 serialVersionUID:  4448840549804800321L

 
   
 
 
  
 Serialized Fields   
 
 

 
m_opType 
 
 FieldMetaInfo.Optype   m_opType  
 
 The optype of this Expression
 
 
 
 
 
 
m_fieldDefs 
 
java.util.ArrayList<E>  m_fieldDefs  
 
 The field defs
 
 
 
 

 
   
 
 
  
 Class  weka.core.pmml.FieldMetaInfo  extends java.lang.Object implements Serializable   
 
 

 
   
 
 
  
 Serialized Fields   
 
 

 
m_fieldName 
 
java.lang.String  m_fieldName  
 
 the name of the field
 
 
 
 
 
 
m_optype 
 
 FieldMetaInfo.Optype   m_optype  
 
 The optype for the target
 
 
 
 

 
   
 
 
  
 Class  weka.core.pmml.FieldMetaInfo.Interval  extends java.lang.Object implements Serializable   
 
 

 
 serialVersionUID:  -7339790632684638012L

 
   
 
 
  
 Serialized Fields   
 
 

 
m_leftMargin 
 
double  m_leftMargin  
 
 The left boundary value
 
 
 
 
 
 
m_rightMargin 
 
double  m_rightMargin  
 
 The right boundary value
 
 
 
 
 
 
m_closure 
 
 FieldMetaInfo.Interval.Closure   m_closure  
 
 
 
 

 
   
 
 
  
 Class  weka.core.pmml.FieldMetaInfo.Value  extends java.lang.Object implements Serializable   
 
 

 
 serialVersionUID:  -3981030320273649739L

 
   
 
 
  
 Serialized Fields   
 
 

 
m_value 
 
java.lang.String  m_value  
 
 The value
 
 
 
 
 
 
m_displayValue 
 
java.lang.String  m_displayValue  
 
 The display value (might hold a human readable value - e.g.
 product name instead of cryptic code).
 
 
 
 
 
 
m_property 
 
 FieldMetaInfo.Value.Property   m_property  
 
 
 
 

 
   
 
 
  
 Class  weka.core.pmml.FieldRef  extends  Expression  implements Serializable   
 
 

 
 serialVersionUID:  -8009605897876168409L

 
   
 
 
  
 Serialized Fields   
 
 

 
m_fieldName 
 
java.lang.String  m_fieldName  
 
 The name of the field to reference
 
 
 
 

 
   
 
 
  
 Class  weka.core.pmml.Function  extends java.lang.Object implements Serializable   
 
 

 
 serialVersionUID:  -6997738288201933171L

 
   
 
 
  
 Serialized Fields   
 
 

 
m_functionName 
 
java.lang.String  m_functionName  
 
 The name of this function
 
 
 
 
 
 
m_parameterDefs 
 
java.util.ArrayList<E>  m_parameterDefs  
 
 The structure of the parameters to this function
 
 
 
 

 
   
 
 
  
 Class  weka.core.pmml.MappingInfo  extends java.lang.Object implements Serializable   
 
 

 
   
 
 
  
 Serialized Fields   
 
 

 
m_fieldsMap 
 
int[]  m_fieldsMap  
 
 Map the incoming attributes to the mining schema attributes.
 Each entry holds the index of the incoming attribute that
 corresponds to this mining schema attribute.
 
 
 
 
 
 
m_nominalValueMaps 
 
int[][]  m_nominalValueMaps  
 
 Map indexes for nominal values in incoming structure to those
 in the mining schema. There will be as many entries as there are
 attributes in this array. Non-nominal attributes will have
 null entries. Each non-null entry is an array of integer indexes.
 Each entry in a given array (for a given attribute) holds the index of
 the mining schema value that corresponds to this incoming value.
 UNKNOWN_NOMINAL_VALUE is used as the index for those incoming values 
 that are not defined in the mining schema.
 
 
 
 
 
 
m_fieldsMappingText 
 
java.lang.String  m_fieldsMappingText  
 
 Holds a textual description of the fields mapping
 
 
 
 
 
 
m_log 
 
 Logger   m_log  
 
 For logging
 
 
 
 

 
   
 
 
  
 Class  weka.core.pmml.MiningFieldMetaInfo  extends  FieldMetaInfo  implements Serializable   
 
 

 
 serialVersionUID:  -1256774332779563185L

 
   
 
 
  
 Serialized Fields   
 
 

 
m_usageType 
 
weka.core.pmml.MiningFieldMetaInfo.Usage  m_usageType  
 
 usage type
 
 
 
 
 
 
m_outlierTreatmentMethod 
 
weka.core.pmml.MiningFieldMetaInfo.Outlier  m_outlierTreatmentMethod  
 
 outlier treatmemnt method
 
 
 
 
 
 
m_lowValue 
 
double  m_lowValue  
 
 outlier low value
 
 
 
 
 
 
m_highValue 
 
double  m_highValue  
 
 outlier high value
 
 
 
 
 
 
m_missingValueTreatmentMethod 
 
weka.core.pmml.MiningFieldMetaInfo.Missing  m_missingValueTreatmentMethod  
 
 missing values treatment method
 
 
 
 
 
 
m_missingValueReplacementNominal 
 
java.lang.String  m_missingValueReplacementNominal  
 
 actual missing value replacements (if specified)
 
 
 
 
 
 
m_missingValueReplacementNumeric 
 
double  m_missingValueReplacementNumeric  
 
 
 
 
 
 
m_optypeOverride 
 
 FieldMetaInfo.Optype   m_optypeOverride  
 
 optype overrides (override data dictionary type - NOT SUPPORTED AT PRESENT)
 
 
 
 
 
 
m_index 
 
int  m_index  
 
 the index of the field in the mining schema Instances
 
 
 
 
 
 
m_importance 
 
double  m_importance  
 
 importance (if defined)
 
 
 
 
 
 
m_miningSchemaI 
 
 Instances   m_miningSchemaI  
 
 mining schema (needed for toString method)
 
 
 
 

 
   
 
 
  
 Class  weka.core.pmml.MiningSchema  extends java.lang.Object implements Serializable   
 
 

 
 serialVersionUID:  7144380586726330455L

 
   
 
 
  
 Serialized Fields   
 
 

 
m_fieldInstancesStructure 
 
 Instances   m_fieldInstancesStructure  
 
 The structure of all the fields (both mining schema and derived) as Instances
 
 
 
 
 
 
m_miningSchemaInstancesStructure 
 
 Instances   m_miningSchemaInstancesStructure  
 
 Just the mining schema fields as Instances
 
 
 
 
 
 
m_miningMeta 
 
java.util.ArrayList<E>  m_miningMeta  
 
 Meta information about the mining schema fields
 
 
 
 
 
 
m_derivedMeta 
 
java.util.ArrayList<E>  m_derivedMeta  
 
 Meta information about derived fields (those defined in
 the TransformationDictionary followed by those defined in
 LocalTransformations)
 
 
 
 
 
 
m_transformationDictionary 
 
weka.core.pmml.TransformationDictionary  m_transformationDictionary  
 
 The transformation dictionary (if defined)
 
 
 
 
 
 
m_targetMetaInfo 
 
 TargetMetaInfo   m_targetMetaInfo  
 
 target meta info (may be null if not defined)
 
 
 
 

 
   
 
 
  
 Class  weka.core.pmml.NormContinuous  extends  Expression  implements Serializable   
 
 

 
 serialVersionUID:  4714332374909851542L

 
   
 
 
  
 Serialized Fields   
 
 

 
m_fieldName 
 
java.lang.String  m_fieldName  
 
 The name of the field to use
 
 
 
 
 
 
m_fieldIndex 
 
int  m_fieldIndex  
 
 The index of the field
 
 
 
 
 
 
m_mapMissingDefined 
 
boolean  m_mapMissingDefined  
 
 True if a replacement for missing values has been specified
 
 
 
 
 
 
m_mapMissingTo 
 
double  m_mapMissingTo  
 
 The value of the missing value replacement (if defined)
 
 
 
 
 
 
m_outlierTreatmentMethod 
 
weka.core.pmml.MiningFieldMetaInfo.Outlier  m_outlierTreatmentMethod  
 
 Outlier treatment method (default = asIs)
 
 
 
 
 
 
m_linearNormOrig 
 
double[]  m_linearNormOrig  
 
 original values for the LinearNorm entries
 
 
 
 
 
 
m_linearNormNorm 
 
double[]  m_linearNormNorm  
 
 norm values for the LinearNorm entries
 
 
 
 

 
   
 
 
  
 Class  weka.core.pmml.NormDiscrete  extends  Expression  implements Serializable   
 
 

 
 serialVersionUID:  -8854409417983908220L

 
   
 
 
  
 Serialized Fields   
 
 

 
m_fieldName 
 
java.lang.String  m_fieldName  
 
 The name of the field to lookup our value in
 
 
 
 
 
 
m_field 
 
 Attribute   m_field  
 
 The actual attribute itself
 
 
 
 
 
 
m_fieldIndex 
 
int  m_fieldIndex  
 
 The index of the attribute
 
 
 
 
 
 
m_fieldValue 
 
java.lang.String  m_fieldValue  
 
 The actual value (as a String) that will correspond to an output of 1
 
 
 
 
 
 
m_mapMissingDefined 
 
boolean  m_mapMissingDefined  
 
 True if a replacement for missing values has been specified
 
 
 
 
 
 
m_mapMissingTo 
 
double  m_mapMissingTo  
 
 The value of the missing value replacement (if defined)
 
 
 
 
 
 
m_fieldValueIndex 
 
int  m_fieldValueIndex  
 
 If we are referring to a nominal (rather than String) attribute
 then this holds the index of the value in question. Will be faster
 than searching for the value each time.
 
 
 
 

 
   
 
 
  
 Class  weka.core.pmml.TargetMetaInfo  extends  FieldMetaInfo  implements Serializable   
 
 

 
 serialVersionUID:  863500462237904927L

 
   
 
 
  
 Serialized Fields   
 
 

 
m_min 
 
double  m_min  
 
 min and max
 
 
 
 
 
 
m_max 
 
double  m_max  
 
 
 
 
 
 
m_rescaleConstant 
 
double  m_rescaleConstant  
 
 re-scaling of target value (if defined)
 
 
 
 
 
 
m_rescaleFactor 
 
double  m_rescaleFactor  
 
 
 
 
 
 
m_castInteger 
 
java.lang.String  m_castInteger  
 
 cast integers (default no casting)
 
 
 
 
 
 
m_defaultValueOrPriorProbs 
 
double[]  m_defaultValueOrPriorProbs  
 
 default value (numeric) or prior distribution (categorical)
 
 
 
 
 
 
m_values 
 
java.util.ArrayList<E>  m_values  
 
 for categorical values. Actual values
 
 
 
 
 
 
m_displayValues 
 
java.util.ArrayList<E>  m_displayValues  
 
 corresponding display values
 
 
 
 
 

 
 
  
 Package   weka.core.stemmers   
 
 

 
   
 
 
  
 Class  weka.core.stemmers.IteratedLovinsStemmer  extends  LovinsStemmer  implements Serializable   
 
 

 
 serialVersionUID:  960689687163788264L

 

 
   
 
 
  
 Class  weka.core.stemmers.LovinsStemmer  extends java.lang.Object implements Serializable   
 
 

 
 serialVersionUID:  -6113024782588197L

 

 
   
 
 
  
 Class  weka.core.stemmers.NullStemmer  extends java.lang.Object implements Serializable   
 
 

 
 serialVersionUID:  -3671261636532625496L

 

 
   
 
 
  
 Class  weka.core.stemmers.SnowballStemmer  extends java.lang.Object implements Serializable   
 
 

 
 serialVersionUID:  -6111170431963015178L

 
   
 
 
  
 Serialized Fields   
 
 

 
m_Stemmer 
 
java.lang.Object  m_Stemmer  
 
 the current stemmer.
 
 
 
 
 

 
 
  
 Package   weka.core.tokenizers   
 
 

 
   
 
 
  
 Class  weka.core.tokenizers.AlphabeticTokenizer  extends  Tokenizer  implements Serializable   
 
 

 
 serialVersionUID:  6705199562609861697L

 
   
 
 
  
 Serialized Fields   
 
 

 
m_Str 
 
char[]  m_Str  
 
 the characters of the string
 
 
 
 
 
 
m_CurrentPos 
 
int  m_CurrentPos  
 
 the current position
 
 
 
 

 
   
 
 
  
 Class  weka.core.tokenizers.CharacterDelimitedTokenizer  extends  Tokenizer  implements Serializable   
 
 

 
   
 
 
  
 Serialized Fields   
 
 

 
m_Delimiters 
 
java.lang.String  m_Delimiters  
 
 Delimiters used in tokenization
 
 
 
 

 
   
 
 
  
 Class  weka.core.tokenizers.NGramTokenizer  extends  CharacterDelimitedTokenizer  implements Serializable   
 
 

 
 serialVersionUID:  -2181896254171647219L

 
   
 
 
  
 Serialized Fields   
 
 

 
m_NMax 
 
int  m_NMax  
 
 the maximum number of N
 
 
 
 
 
 
m_NMin 
 
int  m_NMin  
 
 the minimum number of N
 
 
 
 
 
 
m_N 
 
int  m_N  
 
 the current length of the N-grams
 
 
 
 
 
 
m_MaxPosition 
 
int  m_MaxPosition  
 
 the number of strings available
 
 
 
 
 
 
m_CurrentPosition 
 
int  m_CurrentPosition  
 
 the current position for returning elements
 
 
 
 
 
 
m_SplitString 
 
java.lang.String[]  m_SplitString  
 
 all the available grams
 
 
 
 

 
   
 
 
  
 Class  weka.core.tokenizers.Tokenizer  extends java.lang.Object implements Serializable   
 
 

 

 
   
 
 
  
 Class  weka.core.tokenizers.WordTokenizer  extends  CharacterDelimitedTokenizer  implements Serializable   
 
 

 
 serialVersionUID:  -930893034037880773L

 
 

 
 
  
 Package   weka.core.xml   
 
 

 
   
 
 
  
 Class  weka.core.xml.XMLInstances  extends  XMLDocument  implements Serializable   
 
 

 
 serialVersionUID:  3626821327547416099L

 
   
 
 
  
 Serialized Fields   
 
 

 
m_Precision 
 
int  m_Precision  
 
 the precision for numbers
 
 
 
 
 
 
m_Instances 
 
 Instances   m_Instances  
 
 the underlying Instances
 
 
 
 
 

 
 
  
 Package   weka.datagenerators   
 
 

 
   
 
 
  
 Class  weka.datagenerators.ClassificationGenerator  extends  DataGenerator  implements Serializable   
 
 

 
 serialVersionUID:  -5261662546673517844L

 
   
 
 
  
 Serialized Fields   
 
 

 
m_NumExamples 
 
int  m_NumExamples  
 
 Number of instances
 
 
 
 

 
   
 
 
  
 Class  weka.datagenerators.ClusterDefinition  extends java.lang.Object implements Serializable   
 
 

 
 serialVersionUID:  -5950001207047429961L

 
   
 
 
  
 Serialized Fields   
 
 

 
m_Parent 
 
 ClusterGenerator   m_Parent  
 
 the parent of the cluster
 
 
 
 

 
   
 
 
  
 Class  weka.datagenerators.ClusterGenerator  extends  DataGenerator  implements Serializable   
 
 

 
 serialVersionUID:  6131722618472046365L

 
   
 
 
  
 Serialized Fields   
 
 

 
m_NumAttributes 
 
int  m_NumAttributes  
 
 Number of attribute the dataset should have
 
 
 
 
 
 
m_ClassFlag 
 
boolean  m_ClassFlag  
 
 class flag
 
 
 
 
 
 
m_booleanCols 
 
 Range   m_booleanCols  
 
 Stores which columns are boolean (default numeric)
 
 
 
 
 
 
m_nominalCols 
 
 Range   m_nominalCols  
 
 Stores which columns are nominal (default numeric)
 
 
 
 

 
   
 
 
  
 Class  weka.datagenerators.DataGenerator  extends java.lang.Object implements Serializable   
 
 

 
 serialVersionUID:  -3698585946221802578L

 
   
 
 
  
 Serialized Fields   
 
 

 
m_Debug 
 
boolean  m_Debug  
 
 Debugging mode
 
 
 
 
 
 
m_DatasetFormat 
 
 Instances   m_DatasetFormat  
 
 The format for the generated dataset
 
 
 
 
 
 
m_RelationName 
 
java.lang.String  m_RelationName  
 
 Relation name the dataset should have
 
 
 
 
 
 
m_NumExamplesAct 
 
int  m_NumExamplesAct  
 
 Number of instances that should be produced into the dataset 
 this number is by default m_NumExamples,
 but can be reset by the generator
 
 
 
 
 
 
m_Seed 
 
int  m_Seed  
 
 random number generator seed
 
 
 
 
 
 
m_Random 
 
java.util.Random  m_Random  
 
 random number generator
 
 
 
 
 
 
m_CreatingRelationName 
 
boolean  m_CreatingRelationName  
 
 flag, that indicates whether the relationname is currently assembled
 
 
 
 

 
   
 
 
  
 Class  weka.datagenerators.RegressionGenerator  extends  DataGenerator  implements Serializable   
 
 

 
 serialVersionUID:  3073254041275658221L

 
   
 
 
  
 Serialized Fields   
 
 

 
m_NumExamples 
 
int  m_NumExamples  
 
 Number of instances
 
 
 
 

 
   
 
 
  
 Class  weka.datagenerators.Test  extends java.lang.Object implements Serializable   
 
 

 
 serialVersionUID:  -8890645875887157782L

 
   
 
 
  
 Serialized Fields   
 
 

 
m_AttIndex 
 
int  m_AttIndex  
 
 the attribute index
 
 
 
 
 
 
m_Split 
 
double  m_Split  
 
 the split
 
 
 
 
 
 
m_Not 
 
boolean  m_Not  
 
 whether to negate the test
 
 
 
 
 
 
m_Dataset 
 
 Instances   m_Dataset  
 
 the dataset
 
 
 
 
 

 
 
  
 Package   weka.datagenerators.classifiers.classification   
 
 

 
   
 
 
  
 Class  weka.datagenerators.classifiers.classification.Agrawal  extends  ClassificationGenerator  implements Serializable   
 
 

 
 serialVersionUID:  2254651939636143025L

 
   
 
 
  
 Serialized Fields   
 
 

 
m_Function 
 
int  m_Function  
 
 the function to use for generating the data
 
 
 
 
 
 
m_BalanceClass 
 
boolean  m_BalanceClass  
 
 whether to balance the class
 
 
 
 
 
 
m_PerturbationFraction 
 
double  m_PerturbationFraction  
 
 the perturabation fraction
 
 
 
 
 
 
m_nextClassShouldBeZero 
 
boolean  m_nextClassShouldBeZero  
 
 used for balancing the class
 
 
 
 
 
 
m_lastLabel 
 
double  m_lastLabel  
 
 the last class label that was generated
 
 
 
 

 
   
 
 
  
 Class  weka.datagenerators.classifiers.classification.BayesNet  extends  ClassificationGenerator  implements Serializable   
 
 

 
 serialVersionUID:  -796118162379901512L

 
   
 
 
  
 Serialized Fields   
 
 

 
m_Generator 
 
 BayesNetGenerator   m_Generator  
 
 the bayesian net generator, that produces the actual data
 
 
 
 

 
   
 
 
  
 Class  weka.datagenerators.classifiers.classification.LED24  extends  ClassificationGenerator  implements Serializable   
 
 

 
 serialVersionUID:  -7880209100415868737L

 
   
 
 
  
 Serialized Fields   
 
 

 
m_NoisePercent 
 
double  m_NoisePercent  
 
 the noise rate
 
 
 
 
 
 
m_numIrrelevantAttributes 
 
int  m_numIrrelevantAttributes  
 
 used for generating the output, i.e., the additional noise attributes
 
 
 
 

 
   
 
 
  
 Class  weka.datagenerators.classifiers.classification.RandomRBF  extends  ClassificationGenerator  implements Serializable   
 
 

 
 serialVersionUID:  6069033710635728720L

 
   
 
 
  
 Serialized Fields   
 
 

 
m_NumAttributes 
 
int  m_NumAttributes  
 
 Number of attribute the dataset should have
 
 
 
 
 
 
m_NumClasses 
 
int  m_NumClasses  
 
 Number of Classes the dataset should have
 
 
 
 
 
 
m_NumCentroids 
 
int  m_NumCentroids  
 
 the number of centroids to use for generation
 
 
 
 
 
 
m_centroids 
 
double[][]  m_centroids  
 
 the centroids
 
 
 
 
 
 
m_centroidClasses 
 
int[]  m_centroidClasses  
 
 the classes of the centroids
 
 
 
 
 
 
m_centroidWeights 
 
double[]  m_centroidWeights  
 
 the weights of the centroids
 
 
 
 
 
 
m_centroidStdDevs 
 
double[]  m_centroidStdDevs  
 
 the stddevs of the centroids
 
 
 
 

 
   
 
 
  
 Class  weka.datagenerators.classifiers.classification.RDG1  extends  ClassificationGenerator  implements Serializable   
 
 

 
 serialVersionUID:  7751005204635320414L

 
   
 
 
  
 Serialized Fields   
 
 

 
m_NumAttributes 
 
int  m_NumAttributes  
 
 Number of attribute the dataset should have
 
 
 
 
 
 
m_NumClasses 
 
int  m_NumClasses  
 
 Number of Classes the dataset should have
 
 
 
 
 
 
m_MaxRuleSize 
 
int  m_MaxRuleSize  
 
 maximum rule size
 
 
 
 
 
 
m_MinRuleSize 
 
int  m_MinRuleSize  
 
 minimum rule size
 
 
 
 
 
 
m_NumIrrelevant 
 
int  m_NumIrrelevant  
 
 number of irrelevant attributes.
 
 
 
 
 
 
m_NumNumeric 
 
int  m_NumNumeric  
 
 number of numeric attribute
 
 
 
 
 
 
m_VoteFlag 
 
boolean  m_VoteFlag  
 
 flag that stores if voting is wished
 
 
 
 
 
 
m_DecisionList 
 
 FastVector   m_DecisionList  
 
 decision list
 
 
 
 
 
 
m_AttList_Irr 
 
boolean[]  m_AttList_Irr  
 
 array defines which attributes are irrelevant, with:
 true = attribute is irrelevant; false = attribute is not irrelevant
 
 
 
 
 

 
 
  
 Package   weka.datagenerators.classifiers.regression   
 
 

 
   
 
 
  
 Class  weka.datagenerators.classifiers.regression.Expression  extends  MexicanHat  implements Serializable   
 
 

 
 serialVersionUID:  -4237047357682277211L

 
   
 
 
  
 Serialized Fields   
 
 

 
m_Expression 
 
java.lang.String  m_Expression  
 
 the expression for computing y
 
 
 
 
 
 
m_Filter 
 
 AddExpression   m_Filter  
 
 the filter for generating y out of x
 
 
 
 
 
 
m_RawData 
 
 Instances   m_RawData  
 
 the input data structure for the filter
 
 
 
 

 
   
 
 
  
 Class  weka.datagenerators.classifiers.regression.MexicanHat  extends  RegressionGenerator  implements Serializable   
 
 

 
 serialVersionUID:  4577016375261512975L

 
   
 
 
  
 Serialized Fields   
 
 

 
m_Amplitude 
 
double  m_Amplitude  
 
 the amplitude of y
 
 
 
 
 
 
m_MinRange 
 
double  m_MinRange  
 
 the lower boundary of the range, x is drawn from
 
 
 
 
 
 
m_MaxRange 
 
double  m_MaxRange  
 
 the upper boundary of the range, x is drawn from
 
 
 
 
 
 
m_NoiseRate 
 
double  m_NoiseRate  
 
 the rate of the gaussian noise
 
 
 
 
 
 
m_NoiseVariance 
 
double  m_NoiseVariance  
 
 the variance of the gaussian noise
 
 
 
 
 
 
m_NoiseRandom 
 
java.util.Random  m_NoiseRandom  
 
 the random number generator for the noise
 
 
 
 
 

 
 
  
 Package   weka.datagenerators.clusterers   
 
 

 
   
 
 
  
 Class  weka.datagenerators.clusterers.BIRCHCluster  extends  ClusterGenerator  implements Serializable   
 
 

 
 serialVersionUID:  -334820527230755027L

 
   
 
 
  
 Serialized Fields   
 
 

 
m_NumClusters 
 
int  m_NumClusters  
 
 Number of Clusters the dataset should have
 
 
 
 
 
 
m_MinInstNum 
 
int  m_MinInstNum  
 
 minimal number of instances per cluster (option N)
 
 
 
 
 
 
m_MaxInstNum 
 
int  m_MaxInstNum  
 
 maximal number of instances per cluster (option N)
 
 
 
 
 
 
m_MinRadius 
 
double  m_MinRadius  
 
 minimum radius (option R)
 
 
 
 
 
 
m_MaxRadius 
 
double  m_MaxRadius  
 
 maximum radius (option R)
 
 
 
 
 
 
m_Pattern 
 
int  m_Pattern  
 
 pattern (changed with options G or S)
 
 
 
 
 
 
m_DistMult 
 
double  m_DistMult  
 
 distance multiplier (option M)
 
 
 
 
 
 
m_NumCycles 
 
int  m_NumCycles  
 
 number of cycles (option C)
 
 
 
 
 
 
m_InputOrder 
 
int  m_InputOrder  
 
 input order (changed with option O)
 
 
 
 
 
 
m_NoiseRate 
 
double  m_NoiseRate  
 
 noise rate in percent (option P,  between 0 and 30)
 
 
 
 
 
 
m_ClusterList 
 
 FastVector   m_ClusterList  
 
 cluster list
 
 
 
 
 
 
m_GridSize 
 
int  m_GridSize  
 
 grid size
 
 
 
 
 
 
m_GridWidth 
 
double  m_GridWidth  
 
 grid width
 
 
 
 

 
   
 
 
  
 Class  weka.datagenerators.clusterers.SubspaceCluster  extends  ClusterGenerator  implements Serializable   
 
 

 
 serialVersionUID:  -3454999858505621128L

 
   
 
 
  
 Serialized Fields   
 
 

 
m_NoiseRate 
 
double  m_NoiseRate  
 
 noise rate in percent (option P,  between 0 and 30)
 
 
 
 
 
 
m_Clusters 
 
 ClusterDefinition []  m_Clusters  
 
 cluster list
 
 
 
 
 
 
m_numValues 
 
int[]  m_numValues  
 
 if nominal, store number of values
 
 
 
 
 
 
m_globalMinValue 
 
double[]  m_globalMinValue  
 
 store global min values
 
 
 
 
 
 
m_globalMaxValue 
 
double[]  m_globalMaxValue  
 
 store global max values
 
 
 
 

 
   
 
 
  
 Class  weka.datagenerators.clusterers.SubspaceClusterDefinition  extends  ClusterDefinition  implements Serializable   
 
 

 
 serialVersionUID:  3135678125044007231L

 
   
 
 
  
 Serialized Fields   
 
 

 
m_clustertype 
 
int  m_clustertype  
 
 cluster type
 
 
 
 
 
 
m_clustersubtype 
 
int  m_clustersubtype  
 
 cluster subtypes
 
 
 
 
 
 
m_numClusterAttributes 
 
int  m_numClusterAttributes  
 
 number of attributes the cluster is defined for
 
 
 
 
 
 
m_numInstances 
 
int  m_numInstances  
 
 number of instances for this cluster
 
 
 
 
 
 
m_MinInstNum 
 
int  m_MinInstNum  
 
 minimal number of instances for this cluster
 
 
 
 
 
 
m_MaxInstNum 
 
int  m_MaxInstNum  
 
 maximal number of instances for this cluster
 
 
 
 
 
 
m_AttrIndexRange 
 
 Range   m_AttrIndexRange  
 
 range of atttributes
 
 
 
 
 
 
m_attributes 
 
boolean[]  m_attributes  
 
 attributes of this cluster
 
 
 
 
 
 
m_attrIndices 
 
int[]  m_attrIndices  
 
 global indices of the attributes of the cluster
 
 
 
 
 
 
m_minValue 
 
double[]  m_minValue  
 
 ranges of each attribute (min); not used if gaussian
 
 
 
 
 
 
m_maxValue 
 
double[]  m_maxValue  
 
 ranges of each attribute (max); not used if gaussian
 
 
 
 
 
 
m_meanValue 
 
double[]  m_meanValue  
 
 mean ; only used if gaussian
 
 
 
 
 
 
m_stddevValue 
 
double[]  m_stddevValue  
 
 standarddev; only used if gaussian
 
 
 
 
 

 
 
  
 Package   weka.estimators   
 
 

 
   
 
 
  
 Class  weka.estimators.DiscreteEstimator  extends  Estimator  implements Serializable   
 
 

 
 serialVersionUID:  -5526486742612434779L

 
   
 
 
  
 Serialized Fields   
 
 

 
m_Counts 
 
double[]  m_Counts  
 
 Hold the counts
 
 
 
 
 
 
m_SumOfCounts 
 
double  m_SumOfCounts  
 
 Hold the sum of counts
 
 
 
 

 
   
 
 
  
 Class  weka.estimators.Estimator  extends java.lang.Object implements Serializable   
 
 

 
 serialVersionUID:  -5902411487362274342L

 
   
 
 
  
 Serialized Fields   
 
 

 
m_Debug 
 
boolean  m_Debug  
 
 Debugging mode
 
 
 
 
 
 
m_classValueIndex 
 
double  m_classValueIndex  
 
 The class value index is > -1 if subset is taken with specific class value only
 
 
 
 
 
 
m_noClass 
 
boolean  m_noClass  
 
 set if class is not important
 
 
 
 

 
   
 
 
  
 Class  weka.estimators.KernelEstimator  extends  Estimator  implements Serializable   
 
 

 
 serialVersionUID:  3646923563367683925L

 
   
 
 
  
 Serialized Fields   
 
 

 
m_Values 
 
double[]  m_Values  
 
 Vector containing all of the values seen
 
 
 
 
 
 
m_Weights 
 
double[]  m_Weights  
 
 Vector containing the associated weights
 
 
 
 
 
 
m_NumValues 
 
int  m_NumValues  
 
 Number of values stored in m_Weights and m_Values so far
 
 
 
 
 
 
m_SumOfWeights 
 
double  m_SumOfWeights  
 
 The sum of the weights so far
 
 
 
 
 
 
m_StandardDev 
 
double  m_StandardDev  
 
 The standard deviation
 
 
 
 
 
 
m_Precision 
 
double  m_Precision  
 
 The precision of data values
 
 
 
 
 
 
m_AllWeightsOne 
 
boolean  m_AllWeightsOne  
 
 Whether we can optimise the kernel summation
 
 
 
 

 
   
 
 
  
 Class  weka.estimators.MahalanobisEstimator  extends  Estimator  implements Serializable   
 
 

 
 serialVersionUID:  8950225468990043868L

 
   
 
 
  
 Serialized Fields   
 
 

 
m_CovarianceInverse 
 
 Matrix   m_CovarianceInverse  
 
 The inverse of the covariance matrix
 
 
 
 
 
 
m_Determinant 
 
double  m_Determinant  
 
 The determinant of the covariance matrix
 
 
 
 
 
 
m_ConstDelta 
 
double  m_ConstDelta  
 
 The difference between the conditioning value and the conditioning mean
 
 
 
 
 
 
m_ValueMean 
 
double  m_ValueMean  
 
 The mean of the values
 
 
 
 

 
   
 
 
  
 Class  weka.estimators.NormalEstimator  extends  Estimator  implements Serializable   
 
 

 
 serialVersionUID:  93584379632315841L

 
   
 
 
  
 Serialized Fields   
 
 

 
m_SumOfWeights 
 
double  m_SumOfWeights  
 
 The sum of the weights
 
 
 
 
 
 
m_SumOfValues 
 
double  m_SumOfValues  
 
 The sum of the values seen
 
 
 
 
 
 
m_SumOfValuesSq 
 
double  m_SumOfValuesSq  
 
 The sum of the values squared
 
 
 
 
 
 
m_Mean 
 
double  m_Mean  
 
 The current mean
 
 
 
 
 
 
m_StandardDev 
 
double  m_StandardDev  
 
 The current standard deviation
 
 
 
 
 
 
m_Precision 
 
double  m_Precision  
 
 The precision of numeric values ( = minimum std dev permitted)
 
 
 
 

 
   
 
 
  
 Class  weka.estimators.PoissonEstimator  extends  Estimator  implements Serializable   
 
 

 
 serialVersionUID:  7669362595289236662L

 
   
 
 
  
 Serialized Fields   
 
 

 
m_NumValues 
 
double  m_NumValues  
 
 The number of values seen
 
 
 
 
 
 
m_SumOfValues 
 
double  m_SumOfValues  
 
 The sum of the values seen
 
 
 
 
 
 
m_Lambda 
 
double  m_Lambda  
 
 The average number of times
 an event occurs in an interval.
 
 
 
 
 

 
 
  
 Package   weka.experiment   
 
 

 
   
 
 
  
 Class  weka.experiment.AveragingResultProducer  extends java.lang.Object implements Serializable   
 
 

 
 serialVersionUID:  2551284958501991352L

 
   
 
 
  
 Serialized Fields   
 
 

 
m_Instances 
 
 Instances   m_Instances  
 
 The dataset of interest
 
 
 
 
 
 
m_ResultListener 
 
 ResultListener   m_ResultListener  
 
 The ResultListener to send results to
 
 
 
 
 
 
m_ResultProducer 
 
 ResultProducer   m_ResultProducer  
 
 The ResultProducer used to generate results
 
 
 
 
 
 
m_AdditionalMeasures 
 
java.lang.String[]  m_AdditionalMeasures  
 
 The names of any additional measures to look for in SplitEvaluators
 
 
 
 
 
 
m_ExpectedResultsPerAverage 
 
int  m_ExpectedResultsPerAverage  
 
 The number of results expected to average over for each run
 
 
 
 
 
 
m_CalculateStdDevs 
 
boolean  m_CalculateStdDevs  
 
 True if standard deviation fields should be produced
 
 
 
 
 
 
m_CountFieldName 
 
java.lang.String  m_CountFieldName  
 
 The name of the field that will contain the number of results
 averaged over.
 
 
 
 
 
 
m_KeyFieldName 
 
java.lang.String  m_KeyFieldName  
 
 The name of the key field to average over
 
 
 
 
 
 
m_KeyIndex 
 
int  m_KeyIndex  
 
 The index of the field to average over in the resultproducers key
 
 
 
 
 
 
m_Keys 
 
 FastVector   m_Keys  
 
 Collects the keys from a single run
 
 
 
 
 
 
m_Results 
 
 FastVector   m_Results  
 
 Collects the results from a single run
 
 
 
 

 
   
 
 
  
 Class  weka.experiment.ClassifierSplitEvaluator  extends java.lang.Object implements Serializable   
 
 

 
 serialVersionUID:  -8511241602760467265L

 
   
 
 
  
 Serialized Fields   
 
 

 
m_Template 
 
 Classifier   m_Template  
 
 The template classifier
 
 
 
 
 
 
m_Classifier 
 
 Classifier   m_Classifier  
 
 The classifier used for evaluation
 
 
 
 
 
 
m_AdditionalMeasures 
 
java.lang.String[]  m_AdditionalMeasures  
 
 The names of any additional measures to look for in SplitEvaluators
 
 
 
 
 
 
m_doesProduce 
 
boolean[]  m_doesProduce  
 
 Array of booleans corresponding to the measures in m_AdditionalMeasures
      indicating which of the AdditionalMeasures the current classifier
      can produce
 
 
 
 
 
 
m_numberAdditionalMeasures 
 
int  m_numberAdditionalMeasures  
 
 The number of additional measures that need to be filled in
      after taking into account column constraints imposed by the final
      destination for results
 
 
 
 
 
 
m_result 
 
java.lang.String  m_result  
 
 Holds the statistics for the most recent application of the classifier
 
 
 
 
 
 
m_ClassifierOptions 
 
java.lang.String  m_ClassifierOptions  
 
 The classifier options (if any)
 
 
 
 
 
 
m_ClassifierVersion 
 
java.lang.String  m_ClassifierVersion  
 
 The classifier version
 
 
 
 
 
 
m_IRclass 
 
int  m_IRclass  
 
 Class index for information retrieval statistics (default 0)
 
 
 
 
 
 
m_predTargetColumn 
 
boolean  m_predTargetColumn  
 
 Flag for prediction and target columns output.
 
 
 
 
 
 
m_attID 
 
int  m_attID  
 
 Attribute index of instance identifier (default -1)
 
 
 
 

 
   
 
 
  
 Class  weka.experiment.CostSensitiveClassifierSplitEvaluator  extends  ClassifierSplitEvaluator  implements Serializable   
 
 

 
 serialVersionUID:  -8069566663019501276L

 
   
 
 
  
 Serialized Fields   
 
 

 
m_OnDemandDirectory 
 
java.io.File  m_OnDemandDirectory  
 
 The directory used when loading cost files on demand, null indicates
 current directory
 
 
 
 

 
   
 
 
  
 Class  weka.experiment.CrossValidationResultProducer  extends java.lang.Object implements Serializable   
 
 

 
 serialVersionUID:  -1580053925080091917L

 
   
 
 
  
 Serialized Fields   
 
 

 
m_Instances 
 
 Instances   m_Instances  
 
 The dataset of interest
 
 
 
 
 
 
m_ResultListener 
 
 ResultListener   m_ResultListener  
 
 The ResultListener to send results to
 
 
 
 
 
 
m_NumFolds 
 
int  m_NumFolds  
 
 The number of folds in the cross-validation
 
 
 
 
 
 
m_debugOutput 
 
boolean  m_debugOutput  
 
 Save raw output of split evaluators --- for debugging purposes
 
 
 
 
 
 
m_ZipDest 
 
 OutputZipper   m_ZipDest  
 
 The output zipper to use for saving raw splitEvaluator output
 
 
 
 
 
 
m_OutputFile 
 
java.io.File  m_OutputFile  
 
 The destination output file/directory for raw output
 
 
 
 
 
 
m_SplitEvaluator 
 
 SplitEvaluator   m_SplitEvaluator  
 
 The SplitEvaluator used to generate results
 
 
 
 
 
 
m_AdditionalMeasures 
 
java.lang.String[]  m_AdditionalMeasures  
 
 The names of any additional measures to look for in SplitEvaluators
 
 
 
 

 
   
 
 
  
 Class  weka.experiment.CSVResultListener  extends java.lang.Object implements Serializable   
 
 

 
 serialVersionUID:  -623185072785174658L

 
   
 
 
  
 Serialized Fields   
 
 

 
m_RP 
 
 ResultProducer   m_RP  
 
 The ResultProducer sending us results
 
 
 
 
 
 
m_OutputFile 
 
java.io.File  m_OutputFile  
 
 The destination output file, null sends to System.out
 
 
 
 
 
 
m_OutputFileName 
 
java.lang.String  m_OutputFileName  
 
 The name of the output file. Empty for temporary file.
 
 
 
 

 
   
 
 
  
 Class  weka.experiment.DatabaseResultListener  extends  DatabaseUtils  implements Serializable   
 
 

 
 serialVersionUID:  7388014746954652818L

 
   
 
 
  
 Serialized Fields   
 
 

 
m_ResultProducer 
 
 ResultProducer   m_ResultProducer  
 
 The ResultProducer to listen to
 
 
 
 
 
 
m_ResultsTableName 
 
java.lang.String  m_ResultsTableName  
 
 The name of the current results table
 
 
 
 
 
 
m_Debug 
 
boolean  m_Debug  
 
 True if debugging output should be printed
 
 
 
 
 
 
m_CacheKeyName 
 
java.lang.String  m_CacheKeyName  
 
 Holds the name of the key field to cache upon, or null if no caching
 
 
 
 
 
 
m_CacheKeyIndex 
 
int  m_CacheKeyIndex  
 
 Stores the index of the key column holding the cache key data
 
 
 
 
 
 
m_CacheKey 
 
java.lang.Object[]  m_CacheKey  
 
 Stores the key for which the cache is valid
 
 
 
 
 
 
m_Cache 
 
 FastVector   m_Cache  
 
 Stores the cached values
 
 
 
 

 
   
 
 
  
 Class  weka.experiment.DatabaseResultProducer  extends  DatabaseResultListener  implements Serializable   
 
 

 
 serialVersionUID:  -5620660780203158666L

 
   
 
 
  
 Serialized Fields   
 
 

 
m_Instances 
 
 Instances   m_Instances  
 
 The dataset of interest
 
 
 
 
 
 
m_ResultListener 
 
 ResultListener   m_ResultListener  
 
 The ResultListener to send results to
 
 
 
 
 
 
m_ResultProducer 
 
 ResultProducer   m_ResultProducer  
 
 The ResultProducer used to generate results
 
 
 
 
 
 
m_AdditionalMeasures 
 
java.lang.String[]  m_AdditionalMeasures  
 
 The names of any additional measures to look for in SplitEvaluators
 
 
 
 

 
   
 
 
  
 Class  weka.experiment.DatabaseUtils  extends java.lang.Object implements Serializable   
 
 

 
 serialVersionUID:  -8252351994547116729L

 
   
 
 
  
 Serialized Fields   
 
 

 
DRIVERS 
 
java.util.Vector<E>  DRIVERS  
 
 Holds the jdbc drivers to be used (only to stop them being gc'ed).
 
 
 
 
 
 
PROPERTIES 
 
java.util.Properties  PROPERTIES  
 
 Properties associated with the database connection.
 
 
 
 
 
 
m_DatabaseURL 
 
java.lang.String  m_DatabaseURL  
 
 Database URL.
 
 
 
 
 
 
m_Debug 
 
boolean  m_Debug  
 
 True if debugging output should be printed.
 
 
 
 
 
 
m_userName 
 
java.lang.String  m_userName  
 
 Database username.
 
 
 
 
 
 
m_password 
 
java.lang.String  m_password  
 
 Database Password.
 
 
 
 
 
 
m_stringType 
 
java.lang.String  m_stringType  
 
 string type for the create table statement.
 
 
 
 
 
 
m_intType 
 
java.lang.String  m_intType  
 
 integer type for the create table statement.
 
 
 
 
 
 
m_doubleType 
 
java.lang.String  m_doubleType  
 
 double type for the create table statement.
 
 
 
 
 
 
m_checkForUpperCaseNames 
 
boolean  m_checkForUpperCaseNames  
 
 For databases where Tables and Columns are created in upper case.
 
 
 
 
 
 
m_checkForLowerCaseNames 
 
boolean  m_checkForLowerCaseNames  
 
 For databases where Tables and Columns are created in lower case.
 
 
 
 
 
 
m_setAutoCommit 
 
boolean  m_setAutoCommit  
 
 setAutoCommit on the database?
 
 
 
 
 
 
m_createIndex 
 
boolean  m_createIndex  
 
 create index on the database?
 
 
 
 
 
 
m_Keywords 
 
java.util.HashSet<E>  m_Keywords  
 
 the keywords for the current database type.
 
 
 
 
 
 
m_KeywordsMaskChar 
 
java.lang.String  m_KeywordsMaskChar  
 
 the character to mask SQL keywords (by appending this character).
 
 
 
 

 
   
 
 
  
 Class  weka.experiment.DensityBasedClustererSplitEvaluator  extends java.lang.Object implements Serializable   
 
 

 
   
 
 
  
 Serialized Fields   
 
 

 
m_removeClassColumn 
 
boolean  m_removeClassColumn  
 
 Remove the class column (if set) from the data
 
 
 
 
 
 
m_clusterer 
 
 DensityBasedClusterer   m_clusterer  
 
 The clusterer used for evaluation
 
 
 
 
 
 
m_additionalMeasures 
 
java.lang.String[]  m_additionalMeasures  
 
 The names of any additional measures to look for in SplitEvaluators
 
 
 
 
 
 
m_doesProduce 
 
boolean[]  m_doesProduce  
 
 Array of booleans corresponding to the measures in m_AdditionalMeasures
      indicating which of the AdditionalMeasures the current clusterer
      can produce
 
 
 
 
 
 
m_numberAdditionalMeasures 
 
int  m_numberAdditionalMeasures  
 
 The number of additional measures that need to be filled in
      after taking into account column constraints imposed by the final
      destination for results
 
 
 
 
 
 
m_result 
 
java.lang.String  m_result  
 
 Holds the statistics for the most recent application of the clusterer
 
 
 
 
 
 
m_clustererOptions 
 
java.lang.String  m_clustererOptions  
 
 The clusterer options (if any)
 
 
 
 
 
 
m_clustererVersion 
 
java.lang.String  m_clustererVersion  
 
 The clusterer version
 
 
 
 

 
   
 
 
  
 Class  weka.experiment.Experiment  extends java.lang.Object implements Serializable   
 
 

 
 serialVersionUID:  44945596742646663L

 
   
 
 
  
 Serialized Fields   
 
 

 
m_ResultListener 
 
 ResultListener   m_ResultListener  
 
 Where results will be sent
 
 
 
 
 
 
m_ResultProducer 
 
 ResultProducer   m_ResultProducer  
 
 The result producer
 
 
 
 
 
 
m_RunLower 
 
int  m_RunLower  
 
 Lower run number
 
 
 
 
 
 
m_RunUpper 
 
int  m_RunUpper  
 
 Upper run number
 
 
 
 
 
 
m_Datasets 
 
javax.swing.DefaultListModel  m_Datasets  
 
 An array of dataset files
 
 
 
 
 
 
m_UsePropertyIterator 
 
boolean  m_UsePropertyIterator  
 
 True if the exp should also iterate over a property of the RP
 
 
 
 
 
 
m_PropertyPath 
 
 PropertyNode []  m_PropertyPath  
 
 The path to the iterator property
 
 
 
 
 
 
m_PropertyArray 
 
java.lang.Object  m_PropertyArray  
 
 The array of values to set the property to
 
 
 
 
 
 
m_Notes 
 
java.lang.String  m_Notes  
 
 User notes about the experiment
 
 
 
 
 
 
m_AdditionalMeasures 
 
java.lang.String[]  m_AdditionalMeasures  
 
 Method names of additional measures of objects contained in the 
      custom property iterator. Only methods names beginning with "measure"
      and returning doubles are recognised
 
 
 
 
 
 
m_ClassFirst 
 
boolean  m_ClassFirst  
 
 True if the class attribute is the first attribute for all
      datasets involved in this experiment.
 
 
 
 
 
 
m_AdvanceDataSetFirst 
 
boolean  m_AdvanceDataSetFirst  
 
 If true an experiment will advance the current data set befor
      any custom itererator
 
 
 
 

 
   
 
 
  
 Class  weka.experiment.InstanceQuery  extends  DatabaseUtils  implements Serializable   
 
 

 
 serialVersionUID:  718158370917782584L

 
   
 
 
  
 Serialized Fields   
 
 

 
m_CreateSparseData 
 
boolean  m_CreateSparseData  
 
 Determines whether sparse data is created
 
 
 
 
 
 
m_Query 
 
java.lang.String  m_Query  
 
 Query to execute
 
 
 
 

 
   
 
 
  
 Class  weka.experiment.InstancesResultListener  extends  CSVResultListener  implements Serializable   
 
 

 
 serialVersionUID:  -2203808461809311178L

 

 
   
 
 
  
 Class  weka.experiment.LearningRateResultProducer  extends java.lang.Object implements Serializable   
 
 

 
 serialVersionUID:  -3841159673490861331L

 
   
 
 
  
 Serialized Fields   
 
 

 
m_Instances 
 
 Instances   m_Instances  
 
 The dataset of interest
 
 
 
 
 
 
m_ResultListener 
 
 ResultListener   m_ResultListener  
 
 The ResultListener to send results to
 
 
 
 
 
 
m_ResultProducer 
 
 ResultProducer   m_ResultProducer  
 
 The ResultProducer used to generate results
 
 
 
 
 
 
m_AdditionalMeasures 
 
java.lang.String[]  m_AdditionalMeasures  
 
 The names of any additional measures to look for in SplitEvaluators
 
 
 
 
 
 
m_LowerSize 
 
int  m_LowerSize  
 
 The minimum number of instances to use. If this is zero, the first
 step will contain m_StepSize instances
 
 
 
 
 
 
m_UpperSize 
 
int  m_UpperSize  
 
 The maximum number of instances to use. -1 indicates no maximum 
 (other than the total number of instances)
 
 
 
 
 
 
m_StepSize 
 
int  m_StepSize  
 
 The number of instances to add at each step
 
 
 
 
 
 
m_CurrentSize 
 
int  m_CurrentSize  
 
 The current dataset size during stepping
 
 
 
 

 
   
 
 
  
 Class  weka.experiment.PairedCorrectedTTester  extends  PairedTTester  implements Serializable   
 
 

 
 serialVersionUID:  -3105268939845653323L

 

 
   
 
 
  
 Class  weka.experiment.PairedTTester  extends java.lang.Object implements Serializable   
 
 

 
 serialVersionUID:  8370014624008728610L

 
   
 
 
  
 Serialized Fields   
 
 

 
m_Instances 
 
 Instances   m_Instances  
 
 The set of instances we will analyse
 
 
 
 
 
 
m_RunColumn 
 
int  m_RunColumn  
 
 The index of the column containing the run number
 
 
 
 
 
 
m_RunColumnSet 
 
int  m_RunColumnSet  
 
 The option setting for the run number column (-1 means last)
 
 
 
 
 
 
m_FoldColumn 
 
int  m_FoldColumn  
 
 The option setting for the fold number column (-1 means none)
 
 
 
 
 
 
m_SortColumn 
 
int  m_SortColumn  
 
 The column to sort on (-1 means default sorting)
 
 
 
 
 
 
m_SortOrder 
 
int[]  m_SortOrder  
 
 The sorting of the datasets (according to the sort column)
 
 
 
 
 
 
m_ColOrder 
 
int[]  m_ColOrder  
 
 The sorting of the columns (test base is always first)
 
 
 
 
 
 
m_SignificanceLevel 
 
double  m_SignificanceLevel  
 
 The significance level for comparisons
 
 
 
 
 
 
m_DatasetKeyColumnsRange 
 
 Range   m_DatasetKeyColumnsRange  
 
 The range of columns that specify a unique "dataset"
 (eg: scheme plus configuration)
 
 
 
 
 
 
m_DatasetKeyColumns 
 
int[]  m_DatasetKeyColumns  
 
 An array containing the indexes of just the selected columns
 
 
 
 
 
 
m_DatasetSpecifiers 
 
weka.experiment.PairedTTester.DatasetSpecifiers  m_DatasetSpecifiers  
 
 The list of dataset specifiers
 
 
 
 
 
 
m_ResultsetKeyColumnsRange 
 
 Range   m_ResultsetKeyColumnsRange  
 
 The range of columns that specify a unique result set
 (eg: scheme plus configuration)
 
 
 
 
 
 
m_ResultsetKeyColumns 
 
int[]  m_ResultsetKeyColumns  
 
 An array containing the indexes of just the selected columns
 
 
 
 
 
 
m_DisplayedResultsets 
 
int[]  m_DisplayedResultsets  
 
 An array containing the indexes of the datasets to display
 
 
 
 
 
 
m_Resultsets 
 
 FastVector   m_Resultsets  
 
 Stores a vector for each resultset holding all instances in each set
 
 
 
 
 
 
m_ResultsetsValid 
 
boolean  m_ResultsetsValid  
 
 Indicates whether the instances have been partitioned
 
 
 
 
 
 
m_ShowStdDevs 
 
boolean  m_ShowStdDevs  
 
 Indicates whether standard deviations should be displayed
 
 
 
 
 
 
m_ResultMatrix 
 
 ResultMatrix   m_ResultMatrix  
 
 the instance of the class to produce the output.
 
 
 
 

 
   
 
 
  
 Class  weka.experiment.PropertyNode  extends java.lang.Object implements Serializable   
 
 

 
 serialVersionUID:  -8718165742572631384L

 
   
 
 
  
 Serialization Methods   
 
 

 
    
readObject 
 
private void  readObject (java.io.ObjectInputStream in)
                 throws java.io.IOException,
                        java.lang.ClassNotFoundException 
 
  

  Throws: 
  java.io.IOException 
  java.lang.ClassNotFoundException  
 
 
 
    
writeObject 
 
private void  writeObject (java.io.ObjectOutputStream out)
                  throws java.io.IOException 
 
  

  Throws: 
  java.io.IOException  
 
 
   
 
 
  
 Serialized Fields   
 
 

 
value 
 
java.lang.Object  value  
 
 The current property value
 
 
 
 
 
 
parentClass 
 
java.lang.Class<T>  parentClass  
 
 The class of the object with this property
 
 
 
 
 
 
property 
 
java.beans.PropertyDescriptor  property  
 
 Other info about the property
 
 
 
 

 
   
 
 
  
 Class  weka.experiment.RandomSplitResultProducer  extends java.lang.Object implements Serializable   
 
 

 
 serialVersionUID:  1403798165056795073L

 
   
 
 
  
 Serialized Fields   
 
 

 
m_Instances 
 
 Instances   m_Instances  
 
 The dataset of interest
 
 
 
 
 
 
m_ResultListener 
 
 ResultListener   m_ResultListener  
 
 The ResultListener to send results to
 
 
 
 
 
 
m_TrainPercent 
 
double  m_TrainPercent  
 
 The percentage of instances to use for training
 
 
 
 
 
 
m_randomize 
 
boolean  m_randomize  
 
 Whether dataset is to be randomized
 
 
 
 
 
 
m_SplitEvaluator 
 
 SplitEvaluator   m_SplitEvaluator  
 
 The SplitEvaluator used to generate results
 
 
 
 
 
 
m_AdditionalMeasures 
 
java.lang.String[]  m_AdditionalMeasures  
 
 The names of any additional measures to look for in SplitEvaluators
 
 
 
 
 
 
m_debugOutput 
 
boolean  m_debugOutput  
 
 Save raw output of split evaluators --- for debugging purposes
 
 
 
 
 
 
m_ZipDest 
 
 OutputZipper   m_ZipDest  
 
 The output zipper to use for saving raw splitEvaluator output
 
 
 
 
 
 
m_OutputFile 
 
java.io.File  m_OutputFile  
 
 The destination output file/directory for raw output
 
 
 
 

 
   
 
 
  
 Class  weka.experiment.RegressionSplitEvaluator  extends java.lang.Object implements Serializable   
 
 

 
 serialVersionUID:  -328181640503349202L

 
   
 
 
  
 Serialized Fields   
 
 

 
m_Template 
 
 Classifier   m_Template  
 
 The template classifier
 
 
 
 
 
 
m_Classifier 
 
 Classifier   m_Classifier  
 
 The classifier used for evaluation
 
 
 
 
 
 
m_AdditionalMeasures 
 
java.lang.String[]  m_AdditionalMeasures  
 
 The names of any additional measures to look for in SplitEvaluators
 
 
 
 
 
 
m_doesProduce 
 
boolean[]  m_doesProduce  
 
 Array of booleans corresponding to the measures in m_AdditionalMeasures
      indicating which of the AdditionalMeasures the current classifier
      can produce
 
 
 
 
 
 
m_result 
 
java.lang.String  m_result  
 
 Holds the statistics for the most recent application of the classifier
 
 
 
 
 
 
m_ClassifierOptions 
 
java.lang.String  m_ClassifierOptions  
 
 The classifier options (if any)
 
 
 
 
 
 
m_ClassifierVersion 
 
java.lang.String  m_ClassifierVersion  
 
 The classifier version
 
 
 
 

 
   
 
 
  
 Class  weka.experiment.RemoteEngine  extends java.rmi.server.UnicastRemoteObject implements Serializable   
 
 

 
 serialVersionUID:  -1021538162895448259L

 
   
 
 
  
 Serialized Fields   
 
 

 
m_HostName 
 
java.lang.String  m_HostName  
 
 The name of the host that this engine is started on
 
 
 
 
 
 
m_TaskQueue 
 
 Queue   m_TaskQueue  
 
 A queue of waiting tasks
 
 
 
 
 
 
m_TaskIdQueue 
 
 Queue   m_TaskIdQueue  
 
 A queue of corresponding ID's for tasks
 
 
 
 
 
 
m_TaskStatus 
 
java.util.Hashtable<K,V>  m_TaskStatus  
 
 A hashtable of experiment status
 
 
 
 
 
 
m_TaskRunning 
 
boolean  m_TaskRunning  
 
 Is there a task running
 
 
 
 

 
   
 
 
  
 Class weka.experiment.RemoteEngine_Stub extends java.rmi.server.RemoteStub implements Serializable   
 
 

 
 serialVersionUID:  2L

 

 
   
 
 
  
 Class  weka.experiment.RemoteExperiment  extends  Experiment  implements Serializable   
 
 

 
 serialVersionUID:  -7357668825635314937L

 
   
 
 
  
 Serialized Fields   
 
 

 
m_listeners 
 
 FastVector   m_listeners  
 
 The list of objects listening for remote experiment events
 
 
 
 
 
 
m_remoteHosts 
 
javax.swing.DefaultListModel  m_remoteHosts  
 
 Holds the names of machines with remoteEngine servers running
 
 
 
 
 
 
m_remoteHostsQueue 
 
 Queue   m_remoteHostsQueue  
 
 The queue of available hosts
 
 
 
 
 
 
m_remoteHostsStatus 
 
int[]  m_remoteHostsStatus  
 
 The status of each of the remote hosts
 
 
 
 
 
 
m_remoteHostFailureCounts 
 
int[]  m_remoteHostFailureCounts  
 
 The number of times tasks have failed on each remote host
 
 
 
 
 
 
m_experimentAborted 
 
boolean  m_experimentAborted  
 
 Set to true if MAX_FAILURES exceeded on all hosts or connections fail 
      on all hosts or user aborts experiment (via gui)
 
 
 
 
 
 
m_removedHosts 
 
int  m_removedHosts  
 
 The number of hosts removed due to exceeding max failures
 
 
 
 
 
 
m_failedCount 
 
int  m_failedCount  
 
 The count of failed sub-experiments
 
 
 
 
 
 
m_finishedCount 
 
int  m_finishedCount  
 
 The count of successfully completed sub-experiments
 
 
 
 
 
 
m_baseExperiment 
 
 Experiment   m_baseExperiment  
 
 The base experiment to split up into sub experiments for remote
      execution
 
 
 
 
 
 
m_subExperiments 
 
 Experiment []  m_subExperiments  
 
 The sub experiments
 
 
 
 
 
 
m_subExpQueue 
 
 Queue   m_subExpQueue  
 
 The queue of sub experiments waiting to be processed
 
 
 
 
 
 
m_subExpComplete 
 
int[]  m_subExpComplete  
 
 The status of each of the sub-experiments
 
 
 
 
 
 
m_splitByDataSet 
 
boolean  m_splitByDataSet  
 
 If true, then sub experiments are created on the basis of data sets
 rather than run number.
 
 
 
 

 
   
 
 
  
 Class  weka.experiment.RemoteExperimentEvent  extends java.lang.Object implements Serializable   
 
 

 
 serialVersionUID:  7000867987391866451L

 
   
 
 
  
 Serialized Fields   
 
 

 
m_statusMessage 
 
boolean  m_statusMessage  
 
 A status type message
 
 
 
 
 
 
m_logMessage 
 
boolean  m_logMessage  
 
 A log type message
 
 
 
 
 
 
m_messageString 
 
java.lang.String  m_messageString  
 
 The message
 
 
 
 
 
 
m_experimentFinished 
 
boolean  m_experimentFinished  
 
 True if a remote experiment has finished
 
 
 
 

 
   
 
 
  
 Class  weka.experiment.RemoteExperimentSubTask  extends java.lang.Object implements Serializable   
 
 

 
   
 
 
  
 Serialized Fields   
 
 

 
m_result 
 
 TaskStatusInfo   m_result  
 
 
 
 
 
 
m_experiment 
 
 Experiment   m_experiment  
 
 
 
 

 
   
 
 
  
 Class  weka.experiment.ResultMatrix  extends java.lang.Object implements Serializable   
 
 

 
 serialVersionUID:  4487179306428209739L

 
   
 
 
  
 Serialized Fields   
 
 

 
TIE_STRING 
 
java.lang.String  TIE_STRING  
 
 tie string
 
 
 
 
 
 
WIN_STRING 
 
java.lang.String  WIN_STRING  
 
 win string
 
 
 
 
 
 
LOSS_STRING 
 
java.lang.String  LOSS_STRING  
 
 loss string
 
 
 
 
 
 
LEFT_PARENTHESES 
 
java.lang.String  LEFT_PARENTHESES  
 
 the left parentheses for enumerating cols/rows
 
 
 
 
 
 
RIGHT_PARENTHESES 
 
java.lang.String  RIGHT_PARENTHESES  
 
 the right parentheses for enumerating cols/rows
 
 
 
 
 
 
m_ColNames 
 
java.lang.String[]  m_ColNames  
 
 the column names
 
 
 
 
 
 
m_RowNames 
 
java.lang.String[]  m_RowNames  
 
 the row names
 
 
 
 
 
 
m_ColHidden 
 
boolean[]  m_ColHidden  
 
 whether a column is hidden
 
 
 
 
 
 
m_RowHidden 
 
boolean[]  m_RowHidden  
 
 whether a row is hidden
 
 
 
 
 
 
m_Significance 
 
int[][]  m_Significance  
 
 the significance
 
 
 
 
 
 
m_Mean 
 
double[][]  m_Mean  
 
 the values
 
 
 
 
 
 
m_StdDev 
 
double[][]  m_StdDev  
 
 the standard deviation
 
 
 
 
 
 
m_Counts 
 
double[]  m_Counts  
 
 the counts for the different datasets
 
 
 
 
 
 
m_MeanPrec 
 
int  m_MeanPrec  
 
 the standard mean precision
 
 
 
 
 
 
m_StdDevPrec 
 
int  m_StdDevPrec  
 
 the standard std. deviation preicision
 
 
 
 
 
 
m_ShowStdDev 
 
boolean  m_ShowStdDev  
 
 whether std. deviations are printed as well
 
 
 
 
 
 
m_ShowAverage 
 
boolean  m_ShowAverage  
 
 whether the average for each column should be printed
 
 
 
 
 
 
m_PrintColNames 
 
boolean  m_PrintColNames  
 
 whether the names or numbers are output as column declarations
 
 
 
 
 
 
m_PrintRowNames 
 
boolean  m_PrintRowNames  
 
 whether the names or numbers are output as row declarations
 
 
 
 
 
 
m_EnumerateColNames 
 
boolean  m_EnumerateColNames  
 
 whether a "(x)" is printed before each column name with "x" as the
 index
 
 
 
 
 
 
m_EnumerateRowNames 
 
boolean  m_EnumerateRowNames  
 
 whether a "(x)" is printed before each row name with "x" as the index
 
 
 
 
 
 
m_ColNameWidth 
 
int  m_ColNameWidth  
 
 the size of the names of the columns
 
 
 
 
 
 
m_RowNameWidth 
 
int  m_RowNameWidth  
 
 the size of the names of the rows
 
 
 
 
 
 
m_MeanWidth 
 
int  m_MeanWidth  
 
 the size of the mean columns
 
 
 
 
 
 
m_StdDevWidth 
 
int  m_StdDevWidth  
 
 the size of the std dev columns
 
 
 
 
 
 
m_SignificanceWidth 
 
int  m_SignificanceWidth  
 
 the size of the significance columns
 
 
 
 
 
 
m_CountWidth 
 
int  m_CountWidth  
 
 the size of the counts
 
 
 
 
 
 
m_HeaderKeys 
 
java.util.Vector<E>  m_HeaderKeys  
 
 contains the keys for the header
 
 
 
 
 
 
m_HeaderValues 
 
java.util.Vector<E>  m_HeaderValues  
 
 contains the values for the header
 
 
 
 
 
 
m_NonSigWins 
 
int[][]  m_NonSigWins  
 
 the non-significant wins
 
 
 
 
 
 
m_Wins 
 
int[][]  m_Wins  
 
 the significant wins
 
 
 
 
 
 
m_RankingWins 
 
int[]  m_RankingWins  
 
 the wins in ranking
 
 
 
 
 
 
m_RankingLosses 
 
int[]  m_RankingLosses  
 
 the losses in ranking
 
 
 
 
 
 
m_RankingDiff 
 
int[]  m_RankingDiff  
 
 the difference between wins and losses
 
 
 
 
 
 
m_RowOrder 
 
int[]  m_RowOrder  
 
 the ordering of the rows
 
 
 
 
 
 
m_ColOrder 
 
int[]  m_ColOrder  
 
 the ordering of the columns
 
 
 
 
 
 
m_RemoveFilterName 
 
boolean  m_RemoveFilterName  
 
 whether to remove the filter name from the dataaset name
 
 
 
 

 
   
 
 
  
 Class  weka.experiment.ResultMatrixCSV  extends  ResultMatrix  implements Serializable   
 
 

 
 serialVersionUID:  -171838863135042743L

 

 
   
 
 
  
 Class  weka.experiment.ResultMatrixGnuPlot  extends  ResultMatrix  implements Serializable   
 
 

 
 serialVersionUID:  -234648254944790097L

 

 
   
 
 
  
 Class  weka.experiment.ResultMatrixHTML  extends  ResultMatrix  implements Serializable   
 
 

 
 serialVersionUID:  6672380422544799990L

 

 
   
 
 
  
 Class  weka.experiment.ResultMatrixLatex  extends  ResultMatrix  implements Serializable   
 
 

 
 serialVersionUID:  777690788447600978L

 

 
   
 
 
  
 Class  weka.experiment.ResultMatrixPlainText  extends  ResultMatrix  implements Serializable   
 
 

 
 serialVersionUID:  1502934525382357937L

 

 
   
 
 
  
 Class  weka.experiment.ResultMatrixSignificance  extends  ResultMatrix  implements Serializable   
 
 

 
 serialVersionUID:  -1280545644109764206L

 

 
   
 
 
  
 Class  weka.experiment.Stats  extends java.lang.Object implements Serializable   
 
 

 
 serialVersionUID:  -8610544539090024102L

 
   
 
 
  
 Serialized Fields   
 
 

 
count 
 
double  count  
 
 The number of values seen
 
 
 
 
 
 
sum 
 
double  sum  
 
 The sum of values seen
 
 
 
 
 
 
sumSq 
 
double  sumSq  
 
 The sum of values squared seen
 
 
 
 
 
 
stdDev 
 
double  stdDev  
 
 The std deviation of values at the last calculateDerived() call
 
 
 
 
 
 
mean 
 
double  mean  
 
 The mean of values at the last calculateDerived() call
 
 
 
 
 
 
min 
 
double  min  
 
 The minimum value seen, or Double.NaN if no values seen
 
 
 
 
 
 
max 
 
double  max  
 
 The maximum value seen, or Double.NaN if no values seen
 
 
 
 

 
   
 
 
  
 Class  weka.experiment.TaskStatusInfo  extends java.lang.Object implements Serializable   
 
 

 
 serialVersionUID:  -6129343303703560015L

 
   
 
 
  
 Serialized Fields   
 
 

 
m_ExecutionStatus 
 
int  m_ExecutionStatus  
 
 Holds current execution status.
 
 
 
 
 
 
m_StatusMessage 
 
java.lang.String  m_StatusMessage  
 
 Holds current status message.
 
 
 
 
 
 
m_TaskResult 
 
java.lang.Object  m_TaskResult  
 
 Holds task result. Set to null for no returnable result.
 
 
 
 
 

 
 
  
 Package   weka.filters   
 
 

 
   
 
 
  
 Class  weka.filters.AllFilter  extends  Filter  implements Serializable   
 
 

 
 serialVersionUID:  5022109283147503266L

 

 
   
 
 
  
 Class  weka.filters.Filter  extends java.lang.Object implements Serializable   
 
 

 
 serialVersionUID:  -8835063755891851218L

 
   
 
 
  
 Serialized Fields   
 
 

 
m_OutputFormat 
 
 Instances   m_OutputFormat  
 
 The output format for instances
 
 
 
 
 
 
m_OutputQueue 
 
 Queue   m_OutputQueue  
 
 The output instance queue
 
 
 
 
 
 
m_OutputStringAtts 
 
 StringLocator   m_OutputStringAtts  
 
 Indices of string attributes in the output format
 
 
 
 
 
 
m_InputStringAtts 
 
 StringLocator   m_InputStringAtts  
 
 Indices of string attributes in the input format
 
 
 
 
 
 
m_OutputRelAtts 
 
 RelationalLocator   m_OutputRelAtts  
 
 Indices of relational attributes in the output format
 
 
 
 
 
 
m_InputRelAtts 
 
 RelationalLocator   m_InputRelAtts  
 
 Indices of relational attributes in the input format
 
 
 
 
 
 
m_InputFormat 
 
 Instances   m_InputFormat  
 
 The input format for instances
 
 
 
 
 
 
m_NewBatch 
 
boolean  m_NewBatch  
 
 Record whether the filter is at the start of a batch
 
 
 
 
 
 
m_FirstBatchDone 
 
boolean  m_FirstBatchDone  
 
 True if the first batch has been done
 
 
 
 

 
   
 
 
  
 Class  weka.filters.MultiFilter  extends  SimpleStreamFilter  implements Serializable   
 
 

 
 serialVersionUID:  -6293720886005713120L

 
   
 
 
  
 Serialized Fields   
 
 

 
m_Filters 
 
 Filter []  m_Filters  
 
 The filters
 
 
 
 
 
 
m_Streamable 
 
boolean  m_Streamable  
 
 caches the streamable state
 
 
 
 
 
 
m_StreamableChecked 
 
boolean  m_StreamableChecked  
 
 whether we already checked the streamable state
 
 
 
 

 
   
 
 
  
 Class  weka.filters.SimpleBatchFilter  extends  SimpleFilter  implements Serializable   
 
 

 
 serialVersionUID:  8102908673378055114L

 

 
   
 
 
  
 Class  weka.filters.SimpleFilter  extends  Filter  implements Serializable   
 
 

 
 serialVersionUID:  5702974949137433141L

 
   
 
 
  
 Serialized Fields   
 
 

 
m_Debug 
 
boolean  m_Debug  
 
 Whether debugging is on
 
 
 
 

 
   
 
 
  
 Class  weka.filters.SimpleStreamFilter  extends  SimpleFilter  implements Serializable   
 
 

 
 serialVersionUID:  2754882676192747091L

 
 

 
 
  
 Package   weka.filters.supervised.attribute   
 
 

 
   
 
 
  
 Class  weka.filters.supervised.attribute.AddClassification  extends  SimpleBatchFilter  implements Serializable   
 
 

 
 serialVersionUID:  -1931467132568441909L

 
   
 
 
  
 Serialized Fields   
 
 

 
m_Classifier 
 
 Classifier   m_Classifier  
 
 The classifier template used to do the classification.
 
 
 
 
 
 
m_SerializedClassifierFile 
 
java.io.File  m_SerializedClassifierFile  
 
 The file from which to load a serialized classifier.
 
 
 
 
 
 
m_ActualClassifier 
 
 Classifier   m_ActualClassifier  
 
 The actual classifier used to do the classification.
 
 
 
 
 
 
m_SerializedHeader 
 
 Instances   m_SerializedHeader  
 
 the header of the file the serialized classifier was trained with.
 
 
 
 
 
 
m_OutputClassification 
 
boolean  m_OutputClassification  
 
 whether to output the classification.
 
 
 
 
 
 
m_RemoveOldClass 
 
boolean  m_RemoveOldClass  
 
 whether to remove the old class attribute.
 
 
 
 
 
 
m_OutputDistribution 
 
boolean  m_OutputDistribution  
 
 whether to output the class distribution.
 
 
 
 
 
 
m_OutputErrorFlag 
 
boolean  m_OutputErrorFlag  
 
 whether to output the error flag.
 
 
 
 

 
   
 
 
  
 Class  weka.filters.supervised.attribute.AttributeSelection  extends  Filter  implements Serializable   
 
 

 
 serialVersionUID:  -296211247688169716L

 
   
 
 
  
 Serialized Fields   
 
 

 
m_trainSelector 
 
 AttributeSelection   m_trainSelector  
 
 the attribute selection evaluation object
 
 
 
 
 
 
m_ASEvaluator 
 
 ASEvaluation   m_ASEvaluator  
 
 the attribute evaluator to use
 
 
 
 
 
 
m_ASSearch 
 
 ASSearch   m_ASSearch  
 
 the search method if any
 
 
 
 
 
 
m_FilterOptions 
 
java.lang.String[]  m_FilterOptions  
 
 holds a copy of the full set of valid  options passed to the filter
 
 
 
 
 
 
m_SelectedAttributes 
 
int[]  m_SelectedAttributes  
 
 holds the selected attributes
 
 
 
 

 
   
 
 
  
 Class  weka.filters.supervised.attribute.ClassOrder  extends  Filter  implements Serializable   
 
 

 
 serialVersionUID:  -2116226838887628411L

 
   
 
 
  
 Serialized Fields   
 
 

 
m_Seed 
 
long  m_Seed  
 
 The seed of randomization
 
 
 
 
 
 
m_Random 
 
java.util.Random  m_Random  
 
 The random object
 
 
 
 
 
 
m_Converter 
 
int[]  m_Converter  
 
 The 1-1 converting table from the original class values
 to the new values
 
 
 
 
 
 
m_ClassAttribute 
 
 Attribute   m_ClassAttribute  
 
 Class attribute of the data
 
 
 
 
 
 
m_ClassOrder 
 
int  m_ClassOrder  
 
 The class order to be sorted
 
 
 
 
 
 
m_ClassCounts 
 
double[]  m_ClassCounts  
 
 This class can provide the class distribution in the sorted order 
  as side effect
 
 
 
 

 
   
 
 
  
 Class  weka.filters.supervised.attribute.Discretize  extends  Filter  implements Serializable   
 
 

 
 serialVersionUID:  -3141006402280129097L

 
   
 
 
  
 Serialized Fields   
 
 

 
m_DiscretizeCols 
 
 Range   m_DiscretizeCols  
 
 Stores which columns to Discretize
 
 
 
 
 
 
m_CutPoints 
 
double[][]  m_CutPoints  
 
 Store the current cutpoints
 
 
 
 
 
 
m_MakeBinary 
 
boolean  m_MakeBinary  
 
 Output binary attributes for discretized attributes.
 
 
 
 
 
 
m_UseBetterEncoding 
 
boolean  m_UseBetterEncoding  
 
 Use better encoding of split point for MDL.
 
 
 
 
 
 
m_UseKononenko 
 
boolean  m_UseKononenko  
 
 Use Kononenko's MDL criterion instead of Fayyad et al.'s
 
 
 
 

 
   
 
 
  
 Class  weka.filters.supervised.attribute.NominalToBinary  extends  Filter  implements Serializable   
 
 

 
 serialVersionUID:  -5004607029857673950L

 
   
 
 
  
 Serialized Fields   
 
 

 
m_Indices 
 
int[][]  m_Indices  
 
 The sorted indices of the attribute values.
 
 
 
 
 
 
m_Numeric 
 
boolean  m_Numeric  
 
 Are the new attributes going to be nominal or numeric ones?
 
 
 
 
 
 
m_TransformAll 
 
boolean  m_TransformAll  
 
 Are all values transformed into new attributes?
 
 
 
 

 
   
 
 
  
 Class  weka.filters.supervised.attribute.PLSFilter  extends  SimpleBatchFilter  implements Serializable   
 
 

 
 serialVersionUID:  -3335106965521265631L

 
   
 
 
  
 Serialized Fields   
 
 

 
m_NumComponents 
 
int  m_NumComponents  
 
 the maximum number of components to generate
 
 
 
 
 
 
m_Algorithm 
 
int  m_Algorithm  
 
 the type of algorithm
 
 
 
 
 
 
m_PLS1_RegVector 
 
 Matrix   m_PLS1_RegVector  
 
 the regression vector "r-hat" for PLS1
 
 
 
 
 
 
m_PLS1_P 
 
 Matrix   m_PLS1_P  
 
 the P matrix for PLS1
 
 
 
 
 
 
m_PLS1_W 
 
 Matrix   m_PLS1_W  
 
 the W matrix for PLS1
 
 
 
 
 
 
m_PLS1_b_hat 
 
 Matrix   m_PLS1_b_hat  
 
 the b-hat vector for PLS1
 
 
 
 
 
 
m_SIMPLS_W 
 
 Matrix   m_SIMPLS_W  
 
 the W matrix for SIMPLS
 
 
 
 
 
 
m_SIMPLS_B 
 
 Matrix   m_SIMPLS_B  
 
 the B matrix for SIMPLS (used for prediction)
 
 
 
 
 
 
m_PerformPrediction 
 
boolean  m_PerformPrediction  
 
 whether to include the prediction, i.e., modifying the class attribute
 
 
 
 
 
 
m_Missing 
 
 Filter   m_Missing  
 
 for replacing missing values
 
 
 
 
 
 
m_ReplaceMissing 
 
boolean  m_ReplaceMissing  
 
 whether to replace missing values
 
 
 
 
 
 
m_Filter 
 
 Filter   m_Filter  
 
 for centering the data
 
 
 
 
 
 
m_Preprocessing 
 
int  m_Preprocessing  
 
 the type of preprocessing
 
 
 
 
 
 
m_ClassMean 
 
double  m_ClassMean  
 
 the mean of the class
 
 
 
 
 
 
m_ClassStdDev 
 
double  m_ClassStdDev  
 
 the standard deviation of the class
 
 
 
 
 

 
 
  
 Package   weka.filters.supervised.instance   
 
 

 
   
 
 
  
 Class  weka.filters.supervised.instance.Resample  extends  Filter  implements Serializable   
 
 

 
 serialVersionUID:  7079064953548300681L

 
   
 
 
  
 Serialized Fields   
 
 

 
m_SampleSizePercent 
 
double  m_SampleSizePercent  
 
 The subsample size, percent of original set, default 100%.
 
 
 
 
 
 
m_RandomSeed 
 
int  m_RandomSeed  
 
 The random number generator seed.
 
 
 
 
 
 
m_BiasToUniformClass 
 
double  m_BiasToUniformClass  
 
 The degree of bias towards uniform (nominal) class distribution.
 
 
 
 
 
 
m_NoReplacement 
 
boolean  m_NoReplacement  
 
 Whether to perform sampling with replacement or without.
 
 
 
 
 
 
m_InvertSelection 
 
boolean  m_InvertSelection  
 
 Whether to invert the selection (only if instances are drawn WITHOUT 
 replacement).
 
 
  See Also:    Resample.m_NoReplacement   
 

 
   
 
 
  
 Class  weka.filters.supervised.instance.SMOTE  extends  Filter  implements Serializable   
 
 

 
 serialVersionUID:  -1653880819059250364L

 
   
 
 
  
 Serialized Fields   
 
 

 
m_NearestNeighbors 
 
int  m_NearestNeighbors  
 
 the number of neighbors to use.
 
 
 
 
 
 
m_RandomSeed 
 
int  m_RandomSeed  
 
 the random seed to use.
 
 
 
 
 
 
m_Percentage 
 
double  m_Percentage  
 
 the percentage of SMOTE instances to create.
 
 
 
 
 
 
m_ClassValueIndex 
 
java.lang.String  m_ClassValueIndex  
 
 the index of the class value.
 
 
 
 
 
 
m_DetectMinorityClass 
 
boolean  m_DetectMinorityClass  
 
 whether to detect the minority class automatically.
 
 
 
 

 
   
 
 
  
 Class  weka.filters.supervised.instance.SpreadSubsample  extends  Filter  implements Serializable   
 
 

 
 serialVersionUID:  -3947033795243930016L

 
   
 
 
  
 Serialized Fields   
 
 

 
m_RandomSeed 
 
int  m_RandomSeed  
 
 The random number generator seed
 
 
 
 
 
 
m_MaxCount 
 
int  m_MaxCount  
 
 The maximum count of any class
 
 
 
 
 
 
m_DistributionSpread 
 
double  m_DistributionSpread  
 
 True if the first batch has been done
 
 
 
 
 
 
m_AdjustWeights 
 
boolean  m_AdjustWeights  
 
 True if instance weights will be adjusted to maintain
 total weight per class.
 
 
 
 

 
   
 
 
  
 Class  weka.filters.supervised.instance.StratifiedRemoveFolds  extends  Filter  implements Serializable   
 
 

 
 serialVersionUID:  -7069148179905814324L

 
   
 
 
  
 Serialized Fields   
 
 

 
m_Inverse 
 
boolean  m_Inverse  
 
 Indicates if inverse of selection is to be output.
 
 
 
 
 
 
m_NumFolds 
 
int  m_NumFolds  
 
 Number of folds to split dataset into
 
 
 
 
 
 
m_Fold 
 
int  m_Fold  
 
 Fold to output
 
 
 
 
 
 
m_Seed 
 
long  m_Seed  
 
 Random number seed.
 
 
 
 
 

 
 
  
 Package   weka.filters.unsupervised.attribute   
 
 

 
   
 
 
  
 Class  weka.filters.unsupervised.attribute.AbstractTimeSeries  extends  Filter  implements Serializable   
 
 

 
 serialVersionUID:  -3795656792078022357L

 
   
 
 
  
 Serialized Fields   
 
 

 
m_SelectedCols 
 
 Range   m_SelectedCols  
 
 Stores which columns to copy
 
 
 
 
 
 
m_FillWithMissing 
 
boolean  m_FillWithMissing  
 
 True if missing values should be used rather than removing instances
 where the translated value is not known (due to border effects).
 
 
 
 
 
 
m_InstanceRange 
 
int  m_InstanceRange  
 
 The number of instances forward to translate values between.
 A negative number indicates taking values from a past instance.
 
 
 
 
 
 
m_History 
 
 Queue   m_History  
 
 Stores the historical instances to copy values between
 
 
 
 

 
   
 
 
  
 Class  weka.filters.unsupervised.attribute.Add  extends  Filter  implements Serializable   
 
 

 
 serialVersionUID:  761386447332932389L

 
   
 
 
  
 Serialized Fields   
 
 

 
m_AttributeType 
 
int  m_AttributeType  
 
 Record the type of attribute to insert.
 
 
 
 
 
 
m_Name 
 
java.lang.String  m_Name  
 
 The name for the new attribute.
 
 
 
 
 
 
m_Insert 
 
 SingleIndex   m_Insert  
 
 The location to insert the new attribute.
 
 
 
 
 
 
m_Labels 
 
 FastVector   m_Labels  
 
 The list of labels for nominal attribute.
 
 
 
 
 
 
m_DateFormat 
 
java.lang.String  m_DateFormat  
 
 The date format.
 
 
 
 

 
   
 
 
  
 Class  weka.filters.unsupervised.attribute.AddCluster  extends  Filter  implements Serializable   
 
 

 
 serialVersionUID:  7414280611943807337L

 
   
 
 
  
 Serialized Fields   
 
 

 
m_Clusterer 
 
 Clusterer   m_Clusterer  
 
 The clusterer used to do the cleansing
 
 
 
 
 
 
m_IgnoreAttributesRange 
 
 Range   m_IgnoreAttributesRange  
 
 Range of attributes to ignore
 
 
 
 
 
 
m_removeAttributes 
 
 Filter   m_removeAttributes  
 
 Filter for removing attributes
 
 
 
 

 
   
 
 
  
 Class  weka.filters.unsupervised.attribute.AddExpression  extends  Filter  implements Serializable   
 
 

 
 serialVersionUID:  402130384261736245L

 
   
 
 
  
 Serialized Fields   
 
 

 
m_infixExpression 
 
java.lang.String  m_infixExpression  
 
 The infix expression
 
 
 
 
 
 
m_attributeName 
 
java.lang.String  m_attributeName  
 
 Name of the new attribute. "expression"  length string will use the 
      provided expression as the new attribute name
 
 
 
 
 
 
m_Debug 
 
boolean  m_Debug  
 
 If true, makes the attribute name equal to the postfix parse of the
      expression
 
 
 
 
 
 
m_attributeExpression 
 
 AttributeExpression   m_attributeExpression  
 
 
 
 

 
   
 
 
  
 Class  weka.filters.unsupervised.attribute.AddID  extends  Filter  implements Serializable   
 
 

 
 serialVersionUID:  4734383199819293390L

 
   
 
 
  
 Serialized Fields   
 
 

 
m_Index 
 
 SingleIndex   m_Index  
 
 the index of the attribute
 
 
 
 
 
 
m_Name 
 
java.lang.String  m_Name  
 
 the name of the attribute
 
 
 
 
 
 
m_Counter 
 
int  m_Counter  
 
 the counter for the ID
 
 
 
 

 
   
 
 
  
 Class  weka.filters.unsupervised.attribute.AddNoise  extends  Filter  implements Serializable   
 
 

 
 serialVersionUID:  -8499673222857299082L

 
   
 
 
  
 Serialized Fields   
 
 

 
m_AttIndex 
 
 SingleIndex   m_AttIndex  
 
 The attribute's index setting.
 
 
 
 
 
 
m_UseMissing 
 
boolean  m_UseMissing  
 
 Flag if missing values are taken as value.
 
 
 
 
 
 
m_Percent 
 
int  m_Percent  
 
 The subsample size, percent of original set, default 10%
 
 
 
 
 
 
m_RandomSeed 
 
int  m_RandomSeed  
 
 The random number generator seed
 
 
 
 

 
   
 
 
  
 Class  weka.filters.unsupervised.attribute.AddValues  extends  Filter  implements Serializable   
 
 

 
 serialVersionUID:  -8100622241742393656L

 
   
 
 
  
 Serialized Fields   
 
 

 
m_AttIndex 
 
 SingleIndex   m_AttIndex  
 
 The attribute's index setting.
 
 
 
 
 
 
m_Labels 
 
java.util.Vector<E>  m_Labels  
 
 The values to add.
 
 
 
 
 
 
m_Sort 
 
boolean  m_Sort  
 
 Whether to sort the values.
 
 
 
 
 
 
m_SortedIndices 
 
int[]  m_SortedIndices  
 
 the array with the sorted label indices
 
 
 
 

 
   
 
 
  
 Class  weka.filters.unsupervised.attribute.Center  extends  PotentialClassIgnorer  implements Serializable   
 
 

 
 serialVersionUID:  -9101338448900581023L

 
   
 
 
  
 Serialized Fields   
 
 

 
m_Means 
 
double[]  m_Means  
 
 The means
 
 
 
 

 
   
 
 
  
 Class  weka.filters.unsupervised.attribute.ChangeDateFormat  extends  Filter  implements Serializable   
 
 

 
 serialVersionUID:  -1609344074013448737L

 
   
 
 
  
 Serialized Fields   
 
 

 
m_AttIndex 
 
 SingleIndex   m_AttIndex  
 
 The attribute's index setting.
 
 
 
 
 
 
m_DateFormat 
 
java.text.SimpleDateFormat  m_DateFormat  
 
 The output date format.
 
 
 
 
 
 
m_OutputAttribute 
 
 Attribute   m_OutputAttribute  
 
 The output attribute.
 
 
 
 

 
   
 
 
  
 Class  weka.filters.unsupervised.attribute.ClassAssigner  extends  SimpleStreamFilter  implements Serializable   
 
 

 
 serialVersionUID:  1775780193887394115L

 
   
 
 
  
 Serialized Fields   
 
 

 
m_ClassIndex 
 
int  m_ClassIndex  
 
 the class index.
 
 
 
 

 
   
 
 
  
 Class  weka.filters.unsupervised.attribute.ClusterMembership  extends  Filter  implements Serializable   
 
 

 
 serialVersionUID:  6675702504667714026L

 
   
 
 
  
 Serialized Fields   
 
 

 
m_clusterer 
 
 DensityBasedClusterer   m_clusterer  
 
 The clusterer
 
 
 
 
 
 
m_clusterers 
 
 DensityBasedClusterer []  m_clusterers  
 
 Array for storing the clusterers
 
 
 
 
 
 
m_ignoreAttributesRange 
 
 Range   m_ignoreAttributesRange  
 
 Range of attributes to ignore
 
 
 
 
 
 
m_removeAttributes 
 
 Filter   m_removeAttributes  
 
 Filter for removing attributes
 
 
 
 
 
 
m_priors 
 
double[]  m_priors  
 
 The prior probability for each class
 
 
 
 

 
   
 
 
  
 Class  weka.filters.unsupervised.attribute.Copy  extends  Filter  implements Serializable   
 
 

 
 serialVersionUID:  -8543707493627441566L

 
   
 
 
  
 Serialized Fields   
 
 

 
m_CopyCols 
 
 Range   m_CopyCols  
 
 Stores which columns to copy
 
 
 
 
 
 
m_SelectedAttributes 
 
int[]  m_SelectedAttributes  
 
 Stores the indexes of the selected attributes in order, once the
 dataset is seen
 
 
 
 

 
   
 
 
  
 Class  weka.filters.unsupervised.attribute.Discretize  extends  PotentialClassIgnorer  implements Serializable   
 
 

 
 serialVersionUID:  -1358531742174527279L

 
   
 
 
  
 Serialized Fields   
 
 

 
m_DiscretizeCols 
 
 Range   m_DiscretizeCols  
 
 Stores which columns to Discretize
 
 
 
 
 
 
m_NumBins 
 
int  m_NumBins  
 
 The number of bins to divide the attribute into
 
 
 
 
 
 
m_DesiredWeightOfInstancesPerInterval 
 
double  m_DesiredWeightOfInstancesPerInterval  
 
 The desired weight of instances per bin
 
 
 
 
 
 
m_CutPoints 
 
double[][]  m_CutPoints  
 
 Store the current cutpoints
 
 
 
 
 
 
m_MakeBinary 
 
boolean  m_MakeBinary  
 
 Output binary attributes for discretized attributes.
 
 
 
 
 
 
m_FindNumBins 
 
boolean  m_FindNumBins  
 
 Find the number of bins using cross-validated entropy.
 
 
 
 
 
 
m_UseEqualFrequency 
 
boolean  m_UseEqualFrequency  
 
 Use equal-frequency binning if unsupervised discretization turned on
 
 
 
 
 
 
m_DefaultCols 
 
java.lang.String  m_DefaultCols  
 
 The default columns to discretize
 
 
 
 

 
   
 
 
  
 Class  weka.filters.unsupervised.attribute.FirstOrder  extends  Filter  implements Serializable   
 
 

 
 serialVersionUID:  -7500464545400454179L

 
   
 
 
  
 Serialized Fields   
 
 

 
m_DeltaCols 
 
 Range   m_DeltaCols  
 
 Stores which columns to take differences between
 
 
 
 

 
   
 
 
  
 Class  weka.filters.unsupervised.attribute.InterquartileRange  extends  SimpleBatchFilter  implements Serializable   
 
 

 
 serialVersionUID:  -227879653639723030L

 
   
 
 
  
 Serialized Fields   
 
 

 
m_Attributes 
 
 Range   m_Attributes  
 
 the attribute range to work on
 
 
 
 
 
 
m_AttributeIndices 
 
int[]  m_AttributeIndices  
 
 the generated indices (only for performance reasons)
 
 
 
 
 
 
m_OutlierFactor 
 
double  m_OutlierFactor  
 
 the factor for detecting outliers
 
 
 
 
 
 
m_ExtremeValuesFactor 
 
double  m_ExtremeValuesFactor  
 
 the factor for detecting extreme values, by default 2*m_OutlierFactor
 
 
 
 
 
 
m_ExtremeValuesAsOutliers 
 
boolean  m_ExtremeValuesAsOutliers  
 
 whether extreme values are also tagged as outliers
 
 
 
 
 
 
m_UpperExtremeValue 
 
double[]  m_UpperExtremeValue  
 
 the upper extreme value threshold (= Q3 + EVF*IQR)
 
 
 
 
 
 
m_UpperOutlier 
 
double[]  m_UpperOutlier  
 
 the upper outlier threshold (= Q3 + OF*IQR)
 
 
 
 
 
 
m_LowerOutlier 
 
double[]  m_LowerOutlier  
 
 the lower outlier threshold (= Q1 - OF*IQR)
 
 
 
 
 
 
m_IQR 
 
double[]  m_IQR  
 
 the interquartile range
 
 
 
 
 
 
m_Median 
 
double[]  m_Median  
 
 the median
 
 
 
 
 
 
m_LowerExtremeValue 
 
double[]  m_LowerExtremeValue  
 
 the lower extreme value threshold (= Q1 - EVF*IQR)
 
 
 
 
 
 
m_DetectionPerAttribute 
 
boolean  m_DetectionPerAttribute  
 
 whether to generate Outlier/ExtremeValue attributes for each attribute
 instead of a general one
 
 
 
 
 
 
m_OutlierAttributePosition 
 
int[]  m_OutlierAttributePosition  
 
 the position of the outlier attribute
 
 
 
 
 
 
m_OutputOffsetMultiplier 
 
boolean  m_OutputOffsetMultiplier  
 
 whether to add another attribute called "Offset", that lists the 
 'multiplier' by which the outlier/extreme value is away from the median,
 i.e., value = median + 'multiplier' * IQR  
 automatically enables m_DetectionPerAttribute!
 
 
 
 

 
   
 
 
  
 Class  weka.filters.unsupervised.attribute.KernelFilter  extends  SimpleBatchFilter  implements Serializable   
 
 

 
 serialVersionUID:  213800899640387499L

 
   
 
 
  
 Serialized Fields   
 
 

 
m_NumTrainInstances 
 
int  m_NumTrainInstances  
 
 The number of instances in the training data.
 
 
 
 
 
 
m_Kernel 
 
 Kernel   m_Kernel  
 
 Kernel to use
 
 
 
 
 
 
m_ActualKernel 
 
 Kernel   m_ActualKernel  
 
 the Kernel which is actually used for computation
 
 
 
 
 
 
m_checksTurnedOff 
 
boolean  m_checksTurnedOff  
 
 Turn off all checks and conversions? Turning them off assumes
      that data is purely numeric, doesn't contain any missing values,
      and has a nominal class. Turning them off also means that
      no header information will be stored if the machine is linear. 
      Finally, it also assumes that no instance has a weight equal to 0.
 
 
 
 
 
 
m_NominalToBinary 
 
 NominalToBinary   m_NominalToBinary  
 
 The filter used to make attributes numeric.
 
 
 
 
 
 
m_Missing 
 
 ReplaceMissingValues   m_Missing  
 
 The filter used to get rid of missing values.
 
 
 
 
 
 
m_InitFile 
 
java.io.File  m_InitFile  
 
 The dataset to initialize the filter with
 
 
 
 
 
 
m_InitFileClassIndex 
 
 SingleIndex   m_InitFileClassIndex  
 
 the class index for the file to initialized with
 
 
  See Also:    KernelFilter.m_InitFile   
 
 
 
m_Initialized 
 
boolean  m_Initialized  
 
 whether the filter was initialized
 
 
 
 
 
 
m_KernelFactorExpression 
 
java.lang.String  m_KernelFactorExpression  
 
 optimizes the kernel with this formula 
 (A = # of attributes, N = # of instances)
 
 
 
 
 
 
m_KernelFactor 
 
double  m_KernelFactor  
 
 the calculated kernel factor
 
 
  See Also:    KernelFilter.m_KernelFactorExpression   
 
 
 
m_Filter 
 
 Filter   m_Filter  
 
 for centering/standardizing the data
 
 
 
 
 
 
m_ActualFilter 
 
 Filter   m_ActualFilter  
 
 for centering/standardizing the data (the actual filter to use)
 
 
 
 

 
   
 
 
  
 Class  weka.filters.unsupervised.attribute.MakeIndicator  extends  Filter  implements Serializable   
 
 

 
 serialVersionUID:  766001176862773163L

 
   
 
 
  
 Serialized Fields   
 
 

 
m_AttIndex 
 
 SingleIndex   m_AttIndex  
 
 The attribute's index setting.
 
 
 
 
 
 
m_ValIndex 
 
 Range   m_ValIndex  
 
 The value's index
 
 
 
 
 
 
m_Numeric 
 
boolean  m_Numeric  
 
 Make boolean attribute numeric.
 
 
 
 

 
   
 
 
  
 Class  weka.filters.unsupervised.attribute.MathExpression  extends  PotentialClassIgnorer  implements Serializable   
 
 

 
 serialVersionUID:  -3713222714671997901L

 
   
 
 
  
 Serialized Fields   
 
 

 
m_SelectCols 
 
 Range   m_SelectCols  
 
 Stores which columns to select as a funky range
 
 
 
 
 
 
m_expression 
 
java.lang.String  m_expression  
 
 The modification expression
 
 
 
 
 
 
m_attStats 
 
 AttributeStats []  m_attStats  
 
 Attributes statistics
 
 
 
 

 
   
 
 
  
 Class  weka.filters.unsupervised.attribute.MergeTwoValues  extends  Filter  implements Serializable   
 
 

 
 serialVersionUID:  2925048980504034018L

 
   
 
 
  
 Serialized Fields   
 
 

 
m_AttIndex 
 
 SingleIndex   m_AttIndex  
 
 The attribute's index setting.
 
 
 
 
 
 
m_FirstIndex 
 
 SingleIndex   m_FirstIndex  
 
 The first value's index setting.
 
 
 
 
 
 
m_SecondIndex 
 
 SingleIndex   m_SecondIndex  
 
 The second value's index setting.
 
 
 
 

 
   
 
 
  
 Class  weka.filters.unsupervised.attribute.MultiInstanceToPropositional  extends  Filter  implements Serializable   
 
 

 
 serialVersionUID:  -4102847628883002530L

 
   
 
 
  
 Serialized Fields   
 
 

 
m_NumBags 
 
int  m_NumBags  
 
 the total number of bags
 
 
 
 
 
 
m_BagStringAtts 
 
 StringLocator   m_BagStringAtts  
 
 Indices of string attributes in the bag
 
 
 
 
 
 
m_BagRelAtts 
 
 RelationalLocator   m_BagRelAtts  
 
 Indices of relational attributes in the bag
 
 
 
 
 
 
m_NumInstances 
 
int  m_NumInstances  
 
 the total number of the propositional instance in the dataset
 
 
 
 
 
 
m_WeightMethod 
 
int  m_WeightMethod  
 
 the propositional instance weight setting method
 
 
 
 

 
   
 
 
  
 Class  weka.filters.unsupervised.attribute.NominalToBinary  extends  Filter  implements Serializable   
 
 

 
 serialVersionUID:  -1130642825710549138L

 
   
 
 
  
 Serialized Fields   
 
 

 
m_Columns 
 
 Range   m_Columns  
 
 Stores which columns to act on
 
 
 
 
 
 
m_Numeric 
 
boolean  m_Numeric  
 
 Are the new attributes going to be nominal or numeric ones?
 
 
 
 
 
 
m_TransformAll 
 
boolean  m_TransformAll  
 
 Are all values transformed into new attributes?
 
 
 
 

 
   
 
 
  
 Class  weka.filters.unsupervised.attribute.NominalToString  extends  Filter  implements Serializable   
 
 

 
 serialVersionUID:  8655492378380068939L

 
   
 
 
  
 Serialized Fields   
 
 

 
m_AttIndex 
 
 Range   m_AttIndex  
 
 The attribute's index setting.
 
 
 
 

 
   
 
 
  
 Class  weka.filters.unsupervised.attribute.Normalize  extends  PotentialClassIgnorer  implements Serializable   
 
 

 
 serialVersionUID:  -8158531150984362898L

 
   
 
 
  
 Serialized Fields   
 
 

 
m_MinArray 
 
double[]  m_MinArray  
 
 The minimum values for numeric attributes.
 
 
 
 
 
 
m_MaxArray 
 
double[]  m_MaxArray  
 
 The maximum values for numeric attributes.
 
 
 
 
 
 
m_Translation 
 
double  m_Translation  
 
 The translation of the output range.
 
 
 
 
 
 
m_Scale 
 
double  m_Scale  
 
 The scaling factor of the output range.
 
 
 
 

 
   
 
 
  
 Class  weka.filters.unsupervised.attribute.NumericCleaner  extends  SimpleStreamFilter  implements Serializable   
 
 

 
 serialVersionUID:  -352890679895066592L

 
   
 
 
  
 Serialized Fields   
 
 

 
m_MinThreshold 
 
double  m_MinThreshold  
 
 the minimum threshold
 
 
 
 
 
 
m_MinDefault 
 
double  m_MinDefault  
 
 the minimum default replacement value
 
 
 
 
 
 
m_MaxThreshold 
 
double  m_MaxThreshold  
 
 the maximum threshold
 
 
 
 
 
 
m_MaxDefault 
 
double  m_MaxDefault  
 
 the maximum default replacement value
 
 
 
 
 
 
m_CloseTo 
 
double  m_CloseTo  
 
 the number the values are checked for closeness to
 
 
 
 
 
 
m_CloseToDefault 
 
double  m_CloseToDefault  
 
 the default replacement value for numbers "close-to"
 
 
 
 
 
 
m_CloseToTolerance 
 
double  m_CloseToTolerance  
 
 the tolerance distance, below which numbers are considered being "close-to"
 
 
 
 
 
 
m_Cols 
 
 Range   m_Cols  
 
 Stores which columns to cleanse
 
 
 
 
 
 
m_IncludeClass 
 
boolean  m_IncludeClass  
 
 whether to include the class attribute
 
 
 
 
 
 
m_Decimals 
 
int  m_Decimals  
 
 the number of decimals to round to (-1 means no rounding)
 
 
 
 

 
   
 
 
  
 Class  weka.filters.unsupervised.attribute.NumericToBinary  extends  PotentialClassIgnorer  implements Serializable   
 
 

 
 serialVersionUID:  2616879323359470802L

 

 
   
 
 
  
 Class  weka.filters.unsupervised.attribute.NumericToNominal  extends  SimpleBatchFilter  implements Serializable   
 
 

 
 serialVersionUID:  -6614630932899796239L

 
   
 
 
  
 Serialized Fields   
 
 

 
m_Cols 
 
 Range   m_Cols  
 
 Stores which columns to turn into nominals
 
 
 
 
 
 
m_DefaultCols 
 
java.lang.String  m_DefaultCols  
 
 The default columns to turn into nominals
 
 
 
 

 
   
 
 
  
 Class  weka.filters.unsupervised.attribute.NumericTransform  extends  Filter  implements Serializable   
 
 

 
 serialVersionUID:  -8561413333351366934L

 
   
 
 
  
 Serialized Fields   
 
 

 
m_Cols 
 
 Range   m_Cols  
 
 Stores which columns to transform.
 
 
 
 
 
 
m_Class 
 
java.lang.String  m_Class  
 
 Class containing transformation method.
 
 
 
 
 
 
m_Method 
 
java.lang.String  m_Method  
 
 Transformation method.
 
 
 
 

 
   
 
 
  
 Class  weka.filters.unsupervised.attribute.Obfuscate  extends  Filter  implements Serializable   
 
 

 
 serialVersionUID:  -343922772462971561L

 

 
   
 
 
  
 Class  weka.filters.unsupervised.attribute.PartitionedMultiFilter  extends  SimpleBatchFilter  implements Serializable   
 
 

 
 serialVersionUID:  -6293720886005713120L

 
   
 
 
  
 Serialized Fields   
 
 

 
m_Filters 
 
 Filter []  m_Filters  
 
 The filters.
 
 
 
 
 
 
m_Ranges 
 
 Range []  m_Ranges  
 
 The attribute ranges.
 
 
 
 
 
 
m_RemoveUnused 
 
boolean  m_RemoveUnused  
 
 Whether unused attributes are left out of the output.
 
 
 
 
 
 
m_IndicesUnused 
 
int[]  m_IndicesUnused  
 
 the indices of the unused attributes.
 
 
 
 

 
   
 
 
  
 Class  weka.filters.unsupervised.attribute.PKIDiscretize  extends  Discretize  implements Serializable   
 
 

 
 serialVersionUID:  6153101248977702675L

 

 
   
 
 
  
 Class  weka.filters.unsupervised.attribute.PotentialClassIgnorer  extends  Filter  implements Serializable   
 
 

 
 serialVersionUID:  8625371119276845454L

 
   
 
 
  
 Serialized Fields   
 
 

 
m_IgnoreClass 
 
boolean  m_IgnoreClass  
 
 True if the class is to be unset
 
 
 
 
 
 
m_ClassIndex 
 
int  m_ClassIndex  
 
 Storing the class index
 
 
 
 

 
   
 
 
  
 Class  weka.filters.unsupervised.attribute.PrincipalComponents  extends  Filter  implements Serializable   
 
 

 
 serialVersionUID:  -5649876869480249303L

 
   
 
 
  
 Serialized Fields   
 
 

 
m_TrainInstances 
 
 Instances   m_TrainInstances  
 
 The data to transform analyse/transform.
 
 
 
 
 
 
m_TrainCopy 
 
 Instances   m_TrainCopy  
 
 Keep a copy for the class attribute (if set).
 
 
 
 
 
 
m_TransformedFormat 
 
 Instances   m_TransformedFormat  
 
 The header for the transformed data format.
 
 
 
 
 
 
m_HasClass 
 
boolean  m_HasClass  
 
 Data has a class set.
 
 
 
 
 
 
m_ClassIndex 
 
int  m_ClassIndex  
 
 Class index.
 
 
 
 
 
 
m_NumAttribs 
 
int  m_NumAttribs  
 
 Number of attributes.
 
 
 
 
 
 
m_NumInstances 
 
int  m_NumInstances  
 
 Number of instances.
 
 
 
 
 
 
m_Correlation 
 
double[][]  m_Correlation  
 
 Correlation matrix for the original data.
 
 
 
 
 
 
m_center 
 
boolean  m_center  
 
 If true, center (rather than standardize) the data and
 compute PCA from covariance (rather than correlation)
 matrix.
 
 
 
 
 
 
m_Eigenvectors 
 
double[][]  m_Eigenvectors  
 
 Will hold the unordered linear transformations of the (normalized)
      original data.
 
 
 
 
 
 
m_Eigenvalues 
 
double[]  m_Eigenvalues  
 
 Eigenvalues for the corresponding eigenvectors.
 
 
 
 
 
 
m_SortedEigens 
 
int[]  m_SortedEigens  
 
 Sorted eigenvalues.
 
 
 
 
 
 
m_SumOfEigenValues 
 
double  m_SumOfEigenValues  
 
 sum of the eigenvalues.
 
 
 
 
 
 
m_ReplaceMissingFilter 
 
 ReplaceMissingValues   m_ReplaceMissingFilter  
 
 Filters for replacing missing values.
 
 
 
 
 
 
m_NominalToBinaryFilter 
 
 NominalToBinary   m_NominalToBinaryFilter  
 
 Filter for turning nominal values into numeric ones.
 
 
 
 
 
 
m_AttributeFilter 
 
 Remove   m_AttributeFilter  
 
 Filter for removing class attribute, nominal attributes with 0 or 1 value.
 
 
 
 
 
 
m_standardizeFilter 
 
 Standardize   m_standardizeFilter  
 
 Filter for standardizing the data
 
 
 
 
 
 
m_centerFilter 
 
 Center   m_centerFilter  
 
 Filter for centering the data
 
 
 
 
 
 
m_OutputNumAtts 
 
int  m_OutputNumAtts  
 
 The number of attributes in the pc transformed data.
 
 
 
 
 
 
m_CoverVariance 
 
double  m_CoverVariance  
 
 the amount of varaince to cover in the original data when
      retaining the best n PC's.
 
 
 
 
 
 
m_MaxAttrsInName 
 
int  m_MaxAttrsInName  
 
 maximum number of attributes in the transformed attribute name.
 
 
 
 
 
 
m_MaxAttributes 
 
int  m_MaxAttributes  
 
 maximum number of attributes in the transformed data (-1 for all).
 
 
 
 

 
   
 
 
  
 Class  weka.filters.unsupervised.attribute.PropositionalToMultiInstance  extends  Filter  implements Serializable   
 
 

 
 serialVersionUID:  5825873573912102482L

 
   
 
 
  
 Serialized Fields   
 
 

 
m_Seed 
 
int  m_Seed  
 
 the seed for randomizing, default is 1
 
 
 
 
 
 
m_Randomize 
 
boolean  m_Randomize  
 
 whether to randomize the output data
 
 
 
 
 
 
m_BagStringAtts 
 
 StringLocator   m_BagStringAtts  
 
 Indices of string attributes in the bag
 
 
 
 
 
 
m_BagRelAtts 
 
 RelationalLocator   m_BagRelAtts  
 
 Indices of relational attributes in the bag
 
 
 
 

 
   
 
 
  
 Class  weka.filters.unsupervised.attribute.RandomProjection  extends  Filter  implements Serializable   
 
 

 
 serialVersionUID:  4428905532728645880L

 
   
 
 
  
 Serialized Fields   
 
 

 
m_k 
 
int  m_k  
 
 Stores the number of dimensions to reduce the data to
 
 
 
 
 
 
m_percent 
 
double  m_percent  
 
 Stores the dimensionality the data should be reduced to as percentage of the original dimension
 
 
 
 
 
 
m_useGaussian 
 
boolean  m_useGaussian  
 
 Is the random matrix will be computed using 
      Gaussian distribution or not
 
 
 
 
 
 
m_distribution 
 
int  m_distribution  
 
 Stores the distribution to use for calculating the
      random matrix
 
 
 
 
 
 
m_useReplaceMissing 
 
boolean  m_useReplaceMissing  
 
 Should the missing values be replaced using 
      unsupervised.ReplaceMissingValues filter
 
 
 
 
 
 
m_OutputFormatDefined 
 
boolean  m_OutputFormatDefined  
 
 Keeps track of output format if it is defined or not
 
 
 
 
 
 
m_ntob 
 
 Filter   m_ntob  
 
 The NominalToBinary filter applied to the data before this filter
 
 
 
 
 
 
m_replaceMissing 
 
 Filter   m_replaceMissing  
 
 The ReplaceMissingValues filter
 
 
 
 
 
 
m_rndmSeed 
 
long  m_rndmSeed  
 
 Stores the random seed used to generate the random matrix
 
 
 
 
 
 
m_rmatrix 
 
double[][]  m_rmatrix  
 
 The random matrix
 
 
 
 
 
 
m_random 
 
java.util.Random  m_random  
 
 The random number generator used for generating the random matrix
 
 
 
 

 
   
 
 
  
 Class  weka.filters.unsupervised.attribute.RandomSubset  extends  SimpleStreamFilter  implements Serializable   
 
 

 
 serialVersionUID:  2911221724251628050L

 
   
 
 
  
 Serialized Fields   
 
 

 
m_NumAttributes 
 
double  m_NumAttributes  
 
 The number of attributes to randomly choose (>= 1 absolute number of
 attributes, < 1 percentage).
 
 
 
 
 
 
m_Seed 
 
int  m_Seed  
 
 The seed value.
 
 
 
 
 
 
m_Indices 
 
int[]  m_Indices  
 
 The indices of the attributes that got selected.
 
 
 
 

 
   
 
 
  
 Class  weka.filters.unsupervised.attribute.RELAGGS  extends  SimpleBatchFilter  implements Serializable   
 
 

 
 serialVersionUID:  -3333791375278589231L

 
   
 
 
  
 Serialized Fields   
 
 

 
m_MaxCardinality 
 
int  m_MaxCardinality  
 
 the max. cardinality for nominal attributes
 
 
 
 
 
 
m_SelectedRange 
 
 Range   m_SelectedRange  
 
 the range of attributes to process (only relational ones will be processed)
 
 
 
 
 
 
m_AttStats 
 
java.util.Hashtable<K,V>  m_AttStats  
 
 stores the attribute statistics 
  att_index-att_index_in_rel_att <-> AttributeStats 
 
 
 
 

 
   
 
 
  
 Class  weka.filters.unsupervised.attribute.Remove  extends  Filter  implements Serializable   
 
 

 
 serialVersionUID:  5011337331921522847L

 
   
 
 
  
 Serialized Fields   
 
 

 
m_SelectCols 
 
 Range   m_SelectCols  
 
 Stores which columns to select as a funky range
 
 
 
 
 
 
m_SelectedAttributes 
 
int[]  m_SelectedAttributes  
 
 Stores the indexes of the selected attributes in order, once the
 dataset is seen
 
 
 
 

 
   
 
 
  
 Class  weka.filters.unsupervised.attribute.RemoveType  extends  Filter  implements Serializable   
 
 

 
 serialVersionUID:  -3563999462782486279L

 
   
 
 
  
 Serialized Fields   
 
 

 
m_attributeFilter 
 
 Remove   m_attributeFilter  
 
 The attribute filter used to do the filtering
 
 
 
 
 
 
m_attTypeToDelete 
 
int  m_attTypeToDelete  
 
 The type of attribute to delete
 
 
 
 
 
 
m_invert 
 
boolean  m_invert  
 
 Whether to invert selection
 
 
 
 

 
   
 
 
  
 Class  weka.filters.unsupervised.attribute.RemoveUseless  extends  Filter  implements Serializable   
 
 

 
 serialVersionUID:  -8659417851407640038L

 
   
 
 
  
 Serialized Fields   
 
 

 
m_removeFilter 
 
 Remove   m_removeFilter  
 
 The filter used to remove attributes
 
 
 
 
 
 
m_maxVariancePercentage 
 
double  m_maxVariancePercentage  
 
 The type of attribute to delete
 
 
 
 

 
   
 
 
  
 Class  weka.filters.unsupervised.attribute.Reorder  extends  Filter  implements Serializable   
 
 

 
 serialVersionUID:  -1135571321097202292L

 
   
 
 
  
 Serialized Fields   
 
 

 
m_NewOrderCols 
 
java.lang.String  m_NewOrderCols  
 
 Stores which columns to reorder
 
 
 
 
 
 
m_SelectedAttributes 
 
int[]  m_SelectedAttributes  
 
 Stores the indexes of the selected attributes in order, once the
 dataset is seen
 
 
 
 
 
 
m_InputStringIndex 
 
int[]  m_InputStringIndex  
 
 Contains an index of string attributes in the input format
 that survive the filtering process -- some entries may be duplicated
 
 
 
 

 
   
 
 
  
 Class  weka.filters.unsupervised.attribute.ReplaceMissingValues  extends  PotentialClassIgnorer  implements Serializable   
 
 

 
 serialVersionUID:  8349568310991609867L

 
   
 
 
  
 Serialized Fields   
 
 

 
m_ModesAndMeans 
 
double[]  m_ModesAndMeans  
 
 The modes and means
 
 
 
 

 
   
 
 
  
 Class  weka.filters.unsupervised.attribute.Standardize  extends  PotentialClassIgnorer  implements Serializable   
 
 

 
 serialVersionUID:  -6830769026855053281L

 
   
 
 
  
 Serialized Fields   
 
 

 
m_Means 
 
double[]  m_Means  
 
 The means
 
 
 
 
 
 
m_StdDevs 
 
double[]  m_StdDevs  
 
 The variances
 
 
 
 

 
   
 
 
  
 Class  weka.filters.unsupervised.attribute.StringToNominal  extends  Filter  implements Serializable   
 
 

 
 serialVersionUID:  4864084427902797605L

 
   
 
 
  
 Serialized Fields   
 
 

 
m_AttIndices 
 
 Range   m_AttIndices  
 
 The attribute's range indices setting.
 
 
 
 

 
   
 
 
  
 Class  weka.filters.unsupervised.attribute.StringToWordVector  extends  Filter  implements Serializable   
 
 

 
 serialVersionUID:  8249106275278565424L

 
   
 
 
  
 Serialized Fields   
 
 

 
m_SelectedRange 
 
 Range   m_SelectedRange  
 
 Range of columns to convert to word vectors.
 
 
 
 
 
 
m_Dictionary 
 
java.util.TreeMap<K,V>  m_Dictionary  
 
 Contains a mapping of valid words to attribute indexes.
 
 
 
 
 
 
m_OutputCounts 
 
boolean  m_OutputCounts  
 
 True if output instances should contain word frequency rather than boolean 0 or 1.
 
 
 
 
 
 
m_Prefix 
 
java.lang.String  m_Prefix  
 
 A String prefix for the attribute names.
 
 
 
 
 
 
m_DocsCounts 
 
int[]  m_DocsCounts  
 
 Contains the number of documents (instances) a particular word appears in.
          The counts are stored with the same indexing as given by m_Dictionary.
 
 
 
 
 
 
m_NumInstances 
 
int  m_NumInstances  
 
 Contains the number of documents (instances) in the input format from 
          which the dictionary is created. It is used in IDF transform.
 
 
 
 
 
 
m_AvgDocLength 
 
double  m_AvgDocLength  
 
 Contains the average length of documents (among the first batch of 
 instances aka training data). This is used in length normalization of 
 documents which will be normalized to average document length.
 
 
 
 
 
 
m_WordsToKeep 
 
int  m_WordsToKeep  
 
 The default number of words (per class if there is a class attribute
 assigned) to attempt to keep.
 
 
 
 
 
 
m_PeriodicPruningRate 
 
double  m_PeriodicPruningRate  
 
 The percentage at which to periodically prune the dictionary.
 
 
 
 
 
 
m_TFTransform 
 
boolean  m_TFTransform  
 
 True if word frequencies should be transformed into log(1+fi) 
          where fi is the frequency of word i.
 
 
 
 
 
 
m_filterType 
 
int  m_filterType  
 
 The normalization to apply.
 
 
 
 
 
 
m_IDFTransform 
 
boolean  m_IDFTransform  
 
 True if word frequencies should be transformed into 
          fij*log(numOfDocs/numOfDocsWithWordi).
 
 
 
 
 
 
m_lowerCaseTokens 
 
boolean  m_lowerCaseTokens  
 
 True if all tokens should be downcased.
 
 
 
 
 
 
m_useStoplist 
 
boolean  m_useStoplist  
 
 True if tokens that are on a stoplist are to be ignored.
 
 
 
 
 
 
m_Stemmer 
 
 Stemmer   m_Stemmer  
 
 the stemming algorithm.
 
 
 
 
 
 
m_minTermFreq 
 
int  m_minTermFreq  
 
 the minimum (per-class) word frequency.
 
 
 
 
 
 
m_doNotOperateOnPerClassBasis 
 
boolean  m_doNotOperateOnPerClassBasis  
 
 whether to operate on a per-class basis.
 
 
 
 
 
 
m_Stopwords 
 
java.io.File  m_Stopwords  
 
 a file containing stopwords for using others than the default Rainbow 
 ones.
 
 
 
 
 
 
m_Tokenizer 
 
 Tokenizer   m_Tokenizer  
 
 the tokenizer algorithm to use.
 
 
 
 

 
   
 
 
  
 Class  weka.filters.unsupervised.attribute.SwapValues  extends  Filter  implements Serializable   
 
 

 
 serialVersionUID:  6155834679414275855L

 
   
 
 
  
 Serialized Fields   
 
 

 
m_AttIndex 
 
 SingleIndex   m_AttIndex  
 
 The attribute's index setting.
 
 
 
 
 
 
m_FirstIndex 
 
 SingleIndex   m_FirstIndex  
 
 The first value's index setting.
 
 
 
 
 
 
m_SecondIndex 
 
 SingleIndex   m_SecondIndex  
 
 The second value's index setting.
 
 
 
 

 
   
 
 
  
 Class  weka.filters.unsupervised.attribute.TimeSeriesDelta  extends  TimeSeriesTranslate  implements Serializable   
 
 

 
 serialVersionUID:  3101490081896634942L

 

 
   
 
 
  
 Class  weka.filters.unsupervised.attribute.TimeSeriesTranslate  extends  AbstractTimeSeries  implements Serializable   
 
 

 
 serialVersionUID:  -8901621509691785705L

 

 
   
 
 
  
 Class  weka.filters.unsupervised.attribute.Wavelet  extends  SimpleBatchFilter  implements Serializable   
 
 

 
 serialVersionUID:  -3335106965521265631L

 
   
 
 
  
 Serialized Fields   
 
 

 
m_Filter 
 
 Filter   m_Filter  
 
 an optional filter for preprocessing of the data
 
 
 
 
 
 
m_Algorithm 
 
int  m_Algorithm  
 
 the type of algorithm
 
 
 
 
 
 
m_Padding 
 
int  m_Padding  
 
 the type of padding
 
 
 
 
 

 
 
  
 Package   weka.filters.unsupervised.instance   
 
 

 
   
 
 
  
 Class  weka.filters.unsupervised.instance.NonSparseToSparse  extends  Filter  implements Serializable   
 
 

 
 serialVersionUID:  4694489111366063852L

 

 
   
 
 
  
 Class  weka.filters.unsupervised.instance.Normalize  extends  Filter  implements Serializable   
 
 

 
 serialVersionUID:  -7947971807522917395L

 
   
 
 
  
 Serialized Fields   
 
 

 
m_Norm 
 
double  m_Norm  
 
 The norm that each instance must have at the end
 
 
 
 
 
 
m_LNorm 
 
double  m_LNorm  
 
 The L-norm to use
 
 
 
 

 
   
 
 
  
 Class  weka.filters.unsupervised.instance.Randomize  extends  Filter  implements Serializable   
 
 

 
 serialVersionUID:  8854479785121877582L

 
   
 
 
  
 Serialized Fields   
 
 

 
m_Seed 
 
int  m_Seed  
 
 The random number seed
 
 
 
 
 
 
m_Random 
 
java.util.Random  m_Random  
 
 The current random number generator
 
 
 
 

 
   
 
 
  
 Class  weka.filters.unsupervised.instance.RemoveFolds  extends  Filter  implements Serializable   
 
 

 
 serialVersionUID:  8220373305559055700L

 
   
 
 
  
 Serialized Fields   
 
 

 
m_Inverse 
 
boolean  m_Inverse  
 
 Indicates if inverse of selection is to be output.
 
 
 
 
 
 
m_NumFolds 
 
int  m_NumFolds  
 
 Number of folds to split dataset into
 
 
 
 
 
 
m_Fold 
 
int  m_Fold  
 
 Fold to output
 
 
 
 
 
 
m_Seed 
 
long  m_Seed  
 
 Random number seed.
 
 
 
 

 
   
 
 
  
 Class  weka.filters.unsupervised.instance.RemoveFrequentValues  extends  Filter  implements Serializable   
 
 

 
 serialVersionUID:  -2447432930070059511L

 
   
 
 
  
 Serialized Fields   
 
 

 
m_AttIndex 
 
 SingleIndex   m_AttIndex  
 
 The attribute's index setting.
 
 
 
 
 
 
m_NumValues 
 
int  m_NumValues  
 
 the number of values to retain.
 
 
 
 
 
 
m_LeastValues 
 
boolean  m_LeastValues  
 
 whether to retain values with least instances instead of most.
 
 
 
 
 
 
m_Invert 
 
boolean  m_Invert  
 
 whether to invert the matching sense.
 
 
 
 
 
 
m_ModifyHeader 
 
boolean  m_ModifyHeader  
 
 Modify header for nominal attributes?
 
 
 
 
 
 
m_NominalMapping 
 
int[]  m_NominalMapping  
 
 If m_ModifyHeader, stores a mapping from old to new indexes
 
 
 
 
 
 
m_Values 
 
java.util.HashSet<E>  m_Values  
 
 contains the values to retain
 
 
 
 

 
   
 
 
  
 Class  weka.filters.unsupervised.instance.RemoveMisclassified  extends  Filter  implements Serializable   
 
 

 
 serialVersionUID:  5469157004717663171L

 
   
 
 
  
 Serialized Fields   
 
 

 
m_cleansingClassifier 
 
 Classifier   m_cleansingClassifier  
 
 The classifier used to do the cleansing
 
 
 
 
 
 
m_classIndex 
 
int  m_classIndex  
 
 The attribute to treat as the class for purposes of cleansing.
 
 
 
 
 
 
m_numOfCrossValidationFolds 
 
int  m_numOfCrossValidationFolds  
 
 The number of cross validation folds to perform (<2 = no cross validation)
 
 
 
 
 
 
m_numOfCleansingIterations 
 
int  m_numOfCleansingIterations  
 
 The maximum number of cleansing iterations to perform (<1 = until fully cleansed)
 
 
 
 
 
 
m_numericClassifyThreshold 
 
double  m_numericClassifyThreshold  
 
 The threshold for deciding when a numeric value is correctly classified
 
 
 
 
 
 
m_invertMatching 
 
boolean  m_invertMatching  
 
 Whether to invert the match so the correctly classified instances are discarded
 
 
 
 
 
 
m_firstBatchFinished 
 
boolean  m_firstBatchFinished  
 
 Have we processed the first batch (i.e. training data)?
 
 
 
 

 
   
 
 
  
 Class  weka.filters.unsupervised.instance.RemovePercentage  extends  Filter  implements Serializable   
 
 

 
 serialVersionUID:  2150341191158533133L

 
   
 
 
  
 Serialized Fields   
 
 

 
m_Percentage 
 
double  m_Percentage  
 
 Percentage of instances to select.
 
 
 
 
 
 
m_Inverse 
 
boolean  m_Inverse  
 
 Indicates if inverse of selection is to be output.
 
 
 
 

 
   
 
 
  
 Class  weka.filters.unsupervised.instance.RemoveRange  extends  Filter  implements Serializable   
 
 

 
 serialVersionUID:  -3064641215340828695L

 
   
 
 
  
 Serialized Fields   
 
 

 
m_Range 
 
 Range   m_Range  
 
 Range of instances requested by the user.
 
 
 
 

 
   
 
 
  
 Class  weka.filters.unsupervised.instance.RemoveWithValues  extends  Filter  implements Serializable   
 
 

 
 serialVersionUID:  4752870193679263361L

 
   
 
 
  
 Serialized Fields   
 
 

 
m_AttIndex 
 
 SingleIndex   m_AttIndex  
 
 The attribute's index setting.
 
 
 
 
 
 
m_Values 
 
 Range   m_Values  
 
 Stores which values of nominal attribute are to be used for filtering.
 
 
 
 
 
 
m_Value 
 
double  m_Value  
 
 Stores which value of a numeric attribute is to be used for filtering.
 
 
 
 
 
 
m_MatchMissingValues 
 
boolean  m_MatchMissingValues  
 
 True if missing values should count as a match
 
 
 
 
 
 
m_ModifyHeader 
 
boolean  m_ModifyHeader  
 
 Modify header for nominal attributes?
 
 
 
 
 
 
m_NominalMapping 
 
int[]  m_NominalMapping  
 
 If m_ModifyHeader, stores a mapping from old to new indexes
 
 
 
 
 
 
m_dontFilterAfterFirstBatch 
 
boolean  m_dontFilterAfterFirstBatch  
 
 Whether to filter instances after the first batch has been processed
 
 
 
 

 
   
 
 
  
 Class  weka.filters.unsupervised.instance.Resample  extends  Filter  implements Serializable   
 
 

 
 serialVersionUID:  3119607037607101160L

 
   
 
 
  
 Serialized Fields   
 
 

 
m_SampleSizePercent 
 
double  m_SampleSizePercent  
 
 The subsample size, percent of original set, default 100%
 
 
 
 
 
 
m_RandomSeed 
 
int  m_RandomSeed  
 
 The random number generator seed
 
 
 
 
 
 
m_NoReplacement 
 
boolean  m_NoReplacement  
 
 Whether to perform sampling with replacement or without
 
 
 
 
 
 
m_InvertSelection 
 
boolean  m_InvertSelection  
 
 Whether to invert the selection (only if instances are drawn WITHOUT 
 replacement)
 
 
  See Also:    Resample.m_NoReplacement   
 

 
   
 
 
  
 Class  weka.filters.unsupervised.instance.ReservoirSample  extends  Filter  implements Serializable   
 
 

 
 serialVersionUID:  3119607037607101160L

 
   
 
 
  
 Serialized Fields   
 
 

 
m_SampleSize 
 
int  m_SampleSize  
 
 The subsample size, number of instances%
 
 
 
 
 
 
m_subSample 
 
 Instance []  m_subSample  
 
 Holds the sub-sample (reservoir)
 
 
 
 
 
 
m_currentInst 
 
int  m_currentInst  
 
 The current instance being processed
 
 
 
 
 
 
m_RandomSeed 
 
int  m_RandomSeed  
 
 The random number generator seed
 
 
 
 
 
 
m_random 
 
java.util.Random  m_random  
 
 The random number generator
 
 
 
 

 
   
 
 
  
 Class  weka.filters.unsupervised.instance.SparseToNonSparse  extends  Filter  implements Serializable   
 
 

 
 serialVersionUID:  2481634184210236074L

 

 
   
 
 
  
 Class  weka.filters.unsupervised.instance.SubsetByExpression  extends  SimpleBatchFilter  implements Serializable   
 
 

 
 serialVersionUID:  5628686110979589602L

 
   
 
 
  
 Serialized Fields   
 
 

 
m_Expression 
 
java.lang.String  m_Expression  
 
 the expresion to use for filtering.
 
 
 
 
 
 
m_filterAfterFirstBatch 
 
boolean  m_filterAfterFirstBatch  
 
 Whether to filter instances after the first batch has been processed
 
 
 
 
 

 
 
  
 Package   weka.gui   
 
 

 
   
 
 
  
 Class  weka.gui.AttributeListPanel  extends javax.swing.JPanel implements Serializable   
 
 

 
 serialVersionUID:  -2030706987910400362L

 
   
 
 
  
 Serialized Fields   
 
 

 
m_Table 
 
javax.swing.JTable  m_Table  
 
 The table displaying attribute names
 
 
 
 
 
 
m_Model 
 
weka.gui.AttributeListPanel.AttributeTableModel  m_Model  
 
 The table model containing attribute names
 
 
 
 

 
   
 
 
  
 Class  weka.gui.AttributeSelectionPanel  extends javax.swing.JPanel implements Serializable   
 
 

 
 serialVersionUID:  627131485290359194L

 
   
 
 
  
 Serialized Fields   
 
 

 
m_IncludeAll 
 
javax.swing.JButton  m_IncludeAll  
 
 Press to select all attributes
 
 
 
 
 
 
m_RemoveAll 
 
javax.swing.JButton  m_RemoveAll  
 
 Press to deselect all attributes
 
 
 
 
 
 
m_Invert 
 
javax.swing.JButton  m_Invert  
 
 Press to invert the current selection
 
 
 
 
 
 
m_Pattern 
 
javax.swing.JButton  m_Pattern  
 
 Press to enter a perl regular expression for selection
 
 
 
 
 
 
m_Table 
 
javax.swing.JTable  m_Table  
 
 The table displaying attribute names and selection status
 
 
 
 
 
 
m_Model 
 
weka.gui.AttributeSelectionPanel.AttributeTableModel  m_Model  
 
 The table model containing attribute names and selection status
 
 
 
 
 
 
m_PatternRegEx 
 
java.lang.String  m_PatternRegEx  
 
 The current regular expression.
 
 
 
 

 
   
 
 
  
 Class  weka.gui.AttributeSummaryPanel  extends javax.swing.JPanel implements Serializable   
 
 

 
 serialVersionUID:  -5434987925737735880L

 
   
 
 
  
 Serialized Fields   
 
 

 
m_AttributeNameLab 
 
javax.swing.JLabel  m_AttributeNameLab  
 
 Displays the name of the relation
 
 
 
 
 
 
m_AttributeTypeLab 
 
javax.swing.JLabel  m_AttributeTypeLab  
 
 Displays the type of attribute
 
 
 
 
 
 
m_MissingLab 
 
javax.swing.JLabel  m_MissingLab  
 
 Displays the number of missing values
 
 
 
 
 
 
m_UniqueLab 
 
javax.swing.JLabel  m_UniqueLab  
 
 Displays the number of unique values
 
 
 
 
 
 
m_DistinctLab 
 
javax.swing.JLabel  m_DistinctLab  
 
 Displays the number of distinct values
 
 
 
 
 
 
m_StatsTable 
 
javax.swing.JTable  m_StatsTable  
 
 Displays other stats in a table
 
 
 
 
 
 
m_Instances 
 
 Instances   m_Instances  
 
 The instances we're playing with
 
 
 
 
 
 
m_AttributeStats 
 
 AttributeStats []  m_AttributeStats  
 
 Cached stats on the attributes we've summarized so far
 
 
 
 

 
   
 
 
  
 Class  weka.gui.AttributeVisualizationPanel  extends  PrintablePanel  implements Serializable   
 
 

 
 serialVersionUID:  -8650490488825371193L

 
   
 
 
  
 Serialized Fields   
 
 

 
m_data 
 
 Instances   m_data  
 
 This holds the current set of instances
 
 
 
 
 
 
m_as 
 
 AttributeStats   m_as  
 
 This holds the attribute stats of the current attribute on display. It is
 calculated in setAttribute(int idx) when it is called to set a new
 attribute index.
 
 
 
 
 
 
m_asCache 
 
 AttributeStats []  m_asCache  
 
 Cache of attribute stats info for the current data set
 
 
 
 
 
 
m_attribIndex 
 
int  m_attribIndex  
 
 This holds the index of the current attribute on display and should be
  set through setAttribute(int idx).
 
 
 
 
 
 
m_maxValue 
 
int  m_maxValue  
 
 This holds the max value of the current attribute. In case of nominal
 attribute it is the highest count that a nominal value has in the
 attribute (given by m_as.nominalCounts[i]), otherwise in case of numeric
 attribute it is simply the maximum value present in the attribute (given by
 m_as.numericStats.max). It is used to calculate the ratio of the height of
 the bars with respect to the height of the display area.
 
 
 
 
 
 
m_histBarCounts 
 
int[]  m_histBarCounts  
 
 This array holds the count (or height) for the each of the bars in a
 barplot or a histogram. In case of barplots (and current attribute being
 nominal) its length (and the number of bars) is equal to the number of
 nominal values in the current attribute, with each field of the array being
 equal to the count of each nominal that it represents (the count of ith
 nominal value of an attribute is given by m_as.nominalCounts[i]). Whereas,
 in case of histograms (and current attribute being numeric) the width of
 its intervals is calculated by Scott's(1979) method:  
    intervalWidth = Max(1, 3.49*Std.Dev*numInstances^(1/3))
 And the number of intervals by:  
   intervals = max(1, Math.round(Range/intervalWidth);
 Then each field of this array contains the number of values of the current
 attribute that fall in the histogram interval that it represents.  
 NOTE: The values of this array are only calculated if the class attribute
 is not set or if it is numeric.
 
 
 
 
 
 
m_histBarClassCounts 
 
 SparseInstance []  m_histBarClassCounts  
 
 This array holds the per class count (or per class height) of the each of
 the bars in a barplot or a histogram.
 For nominal attributes the format is:  
    m_histBarClassCounts[nominalValue][classValue+1].
 For numeric attributes the format is:  
    m_histBarClassCounts[interval][classValues+1],  
      where the number of intervals is calculated by the Scott's method as
            mentioned above.
 The array is initialized to have 1+numClasses to accomodate for instances
 with missing class value. The ones with missing class value are displayed
 as a black sub par in a histogram or a barplot.

 NOTE: The values of this array are only calculated if the class attribute
 is set and it is nominal.
 
 
 
 
 
 
m_barRange 
 
double  m_barRange  
 
 Contains the range of each bar in a histogram. It is used to work out the
 range of bar the mouse pointer is on in getToolTipText().
 
 
 
 
 
 
m_classIndex 
 
int  m_classIndex  
 
 Contains the current class index.
 
 
 
 
 
 
m_hc 
 
java.lang.Thread  m_hc  
 
 This stores the BarCalc or HistCalc thread while a new barplot or
 histogram is being calculated.
 
 
 
 
 
 
m_threadRun 
 
boolean  m_threadRun  
 
 True if the thread m_hc above is running.
 
 
 
 
 
 
m_doneCurrentAttribute 
 
boolean  m_doneCurrentAttribute  
 
 
 
 
 
 
m_displayCurrentAttribute 
 
boolean  m_displayCurrentAttribute  
 
 
 
 
 
 
m_colorAttrib 
 
javax.swing.JComboBox  m_colorAttrib  
 
 This stores and lets the user select a class attribute. It also has
 an entry "No Class" if the user does not want to set a class attribute
 for colouring.
 
 
 
 
 
 
m_fm 
 
java.awt.FontMetrics  m_fm  
 
 Fontmetrics used to get the font size which is required for calculating
 displayable area size, bar height ratio and width of strings that are
 displayed on top of bars indicating their count.
 
 
 
 
 
 
m_locker 
 
java.lang.Integer  m_locker  
 
 Lock variable to synchronize the different threads running currently in
 this class. There are two to three threads in this class, AWT paint thread
 which is handled differently in paintComponent() which checks on
 m_threadRun to determine if it can perform full paint or not, the second
 thread is the main execution thread and the third is the one represented by
 m_hc which we start when we want to calculate the internal fields for a bar
 plot or a histogram.
 
 
 
 
 
 
m_colorList 
 
 FastVector   m_colorList  
 
 Contains discrete colours for colouring of subbars of histograms and
 bar plots when the class attribute is set and is nominal
 
 
 
 

 
   
 
 
  
 Class  weka.gui.CheckBoxList  extends javax.swing.JList implements Serializable   
 
 

 
 serialVersionUID:  -4359573373359270258L

 

 
   
 
 
  
 Class  weka.gui.CheckBoxList.CheckBoxListModel  extends javax.swing.DefaultListModel implements Serializable   
 
 

 
 serialVersionUID:  7772455499540273507L

 

 
   
 
 
  
 Class  weka.gui.CheckBoxList.CheckBoxListRenderer  extends javax.swing.JCheckBox implements Serializable   
 
 

 
 serialVersionUID:  1059591605858524586L

 

 
   
 
 
  
 Class  weka.gui.ConverterFileChooser  extends javax.swing.JFileChooser implements Serializable   
 
 

 
 serialVersionUID:  -5373058011025481738L

 
   
 
 
  
 Serialized Fields   
 
 

 
m_Self 
 
 ConverterFileChooser   m_Self  
 
 the file chooser itself
 
 
 
 
 
 
m_DialogType 
 
int  m_DialogType  
 
 the type of dialog to display
 
 
 
 
 
 
m_CurrentConverter 
 
java.lang.Object  m_CurrentConverter  
 
 the converter that was chosen by the user
 
 
 
 
 
 
m_ConfigureButton 
 
javax.swing.JButton  m_ConfigureButton  
 
 the configure button
 
 
 
 
 
 
m_Listener 
 
java.beans.PropertyChangeListener  m_Listener  
 
 the propertychangelistener
 
 
 
 
 
 
m_LastFilter 
 
javax.swing.filechooser.FileFilter  m_LastFilter  
 
 the last filter that was used for opening/saving
 
 
 
 
 
 
m_CapabilitiesFilter 
 
 Capabilities   m_CapabilitiesFilter  
 
 the Capabilities filter for the savers
 
 
 
 
 
 
m_OverwriteWarning 
 
boolean  m_OverwriteWarning  
 
 whether to popup a dialog in case the file already exists (only save
 dialog)
 
 
 
 
 
 
m_FileMustExist 
 
boolean  m_FileMustExist  
 
 whether the file to be opened must exist (only open dialog)
 
 
 
 
 
 
m_CoreConvertersOnly 
 
boolean  m_CoreConvertersOnly  
 
 whether to display only core converters (hardcoded in ConverterUtils).
 Necessary for RMI/Remote Experiments for instance.
 
 
  See Also:    ConverterUtils.CORE_FILE_LOADERS  , 
  ConverterUtils.CORE_FILE_SAVERS   
 

 
   
 
 
  
 Class  weka.gui.DatabaseConnectionDialog  extends javax.swing.JDialog implements Serializable   
 
 

 
 serialVersionUID:  -1081946748666245054L

 
   
 
 
  
 Serialized Fields   
 
 

 
m_DbaseURLText 
 
javax.swing.JTextField  m_DbaseURLText  
 
 
 
 
 
 
m_DbaseURLLab 
 
javax.swing.JLabel  m_DbaseURLLab  
 
 
 
 
 
 
m_UserNameText 
 
javax.swing.JTextField  m_UserNameText  
 
 
 
 
 
 
m_UserNameLab 
 
javax.swing.JLabel  m_UserNameLab  
 
 
 
 
 
 
m_PasswordText 
 
javax.swing.JPasswordField  m_PasswordText  
 
 
 
 
 
 
m_PasswordLab 
 
javax.swing.JLabel  m_PasswordLab  
 
 
 
 
 
 
m_DebugCheckBox 
 
javax.swing.JCheckBox  m_DebugCheckBox  
 
 
 
 
 
 
m_DebugLab 
 
javax.swing.JLabel  m_DebugLab  
 
 
 
 
 
 
m_returnValue 
 
int  m_returnValue  
 
 
 
 

 
   
 
 
  
 Class  weka.gui.ExtensionFileFilter  extends javax.swing.filechooser.FileFilter implements Serializable   
 
 

 
   
 
 
  
 Serialized Fields   
 
 

 
m_Description 
 
java.lang.String  m_Description  
 
 The text description of the types of files accepted
 
 
 
 
 
 
m_Extension 
 
java.lang.String[]  m_Extension  
 
 The filename extensions of accepted files
 
 
 
 

 
   
 
 
  
 Class  weka.gui.GenericArrayEditor  extends javax.swing.JPanel implements Serializable   
 
 

 
 serialVersionUID:  3914616975334750480L

 
   
 
 
  
 Serialized Fields   
 
 

 
m_Support 
 
java.beans.PropertyChangeSupport  m_Support  
 
 Handles property change notification.
 
 
 
 
 
 
m_Label 
 
javax.swing.JLabel  m_Label  
 
 The label for when we can't edit that type.
 
 
 
 
 
 
m_ElementList 
 
javax.swing.JList  m_ElementList  
 
 The list component displaying current values.
 
 
 
 
 
 
m_ElementClass 
 
java.lang.Class<T>  m_ElementClass  
 
 The class of objects allowed in the array.
 
 
 
 
 
 
m_ListModel 
 
javax.swing.DefaultListModel  m_ListModel  
 
 The defaultlistmodel holding our data.
 
 
 
 
 
 
m_ElementEditor 
 
java.beans.PropertyEditor  m_ElementEditor  
 
 The property editor for the class we are editing.
 
 
 
 
 
 
m_DeleteBut 
 
javax.swing.JButton  m_DeleteBut  
 
 Click this to delete the selected array values.
 
 
 
 
 
 
m_EditBut 
 
javax.swing.JButton  m_EditBut  
 
 Click this to edit the selected array value.
 
 
 
 
 
 
m_UpBut 
 
javax.swing.JButton  m_UpBut  
 
 Click this to move the selected array value(s) one up.
 
 
 
 
 
 
m_DownBut 
 
javax.swing.JButton  m_DownBut  
 
 Click this to move the selected array value(s) one down.
 
 
 
 
 
 
m_AddBut 
 
javax.swing.JButton  m_AddBut  
 
 Click to add the current object configuration to the array.
 
 
 
 
 
 
m_Editor 
 
java.beans.PropertyEditor  m_Editor  
 
 The property editor for editing existing elements.
 
 
 
 
 
 
m_PD 
 
 PropertyDialog   m_PD  
 
 The currently displayed property dialog, if any.
 
 
 
 
 
 
m_InnerActionListener 
 
java.awt.event.ActionListener  m_InnerActionListener  
 
 Listens to buttons being pressed and taking the appropriate action.
 
 
 
 
 
 
m_InnerSelectionListener 
 
javax.swing.event.ListSelectionListener  m_InnerSelectionListener  
 
 Listens to list items being selected and takes appropriate action.
 
 
 
 
 
 
m_InnerMouseListener 
 
java.awt.event.MouseListener  m_InnerMouseListener  
 
 Listens to mouse events and takes appropriate action.
 
 
 
 

 
   
 
 
  
 Class  weka.gui.GenericObjectEditor.CapabilitiesFilterDialog  extends javax.swing.JDialog implements Serializable   
 
 

 
 serialVersionUID:  -7845503345689646266L

 
   
 
 
  
 Serialized Fields   
 
 

 
m_Self 
 
javax.swing.JDialog  m_Self  
 
 the dialog itself.
 
 
 
 
 
 
m_Popup 
 
javax.swing.JPopupMenu  m_Popup  
 
 the popup to display again.
 
 
 
 
 
 
m_Capabilities 
 
 Capabilities   m_Capabilities  
 
 the capabilities used for initializing the dialog.
 
 
 
 
 
 
m_InfoLabel 
 
javax.swing.JLabel  m_InfoLabel  
 
 the label, listing the name of the superclass.
 
 
 
 
 
 
m_List 
 
 CheckBoxList   m_List  
 
 the list with all the capabilities.
 
 
 
 
 
 
m_OkButton 
 
javax.swing.JButton  m_OkButton  
 
 the OK button.
 
 
 
 
 
 
m_CancelButton 
 
javax.swing.JButton  m_CancelButton  
 
 the Cancel button.
 
 
 
 

 
   
 
 
  
 Class  weka.gui.GenericObjectEditor.GOEPanel  extends javax.swing.JPanel implements Serializable   
 
 

 
 serialVersionUID:  3656028520876011335L

 
   
 
 
  
 Serialized Fields   
 
 

 
m_ChildPropertySheet 
 
 PropertySheetPanel   m_ChildPropertySheet  
 
 The component that performs classifier customization.
 
 
 
 
 
 
m_ClassNameLabel 
 
javax.swing.JLabel  m_ClassNameLabel  
 
 The name of the current class.
 
 
 
 
 
 
m_OpenBut 
 
javax.swing.JButton  m_OpenBut  
 
 Open object from disk.
 
 
 
 
 
 
m_SaveBut 
 
javax.swing.JButton  m_SaveBut  
 
 Save object to disk.
 
 
 
 
 
 
m_okBut 
 
javax.swing.JButton  m_okBut  
 
 ok button.
 
 
 
 
 
 
m_cancelBut 
 
javax.swing.JButton  m_cancelBut  
 
 cancel button.
 
 
 
 
 
 
m_FileChooser 
 
javax.swing.JFileChooser  m_FileChooser  
 
 The filechooser for opening and saving object files.
 
 
 
 

 
   
 
 
  
 Class  weka.gui.GenericObjectEditor.GOETreeNode  extends javax.swing.tree.DefaultMutableTreeNode implements Serializable   
 
 

 
 serialVersionUID:  -1707872446682150133L

 
   
 
 
  
 Serialized Fields   
 
 

 
m_Capabilities 
 
 Capabilities   m_Capabilities  
 
 the Capabilities object to use for filtering.
 
 
 
 

 
   
 
 
  
 Class  weka.gui.GenericObjectEditor.JTreePopupMenu  extends javax.swing.JPopupMenu implements Serializable   
 
 

 
 serialVersionUID:  -3404546329655057387L

 
   
 
 
  
 Serialized Fields   
 
 

 
m_Self 
 
javax.swing.JPopupMenu  m_Self  
 
 the popup itself.
 
 
 
 
 
 
m_tree 
 
javax.swing.JTree  m_tree  
 
 The tree.
 
 
 
 
 
 
m_scroller 
 
javax.swing.JScrollPane  m_scroller  
 
 The scroller.
 
 
 
 
 
 
m_FilterButton 
 
javax.swing.JButton  m_FilterButton  
 
 The filter button in case of CapabilitiesHandlers.
 
 
 
 
 
 
m_RemoveFilterButton 
 
javax.swing.JButton  m_RemoveFilterButton  
 
 The remove filter button in case of CapabilitiesHandlers.
 
 
 
 
 
 
m_CloseButton 
 
javax.swing.JButton  m_CloseButton  
 
 The button for closing the popup again.
 
 
 
 

 
   
 
 
  
 Class  weka.gui.GUIChooser  extends javax.swing.JFrame implements Serializable   
 
 

 
 serialVersionUID:  9001529425230247914L

 
   
 
 
  
 Serialized Fields   
 
 

 
m_Self 
 
 GUIChooser   m_Self  
 
 the GUIChooser itself
 
 
 
 
 
 
m_jMenuBar 
 
javax.swing.JMenuBar  m_jMenuBar  
 
 
 
 
 
 
m_jMenuProgram 
 
javax.swing.JMenu  m_jMenuProgram  
 
 
 
 
 
 
m_jMenuVisualization 
 
javax.swing.JMenu  m_jMenuVisualization  
 
 
 
 
 
 
m_jMenuTools 
 
javax.swing.JMenu  m_jMenuTools  
 
 
 
 
 
 
m_jMenuHelp 
 
javax.swing.JMenu  m_jMenuHelp  
 
 
 
 
 
 
m_PanelApplications 
 
javax.swing.JPanel  m_PanelApplications  
 
 the panel for the application buttons
 
 
 
 
 
 
m_ExplorerBut 
 
javax.swing.JButton  m_ExplorerBut  
 
 Click to open the Explorer
 
 
 
 
 
 
m_ExplorerFrame 
 
javax.swing.JFrame  m_ExplorerFrame  
 
 The frame containing the explorer interface
 
 
 
 
 
 
m_ExperimenterBut 
 
javax.swing.JButton  m_ExperimenterBut  
 
 Click to open the Explorer
 
 
 
 
 
 
m_ExperimenterFrame 
 
javax.swing.JFrame  m_ExperimenterFrame  
 
 The frame containing the experiment interface
 
 
 
 
 
 
m_KnowledgeFlowBut 
 
javax.swing.JButton  m_KnowledgeFlowBut  
 
 Click to open the KnowledgeFlow
 
 
 
 
 
 
m_pendingKnowledgeFlowLoad 
 
java.lang.String  m_pendingKnowledgeFlowLoad  
 
 Pending file to load on startup of the KnowledgeFlow
 
 
 
 
 
 
m_KnowledgeFlowFrame 
 
javax.swing.JFrame  m_KnowledgeFlowFrame  
 
 The frame containing the knowledge flow interface
 
 
 
 
 
 
m_SimpleBut 
 
javax.swing.JButton  m_SimpleBut  
 
 Click to open the simplecli
 
 
 
 
 
 
m_SimpleCLI 
 
 SimpleCLI   m_SimpleCLI  
 
 The SimpleCLI
 
 
 
 
 
 
m_ArffViewers 
 
java.util.Vector<E>  m_ArffViewers  
 
 keeps track of the opened ArffViewer instancs
 
 
 
 
 
 
m_SqlViewerFrame 
 
javax.swing.JFrame  m_SqlViewerFrame  
 
 The frame containing the SqlViewer
 
 
 
 
 
 
m_BayesNetGUIFrame 
 
javax.swing.JFrame  m_BayesNetGUIFrame  
 
 The frame containing the Bayes net GUI
 
 
 
 
 
 
m_EnsembleLibraryFrame 
 
javax.swing.JFrame  m_EnsembleLibraryFrame  
 
 The frame containing the ensemble library interface
 
 
 
 
 
 
m_Plots 
 
java.util.Vector<E>  m_Plots  
 
 keeps track of the opened plots
 
 
 
 
 
 
m_ROCs 
 
java.util.Vector<E>  m_ROCs  
 
 keeps track of the opened ROCs
 
 
 
 
 
 
m_TreeVisualizers 
 
java.util.Vector<E>  m_TreeVisualizers  
 
 keeps track of the opened tree visualizer instancs
 
 
 
 
 
 
m_GraphVisualizers 
 
java.util.Vector<E>  m_GraphVisualizers  
 
 keeps track of the opened graph visualizer instancs
 
 
 
 
 
 
m_BoundaryVisualizerFrame 
 
javax.swing.JFrame  m_BoundaryVisualizerFrame  
 
 The frame containing the boundary visualizer
 
 
 
 
 
 
m_SystemInfoFrame 
 
javax.swing.JFrame  m_SystemInfoFrame  
 
 The frame containing the system info
 
 
 
 
 
 
m_MemoryUsageFrame 
 
javax.swing.JFrame  m_MemoryUsageFrame  
 
 The frame containing the memory usage
 
 
 
 
 
 
m_weka 
 
java.awt.Image  m_weka  
 
 The weka image
 
 
 
 
 
 
m_FileChooserTreeVisualizer 
 
javax.swing.JFileChooser  m_FileChooserTreeVisualizer  
 
 filechooser for the TreeVisualizer
 
 
 
 
 
 
m_FileChooserGraphVisualizer 
 
javax.swing.JFileChooser  m_FileChooserGraphVisualizer  
 
 filechooser for the GraphVisualizer
 
 
 
 
 
 
m_FileChooserPlot 
 
javax.swing.JFileChooser  m_FileChooserPlot  
 
 filechooser for Plots
 
 
 
 
 
 
m_FileChooserROC 
 
javax.swing.JFileChooser  m_FileChooserROC  
 
 filechooser for ROC curves
 
 
 
 
 
 
m_Icon 
 
java.awt.Image  m_Icon  
 
 the icon for the frames
 
 
 
 
 
 
m_ChildFrames 
 
java.util.HashSet<E>  m_ChildFrames  
 
 contains the child frames (title <-> object).
 
 
 
 

 
   
 
 
  
 Class  weka.gui.GUIChooser.ChildFrameSDI  extends javax.swing.JFrame implements Serializable   
 
 

 
 serialVersionUID:  8588293938686425618L

 
   
 
 
  
 Serialized Fields   
 
 

 
m_Parent 
 
 GUIChooser   m_Parent  
 
 the parent frame.
 
 
 
 

 
   
 
 
  
 Class  weka.gui.HierarchyPropertyParser  extends java.lang.Object implements Serializable   
 
 

 
 serialVersionUID:  -4151103338506077544L

 
   
 
 
  
 Serialized Fields   
 
 

 
m_Root 
 
weka.gui.HierarchyPropertyParser.TreeNode  m_Root  
 
 Keep track of the root of the tree
 
 
 
 
 
 
m_Current 
 
weka.gui.HierarchyPropertyParser.TreeNode  m_Current  
 
 Keep track of the current node when traversing the tree
 
 
 
 
 
 
m_Seperator 
 
java.lang.String  m_Seperator  
 
 The level separate in the path
 
 
 
 
 
 
m_Depth 
 
int  m_Depth  
 
 The depth of the tree
 
 
 
 

 
   
 
 
  
 Class  weka.gui.InstancesSummaryPanel  extends javax.swing.JPanel implements Serializable   
 
 

 
 serialVersionUID:  -5243579535296681063L

 
   
 
 
  
 Serialized Fields   
 
 

 
m_RelationNameLab 
 
javax.swing.JLabel  m_RelationNameLab  
 
 Displays the name of the relation
 
 
 
 
 
 
m_NumInstancesLab 
 
javax.swing.JLabel  m_NumInstancesLab  
 
 Displays the number of instances
 
 
 
 
 
 
m_NumAttributesLab 
 
javax.swing.JLabel  m_NumAttributesLab  
 
 Displays the number of attributes
 
 
 
 
 
 
m_Instances 
 
 Instances   m_Instances  
 
 The instances we're playing with
 
 
 
 

 
   
 
 
  
 Class  weka.gui.ListSelectorDialog  extends javax.swing.JDialog implements Serializable   
 
 

 
 serialVersionUID:  906147926840288895L

 
   
 
 
  
 Serialized Fields   
 
 

 
m_SelectBut 
 
javax.swing.JButton  m_SelectBut  
 
 Click to choose the currently selected property
 
 
 
 
 
 
m_CancelBut 
 
javax.swing.JButton  m_CancelBut  
 
 Click to cancel the property selection
 
 
 
 
 
 
m_PatternBut 
 
javax.swing.JButton  m_PatternBut  
 
 Click to enter a regex pattern for selection
 
 
 
 
 
 
m_List 
 
javax.swing.JList  m_List  
 
 The list component
 
 
 
 
 
 
m_Result 
 
int  m_Result  
 
 Whether the selection was made or cancelled
 
 
 
 
 
 
m_PatternRegEx 
 
java.lang.String  m_PatternRegEx  
 
 The current regular expression.
 
 
 
 

 
   
 
 
  
 Class  weka.gui.LogPanel  extends javax.swing.JPanel implements Serializable   
 
 

 
 serialVersionUID:  -4072464549112439484L

 
   
 
 
  
 Serialized Fields   
 
 

 
m_StatusLab 
 
javax.swing.JLabel  m_StatusLab  
 
 Displays the current status
 
 
 
 
 
 
m_LogText 
 
javax.swing.JTextArea  m_LogText  
 
 Displays the log messages
 
 
 
 
 
 
m_logButton 
 
javax.swing.JButton  m_logButton  
 
 The button for viewing the log
 
 
 
 
 
 
m_First 
 
boolean  m_First  
 
 An indicator for whether text has been output yet
 
 
 
 
 
 
m_TaskMonitor 
 
 WekaTaskMonitor   m_TaskMonitor  
 
 The panel for monitoring the number of running tasks (if supplied)
 
 
 
 

 
   
 
 
  
 Class  weka.gui.LogWindow  extends javax.swing.JFrame implements Serializable   
 
 

 
 serialVersionUID:  5650947361381061112L

 
   
 
 
  
 Serialized Fields   
 
 

 
m_UseWordwrap 
 
boolean  m_UseWordwrap  
 
 whether the JTextPane has wordwrap or not
 
 
 
 
 
 
m_Output 
 
javax.swing.JTextPane  m_Output  
 
 the output
 
 
 
 
 
 
m_ButtonClear 
 
javax.swing.JButton  m_ButtonClear  
 
 the clear button
 
 
 
 
 
 
m_ButtonClose 
 
javax.swing.JButton  m_ButtonClose  
 
 the close button
 
 
 
 
 
 
m_LabelCurrentSize 
 
javax.swing.JLabel  m_LabelCurrentSize  
 
 the current size
 
 
 
 
 
 
m_SpinnerMaxSize 
 
javax.swing.JSpinner  m_SpinnerMaxSize  
 
 the spinner for the max number of chars
 
 
 
 
 
 
m_CheckBoxWordwrap 
 
javax.swing.JCheckBox  m_CheckBoxWordwrap  
 
 whether to allow wordwrap or not
 
 
 
 

 
   
 
 
  
 Class  weka.gui.Main  extends javax.swing.JFrame implements Serializable   
 
 

 
 serialVersionUID:  1453813254824253849L

 
   
 
 
  
 Serialized Fields   
 
 

 
m_Self 
 
 Main   m_Self  
 
 the frame itself.
 
 
 
 
 
 
m_GUIType 
 
int  m_GUIType  
 
 the type of GUI to display.
 
 
 
 
 
 
m_ChildFrames 
 
java.util.HashSet<E>  m_ChildFrames  
 
 contains the child frames (title <-> object).
 
 
 
 
 
 
m_FileChooserTreeVisualizer 
 
javax.swing.JFileChooser  m_FileChooserTreeVisualizer  
 
 filechooser for the TreeVisualizer.
 
 
 
 
 
 
m_FileChooserGraphVisualizer 
 
javax.swing.JFileChooser  m_FileChooserGraphVisualizer  
 
 filechooser for the GraphVisualizer.
 
 
 
 
 
 
m_FileChooserPlot 
 
javax.swing.JFileChooser  m_FileChooserPlot  
 
 filechooser for Plots.
 
 
 
 
 
 
m_FileChooserROC 
 
javax.swing.JFileChooser  m_FileChooserROC  
 
 filechooser for ROC curves.
 
 
 
 
 
 
jMenuHelp 
 
javax.swing.JMenu  jMenuHelp  
 
 
 
 
 
 
jMenuVisualization 
 
javax.swing.JMenu  jMenuVisualization  
 
 
 
 
 
 
jMenuTools 
 
javax.swing.JMenu  jMenuTools  
 
 
 
 
 
 
jDesktopPane 
 
javax.swing.JDesktopPane  jDesktopPane  
 
 
 
 
 
 
jMenuApplications 
 
javax.swing.JMenu  jMenuApplications  
 
 
 
 
 
 
jMenuItemHelpSystemInfo 
 
javax.swing.JMenuItem  jMenuItemHelpSystemInfo  
 
 
 
 
 
 
jMenuItemHelpAbout 
 
javax.swing.JMenuItem  jMenuItemHelpAbout  
 
 
 
 
 
 
jMenuItemHelpHomepage 
 
javax.swing.JMenuItem  jMenuItemHelpHomepage  
 
 
 
 
 
 
jMenuItemHelpWekaWiki 
 
javax.swing.JMenuItem  jMenuItemHelpWekaWiki  
 
 
 
 
 
 
jMenuItemHelpWekaDoc 
 
javax.swing.JMenuItem  jMenuItemHelpWekaDoc  
 
 
 
 
 
 
jMenuItemHelpSourceforge 
 
javax.swing.JMenuItem  jMenuItemHelpSourceforge  
 
 
 
 
 
 
jMenuItemVisualizationBoundaryVisualizer 
 
javax.swing.JMenuItem  jMenuItemVisualizationBoundaryVisualizer  
 
 
 
 
 
 
jMenuItemVisualizationGraphVisualizer 
 
javax.swing.JMenuItem  jMenuItemVisualizationGraphVisualizer  
 
 
 
 
 
 
jMenuItemVisualizationTreeVisualizer 
 
javax.swing.JMenuItem  jMenuItemVisualizationTreeVisualizer  
 
 
 
 
 
 
jMenuItemVisualizationROC 
 
javax.swing.JMenuItem  jMenuItemVisualizationROC  
 
 
 
 
 
 
jMenuItemVisualizationPlot 
 
javax.swing.JMenuItem  jMenuItemVisualizationPlot  
 
 
 
 
 
 
jMenuItemToolsEnsembleLibrary 
 
javax.swing.JMenuItem  jMenuItemToolsEnsembleLibrary  
 
 
 
 
 
 
jMenuItemToolsSqlViewer 
 
javax.swing.JMenuItem  jMenuItemToolsSqlViewer  
 
 
 
 
 
 
jMenuItemToolsArffViewer 
 
javax.swing.JMenuItem  jMenuItemToolsArffViewer  
 
 
 
 
 
 
jMenuItemApplicationsSimpleCLI 
 
javax.swing.JMenuItem  jMenuItemApplicationsSimpleCLI  
 
 
 
 
 
 
jMenuItemApplicationsKnowledgeFlow 
 
javax.swing.JMenuItem  jMenuItemApplicationsKnowledgeFlow  
 
 
 
 
 
 
jMenuItemApplicationsExperimenter 
 
javax.swing.JMenuItem  jMenuItemApplicationsExperimenter  
 
 
 
 
 
 
jMenuItemApplicationsExplorer 
 
javax.swing.JMenuItem  jMenuItemApplicationsExplorer  
 
 
 
 
 
 
jMenuItemProgramExit 
 
javax.swing.JMenuItem  jMenuItemProgramExit  
 
 
 
 
 
 
jMenuItemProgramLogWindow 
 
javax.swing.JMenuItem  jMenuItemProgramLogWindow  
 
 
 
 
 
 
jMenuItemProgramMemoryUsage 
 
javax.swing.JMenuItem  jMenuItemProgramMemoryUsage  
 
 
 
 
 
 
jMenuItemProgramPreferences 
 
javax.swing.JMenuItem  jMenuItemProgramPreferences  
 
 
 
 
 
 
jMenuProgram 
 
javax.swing.JMenu  jMenuProgram  
 
 
 
 
 
 
jMenuExtensions 
 
javax.swing.JMenu  jMenuExtensions  
 
 
 
 
 
 
jMenuWindows 
 
javax.swing.JMenu  jMenuWindows  
 
 
 
 
 
 
jMenuBar 
 
javax.swing.JMenuBar  jMenuBar  
 
 
 
 

 
   
 
 
  
 Class  weka.gui.Main.BackgroundDesktopPane  extends javax.swing.JDesktopPane implements Serializable   
 
 

 
 serialVersionUID:  2046713123452402745L

 
   
 
 
  
 Serialized Fields   
 
 

 
m_Background 
 
java.awt.Image  m_Background  
 
 the actual background image.
 
 
 
 

 
   
 
 
  
 Class  weka.gui.Main.ChildFrameMDI  extends javax.swing.JInternalFrame implements Serializable   
 
 

 
 serialVersionUID:  3772573515346899959L

 
   
 
 
  
 Serialized Fields   
 
 

 
m_Parent 
 
 Main   m_Parent  
 
 the parent frame.
 
 
 
 

 
   
 
 
  
 Class  weka.gui.Main.ChildFrameSDI  extends javax.swing.JFrame implements Serializable   
 
 

 
 serialVersionUID:  8588293938686425618L

 
   
 
 
  
 Serialized Fields   
 
 

 
m_Parent 
 
 Main   m_Parent  
 
 the parent frame.
 
 
 
 

 
   
 
 
  
 Class  weka.gui.MemoryUsagePanel  extends javax.swing.JPanel implements Serializable   
 
 

 
 serialVersionUID:  -4812319791687471721L

 
   
 
 
  
 Serialized Fields   
 
 

 
m_History 
 
java.util.Vector<E>  m_History  
 
 the memory usage over time.
 
 
 
 
 
 
m_Memory 
 
 Memory   m_Memory  
 
 for monitoring the memory usage.
 
 
 
 
 
 
m_Monitor 
 
weka.gui.MemoryUsagePanel.MemoryMonitor  m_Monitor  
 
 the thread for monitoring the memory usage.
 
 
 
 
 
 
m_ButtonGC 
 
javax.swing.JButton  m_ButtonGC  
 
 the button for running the garbage collector.
 
 
 
 
 
 
m_Percentages 
 
java.util.Vector<E>  m_Percentages  
 
 the threshold percentages to change color.
 
 
 
 
 
 
m_Colors 
 
java.util.Hashtable<K,V>  m_Colors  
 
 the corresponding colors for the thresholds.
 
 
 
 
 
 
m_DefaultColor 
 
java.awt.Color  m_DefaultColor  
 
 the default color.
 
 
 
 
 
 
m_BackgroundColor 
 
java.awt.Color  m_BackgroundColor  
 
 the background color.
 
 
 
 
 
 
m_FrameLocation 
 
java.awt.Point  m_FrameLocation  
 
 the position for the dialog.
 
 
 
 

 
   
 
 
  
 Class  weka.gui.PropertyDialog  extends javax.swing.JDialog implements Serializable   
 
 

 
 serialVersionUID:  -2314850859392433539L

 
   
 
 
  
 Serialized Fields   
 
 

 
m_Editor 
 
java.beans.PropertyEditor  m_Editor  
 
 The property editor.
 
 
 
 
 
 
m_EditorComponent 
 
java.awt.Component  m_EditorComponent  
 
 The custom editor component.
 
 
 
 

 
   
 
 
  
 Class  weka.gui.PropertyPanel  extends javax.swing.JPanel implements Serializable   
 
 

 
 serialVersionUID:  5370025273466728904L

 
   
 
 
  
 Serialized Fields   
 
 

 
m_Editor 
 
java.beans.PropertyEditor  m_Editor  
 
 The property editor
 
 
 
 
 
 
m_PD 
 
 PropertyDialog   m_PD  
 
 The currently displayed property dialog, if any
 
 
 
 
 
 
m_HasCustomPanel 
 
boolean  m_HasCustomPanel  
 
 Whether the editor has provided its own panel
 
 
 
 
 
 
m_CustomPanel 
 
javax.swing.JPanel  m_CustomPanel  
 
 The custom panel (if any)
 
 
 
 

 
   
 
 
  
 Class  weka.gui.PropertySelectorDialog  extends javax.swing.JDialog implements Serializable   
 
 

 
 serialVersionUID:  -3155058124137930518L

 
   
 
 
  
 Serialized Fields   
 
 

 
m_SelectBut 
 
javax.swing.JButton  m_SelectBut  
 
 Click to choose the currently selected property
 
 
 
 
 
 
m_CancelBut 
 
javax.swing.JButton  m_CancelBut  
 
 Click to cancel the property selection
 
 
 
 
 
 
m_Root 
 
javax.swing.tree.DefaultMutableTreeNode  m_Root  
 
 The root of the property tree
 
 
 
 
 
 
m_RootObject 
 
java.lang.Object  m_RootObject  
 
 The object at the root of the tree
 
 
 
 
 
 
m_Result 
 
int  m_Result  
 
 Whether the selection was made or cancelled
 
 
 
 
 
 
m_ResultPath 
 
java.lang.Object[]  m_ResultPath  
 
 Stores the path to the selected property
 
 
 
 
 
 
m_Tree 
 
javax.swing.JTree  m_Tree  
 
 The component displaying the property tree
 
 
 
 

 
   
 
 
  
 Class  weka.gui.PropertySheetPanel  extends javax.swing.JPanel implements Serializable   
 
 

 
 serialVersionUID:  -8939835593429918345L

 
   
 
 
  
 Serialized Fields   
 
 

 
m_Target 
 
java.lang.Object  m_Target  
 
 The target object being edited.
 
 
 
 
 
 
m_Properties 
 
java.beans.PropertyDescriptor[]  m_Properties  
 
 Holds properties of the target.
 
 
 
 
 
 
m_Methods 
 
java.beans.MethodDescriptor[]  m_Methods  
 
 Holds the methods of the target.
 
 
 
 
 
 
m_Editors 
 
java.beans.PropertyEditor[]  m_Editors  
 
 Holds property editors of the object.
 
 
 
 
 
 
m_Values 
 
java.lang.Object[]  m_Values  
 
 Holds current object values for each property.
 
 
 
 
 
 
m_Views 
 
javax.swing.JComponent[]  m_Views  
 
 Stores GUI components containing each editing component.
 
 
 
 
 
 
m_Labels 
 
javax.swing.JLabel[]  m_Labels  
 
 The labels for each property.
 
 
 
 
 
 
m_TipTexts 
 
java.lang.String[]  m_TipTexts  
 
 The tool tip text for each property.
 
 
 
 
 
 
m_HelpText 
 
java.lang.StringBuffer  m_HelpText  
 
 StringBuffer containing help text for the object being edited.
 
 
 
 
 
 
m_HelpDialog 
 
javax.swing.JDialog  m_HelpDialog  
 
 Help dialog.
 
 
 
 
 
 
m_CapabilitiesDialog 
 
weka.gui.PropertySheetPanel.CapabilitiesHelpDialog  m_CapabilitiesDialog  
 
 Capabilities Help dialog.
 
 
 
 
 
 
m_HelpBut 
 
javax.swing.JButton  m_HelpBut  
 
 Button to pop up the full help text in a separate dialog.
 
 
 
 
 
 
m_CapabilitiesBut 
 
javax.swing.JButton  m_CapabilitiesBut  
 
 Button to pop up the capabilities in a separate dialog.
 
 
 
 
 
 
m_CapabilitiesText 
 
javax.swing.JTextArea  m_CapabilitiesText  
 
 the TextArea of the Capabilities help dialog.
 
 
 
 
 
 
m_NumEditable 
 
int  m_NumEditable  
 
 A count of the number of properties we have an editor for.
 
 
 
 
 
 
m_aboutPanel 
 
javax.swing.JPanel  m_aboutPanel  
 
 The panel holding global info and help, if provided by
      the object being editied.
 
 
 
 
 
 
support 
 
java.beans.PropertyChangeSupport  support  
 
 A support object for handling property change listeners.
 
 
 
 

 
   
 
 
  
 Class weka.gui.PropertySheetPanel.CapabilitiesHelpDialog extends javax.swing.JDialog implements Serializable   
 
 

 
 serialVersionUID:  -1404770987103289858L

 
   
 
 
  
 Serialized Fields   
 
 

 
m_Self 
 
weka.gui.PropertySheetPanel.CapabilitiesHelpDialog  m_Self  
 
 the dialog itself.
 
 
 
 

 
   
 
 
  
 Class  weka.gui.ResultHistoryPanel  extends javax.swing.JPanel implements Serializable   
 
 

 
 serialVersionUID:  4297069440135326829L

 
   
 
 
  
 Serialized Fields   
 
 

 
m_SingleText 
 
javax.swing.text.JTextComponent  m_SingleText  
 
 An optional component for single-click display
 
 
 
 
 
 
m_SingleName 
 
java.lang.String  m_SingleName  
 
 The named result being viewed in the single-click display
 
 
 
 
 
 
m_Model 
 
javax.swing.DefaultListModel  m_Model  
 
 The list model
 
 
 
 
 
 
m_List 
 
javax.swing.JList  m_List  
 
 The list component
 
 
 
 
 
 
m_Results 
 
java.util.Hashtable<K,V>  m_Results  
 
 A Hashtable mapping names to result buffers
 
 
 
 
 
 
m_FramedOutput 
 
java.util.Hashtable<K,V>  m_FramedOutput  
 
 A Hashtable mapping names to output text components
 
 
 
 
 
 
m_Objs 
 
java.util.Hashtable<K,V>  m_Objs  
 
 A hashtable mapping names to arbitrary objects
 
 
 
 
 
 
m_HandleRightClicks 
 
boolean  m_HandleRightClicks  
 
 Let the result history list handle right clicks in the default
      manner---ie, pop up a window displaying the buffer
 
 
 
 
 
 
m_Printer 
 
 PrintableComponent   m_Printer  
 
 for printing the output to files
 
 
 
 

 
   
 
 
  
 Class  weka.gui.ResultHistoryPanel.RKeyAdapter  extends java.awt.event.KeyAdapter implements Serializable   
 
 

 
 serialVersionUID:  -8675332541861828079L

 

 
   
 
 
  
 Class  weka.gui.ResultHistoryPanel.RMouseAdapter  extends java.awt.event.MouseAdapter implements Serializable   
 
 

 
 serialVersionUID:  -8991922650552358669L

 

 
   
 
 
  
 Class  weka.gui.SetInstancesPanel  extends javax.swing.JPanel implements Serializable   
 
 

 
 serialVersionUID:  -384804041420453735L

 
   
 
 
  
 Serialized Fields   
 
 

 
m_OpenFileBut 
 
javax.swing.JButton  m_OpenFileBut  
 
 Click to open instances from a file
 
 
 
 
 
 
m_OpenURLBut 
 
javax.swing.JButton  m_OpenURLBut  
 
 Click to open instances from a URL
 
 
 
 
 
 
m_CloseBut 
 
javax.swing.JButton  m_CloseBut  
 
 Click to close the dialog
 
 
 
 
 
 
m_Summary 
 
 InstancesSummaryPanel   m_Summary  
 
 The instance summary component
 
 
 
 
 
 
m_FileChooser 
 
 ConverterFileChooser   m_FileChooser  
 
 The file chooser for selecting arff files
 
 
 
 
 
 
m_LastURL 
 
java.lang.String  m_LastURL  
 
 Stores the last URL that instances were loaded from
 
 
 
 
 
 
m_IOThread 
 
java.lang.Thread  m_IOThread  
 
 The thread we do loading in
 
 
 
 
 
 
m_Support 
 
java.beans.PropertyChangeSupport  m_Support  
 
 Manages sending notifications to people when we change the set of
 working instances.
 
 
 
 
 
 
m_Instances 
 
 Instances   m_Instances  
 
 The current set of instances loaded
 
 
 
 
 
 
m_Loader 
 
 Loader   m_Loader  
 
 The current loader used to obtain the current instances
 
 
 
 
 
 
m_ParentFrame 
 
javax.swing.JFrame  m_ParentFrame  
 
 the parent frame. if one is provided, the close-button is displayed
 
 
 
 
 
 
m_CloseButPanel 
 
javax.swing.JPanel  m_CloseButPanel  
 
 the panel the Close-Button is located in
 
 
 
 
 
 
m_readIncrementally 
 
boolean  m_readIncrementally  
 
 
 
 

 
   
 
 
  
 Class  weka.gui.SimpleCLI  extends java.awt.Frame implements Serializable   
 
 

 
 serialVersionUID:  -50661410800566036L

 

 
   
 
 
  
 Class  weka.gui.SimpleCLIPanel  extends javax.swing.JPanel implements Serializable   
 
 

 
 serialVersionUID:  -7377739469759943231L

 
   
 
 
  
 Serialized Fields   
 
 

 
m_OutputArea 
 
javax.swing.JTextArea  m_OutputArea  
 
 The output area canvas added to the frame.
 
 
 
 
 
 
m_Input 
 
javax.swing.JTextField  m_Input  
 
 The command input area.
 
 
 
 
 
 
m_CommandHistory 
 
java.util.Vector<E>  m_CommandHistory  
 
 The history of commands entered interactively.
 
 
 
 
 
 
m_HistoryPos 
 
int  m_HistoryPos  
 
 The current position in the command history.
 
 
 
 
 
 
m_POO 
 
java.io.PipedOutputStream  m_POO  
 
 The new output stream for System.out.
 
 
 
 
 
 
m_POE 
 
java.io.PipedOutputStream  m_POE  
 
 The new output stream for System.err.
 
 
 
 
 
 
m_OutRedirector 
 
java.lang.Thread  m_OutRedirector  
 
 The thread that sends output from m_POO to the output box.
 
 
 
 
 
 
m_ErrRedirector 
 
java.lang.Thread  m_ErrRedirector  
 
 The thread that sends output from m_POE to the output box.
 
 
 
 
 
 
m_RunThread 
 
java.lang.Thread  m_RunThread  
 
 The thread currently running a class main method.
 
 
 
 
 
 
m_Completion 
 
 SimpleCLIPanel.CommandlineCompletion   m_Completion  
 
 The commandline completion.
 
 
 
 

 
   
 
 
  
 Class  weka.gui.SortedTableModel  extends javax.swing.table.AbstractTableModel implements Serializable   
 
 

 
 serialVersionUID:  4030907921461127548L

 
   
 
 
  
 Serialized Fields   
 
 

 
mModel 
 
javax.swing.table.TableModel  mModel  
 
 the actual table model
 
 
 
 
 
 
mIndices 
 
int[]  mIndices  
 
 the mapping between displayed and actual index
 
 
 
 
 
 
mSortColumn 
 
int  mSortColumn  
 
 the sort column
 
 
 
 
 
 
mAscending 
 
boolean  mAscending  
 
 whether sorting is ascending or descending
 
 
 
 

 
   
 
 
  
 Class  weka.gui.SplashWindow  extends java.awt.Window implements Serializable   
 
 

 
 serialVersionUID:  -2685134277041307795L

 
   
 
 
  
 Serialized Fields   
 
 

 
image 
 
java.awt.Image  image  
 
 The splash image which is displayed on the splash window.
 
 
 
 
 
 
paintCalled 
 
boolean  paintCalled  
 
 This attribute indicates whether the method
 paint(Graphics) has been called at least once since the
 construction of this window. 
 This attribute is used to notify method splash(Image)
 that the window has been drawn at least once
 by the AWT event dispatcher thread. 
 This attribute acts like a latch. Once set to true,
 it will never be changed back to false again.
 
 
  See Also:    SplashWindow.paint(java.awt.Graphics)  , 
  SplashWindow.splash(java.awt.Image)   
 

 
   
 
 
  
 Class  weka.gui.ViewerDialog  extends javax.swing.JDialog implements Serializable   
 
 

 
 serialVersionUID:  6747718484736047752L

 
   
 
 
  
 Serialized Fields   
 
 

 
m_Result 
 
int  m_Result  
 
 the result of the user's action, either OK or CANCEL
 
 
 
 
 
 
m_OkButton 
 
javax.swing.JButton  m_OkButton  
 
 Click to activate the current set parameters
 
 
 
 
 
 
m_CancelButton 
 
javax.swing.JButton  m_CancelButton  
 
 Click to cancel the dialog
 
 
 
 
 
 
m_UndoButton 
 
javax.swing.JButton  m_UndoButton  
 
 Click to undo the last action
 
 
 
 
 
 
m_ArffPanel 
 
 ArffPanel   m_ArffPanel  
 
 the panel to display the Instances-object
 
 
 
 

 
   
 
 
  
 Class  weka.gui.WekaTaskMonitor  extends javax.swing.JPanel implements Serializable   
 
 

 
 serialVersionUID:  508309816292197578L

 
   
 
 
  
 Serialized Fields   
 
 

 
m_ActiveTasks 
 
int  m_ActiveTasks  
 
 The number of running weka threads
 
 
 
 
 
 
m_MonitorLabel 
 
javax.swing.JLabel  m_MonitorLabel  
 
 The label for displaying info
 
 
 
 
 
 
m_iconStationary 
 
javax.swing.ImageIcon  m_iconStationary  
 
 The icon for the stationary bird
 
 
 
 
 
 
m_iconAnimated 
 
javax.swing.ImageIcon  m_iconAnimated  
 
 The icon for the animated bird
 
 
 
 
 
 
m_animating 
 
boolean  m_animating  
 
 True if their are active tasks
 
 
 
 
 

 
 
  
 Package   weka.gui.arffviewer   
 
 

 
   
 
 
  
 Class  weka.gui.arffviewer.ArffPanel  extends javax.swing.JPanel implements Serializable   
 
 

 
 serialVersionUID:  -4697041150989513939L

 
   
 
 
  
 Serialized Fields   
 
 

 
m_TableArff 
 
 ArffTable   m_TableArff  
 
 the underlying table
 
 
 
 
 
 
m_PopupHeader 
 
javax.swing.JPopupMenu  m_PopupHeader  
 
 the popup menu for the header row
 
 
 
 
 
 
m_PopupRows 
 
javax.swing.JPopupMenu  m_PopupRows  
 
 the popup menu for the data rows
 
 
 
 
 
 
m_LabelName 
 
javax.swing.JLabel  m_LabelName  
 
 displays the relation name
 
 
 
 
 
 
menuItemMean 
 
javax.swing.JMenuItem  menuItemMean  
 
 
 
 
 
 
menuItemSetAllValues 
 
javax.swing.JMenuItem  menuItemSetAllValues  
 
 
 
 
 
 
menuItemSetMissingValues 
 
javax.swing.JMenuItem  menuItemSetMissingValues  
 
 
 
 
 
 
menuItemReplaceValues 
 
javax.swing.JMenuItem  menuItemReplaceValues  
 
 
 
 
 
 
menuItemRenameAttribute 
 
javax.swing.JMenuItem  menuItemRenameAttribute  
 
 
 
 
 
 
menuItemAttributeAsClass 
 
javax.swing.JMenuItem  menuItemAttributeAsClass  
 
 
 
 
 
 
menuItemDeleteAttribute 
 
javax.swing.JMenuItem  menuItemDeleteAttribute  
 
 
 
 
 
 
menuItemDeleteAttributes 
 
javax.swing.JMenuItem  menuItemDeleteAttributes  
 
 
 
 
 
 
menuItemSortInstances 
 
javax.swing.JMenuItem  menuItemSortInstances  
 
 
 
 
 
 
menuItemDeleteSelectedInstance 
 
javax.swing.JMenuItem  menuItemDeleteSelectedInstance  
 
 
 
 
 
 
menuItemDeleteAllSelectedInstances 
 
javax.swing.JMenuItem  menuItemDeleteAllSelectedInstances  
 
 
 
 
 
 
menuItemSearch 
 
javax.swing.JMenuItem  menuItemSearch  
 
 
 
 
 
 
menuItemClearSearch 
 
javax.swing.JMenuItem  menuItemClearSearch  
 
 
 
 
 
 
menuItemUndo 
 
javax.swing.JMenuItem  menuItemUndo  
 
 
 
 
 
 
menuItemCopy 
 
javax.swing.JMenuItem  menuItemCopy  
 
 
 
 
 
 
menuItemOptimalColWidth 
 
javax.swing.JMenuItem  menuItemOptimalColWidth  
 
 
 
 
 
 
menuItemOptimalColWidths 
 
javax.swing.JMenuItem  menuItemOptimalColWidths  
 
 
 
 
 
 
m_Filename 
 
java.lang.String  m_Filename  
 
 the filename used in the title
 
 
 
 
 
 
m_Title 
 
java.lang.String  m_Title  
 
 the title prefix
 
 
 
 
 
 
m_CurrentCol 
 
int  m_CurrentCol  
 
 the currently selected column
 
 
 
 
 
 
m_Changed 
 
boolean  m_Changed  
 
 flag for whether data got changed
 
 
 
 
 
 
m_ChangeListeners 
 
java.util.HashSet<E>  m_ChangeListeners  
 
 the listeners that listen for modifications
 
 
 
 
 
 
m_LastSearch 
 
java.lang.String  m_LastSearch  
 
 the string used in the last search
 
 
 
 
 
 
m_LastReplace 
 
java.lang.String  m_LastReplace  
 
 the string used in the last replace
 
 
 
 

 
   
 
 
  
 Class  weka.gui.arffviewer.ArffSortedTableModel  extends  SortedTableModel  implements Serializable   
 
 

 
 serialVersionUID:  -5733148376354254030L

 

 
   
 
 
  
 Class  weka.gui.arffviewer.ArffTable  extends javax.swing.JTable implements Serializable   
 
 

 
 serialVersionUID:  -2016200506908637967L

 
   
 
 
  
 Serialized Fields   
 
 

 
m_SearchString 
 
java.lang.String  m_SearchString  
 
 the search string
 
 
 
 
 
 
m_ChangeListeners 
 
java.util.HashSet<E>  m_ChangeListeners  
 
 the listeners for changes
 
 
 
 

 
   
 
 
  
 Class weka.gui.arffviewer.ArffTable.RelationalCellEditor extends javax.swing.AbstractCellEditor implements Serializable   
 
 

 
 serialVersionUID:  657969163293205963L

 
   
 
 
  
 Serialized Fields   
 
 

 
m_Button 
 
javax.swing.JButton  m_Button  
 
 the button for opening the dialog
 
 
 
 
 
 
m_CurrentInst 
 
 Instances   m_CurrentInst  
 
 the current instances
 
 
 
 
 
 
m_RowIndex 
 
int  m_RowIndex  
 
 the row index this editor is for
 
 
 
 
 
 
m_ColumnIndex 
 
int  m_ColumnIndex  
 
 the column index this editor is for
 
 
 
 

 
   
 
 
  
 Class  weka.gui.arffviewer.ArffTableCellRenderer  extends javax.swing.table.DefaultTableCellRenderer implements Serializable   
 
 

 
 serialVersionUID:  9195794493301191171L

 
   
 
 
  
 Serialized Fields   
 
 

 
missingColor 
 
java.awt.Color  missingColor  
 
 the color for missing values
 
 
 
 
 
 
missingColorSelected 
 
java.awt.Color  missingColorSelected  
 
 the color for selected missing values
 
 
 
 
 
 
highlightColor 
 
java.awt.Color  highlightColor  
 
 the color for highlighted values
 
 
 
 
 
 
highlightColorSelected 
 
java.awt.Color  highlightColorSelected  
 
 the color for selected highlighted values
 
 
 
 

 
   
 
 
  
 Class  weka.gui.arffviewer.ArffViewer  extends javax.swing.JFrame implements Serializable   
 
 

 
 serialVersionUID:  -7455845566922685175L

 
   
 
 
  
 Serialized Fields   
 
 

 
m_MainPanel 
 
 ArffViewerMainPanel   m_MainPanel  
 
 the main panel
 
 
 
 

 
   
 
 
  
 Class  weka.gui.arffviewer.ArffViewerMainPanel  extends javax.swing.JPanel implements Serializable   
 
 

 
 serialVersionUID:  -8763161167586738753L

 
   
 
 
  
 Serialized Fields   
 
 

 
parent 
 
java.awt.Container  parent  
 
 
 
 
 
 
tabbedPane 
 
javax.swing.JTabbedPane  tabbedPane  
 
 
 
 
 
 
menuBar 
 
javax.swing.JMenuBar  menuBar  
 
 
 
 
 
 
menuFile 
 
javax.swing.JMenu  menuFile  
 
 
 
 
 
 
menuFileOpen 
 
javax.swing.JMenuItem  menuFileOpen  
 
 
 
 
 
 
menuFileSave 
 
javax.swing.JMenuItem  menuFileSave  
 
 
 
 
 
 
menuFileSaveAs 
 
javax.swing.JMenuItem  menuFileSaveAs  
 
 
 
 
 
 
menuFileClose 
 
javax.swing.JMenuItem  menuFileClose  
 
 
 
 
 
 
menuFileCloseAll 
 
javax.swing.JMenuItem  menuFileCloseAll  
 
 
 
 
 
 
menuFileProperties 
 
javax.swing.JMenuItem  menuFileProperties  
 
 
 
 
 
 
menuFileExit 
 
javax.swing.JMenuItem  menuFileExit  
 
 
 
 
 
 
menuEdit 
 
javax.swing.JMenu  menuEdit  
 
 
 
 
 
 
menuEditUndo 
 
javax.swing.JMenuItem  menuEditUndo  
 
 
 
 
 
 
menuEditCopy 
 
javax.swing.JMenuItem  menuEditCopy  
 
 
 
 
 
 
menuEditSearch 
 
javax.swing.JMenuItem  menuEditSearch  
 
 
 
 
 
 
menuEditClearSearch 
 
javax.swing.JMenuItem  menuEditClearSearch  
 
 
 
 
 
 
menuEditDeleteAttribute 
 
javax.swing.JMenuItem  menuEditDeleteAttribute  
 
 
 
 
 
 
menuEditDeleteAttributes 
 
javax.swing.JMenuItem  menuEditDeleteAttributes  
 
 
 
 
 
 
menuEditRenameAttribute 
 
javax.swing.JMenuItem  menuEditRenameAttribute  
 
 
 
 
 
 
menuEditAttributeAsClass 
 
javax.swing.JMenuItem  menuEditAttributeAsClass  
 
 
 
 
 
 
menuEditDeleteInstance 
 
javax.swing.JMenuItem  menuEditDeleteInstance  
 
 
 
 
 
 
menuEditDeleteInstances 
 
javax.swing.JMenuItem  menuEditDeleteInstances  
 
 
 
 
 
 
menuEditSortInstances 
 
javax.swing.JMenuItem  menuEditSortInstances  
 
 
 
 
 
 
menuView 
 
javax.swing.JMenu  menuView  
 
 
 
 
 
 
menuViewAttributes 
 
javax.swing.JMenuItem  menuViewAttributes  
 
 
 
 
 
 
menuViewValues 
 
javax.swing.JMenuItem  menuViewValues  
 
 
 
 
 
 
menuViewOptimalColWidths 
 
javax.swing.JMenuItem  menuViewOptimalColWidths  
 
 
 
 
 
 
fileChooser 
 
 ConverterFileChooser   fileChooser  
 
 
 
 
 
 
frameTitle 
 
java.lang.String  frameTitle  
 
 
 
 
 
 
confirmExit 
 
boolean  confirmExit  
 
 
 
 
 
 
width 
 
int  width  
 
 
 
 
 
 
height 
 
int  height  
 
 
 
 
 
 
top 
 
int  top  
 
 
 
 
 
 
left 
 
int  left  
 
 
 
 
 
 
exitOnClose 
 
boolean  exitOnClose  
 
 
 
 
 

 
 
  
 Package   weka.gui.beans   
 
 

 
   
 
 
  
 Class  weka.gui.beans.AbstractDataSink  extends javax.swing.JPanel implements Serializable   
 
 

 
 serialVersionUID:  3956528599473814287L

 
   
 
 
  
 Serialized Fields   
 
 

 
m_visual 
 
 BeanVisual   m_visual  
 
 Default visual for data sources
 
 
 
 
 
 
m_listenee 
 
java.lang.Object  m_listenee  
 
 Non null if this object is a target for any events.
 Provides for the simplest case when only one incomming connection
 is allowed. Subclasses can overide the appropriate BeanCommon methods
 to change this behaviour and allow multiple connections if desired
 
 
 
 

 
   
 
 
  
 Class  weka.gui.beans.AbstractDataSource  extends javax.swing.JPanel implements Serializable   
 
 

 
 serialVersionUID:  -4127257701890044793L

 
   
 
 
  
 Serialized Fields   
 
 

 
m_design 
 
boolean  m_design  
 
 True if this bean's appearance is the design mode appearance
 
 
 
 
 
 
m_bcSupport 
 
java.beans.beancontext.BeanContextChildSupport  m_bcSupport  
 
 BeanContextChild support
 
 
 
 
 
 
m_visual 
 
 BeanVisual   m_visual  
 
 Default visual for data sources
 
 
 
 
 
 
m_listeners 
 
java.util.Vector<E>  m_listeners  
 
 Objects listening for events from data sources
 
 
 
 

 
   
 
 
  
 Class  weka.gui.beans.AbstractEvaluator  extends javax.swing.JPanel implements Serializable   
 
 

 
 serialVersionUID:  3983303541814121632L

 
   
 
 
  
 Serialized Fields   
 
 

 
m_visual 
 
 BeanVisual   m_visual  
 
 Default visual for evaluators
 
 
 
 
 
 
m_listenee 
 
java.lang.Object  m_listenee  
 
 
 
 

 
   
 
 
  
 Class  weka.gui.beans.AbstractTestSetProducer  extends javax.swing.JPanel implements Serializable   
 
 

 
 serialVersionUID:  -7905764845789349839L

 
   
 
 
  
 Serialized Fields   
 
 

 
m_listeners 
 
java.util.Vector<E>  m_listeners  
 
 Objects listening to us
 
 
 
 
 
 
m_visual 
 
 BeanVisual   m_visual  
 
 
 
 
 
 
m_listenee 
 
java.lang.Object  m_listenee  
 
 non null if this object is a target for any events.
 
 
 
 

 
   
 
 
  
 Class  weka.gui.beans.AbstractTrainAndTestSetProducer  extends javax.swing.JPanel implements Serializable   
 
 

 
 serialVersionUID:  -1809339823613492037L

 
   
 
 
  
 Serialized Fields   
 
 

 
m_trainingListeners 
 
java.util.Vector<E>  m_trainingListeners  
 
 Objects listening for trainin set events
 
 
 
 
 
 
m_testListeners 
 
java.util.Vector<E>  m_testListeners  
 
 Objects listening for test set events
 
 
 
 
 
 
m_visual 
 
 BeanVisual   m_visual  
 
 
 
 
 
 
m_listenee 
 
java.lang.Object  m_listenee  
 
 non null if this object is a target for any events.
 
 
 
 

 
   
 
 
  
 Class  weka.gui.beans.AbstractTrainingSetProducer  extends javax.swing.JPanel implements Serializable   
 
 

 
 serialVersionUID:  -7842746199524591125L

 
   
 
 
  
 Serialized Fields   
 
 

 
m_listeners 
 
java.util.Vector<E>  m_listeners  
 
 Objects listening for training set events
 
 
 
 
 
 
m_visual 
 
 BeanVisual   m_visual  
 
 
 
 
 
 
m_listenee 
 
java.lang.Object  m_listenee  
 
 non null if this object is a target for any events.
 
 
 
 

 
   
 
 
  
 Class  weka.gui.beans.Associator  extends javax.swing.JPanel implements Serializable   
 
 

 
 serialVersionUID:  -7843500322130210057L

 
   
 
 
  
 Serialized Fields   
 
 

 
m_visual 
 
 BeanVisual   m_visual  
 
 
 
 
 
 
m_state 
 
int  m_state  
 
 
 
 
 
 
m_buildThread 
 
java.lang.Thread  m_buildThread  
 
 
 
 
 
 
m_globalInfo 
 
java.lang.String  m_globalInfo  
 
 Global info for the wrapped associator (if it exists).
 
 
 
 
 
 
m_listenees 
 
java.util.Hashtable<K,V>  m_listenees  
 
 Objects talking to us
 
 
 
 
 
 
m_textListeners 
 
java.util.Vector<E>  m_textListeners  
 
 Objects listening for text events
 
 
 
 
 
 
m_graphListeners 
 
java.util.Vector<E>  m_graphListeners  
 
 Objects listening for graph events
 
 
 
 
 
 
m_Associator 
 
 Associator   m_Associator  
 
 
 
 

 
   
 
 
  
 Class  weka.gui.beans.AssociatorCustomizer  extends javax.swing.JPanel implements Serializable   
 
 

 
 serialVersionUID:  5767664969353495974L

 
   
 
 
  
 Serialized Fields   
 
 

 
m_pcSupport 
 
java.beans.PropertyChangeSupport  m_pcSupport  
 
 
 
 
 
 
m_dsAssociator 
 
 Associator   m_dsAssociator  
 
 
 
 
 
 
m_AssociatorEditor 
 
 PropertySheetPanel   m_AssociatorEditor  
 
 
 
 
 
 
m_parentFrame 
 
javax.swing.JFrame  m_parentFrame  
 
 
 
 
 
 
m_backup 
 
 Associator   m_backup  
 
 Backup is user presses cancel
 
 
 
 

 
   
 
 
  
 Class  weka.gui.beans.AttributeSummarizer  extends  DataVisualizer  implements Serializable   
 
 

 
 serialVersionUID:  -294354961169372758L

 
   
 
 
  
 Serialized Fields   
 
 

 
m_gridWidth 
 
int  m_gridWidth  
 
 The number of plots horizontally in the display
 
 
 
 
 
 
m_maxPlots 
 
int  m_maxPlots  
 
 The maximum number of plots to show
 
 
 
 
 
 
m_coloringIndex 
 
int  m_coloringIndex  
 
 Index on which to color the plots.
 
 
 
 

 
   
 
 
  
 Class  weka.gui.beans.BatchClassifierEvent  extends java.util.EventObject implements Serializable   
 
 

 
 serialVersionUID:  878097199815991084L

 
   
 
 
  
 Serialized Fields   
 
 

 
m_classifier 
 
 Classifier   m_classifier  
 
 The classifier
 
 
 
 
 
 
m_testSet 
 
 DataSetEvent   m_testSet  
 
 Instances that can be used for testing the classifier
 
 
 
 
 
 
m_trainSet 
 
 DataSetEvent   m_trainSet  
 
 Instances that were used to train the classifier (may be null if not available)
 
 
 
 
 
 
m_runNumber 
 
int  m_runNumber  
 
 The run number that this classifier was generated for
 
 
 
 
 
 
m_maxRunNumber 
 
int  m_maxRunNumber  
 
 The maximum number of runs
 
 
 
 
 
 
m_setNumber 
 
int  m_setNumber  
 
 The set number for the test set
 
 
 
 
 
 
m_maxSetNumber 
 
int  m_maxSetNumber  
 
 The last set number for this series
 
 
 
 
 
 
m_groupIdentifier 
 
long  m_groupIdentifier  
 
 An identifier that can be used to group all related runs/sets
 together.
 
 
 
 

 
   
 
 
  
 Class  weka.gui.beans.BatchClustererEvent  extends java.util.EventObject implements Serializable   
 
 

 
 serialVersionUID:  7268777944939129714L

 
   
 
 
  
 Serialized Fields   
 
 

 
m_clusterer 
 
 Clusterer   m_clusterer  
 
 The clusterer
 
 
 
 
 
 
m_testSet 
 
 DataSetEvent   m_testSet  
 
 Training or Test Instances
 
 
 
 
 
 
m_setNumber 
 
int  m_setNumber  
 
 The set number for the test set
 
 
 
 
 
 
m_testOrTrain 
 
int  m_testOrTrain  
 
 Indicates if m_testSet is a training or a test set. 0 for test, >0 for training
 
 
 
 
 
 
m_maxSetNumber 
 
int  m_maxSetNumber  
 
 The last set number for this series
 
 
 
 

 
   
 
 
  
 Class  weka.gui.beans.BeanConnection  extends java.lang.Object implements Serializable   
 
 

 
 serialVersionUID:  8804264241791332064L

 
   
 
 
  
 Serialized Fields   
 
 

 
m_source 
 
 BeanInstance   m_source  
 
 
 
 
 
 
m_target 
 
 BeanInstance   m_target  
 
 
 
 
 
 
m_eventName 
 
java.lang.String  m_eventName  
 
 The name of the event for this connection
 
 
 
 
 
 
m_hidden 
 
boolean  m_hidden  
 
 
 
 

 
   
 
 
  
 Class  weka.gui.beans.BeanInstance  extends java.lang.Object implements Serializable   
 
 

 
 serialVersionUID:  -7575653109025406342L

 
   
 
 
  
 Serialized Fields   
 
 

 
m_bean 
 
java.lang.Object  m_bean  
 
 Holds the bean encapsulated in this instance
 
 
 
 
 
 
m_x 
 
int  m_x  
 
 
 
 
 
 
m_y 
 
int  m_y  
 
 
 
 

 
   
 
 
  
 Class  weka.gui.beans.BeanVisual  extends javax.swing.JPanel implements Serializable   
 
 

 
 serialVersionUID:  -6677473561687129614L

 
   
 
 
  
 Serialization Methods   
 
 

 
    
readObject 
 
private void  readObject (java.io.ObjectInputStream ois)
                 throws java.io.IOException,
                        java.lang.ClassNotFoundException 
 
 Overides default read object in order to reload icons.
 This is necessary because for some strange reason animated
 gifs stop being animated after being serialized/deserialized.
 
  

  Throws: 
  java.io.IOException  - if an error occurs
  java.lang.ClassNotFoundException  - if an error occurs 
 
 
   
 
 
  
 Serialized Fields   
 
 

 
m_iconPath 
 
java.lang.String  m_iconPath  
 
 Holds name (including path) of the static icon
 
 
 
 
 
 
m_animatedIconPath 
 
java.lang.String  m_animatedIconPath  
 
 Holds name (including path) of the animated icon
 
 
 
 
 
 
m_visualName 
 
java.lang.String  m_visualName  
 
 Name for the bean
 
 
 
 
 
 
m_visualLabel 
 
javax.swing.JLabel  m_visualLabel  
 
 
 
 
 
 
m_stationary 
 
boolean  m_stationary  
 
 Container for the icon
 
 
 
 
 
 
m_pcs 
 
java.beans.PropertyChangeSupport  m_pcs  
 
 
 
 
 
 
m_displayConnectors 
 
boolean  m_displayConnectors  
 
 
 
 
 
 
m_connectorColor 
 
java.awt.Color  m_connectorColor  
 
 
 
 

 
   
 
 
  
 Class  weka.gui.beans.ChartEvent  extends java.util.EventObject implements Serializable   
 
 

 
 serialVersionUID:  7812460715499569390L

 
   
 
 
  
 Serialized Fields   
 
 

 
m_legendText 
 
java.util.Vector<E>  m_legendText  
 
 
 
 
 
 
m_max 
 
double  m_max  
 
 
 
 
 
 
m_min 
 
double  m_min  
 
 
 
 
 
 
m_reset 
 
boolean  m_reset  
 
 
 
 
 
 
m_dataPoint 
 
double[]  m_dataPoint  
 
 Y values of the data points
 
 
 
 

 
   
 
 
  
 Class  weka.gui.beans.ClassAssigner  extends javax.swing.JPanel implements Serializable   
 
 

 
 serialVersionUID:  4011131665025817924L

 
   
 
 
  
 Serialized Fields   
 
 

 
m_classColumn 
 
java.lang.String  m_classColumn  
 
 
 
 
 
 
m_connectedFormat 
 
 Instances   m_connectedFormat  
 
 format of instances for current incoming connection (if any)
 
 
 
 
 
 
m_trainingProvider 
 
java.lang.Object  m_trainingProvider  
 
 
 
 
 
 
m_testProvider 
 
java.lang.Object  m_testProvider  
 
 
 
 
 
 
m_dataProvider 
 
java.lang.Object  m_dataProvider  
 
 
 
 
 
 
m_instanceProvider 
 
java.lang.Object  m_instanceProvider  
 
 
 
 
 
 
m_trainingListeners 
 
java.util.Vector<E>  m_trainingListeners  
 
 
 
 
 
 
m_testListeners 
 
java.util.Vector<E>  m_testListeners  
 
 
 
 
 
 
m_dataListeners 
 
java.util.Vector<E>  m_dataListeners  
 
 
 
 
 
 
m_instanceListeners 
 
java.util.Vector<E>  m_instanceListeners  
 
 
 
 
 
 
m_dataFormatListeners 
 
java.util.Vector<E>  m_dataFormatListeners  
 
 
 
 
 
 
m_visual 
 
 BeanVisual   m_visual  
 
 
 
 

 
   
 
 
  
 Class  weka.gui.beans.ClassAssignerCustomizer  extends javax.swing.JPanel implements Serializable   
 
 

 
 serialVersionUID:  476539385765301907L

 
   
 
 
  
 Serialized Fields   
 
 

 
m_displayColNames 
 
boolean  m_displayColNames  
 
 
 
 
 
 
m_classAssigner 
 
 ClassAssigner   m_classAssigner  
 
 
 
 
 
 
m_pcSupport 
 
java.beans.PropertyChangeSupport  m_pcSupport  
 
 
 
 
 
 
m_caEditor 
 
 PropertySheetPanel   m_caEditor  
 
 
 
 
 
 
m_ClassCombo 
 
javax.swing.JComboBox  m_ClassCombo  
 
 
 
 
 
 
m_holderP 
 
javax.swing.JPanel  m_holderP  
 
 
 
 

 
   
 
 
  
 Class  weka.gui.beans.Classifier  extends javax.swing.JPanel implements Serializable   
 
 

 
 serialVersionUID:  659603893917736008L

 
   
 
 
  
 Serialized Fields   
 
 

 
m_visual 
 
 BeanVisual   m_visual  
 
 
 
 
 
 
m_state 
 
int  m_state  
 
 
 
 
 
 
m_globalInfo 
 
java.lang.String  m_globalInfo  
 
 Global info for the wrapped classifier (if it exists).
 
 
 
 
 
 
m_listenees 
 
java.util.Hashtable<K,V>  m_listenees  
 
 Objects talking to us
 
 
 
 
 
 
m_batchClassifierListeners 
 
java.util.Vector<E>  m_batchClassifierListeners  
 
 Objects listening for batch classifier events
 
 
 
 
 
 
m_incrementalClassifierListeners 
 
java.util.Vector<E>  m_incrementalClassifierListeners  
 
 Objects listening for incremental classifier events
 
 
 
 
 
 
m_graphListeners 
 
java.util.Vector<E>  m_graphListeners  
 
 Objects listening for graph events
 
 
 
 
 
 
m_textListeners 
 
java.util.Vector<E>  m_textListeners  
 
 Objects listening for text events
 
 
 
 
 
 
m_trainingSet 
 
 Instances   m_trainingSet  
 
 Holds training instances for batch training. Not transient because
 header is retained for validating any instance events that this
 classifier might be asked to predict in the future.
 
 
 
 
 
 
m_Classifier 
 
 Classifier   m_Classifier  
 
 
 
 
 
 
m_ClassifierTemplate 
 
 Classifier   m_ClassifierTemplate  
 
 Template used for creating copies when building in parallel
 
 
 
 
 
 
m_ie 
 
 IncrementalClassifierEvent   m_ie  
 
 
 
 
 
 
m_binaryFilter 
 
javax.swing.filechooser.FileFilter  m_binaryFilter  
 
 
 
 
 
 
m_KOMLFilter 
 
javax.swing.filechooser.FileFilter  m_KOMLFilter  
 
 
 
 
 
 
m_XStreamFilter 
 
javax.swing.filechooser.FileFilter  m_XStreamFilter  
 
 
 
 
 
 
m_updateIncrementalClassifier 
 
boolean  m_updateIncrementalClassifier  
 
 If the classifier is an incremental classifier, should we
 update it (ie train it on incoming instances). This makes it
 possible incrementally test on a separate stream of instances
 without updating the classifier, or mix batch training/testing
 with incremental training/testing
 
 
 
 
 
 
m_incrementalEvent 
 
 InstanceEvent   m_incrementalEvent  
 
 Event to handle when processing incremental updates
 
 
 
 
 
 
m_executionSlots 
 
int  m_executionSlots  
 
 Number of threads to use to train models with
 
 
 
 
 
 
m_oldText 
 
java.lang.String  m_oldText  
 
 Holds original icon label text
 
 
 
 
 
 
m_block 
 
boolean  m_block  
 
 true if we should block any further training data sets.
 
 
 
 

 
   
 
 
  
 Class weka.gui.beans.Classifier.TrainingTask extends java.lang.Object implements Serializable   
 
 

 
   
 
 
  
 Serialized Fields   
 
 

 
m_runNum 
 
int  m_runNum  
 
 
 
 
 
 
m_maxRunNum 
 
int  m_maxRunNum  
 
 
 
 
 
 
m_setNum 
 
int  m_setNum  
 
 
 
 
 
 
m_maxSetNum 
 
int  m_maxSetNum  
 
 
 
 
 
 
m_train 
 
 Instances   m_train  
 
 
 
 
 
 
m_taskInfo 
 
 TaskStatusInfo   m_taskInfo  
 
 
 
 

 
   
 
 
  
 Class  weka.gui.beans.ClassifierCustomizer  extends javax.swing.JPanel implements Serializable   
 
 

 
 serialVersionUID:  -6688000820160821429L

 
   
 
 
  
 Serialized Fields   
 
 

 
m_pcSupport 
 
java.beans.PropertyChangeSupport  m_pcSupport  
 
 
 
 
 
 
m_dsClassifier 
 
 Classifier   m_dsClassifier  
 
 
 
 
 
 
m_ClassifierEditor 
 
 PropertySheetPanel   m_ClassifierEditor  
 
 
 
 
 
 
m_incrementalPanel 
 
javax.swing.JPanel  m_incrementalPanel  
 
 
 
 
 
 
m_updateIncrementalClassifier 
 
javax.swing.JCheckBox  m_updateIncrementalClassifier  
 
 
 
 
 
 
m_panelVisible 
 
boolean  m_panelVisible  
 
 
 
 
 
 
m_holderPanel 
 
javax.swing.JPanel  m_holderPanel  
 
 
 
 
 
 
m_executionSlotsText 
 
javax.swing.JTextField  m_executionSlotsText  
 
 
 
 
 
 
m_parentFrame 
 
javax.swing.JFrame  m_parentFrame  
 
 
 
 
 
 
m_backup 
 
 Classifier   m_backup  
 
 Copy of the current classifier in case cancel is selected
 
 
 
 

 
   
 
 
  
 Class  weka.gui.beans.ClassifierPerformanceEvaluator  extends  AbstractEvaluator  implements Serializable   
 
 

 
 serialVersionUID:  -3511801418192148690L

 
   
 
 
  
 Serialized Fields   
 
 

 
m_textListeners 
 
java.util.Vector<E>  m_textListeners  
 
 
 
 
 
 
m_thresholdListeners 
 
java.util.Vector<E>  m_thresholdListeners  
 
 
 
 
 
 
m_visualizableErrorListeners 
 
java.util.Vector<E>  m_visualizableErrorListeners  
 
 
 
 
 
 
m_rocListenersConnected 
 
boolean  m_rocListenersConnected  
 
 
 
 

 
   
 
 
  
 Class  weka.gui.beans.ClassValuePicker  extends javax.swing.JPanel implements Serializable   
 
 

 
 serialVersionUID:  -1196143276710882989L

 
   
 
 
  
 Serialized Fields   
 
 

 
m_classValue 
 
java.lang.String  m_classValue  
 
 the class value considered to be the positive class
 
 
 
 
 
 
m_connectedFormat 
 
 Instances   m_connectedFormat  
 
 format of instances for the current incoming connection (if any)
 
 
 
 
 
 
m_dataProvider 
 
java.lang.Object  m_dataProvider  
 
 
 
 
 
 
m_dataListeners 
 
java.util.Vector<E>  m_dataListeners  
 
 
 
 
 
 
m_dataFormatListeners 
 
java.util.Vector<E>  m_dataFormatListeners  
 
 
 
 
 
 
m_visual 
 
 BeanVisual   m_visual  
 
 
 
 

 
   
 
 
  
 Class  weka.gui.beans.ClassValuePickerCustomizer  extends javax.swing.JPanel implements Serializable   
 
 

 
 serialVersionUID:  8213423053861600469L

 
   
 
 
  
 Serialized Fields   
 
 

 
m_displayValNames 
 
boolean  m_displayValNames  
 
 
 
 
 
 
m_classValuePicker 
 
 ClassValuePicker   m_classValuePicker  
 
 
 
 
 
 
m_pcSupport 
 
java.beans.PropertyChangeSupport  m_pcSupport  
 
 
 
 
 
 
m_ClassValueCombo 
 
javax.swing.JComboBox  m_ClassValueCombo  
 
 
 
 
 
 
m_holderP 
 
javax.swing.JPanel  m_holderP  
 
 
 
 
 
 
m_messageLabel 
 
javax.swing.JLabel  m_messageLabel  
 
 
 
 
 
 
m_parent 
 
javax.swing.JFrame  m_parent  
 
 
 
 
 
 
m_backup 
 
java.lang.String  m_backup  
 
 
 
 
 
 
m_textBoxEntryMode 
 
boolean  m_textBoxEntryMode  
 
 
 
 
 
 
m_valueTextBox 
 
javax.swing.JTextField  m_valueTextBox  
 
 
 
 

 
   
 
 
  
 Class  weka.gui.beans.Clusterer  extends javax.swing.JPanel implements Serializable   
 
 

 
 serialVersionUID:  7729795159836843810L

 
   
 
 
  
 Serialized Fields   
 
 

 
m_visual 
 
 BeanVisual   m_visual  
 
 
 
 
 
 
m_state 
 
int  m_state  
 
 
 
 
 
 
m_buildThread 
 
java.lang.Thread  m_buildThread  
 
 
 
 
 
 
m_globalInfo 
 
java.lang.String  m_globalInfo  
 
 Global info for the wrapped classifier (if it exists).
 
 
 
 
 
 
m_listenees 
 
java.util.Hashtable<K,V>  m_listenees  
 
 Objects talking to us
 
 
 
 
 
 
m_batchClustererListeners 
 
java.util.Vector<E>  m_batchClustererListeners  
 
 Objects listening for batch clusterer events
 
 
 
 
 
 
m_graphListeners 
 
java.util.Vector<E>  m_graphListeners  
 
 Objects listening for graph events
 
 
 
 
 
 
m_textListeners 
 
java.util.Vector<E>  m_textListeners  
 
 Objects listening for text events
 
 
 
 
 
 
m_trainingSet 
 
 Instances   m_trainingSet  
 
 Holds training instances for batch training.
 
 
 
 
 
 
m_Clusterer 
 
 Clusterer   m_Clusterer  
 
 
 
 
 
 
m_dummy 
 
java.lang.Double  m_dummy  
 
 
 
 

 
   
 
 
  
 Class  weka.gui.beans.ClustererCustomizer  extends javax.swing.JPanel implements Serializable   
 
 

 
 serialVersionUID:  -2035688458149534161L

 
   
 
 
  
 Serialized Fields   
 
 

 
m_pcSupport 
 
java.beans.PropertyChangeSupport  m_pcSupport  
 
 
 
 
 
 
m_dsClusterer 
 
 Clusterer   m_dsClusterer  
 
 
 
 
 
 
m_ClustererEditor 
 
 PropertySheetPanel   m_ClustererEditor  
 
 
 
 
 
 
m_parentFrame 
 
javax.swing.JFrame  m_parentFrame  
 
 
 
 
 
 
m_backup 
 
 Clusterer   m_backup  
 
 Backup if the user presses cancel
 
 
 
 

 
   
 
 
  
 Class  weka.gui.beans.ClustererPerformanceEvaluator  extends  AbstractEvaluator  implements Serializable   
 
 

 
 serialVersionUID:  8041163601333978584L

 
   
 
 
  
 Serialized Fields   
 
 

 
m_textListeners 
 
java.util.Vector<E>  m_textListeners  
 
 
 
 

 
   
 
 
  
 Class  weka.gui.beans.CostBenefitAnalysis  extends javax.swing.JPanel implements Serializable   
 
 

 
 serialVersionUID:  8647471654613320469L

 
   
 
 
  
 Serialized Fields   
 
 

 
m_visual 
 
 BeanVisual   m_visual  
 
 
 
 
 
 
m_framePoppedUp 
 
boolean  m_framePoppedUp  
 
 
 
 
 
 
m_design 
 
boolean  m_design  
 
 True if this bean's appearance is the design mode appearance
 
 
 
 
 
 
m_bcSupport 
 
java.beans.beancontext.BeanContextChildSupport  m_bcSupport  
 
 BeanContextChild support
 
 
 
 
 
 
m_listenee 
 
java.lang.Object  m_listenee  
 
 The object sending us data (we allow only one connection at any one time)
 
 
 
 

 
   
 
 
  
 Class weka.gui.beans.CostBenefitAnalysis.AnalysisPanel extends javax.swing.JPanel implements Serializable   
 
 

 
 serialVersionUID:  5364871945448769003L

 
   
 
 
  
 Serialized Fields   
 
 

 
m_performancePanel 
 
 VisualizePanel   m_performancePanel  
 
 Displays the performance graphs(s)
 
 
 
 
 
 
m_costBenefitPanel 
 
 VisualizePanel   m_costBenefitPanel  
 
 Displays the cost/benefit (profit/loss) graph
 
 
 
 
 
 
m_classAttribute 
 
 Attribute   m_classAttribute  
 
 The class attribute from the data that was used to generate
 the threshold curve
 
 
 
 
 
 
m_masterPlot 
 
 PlotData2D   m_masterPlot  
 
 Data for the threshold curve
 
 
 
 
 
 
m_costBenefit 
 
 PlotData2D   m_costBenefit  
 
 Data for the cost/benefit curve
 
 
 
 
 
 
m_shapeSizes 
 
int[]  m_shapeSizes  
 
 The size of the points being plotted
 
 
 
 
 
 
m_previousShapeIndex 
 
int  m_previousShapeIndex  
 
 The index of the previous plotted point that was highlighted
 
 
 
 
 
 
m_thresholdSlider 
 
javax.swing.JSlider  m_thresholdSlider  
 
 The slider for adjusting the threshold
 
 
 
 
 
 
m_percPop 
 
javax.swing.JRadioButton  m_percPop  
 
 
 
 
 
 
m_percOfTarget 
 
javax.swing.JRadioButton  m_percOfTarget  
 
 
 
 
 
 
m_threshold 
 
javax.swing.JRadioButton  m_threshold  
 
 
 
 
 
 
m_percPopLab 
 
javax.swing.JLabel  m_percPopLab  
 
 
 
 
 
 
m_percOfTargetLab 
 
javax.swing.JLabel  m_percOfTargetLab  
 
 
 
 
 
 
m_thresholdLab 
 
javax.swing.JLabel  m_thresholdLab  
 
 
 
 
 
 
m_conf_predictedA 
 
javax.swing.JLabel  m_conf_predictedA  
 
 
 
 
 
 
m_conf_predictedB 
 
javax.swing.JLabel  m_conf_predictedB  
 
 
 
 
 
 
m_conf_actualA 
 
javax.swing.JLabel  m_conf_actualA  
 
 
 
 
 
 
m_conf_actualB 
 
javax.swing.JLabel  m_conf_actualB  
 
 
 
 
 
 
m_conf_aa 
 
weka.gui.beans.CostBenefitAnalysis.AnalysisPanel.ConfusionCell  m_conf_aa  
 
 
 
 
 
 
m_conf_ab 
 
weka.gui.beans.CostBenefitAnalysis.AnalysisPanel.ConfusionCell  m_conf_ab  
 
 
 
 
 
 
m_conf_ba 
 
weka.gui.beans.CostBenefitAnalysis.AnalysisPanel.ConfusionCell  m_conf_ba  
 
 
 
 
 
 
m_conf_bb 
 
weka.gui.beans.CostBenefitAnalysis.AnalysisPanel.ConfusionCell  m_conf_bb  
 
 
 
 
 
 
m_cost_predictedA 
 
javax.swing.JLabel  m_cost_predictedA  
 
 
 
 
 
 
m_cost_predictedB 
 
javax.swing.JLabel  m_cost_predictedB  
 
 
 
 
 
 
m_cost_actualA 
 
javax.swing.JLabel  m_cost_actualA  
 
 
 
 
 
 
m_cost_actualB 
 
javax.swing.JLabel  m_cost_actualB  
 
 
 
 
 
 
m_cost_aa 
 
javax.swing.JTextField  m_cost_aa  
 
 
 
 
 
 
m_cost_ab 
 
javax.swing.JTextField  m_cost_ab  
 
 
 
 
 
 
m_cost_ba 
 
javax.swing.JTextField  m_cost_ba  
 
 
 
 
 
 
m_cost_bb 
 
javax.swing.JTextField  m_cost_bb  
 
 
 
 
 
 
m_maximizeCB 
 
javax.swing.JButton  m_maximizeCB  
 
 
 
 
 
 
m_minimizeCB 
 
javax.swing.JButton  m_minimizeCB  
 
 
 
 
 
 
m_costR 
 
javax.swing.JRadioButton  m_costR  
 
 
 
 
 
 
m_benefitR 
 
javax.swing.JRadioButton  m_benefitR  
 
 
 
 
 
 
m_costBenefitL 
 
javax.swing.JLabel  m_costBenefitL  
 
 
 
 
 
 
m_costBenefitV 
 
javax.swing.JLabel  m_costBenefitV  
 
 
 
 
 
 
m_randomV 
 
javax.swing.JLabel  m_randomV  
 
 
 
 
 
 
m_gainV 
 
javax.swing.JLabel  m_gainV  
 
 
 
 
 
 
m_originalPopSize 
 
int  m_originalPopSize  
 
 
 
 
 
 
m_totalPopField 
 
javax.swing.JTextField  m_totalPopField  
 
 Population text field
 
 
 
 
 
 
m_totalPopPrevious 
 
int  m_totalPopPrevious  
 
 
 
 
 
 
m_classificationAccV 
 
javax.swing.JLabel  m_classificationAccV  
 
 Classification accuracy
 
 
 
 
 
 
m_tpPrevious 
 
double  m_tpPrevious  
 
 
 
 
 
 
m_fpPrevious 
 
double  m_fpPrevious  
 
 
 
 
 
 
m_tnPrevious 
 
double  m_tnPrevious  
 
 
 
 
 
 
m_fnPrevious 
 
double  m_fnPrevious  
 
 
 
 

 
   
 
 
  
 Class weka.gui.beans.CostBenefitAnalysis.AnalysisPanel.ConfusionCell extends javax.swing.JPanel implements Serializable   
 
 

 
 serialVersionUID:  6148640235434494767L

 
   
 
 
  
 Serialized Fields   
 
 

 
m_conf_cell 
 
javax.swing.JLabel  m_conf_cell  
 
 
 
 
 
 
m_conf_perc 
 
javax.swing.JLabel  m_conf_perc  
 
 
 
 
 
 
m_percentageP 
 
javax.swing.JPanel  m_percentageP  
 
 
 
 
 
 
m_percentage 
 
double  m_percentage  
 
 
 
 

 
   
 
 
  
 Class  weka.gui.beans.CrossValidationFoldMaker  extends  AbstractTrainAndTestSetProducer  implements Serializable   
 
 

 
 serialVersionUID:  -6350179298851891512L

 
   
 
 
  
 Serialized Fields   
 
 

 
m_numFolds 
 
int  m_numFolds  
 
 
 
 
 
 
m_randomSeed 
 
int  m_randomSeed  
 
 
 
 

 
   
 
 
  
 Class  weka.gui.beans.CrossValidationFoldMakerCustomizer  extends javax.swing.JPanel implements Serializable   
 
 

 
 serialVersionUID:  1229878140258668581L

 
   
 
 
  
 Serialized Fields   
 
 

 
m_pcSupport 
 
java.beans.PropertyChangeSupport  m_pcSupport  
 
 
 
 
 
 
m_cvEditor 
 
 PropertySheetPanel   m_cvEditor  
 
 
 
 

 
   
 
 
  
 Class  weka.gui.beans.DataSetEvent  extends java.util.EventObject implements Serializable   
 
 

 
 serialVersionUID:  -5111218447577318057L

 
   
 
 
  
 Serialized Fields   
 
 

 
m_dataSet 
 
 Instances   m_dataSet  
 
 
 
 
 
 
m_structureOnly 
 
boolean  m_structureOnly  
 
 
 
 

 
   
 
 
  
 Class  weka.gui.beans.DataVisualizer  extends javax.swing.JPanel implements Serializable   
 
 

 
 serialVersionUID:  1949062132560159028L

 
   
 
 
  
 Serialized Fields   
 
 

 
m_visual 
 
 BeanVisual   m_visual  
 
 
 
 
 
 
m_framePoppedUp 
 
boolean  m_framePoppedUp  
 
 
 
 
 
 
m_design 
 
boolean  m_design  
 
 True if this bean's appearance is the design mode appearance
 
 
 
 
 
 
m_visPanel 
 
 VisualizePanel   m_visPanel  
 
 
 
 
 
 
m_dataSetListeners 
 
java.util.Vector<E>  m_dataSetListeners  
 
 Objects listening for data set events
 
 
 
 
 
 
m_bcSupport 
 
java.beans.beancontext.BeanContextChildSupport  m_bcSupport  
 
 BeanContextChild support
 
 
 
 

 
   
 
 
  
 Class  weka.gui.beans.Filter  extends javax.swing.JPanel implements Serializable   
 
 

 
 serialVersionUID:  8249759470189439321L

 
   
 
 
  
 Serialized Fields   
 
 

 
m_visual 
 
 BeanVisual   m_visual  
 
 
 
 
 
 
m_state 
 
int  m_state  
 
 
 
 
 
 
m_filterThread 
 
java.lang.Thread  m_filterThread  
 
 
 
 
 
 
m_globalInfo 
 
java.lang.String  m_globalInfo  
 
 Global info for the wrapped filter (if it exists).
 
 
 
 
 
 
m_listenees 
 
java.util.Hashtable<K,V>  m_listenees  
 
 Objects talking to us
 
 
 
 
 
 
m_trainingListeners 
 
java.util.Vector<E>  m_trainingListeners  
 
 Objects listening for training set events
 
 
 
 
 
 
m_testListeners 
 
java.util.Vector<E>  m_testListeners  
 
 Objects listening for test set events
 
 
 
 
 
 
m_instanceListeners 
 
java.util.Vector<E>  m_instanceListeners  
 
 Objects listening for instance events
 
 
 
 
 
 
m_dataListeners 
 
java.util.Vector<E>  m_dataListeners  
 
 Objects listening for data set events
 
 
 
 
 
 
m_Filter 
 
 Filter   m_Filter  
 
 The filter to use.
 
 
 
 
 
 
m_ie 
 
 InstanceEvent   m_ie  
 
 Instance event object for passing on filtered instance streams
 
 
 
 
 
 
m_structurePassedOn 
 
boolean  m_structurePassedOn  
 
 
 
 

 
   
 
 
  
 Class  weka.gui.beans.FilterCustomizer  extends javax.swing.JPanel implements Serializable   
 
 

 
 serialVersionUID:  2049895469240109738L

 
   
 
 
  
 Serialized Fields   
 
 

 
m_pcSupport 
 
java.beans.PropertyChangeSupport  m_pcSupport  
 
 
 
 
 
 
m_filter 
 
 Filter   m_filter  
 
 
 
 
 
 
m_backup 
 
 Filter   m_backup  
 
 Backup if user presses cancel
 
 
 
 
 
 
m_filterEditor 
 
 PropertySheetPanel   m_filterEditor  
 
 
 
 
 
 
m_parentFrame 
 
javax.swing.JFrame  m_parentFrame  
 
 
 
 

 
   
 
 
  
 Class  weka.gui.beans.GraphEvent  extends java.util.EventObject implements Serializable   
 
 

 
 serialVersionUID:  2099494034652519986L

 
   
 
 
  
 Serialized Fields   
 
 

 
m_graphString 
 
java.lang.String  m_graphString  
 
 
 
 
 
 
m_graphTitle 
 
java.lang.String  m_graphTitle  
 
 
 
 
 
 
m_graphType 
 
int  m_graphType  
 
 
 
 

 
   
 
 
  
 Class  weka.gui.beans.GraphViewer  extends javax.swing.JPanel implements Serializable   
 
 

 
 serialVersionUID:  -5183121972114900617L

 
   
 
 
  
 Serialized Fields   
 
 

 
m_visual 
 
 BeanVisual   m_visual  
 
 
 
 
 
 
m_bcSupport 
 
java.beans.beancontext.BeanContextChildSupport  m_bcSupport  
 
 BeanContextChild support
 
 
 
 
 
 
m_design 
 
boolean  m_design  
 
 True if this bean's appearance is the design mode appearance
 
 
 
 

 
   
 
 
  
 Class  weka.gui.beans.IncrementalClassifierEvaluator  extends  AbstractEvaluator  implements Serializable   
 
 

 
 serialVersionUID:  -3105419818939541291L

 
   
 
 
  
 Serialized Fields   
 
 

 
m_listeners 
 
java.util.Vector<E>  m_listeners  
 
 
 
 
 
 
m_textListeners 
 
java.util.Vector<E>  m_textListeners  
 
 
 
 
 
 
m_dataLegend 
 
java.util.Vector<E>  m_dataLegend  
 
 
 
 
 
 
m_ce 
 
 ChartEvent   m_ce  
 
 
 
 
 
 
m_dataPoint 
 
double[]  m_dataPoint  
 
 
 
 
 
 
m_reset 
 
boolean  m_reset  
 
 
 
 
 
 
m_min 
 
double  m_min  
 
 
 
 
 
 
m_max 
 
double  m_max  
 
 
 
 
 
 
m_statusFrequency 
 
int  m_statusFrequency  
 
 
 
 
 
 
m_instanceCount 
 
int  m_instanceCount  
 
 
 
 
 
 
m_outputInfoRetrievalStats 
 
boolean  m_outputInfoRetrievalStats  
 
 
 
 

 
   
 
 
  
 Class  weka.gui.beans.IncrementalClassifierEvaluatorCustomizer  extends javax.swing.JPanel implements Serializable   
 
 

 
   
 
 
  
 Serialized Fields   
 
 

 
m_pcSupport 
 
java.beans.PropertyChangeSupport  m_pcSupport  
 
 for serialization
 
 
 
 
 
 
m_ieEditor 
 
 PropertySheetPanel   m_ieEditor  
 
 
 
 

 
   
 
 
  
 Class  weka.gui.beans.IncrementalClassifierEvent  extends java.util.EventObject implements Serializable   
 
 

 
 serialVersionUID:  28979464317643232L

 
   
 
 
  
 Serialized Fields   
 
 

 
m_structure 
 
 Instances   m_structure  
 
 
 
 
 
 
m_status 
 
int  m_status  
 
 
 
 
 
 
m_classifier 
 
 Classifier   m_classifier  
 
 
 
 
 
 
m_currentInstance 
 
 Instance   m_currentInstance  
 
 
 
 

 
   
 
 
  
 Class  weka.gui.beans.InstanceEvent  extends java.util.EventObject implements Serializable   
 
 

 
 serialVersionUID:  6104920894559423946L

 
   
 
 
  
 Serialized Fields   
 
 

 
m_structure 
 
 Instances   m_structure  
 
 
 
 
 
 
m_instance 
 
 Instance   m_instance  
 
 
 
 
 
 
m_status 
 
int  m_status  
 
 
 
 

 
   
 
 
  
 Class  weka.gui.beans.InstanceStreamToBatchMaker  extends javax.swing.JPanel implements Serializable   
 
 

 
 serialVersionUID:  -7037141087208627799L

 
   
 
 
  
 Serialized Fields   
 
 

 
m_visual 
 
 BeanVisual   m_visual  
 
 
 
 
 
 
m_listenee 
 
java.lang.Object  m_listenee  
 
 Component connected to us.
 
 
 
 
 
 
m_dataListeners 
 
java.util.ArrayList<E>  m_dataListeners  
 
 
 
 
 
 
m_batch 
 
java.util.ArrayList<E>  m_batch  
 
 Collects up the instances.
 
 
 
 
 
 
m_structure 
 
 Instances   m_structure  
 
 
 
 

 
   
 
 
  
 Class  weka.gui.beans.KnowledgeFlowApp  extends javax.swing.JPanel implements Serializable   
 
 

 
 serialVersionUID:  -7064906770289728431L

 
   
 
 
  
 Serialized Fields   
 
 

 
m_fontM 
 
java.awt.FontMetrics  m_fontM  
 
 
 
 
 
 
m_mode 
 
int  m_mode  
 
 
 
 
 
 
m_toolBarGroup 
 
javax.swing.ButtonGroup  m_toolBarGroup  
 
 Button group to manage all toolbar buttons
 
 
 
 
 
 
m_toolBarBean 
 
java.lang.Object  m_toolBarBean  
 
 Holds the selected toolbar bean
 
 
 
 
 
 
m_beanLayout 
 
weka.gui.beans.KnowledgeFlowApp.BeanLayout  m_beanLayout  
 
 The layout area
 
 
 
 
 
 
m_toolBars 
 
javax.swing.JTabbedPane  m_toolBars  
 
 Tabbed pane to hold tool bars
 
 
 
 
 
 
m_pluginsToolBar 
 
javax.swing.JToolBar  m_pluginsToolBar  
 
 Stuff relating to plugin beans
 
 
 
 
 
 
m_pluginsBoxPanel 
 
javax.swing.Box  m_pluginsBoxPanel  
 
 
 
 
 
 
m_userToolBar 
 
javax.swing.JToolBar  m_userToolBar  
 
 Stuff relating to user created meta beans
 
 
 
 
 
 
m_userBoxPanel 
 
javax.swing.Box  m_userBoxPanel  
 
 
 
 
 
 
m_userComponents 
 
java.util.Vector<E>  m_userComponents  
 
 
 
 
 
 
m_firstUserComponentOpp 
 
boolean  m_firstUserComponentOpp  
 
 
 
 
 
 
m_pointerB 
 
javax.swing.JToggleButton  m_pointerB  
 
 
 
 
 
 
m_saveB 
 
javax.swing.JButton  m_saveB  
 
 
 
 
 
 
m_loadB 
 
javax.swing.JButton  m_loadB  
 
 
 
 
 
 
m_stopB 
 
javax.swing.JButton  m_stopB  
 
 
 
 
 
 
m_helpB 
 
javax.swing.JButton  m_helpB  
 
 
 
 
 
 
m_newB 
 
javax.swing.JButton  m_newB  
 
 
 
 
 
 
m_editElement 
 
 BeanInstance   m_editElement  
 
 Reference to bean being manipulated
 
 
 
 
 
 
m_sourceEventSetDescriptor 
 
java.beans.EventSetDescriptor  m_sourceEventSetDescriptor  
 
 Event set descriptor for the bean being manipulated
 
 
 
 
 
 
m_oldX 
 
int  m_oldX  
 
 Used to record screen coordinates during move, select and connect
 operations
 
 
 
 
 
 
m_oldY 
 
int  m_oldY  
 
 Used to record screen coordinates during move, select and connect
 operations
 
 
 
 
 
 
m_startX 
 
int  m_startX  
 
 
 
 
 
 
m_startY 
 
int  m_startY  
 
 
 
 
 
 
m_FileChooser 
 
javax.swing.JFileChooser  m_FileChooser  
 
 The file chooser for selecting layout files
 
 
 
 
 
 
m_logPanel 
 
 LogPanel   m_logPanel  
 
 
 
 
 
 
m_bcSupport 
 
java.beans.beancontext.BeanContextSupport  m_bcSupport  
 
 
 
 
 
 
m_KfFilter 
 
javax.swing.filechooser.FileFilter  m_KfFilter  
 
 A filter to ensure only KnowledgeFlow files in binary format get shown in
      the chooser
 
 
 
 
 
 
m_KOMLFilter 
 
javax.swing.filechooser.FileFilter  m_KOMLFilter  
 
 A filter to ensure only KnowledgeFlow files in KOML format 
      get shown in the chooser
 
 
 
 
 
 
m_XStreamFilter 
 
javax.swing.filechooser.FileFilter  m_XStreamFilter  
 
 A filter to ensure only KnowledgeFlow files in XStream format 
      get shown in the chooser
 
 
 
 
 
 
m_XMLFilter 
 
javax.swing.filechooser.FileFilter  m_XMLFilter  
 
 A filter to ensure only KnowledgeFlow layout files in XML format get 
      shown in the chooser
 
 
 
 
 
 
m_ScrollBarIncrementLayout 
 
int  m_ScrollBarIncrementLayout  
 
 the scrollbar increment of the layout scrollpane
 
 
 
 
 
 
m_ScrollBarIncrementComponents 
 
int  m_ScrollBarIncrementComponents  
 
 the scrollbar increment of the components scrollpane
 
 
 
 
 
 
m_FlowWidth 
 
int  m_FlowWidth  
 
 the flow layout width
 
 
 
 
 
 
m_FlowHeight 
 
int  m_FlowHeight  
 
 the flow layout height
 
 
 
 
 
 
m_PreferredExtension 
 
java.lang.String  m_PreferredExtension  
 
 the preferred file extension
 
 
 
 
 
 
m_UserComponentsInXML 
 
boolean  m_UserComponentsInXML  
 
 whether to store the user components in XML or in binary format
 
 
 
 
 
 
m_flowEnvironment 
 
 Environment   m_flowEnvironment  
 
 Environment variables for the current flow
 
 
 
 
 
 
m_showFileMenu 
 
boolean  m_showFileMenu  
 
 
 
 

 
   
 
 
  
 Class weka.gui.beans.KnowledgeFlowApp.BeanLayout extends  PrintablePanel  implements Serializable   
 
 

 
 serialVersionUID:  -146377012429662757L

 

 
   
 
 
  
 Class  weka.gui.beans.Loader  extends  AbstractDataSource  implements Serializable   
 
 

 
 serialVersionUID:  1993738191961163027L

 
   
 
 
  
 Serialization Methods   
 
 

 
    
readObject 
 
private void  readObject (java.io.ObjectInputStream aStream)
                 throws java.io.IOException,
                        java.lang.ClassNotFoundException 
 
  

  Throws: 
  java.io.IOException 
  java.lang.ClassNotFoundException  
 
 
 
    
readResolve 
 
private java.lang.Object  readResolve ()
                              throws java.io.ObjectStreamException 
 
  

  Throws: 
  java.io.ObjectStreamException  
 
 
   
 
 
  
 Serialized Fields   
 
 

 
m_globalInfo 
 
java.lang.String  m_globalInfo  
 
 Global info for the wrapped loader (if it exists).
 
 
 
 
 
 
m_ioThread 
 
weka.gui.beans.Loader.LoadThread  m_ioThread  
 
 Thread for doing IO in
 
 
 
 
 
 
m_state 
 
int  m_state  
 
 
 
 
 
 
m_Loader 
 
 Loader   m_Loader  
 
 Loader
 
 
 
 
 
 
m_ie 
 
 InstanceEvent   m_ie  
 
 
 
 
 
 
m_instanceEventTargets 
 
int  m_instanceEventTargets  
 
 Keep track of how many listeners for different types of events there are.
 
 
 
 
 
 
m_dataSetEventTargets 
 
int  m_dataSetEventTargets  
 
 
 
 
 
 
m_dbSet 
 
boolean  m_dbSet  
 
 Flag indicating that a database has already been configured
 
 
 
 
 
 
m_stopped 
 
boolean  m_stopped  
 
 Asked to stop?
 
 
 
 

 
   
 
 
  
 Class  weka.gui.beans.LoaderCustomizer  extends javax.swing.JPanel implements Serializable   
 
 

 
 serialVersionUID:  6990446313118930298L

 
   
 
 
  
 Serialized Fields   
 
 

 
m_pcSupport 
 
java.beans.PropertyChangeSupport  m_pcSupport  
 
 
 
 
 
 
m_dsLoader 
 
 Loader   m_dsLoader  
 
 
 
 
 
 
m_LoaderEditor 
 
 PropertySheetPanel   m_LoaderEditor  
 
 
 
 
 
 
m_fileChooser 
 
javax.swing.JFileChooser  m_fileChooser  
 
 
 
 
 
 
m_parentFrame 
 
javax.swing.JFrame  m_parentFrame  
 
 
 
 
 
 
m_dbaseURLText 
 
javax.swing.JTextField  m_dbaseURLText  
 
 
 
 
 
 
m_userNameText 
 
javax.swing.JTextField  m_userNameText  
 
 
 
 
 
 
m_queryText 
 
javax.swing.JTextField  m_queryText  
 
 
 
 
 
 
m_keyText 
 
javax.swing.JTextField  m_keyText  
 
 
 
 
 
 
m_passwordText 
 
javax.swing.JPasswordField  m_passwordText  
 
 
 
 
 
 
m_relativeFilePath 
 
javax.swing.JCheckBox  m_relativeFilePath  
 
 
 
 

 
   
 
 
  
 Class  weka.gui.beans.LogPanel  extends javax.swing.JPanel implements Serializable   
 
 

 
   
 
 
  
 Serialized Fields   
 
 

 
m_tableIndexes 
 
java.util.HashMap<K,V>  m_tableIndexes  
 
 Holds the index (line number) in the JTable of each component
 being tracked.
 
 
 
 
 
 
m_timers 
 
java.util.HashMap<K,V>  m_timers  
 
 Holds the timers associated with each component being tracked.
 
 
 
 
 
 
m_tableModel 
 
javax.swing.table.DefaultTableModel  m_tableModel  
 
 The table model for the JTable used in the status area
 
 
 
 
 
 
m_table 
 
javax.swing.JTable  m_table  
 
 The table for the status area
 
 
 
 
 
 
m_tabs 
 
javax.swing.JTabbedPane  m_tabs  
 
 Tabbed pane to hold both the status and the log
 
 
 
 
 
 
m_logPanel 
 
 LogPanel   m_logPanel  
 
 The log panel to delegate log messages to.
 
 
 
 

 
   
 
 
  
 Class  weka.gui.beans.MetaBean  extends javax.swing.JPanel implements Serializable   
 
 

 
 serialVersionUID:  -6582768902038027077L

 
   
 
 
  
 Serialized Fields   
 
 

 
m_visual 
 
 BeanVisual   m_visual  
 
 
 
 
 
 
m_subFlow 
 
java.util.Vector<E>  m_subFlow  
 
 
 
 
 
 
m_inputs 
 
java.util.Vector<E>  m_inputs  
 
 
 
 
 
 
m_outputs 
 
java.util.Vector<E>  m_outputs  
 
 
 
 
 
 
m_associatedConnections 
 
java.util.Vector<E>  m_associatedConnections  
 
 
 
 
 
 
m_subFlowPreview 
 
javax.swing.ImageIcon  m_subFlowPreview  
 
 
 
 
 
 
m_originalCoords 
 
java.util.Vector<E>  m_originalCoords  
 
 
 
 

 
   
 
 
  
 Class  weka.gui.beans.ModelPerformanceChart  extends javax.swing.JPanel implements Serializable   
 
 

 
 serialVersionUID:  -4602034200071195924L

 
   
 
 
  
 Serialized Fields   
 
 

 
m_visual 
 
 BeanVisual   m_visual  
 
 
 
 
 
 
m_framePoppedUp 
 
boolean  m_framePoppedUp  
 
 
 
 
 
 
m_design 
 
boolean  m_design  
 
 True if this bean's appearance is the design mode appearance
 
 
 
 
 
 
m_bcSupport 
 
java.beans.beancontext.BeanContextChildSupport  m_bcSupport  
 
 BeanContextChild support
 
 
 
 

 
   
 
 
  
 Class  weka.gui.beans.PredictionAppender  extends javax.swing.JPanel implements Serializable   
 
 

 
 serialVersionUID:  -2987740065058976673L

 
   
 
 
  
 Serialized Fields   
 
 

 
m_dataSourceListeners 
 
java.util.Vector<E>  m_dataSourceListeners  
 
 Objects listenening for dataset events
 
 
 
 
 
 
m_instanceListeners 
 
java.util.Vector<E>  m_instanceListeners  
 
 Objects listening for instances events
 
 
 
 
 
 
m_trainingSetListeners 
 
java.util.Vector<E>  m_trainingSetListeners  
 
 Objects listening for training set events
 
 
 
 
 
 
m_testSetListeners 
 
java.util.Vector<E>  m_testSetListeners  
 
 Objects listening for test set events
 
 
 
 
 
 
m_listenee 
 
java.lang.Object  m_listenee  
 
 Non null if this object is a target for any events.
 
 
 
 
 
 
m_format 
 
 Instances   m_format  
 
 Format of instances to be produced.
 
 
 
 
 
 
m_visual 
 
 BeanVisual   m_visual  
 
 
 
 
 
 
m_appendProbabilities 
 
boolean  m_appendProbabilities  
 
 Append classifier's predicted probabilities (if the class is discrete
 and the classifier is a distribution classifier)
 
 
 
 
 
 
m_instanceEvent 
 
 InstanceEvent   m_instanceEvent  
 
 
 
 

 
   
 
 
  
 Class  weka.gui.beans.PredictionAppenderCustomizer  extends javax.swing.JPanel implements Serializable   
 
 

 
 serialVersionUID:  6884933202506331888L

 
   
 
 
  
 Serialized Fields   
 
 

 
m_pcSupport 
 
java.beans.PropertyChangeSupport  m_pcSupport  
 
 
 
 
 
 
m_paEditor 
 
 PropertySheetPanel   m_paEditor  
 
 
 
 

 
   
 
 
  
 Class  weka.gui.beans.Saver  extends  AbstractDataSink  implements Serializable   
 
 

 
 serialVersionUID:  5371716690308950755L

 
   
 
 
  
 Serialization Methods   
 
 

 
    
readObject 
 
private void  readObject (java.io.ObjectInputStream aStream)
                 throws java.io.IOException,
                        java.lang.ClassNotFoundException 
 
  

  Throws: 
  java.io.IOException 
  java.lang.ClassNotFoundException  
 
 
   
 
 
  
 Serialized Fields   
 
 

 
m_dataSet 
 
 Instances   m_dataSet  
 
 Holds the instances to be saved
 
 
 
 
 
 
m_structure 
 
 Instances   m_structure  
 
 Holds the structure
 
 
 
 
 
 
m_globalInfo 
 
java.lang.String  m_globalInfo  
 
 Global info for the wrapped loader (if it exists).
 
 
 
 
 
 
m_Saver 
 
 Saver   m_Saver  
 
 Saver
 
 
 
 
 
 
m_SaverTemplate 
 
 Saver   m_SaverTemplate  
 
 
 
 
 
 
m_fileName 
 
java.lang.String  m_fileName  
 
 The relation name that becomes part of the file name
 
 
 
 
 
 
m_isDBSaver 
 
boolean  m_isDBSaver  
 
 Flag indicating that instances will be saved to database. Used because structure information can only be sent after a database has been configured.
 
 
 
 
 
 
m_relationNameForFilename 
 
boolean  m_relationNameForFilename  
 
 For file-based savers - if true (default), relation name is used
 as the primary part of the filename. If false, then the prefix is
 used as the filename. Useful for preventing filenames from getting
 too long when there are many filters in a flow.
 
 
 
 
 
 
m_count 
 
int  m_count  
 
 Count for structure available messages
 
 
 
 

 
   
 
 
  
 Class  weka.gui.beans.SaverCustomizer  extends javax.swing.JPanel implements Serializable   
 
 

 
 serialVersionUID:  -4874208115942078471L

 
   
 
 
  
 Serialized Fields   
 
 

 
m_pcSupport 
 
java.beans.PropertyChangeSupport  m_pcSupport  
 
 
 
 
 
 
m_dsSaver 
 
 Saver   m_dsSaver  
 
 
 
 
 
 
m_SaverEditor 
 
 PropertySheetPanel   m_SaverEditor  
 
 
 
 
 
 
m_fileChooser 
 
javax.swing.JFileChooser  m_fileChooser  
 
 
 
 
 
 
m_parentFrame 
 
javax.swing.JFrame  m_parentFrame  
 
 
 
 
 
 
m_dbaseURLText 
 
javax.swing.JTextField  m_dbaseURLText  
 
 
 
 
 
 
m_userNameText 
 
javax.swing.JTextField  m_userNameText  
 
 
 
 
 
 
m_passwordText 
 
javax.swing.JPasswordField  m_passwordText  
 
 
 
 
 
 
m_tableText 
 
javax.swing.JTextField  m_tableText  
 
 
 
 
 
 
m_idBox 
 
javax.swing.JComboBox  m_idBox  
 
 
 
 
 
 
m_tabBox 
 
javax.swing.JComboBox  m_tabBox  
 
 
 
 
 
 
m_prefixText 
 
javax.swing.JTextField  m_prefixText  
 
 
 
 
 
 
m_relativeFilePath 
 
javax.swing.JCheckBox  m_relativeFilePath  
 
 
 
 
 
 
m_relationNameForFilename 
 
javax.swing.JCheckBox  m_relationNameForFilename  
 
 
 
 

 
   
 
 
  
 Class  weka.gui.beans.ScatterPlotMatrix  extends  DataVisualizer  implements Serializable   
 
 

 
 serialVersionUID:  -657856527563507491L

 
   
 
 
  
 Serialized Fields   
 
 

 
m_matrixPanel 
 
 MatrixPanel   m_matrixPanel  
 
 
 
 

 
   
 
 
  
 Class  weka.gui.beans.SerializedModelSaver  extends javax.swing.JPanel implements Serializable   
 
 

 
 serialVersionUID:  3956528599473814287L

 
   
 
 
  
 Serialization Methods   
 
 

 
    
readObject 
 
private void  readObject (java.io.ObjectInputStream aStream)
                 throws java.io.IOException,
                        java.lang.ClassNotFoundException 
 
  

  Throws: 
  java.io.IOException 
  java.lang.ClassNotFoundException  
 
 
   
 
 
  
 Serialized Fields   
 
 

 
m_visual 
 
 BeanVisual   m_visual  
 
 Default visual for data sources
 
 
 
 
 
 
m_listenee 
 
java.lang.Object  m_listenee  
 
 Non null if this object is a target for any events.
 Provides for the simplest case when only one incomming connection
 is allowed.
 
 
 
 
 
 
m_filenamePrefix 
 
java.lang.String  m_filenamePrefix  
 
 The prefix for the file name (model + training set info will be appended)
 
 
 
 
 
 
m_directory 
 
java.io.File  m_directory  
 
 The directory to hold the saved model(s)
 
 
 
 
 
 
m_fileFormat 
 
 Tag   m_fileFormat  
 
 File format stuff
 
 
 
 
 
 
m_useRelativePath 
 
boolean  m_useRelativePath  
 
 relative path for the directory (relative to the user.dir (startup directory))?
 
 
 
 

 
   
 
 
  
 Class  weka.gui.beans.SerializedModelSaverCustomizer  extends javax.swing.JPanel implements Serializable   
 
 

 
 serialVersionUID:  -4874208115942078471L

 
   
 
 
  
 Serialized Fields   
 
 

 
m_pcSupport 
 
java.beans.PropertyChangeSupport  m_pcSupport  
 
 
 
 
 
 
m_smSaver 
 
 SerializedModelSaver   m_smSaver  
 
 
 
 
 
 
m_SaverEditor 
 
 PropertySheetPanel   m_SaverEditor  
 
 
 
 
 
 
m_fileChooser 
 
javax.swing.JFileChooser  m_fileChooser  
 
 
 
 
 
 
m_parentFrame 
 
javax.swing.JFrame  m_parentFrame  
 
 
 
 
 
 
m_prefixText 
 
javax.swing.JTextField  m_prefixText  
 
 
 
 
 
 
m_fileFormatBox 
 
javax.swing.JComboBox  m_fileFormatBox  
 
 
 
 
 
 
m_relativeFilePath 
 
javax.swing.JCheckBox  m_relativeFilePath  
 
 
 
 

 
   
 
 
  
 Class  weka.gui.beans.StripChart  extends javax.swing.JPanel implements Serializable   
 
 

 
 serialVersionUID:  1483649041577695019L

 
   
 
 
  
 Serialization Methods   
 
 

 
    
readObject 
 
private void  readObject (java.io.ObjectInputStream ois)
                 throws java.io.IOException,
                        java.lang.ClassNotFoundException 
 
 Provide some necessary initialization after object has
 been deserialized.
 
  

  Throws: 
  java.io.IOException  - if an error occurs
  java.lang.ClassNotFoundException  - if an error occurs 
 
 
   
 
 
  
 Serialized Fields   
 
 

 
m_colorList 
 
java.awt.Color[]  m_colorList  
 
 default colours for colouring lines
 
 
 
 
 
 
m_BackgroundColor 
 
java.awt.Color  m_BackgroundColor  
 
 the background color.
 
 
 
 
 
 
m_LegendPanelBorderColor 
 
java.awt.Color  m_LegendPanelBorderColor  
 
 the color of the legend panel's border.
 
 
 
 
 
 
m_iheight 
 
int  m_iheight  
 
 Width and height of the off screen image.
 
 
 
 
 
 
m_iwidth 
 
int  m_iwidth  
 
 
 
 
 
 
m_max 
 
double  m_max  
 
 Max value for the y axis.
 
 
 
 
 
 
m_min 
 
double  m_min  
 
 Min value for the y axis.
 
 
 
 
 
 
m_yScaleUpdate 
 
boolean  m_yScaleUpdate  
 
 Scale update requested.
 
 
 
 
 
 
m_oldMax 
 
double  m_oldMax  
 
 
 
 
 
 
m_oldMin 
 
double  m_oldMin  
 
 
 
 
 
 
m_labelFont 
 
java.awt.Font  m_labelFont  
 
 
 
 
 
 
m_labelMetrics 
 
java.awt.FontMetrics  m_labelMetrics  
 
 
 
 
 
 
m_legendText 
 
java.util.Vector<E>  m_legendText  
 
 
 
 
 
 
m_scalePanel 
 
weka.gui.beans.StripChart.ScalePanel  m_scalePanel  
 
 the scale.
 
 
 
 
 
 
m_legendPanel 
 
weka.gui.beans.StripChart.LegendPanel  m_legendPanel  
 
 the legend.
 
 
 
 
 
 
m_dataList 
 
java.util.LinkedList<E>  m_dataList  
 
 Holds the data to be plotted. Entries in the list are arrays of
 y points.
 
 
 
 
 
 
m_previousY 
 
double[]  m_previousY  
 
 
 
 
 
 
m_visual 
 
 BeanVisual   m_visual  
 
 
 
 
 
 
m_listenee 
 
java.lang.Object  m_listenee  
 
 
 
 
 
 
m_xValFreq 
 
int  m_xValFreq  
 
 Print x axis labels every m_xValFreq points
 
 
 
 
 
 
m_xCount 
 
int  m_xCount  
 
 
 
 
 
 
m_refreshWidth 
 
int  m_refreshWidth  
 
 Shift the plot by this many pixels every time a point is plotted
 
 
 
 
 
 
m_refreshFrequency 
 
int  m_refreshFrequency  
 
 Plot every m_refreshFrequency'th point
 
 
 
 
 
 
m_Printer 
 
 PrintableComponent   m_Printer  
 
 the class responsible for printing
 
 
 
 
 
 
m_ce 
 
 ChartEvent   m_ce  
 
 
 
 
 
 
m_dataPoint 
 
double[]  m_dataPoint  
 
 
 
 

 
   
 
 
  
 Class  weka.gui.beans.StripChartCustomizer  extends javax.swing.JPanel implements Serializable   
 
 

 
 serialVersionUID:  7441741530975984608L

 
   
 
 
  
 Serialized Fields   
 
 

 
m_pcSupport 
 
java.beans.PropertyChangeSupport  m_pcSupport  
 
 
 
 
 
 
m_cvEditor 
 
 PropertySheetPanel   m_cvEditor  
 
 
 
 

 
   
 
 
  
 Class  weka.gui.beans.TestSetEvent  extends java.util.EventObject implements Serializable   
 
 

 
 serialVersionUID:  8780718708498854231L

 
   
 
 
  
 Serialized Fields   
 
 

 
m_testSet 
 
 Instances   m_testSet  
 
 The test set instances
 
 
 
 
 
 
m_structureOnly 
 
boolean  m_structureOnly  
 
 
 
 
 
 
m_runNumber 
 
int  m_runNumber  
 
 What run number is this training set from.
 
 
 
 
 
 
m_maxRunNumber 
 
int  m_maxRunNumber  
 
 Maximum number of runs.
 
 
 
 
 
 
m_setNumber 
 
int  m_setNumber  
 
 what number is this test set (ie fold 2 of 10 folds)
 
 
 
 
 
 
m_maxSetNumber 
 
int  m_maxSetNumber  
 
 Maximum number of sets (ie 10 in a 10 fold)
 
 
 
 

 
   
 
 
  
 Class  weka.gui.beans.TestSetMaker  extends  AbstractTestSetProducer  implements Serializable   
 
 

 
 serialVersionUID:  -8473882857628061841L

 
   
 
 
  
 Serialized Fields   
 
 

 
m_receivedStopNotification 
 
boolean  m_receivedStopNotification  
 
 
 
 

 
   
 
 
  
 Class  weka.gui.beans.TextEvent  extends java.util.EventObject implements Serializable   
 
 

 
 serialVersionUID:  4196810607402973744L

 
   
 
 
  
 Serialized Fields   
 
 

 
m_text 
 
java.lang.String  m_text  
 
 The text
 
 
 
 
 
 
m_textTitle 
 
java.lang.String  m_textTitle  
 
 The title for the text. Could be used in a list component
 
 
 
 

 
   
 
 
  
 Class  weka.gui.beans.TextViewer  extends javax.swing.JPanel implements Serializable   
 
 

 
 serialVersionUID:  104838186352536832L

 
   
 
 
  
 Serialized Fields   
 
 

 
m_visual 
 
 BeanVisual   m_visual  
 
 
 
 
 
 
m_design 
 
boolean  m_design  
 
 True if this bean's appearance is the design mode appearance
 
 
 
 
 
 
m_bcSupport 
 
java.beans.beancontext.BeanContextChildSupport  m_bcSupport  
 
 BeanContextChild support
 
 
 
 
 
 
m_textListeners 
 
java.util.Vector<E>  m_textListeners  
 
 Objects listening for text events
 
 
 
 

 
   
 
 
  
 Class  weka.gui.beans.ThresholdDataEvent  extends java.util.EventObject implements Serializable   
 
 

 
 serialVersionUID:  -8309334224492439644L

 
   
 
 
  
 Serialized Fields   
 
 

 
m_dataSet 
 
 PlotData2D   m_dataSet  
 
 
 
 
 
 
m_classAttribute 
 
 Attribute   m_classAttribute  
 
 
 
 

 
   
 
 
  
 Class  weka.gui.beans.TrainingSetEvent  extends java.util.EventObject implements Serializable   
 
 

 
 serialVersionUID:  5872343811810872662L

 
   
 
 
  
 Serialized Fields   
 
 

 
m_trainingSet 
 
 Instances   m_trainingSet  
 
 The training instances
 
 
 
 
 
 
m_structureOnly 
 
boolean  m_structureOnly  
 
 
 
 
 
 
m_runNumber 
 
int  m_runNumber  
 
 What run number is this training set from.
 
 
 
 
 
 
m_maxRunNumber 
 
int  m_maxRunNumber  
 
 Maximum number of runs.
 
 
 
 
 
 
m_setNumber 
 
int  m_setNumber  
 
 what number is this training set (ie fold 2 of 10 folds)
 
 
 
 
 
 
m_maxSetNumber 
 
int  m_maxSetNumber  
 
 Maximum number of sets (ie 10 in a 10 fold)
 
 
 
 

 
   
 
 
  
 Class  weka.gui.beans.TrainingSetMaker  extends  AbstractTrainingSetProducer  implements Serializable   
 
 

 
 serialVersionUID:  -6152577265471535786L

 
   
 
 
  
 Serialized Fields   
 
 

 
m_receivedStopNotification 
 
boolean  m_receivedStopNotification  
 
 
 
 

 
   
 
 
  
 Class  weka.gui.beans.TrainTestSplitMaker  extends  AbstractTrainAndTestSetProducer  implements Serializable   
 
 

 
 serialVersionUID:  7390064039444605943L

 
   
 
 
  
 Serialized Fields   
 
 

 
m_trainPercentage 
 
double  m_trainPercentage  
 
 
 
 
 
 
m_randomSeed 
 
int  m_randomSeed  
 
 
 
 
 
 
m_splitThread 
 
java.lang.Thread  m_splitThread  
 
 
 
 

 
   
 
 
  
 Class  weka.gui.beans.TrainTestSplitMakerCustomizer  extends javax.swing.JPanel implements Serializable   
 
 

 
 serialVersionUID:  -1684662340241807260L

 
   
 
 
  
 Serialized Fields   
 
 

 
m_pcSupport 
 
java.beans.PropertyChangeSupport  m_pcSupport  
 
 
 
 
 
 
m_splitEditor 
 
 PropertySheetPanel   m_splitEditor  
 
 
 
 

 
   
 
 
  
 Class  weka.gui.beans.VisualizableErrorEvent  extends java.util.EventObject implements Serializable   
 
 

 
 serialVersionUID:  -5811819270887223400L

 
   
 
 
  
 Serialized Fields   
 
 

 
m_dataSet 
 
 PlotData2D   m_dataSet  
 
 
 
 
 

 
 
  
 Package   weka.gui.boundaryvisualizer   
 
 

 
   
 
 
  
 Class  weka.gui.boundaryvisualizer.BoundaryPanel  extends javax.swing.JPanel implements Serializable   
 
 

 
 serialVersionUID:  -8499445518744770458L

 
   
 
 
  
 Serialized Fields   
 
 

 
m_Colors 
 
 FastVector   m_Colors  
 
 
 
 
 
 
m_trainingData 
 
 Instances   m_trainingData  
 
 training data
 
 
 
 
 
 
m_classifier 
 
 Classifier   m_classifier  
 
 distribution classifier to use
 
 
 
 
 
 
m_dataGenerator 
 
 DataGenerator   m_dataGenerator  
 
 data generator to use
 
 
 
 
 
 
m_classIndex 
 
int  m_classIndex  
 
 index of the class attribute
 
 
 
 
 
 
m_xAttribute 
 
int  m_xAttribute  
 
 
 
 
 
 
m_yAttribute 
 
int  m_yAttribute  
 
 
 
 
 
 
m_minX 
 
double  m_minX  
 
 
 
 
 
 
m_minY 
 
double  m_minY  
 
 
 
 
 
 
m_maxX 
 
double  m_maxX  
 
 
 
 
 
 
m_maxY 
 
double  m_maxY  
 
 
 
 
 
 
m_rangeX 
 
double  m_rangeX  
 
 
 
 
 
 
m_rangeY 
 
double  m_rangeY  
 
 
 
 
 
 
m_pixHeight 
 
double  m_pixHeight  
 
 
 
 
 
 
m_pixWidth 
 
double  m_pixWidth  
 
 
 
 
 
 
m_osi 
 
java.awt.Image  m_osi  
 
 used for offscreen drawing
 
 
 
 
 
 
m_panelWidth 
 
int  m_panelWidth  
 
 
 
 
 
 
m_panelHeight 
 
int  m_panelHeight  
 
 
 
 
 
 
m_numOfSamplesPerRegion 
 
int  m_numOfSamplesPerRegion  
 
 
 
 
 
 
m_numOfSamplesPerGenerator 
 
int  m_numOfSamplesPerGenerator  
 
 
 
 
 
 
m_samplesBase 
 
double  m_samplesBase  
 
 
 
 
 
 
m_listeners 
 
java.util.Vector<E>  m_listeners  
 
 listeners to be notified when plot is complete
 
 
 
 
 
 
m_plotPanel 
 
weka.gui.boundaryvisualizer.BoundaryPanel.PlotPanel  m_plotPanel  
 
 the actual plotting area
 
 
 
 
 
 
m_plotThread 
 
java.lang.Thread  m_plotThread  
 
 thread for running the plotting operation in
 
 
 
 
 
 
m_stopPlotting 
 
boolean  m_stopPlotting  
 
 Stop the plotting thread
 
 
 
 
 
 
m_stopReplotting 
 
boolean  m_stopReplotting  
 
 Stop any replotting threads
 
 
 
 
 
 
m_dummy 
 
java.lang.Double  m_dummy  
 
 
 
 
 
 
m_pausePlotting 
 
boolean  m_pausePlotting  
 
 
 
 
 
 
m_size 
 
int  m_size  
 
 what size of tile is currently being plotted
 
 
 
 
 
 
m_initialTiling 
 
boolean  m_initialTiling  
 
 is the main plot thread performing the initial coarse tiling
 
 
 
 
 
 
m_random 
 
java.util.Random  m_random  
 
 A random number generator
 
 
 
 
 
 
m_probabilityCache 
 
double[][][]  m_probabilityCache  
 
 cache of probabilities for fast replotting
 
 
 
 
 
 
m_plotTrainingData 
 
boolean  m_plotTrainingData  
 
 plot the training data
 
 
 
 

 
   
 
 
  
 Class  weka.gui.boundaryvisualizer.BoundaryPanelDistributed  extends  BoundaryPanel  implements Serializable   
 
 

 
 serialVersionUID:  -1743284397893937776L

 
   
 
 
  
 Serialized Fields   
 
 

 
m_listeners 
 
java.util.Vector<E>  m_listeners  
 
 a list of RemoteExperimentListeners
 
 
 
 
 
 
m_remoteHosts 
 
java.util.Vector<E>  m_remoteHosts  
 
 Holds the names of machines with remoteEngine servers running
 
 
 
 
 
 
m_remoteHostsQueue 
 
 Queue   m_remoteHostsQueue  
 
 The queue of available hosts
 
 
 
 
 
 
m_remoteHostsStatus 
 
int[]  m_remoteHostsStatus  
 
 The status of each of the remote hosts
 
 
 
 
 
 
m_remoteHostFailureCounts 
 
int[]  m_remoteHostFailureCounts  
 
 The number of times tasks have failed on each remote host
 
 
 
 
 
 
m_plottingAborted 
 
boolean  m_plottingAborted  
 
 Set to true if MAX_FAILURES exceeded on all hosts or connections fail 
      on all hosts or user aborts plotting
 
 
 
 
 
 
m_removedHosts 
 
int  m_removedHosts  
 
 The number of hosts removed due to exceeding max failures
 
 
 
 
 
 
m_failedCount 
 
int  m_failedCount  
 
 The count of failed sub-tasks
 
 
 
 
 
 
m_finishedCount 
 
int  m_finishedCount  
 
 The count of successfully completed sub-tasks
 
 
 
 
 
 
m_subExpQueue 
 
 Queue   m_subExpQueue  
 
 The queue of sub-tasks waiting to be processed
 
 
 
 
 
 
m_minTaskPollTime 
 
int  m_minTaskPollTime  
 
 number of seconds between polling server
 
 
 
 
 
 
m_hostPollingTime 
 
int[]  m_hostPollingTime  
 
 
 
 

 
   
 
 
  
 Class  weka.gui.boundaryvisualizer.BoundaryVisualizer  extends javax.swing.JPanel implements Serializable   
 
 

 
 serialVersionUID:  3933877580074013208L

 
   
 
 
  
 Serialized Fields   
 
 

 
m_trainingInstances 
 
 Instances   m_trainingInstances  
 
 the training instances
 
 
 
 
 
 
m_classifier 
 
 Classifier   m_classifier  
 
 the classifier to use
 
 
 
 
 
 
m_plotAreaWidth 
 
int  m_plotAreaWidth  
 
 
 
 
 
 
m_plotAreaHeight 
 
int  m_plotAreaHeight  
 
 
 
 
 
 
m_boundaryPanel 
 
 BoundaryPanel   m_boundaryPanel  
 
 the plotting panel
 
 
 
 
 
 
m_classAttBox 
 
javax.swing.JComboBox  m_classAttBox  
 
 
 
 
 
 
m_xAttBox 
 
javax.swing.JComboBox  m_xAttBox  
 
 
 
 
 
 
m_yAttBox 
 
javax.swing.JComboBox  m_yAttBox  
 
 
 
 
 
 
COMBO_SIZE 
 
java.awt.Dimension  COMBO_SIZE  
 
 
 
 
 
 
m_startBut 
 
javax.swing.JButton  m_startBut  
 
 
 
 
 
 
m_plotTrainingData 
 
javax.swing.JCheckBox  m_plotTrainingData  
 
 
 
 
 
 
m_controlPanel 
 
javax.swing.JPanel  m_controlPanel  
 
 
 
 
 
 
m_classPanel 
 
 ClassPanel   m_classPanel  
 
 
 
 
 
 
m_xAxisPanel 
 
weka.gui.boundaryvisualizer.BoundaryVisualizer.AxisPanel  m_xAxisPanel  
 
 
 
 
 
 
m_yAxisPanel 
 
weka.gui.boundaryvisualizer.BoundaryVisualizer.AxisPanel  m_yAxisPanel  
 
 
 
 
 
 
m_maxX 
 
double  m_maxX  
 
 
 
 
 
 
m_maxY 
 
double  m_maxY  
 
 
 
 
 
 
m_minX 
 
double  m_minX  
 
 
 
 
 
 
m_minY 
 
double  m_minY  
 
 
 
 
 
 
m_xIndex 
 
int  m_xIndex  
 
 
 
 
 
 
m_yIndex 
 
int  m_yIndex  
 
 
 
 
 
 
m_dataGenerator 
 
 KDDataGenerator   m_dataGenerator  
 
 
 
 
 
 
m_numberOfSamplesFromEachRegion 
 
int  m_numberOfSamplesFromEachRegion  
 
 
 
 
 
 
m_generatorSamplesBase 
 
int  m_generatorSamplesBase  
 
 base for sampling in the non-fixed dimensions
 
 
 
 
 
 
m_kernelBandwidth 
 
int  m_kernelBandwidth  
 
 Set the kernel bandwidth to cover this many nearest neighbours
 
 
 
 
 
 
m_regionSamplesText 
 
javax.swing.JTextField  m_regionSamplesText  
 
 
 
 
 
 
m_generatorSamplesText 
 
javax.swing.JTextField  m_generatorSamplesText  
 
 
 
 
 
 
m_kernelBandwidthText 
 
javax.swing.JTextField  m_kernelBandwidthText  
 
 
 
 
 
 
m_classifierEditor 
 
 GenericObjectEditor   m_classifierEditor  
 
 
 
 
 
 
m_ClassifierPanel 
 
 PropertyPanel   m_ClassifierPanel  
 
 
 
 
 
 
m_FileChooser 
 
javax.swing.JFileChooser  m_FileChooser  
 
 The file chooser for selecting arff files
 
 
 
 
 
 
m_arffFileFilter 
 
 ExtensionFileFilter   m_arffFileFilter  
 
 
 
 
 
 
dataFileLabel 
 
javax.swing.JLabel  dataFileLabel  
 
 
 
 
 
 
m_addRemovePointsPanel 
 
javax.swing.JPanel  m_addRemovePointsPanel  
 
 
 
 
 
 
m_classValueSelector 
 
javax.swing.JComboBox  m_classValueSelector  
 
 
 
 
 
 
m_addPointsButton 
 
javax.swing.JRadioButton  m_addPointsButton  
 
 
 
 
 
 
m_removePointsButton 
 
javax.swing.JRadioButton  m_removePointsButton  
 
 
 
 
 
 
m_addRemovePointsButtonGroup 
 
javax.swing.ButtonGroup  m_addRemovePointsButtonGroup  
 
 
 
 
 
 
removeAllButton 
 
javax.swing.JButton  removeAllButton  
 
 
 
 
 
 
chooseButton 
 
javax.swing.JButton  chooseButton  
 
 
 
 

 
   
 
 
  
 Class  weka.gui.boundaryvisualizer.KDDataGenerator  extends java.lang.Object implements Serializable   
 
 

 
 serialVersionUID:  -958573275606402792L

 
   
 
 
  
 Serialized Fields   
 
 

 
m_instances 
 
 Instances   m_instances  
 
 the instances to use
 
 
 
 
 
 
m_standardDeviations 
 
double[]  m_standardDeviations  
 
 standard deviations of the normal distributions for numeric attributes in
 each KD estimator
 
 
 
 
 
 
m_globalMeansOrModes 
 
double[]  m_globalMeansOrModes  
 
 global means or modes to use for missing values
 
 
 
 
 
 
m_minStdDev 
 
double  m_minStdDev  
 
 minimum standard deviation for numeric attributes
 
 
 
 
 
 
m_laplaceConst 
 
double  m_laplaceConst  
 
 Laplace correction for discrete distributions
 
 
 
 
 
 
m_seed 
 
int  m_seed  
 
 random number seed
 
 
 
 
 
 
m_random 
 
java.util.Random  m_random  
 
 random number generator
 
 
 
 
 
 
m_weightingDimensions 
 
boolean[]  m_weightingDimensions  
 
 which dimensions to use for computing a weight for each generated
 instance
 
 
 
 
 
 
m_weightingValues 
 
double[]  m_weightingValues  
 
 the values for the weighting dimensions to use for computing the weight 
 for the next instance to be generated
 
 
 
 
 
 
m_kernelBandwidth 
 
int  m_kernelBandwidth  
 
 Number of neighbours to use for kernel bandwidth
 
 
 
 
 
 
m_kernelParams 
 
double[][]  m_kernelParams  
 
 standard deviations for numeric attributes computed from the 
 m_kernelBandwidth nearest neighbours for each kernel.
 
 
 
 
 
 
m_Min 
 
double[]  m_Min  
 
 The minimum values for numeric attributes.
 
 
 
 
 
 
m_Max 
 
double[]  m_Max  
 
 The maximum values for numeric attributes.
 
 
 
 

 
   
 
 
  
 Class  weka.gui.boundaryvisualizer.RemoteBoundaryVisualizerSubTask  extends java.lang.Object implements Serializable   
 
 

 
   
 
 
  
 Serialized Fields   
 
 

 
m_status 
 
 TaskStatusInfo   m_status  
 
 
 
 
 
 
m_result 
 
 RemoteResult   m_result  
 
 
 
 
 
 
m_rowNumber 
 
int  m_rowNumber  
 
 
 
 
 
 
m_panelHeight 
 
int  m_panelHeight  
 
 
 
 
 
 
m_panelWidth 
 
int  m_panelWidth  
 
 
 
 
 
 
m_classifier 
 
 Classifier   m_classifier  
 
 
 
 
 
 
m_dataGenerator 
 
 DataGenerator   m_dataGenerator  
 
 
 
 
 
 
m_trainingData 
 
 Instances   m_trainingData  
 
 
 
 
 
 
m_xAttribute 
 
int  m_xAttribute  
 
 
 
 
 
 
m_yAttribute 
 
int  m_yAttribute  
 
 
 
 
 
 
m_pixHeight 
 
double  m_pixHeight  
 
 
 
 
 
 
m_pixWidth 
 
double  m_pixWidth  
 
 
 
 
 
 
m_minX 
 
double  m_minX  
 
 
 
 
 
 
m_minY 
 
double  m_minY  
 
 
 
 
 
 
m_maxX 
 
double  m_maxX  
 
 
 
 
 
 
m_maxY 
 
double  m_maxY  
 
 
 
 
 
 
m_numOfSamplesPerRegion 
 
int  m_numOfSamplesPerRegion  
 
 
 
 
 
 
m_numOfSamplesPerGenerator 
 
int  m_numOfSamplesPerGenerator  
 
 
 
 
 
 
m_samplesBase 
 
double  m_samplesBase  
 
 
 
 
 
 
m_random 
 
java.util.Random  m_random  
 
 
 
 
 
 
m_weightingAttsValues 
 
double[]  m_weightingAttsValues  
 
 
 
 
 
 
m_attsToWeightOn 
 
boolean[]  m_attsToWeightOn  
 
 
 
 
 
 
m_vals 
 
double[]  m_vals  
 
 
 
 
 
 
m_dist 
 
double[]  m_dist  
 
 
 
 
 
 
m_predInst 
 
 Instance   m_predInst  
 
 
 
 

 
   
 
 
  
 Class  weka.gui.boundaryvisualizer.RemoteResult  extends java.lang.Object implements Serializable   
 
 

 
 serialVersionUID:  1873271280044633808L

 
   
 
 
  
 Serialized Fields   
 
 

 
m_rowNumber 
 
int  m_rowNumber  
 
 the row number that this result corresponds to
 
 
 
 
 
 
m_rowLength 
 
int  m_rowLength  
 
 how many pixels in a row
 
 
 
 
 
 
m_probabilities 
 
double[][]  m_probabilities  
 
 the result - ie. the probability distributions produced by the
 classifier for this row in the visualization
 
 
 
 
 
 
m_percentCompleted 
 
int  m_percentCompleted  
 
 progress on computing this row
 
 
 
 
 

 
 
  
 Package   weka.gui.experiment   
 
 

 
   
 
 
  
 Class  weka.gui.experiment.AlgorithmListPanel  extends javax.swing.JPanel implements Serializable   
 
 

 
 serialVersionUID:  -7204528834764898671L

 
   
 
 
  
 Serialized Fields   
 
 

 
m_Exp 
 
 Experiment   m_Exp  
 
 The experiment to set the algorithm list of
 
 
 
 
 
 
m_List 
 
javax.swing.JList  m_List  
 
 The component displaying the algorithm list
 
 
 
 
 
 
m_AddBut 
 
javax.swing.JButton  m_AddBut  
 
 Click to add an algorithm
 
 
 
 
 
 
m_EditBut 
 
javax.swing.JButton  m_EditBut  
 
 Click to edit the selected algorithm
 
 
 
 
 
 
m_DeleteBut 
 
javax.swing.JButton  m_DeleteBut  
 
 Click to remove the selected dataset from the list
 
 
 
 
 
 
m_LoadOptionsBut 
 
javax.swing.JButton  m_LoadOptionsBut  
 
 Click to edit the load the options for athe selected algorithm
 
 
 
 
 
 
m_SaveOptionsBut 
 
javax.swing.JButton  m_SaveOptionsBut  
 
 Click to edit the save the options from selected algorithm
 
 
 
 
 
 
m_UpBut 
 
javax.swing.JButton  m_UpBut  
 
 Click to move the selected algorithm(s) one up
 
 
 
 
 
 
m_DownBut 
 
javax.swing.JButton  m_DownBut  
 
 Click to move the selected algorithm(s) one down
 
 
 
 
 
 
m_FileChooser 
 
javax.swing.JFileChooser  m_FileChooser  
 
 The file chooser for selecting experiments
 
 
 
 
 
 
m_XMLFilter 
 
javax.swing.filechooser.FileFilter  m_XMLFilter  
 
 A filter to ensure only experiment (in XML format) files get shown in the chooser
 
 
 
 
 
 
m_Editing 
 
boolean  m_Editing  
 
 Whether an algorithm is added or only edited
 
 
 
 
 
 
m_ClassifierEditor 
 
 GenericObjectEditor   m_ClassifierEditor  
 
 Lets the user configure the classifier
 
 
 
 
 
 
m_PD 
 
 PropertyDialog   m_PD  
 
 The currently displayed property dialog, if any
 
 
 
 
 
 
m_AlgorithmListModel 
 
javax.swing.DefaultListModel  m_AlgorithmListModel  
 
 The list model used
 
 
 
 

 
   
 
 
  
 Class  weka.gui.experiment.AlgorithmListPanel.ObjectCellRenderer  extends javax.swing.DefaultListCellRenderer implements Serializable   
 
 

 
 serialVersionUID:  -5067138526587433808L

 

 
   
 
 
  
 Class  weka.gui.experiment.DatasetListPanel  extends javax.swing.JPanel implements Serializable   
 
 

 
 serialVersionUID:  7068857852794405769L

 
   
 
 
  
 Serialized Fields   
 
 

 
m_Exp 
 
 Experiment   m_Exp  
 
 The experiment to set the dataset list of.
 
 
 
 
 
 
m_List 
 
javax.swing.JList  m_List  
 
 The component displaying the dataset list.
 
 
 
 
 
 
m_AddBut 
 
javax.swing.JButton  m_AddBut  
 
 Click to add a dataset.
 
 
 
 
 
 
m_EditBut 
 
javax.swing.JButton  m_EditBut  
 
 Click to edit the selected algorithm.
 
 
 
 
 
 
m_DeleteBut 
 
javax.swing.JButton  m_DeleteBut  
 
 Click to remove the selected dataset from the list.
 
 
 
 
 
 
m_UpBut 
 
javax.swing.JButton  m_UpBut  
 
 Click to move the selected dataset(s) one up.
 
 
 
 
 
 
m_DownBut 
 
javax.swing.JButton  m_DownBut  
 
 Click to move the selected dataset(s) one down.
 
 
 
 
 
 
m_relativeCheck 
 
javax.swing.JCheckBox  m_relativeCheck  
 
 Make file paths relative to the user (start) directory.
 
 
 
 
 
 
m_FileChooser 
 
 ConverterFileChooser   m_FileChooser  
 
 The file chooser component.
 
 
 
 

 
   
 
 
  
 Class  weka.gui.experiment.DistributeExperimentPanel  extends javax.swing.JPanel implements Serializable   
 
 

 
 serialVersionUID:  5206721431754800278L

 
   
 
 
  
 Serialized Fields   
 
 

 
m_Exp 
 
 RemoteExperiment   m_Exp  
 
 The experiment to configure.
 
 
 
 
 
 
m_enableDistributedExperiment 
 
javax.swing.JCheckBox  m_enableDistributedExperiment  
 
 Distribute the current experiment to remote hosts
 
 
 
 
 
 
m_configureHostNames 
 
javax.swing.JButton  m_configureHostNames  
 
 Popup the HostListPanel
 
 
 
 
 
 
m_hostList 
 
 HostListPanel   m_hostList  
 
 The host list panel
 
 
 
 
 
 
m_splitByDataSet 
 
javax.swing.JRadioButton  m_splitByDataSet  
 
 Split experiment up by data set.
 
 
 
 
 
 
m_splitByRun 
 
javax.swing.JRadioButton  m_splitByRun  
 
 Split experiment up by run number.
 
 
 
 
 
 
m_radioListener 
 
java.awt.event.ActionListener  m_radioListener  
 
 Handle radio buttons
 
 
 
 

 
   
 
 
  
 Class  weka.gui.experiment.Experimenter  extends javax.swing.JPanel implements Serializable   
 
 

 
 serialVersionUID:  -5751617505738193788L

 
   
 
 
  
 Serialized Fields   
 
 

 
m_SetupPanel 
 
 SetupModePanel   m_SetupPanel  
 
 The panel for configuring the experiment
 
 
 
 
 
 
m_RunPanel 
 
 RunPanel   m_RunPanel  
 
 The panel for running the experiment
 
 
 
 
 
 
m_ResultsPanel 
 
 ResultsPanel   m_ResultsPanel  
 
 The panel for analysing experimental results
 
 
 
 
 
 
m_TabbedPane 
 
javax.swing.JTabbedPane  m_TabbedPane  
 
 The tabbed pane that controls which sub-pane we are working with
 
 
 
 
 
 
m_ClassFirst 
 
boolean  m_ClassFirst  
 
 True if the class attribute is the first attribute for all
      datasets involved in this experiment.
 
 
 
 

 
   
 
 
  
 Class  weka.gui.experiment.ExperimenterDefaults  extends java.lang.Object implements Serializable   
 
 

 
 serialVersionUID:  -2835933184632147981L

 

 
   
 
 
  
 Class  weka.gui.experiment.GeneratorPropertyIteratorPanel  extends javax.swing.JPanel implements Serializable   
 
 

 
 serialVersionUID:  -6026938995241632139L

 
   
 
 
  
 Serialized Fields   
 
 

 
m_ConfigureBut 
 
javax.swing.JButton  m_ConfigureBut  
 
 Click to select the property to iterate over
 
 
 
 
 
 
m_StatusBox 
 
javax.swing.JComboBox  m_StatusBox  
 
 Controls whether the custom iterator is used or not
 
 
 
 
 
 
m_ArrayEditor 
 
 GenericArrayEditor   m_ArrayEditor  
 
 Allows editing of the custom property values
 
 
 
 
 
 
m_Exp 
 
 Experiment   m_Exp  
 
 The experiment this all applies to
 
 
 
 
 
 
m_Listeners 
 
 FastVector   m_Listeners  
 
 Listeners who want to be notified about editing status of this
      panel
 
 
 
 

 
   
 
 
  
 Class  weka.gui.experiment.HostListPanel  extends javax.swing.JPanel implements Serializable   
 
 

 
 serialVersionUID:  7182791134585882197L

 
   
 
 
  
 Serialized Fields   
 
 

 
m_Exp 
 
 RemoteExperiment   m_Exp  
 
 The remote experiment to set the host list of
 
 
 
 
 
 
m_List 
 
javax.swing.JList  m_List  
 
 The component displaying the host list
 
 
 
 
 
 
m_DeleteBut 
 
javax.swing.JButton  m_DeleteBut  
 
 Click to remove the selected host from the list
 
 
 
 
 
 
m_HostField 
 
javax.swing.JTextField  m_HostField  
 
 The field with which to enter host names
 
 
 
 

 
   
 
 
  
 Class  weka.gui.experiment.OutputFormatDialog  extends javax.swing.JDialog implements Serializable   
 
 

 
 serialVersionUID:  2169792738187807378L

 
   
 
 
  
 Serialized Fields   
 
 

 
m_Result 
 
int  m_Result  
 
 the result of the user's action, either OK or CANCEL.
 
 
 
 
 
 
m_ResultMatrix 
 
java.lang.Class<T>  m_ResultMatrix  
 
 the output format specific matrix.
 
 
 
 
 
 
m_OutputFormatComboBox 
 
javax.swing.JComboBox  m_OutputFormatComboBox  
 
 lets the user choose the format for the output.
 
 
 
 
 
 
m_MeanPrecSpinner 
 
javax.swing.JSpinner  m_MeanPrecSpinner  
 
 the spinner to choose the precision for the mean from.
 
 
 
 
 
 
m_StdDevPrecSpinner 
 
javax.swing.JSpinner  m_StdDevPrecSpinner  
 
 the spinner to choose the precision for the std. deviation from
 
 
 
 
 
 
m_ShowAverageCheckBox 
 
javax.swing.JCheckBox  m_ShowAverageCheckBox  
 
 the checkbox for outputting the average.
 
 
 
 
 
 
m_RemoveFilterNameCheckBox 
 
javax.swing.JCheckBox  m_RemoveFilterNameCheckBox  
 
 the checkbox for the removing of filter classnames.
 
 
 
 
 
 
m_OkButton 
 
javax.swing.JButton  m_OkButton  
 
 Click to activate the current set parameters.
 
 
 
 
 
 
m_CancelButton 
 
javax.swing.JButton  m_CancelButton  
 
 Click to cancel the dialog.
 
 
 
 
 
 
m_MeanPrec 
 
int  m_MeanPrec  
 
 the number of digits after the period (= precision) for printing the mean.
 
 
 
 
 
 
m_StdDevPrec 
 
int  m_StdDevPrec  
 
 the number of digits after the period (= precision) for printing the std.
 deviation
 
 
 
 
 
 
m_RemoveFilterName 
 
boolean  m_RemoveFilterName  
 
 whether to remove the filter names from the names.
 
 
 
 
 
 
m_ShowAverage 
 
boolean  m_ShowAverage  
 
 whether to show the average too.
 
 
 
 

 
   
 
 
  
 Class  weka.gui.experiment.ResultsPanel  extends javax.swing.JPanel implements Serializable   
 
 

 
 serialVersionUID:  -4913007978534178569L

 
   
 
 
  
 Serialized Fields   
 
 

 
m_FromFileBut 
 
javax.swing.JButton  m_FromFileBut  
 
 Click to load results from a file.
 
 
 
 
 
 
m_FromDBaseBut 
 
javax.swing.JButton  m_FromDBaseBut  
 
 Click to load results from a database.
 
 
 
 
 
 
m_FromExpBut 
 
javax.swing.JButton  m_FromExpBut  
 
 Click to get results from the destination given in the experiment.
 
 
 
 
 
 
m_FromLab 
 
javax.swing.JLabel  m_FromLab  
 
 Displays a message about the current result set.
 
 
 
 
 
 
m_DatasetModel 
 
javax.swing.DefaultComboBoxModel  m_DatasetModel  
 
 The model embedded in m_DatasetCombo.
 
 
 
 
 
 
m_CompareModel 
 
javax.swing.DefaultComboBoxModel  m_CompareModel  
 
 The model embedded in m_CompareCombo.
 
 
 
 
 
 
m_SortModel 
 
javax.swing.DefaultComboBoxModel  m_SortModel  
 
 The model embedded in m_SortCombo.
 
 
 
 
 
 
m_TestsModel 
 
javax.swing.DefaultListModel  m_TestsModel  
 
 The model embedded in m_TestsList.
 
 
 
 
 
 
m_DisplayedModel 
 
javax.swing.DefaultListModel  m_DisplayedModel  
 
 The model embedded in m_DisplayedList.
 
 
 
 
 
 
m_TesterClassesLabel 
 
javax.swing.JLabel  m_TesterClassesLabel  
 
 Displays the currently selected Tester-Class.
 
 
 
 
 
 
m_TesterClasses 
 
javax.swing.JComboBox  m_TesterClasses  
 
 Lists all the available classes implementing the Tester-Interface.
 
 
  See Also:    Tester   
 
 
 
m_DatasetKeyLabel 
 
javax.swing.JLabel  m_DatasetKeyLabel  
 
 Displays the currently selected column names for the scheme & options.
 
 
 
 
 
 
m_DatasetKeyBut 
 
javax.swing.JButton  m_DatasetKeyBut  
 
 Click to edit the columns used to determine the scheme.
 
 
 
 
 
 
m_DatasetKeyModel 
 
javax.swing.DefaultListModel  m_DatasetKeyModel  
 
 Stores the list of attributes for selecting the scheme columns.
 
 
 
 
 
 
m_DatasetKeyList 
 
javax.swing.JList  m_DatasetKeyList  
 
 Displays the list of selected columns determining the scheme.
 
 
 
 
 
 
m_ResultKeyLabel 
 
javax.swing.JLabel  m_ResultKeyLabel  
 
 Displays the currently selected column names for the scheme & options.
 
 
 
 
 
 
m_ResultKeyBut 
 
javax.swing.JButton  m_ResultKeyBut  
 
 Click to edit the columns used to determine the scheme.
 
 
 
 
 
 
m_ResultKeyModel 
 
javax.swing.DefaultListModel  m_ResultKeyModel  
 
 Stores the list of attributes for selecting the scheme columns.
 
 
 
 
 
 
m_ResultKeyList 
 
javax.swing.JList  m_ResultKeyList  
 
 Displays the list of selected columns determining the scheme.
 
 
 
 
 
 
m_TestsButton 
 
javax.swing.JButton  m_TestsButton  
 
 Lets the user select which scheme to base comparisons against.
 
 
 
 
 
 
m_DisplayedButton 
 
javax.swing.JButton  m_DisplayedButton  
 
 Lets the user select which schemes are compared to base.
 
 
 
 
 
 
m_TestsList 
 
javax.swing.JList  m_TestsList  
 
 Holds the list of schemes to base the test against.
 
 
 
 
 
 
m_DisplayedList 
 
javax.swing.JList  m_DisplayedList  
 
 Holds the list of schemes to display.
 
 
 
 
 
 
m_CompareCombo 
 
javax.swing.JComboBox  m_CompareCombo  
 
 Lets the user select which performance measure to analyze.
 
 
 
 
 
 
m_SortCombo 
 
javax.swing.JComboBox  m_SortCombo  
 
 Lets the user select which column to use for sorting.
 
 
 
 
 
 
m_SigTex 
 
javax.swing.JTextField  m_SigTex  
 
 Lets the user edit the test significance.
 
 
 
 
 
 
m_ShowStdDevs 
 
javax.swing.JCheckBox  m_ShowStdDevs  
 
 Lets the user select whether standard deviations are to be output
      or not.
 
 
 
 
 
 
m_OutputFormatButton 
 
javax.swing.JButton  m_OutputFormatButton  
 
 lets the user choose the format for the output.
 
 
 
 
 
 
m_PerformBut 
 
javax.swing.JButton  m_PerformBut  
 
 Click to start the test.
 
 
 
 
 
 
m_SaveOutBut 
 
javax.swing.JButton  m_SaveOutBut  
 
 Click to save test output to a file.
 
 
 
 
 
 
m_SaveOut 
 
 SaveBuffer   m_SaveOut  
 
 The buffer saving object for saving output.
 
 
 
 
 
 
m_OutText 
 
javax.swing.JTextArea  m_OutText  
 
 Displays the output of tests.
 
 
 
 
 
 
m_History 
 
 ResultHistoryPanel   m_History  
 
 A panel controlling results viewing.
 
 
 
 
 
 
m_FileChooser 
 
javax.swing.JFileChooser  m_FileChooser  
 
 The file chooser for selecting result files.
 
 
 
 
 
 
m_csvFileFilter 
 
 ExtensionFileFilter   m_csvFileFilter  
 
 CSV file filter.
 
 
 
 
 
 
m_arffFileFilter 
 
 ExtensionFileFilter   m_arffFileFilter  
 
 ARFF file filter.
 
 
 
 
 
 
m_TTester 
 
 Tester   m_TTester  
 
 The PairedTTester object.
 
 
 
 
 
 
m_Instances 
 
 Instances   m_Instances  
 
 The instances we're extracting results from.
 
 
 
 
 
 
m_InstanceQuery 
 
 InstanceQuery   m_InstanceQuery  
 
 Does any database querying for us.
 
 
 
 
 
 
m_LoadThread 
 
java.lang.Thread  m_LoadThread  
 
 A thread to load results instances from a file or database.
 
 
 
 
 
 
m_Exp 
 
 Experiment   m_Exp  
 
 An experiment (used for identifying a result source) -- optional.
 
 
 
 
 
 
COMBO_SIZE 
 
java.awt.Dimension  COMBO_SIZE  
 
 the size for a combobox.
 
 
 
 
 
 
m_ResultMatrix 
 
 ResultMatrix   m_ResultMatrix  
 
 the initial result matrix.
 
 
 
 

 
   
 
 
  
 Class  weka.gui.experiment.RunNumberPanel  extends javax.swing.JPanel implements Serializable   
 
 

 
 serialVersionUID:  -1644336658426067852L

 
   
 
 
  
 Serialized Fields   
 
 

 
m_LowerText 
 
javax.swing.JTextField  m_LowerText  
 
 Configures the lower run number
 
 
 
 
 
 
m_UpperText 
 
javax.swing.JTextField  m_UpperText  
 
 Configures the upper run number
 
 
 
 
 
 
m_Exp 
 
 Experiment   m_Exp  
 
 The experiment being configured
 
 
 
 

 
   
 
 
  
 Class  weka.gui.experiment.RunPanel  extends javax.swing.JPanel implements Serializable   
 
 

 
 serialVersionUID:  1691868018596872051L

 
   
 
 
  
 Serialized Fields   
 
 

 
m_StartBut 
 
javax.swing.JButton  m_StartBut  
 
 Click to start running the experiment
 
 
 
 
 
 
m_StopBut 
 
javax.swing.JButton  m_StopBut  
 
 Click to signal the running experiment to halt
 
 
 
 
 
 
m_Log 
 
 LogPanel   m_Log  
 
 
 
 
 
 
m_Exp 
 
 Experiment   m_Exp  
 
 The experiment to run
 
 
 
 
 
 
m_RunThread 
 
java.lang.Thread  m_RunThread  
 
 The thread running the experiment
 
 
 
 
 
 
m_ResultsPanel 
 
 ResultsPanel   m_ResultsPanel  
 
 A pointer to the results panel
 
 
 
 

 
   
 
 
  
 Class  weka.gui.experiment.SetupModePanel  extends javax.swing.JPanel implements Serializable   
 
 

 
 serialVersionUID:  -3758035565520727822L

 
   
 
 
  
 Serialized Fields   
 
 

 
m_SimpleSetupRBut 
 
javax.swing.JRadioButton  m_SimpleSetupRBut  
 
 The button for choosing simple setup mode
 
 
 
 
 
 
m_AdvancedSetupRBut 
 
javax.swing.JRadioButton  m_AdvancedSetupRBut  
 
 The button for choosing advanced setup mode
 
 
 
 
 
 
m_simplePanel 
 
 SimpleSetupPanel   m_simplePanel  
 
 The simple setup panel
 
 
 
 
 
 
m_advancedPanel 
 
 SetupPanel   m_advancedPanel  
 
 The advanced setup panel
 
 
 
 

 
   
 
 
  
 Class  weka.gui.experiment.SetupPanel  extends javax.swing.JPanel implements Serializable   
 
 

 
 serialVersionUID:  6552671886903170033L

 
   
 
 
  
 Serialized Fields   
 
 

 
m_Exp 
 
 Experiment   m_Exp  
 
 The experiment being configured
 
 
 
 
 
 
m_OpenBut 
 
javax.swing.JButton  m_OpenBut  
 
 Click to load an experiment
 
 
 
 
 
 
m_SaveBut 
 
javax.swing.JButton  m_SaveBut  
 
 Click to save an experiment
 
 
 
 
 
 
m_NewBut 
 
javax.swing.JButton  m_NewBut  
 
 Click to create a new experiment with default settings
 
 
 
 
 
 
m_ExpFilter 
 
javax.swing.filechooser.FileFilter  m_ExpFilter  
 
 A filter to ensure only experiment files get shown in the chooser
 
 
 
 
 
 
m_KOMLFilter 
 
javax.swing.filechooser.FileFilter  m_KOMLFilter  
 
 A filter to ensure only experiment (in KOML format) files get shown in the chooser
 
 
 
 
 
 
m_XMLFilter 
 
javax.swing.filechooser.FileFilter  m_XMLFilter  
 
 A filter to ensure only experiment (in XML format) files get shown in the chooser
 
 
 
 
 
 
m_FileChooser 
 
javax.swing.JFileChooser  m_FileChooser  
 
 The file chooser for selecting experiments
 
 
 
 
 
 
m_RPEditor 
 
 GenericObjectEditor   m_RPEditor  
 
 The ResultProducer editor
 
 
 
 
 
 
m_RPEditorPanel 
 
 PropertyPanel   m_RPEditorPanel  
 
 The panel to contain the ResultProducer editor
 
 
 
 
 
 
m_RLEditor 
 
 GenericObjectEditor   m_RLEditor  
 
 The ResultListener editor
 
 
 
 
 
 
m_RLEditorPanel 
 
 PropertyPanel   m_RLEditorPanel  
 
 The panel to contain the ResultListener editor
 
 
 
 
 
 
m_GeneratorPropertyPanel 
 
 GeneratorPropertyIteratorPanel   m_GeneratorPropertyPanel  
 
 The panel that configures iteration on custom resultproducer property
 
 
 
 
 
 
m_RunNumberPanel 
 
 RunNumberPanel   m_RunNumberPanel  
 
 The panel for configuring run numbers
 
 
 
 
 
 
m_DistributeExperimentPanel 
 
 DistributeExperimentPanel   m_DistributeExperimentPanel  
 
 The panel for enabling a distributed experiment
 
 
 
 
 
 
m_DatasetListPanel 
 
 DatasetListPanel   m_DatasetListPanel  
 
 The panel for configuring selected datasets
 
 
 
 
 
 
m_NotesButton 
 
javax.swing.JButton  m_NotesButton  
 
 A button for bringing up the notes
 
 
 
 
 
 
m_NotesFrame 
 
javax.swing.JFrame  m_NotesFrame  
 
 Frame for the notes
 
 
 
 
 
 
m_NotesText 
 
javax.swing.JTextArea  m_NotesText  
 
 Area for user notes Default of 10 rows
 
 
 
 
 
 
m_Support 
 
java.beans.PropertyChangeSupport  m_Support  
 
 Manages sending notifications to people when we change the experiment,
 at this stage, only the resultlistener so the resultpanel can update.
 
 
 
 
 
 
m_advanceDataSetFirst 
 
javax.swing.JRadioButton  m_advanceDataSetFirst  
 
 Click to advacne data set before custom generator
 
 
 
 
 
 
m_advanceIteratorFirst 
 
javax.swing.JRadioButton  m_advanceIteratorFirst  
 
 Click to advance custom generator before data set
 
 
 
 
 
 
m_RadioListener 
 
java.awt.event.ActionListener  m_RadioListener  
 
 Handle radio buttons
 
 
 
 

 
   
 
 
  
 Class  weka.gui.experiment.SimpleSetupPanel  extends javax.swing.JPanel implements Serializable   
 
 

 
 serialVersionUID:  5257424515609176509L

 
   
 
 
  
 Serialized Fields   
 
 

 
m_Exp 
 
 Experiment   m_Exp  
 
 The experiment being configured
 
 
 
 
 
 
m_modePanel 
 
 SetupModePanel   m_modePanel  
 
 The panel which switched between simple and advanced setup modes
 
 
 
 
 
 
m_destinationDatabaseURL 
 
java.lang.String  m_destinationDatabaseURL  
 
 The database destination URL to store results into
 
 
 
 
 
 
m_destinationFilename 
 
java.lang.String  m_destinationFilename  
 
 The filename to store results into
 
 
 
 
 
 
m_numFolds 
 
int  m_numFolds  
 
 The number of folds for a cross-validation experiment
 
 
 
 
 
 
m_trainPercent 
 
double  m_trainPercent  
 
 The training percentage for a train/test split experiment
 
 
 
 
 
 
m_numRepetitions 
 
int  m_numRepetitions  
 
 The number of times to repeat the sub-experiment
 
 
 
 
 
 
m_userHasBeenAskedAboutConversion 
 
boolean  m_userHasBeenAskedAboutConversion  
 
 Whether or not the user has consented for the experiment to be simplified
 
 
 
 
 
 
m_csvFileFilter 
 
 ExtensionFileFilter   m_csvFileFilter  
 
 Filter for choosing CSV files
 
 
 
 
 
 
m_arffFileFilter 
 
 ExtensionFileFilter   m_arffFileFilter  
 
 FIlter for choosing ARFF files
 
 
 
 
 
 
m_OpenBut 
 
javax.swing.JButton  m_OpenBut  
 
 Click to load an experiment
 
 
 
 
 
 
m_SaveBut 
 
javax.swing.JButton  m_SaveBut  
 
 Click to save an experiment
 
 
 
 
 
 
m_NewBut 
 
javax.swing.JButton  m_NewBut  
 
 Click to create a new experiment with default settings
 
 
 
 
 
 
m_ExpFilter 
 
javax.swing.filechooser.FileFilter  m_ExpFilter  
 
 A filter to ensure only experiment files get shown in the chooser
 
 
 
 
 
 
m_KOMLFilter 
 
javax.swing.filechooser.FileFilter  m_KOMLFilter  
 
 A filter to ensure only experiment (in KOML format) files get shown in the chooser
 
 
 
 
 
 
m_XMLFilter 
 
javax.swing.filechooser.FileFilter  m_XMLFilter  
 
 A filter to ensure only experiment (in XML format) files get shown in the chooser
 
 
 
 
 
 
m_FileChooser 
 
javax.swing.JFileChooser  m_FileChooser  
 
 The file chooser for selecting experiments
 
 
 
 
 
 
m_DestFileChooser 
 
javax.swing.JFileChooser  m_DestFileChooser  
 
 The file chooser for selecting result destinations
 
 
 
 
 
 
m_ResultsDestinationCBox 
 
javax.swing.JComboBox  m_ResultsDestinationCBox  
 
 Combo box for choosing experiment destination type
 
 
 
 
 
 
m_ResultsDestinationPathLabel 
 
javax.swing.JLabel  m_ResultsDestinationPathLabel  
 
 Label for destination field
 
 
 
 
 
 
m_ResultsDestinationPathTField 
 
javax.swing.JTextField  m_ResultsDestinationPathTField  
 
 Input field for result destination path
 
 
 
 
 
 
m_BrowseDestinationButton 
 
javax.swing.JButton  m_BrowseDestinationButton  
 
 Button for browsing destination files
 
 
 
 
 
 
m_ExperimentTypeCBox 
 
javax.swing.JComboBox  m_ExperimentTypeCBox  
 
 Combo box for choosing experiment type
 
 
 
 
 
 
m_ExperimentParameterLabel 
 
javax.swing.JLabel  m_ExperimentParameterLabel  
 
 Label for parameter field
 
 
 
 
 
 
m_ExperimentParameterTField 
 
javax.swing.JTextField  m_ExperimentParameterTField  
 
 Input field for experiment parameter
 
 
 
 
 
 
m_ExpClassificationRBut 
 
javax.swing.JRadioButton  m_ExpClassificationRBut  
 
 Radio button for choosing classification experiment
 
 
 
 
 
 
m_ExpRegressionRBut 
 
javax.swing.JRadioButton  m_ExpRegressionRBut  
 
 Radio button for choosing regression experiment
 
 
 
 
 
 
m_NumberOfRepetitionsTField 
 
javax.swing.JTextField  m_NumberOfRepetitionsTField  
 
 Input field for number of repetitions
 
 
 
 
 
 
m_OrderDatasetsFirstRBut 
 
javax.swing.JRadioButton  m_OrderDatasetsFirstRBut  
 
 Radio button for choosing datasets first in order of execution
 
 
 
 
 
 
m_OrderAlgorithmsFirstRBut 
 
javax.swing.JRadioButton  m_OrderAlgorithmsFirstRBut  
 
 Radio button for choosing algorithms first in order of execution
 
 
 
 
 
 
m_DatasetListPanel 
 
 DatasetListPanel   m_DatasetListPanel  
 
 The panel for configuring selected datasets
 
 
 
 
 
 
m_AlgorithmListPanel 
 
 AlgorithmListPanel   m_AlgorithmListPanel  
 
 The panel for configuring selected algorithms
 
 
 
 
 
 
m_NotesButton 
 
javax.swing.JButton  m_NotesButton  
 
 A button for bringing up the notes
 
 
 
 
 
 
m_NotesFrame 
 
javax.swing.JFrame  m_NotesFrame  
 
 Frame for the notes
 
 
 
 
 
 
m_NotesText 
 
javax.swing.JTextArea  m_NotesText  
 
 Area for user notes Default of 10 rows
 
 
 
 
 
 
m_Support 
 
java.beans.PropertyChangeSupport  m_Support  
 
 Manages sending notifications to people when we change the experiment,
 at this stage, only the resultlistener so the resultpanel can update.
 
 
 
 
 

 
 
  
 Package   weka.gui.explorer   
 
 

 
   
 
 
  
 Class  weka.gui.explorer.AssociationsPanel  extends javax.swing.JPanel implements Serializable   
 
 

 
 serialVersionUID:  -6867871711865476971L

 
   
 
 
  
 Serialized Fields   
 
 

 
m_Explorer 
 
 Explorer   m_Explorer  
 
 the parent frame
 
 
 
 
 
 
m_AssociatorEditor 
 
 GenericObjectEditor   m_AssociatorEditor  
 
 Lets the user configure the associator
 
 
 
 
 
 
m_CEPanel 
 
 PropertyPanel   m_CEPanel  
 
 The panel showing the current associator selection
 
 
 
 
 
 
m_OutText 
 
javax.swing.JTextArea  m_OutText  
 
 The output area for associations
 
 
 
 
 
 
m_Log 
 
 Logger   m_Log  
 
 The destination for log/status messages
 
 
 
 
 
 
m_SaveOut 
 
 SaveBuffer   m_SaveOut  
 
 The buffer saving object for saving output
 
 
 
 
 
 
m_History 
 
 ResultHistoryPanel   m_History  
 
 A panel controlling results viewing
 
 
 
 
 
 
m_StartBut 
 
javax.swing.JButton  m_StartBut  
 
 Click to start running the associator
 
 
 
 
 
 
m_StopBut 
 
javax.swing.JButton  m_StopBut  
 
 Click to stop a running associator
 
 
 
 
 
 
m_Instances 
 
 Instances   m_Instances  
 
 The main set of instances we're playing with
 
 
 
 
 
 
m_TestInstances 
 
 Instances   m_TestInstances  
 
 The user-supplied test set (if any)
 
 
 
 
 
 
m_RunThread 
 
java.lang.Thread  m_RunThread  
 
 A thread that associator runs in
 
 
 
 

 
   
 
 
  
 Class  weka.gui.explorer.AttributeSelectionPanel  extends javax.swing.JPanel implements Serializable   
 
 

 
 serialVersionUID:  5627185966993476142L

 
   
 
 
  
 Serialized Fields   
 
 

 
m_Explorer 
 
 Explorer   m_Explorer  
 
 the parent frame
 
 
 
 
 
 
m_AttributeEvaluatorEditor 
 
 GenericObjectEditor   m_AttributeEvaluatorEditor  
 
 Lets the user configure the attribute evaluator
 
 
 
 
 
 
m_AttributeSearchEditor 
 
 GenericObjectEditor   m_AttributeSearchEditor  
 
 Lets the user configure the search method
 
 
 
 
 
 
m_AEEPanel 
 
 PropertyPanel   m_AEEPanel  
 
 The panel showing the current attribute evaluation method
 
 
 
 
 
 
m_ASEPanel 
 
 PropertyPanel   m_ASEPanel  
 
 The panel showing the current search method
 
 
 
 
 
 
m_OutText 
 
javax.swing.JTextArea  m_OutText  
 
 The output area for attribute selection results
 
 
 
 
 
 
m_Log 
 
 Logger   m_Log  
 
 The destination for log/status messages
 
 
 
 
 
 
m_SaveOut 
 
 SaveBuffer   m_SaveOut  
 
 The buffer saving object for saving output
 
 
 
 
 
 
m_History 
 
 ResultHistoryPanel   m_History  
 
 A panel controlling results viewing
 
 
 
 
 
 
m_ClassCombo 
 
javax.swing.JComboBox  m_ClassCombo  
 
 Lets the user select the class column
 
 
 
 
 
 
m_CVBut 
 
javax.swing.JRadioButton  m_CVBut  
 
 Click to set evaluation mode to cross-validation
 
 
 
 
 
 
m_TrainBut 
 
javax.swing.JRadioButton  m_TrainBut  
 
 Click to set test mode to test on training data
 
 
 
 
 
 
m_CVLab 
 
javax.swing.JLabel  m_CVLab  
 
 Label by where the cv folds are entered
 
 
 
 
 
 
m_CVText 
 
javax.swing.JTextField  m_CVText  
 
 The field where the cv folds are entered
 
 
 
 
 
 
m_SeedLab 
 
javax.swing.JLabel  m_SeedLab  
 
 Label by where cv random seed is entered
 
 
 
 
 
 
m_SeedText 
 
javax.swing.JTextField  m_SeedText  
 
 The field where the seed value is entered
 
 
 
 
 
 
m_RadioListener 
 
java.awt.event.ActionListener  m_RadioListener  
 
 Alters the enabled/disabled status of elements associated with each
 radio button
 
 
 
 
 
 
m_StartBut 
 
javax.swing.JButton  m_StartBut  
 
 Click to start running the attribute selector
 
 
 
 
 
 
m_StopBut 
 
javax.swing.JButton  m_StopBut  
 
 Click to stop a running classifier
 
 
 
 
 
 
COMBO_SIZE 
 
java.awt.Dimension  COMBO_SIZE  
 
 Stop the class combo from taking up to much space
 
 
 
 
 
 
m_Instances 
 
 Instances   m_Instances  
 
 The main set of instances we're playing with
 
 
 
 
 
 
m_RunThread 
 
java.lang.Thread  m_RunThread  
 
 A thread that attribute selection runs in
 
 
 
 

 
   
 
 
  
 Class  weka.gui.explorer.ClassifierPanel  extends javax.swing.JPanel implements Serializable   
 
 

 
 serialVersionUID:  6959973704963624003L

 
   
 
 
  
 Serialized Fields   
 
 

 
m_Explorer 
 
 Explorer   m_Explorer  
 
 the parent frame
 
 
 
 
 
 
m_ClassifierEditor 
 
 GenericObjectEditor   m_ClassifierEditor  
 
 Lets the user configure the classifier
 
 
 
 
 
 
m_CEPanel 
 
 PropertyPanel   m_CEPanel  
 
 The panel showing the current classifier selection
 
 
 
 
 
 
m_OutText 
 
javax.swing.JTextArea  m_OutText  
 
 The output area for classification results
 
 
 
 
 
 
m_Log 
 
 Logger   m_Log  
 
 The destination for log/status messages
 
 
 
 
 
 
m_SaveOut 
 
 SaveBuffer   m_SaveOut  
 
 The buffer saving object for saving output
 
 
 
 
 
 
m_History 
 
 ResultHistoryPanel   m_History  
 
 A panel controlling results viewing
 
 
 
 
 
 
m_ClassCombo 
 
javax.swing.JComboBox  m_ClassCombo  
 
 Lets the user select the class column
 
 
 
 
 
 
m_CVBut 
 
javax.swing.JRadioButton  m_CVBut  
 
 Click to set test mode to cross-validation
 
 
 
 
 
 
m_PercentBut 
 
javax.swing.JRadioButton  m_PercentBut  
 
 Click to set test mode to generate a % split
 
 
 
 
 
 
m_TrainBut 
 
javax.swing.JRadioButton  m_TrainBut  
 
 Click to set test mode to test on training data
 
 
 
 
 
 
m_TestSplitBut 
 
javax.swing.JRadioButton  m_TestSplitBut  
 
 Click to set test mode to a user-specified test set
 
 
 
 
 
 
m_StorePredictionsBut 
 
javax.swing.JCheckBox  m_StorePredictionsBut  
 
 Check to save the predictions in the results list for visualizing
      later on
 
 
 
 
 
 
m_OutputModelBut 
 
javax.swing.JCheckBox  m_OutputModelBut  
 
 Check to output the model built from the training data
 
 
 
 
 
 
m_OutputPerClassBut 
 
javax.swing.JCheckBox  m_OutputPerClassBut  
 
 Check to output true/false positives, precision/recall for each class
 
 
 
 
 
 
m_OutputConfusionBut 
 
javax.swing.JCheckBox  m_OutputConfusionBut  
 
 Check to output a confusion matrix
 
 
 
 
 
 
m_OutputEntropyBut 
 
javax.swing.JCheckBox  m_OutputEntropyBut  
 
 Check to output entropy statistics
 
 
 
 
 
 
m_OutputPredictionsTextBut 
 
javax.swing.JCheckBox  m_OutputPredictionsTextBut  
 
 Check to output text predictions
 
 
 
 
 
 
m_OutputAdditionalAttributesText 
 
javax.swing.JTextField  m_OutputAdditionalAttributesText  
 
 Lists indices for additional attributes to output
 
 
 
 
 
 
m_OutputAdditionalAttributesLab 
 
javax.swing.JLabel  m_OutputAdditionalAttributesLab  
 
 Label for the text field with additional attributes in the output
 
 
 
 
 
 
m_OutputAdditionalAttributesRange 
 
 Range   m_OutputAdditionalAttributesRange  
 
 the range of attributes to output
 
 
 
 
 
 
m_EvalWRTCostsBut 
 
javax.swing.JCheckBox  m_EvalWRTCostsBut  
 
 Check to evaluate w.r.t a cost matrix
 
 
 
 
 
 
m_SetCostsBut 
 
javax.swing.JButton  m_SetCostsBut  
 
 for the cost matrix
 
 
 
 
 
 
m_CVLab 
 
javax.swing.JLabel  m_CVLab  
 
 Label by where the cv folds are entered
 
 
 
 
 
 
m_CVText 
 
javax.swing.JTextField  m_CVText  
 
 The field where the cv folds are entered
 
 
 
 
 
 
m_PercentLab 
 
javax.swing.JLabel  m_PercentLab  
 
 Label by where the % split is entered
 
 
 
 
 
 
m_PercentText 
 
javax.swing.JTextField  m_PercentText  
 
 The field where the % split is entered
 
 
 
 
 
 
m_SetTestBut 
 
javax.swing.JButton  m_SetTestBut  
 
 The button used to open a separate test dataset
 
 
 
 
 
 
m_SetTestFrame 
 
javax.swing.JFrame  m_SetTestFrame  
 
 The frame used to show the test set selection panel
 
 
 
 
 
 
m_SetCostsFrame 
 
 PropertyDialog   m_SetCostsFrame  
 
 The frame used to show the cost matrix editing panel
 
 
 
 
 
 
m_RadioListener 
 
java.awt.event.ActionListener  m_RadioListener  
 
 Alters the enabled/disabled status of elements associated with each
 radio button
 
 
 
 
 
 
m_MoreOptions 
 
javax.swing.JButton  m_MoreOptions  
 
 Button for further output/visualize options
 
 
 
 
 
 
m_RandomSeedText 
 
javax.swing.JTextField  m_RandomSeedText  
 
 User specified random seed for cross validation or % split
 
 
 
 
 
 
m_RandomLab 
 
javax.swing.JLabel  m_RandomLab  
 
 the label for the random seed textfield
 
 
 
 
 
 
m_PreserveOrderBut 
 
javax.swing.JCheckBox  m_PreserveOrderBut  
 
 Whether randomization is turned off to preserve order
 
 
 
 
 
 
m_OutputSourceCode 
 
javax.swing.JCheckBox  m_OutputSourceCode  
 
 Whether to output the source code (only for classifiers importing Sourcable)
 
 
 
 
 
 
m_SourceCodeClass 
 
javax.swing.JTextField  m_SourceCodeClass  
 
 The name of the generated class (only applicable to Sourcable schemes)
 
 
 
 
 
 
m_StartBut 
 
javax.swing.JButton  m_StartBut  
 
 Click to start running the classifier
 
 
 
 
 
 
m_StopBut 
 
javax.swing.JButton  m_StopBut  
 
 Click to stop a running classifier
 
 
 
 
 
 
COMBO_SIZE 
 
java.awt.Dimension  COMBO_SIZE  
 
 Stop the class combo from taking up to much space
 
 
 
 
 
 
m_CostMatrixEditor 
 
 CostMatrixEditor   m_CostMatrixEditor  
 
 The cost matrix editor for evaluation costs
 
 
 
 
 
 
m_Instances 
 
 Instances   m_Instances  
 
 The main set of instances we're playing with
 
 
 
 
 
 
m_TestLoader 
 
 Loader   m_TestLoader  
 
 The loader used to load the user-supplied test set (if any)
 
 
 
 
 
 
m_RunThread 
 
java.lang.Thread  m_RunThread  
 
 A thread that classification runs in
 
 
 
 
 
 
m_CurrentVis 
 
 VisualizePanel   m_CurrentVis  
 
 The current visualization object
 
 
 
 
 
 
m_ModelFilter 
 
javax.swing.filechooser.FileFilter  m_ModelFilter  
 
 Filter to ensure only model files are selected
 
 
 
 
 
 
m_PMMLModelFilter 
 
javax.swing.filechooser.FileFilter  m_PMMLModelFilter  
 
 
 
 
 
 
m_FileChooser 
 
javax.swing.JFileChooser  m_FileChooser  
 
 The file chooser for selecting model files
 
 
 
 

 
   
 
 
  
 Class  weka.gui.explorer.ClustererPanel  extends javax.swing.JPanel implements Serializable   
 
 

 
 serialVersionUID:  -2474932792950820990L

 
   
 
 
  
 Serialized Fields   
 
 

 
m_Explorer 
 
 Explorer   m_Explorer  
 
 the parent frame
 
 
 
 
 
 
m_ClustererEditor 
 
 GenericObjectEditor   m_ClustererEditor  
 
 Lets the user configure the clusterer
 
 
 
 
 
 
m_CLPanel 
 
 PropertyPanel   m_CLPanel  
 
 The panel showing the current clusterer selection
 
 
 
 
 
 
m_OutText 
 
javax.swing.JTextArea  m_OutText  
 
 The output area for classification results
 
 
 
 
 
 
m_Log 
 
 Logger   m_Log  
 
 The destination for log/status messages
 
 
 
 
 
 
m_SaveOut 
 
 SaveBuffer   m_SaveOut  
 
 The buffer saving object for saving output
 
 
 
 
 
 
m_History 
 
 ResultHistoryPanel   m_History  
 
 A panel controlling results viewing
 
 
 
 
 
 
m_PercentBut 
 
javax.swing.JRadioButton  m_PercentBut  
 
 Click to set test mode to generate a % split
 
 
 
 
 
 
m_TrainBut 
 
javax.swing.JRadioButton  m_TrainBut  
 
 Click to set test mode to test on training data
 
 
 
 
 
 
m_TestSplitBut 
 
javax.swing.JRadioButton  m_TestSplitBut  
 
 Click to set test mode to a user-specified test set
 
 
 
 
 
 
m_ClassesToClustersBut 
 
javax.swing.JRadioButton  m_ClassesToClustersBut  
 
 Click to set test mode to classes to clusters based evaluation
 
 
 
 
 
 
m_ClassCombo 
 
javax.swing.JComboBox  m_ClassCombo  
 
 Lets the user select the class column for classes to clusters based
      evaluation
 
 
 
 
 
 
m_PercentLab 
 
javax.swing.JLabel  m_PercentLab  
 
 Label by where the % split is entered
 
 
 
 
 
 
m_PercentText 
 
javax.swing.JTextField  m_PercentText  
 
 The field where the % split is entered
 
 
 
 
 
 
m_SetTestBut 
 
javax.swing.JButton  m_SetTestBut  
 
 The button used to open a separate test dataset
 
 
 
 
 
 
m_SetTestFrame 
 
javax.swing.JFrame  m_SetTestFrame  
 
 The frame used to show the test set selection panel
 
 
 
 
 
 
m_ignoreBut 
 
javax.swing.JButton  m_ignoreBut  
 
 The button used to popup a list for choosing attributes to ignore while
      clustering
 
 
 
 
 
 
m_ignoreKeyModel 
 
javax.swing.DefaultListModel  m_ignoreKeyModel  
 
 
 
 
 
 
m_ignoreKeyList 
 
javax.swing.JList  m_ignoreKeyList  
 
 
 
 
 
 
m_RadioListener 
 
java.awt.event.ActionListener  m_RadioListener  
 
 Alters the enabled/disabled status of elements associated with each
 radio button
 
 
 
 
 
 
m_StartBut 
 
javax.swing.JButton  m_StartBut  
 
 Click to start running the clusterer
 
 
 
 
 
 
COMBO_SIZE 
 
java.awt.Dimension  COMBO_SIZE  
 
 Stop the class combo from taking up to much space
 
 
 
 
 
 
m_StopBut 
 
javax.swing.JButton  m_StopBut  
 
 Click to stop a running clusterer
 
 
 
 
 
 
m_Instances 
 
 Instances   m_Instances  
 
 The main set of instances we're playing with
 
 
 
 
 
 
m_TestInstances 
 
 Instances   m_TestInstances  
 
 The user-supplied test set (if any)
 
 
 
 
 
 
m_CurrentVis 
 
 VisualizePanel   m_CurrentVis  
 
 The current visualization object
 
 
 
 
 
 
m_StorePredictionsBut 
 
javax.swing.JCheckBox  m_StorePredictionsBut  
 
 Check to save the predictions in the results list for visualizing
      later on
 
 
 
 
 
 
m_RunThread 
 
java.lang.Thread  m_RunThread  
 
 A thread that clustering runs in
 
 
 
 
 
 
m_Summary 
 
 InstancesSummaryPanel   m_Summary  
 
 The instances summary panel displayed by m_SetTestFrame
 
 
 
 
 
 
m_ModelFilter 
 
javax.swing.filechooser.FileFilter  m_ModelFilter  
 
 Filter to ensure only model files are selected
 
 
 
 
 
 
m_FileChooser 
 
javax.swing.JFileChooser  m_FileChooser  
 
 The file chooser for selecting model files
 
 
 
 

 
   
 
 
  
 Class  weka.gui.explorer.DataGeneratorPanel  extends javax.swing.JPanel implements Serializable   
 
 

 
 serialVersionUID:  -2520408165350629380L

 
   
 
 
  
 Serialized Fields   
 
 

 
m_GeneratorEditor 
 
 GenericObjectEditor   m_GeneratorEditor  
 
 the GOE for the generators
 
 
 
 
 
 
m_Instances 
 
 Instances   m_Instances  
 
 the generated Instances
 
 
 
 
 
 
m_Output 
 
java.io.StringWriter  m_Output  
 
 the generated output (as text)
 
 
 
 
 
 
m_Log 
 
 Logger   m_Log  
 
 The destination for log/status messages
 
 
 
 

 
   
 
 
  
 Class  weka.gui.explorer.Explorer  extends javax.swing.JPanel implements Serializable   
 
 

 
 serialVersionUID:  -7674003708867909578L

 
   
 
 
  
 Serialized Fields   
 
 

 
m_PreprocessPanel 
 
 PreprocessPanel   m_PreprocessPanel  
 
 The panel for preprocessing instances
 
 
 
 
 
 
m_Panels 
 
java.util.Vector<E>  m_Panels  
 
 Contains all the additional panels apart from the pre-processing panel
 
 
 
 
 
 
m_TabbedPane 
 
javax.swing.JTabbedPane  m_TabbedPane  
 
 The tabbed pane that controls which sub-pane we are working with
 
 
 
 
 
 
m_LogPanel 
 
 LogPanel   m_LogPanel  
 
 The panel for log and status messages
 
 
 
 
 
 
m_CapabilitiesFilterChangeListeners 
 
java.util.HashSet<E>  m_CapabilitiesFilterChangeListeners  
 
 the listeners that listen to filter changes
 
 
 
 

 
   
 
 
  
 Class  weka.gui.explorer.Explorer.CapabilitiesFilterChangeEvent  extends javax.swing.event.ChangeEvent implements Serializable   
 
 

 
 serialVersionUID:  1194260517270385559L

 
   
 
 
  
 Serialized Fields   
 
 

 
m_Filter 
 
 Capabilities   m_Filter  
 
 the capabilities filter
 
 
 
 

 
   
 
 
  
 Class  weka.gui.explorer.ExplorerDefaults  extends java.lang.Object implements Serializable   
 
 

 
 serialVersionUID:  4954795757927524225L

 

 
   
 
 
  
 Class  weka.gui.explorer.PreprocessPanel  extends javax.swing.JPanel implements Serializable   
 
 

 
 serialVersionUID:  6764850273874813049L

 
   
 
 
  
 Serialized Fields   
 
 

 
m_InstSummaryPanel 
 
 InstancesSummaryPanel   m_InstSummaryPanel  
 
 Displays simple stats on the working instances
 
 
 
 
 
 
m_OpenFileBut 
 
javax.swing.JButton  m_OpenFileBut  
 
 Click to load base instances from a file
 
 
 
 
 
 
m_OpenURLBut 
 
javax.swing.JButton  m_OpenURLBut  
 
 Click to load base instances from a URL
 
 
 
 
 
 
m_OpenDBBut 
 
javax.swing.JButton  m_OpenDBBut  
 
 Click to load base instances from a Database
 
 
 
 
 
 
m_GenerateBut 
 
javax.swing.JButton  m_GenerateBut  
 
 Click to generate artificial data
 
 
 
 
 
 
m_UndoBut 
 
javax.swing.JButton  m_UndoBut  
 
 Click to revert back to the last saved point
 
 
 
 
 
 
m_EditBut 
 
javax.swing.JButton  m_EditBut  
 
 Click to open the current instances in a viewer
 
 
 
 
 
 
m_SaveBut 
 
javax.swing.JButton  m_SaveBut  
 
 Click to apply filters and save the results
 
 
 
 
 
 
m_AttPanel 
 
 AttributeSelectionPanel   m_AttPanel  
 
 Panel to let the user toggle attributes
 
 
 
 
 
 
m_RemoveButton 
 
javax.swing.JButton  m_RemoveButton  
 
 Button for removing attributes
 
 
 
 
 
 
m_AttSummaryPanel 
 
 AttributeSummaryPanel   m_AttSummaryPanel  
 
 Displays summary stats on the selected attribute
 
 
 
 
 
 
m_FilterEditor 
 
 GenericObjectEditor   m_FilterEditor  
 
 Lets the user configure the filter
 
 
 
 
 
 
m_FilterPanel 
 
 PropertyPanel   m_FilterPanel  
 
 Filter configuration
 
 
 
 
 
 
m_ApplyFilterBut 
 
javax.swing.JButton  m_ApplyFilterBut  
 
 Click to apply filters and save the results
 
 
 
 
 
 
m_FileChooser 
 
 ConverterFileChooser   m_FileChooser  
 
 The file chooser for selecting data files
 
 
 
 
 
 
m_LastURL 
 
java.lang.String  m_LastURL  
 
 Stores the last URL that instances were loaded from
 
 
 
 
 
 
m_SQLQ 
 
java.lang.String  m_SQLQ  
 
 Stores the last sql query executed
 
 
 
 
 
 
m_Instances 
 
 Instances   m_Instances  
 
 The working instances
 
 
 
 
 
 
m_DataGenerator 
 
 DataGenerator   m_DataGenerator  
 
 The last generator that was selected
 
 
 
 
 
 
m_AttVisualizePanel 
 
 AttributeVisualizationPanel   m_AttVisualizePanel  
 
 The visualization of the attribute values
 
 
 
 
 
 
m_tempUndoFiles 
 
java.io.File[]  m_tempUndoFiles  
 
 Keeps track of undo points
 
 
 
 
 
 
m_tempUndoIndex 
 
int  m_tempUndoIndex  
 
 The next available slot for an undo point
 
 
 
 
 
 
m_Support 
 
java.beans.PropertyChangeSupport  m_Support  
 
 Manages sending notifications to people when we change the set of
 working instances.
 
 
 
 
 
 
m_IOThread 
 
java.lang.Thread  m_IOThread  
 
 A thread for loading/saving instances from a file or URL
 
 
 
 
 
 
m_Log 
 
 Logger   m_Log  
 
 The message logger
 
 
 
 
 
 
m_Explorer 
 
 Explorer   m_Explorer  
 
 the parent frame
 
 
 
 

 
   
 
 
  
 Class  weka.gui.explorer.VisualizePanel  extends  MatrixPanel  implements Serializable   
 
 

 
 serialVersionUID:  6084015036853918846L

 
   
 
 
  
 Serialized Fields   
 
 

 
m_Explorer 
 
 Explorer   m_Explorer  
 
 the parent frame
 
 
 
 
 

 
 
  
 Package   weka.gui.graphvisualizer   
 
 

 
   
 
 
  
 Class  weka.gui.graphvisualizer.BIFFormatException  extends java.lang.Exception implements Serializable   
 
 

 
 serialVersionUID:  -4102518086411708140L

 

 
   
 
 
  
 Class  weka.gui.graphvisualizer.GraphVisualizer  extends javax.swing.JPanel implements Serializable   
 
 

 
 serialVersionUID:  -2038911085935515624L

 
   
 
 
  
 Serialized Fields   
 
 

 
m_nodes 
 
 FastVector   m_nodes  
 
 Vector containing nodes
 
 
 
 
 
 
m_edges 
 
 FastVector   m_edges  
 
 Vector containing edges
 
 
 
 
 
 
m_le 
 
 LayoutEngine   m_le  
 
 The current LayoutEngine
 
 
 
 
 
 
m_gp 
 
weka.gui.graphvisualizer.GraphVisualizer.GraphPanel  m_gp  
 
 Panel actually displaying the graph
 
 
 
 
 
 
graphID 
 
java.lang.String  graphID  
 
 String containing graph's name
 
 
 
 
 
 
m_jBtSave 
 
javax.swing.JButton  m_jBtSave  
 
 Save button to save the current graph in DOT or XMLBIF format.
 The graph should be layed out again to get the original form
 if reloaded from command line, as the formats do not allow
 saving specific information for a properly layed out graph.
 
 
 
 
 
 
ICONPATH 
 
java.lang.String  ICONPATH  
 
 path for icons
 
 
 
 
 
 
fm 
 
java.awt.FontMetrics  fm  
 
 
 
 
 
 
scale 
 
double  scale  
 
 
 
 
 
 
nodeHeight 
 
int  nodeHeight  
 
 
 
 
 
 
nodeWidth 
 
int  nodeWidth  
 
 
 
 
 
 
paddedNodeWidth 
 
int  paddedNodeWidth  
 
 
 
 
 
 
jTfNodeWidth 
 
javax.swing.JTextField  jTfNodeWidth  
 
 TextField for node's width
 
 
 
 
 
 
jTfNodeHeight 
 
javax.swing.JTextField  jTfNodeHeight  
 
 TextField for nodes height
 
 
 
 
 
 
jBtLayout 
 
javax.swing.JButton  jBtLayout  
 
 Button for laying out the graph again, necessary after changing node's
 size or some other property of the layout engine
 
 
 
 
 
 
maxStringWidth 
 
int  maxStringWidth  
 
 used for setting appropriate node size
 
 
 
 
 
 
zoomPercents 
 
int[]  zoomPercents  
 
 used when using zoomIn and zoomOut buttons
 
 
 
 
 
 
m_js 
 
javax.swing.JScrollPane  m_js  
 
 this contains the m_gp GraphPanel
 
 
 
 

 
   
 
 
  
 Class  weka.gui.graphvisualizer.LayoutCompleteEvent  extends java.util.EventObject implements Serializable   
 
 

 
 serialVersionUID:  6172467234026258427L

 
 

 
 
  
 Package   weka.gui.hierarchyvisualizer   
 
 

 
   
 
 
  
 Class  weka.gui.hierarchyvisualizer.HierarchyVisualizer  extends  PrintablePanel  implements Serializable   
 
 

 
 serialVersionUID:  1L

 
   
 
 
  
 Serialized Fields   
 
 

 
m_sNewick 
 
java.lang.String  m_sNewick  
 
 
 
 
 
 
m_tree 
 
weka.gui.hierarchyvisualizer.HierarchyVisualizer.Node  m_tree  
 
 
 
 
 
 
m_nLeafs 
 
int  m_nLeafs  
 
 
 
 
 
 
m_fHeight 
 
double  m_fHeight  
 
 
 
 
 
 
m_fScaleX 
 
double  m_fScaleX  
 
 
 
 
 
 
m_fScaleY 
 
double  m_fScaleY  
 
 
 
 
 
 
m_fTmpLength 
 
double  m_fTmpLength  
 
 
 
 
 

 
 
  
 Package   weka.gui.sql   
 
 

 
   
 
 
  
 Class  weka.gui.sql.ConnectionPanel  extends javax.swing.JPanel implements Serializable   
 
 

 
 serialVersionUID:  3499317023969723490L

 
   
 
 
  
 Serialized Fields   
 
 

 
m_Parent 
 
javax.swing.JFrame  m_Parent  
 
 the parent frame.
 
 
 
 
 
 
m_DbDialog 
 
 DatabaseConnectionDialog   m_DbDialog  
 
 the databae connection dialog.
 
 
 
 
 
 
m_URL 
 
java.lang.String  m_URL  
 
 the URL to use.
 
 
 
 
 
 
m_User 
 
java.lang.String  m_User  
 
 the user to use for connecting to the DB.
 
 
 
 
 
 
m_Password 
 
java.lang.String  m_Password  
 
 the password to use for connecting to the DB.
 
 
 
 
 
 
m_LabelURL 
 
javax.swing.JLabel  m_LabelURL  
 
 the label for the URL.
 
 
 
 
 
 
m_TextURL 
 
javax.swing.JTextField  m_TextURL  
 
 the textfield for the URL.
 
 
 
 
 
 
m_ButtonDatabase 
 
javax.swing.JButton  m_ButtonDatabase  
 
 the button for the DB-Dialog.
 
 
 
 
 
 
m_ButtonConnect 
 
javax.swing.JButton  m_ButtonConnect  
 
 the button for connecting to the database.
 
 
 
 
 
 
m_ButtonHistory 
 
javax.swing.JButton  m_ButtonHistory  
 
 the button for the history.
 
 
 
 
 
 
m_ConnectionListeners 
 
java.util.HashSet<E>  m_ConnectionListeners  
 
 the connection listeners.
 
 
 
 
 
 
m_HistoryChangedListeners 
 
java.util.HashSet<E>  m_HistoryChangedListeners  
 
 the history listeners.
 
 
 
 
 
 
m_DbUtils 
 
 DbUtils   m_DbUtils  
 
 for connecting to the database.
 
 
 
 
 
 
m_History 
 
javax.swing.DefaultListModel  m_History  
 
 the history of connections.
 
 
 
 

 
   
 
 
  
 Class  weka.gui.sql.DbUtils  extends  DatabaseUtils  implements Serializable   
 
 

 
 serialVersionUID:  103748569037426479L

 

 
   
 
 
  
 Class  weka.gui.sql.InfoPanel  extends javax.swing.JPanel implements Serializable   
 
 

 
 serialVersionUID:  -7701133696481997973L

 
   
 
 
  
 Serialized Fields   
 
 

 
m_Parent 
 
javax.swing.JFrame  m_Parent  
 
 the parent of this panel
 
 
 
 
 
 
m_Info 
 
javax.swing.JList  m_Info  
 
 the list the contains the messages
 
 
 
 
 
 
m_Model 
 
javax.swing.DefaultListModel  m_Model  
 
 the model for the list
 
 
 
 
 
 
m_ButtonClear 
 
javax.swing.JButton  m_ButtonClear  
 
 the button to clear the area
 
 
 
 
 
 
m_ButtonCopy 
 
javax.swing.JButton  m_ButtonCopy  
 
 the button to copy the selected message
 
 
 
 

 
   
 
 
  
 Class  weka.gui.sql.InfoPanelCellRenderer  extends javax.swing.JLabel implements Serializable   
 
 

 
 serialVersionUID:  -533380118807178531L

 

 
   
 
 
  
 Class  weka.gui.sql.QueryPanel  extends javax.swing.JPanel implements Serializable   
 
 

 
 serialVersionUID:  4348967824619706636L

 
   
 
 
  
 Serialized Fields   
 
 

 
m_Parent 
 
javax.swing.JFrame  m_Parent  
 
 the parent of this panel.
 
 
 
 
 
 
m_TextQuery 
 
javax.swing.JTextArea  m_TextQuery  
 
 the textarea for the query.
 
 
 
 
 
 
m_ButtonExecute 
 
javax.swing.JButton  m_ButtonExecute  
 
 the execute button.
 
 
 
 
 
 
m_ButtonClear 
 
javax.swing.JButton  m_ButtonClear  
 
 the clear button.
 
 
 
 
 
 
m_ButtonHistory 
 
javax.swing.JButton  m_ButtonHistory  
 
 the history button.
 
 
 
 
 
 
m_SpinnerMaxRows 
 
javax.swing.JSpinner  m_SpinnerMaxRows  
 
 the spinner for the maximum number of rows.
 
 
 
 
 
 
m_QueryExecuteListeners 
 
java.util.HashSet<E>  m_QueryExecuteListeners  
 
 the connection listeners.
 
 
 
 
 
 
m_HistoryChangedListeners 
 
java.util.HashSet<E>  m_HistoryChangedListeners  
 
 the history listeners.
 
 
 
 
 
 
m_DbUtils 
 
 DbUtils   m_DbUtils  
 
 for working on the database.
 
 
 
 
 
 
m_Connected 
 
boolean  m_Connected  
 
 whether we have a connection to a database or not.
 
 
 
 
 
 
m_History 
 
javax.swing.DefaultListModel  m_History  
 
 the query history.
 
 
 
 

 
   
 
 
  
 Class  weka.gui.sql.ResultPanel  extends javax.swing.JPanel implements Serializable   
 
 

 
 serialVersionUID:  278654800344034571L

 
   
 
 
  
 Serialized Fields   
 
 

 
m_Parent 
 
javax.swing.JFrame  m_Parent  
 
 the parent of this panel
 
 
 
 
 
 
m_Listeners 
 
java.util.HashSet<E>  m_Listeners  
 
 the result change listeners
 
 
 
 
 
 
m_QueryPanel 
 
 QueryPanel   m_QueryPanel  
 
 the panel where to output the queries
 
 
 
 
 
 
m_TabbedPane 
 
javax.swing.JTabbedPane  m_TabbedPane  
 
 the tabbed pane for the results
 
 
 
 
 
 
m_ButtonClose 
 
javax.swing.JButton  m_ButtonClose  
 
 the close button
 
 
 
 
 
 
m_ButtonCloseAll 
 
javax.swing.JButton  m_ButtonCloseAll  
 
 the close all button
 
 
 
 
 
 
m_ButtonCopyQuery 
 
javax.swing.JButton  m_ButtonCopyQuery  
 
 the button that copies the query into the QueryPanel
 
 
 
 
 
 
m_ButtonOptWidth 
 
javax.swing.JButton  m_ButtonOptWidth  
 
 the button for the optimal column width of the current table
 
 
 
 
 
 
m_NameCounter 
 
int  m_NameCounter  
 
 the counter for the tab names
 
 
 
 

 
   
 
 
  
 Class  weka.gui.sql.ResultSetTable  extends javax.swing.JTable implements Serializable   
 
 

 
 serialVersionUID:  -3391076671854464137L

 
   
 
 
  
 Serialized Fields   
 
 

 
m_Query 
 
java.lang.String  m_Query  
 
 the query the table model is based on
 
 
 
 
 
 
m_URL 
 
java.lang.String  m_URL  
 
 the connect string with which the query was run
 
 
 
 
 
 
m_User 
 
java.lang.String  m_User  
 
 the user that was used to connect to the DB
 
 
 
 
 
 
m_Password 
 
java.lang.String  m_Password  
 
 the password that was used to connect to the DB
 
 
 
 

 
   
 
 
  
 Class  weka.gui.sql.ResultSetTableCellRenderer  extends javax.swing.table.DefaultTableCellRenderer implements Serializable   
 
 

 
 serialVersionUID:  -8106963669703497351L

 
   
 
 
  
 Serialized Fields   
 
 

 
missingColor 
 
java.awt.Color  missingColor  
 
 
 
 
 
 
missingColorSelected 
 
java.awt.Color  missingColorSelected  
 
 
 
 

 
   
 
 
  
 Class  weka.gui.sql.SqlViewer  extends javax.swing.JPanel implements Serializable   
 
 

 
 serialVersionUID:  -4395028775566514329L

 
   
 
 
  
 Serialized Fields   
 
 

 
m_Parent 
 
javax.swing.JFrame  m_Parent  
 
 the parent of this panel.
 
 
 
 
 
 
m_ConnectionPanel 
 
 ConnectionPanel   m_ConnectionPanel  
 
 the connection panel.
 
 
 
 
 
 
m_QueryPanel 
 
 QueryPanel   m_QueryPanel  
 
 the query panel.
 
 
 
 
 
 
m_ResultPanel 
 
 ResultPanel   m_ResultPanel  
 
 the result panel.
 
 
 
 
 
 
m_InfoPanel 
 
 InfoPanel   m_InfoPanel  
 
 the info panel.
 
 
 
 
 
 
m_URL 
 
java.lang.String  m_URL  
 
 the connect string with which the query was run.
 
 
 
 
 
 
m_User 
 
java.lang.String  m_User  
 
 the user that was used to connect to the DB.
 
 
 
 
 
 
m_Password 
 
java.lang.String  m_Password  
 
 the password that was used to connect to the DB.
 
 
 
 
 
 
m_Query 
 
java.lang.String  m_Query  
 
 the currently selected query.
 
 
 
 
 
 
m_History 
 
java.util.Properties  m_History  
 
 stores the history.
 
 
 
 

 
   
 
 
  
 Class  weka.gui.sql.SqlViewerDialog  extends javax.swing.JDialog implements Serializable   
 
 

 
 serialVersionUID:  -31619864037233099L

 
   
 
 
  
 Serialized Fields   
 
 

 
m_Parent 
 
javax.swing.JFrame  m_Parent  
 
 the parent frame
 
 
 
 
 
 
m_Viewer 
 
 SqlViewer   m_Viewer  
 
 the SQL panel
 
 
 
 
 
 
m_PanelButtons 
 
javax.swing.JPanel  m_PanelButtons  
 
 the panel for the buttons
 
 
 
 
 
 
m_ButtonOK 
 
javax.swing.JButton  m_ButtonOK  
 
 the OK button
 
 
 
 
 
 
m_ButtonCancel 
 
javax.swing.JButton  m_ButtonCancel  
 
 the Cancel button
 
 
 
 
 
 
m_LabelQuery 
 
javax.swing.JLabel  m_LabelQuery  
 
 displays the current query
 
 
 
 
 
 
m_ReturnValue 
 
int  m_ReturnValue  
 
 the return value
 
 
 
 
 
 
m_URL 
 
java.lang.String  m_URL  
 
 the connect string with which the query was run
 
 
 
 
 
 
m_User 
 
java.lang.String  m_User  
 
 the user that was used to connect to the DB
 
 
 
 
 
 
m_Password 
 
java.lang.String  m_Password  
 
 the password that was used to connect to the DB
 
 
 
 
 
 
m_Query 
 
java.lang.String  m_Query  
 
 the currently selected query
 
 
 
 
 

 
 
  
 Package   weka.gui.sql.event   
 
 

 
   
 
 
  
 Class  weka.gui.sql.event.ConnectionEvent  extends java.util.EventObject implements Serializable   
 
 

 
 serialVersionUID:  5420308930427835037L

 
   
 
 
  
 Serialized Fields   
 
 

 
m_Type 
 
int  m_Type  
 
 the type of event, CONNECT or DISCONNECT
 
 
 
 
 
 
m_DbUtils 
 
 DbUtils   m_DbUtils  
 
 the databaseutils instance reponsible for the connection
 
 
 
 
 
 
m_Exception 
 
java.lang.Exception  m_Exception  
 
 a possible exception that occurred if not successful
 
 
 
 

 
   
 
 
  
 Class  weka.gui.sql.event.HistoryChangedEvent  extends java.util.EventObject implements Serializable   
 
 

 
 serialVersionUID:  7476087315774869973L

 
   
 
 
  
 Serialized Fields   
 
 

 
m_HistoryName 
 
java.lang.String  m_HistoryName  
 
 the name of the history
 
 
 
 
 
 
m_History 
 
javax.swing.DefaultListModel  m_History  
 
 the history model
 
 
 
 

 
   
 
 
  
 Class  weka.gui.sql.event.QueryExecuteEvent  extends java.util.EventObject implements Serializable   
 
 

 
 serialVersionUID:  -5556385019954730740L

 
   
 
 
  
 Serialized Fields   
 
 

 
m_DbUtils 
 
 DbUtils   m_DbUtils  
 
 the Db utils instance for the current DB connection
 
 
 
 
 
 
m_Query 
 
java.lang.String  m_Query  
 
 the query that was executed
 
 
 
 
 
 
m_ResultSet 
 
java.sql.ResultSet  m_ResultSet  
 
 the produced ResultSet, if any
 
 
 
 
 
 
m_Exception 
 
java.lang.Exception  m_Exception  
 
 a possible exception, if the query failed
 
 
 
 
 
 
m_MaxRows 
 
int  m_MaxRows  
 
 the maximum number of rows to retrieve
 
 
 
 

 
   
 
 
  
 Class  weka.gui.sql.event.ResultChangedEvent  extends java.util.EventObject implements Serializable   
 
 

 
 serialVersionUID:  36042516077236111L

 
   
 
 
  
 Serialized Fields   
 
 

 
m_Query 
 
java.lang.String  m_Query  
 
 the query that is associated with the active result table
 
 
 
 
 
 
m_URL 
 
java.lang.String  m_URL  
 
 the connect string with which the query was run
 
 
 
 
 
 
m_User 
 
java.lang.String  m_User  
 
 the user that was used to connect to the DB
 
 
 
 
 
 
m_Password 
 
java.lang.String  m_Password  
 
 the password that was used to connect to the DB
 
 
 
 
 

 
 
  
 Package   weka.gui.streams   
 
 

 
   
 
 
  
 Class  weka.gui.streams.InstanceCounter  extends javax.swing.JPanel implements Serializable   
 
 

 
 serialVersionUID:  -6084967152645935934L

 
   
 
 
  
 Serialized Fields   
 
 

 
m_Count_Lab 
 
javax.swing.JLabel  m_Count_Lab  
 
 
 
 
 
 
m_Count 
 
int  m_Count  
 
 
 
 
 
 
m_Debug 
 
boolean  m_Debug  
 
 
 
 

 
   
 
 
  
 Class  weka.gui.streams.InstanceEvent  extends java.util.EventObject implements Serializable   
 
 

 
 serialVersionUID:  3207259868110667379L

 
   
 
 
  
 Serialized Fields   
 
 

 
m_ID 
 
int  m_ID  
 
 
 
 

 
   
 
 
  
 Class  weka.gui.streams.InstanceJoiner  extends java.lang.Object implements Serializable   
 
 

 
 serialVersionUID:  -6529972700291329656L

 
   
 
 
  
 Serialized Fields   
 
 

 
listeners 
 
java.util.Vector<E>  listeners  
 
 The listeners
 
 
 
 
 
 
b_Debug 
 
boolean  b_Debug  
 
 Debugging mode
 
 
 
 
 
 
m_InputFormat 
 
 Instances   m_InputFormat  
 
 The input format for instances
 
 
 
 
 
 
m_OutputInstance 
 
 Instance   m_OutputInstance  
 
 The current output instance
 
 
 
 
 
 
b_FirstInputFinished 
 
boolean  b_FirstInputFinished  
 
 Whether the first input batch has finished
 
 
 
 
 
 
b_SecondInputFinished 
 
boolean  b_SecondInputFinished  
 
 
 
 

 
   
 
 
  
 Class  weka.gui.streams.InstanceLoader  extends javax.swing.JPanel implements Serializable   
 
 

 
 serialVersionUID:  -8725567310271862492L

 
   
 
 
  
 Serialized Fields   
 
 

 
m_Listeners 
 
java.util.Vector<E>  m_Listeners  
 
 
 
 
 
 
m_LoaderThread 
 
java.lang.Thread  m_LoaderThread  
 
 
 
 
 
 
m_OutputInstance 
 
 Instance   m_OutputInstance  
 
 
 
 
 
 
m_OutputInstances 
 
 Instances   m_OutputInstances  
 
 
 
 
 
 
m_Debug 
 
boolean  m_Debug  
 
 
 
 
 
 
m_StartBut 
 
javax.swing.JButton  m_StartBut  
 
 
 
 
 
 
m_FileNameTex 
 
javax.swing.JTextField  m_FileNameTex  
 
 
 
 

 
   
 
 
  
 Class  weka.gui.streams.InstanceSavePanel  extends java.awt.Panel implements Serializable   
 
 

 
 serialVersionUID:  -6061005366989295026L

 
   
 
 
  
 Serialized Fields   
 
 

 
count_Lab 
 
java.awt.Label  count_Lab  
 
 
 
 
 
 
m_Count 
 
int  m_Count  
 
 
 
 
 
 
arffFile_Tex 
 
java.awt.TextField  arffFile_Tex  
 
 
 
 
 
 
b_Debug 
 
boolean  b_Debug  
 
 
 
 
 
 
outputWriter 
 
java.io.PrintWriter  outputWriter  
 
 
 
 

 
   
 
 
  
 Class  weka.gui.streams.InstanceTable  extends javax.swing.JPanel implements Serializable   
 
 

 
 serialVersionUID:  -2462533698100834803L

 
   
 
 
  
 Serialized Fields   
 
 

 
m_InstanceTable 
 
javax.swing.JTable  m_InstanceTable  
 
 
 
 
 
 
m_Debug 
 
boolean  m_Debug  
 
 
 
 
 
 
m_Clear 
 
boolean  m_Clear  
 
 
 
 
 
 
m_UpdateString 
 
java.lang.String  m_UpdateString  
 
 
 
 
 
 
m_Instances 
 
 Instances   m_Instances  
 
 
 
 

 
   
 
 
  
 Class  weka.gui.streams.InstanceViewer  extends javax.swing.JPanel implements Serializable   
 
 

 
 serialVersionUID:  -4925729441294121772L

 
   
 
 
  
 Serialized Fields   
 
 

 
m_OutputTex 
 
javax.swing.JTextArea  m_OutputTex  
 
 
 
 
 
 
m_Debug 
 
boolean  m_Debug  
 
 
 
 
 
 
m_Clear 
 
boolean  m_Clear  
 
 
 
 
 
 
m_UpdateString 
 
java.lang.String  m_UpdateString  
 
 
 
 
 

 
 
  
 Package   weka.gui.treevisualizer   
 
 

 
   
 
 
  
 Class  weka.gui.treevisualizer.TreeVisualizer  extends  PrintablePanel  implements Serializable   
 
 

 
 serialVersionUID:  -8668637962504080749L

 
   
 
 
  
 Serialized Fields   
 
 

 
m_placer 
 
 NodePlace   m_placer  
 
 The placement algorithm for the Node structure.
 
 
 
 
 
 
m_topNode 
 
 Node   m_topNode  
 
 The top Node.
 
 
 
 
 
 
m_viewPos 
 
java.awt.Dimension  m_viewPos  
 
 The postion of the view relative to the tree.
 
 
 
 
 
 
m_viewSize 
 
java.awt.Dimension  m_viewSize  
 
 The size of the tree in pixels.
 
 
 
 
 
 
m_currentFont 
 
java.awt.Font  m_currentFont  
 
 The font used to display the tree.
 
 
 
 
 
 
m_fontSize 
 
java.awt.FontMetrics  m_fontSize  
 
 The size information for the current font.
 
 
 
 
 
 
m_numNodes 
 
int  m_numNodes  
 
 The number of Nodes in the tree.
 
 
 
 
 
 
m_numLevels 
 
int  m_numLevels  
 
 The number of levels in the tree.
 
 
 
 
 
 
m_nodes 
 
weka.gui.treevisualizer.TreeVisualizer.NodeInfo[]  m_nodes  
 
 An array with the Nodes sorted into it and display information 
 about the Nodes.
 
 
 
 
 
 
m_edges 
 
weka.gui.treevisualizer.TreeVisualizer.EdgeInfo[]  m_edges  
 
 An array with the Edges sorted into it and display information 
 about the Edges.
 
 
 
 
 
 
m_frameLimiter 
 
javax.swing.Timer  m_frameLimiter  
 
 A timer to keep the frame rate constant.
 
 
 
 
 
 
m_mouseState 
 
int  m_mouseState  
 
 Describes the action the user is performing.
 
 
 
 
 
 
m_oldMousePos 
 
java.awt.Dimension  m_oldMousePos  
 
 A variable used to tag the start pos of a user action.
 
 
 
 
 
 
m_newMousePos 
 
java.awt.Dimension  m_newMousePos  
 
 A variable used to tag the most current point of a user action.
 
 
 
 
 
 
m_clickAvailable 
 
boolean  m_clickAvailable  
 
 A variable used to determine for the clicked method if any other 
 mouse state has already taken place.
 
 
 
 
 
 
m_nViewPos 
 
java.awt.Dimension  m_nViewPos  
 
 A variable used to remember the desired view pos.
 
 
 
 
 
 
m_nViewSize 
 
java.awt.Dimension  m_nViewSize  
 
 A variable used to remember the desired tree size.
 
 
 
 
 
 
m_scaling 
 
int  m_scaling  
 
 The number of frames left to calculate.
 
 
 
 
 
 
m_winMenu 
 
javax.swing.JPopupMenu  m_winMenu  
 
 A right (or middle) click popup menu.
 
 
 
 
 
 
m_topN 
 
javax.swing.JMenuItem  m_topN  
 
 An option on the win_menu
 
 
 
 
 
 
m_fitToScreen 
 
javax.swing.JMenuItem  m_fitToScreen  
 
 An option on the win_menu
 
 
 
 
 
 
m_autoScale 
 
javax.swing.JMenuItem  m_autoScale  
 
 An option on the win_menu
 
 
 
 
 
 
m_selectFont 
 
javax.swing.JMenu  m_selectFont  
 
 A sub group on the win_menu
 
 
 
 
 
 
m_selectFontGroup 
 
javax.swing.ButtonGroup  m_selectFontGroup  
 
 A grouping for the font choices
 
 
 
 
 
 
m_size24 
 
javax.swing.JRadioButtonMenuItem  m_size24  
 
 A font choice.
 
 
 
 
 
 
m_size22 
 
javax.swing.JRadioButtonMenuItem  m_size22  
 
 A font choice.
 
 
 
 
 
 
m_size20 
 
javax.swing.JRadioButtonMenuItem  m_size20  
 
 A font choice.
 
 
 
 
 
 
m_size18 
 
javax.swing.JRadioButtonMenuItem  m_size18  
 
 A font choice.
 
 
 
 
 
 
m_size16 
 
javax.swing.JRadioButtonMenuItem  m_size16  
 
 A font choice.
 
 
 
 
 
 
m_size14 
 
javax.swing.JRadioButtonMenuItem  m_size14  
 
 A font choice.
 
 
 
 
 
 
m_size12 
 
javax.swing.JRadioButtonMenuItem  m_size12  
 
 A font choice.
 
 
 
 
 
 
m_size10 
 
javax.swing.JRadioButtonMenuItem  m_size10  
 
 A font choice.
 
 
 
 
 
 
m_size8 
 
javax.swing.JRadioButtonMenuItem  m_size8  
 
 A font choice.
 
 
 
 
 
 
m_size6 
 
javax.swing.JRadioButtonMenuItem  m_size6  
 
 A font choice.
 
 
 
 
 
 
m_size4 
 
javax.swing.JRadioButtonMenuItem  m_size4  
 
 A font choice.
 
 
 
 
 
 
m_size2 
 
javax.swing.JRadioButtonMenuItem  m_size2  
 
 A font choice.
 
 
 
 
 
 
m_size1 
 
javax.swing.JRadioButtonMenuItem  m_size1  
 
 A font choice.
 
 
 
 
 
 
m_accept 
 
javax.swing.JMenuItem  m_accept  
 
 An option on the win menu.
 
 
 
 
 
 
m_nodeMenu 
 
javax.swing.JPopupMenu  m_nodeMenu  
 
 A right or middle click popup menu for nodes.
 
 
 
 
 
 
m_visualise 
 
javax.swing.JMenuItem  m_visualise  
 
 A visualize choice for the node, may not be available.
 
 
 
 
 
 
m_addChildren 
 
javax.swing.JMenuItem  m_addChildren  
 
 An add children to Node choice, This is only available if the tree
 display has a treedisplay listerner added to it.
 
 
 
 
 
 
m_remChildren 
 
javax.swing.JMenuItem  m_remChildren  
 
 Similar to add children but now it removes children.
 
 
 
 
 
 
m_classifyChild 
 
javax.swing.JMenuItem  m_classifyChild  
 
 Use this to have J48 classify this node.
 
 
 
 
 
 
m_sendInstances 
 
javax.swing.JMenuItem  m_sendInstances  
 
 Use this to dump the instances from this node to the vis panel.
 
 
 
 
 
 
m_focusNode 
 
int  m_focusNode  
 
 The subscript for the currently selected node (this is an internal 
 thing, so the user is unaware of this).
 
 
 
 
 
 
m_highlightNode 
 
int  m_highlightNode  
 
 The Node the user is currently focused on , this is similar to 
 focus node except that it is used by other 
 classes rather than this one.
 
 
 
 
 
 
m_listener 
 
 TreeDisplayListener   m_listener  
 
 
 
 
 
 
m_searchString 
 
javax.swing.JTextField  m_searchString  
 
 
 
 
 
 
m_searchWin 
 
javax.swing.JDialog  m_searchWin  
 
 
 
 
 
 
m_caseSen 
 
javax.swing.JRadioButton  m_caseSen  
 
 
 
 
 
 
m_FontColor 
 
java.awt.Color  m_FontColor  
 
 the font color.
 
 
 
 
 
 
m_BackgroundColor 
 
java.awt.Color  m_BackgroundColor  
 
 the background color.
 
 
 
 
 
 
m_NodeColor 
 
java.awt.Color  m_NodeColor  
 
 the node color.
 
 
 
 
 
 
m_LineColor 
 
java.awt.Color  m_LineColor  
 
 the line color.
 
 
 
 
 
 
m_ZoomBoxColor 
 
java.awt.Color  m_ZoomBoxColor  
 
 the color of the zoombox.
 
 
 
 
 
 
m_ZoomBoxXORColor 
 
java.awt.Color  m_ZoomBoxXORColor  
 
 the XOR color of the zoombox.
 
 
 
 
 
 
m_ShowBorder 
 
boolean  m_ShowBorder  
 
 whether to show the border or not.
 
 
 
 
 

 
 
  
 Package   weka.gui.visualize   
 
 

 
   
 
 
  
 Class  weka.gui.visualize.AttributePanel  extends javax.swing.JScrollPane implements Serializable   
 
 

 
 serialVersionUID:  3533330317806757814L

 
   
 
 
  
 Serialized Fields   
 
 

 
m_plotInstances 
 
 Instances   m_plotInstances  
 
 The instances to be plotted
 
 
 
 
 
 
m_maxC 
 
double  m_maxC  
 
 Holds the min and max values of the colouring attributes
 
 
 
 
 
 
m_minC 
 
double  m_minC  
 
 
 
 
 
 
m_cIndex 
 
int  m_cIndex  
 
 
 
 
 
 
m_xIndex 
 
int  m_xIndex  
 
 
 
 
 
 
m_yIndex 
 
int  m_yIndex  
 
 
 
 
 
 
m_colorList 
 
 FastVector   m_colorList  
 
 The colour map to use for colouring points
 
 
 
 
 
 
m_DefaultColors 
 
java.awt.Color[]  m_DefaultColors  
 
 default colours for colouring discrete class
 
 
 
 
 
 
m_backgroundColor 
 
java.awt.Color  m_backgroundColor  
 
 If set, it allows this panel to avoid setting a color in
 the color list that is equal to the background color
 
 
 
 
 
 
m_Listeners 
 
 FastVector   m_Listeners  
 
 The list of things listening to this panel
 
 
 
 
 
 
m_heights 
 
int[]  m_heights  
 
 Holds the random height for each instance.
 
 
 
 
 
 
m_span 
 
javax.swing.JPanel  m_span  
 
 The container window for the attribute bars, and also where the
 X,Y or B get printed.
 
 
 
 
 
 
m_barColour 
 
java.awt.Color  m_barColour  
 
 The default colour to use for the background of the bars if
      a colour is not defined in Visualize.props
 
 
 
 

 
   
 
 
  
 Class weka.gui.visualize.AttributePanel.AttributeSpacing extends javax.swing.JPanel implements Serializable   
 
 

 
 serialVersionUID:  7220615894321679898L

 
   
 
 
  
 Serialized Fields   
 
 

 
m_maxVal 
 
double  m_maxVal  
 
 The min and max values for this attribute.
 
 
 
 
 
 
m_minVal 
 
double  m_minVal  
 
 
 
 
 
 
m_attrib 
 
 Attribute   m_attrib  
 
 The attribute itself.
 
 
 
 
 
 
m_attribIndex 
 
int  m_attribIndex  
 
 The index for this attribute.
 
 
 
 
 
 
m_cached 
 
int[]  m_cached  
 
 The x position of each point.
 
 
 
 
 
 
m_pointDrawn 
 
boolean[][]  m_pointDrawn  
 
 A temporary array used to strike any instances that would be 
 drawn redundantly.
 
 
 
 
 
 
m_oldWidth 
 
int  m_oldWidth  
 
 Used to determine if the positions need to be recalculated.
 
 
 
 

 
   
 
 
  
 Class  weka.gui.visualize.ClassPanel  extends javax.swing.JPanel implements Serializable   
 
 

 
 serialVersionUID:  -7969401840501661430L

 
   
 
 
  
 Serialized Fields   
 
 

 
m_isEnabled 
 
boolean  m_isEnabled  
 
 True when the panel has been enabled (ie after 
      setNumeric or setNominal has been called
 
 
 
 
 
 
m_isNumeric 
 
boolean  m_isNumeric  
 
 True if the colouring attribute is numeric
 
 
 
 
 
 
m_spectrumHeight 
 
int  m_spectrumHeight  
 
 The height of the spectrum for numeric class
 
 
 
 
 
 
m_maxC 
 
double  m_maxC  
 
 The maximum value for the colouring attribute
 
 
 
 
 
 
m_minC 
 
double  m_minC  
 
 The minimum value for the colouring attribute
 
 
 
 
 
 
m_tickSize 
 
int  m_tickSize  
 
 The size of the ticks
 
 
 
 
 
 
m_labelMetrics 
 
java.awt.FontMetrics  m_labelMetrics  
 
 Font metrics
 
 
 
 
 
 
m_labelFont 
 
java.awt.Font  m_labelFont  
 
 The font used in labeling
 
 
 
 
 
 
m_HorizontalPad 
 
int  m_HorizontalPad  
 
 The amount of space to leave either side of the legend
 
 
 
 
 
 
m_precisionC 
 
int  m_precisionC  
 
 The precision with which to display real values
 
 
 
 
 
 
m_fieldWidthC 
 
int  m_fieldWidthC  
 
 Field width for numeric values
 
 
 
 
 
 
m_oldWidth 
 
int  m_oldWidth  
 
 The old width.
 
 
 
 
 
 
m_Instances 
 
 Instances   m_Instances  
 
 Instances being plotted
 
 
 
 
 
 
m_cIndex 
 
int  m_cIndex  
 
 Index of the colouring attribute
 
 
 
 
 
 
m_colorList 
 
 FastVector   m_colorList  
 
 the list of colours to use for colouring nominal attribute labels
 
 
 
 
 
 
m_Repainters 
 
 FastVector   m_Repainters  
 
 An optional list of Components that use the colour list
      maintained by this class. If the user changes a colour
      using the colour chooser, then these components need to
      be repainted in order to display the change
 
 
 
 
 
 
m_ColourChangeListeners 
 
 FastVector   m_ColourChangeListeners  
 
 An optional list of listeners who want to know when a colour
      changes. Listeners are notified via an ActionEvent
 
 
 
 
 
 
m_DefaultColors 
 
java.awt.Color[]  m_DefaultColors  
 
 default colours for colouring discrete class
 
 
 
 
 
 
m_backgroundColor 
 
java.awt.Color  m_backgroundColor  
 
 if set, it allows this panel to steer away from setting up
 a color in the color list that is equal to the background color
 
 
 
 

 
   
 
 
  
 Class  weka.gui.visualize.LegendPanel  extends javax.swing.JScrollPane implements Serializable   
 
 

 
 serialVersionUID:  -1262384440543001505L

 
   
 
 
  
 Serialized Fields   
 
 

 
m_plots 
 
 FastVector   m_plots  
 
 the list of plot elements
 
 
 
 
 
 
m_span 
 
javax.swing.JPanel  m_span  
 
 the panel that contains the legend entries
 
 
 
 
 
 
m_Repainters 
 
 FastVector   m_Repainters  
 
 a list of components that need to be repainted when a colour is
      changed
 
 
 
 

 
   
 
 
  
 Class weka.gui.visualize.LegendPanel.LegendEntry extends javax.swing.JPanel implements Serializable   
 
 

 
 serialVersionUID:  3879990289042935670L

 
   
 
 
  
 Serialized Fields   
 
 

 
m_plotData 
 
 PlotData2D   m_plotData  
 
 the data for this legend entry
 
 
 
 
 
 
m_dataIndex 
 
int  m_dataIndex  
 
 the index (in the list of plots) of the data for this legend---
        used to draw the correct shape for this data
 
 
 
 
 
 
m_legendText 
 
javax.swing.JLabel  m_legendText  
 
 the text part of this legend
 
 
 
 
 
 
m_pointShape 
 
javax.swing.JPanel  m_pointShape  
 
 displays the point shape associated with this legend entry
 
 
 
 

 
   
 
 
  
 Class  weka.gui.visualize.MatrixPanel  extends javax.swing.JPanel implements Serializable   
 
 

 
 serialVersionUID:  -1232642719869188740L

 
   
 
 
  
 Serialized Fields   
 
 

 
m_plotsPanel 
 
weka.gui.visualize.MatrixPanel.Plot  m_plotsPanel  
 
 The that panel contains the actual matrix
 
 
 
 
 
 
m_cp 
 
 ClassPanel   m_cp  
 
 The panel that displays the legend of the colouring attribute
 
 
 
 
 
 
optionsPanel 
 
javax.swing.JPanel  optionsPanel  
 
 The panel that contains all the buttons and tools, i.e. resize, jitter bars and sub-sampling buttons etc
      on the bottom of the panel
 
 
 
 
 
 
jp 
 
javax.swing.JSplitPane  jp  
 
 Split pane for splitting the matrix and the buttons and bars
 
 
 
 
 
 
m_updateBt 
 
javax.swing.JButton  m_updateBt  
 
 The button that updates the display to reflect the changes made by the user. 
      E.g. changed attribute set for the matrix
 
 
 
 
 
 
m_selAttrib 
 
javax.swing.JButton  m_selAttrib  
 
 The button to display a window to select attributes
 
 
 
 
 
 
m_data 
 
 Instances   m_data  
 
 The dataset for which this panel will display the plot matrix for
 
 
 
 
 
 
m_attribList 
 
javax.swing.JList  m_attribList  
 
 The list for selecting the attributes to display the plot matrix
 
 
 
 
 
 
m_js 
 
javax.swing.JScrollPane  m_js  
 
 The scroll pane to scrolling the matrix
 
 
 
 
 
 
m_classAttrib 
 
javax.swing.JComboBox  m_classAttrib  
 
 The combo box to allow user to select the colouring attribute
 
 
 
 
 
 
m_plotSize 
 
javax.swing.JSlider  m_plotSize  
 
 The slider to adjust the size of the cells in the matrix
 
 
 
 
 
 
m_pointSize 
 
javax.swing.JSlider  m_pointSize  
 
 The slider to adjust the size of the datapoints
 
 
 
 
 
 
m_jitter 
 
javax.swing.JSlider  m_jitter  
 
 The slider to add jitter to the plots
 
 
 
 
 
 
rnd 
 
java.util.Random  rnd  
 
 For adding random jitter
 
 
 
 
 
 
jitterVals 
 
int[][]  jitterVals  
 
 Array containing precalculated jitter values
 
 
 
 
 
 
datapointSize 
 
int  datapointSize  
 
 This stores the size of the datapoint
 
 
 
 
 
 
m_resamplePercent 
 
javax.swing.JTextField  m_resamplePercent  
 
 The text area for percentage to resample data
 
 
 
 
 
 
m_resampleBt 
 
javax.swing.JButton  m_resampleBt  
 
 The label for resample percentage
 
 
 
 
 
 
m_rseed 
 
javax.swing.JTextField  m_rseed  
 
 Random seed for random subsample
 
 
 
 
 
 
m_plotSizeLb 
 
javax.swing.JLabel  m_plotSizeLb  
 
 Displays the current size beside the slider bar for cell size
 
 
 
 
 
 
m_pointSizeLb 
 
javax.swing.JLabel  m_pointSizeLb  
 
 Displays the current size beside the slider bar for point size
 
 
 
 
 
 
m_selectedAttribs 
 
int[]  m_selectedAttribs  
 
 This array contains the indices of the attributes currently selected
 
 
 
 
 
 
m_classIndex 
 
int  m_classIndex  
 
 This contains the index of the currently selected colouring attribute
 
 
 
 
 
 
m_points 
 
int[][]  m_points  
 
 This is a local array cache for all the instance values for faster rendering
 
 
 
 
 
 
m_pointColors 
 
int[]  m_pointColors  
 
 This is an array cache for the colour of each of the instances depending on the 
      colouring attribute. If the colouring attribute is nominal then it contains the 
      index of the colour in our colour list. Otherwise, for numeric colouring attribute,
      it contains the precalculated red component for each instance's colour
 
 
 
 
 
 
m_missing 
 
boolean[][]  m_missing  
 
 Contains true for each attribute value (only the selected attributes+class attribute) 
      that is  missing, for each instance.
      m_missing[i][j] == true if m_selectedAttribs[j] is missing in instance i. 
      m_missing[i][m_missing[].length-1] == true  if class value is missing in instance i.
 
 
 
 
 
 
m_type 
 
int[]  m_type  
 
 This array contains for the classAttribute:  
      m_type[0] = [type of attribute, nominal, string or numeric] 
      m_type[1] = [number of discrete values of nominal or string attribute  
      or same as m_type[0] for numeric attribute]
 
 
 
 
 
 
m_plotLBSizeD 
 
java.awt.Dimension  m_plotLBSizeD  
 
 Stores the maximum size for PlotSize label to keep it's size constant
 
 
 
 
 
 
m_pointLBSizeD 
 
java.awt.Dimension  m_pointLBSizeD  
 
 Stores the maximum size for PointSize label to keep it's size constant
 
 
 
 
 
 
m_colorList 
 
 FastVector   m_colorList  
 
 Contains discrete colours for colouring for nominal attributes
 
 
 
 
 
 
fontColor 
 
java.awt.Color  fontColor  
 
 color for the font used in column and row names
 
 
 
 
 
 
f 
 
java.awt.Font  f  
 
 font used in column and row names
 
 
 
 

 
   
 
 
  
 Class  weka.gui.visualize.Plot2D  extends javax.swing.JPanel implements Serializable   
 
 

 
 serialVersionUID:  -1673162410856660442L

 
   
 
 
  
 Serialized Fields   
 
 

 
m_axisColour 
 
java.awt.Color  m_axisColour  
 
 Default colour for the axis
 
 
 
 
 
 
m_backgroundColour 
 
java.awt.Color  m_backgroundColour  
 
 Default colour for the plot background
 
 
 
 
 
 
m_plots 
 
 FastVector   m_plots  
 
 The plots to display
 
 
 
 
 
 
m_masterPlot 
 
 PlotData2D   m_masterPlot  
 
 The master plot
 
 
 
 
 
 
m_masterName 
 
java.lang.String  m_masterName  
 
 The name of the master plot
 
 
 
 
 
 
m_plotInstances 
 
 Instances   m_plotInstances  
 
 The instances to be plotted
 
 
 
 
 
 
m_plotCompanion 
 
 Plot2DCompanion   m_plotCompanion  
 
 An optional "compainion" of the panel. If specified, this
      class will get to do its thing with our graphics context
      before we do any drawing. Eg. the visualize panel may need
      to draw polygons etc. before we draw plot axis and data points
 
 
 
 
 
 
m_InstanceInfo 
 
javax.swing.JFrame  m_InstanceInfo  
 
 For popping up text info on data points
 
 
 
 
 
 
m_InstanceInfoText 
 
javax.swing.JTextArea  m_InstanceInfoText  
 
 
 
 
 
 
m_colorList 
 
 FastVector   m_colorList  
 
 The list of the colors used
 
 
 
 
 
 
m_DefaultColors 
 
java.awt.Color[]  m_DefaultColors  
 
 default colours for colouring discrete class
 
 
 
 
 
 
m_xIndex 
 
int  m_xIndex  
 
 Indexes of the attributes to go on the x and y axis and the attribute
      to use for colouring and the current shape for drawing
 
 
 
 
 
 
m_yIndex 
 
int  m_yIndex  
 
 
 
 
 
 
m_cIndex 
 
int  m_cIndex  
 
 
 
 
 
 
m_sIndex 
 
int  m_sIndex  
 
 
 
 
 
 
m_maxX 
 
double  m_maxX  
 
 Holds the min and max values of the x, y and colouring attributes 
   over all plots
 
 
 
 
 
 
m_minX 
 
double  m_minX  
 
 
 
 
 
 
m_maxY 
 
double  m_maxY  
 
 
 
 
 
 
m_minY 
 
double  m_minY  
 
 
 
 
 
 
m_maxC 
 
double  m_maxC  
 
 
 
 
 
 
m_minC 
 
double  m_minC  
 
 
 
 
 
 
m_axisPad 
 
int  m_axisPad  
 
 Axis padding
 
 
 
 
 
 
m_tickSize 
 
int  m_tickSize  
 
 Tick size
 
 
 
 
 
 
m_XaxisStart 
 
int  m_XaxisStart  
 
 the offsets of the axes once label metrics are calculated
 
 
 
 
 
 
m_YaxisStart 
 
int  m_YaxisStart  
 
 
 
 
 
 
m_XaxisEnd 
 
int  m_XaxisEnd  
 
 
 
 
 
 
m_YaxisEnd 
 
int  m_YaxisEnd  
 
 
 
 
 
 
m_plotResize 
 
boolean  m_plotResize  
 
 if the user resizes the window, or the attributes selected for
      the attributes change, then the lookup table for points needs
      to be recalculated
 
 
 
 
 
 
m_axisChanged 
 
boolean  m_axisChanged  
 
 if the user changes attribute assigned to an axis
 
 
 
 
 
 
m_drawnPoints 
 
int[][]  m_drawnPoints  
 
 An array used to show if a point is hidden or not.
 This is used for speeding up the drawing of the plot panel
 although I am not sure how much performance this grants over
 not having it.
 
 
 
 
 
 
m_labelFont 
 
java.awt.Font  m_labelFont  
 
 Font for labels
 
 
 
 
 
 
m_labelMetrics 
 
java.awt.FontMetrics  m_labelMetrics  
 
 
 
 
 
 
m_JitterVal 
 
int  m_JitterVal  
 
 the level of jitter
 
 
 
 
 
 
m_JRand 
 
java.util.Random  m_JRand  
 
 random values for perterbing the data points
 
 
 
 
 
 
m_pointLookup 
 
double[][]  m_pointLookup  
 
 lookup table for plotted points
 
 
 
 

 
   
 
 
  
 Class weka.gui.visualize.PrintableComponent.JComponentWriterFileFilter extends  ExtensionFileFilter  implements Serializable   
 
 

 
   
 
 
  
 Serialized Fields   
 
 

 
m_Writer 
 
 JComponentWriter   m_Writer  
 
 the associated writer.
 
 
 
 

 
   
 
 
  
 Class  weka.gui.visualize.PrintablePanel  extends javax.swing.JPanel implements Serializable   
 
 

 
 serialVersionUID:  6281532227633417538L

 
   
 
 
  
 Serialized Fields   
 
 

 
m_Printer 
 
 PrintableComponent   m_Printer  
 
 the class responsible for printing
 
 
 
 

 
   
 
 
  
 Class  weka.gui.visualize.ThresholdVisualizePanel  extends  VisualizePanel  implements Serializable   
 
 

 
 serialVersionUID:  3070002211779443890L

 
   
 
 
  
 Serialized Fields   
 
 

 
m_ROCString 
 
java.lang.String  m_ROCString  
 
 The string to add to the Plot Border.
 
 
 
 
 
 
m_savePanelBorderText 
 
java.lang.String  m_savePanelBorderText  
 
 Original border text
 
 
 
 

 
   
 
 
  
 Class  weka.gui.visualize.VisualizePanel  extends  PrintablePanel  implements Serializable   
 
 

 
 serialVersionUID:  240108358588153943L

 
   
 
 
  
 Serialized Fields   
 
 

 
m_DefaultColors 
 
java.awt.Color[]  m_DefaultColors  
 
 default colours for colouring discrete class
 
 
 
 
 
 
m_XCombo 
 
javax.swing.JComboBox  m_XCombo  
 
 Lets the user select the attribute for the x axis
 
 
 
 
 
 
m_YCombo 
 
javax.swing.JComboBox  m_YCombo  
 
 Lets the user select the attribute for the y axis
 
 
 
 
 
 
m_ColourCombo 
 
javax.swing.JComboBox  m_ColourCombo  
 
 Lets the user select the attribute to use for colouring
 
 
 
 
 
 
m_ShapeCombo 
 
javax.swing.JComboBox  m_ShapeCombo  
 
 Lets the user select the shape they want to create for instance 
 selection.
 
 
 
 
 
 
m_submit 
 
javax.swing.JButton  m_submit  
 
 Button for the user to enter the splits.
 
 
 
 
 
 
m_cancel 
 
javax.swing.JButton  m_cancel  
 
 Button for the user to remove all splits.
 
 
 
 
 
 
m_openBut 
 
javax.swing.JButton  m_openBut  
 
 Button for the user to open the visualized set of instances
 
 
 
 
 
 
m_saveBut 
 
javax.swing.JButton  m_saveBut  
 
 Button for the user to save the visualized set of instances
 
 
 
 
 
 
COMBO_SIZE 
 
java.awt.Dimension  COMBO_SIZE  
 
 Stop the combos from growing out of control
 
 
 
 
 
 
m_FileChooser 
 
javax.swing.JFileChooser  m_FileChooser  
 
 file chooser for saving instances
 
 
 
 
 
 
m_ArffFilter 
 
javax.swing.filechooser.FileFilter  m_ArffFilter  
 
 Filter to ensure only arff files are selected
 
 
 
 
 
 
m_JitterLab 
 
javax.swing.JLabel  m_JitterLab  
 
 Label for the jitter slider
 
 
 
 
 
 
m_Jitter 
 
javax.swing.JSlider  m_Jitter  
 
 The jitter slider
 
 
 
 
 
 
m_plot 
 
weka.gui.visualize.VisualizePanel.PlotPanel  m_plot  
 
 The panel that displays the plot
 
 
 
 
 
 
m_attrib 
 
 AttributePanel   m_attrib  
 
 The panel that displays the attributes , using color to represent 
 another attribute.
 
 
 
 
 
 
m_legendPanel 
 
 LegendPanel   m_legendPanel  
 
 The panel that displays legend info if there is more than one plot
 
 
 
 
 
 
m_plotSurround 
 
javax.swing.JPanel  m_plotSurround  
 
 Panel that surrounds the plot panel with a titled border
 
 
 
 
 
 
m_classSurround 
 
javax.swing.JPanel  m_classSurround  
 
 Panel that surrounds the class panel with a titled border
 
 
 
 
 
 
listener 
 
java.awt.event.ActionListener  listener  
 
 An optional listener that we will inform when ComboBox selections
      change
 
 
 
 
 
 
m_splitListener 
 
 VisualizePanelListener   m_splitListener  
 
 An optional listener that we will inform when the user creates a 
 split to seperate instances.
 
 
 
 
 
 
m_plotName 
 
java.lang.String  m_plotName  
 
 The name of the plot (not currently displayed, but can be used
      in the containing Frame or Panel)
 
 
 
 
 
 
m_classPanel 
 
 ClassPanel   m_classPanel  
 
 The panel that displays the legend for the colouring attribute
 
 
 
 
 
 
m_colorList 
 
 FastVector   m_colorList  
 
 The list of the colors used
 
 
 
 
 
 
m_preferredXDimension 
 
java.lang.String  m_preferredXDimension  
 
 These hold the names of preferred columns to visualize on---if the
      user has defined them in the Visualize.props file
 
 
 
 
 
 
m_preferredYDimension 
 
java.lang.String  m_preferredYDimension  
 
 
 
 
 
 
m_preferredColourDimension 
 
java.lang.String  m_preferredColourDimension  
 
 
 
 
 
 
m_showAttBars 
 
boolean  m_showAttBars  
 
 Show the attribute bar panel
 
 
 
 
 
 
m_showClassPanel 
 
boolean  m_showClassPanel  
 
 Show the class panel
 
 
 
 
 
 
m_Log 
 
 Logger   m_Log  
 
 the logger
 
 
 
 

 
   
 
 
  
 Class weka.gui.visualize.VisualizePanel.PlotPanel extends  PrintablePanel  implements Serializable   
 
 

 
 serialVersionUID:  -4823674171136494204L

 
   
 
 
  
 Serialized Fields   
 
 

 
m_plot2D 
 
 Plot2D   m_plot2D  
 
 The actual generic plotting panel
 
 
 
 
 
 
m_plotInstances 
 
 Instances   m_plotInstances  
 
 The instances from the master plot
 
 
 
 
 
 
m_originalPlot 
 
 PlotData2D   m_originalPlot  
 
 The master plot
 
 
 
 
 
 
m_xIndex 
 
int  m_xIndex  
 
 Indexes of the attributes to go on the x and y axis and the attribute
        to use for colouring and the current shape for drawing
 
 
 
 
 
 
m_yIndex 
 
int  m_yIndex  
 
 
 
 
 
 
m_cIndex 
 
int  m_cIndex  
 
 
 
 
 
 
m_sIndex 
 
int  m_sIndex  
 
 
 
 
 
 
m_XaxisStart 
 
int  m_XaxisStart  
 
 the offsets of the axes once label metrics are calculated
 
 
 
 
 
 
m_YaxisStart 
 
int  m_YaxisStart  
 
 
 
 
 
 
m_XaxisEnd 
 
int  m_XaxisEnd  
 
 
 
 
 
 
m_YaxisEnd 
 
int  m_YaxisEnd  
 
 
 
 
 
 
m_createShape 
 
boolean  m_createShape  
 
 True if the user is currently dragging a box.
 
 
 
 
 
 
m_shapes 
 
 FastVector   m_shapes  
 
 contains all the shapes that have been drawn for these attribs
 
 
 
 
 
 
m_shapePoints 
 
 FastVector   m_shapePoints  
 
 contains the points of the shape currently being drawn.
 
 
 
 
 
 
m_newMousePos 
 
java.awt.Dimension  m_newMousePos  
 
 contains the position of the mouse (used for rubberbanding).
 
 
 
 

 
 


 
   
  
 
 
 
   
 
   
          Overview     
        Package   
        Class   
          Tree     
          Deprecated     
          Index     
          Help           Weka's home     
   
 
 
  
 
 
 

 
  
 PREV 
 NEXT  
  
    FRAMES     
   NO FRAMES     
  
    All Classes  ');
  }
  //-->
 
 
    All Classes
